# Discovery and systematic characterization of risk variants and genes for coronary artery disease in over a million participants

**DOI:** 10.1038/s41588-022-01233-6

**Published:** 2022-12-06

**Authors:** Krishna G. Aragam, Tao Jiang, Anuj Goel, Stavroula Kanoni, Brooke N. Wolford, Deepak S. Atri, Elle M. Weeks, Minxian Wang, George Hindy, Wei Zhou, Christopher Grace, Carolina Roselli, Nicholas A. Marston, Frederick K. Kamanu, Ida Surakka, Loreto Muñoz Venegas, Paul Sherliker, Satoshi Koyama, Kazuyoshi Ishigaki, Bjørn O. Åsvold, Michael R. Brown, Ben Brumpton, Paul S. de Vries, Olga Giannakopoulou, Panagiota Giardoglou, Daniel F. Gudbjartsson, Ulrich Güldener, Syed M. Ijlal Haider, Anna Helgadottir, Maysson Ibrahim, Adnan Kastrati, Thorsten Kessler, Theodosios Kyriakou, Tomasz Konopka, Ling Li, Lijiang Ma, Thomas Meitinger, Sören Mucha, Matthias Munz, Federico Murgia, Jonas B. Nielsen, Markus M. Nöthen, Shichao Pang, Tobias Reinberger, Gavin Schnitzler, Damian Smedley, Gudmar Thorleifsson, Moritz von Scheidt, Jacob C. Ulirsch, Biobank Japan, EPIC-CVD, John Danesh, David O. Arnar, Noël P. Burtt, Maria C. Costanzo, Jason Flannick, Kaoru Ito, Dong-Keun Jang, Yoichiro Kamatani, Amit V. Khera, Issei Komuro, Iftikhar J. Kullo, Luca A. Lotta, Christopher P. Nelson, Robert Roberts, Gudmundur Thorgeirsson, Unnur Thorsteinsdottir, Thomas R. Webb, Aris Baras, Johan L. M. Björkegren, Eric Boerwinkle, George Dedoussis, Hilma Holm, Kristian Hveem, Olle Melander, Alanna C. Morrison, Marju Orho-Melander, Loukianos S. Rallidis, Arno Ruusalepp, Marc S. Sabatine, Kari Stefansson, Pierre Zalloua, Patrick T. Ellinor, Martin Farrall, John Danesh, Christian T. Ruff, Hilary K. Finucane, Jemma C. Hopewell, Robert Clarke, Rajat M. Gupta, Jeanette Erdmann, Nilesh J. Samani, Heribert Schunkert, Hugh Watkins, Cristen J. Willer, Panos Deloukas, Sekar Kathiresan, Adam S. Butterworth, The CARDIoGRAMplusC4D Consortium, Paul S. de Vries, Moritz von Scheidt

**Affiliations:** 1 Cardiovascular Research Center, Massachusetts General Hospital, Boston, MA USA; 2 Center for Genomic Medicine, Massachusetts General Hospital, Boston, MA USA; 3 Cardiovascular Disease Initiative, Broad Institute of MIT and Harvard, Cambridge, MA USA; 4 Program in Medical and Population Genetics, Broad Institute of MIT and Harvard, Cambridge, MA USA; 5 BHF Cardiovascular Epidemiology Unit, Department of Public Health and Primary Care, University of Cambridge, Cambridge, UK; 6 Radcliffe Department of Medicine, Division of Cardiovascular Medicine, University of Oxford, Oxford, UK; 7 Wellcome Centre for Human Genetics, University of Oxford, Oxford, UK; 8 William Harvey Research Institute, Barts and the London School of Medicine and Dentistry, Queen Mary University of London, London, UK; 9 Department of Computational Medicine and Bioinformatics, University of Michigan, Ann Arbor, MI USA; 10 Divisions of Cardiovascular Medicine and Genetics, Brigham and Women’s Hospital, Harvard Medical School, Boston, MA USA; 11 Department of Population Medicine, Qatar University College of Medicine, Doha, Qatar; 12 Analytic and Translational Genetics Unit, Massachusetts General Hospital, Boston, MA USA; 13 Stanley Center for Psychiatric Research, Broad Institute of MIT and Harvard, Cambridge, MA USA; 14 TIMI Study Group, Division of Cardiovascular Medicine, Brigham and Women’s Hospital, Harvard Medical School, Boston, MA USA; 15 Department of Internal Medicine, Division of Cardiology, University of Michigan, Ann Arbor, MI USA; 16 Institute for Cardiogenetics, University of Lübeck, Lübeck, Germany; 17 German Research Center for Cardiovascular Research (DZHK), Partner Site Hamburg/Lübeck/Kiel, Lübeck, Germany; 18 Medical Research Council Population Health Research Unit, CTSU—Nuffield Department of Population Health, Medical Sciences Division, University of Oxford, Oxford, UK; 19 Laboratory for Cardiovascular Genomics and Informatics, RIKEN Center for Integrative Medical Sciences, Tsurumi-ku, Yokohama, Japan; 20 Laboratory for Statistical and Translational Genetics, RIKEN Center for Integrative Medical Sciences, Tsurumi-ku, Yokohama, Japan; 21 Department of Public Health and Nursing, K.G. Jebsen Center for Genetic Epidemiology, Norwegian University of Science and Technology, NTNU, Trondheim, Norway; 22 HUNT Research Centre, Norwegian University of Science and Technology, Levanger, Norway; 23 Department of Endocrinology, Clinic of Medicine, St. Olavs Hospital, Trondheim, Norway; 24 Human Genetics Center, Department of Epidemiology, Human Genetics, and Environmental Sciences, School of Public Health, The University of Texas Health Science Center at Houston, Houston, TX USA; 25 Department of Nutrition–Dietetics, School of Health Science and Education, Harokopio University, Athens, Greece; 26 deCODE Genetics/Amgen, Inc., Reykjavik, Iceland; 27 School of Engineering and Natural Sciences, University of Iceland, Reykjavik, Iceland; 28 German Heart Centre Munich, Department of Cardiology, Technical University of Munich, Munich, Germany; 29 CTSU—Nuffield Department of Population Health, Medical Sciences Division, University of Oxford, Oxford, UK; 30 German Research Center for Cardiovascular Research (DZHK e.V.), Partner Site Munich Heart Alliance, Munich, Germany; 31 Department of Genetics and Genomic Science, Icahn Institute for Genomics and Multiscale Biology, Icahn School of Medicine at Mount Sinai, New York, NY USA; 32 The Zena and Michael A. Wiener Cardiovascular Institute, Icahn School of Medicine at Mount Sinai, New York, NY USA; 33 Institute of Human Genetics, Helmholtz Zentrum München, German Research Center for Environmental Health, Neuherberg, Germany; 34 Klinikum Rechts der Isar, Institute of Human Genetics, Technical University of Munich, Munich, Germany; 35 School of Medicine and University Hospital Bonn, Institute of Human Genetics, University of Bonn, Bonn, Germany; 36 Program in Biological and Biomedical Sciences, Harvard Medical School, Boston, MA USA; 37 Harvard T.H.Chan School of Public Health, Boston, MA USA; 38 Department of Cardiac Surgery, Tartu University Hospital and Institute of Clinical Medicine, Tartu University, Tartu, Estonia; 39 Department of Genetics and Genomic Sciences, Institute of Genomics and Multiscale Biology, Icahn School of Medicine at Mount Sinai, New York, NY USA; 40 Integrated Cardio Metabolic Centre, Karolinska Institutet, Karolinska Universitetssjukhuset, Huddinge, Sweden; 41 Department of Clinical Sciences in Malmö, Lund University, Malmö, Sweden; 42 College of Medicine and Health Sciences, Khalifa University, Abu Dhabi, UAE; 43 Department of Cardiovascular Medicine, The University of Tokyo, Tokyo, Japan; 44 Faculty of Medicine, University of Iceland, Reykjavik, Iceland; 45 Department of Internal Medicine, Division of Cardiology, Landspitali—National University Hospital of Iceland, Hringbraut, Reykjavik, Iceland; 46 Division of Genetics and Genomics, Boston Children’s Hospital, Boston, MA USA; 47 Department of Computational Biology and Medical Sciences, Graduate School of Frontier Sciences, The University of Tokyo, Tokyo, Japan; 48 Department of Cardiovascular Medicine, Mayo Clinic, Rochester, MN USA; 49 Regeneron Genetics Center, Regeneron Pharmaceuticals, Tarrytown, NY USA; 50 Department of Cardiovascular Sciences and NIHR Leicester Biomedical Research Centre, University of Leicester, Glenfield Hospital, Leicester, UK; 51 Cardiovascular Genomics and Genetics, University of Arizona College of Medicin, Phoenix, AZ USA; 52 Clinical Gene Networks AB, Stockholm, Sweden; 53 Human Genome Sequencing Center, Baylor College of Medicine, Houston, TX USA; 54 Second Department of Cardiology, Medical School, National and Kapodistrian University of Athens, University General Hospital Attikon, Athens, Greece; 55 National Institute for Health and Care Research Cambridge Biomedical Research Centre, Cambridge University Hospitals, Cambridge, UK; 56 The National Institute for Health and Care Research Blood and Transplant Unit (NIHR BTRU) in Donor Health and Genomics, University of Cambridge, Cambridge, UK; 57 Human Genetics, Wellcome Sanger Institute, Saffron Walden, UK; 58 Health Data Research UK Cambridge, Wellcome Genome Campus and University of Cambridge, Cambridge, UK; 59 British Heart Foundation Centre of Research Excellence, Division of Cardiovascular Medicine, Addenbrooke’s Hospital, Cambridge, UK; 60 Department of Human Genetics, University of Michigan, Ann Arbor, MI USA; 61 Princess Al-Jawhara Al-Brahim Centre of Excellence in Research of Hereditary Disorders (PACER-HD), King Abdulaziz University, Jeddah, Saudi Arabia; 62 Verve Therapeutics, Cambridge, MA USA

**Keywords:** Acute coronary syndromes, Genome-wide association studies

## Abstract

The discovery of genetic loci associated with complex diseases has outpaced the elucidation of mechanisms of disease pathogenesis. Here we conducted a genome-wide association study (GWAS) for coronary artery disease (CAD) comprising 181,522 cases among 1,165,690 participants of predominantly European ancestry. We detected 241 associations, including 30 new loci. Cross-ancestry meta-analysis with a Japanese GWAS yielded 38 additional new loci. We prioritized likely causal variants using functionally informed fine-mapping, yielding 42 associations with less than five variants in the 95% credible set. Similarity-based clustering suggested roles for early developmental processes, cell cycle signaling and vascular cell migration and proliferation in the pathogenesis of CAD. We prioritized 220 candidate causal genes, combining eight complementary approaches, including 123 supported by three or more approaches. Using CRISPR–Cas9, we experimentally validated the effect of an enhancer in *MYO9B*, which appears to mediate CAD risk by regulating vascular cell motility. Our analysis identifies and systematically characterizes >250 risk loci for CAD to inform experimental interrogation of putative causal mechanisms for CAD.

## Abstract (web summary)

Genome-wide association analyses, functionally informed fine-mapping and complementary gene prioritization approaches identify new risk loci and candidate effector genes for CAD.

## Main

Coronary artery disease (CAD) remains the leading global cause of mortality, reflecting both risk behaviors and genetic susceptibility^1^. Genetic association studies have identified >200 susceptibility loci for CAD. Consistent with other complex diseases, genetic analyses have identified the polygenic architecture of CAD, enabled insights into disease etiology and facilitated the development of new tools for risk prediction^2–10^. However, with rapid increase in the availability of genetic data linked to health outcomes, the identification of disease-associated loci has outpaced their functional characterization.

Several in silico tools have emerged to elucidate the mechanisms connecting genomic regions to disease risk^11,12^. Nonetheless, it remains challenging to identify causal genes as these tools frequently lack consensus^13^. Recent analyses have suggested the value of integrating ‘locus-based’ approaches with more global (similarity-based) assessments of shared pathways and functions to enhance the prediction of causal genes^13–15^. The use of orthogonal and disease-specific resources to aid variant and gene classifications may expedite the transition from gene maps to disease mechanisms.

To extend these approaches to CAD, we analyzed imputed data from nine studies not previously included in genome-wide association study (GWAS) meta-analyses (86,847 cases and 417,789 controls) and combined results with data from UK Biobank, the CARDIoGRAMplusC4D Consortium and Biobank Japan, achieving a total sample of 210,842 CAD cases among 1,378,170 participants^2,3,7,10,16^. Our objectives were to (1) discover new associations with CAD; (2) determine the impact of expanded genetic discovery for identifying biologically relevant loci and improving risk prediction; (3) implement a systematic, integrative approach to prioritize likely causal variants, genes and biological pathways, thereby providing a catalog of testable hypotheses for experimental follow-up and (4) experimentally validate a new locus as proof of principle for our prioritization framework.

## Results

### Discovery of known and new CAD loci

**Extended Data Fig. 1:**
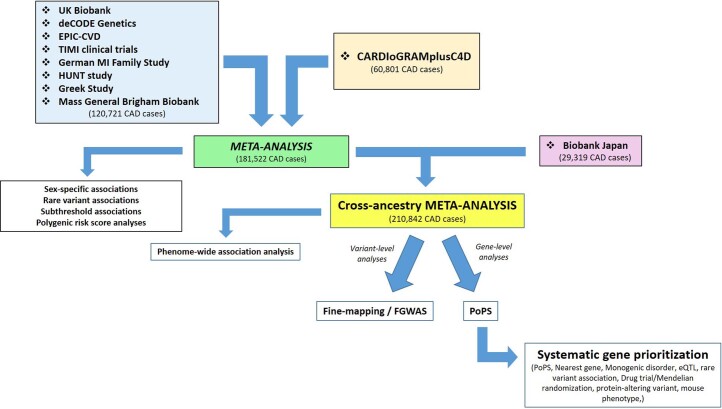
Study design. Flowchart depicting contributing studies and analysis strategy.

Participants were largely (>95%) of European ancestry and 46% were female (Supplementary Table 1). In total, 20,073,070 variants were included in the discovery meta-analysis (Online Methods). We replicated 150 (69.4%) of 216 previously reported CAD loci at conventional genome-wide significance (*P* ≤ 5.0 × 10^−8^) and 38 (17.6%) at nominal significance (*P* ≤ 1.0 × 10^−5^; Supplementary Table 2). Approximate conditional analysis using Genome-wide Complex Trait Analysis (GCTA) identified 241 conditionally independent associations exceeding genome-wide significance at 198 loci (Supplementary Table 3, Extended Data Fig. 1 and Supplementary Data 1). In total, 54 sentinel variants were new, including 30 outside genomic regions previously reported for CAD (Table 1).

**Table 1:** New loci for CAD from primary meta-analysis

Nearest gene	Lead variant rsID	Chr	Position	Effect allele	Non-effect allele	Odds ratio	95% CI	*P* value
*KDF1*	rs79598313	1	27,284,913	T	C	1.10	1.06–1.14	3.6 × 10^−8^
*LOC100131060*	rs71646019	1	59,433,354	T	C	1.04	1.03–1.05	6.1 × 10^−10^
*OTUD7B*	rs67807996	1	149,995,265	A	G	1.04	1.03–1.05	1.1 × 10^−12^
*MIR4432*	rs243071	2	60,619,028	A	G	1.03	1.02–1.04	2.7 × 10^−8^
*SAP130*	rs114192718	2	128,785,663	T	C	1.06	1.04–1.08	2.6 × 10^−8^
*ACVR2A*	rs35611688	2	148,377,860	T	C	0.97	0.96–0.98	1.5 × 10^−8^
*LNX1*	rs17083333	4	54,572,066	T	G	0.97	0.96–0.98	1.2 × 10^−8^
*ITGA1*	rs4074793	5	52,193,125	A	G	0.95	0.93–0.97	1.6 × 10^−8^
*FER*	rs112949822	5	108,085,190	A	G	0.95	0.93–0.96	1.1 × 10^−9^
*DMXL1*	rs13169691	5	118,448,279	T	C	1.04	1.03–1.06	2.6 × 10^−8^
*FBN2*	rs6883598	5	127,926,190	A	C	0.97	0.96–0.98	9.7 × 10^−10^
*PTK7*	rs1034246	6	43,068,370	T	G	0.97	0.96–0.98	6.4 × 10^−10^
*MACC1*	rs10486389	7	20,300,416	A	G	0.97	0.96–0.98	6.5 × 10^−10^
*C9orf146*	rs10961206	9	13,724,051	A	T	1.05	1.04–1.07	8.1 × 10^−10^
*ACER2*	rs10811183	9	19,436,055	A	G	1.04	1.02–1.05	1.6 × 10^−8^
*C5*	rs41312891	9	123,726,749	G	GCAAA	0.94	0.92–0.96	5.9 × 10^−9^
*PLCE1*	rs55753709	10	96,029,170	T	C	0.96	0.95–0.97	2.2 × 10^−13^
*R3HCC1L*	rs884811	10	99,923,763	C	G	1.03	1.02–1.04	3.1 × 10^−9^
*MMP13*	rs1892971	11	102,795,606	A	G	0.96	0.95–0.97	5.1 × 10^−10^
*ST3GAL4*	rs10790800	11	126,262,638	A	G	1.03	1.02–1.04	9.1 × 10^−9^
*TBX3*	rs34606058	12	115,353,368	T	C	0.97	0.96–0.98	7.7 × 10^−9^
*DOCK9*	rs8000794	13	99,434,810	C	G	1.03	1.02–1.04	4.3 × 10^−8^
*LIPC*	rs588136	15	58,730,498	T	C	0.96	0.95–0.98	7.0 × 10^−10^
*UNC13D*	rs2410859	17	73,841,285	T	C	1.03	1.02–1.04	4.3 × 10^−9^
*CPLX4*	rs11663411	18	56,960,510	T	C	0.97	0.96–0.98	2.6 × 10^−8^
*MYO9B*	rs7246865	19	17,219,105	A	G	1.03	1.02–1.05	1.9 × 10^−8^
*RRBP1*	rs1132274	20	17,596,155	A	C	1.04	1.03–1.05	1.8 × 10^−8^
*MAFB*	rs2207132	20	39,142,516	A	G	1.10	1.07–1.13	6.7 × 10^−10^
*ARVCF*	rs71313931	22	19,960,184	C	G	0.97	0.96–0.98	2.3 × 10^−9^
*SCUBE1*	rs139012	22	43,623,972	A	G	0.97	0.96–0.98	2.1 × 10^−8^

Positions are according to GRCh37. Odds ratios (and 95% confidence intervals (CIs)) are for per-allele effect estimates according to the effect allele. Two-sided *P* values are from *Z* scores from a fixed-effect inverse-variance weighted meta-analysis.

As in previous CAD GWAS^9^, we found genetic correlations with several CAD risk factors and other cardiovascular diseases (Supplementary Table 4). To identify potential etiological mechanisms for specific loci, we conducted a phenome-wide association scan (PheWAS) in UK Biobank (Supplementary Table 5). In total, 128 (53%) of the CAD-associated variants had directionally consistent associations with conventional CAD risk factors, such as blood lipids, blood pressure, hyperglycemia or adiposity.

Several new associations (Table 1) were near genes that have not been robustly implicated in CAD via genetic association studies but have strong biological plausibility, including rs6883598 near *FBN2*, encoding fibrillin-2, which mediates the early stages of elastic fiber assembly and is associated with aortic aneurysms and Beals Syndrome, a Marfan-like disorder^17–19^ and rs1892971 near *MMP13*, which encodes matrix metalloproteinase (MMP)-13, an interstitial collagenase that influences the structural integrity of atherosclerotic plaques through regulation and organization of intraplaque collagen^20,21^. While the sentinel variant near *FBN2* was associated with blood pressure in the PheWAS, the lead variant near *MMP13* was not associated with conventional CAD risk factors, suggesting it is likely to act through alternative pathways.

### Allelic architecture

**Fig. 1:**
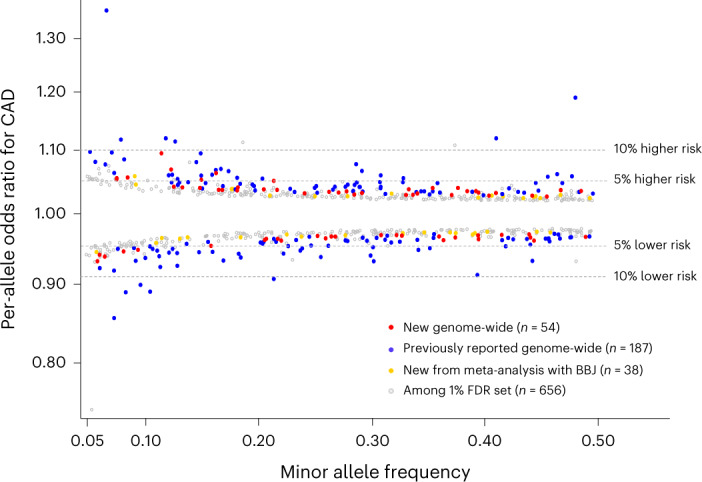
Common variant association signals for CAD. MAF versus per-allele OR for CAD for common sentinel variant (MAF > 5%) associations reaching genome-wide significance or the 1% FDR threshold in our study. Colored circles indicate genome-wide significant associations (*P* < 5.0 × 10^−8^) with sentinel variants that are not correlated (*r*^2^ < 0.2) with a previously reported variant (red), genome-wide significant sentinel variants correlated with a previously reported variant (blue), new genome-wide significant sentinels after meta-analysis with Biobank Japan (gold) and associations reaching the 1% FDR threshold (*P* < 2.52 × 10^−5^) in our meta-analysis (gray). Two-sided *P* values are from *Z* scores from fixed-effect inverse-variance weighted meta-analyses.

**Extended Data Fig. 2:**
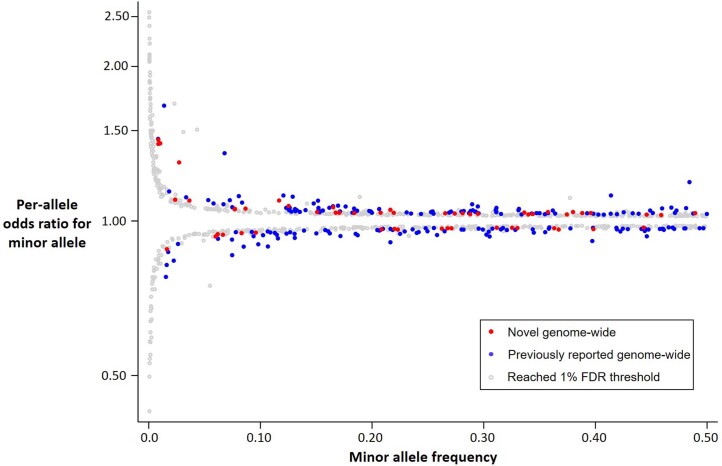
Genetic architecture of 897 association signals for CAD. Minor allele frequency versus per-allele odds ratio for CAD for all sentinel variants reaching genome-wide significance or the 1% FDR threshold in our study. Colored circles indicate genome-wide significant associations (*P* < 5.0 × 10^−8^) with sentinel variants that are not correlated (*r*^2^ < 0.2) with a previously reported variant (red), genome-wide significant sentinel variants correlated with a previously reported variant (blue), and associations reaching the 1% FDR threshold (*P* < 2.52 × 10^−5^) in our meta-analysis (gray). Two-sided *P* values are from *Z*-scores from fixed-effect inverse-variance weighted meta-analyses.

**Extended Data Fig. 3:**
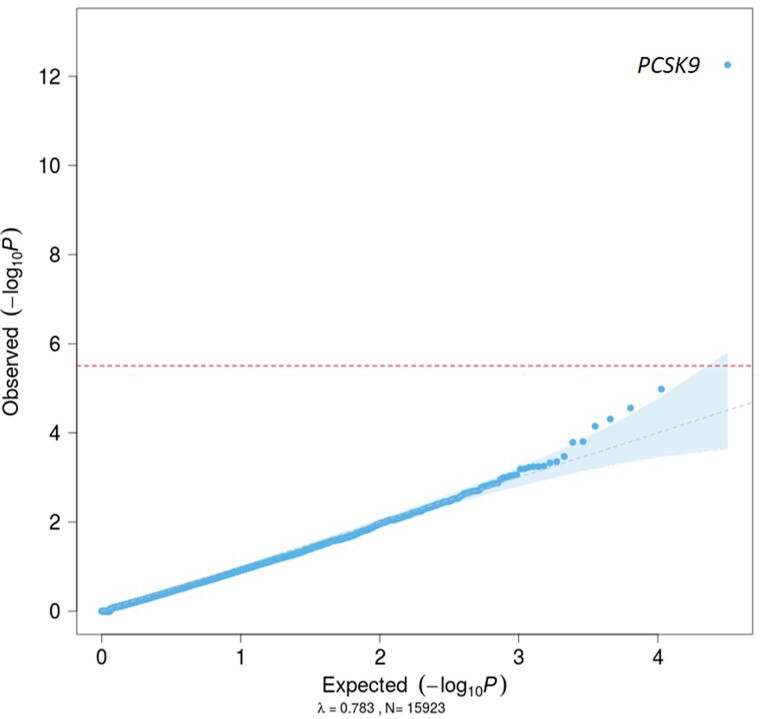
Gene-based association testing of rare variants in UK Biobank. QQ-plot of aggregate variant association tests from 15,923 genes versus CAD in UK Biobank. Results presented here are for the SKAT-O test using the Mask 1 (‘lenient’) filter, which includes variants with minor allele frequency < 5% that are annotated as missense, frameshift, stop gain, stop loss or splice site. Results for all genes, tests and filters are in Supplementary Table 7. Details of masks and test are in Supplementary Table 6. The red dashed line indicates the Bonferroni threshold accounting for the number of genes tested. The gray dashed line indicates the null hypothesis (that is observed = expected under the null). The blue shaded area indicates the 95% confidence interval around the null.

Of the 54 new associations, 46 sentinel variants were common (minor allele frequency (MAF) > 0.05) with relatively weak effects on CAD (odds ratio (OR) per CAD risk allele: 1.03–1.07; Fig. 1). The others were low frequency (MAF = 0.009–0.036) of which, four had comparatively strong effects (OR = 1.30–1.44) and four had more modest effects (OR = 1.10–1.14; Extended Data Fig. 2). We then conducted gene-based tests of missense and predicted loss-of-function variants in UK Biobank (*n* = 33,941 CAD cases, 438,394 controls; Supplementary Table 6) and found a strong signal for *PCSK9*. We did not find evidence for further association with a burden of low-frequency or rare variants (Extended Data Fig. 3 and Supplementary Table 7).

### Differential effects by sex

To identify associations that differ by sex, we conducted sex-stratified GWAS in a subset of studies comprising 77,080 CAD cases (Supplementary Table 8). We found ten associations that reached genome-wide significance (*P* ≤ 5.0 × 10^−8^) and had evidence (*P* ≤ 0.01) for between-sex heterogeneity (Supplementary Table 9). Lead variant rs7696877 was the only signal with a stronger effect in females (per-allele OR = 0.94) than in males (per-allele OR = 0.98, heterogeneity *P* = 0.007).

### Subthreshold associations

At a significance level (*P* < 2.52 × 10^−5^) approximating a 1% false discovery rate (FDR), we identified a further 656 conditionally independent associations with CAD (Supplementary Table 10). Most (486, 74.1%) were common variants, but almost all had modest effects (per-allele OR < 1.07). Several associations had strong biological priors, including rs41279633 (*P* = 1.24 × 10^−6^) in *NPC1L1*, encoding Niemann-Pick C1-like 1, an important mediator of intestinal cholesterol absorption and the target of ezetimibe, a cholesterol-lowering drug. Other examples included *PNPLA3* (rs738408; *P* = 1.04 × 10^−5^), the strongest locus for nonalcoholic fatty liver disease^22^, and *TCF7L2* (rs7903146; *P* = 6.39 × 10^−8^), the strongest locus for type 2 diabetes^23^. The percent of heritability for CAD (on the liability scale) explained by the 241 conditionally independent associations reaching genome-wide significance was 15.5%, increasing to 36.1% for the 897 associations with *P* < 2.52 × 10^−5^.

### Polygenic score associations with incident and recurrent CAD

We evaluated 362 polygenic risk scores (PRS) using combinations of derivation methods (Pruning and Thresholding^24^ or LDpred algorithm^25^) and summary statistics (from the current meta-analysis or an earlier 1000 genomes-imputed GWAS involving around 60,000 CAD cases^7^). We selected the optimal PRS for each combination of the derivation method and GWAS summary statistics based on prediction of incident CAD in a training dataset from the Malmö Diet and Cancer study (MDC; *n* = 22,872; *n*_incident_cases_ = 3,307; Supplementary Table 11). The two top-performing scores were those derived with LDpred and comprised 2,324,653 variants (2022 PRS) and 1,532,758 variants (2015 PRS; Supplementary Tables 12–15). In bootstrapping analyses, the 2022 PRS outperformed the 2015 PRS (age- and sex-adjusted mean hazard ratio (HR) per 1 s.d. higher PRS = 1.56 versus 1.49; *P* = 3.2 × 10^−31^; age- and sex-adjusted mean area under the receiver operator characteristic curve (AUC) = 0.742 versus 0.736; *P* = 6.5 × 10^−16^; Supplementary Table 16).

**Fig. 2:**
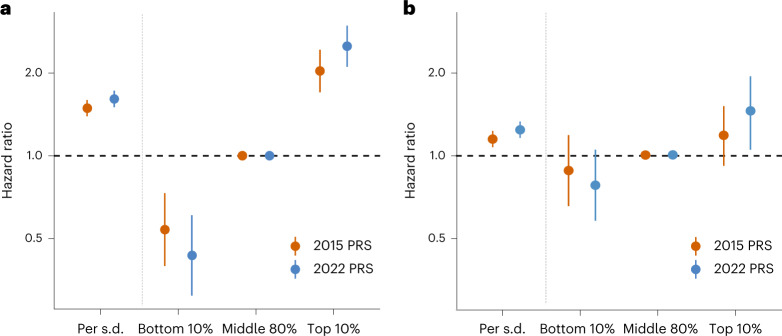
Polygenic prediction of incident and recurrent CAD. **a**,**b**, Prognostication of incident CAD (**a**) and recurrent coronary events (**b**) by optimal PRS derived from the current meta-analysis of ~180 K CAD cases (2022 PRS; includes ~2.3 million variants) or a previously reported GWAS meta-analysis of CAD from 2015 involving ~60 K CAD cases (2015 PRS; includes ~1.5 million variants). We analyzed 815 incident events in the validation subset of the MDC Study and 1,074 recurrent coronary events in the FOURIER trial. Cox proportional hazards models were adjusted for age, sex and genetic principal components.

We validated both scores in a held-out subset of the MDC (*n* = 5,685; *n*_incident_cases_ = 815; Supplementary Table 11). The 2022 PRS was more strongly associated with incident CAD (HR = 1.61; 95% CI = 1.50–1.72) than the 2015 PRS (HR = 1.49; 95% CI = 1.39–1.59), providing improved stratification of participants at higher and lower risk for incident CAD (Fig. 2a). After adjustment for established risk factors (Online Methods), the 2022 PRS remained strongly associated with incident events (HR = 1.54; 95% CI = 1.42–1.66). The 2022 PRS yielded a 5.7-fold higher risk of CAD between the top and bottom deciles of the PRS, compared to a 3.8-fold higher risk with the 2015 PRS.

We then evaluated prediction of recurrent coronary events in the placebo arm of the Further Cardiovascular Outcomes Research with *PCSK9* Inhibition in Subjects with Elevated Risk (FOURIER; *n* = 7,135; *n*_incident_cases_ = 673) clinical trial, a cohort of patients with established atherosclerotic cardiovascular disease^26^. The 2022 PRS demonstrated better recurrent event prediction (HR = 1.20; 95% CI = 1.11–1.29) than the 2015 PRS (HR = 1.13; 95% CI = 1.04–1.22) and enhanced stratification of participants at higher and lower risk (Fig. 2b). The 2022 PRS yielded a 1.7-fold higher risk of recurrent coronary events between the top and bottom deciles of the PRS versus a 1.4-fold higher risk with the 2015 PRS.

### Cross-ancestry comparison and meta-analysis

**Extended Data Fig. 4:**
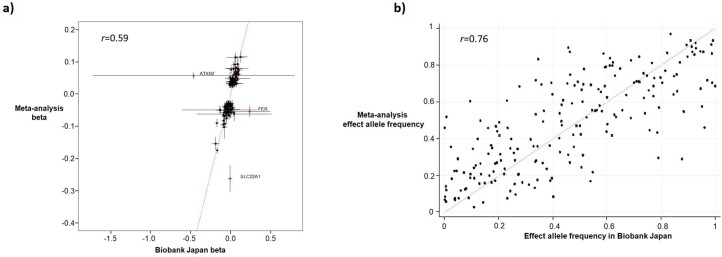
Cross-ancestry comparison. **a**, Comparison of allele frequencies between the meta-analysis and Biobank Japan. Black dots denote the allele frequencies for 199 sentinel variants reaching genome-wide significance in the (predominantly European ancestry) meta-analysis (*y*-axis) that were also present in the publicly available summary statistics from Biobank Japan (*x*-axis). Variants were aligned according to the effect allele in Supplementary Table 3. The Pearson correlation coefficient was 0.76. **b**, Comparison of beta estimates between the meta-analysis and Biobank Japan. Black dots denote the beta estimates for the CAD associations for 199 sentinel variants reaching genome-wide significance in the (predominantly European ancestry) meta-analysis (*y*-axis) that were also present in the publicly available summary statistics from Biobank Japan (*x*-axis). Variants were aligned according to the effect allele in Supplementary Table 3. Horizontal and vertical lines represent 95% confidence intervals. The Pearson correlation coefficient was 0.59, which increased to 0.85 when three outlying variants marked in red (at *ATXN2*, *FER* and *SLC22A1*) were excluded.

We used a large CAD GWAS from Biobank Japan to evaluate the genome-wide significant associations in East Asian ancestry participants^3^. Effect estimates for the 199 sentinel variants in both datasets were strongly positively correlated (*r* = 0.59) between the predominantly European ancestry meta-analysis and the Biobank Japan GWAS (Extended Data Fig. 4a), as were the effect allele frequencies (*r* = 0.76; Extended Data Fig. 4b). To assess the potential for enhanced, cross-ancestry discovery, we meta-analyzed the Biobank Japan summary statistics with the current analysis, yielding 38 additional new loci at genome-wide significance (Table 2, Fig. 1, and Supplementary Table 17). The sentinel variants were common (MAF > 5%) with weak effects (per-allele ORs: 1.026–1.059; Fig. 1), with the exception of rs75655731 near *LINC005999*, which was low-frequency (MAF = 1.4%) with a stronger effect (per-allele OR = 1.090); 36 of these associations were included in the 1% FDR set, including the aforementioned associations at *TCF7L2* and *PNPLA3*.

**Table 2:** New loci for CAD from meta-analysis with Biobank Japan

Nearest gene	Lead variant rsID	Chr	Position	Effect allele	Non-effect allele	Odds ratio	95% CI	*P* value
*CCDC30*	rs6656344	1	42,948,585	A	C	0.97	0.97–0.98	5.0 × 10^−9^
*KIAA0040*	rs2285219	1	175,130,983	A	T	1.03	1.02–1.04	4.0 × 10^−9^
*AAK1*	rs12468870	2	69,679,537	C	G	0.97	0.96–0.98	5.1 × 10^−10^
*CXCR4*	rs4954580	2	136,986,303	T	C	0.96	0.95–0.98	3.0 × 10^−9^
*PDE1A*	rs1430158	2	183,262,128	T	C	0.97	0.97–0.98	2.4 × 10^−8^
*ATP1B3*	rs7622417	3	141,625,999	C	G	1.03	1.02–1.03	1.2 × 10^−8^
*MECOM*	rs11721038	3	168,849,576	T	C	1.05	1.03–1.06	3.5 × 10^−9^
*GNPDA2*	rs12641981	4	45,179,883	T	C	1.03	1.02–1.04	2.0 × 10^−9^
*LOC285696*	rs2652682	5	17,113,657	A	T	0.97	0.96–0.98	1.0 × 10^−9^
*SKP2*	rs5867305^a^	5	36,157,262	CA	C	0.97	0.96–0.98	3.9 × 10^−8^
*SGCD*	rs157333	5	156,117,200	C	G	1.04	1.03–1.05	1.3 × 10^−11^
*TFAP2B*	rs62405422	6	50,796,905	T	C	0.97	0.96–0.98	3.1 × 10^−10^
*TRAF3IP2*–*AS1*	rs9400480	6	111,850,597	C	G	1.03	1.02–1.04	4.1 × 10^−8^
*HBS1L*	rs9399136	6	135,402,339	T	C	1.03	1.02–1.04	2.0 × 10^−8^
*PDE1C*	rs215634	7	32,369,148	A	G	1.03	1.02–1.04	1.2 × 10^−8^
*SEMA3C*	rs1019016	7	80,570,562	T	G	1.03	1.02–1.04	3.8 × 10^−10^
*ZKSCAN1*	rs6953441	7	99,617,067	A	G	1.03	1.02–1.04	6.8 × 10^−9^
*LINC00599*	rs75655731^a^	8	9,721,394	C	G	0.92	0.89–0.95	4.6 × 10^−8^
*DOCK8*	rs1536608	9	223,613	T	G	1.03	1.02–1.03	3.0 × 10^−8^
*GPSM1*	rs3935875	9	139,238,824	A	G	0.97	0.96–0.98	2.2 × 10^−8^
*ARHGAP21*	rs7077962	10	25,054,674	T	C	1.03	1.02–1.04	1.2 × 10^−8^
*NRP1*	rs75082222	10	33,516,373	T	TA	0.97	0.96–0.98	3.5 × 10^−8^
*TCF7L2*	rs7903146	10	114,758,349	T	C	1.03	1.02–1.04	6.2 × 10^−9^
*AFAP1L2*	rs646668	10	116,138,034	A	G	1.03	1.02–1.04	2.8 × 10^−9^
*PPP2R1B*	rs11410951	11	111,621,399	CA	C	0.97	0.96–0.98	2.6 × 10^−8^
*ACVRL1*	rs2277383	12	52,314,388	T	G	0.97	0.95–0.98	3.0 × 10^−8^
*CNPY2*	rs62956461	12	56,706,178	A	AT	1.06	1.04–1.08	2.7 × 10^−9^
*PAWR*	rs8176893	12	79,999,309	A	T	1.03	1.02–1.04	7.9 × 10^−10^
*CDK8*	rs12864131	13	27,045,939	A	G	0.97	0.97–0.98	1.1 × 10^−8^
*GP2*	rs10852238	16	20,253,374	A	T	1.04	1.03–1.05	4.1 × 10^−9^
*XPO6*	rs111806192	16	28,252,382	T	G	0.97	0.96–0.98	1.8 × 10^−8^
*DYNLRB2*	rs16952537	16	80,185,366	A	G	0.97	0.97–0.98	3.9 × 10^−8^
*PIP4K2B*	rs16968377	17	36,942,396	T	C	0.95	0.93–0.97	3.0 × 10^−8^
*TIMP2*	rs8075861	17	76,915,710	A	C	0.97	0.97–0.98	5.0 × 10^−9^
*WDR87*	rs73025613	19	38,334,361	T	C	1.03	1.02–1.03	4.4 × 10^−8^
*RRP1B*	rs35219138	21	45,117,913	A	AT	0.97	0.96–0.98	1.8 × 10^−8^
*SYN3*	rs4452	22	33,283,257	T	C	0.96	0.95–0.97	1.2 × 10^−8^
*PNPLA3*	rs738408	22	44,324,730	T	C	0.97	0.96–0.98	3.8 × 10^−9^

Positions are according to GRCh37. Odds ratios (and 95% CIs) are for per-allele effect estimates according to the effect allele. Two-sided *P* values are from *Z* scores from a fixed-effect inverse-variance weighted meta-analysis. ^a^Variants that did not reach 1% FDR threshold in primary meta-analysis.

### Prioritizing causal variants, genes and biological pathways

Using several independent approaches, we prioritized causal variants, effector genes, relevant tissues and intermediate causal pathways for all 279 significant associations. The presence of a protein-altering (that is, missense or predicted loss of function) variant has been shown to be a strong, causal gene predictor, particularly if the variant is uncommon^14^. At 52 associations, the sentinel variant, or a strong proxy (*r*^2^ ≥ 0.8), was a protein-altering variant (Supplementary Table 18). These included well-known low-frequency missense variants in *PCSK9* (p.R46L) and *ANGPTL4* (p.E40K)^16^. Nineteen of the 52 missense variants were new, including a missense variant (rs129415; p.G398R) in *SCUBE1* that is strongly correlated with the CAD sentinel variant (*r*^2^ = 0.99). *SCUBE1* encodes signal peptide-CUB-EGF domain-containing protein 1, a glycoprotein secreted by activated platelets that protect against thrombosis in mice when inhibited^27^.

### Functionally informed fine-mapping

**Fig. 3:**
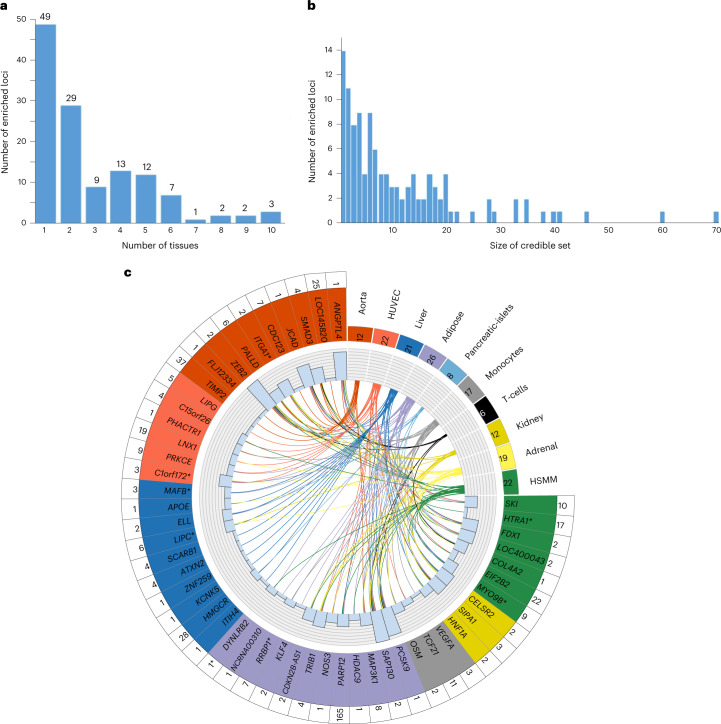
Epigenetic enrichment and functionally informed fine-mapping of CAD loci. **a**, Number of tissues/cell types in which 127 regions were enriched. Of 235 distance-based regions containing genome-wide significant associations in our meta-analysis, 127 regions had significant enrichment in at least one tissue type and were therefore fine-mapped using FGWAS. **b**, Distribution of 95% credible set sizes for the 127 enriched regions. For display purposes, the plot excludes ten regions for which the 95% credible set contained more than 100 variants (Supplementary Table 20). **c**, Circle plot of epigenetic enrichment for 53 significantly enriched GWAS regions containing a variant with PPA ≥ 0.5. The number of regions in which each tissue showed enrichment in is displayed in the upper right quadrant. The number of regions that show enrichment with a given tissue/cell type is displayed in the box next to the tissue/cell type name. The 53 significantly enriched GWAS regions containing a variant with PPA ≥ 0.5 are colored according to the tissue with the strongest evidence of enrichment for that region. Region names with an asterisk denote those for which all conditionally independent association signals were annotated as being new. The histogram shows the total number of tissues with enrichment for each region and the links indicate the tissues/cell types in which each region was enriched. The number of 95% credible variants per region is displayed in the outer ring.

Incorporating functional annotations into fine-mapping approaches has been shown to improve identification of causal variants^28–30^. Using ChromHMM-derived chromatin states from the NIH Roadmap Epigenomics Consortium to functionally annotate the genome, we found more than twofold enrichment for these states in the ten CAD-relevant cell/tissue types we tested, consistent with previous findings (Supplementary Table 19)^7^. Of 235 distance-based regions containing genome-wide significant associations, we found 127 (54.0%) with significant enrichment (Supplementary Table 20). The majority (78; 61.4%) of distance-based regions were relatively tissue specific, showing enrichment in less than three tissues, but eight regions showed widespread enrichment in seven or more tissues (Fig. 3a). Adipose (*n* = 33), liver (*n* = 26) and aorta (*n* = 21) showed the greatest enrichment for the most regions (Supplementary Table 20).

We applied a functionally informed fine-mapping method (functional genome-wide association analysis (FGWAS))^29^, which uses chromatin state enrichment information to reweight GWAS summary statistics and compute variant-specific posterior probabilities of association (PPA). Among the 127 enriched regions, we identified 42 that contained less than five variants in the 95% credible set (Fig. 3b and Supplementary Table 21), while 53 regions contained a variant with PPA ≥ 0.5 (Fig. 3c and Supplementary Table 22) showing that the combination of functional annotation and high statistical power can pinpoint likely causal variants. Indeed, 14 regions were fine-mapped to a single variant, including missense variants in *PCSK9*, *ANGPTL4* and *APOE*, plus other well-studied noncoding variants, such as rs9349379 (*PHACTR1*/*EDN1*)^31^ and rs2107595 (*HDAC9*/*TWIST1*)^32^.

**Extended Data Fig. 5:**
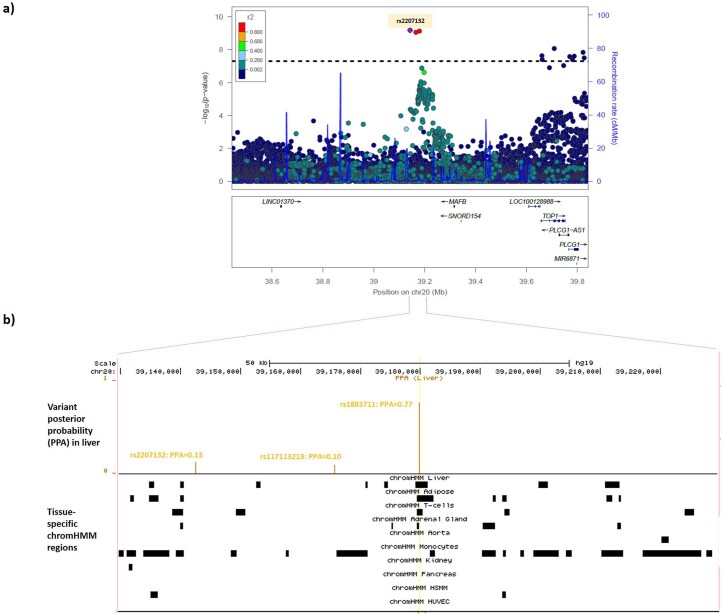
Epigenetically-informed fine-mapping of the *MAFB* locus. **a**, Regional association plot from the CAD meta-analysis for the *MAFB* region. Colored dots represent the position (*x*-axis) in GRCh37 coordinates and –log_10_(meta-analysis *P* value) (*y*-axis) of each variant in the region. Dots are shaded to represent the *r*^2^ with the lead CAD variant (rs2207132), estimated using a random sample of 5,000 European ancestry participants from the UK Biobank. Recombination peaks are plotted in blue based on estimates of recombination from 1000 Genomes European-ancestry individuals. **b**, Tissue-specific imputed chromHMM states at the three credible set variants in the *MAFB* region. The top track shows the position on chromosome 20 (GRCh37) in the *MAFB* region. The second track shows as orange vertical bars the posterior probability (*y*-axis) for each variant in the window from the FGWAS fine-mapping, identifying rs1883711 (PPA = 0.77) as the most likely causal variant. The third track indicates as a black box the position of the imputed chromHMM state in each of the ten CAD-relevant tissues based on epigenomic data from the NIH Roadmap Epigenomics Consortium project. The yellow vertical line indicates the position of the most likely causal variant (rs1883711) with respect to the chromHMM states. rs1883711 lies in an enhancer region for liver (the most strongly enriched tissue for this region) and adipose, the two functionally enriched tissues in the region. The other two variants in the 95% credible set (rs2207132 and rs117113213) do not lie in regions annotated as chromHMM states. HSMM, human skeletal muscle myoblasts; HUVEC, human umbilical vein endothelial cells; PPA, posterior probability of being the causal variant.

At 12 loci, fine-mapping prioritized (PPA ≥ 0.5) variants that were not the sentinel. For example, at the low-density lipoprotein (LDL) cholesterol and adiposity-associated *MAFB* locus^33^, the sentinel variant was rs2207132 (Supplementary Table 3 and Extended Data Fig. 5a). However, a strongly correlated variant (rs1883711; *r*^2^ = 0.92) lies in a region annotated as a likely enhancer in liver and adipose tissue, the two enriched tissues at this locus (Extended Data Fig. 5b). Therefore, rs1883711 was upweighted by FGWAS (PPA = 0.77) over rs2207132 (PPA = 0.13). We queried CAD-associated variants for *cis-*expression quantitative trait loci (*cis*-eQTLs) in CAD-relevant tissues from the Stockholm-Tartu Atherosclerosis Reverse Network Engineering Task (STARNET) and Genotype-Tissue Expression (GTEx) studies (Online Methods)^34,35^. The eQTL for *MAFB* observed in liver samples from CAD patients in STARNET suggests that the CAD association is mediated by changes in *MAFB* expression (encoding MAF bZIP transcription factor B; Supplementary Table 22). MafB expression in macrophages is upregulated by oxidized LDL stimulation^36^, while MafB deficiency in mice appears to increase atherosclerosis by inhibiting foam cell apoptosis^37^.

### Polygenic prioritization of candidate causal genes

Combining locus- and similarity-based approaches has been shown to enhance the prioritization of causal genes^14,38^. However, established similarity-based methods have not leveraged the full polygenic signal to inform gene prioritization. We therefore incorporated a new similarity-based method for gene prioritization, the Polygenic Priority Score (PoPS), which uses the full genome-wide association data^15^. We applied PoPS to summary-level data from the GWAS meta-analysis. Initial 57,543 features—including gene expression, protein–protein interaction networks, and biological pathways—were considered, of which 19,091 features (33.2%) passed a marginal feature selection step and were input into the final PoPS model (Online Methods and Supplementary Table 23). We computed a PoPS score for all protein-coding genes within 500 kb of all 279 genome-wide associations and prioritized the gene with the highest PoPS score in each locus, resulting in 235 prioritized genes. PoPS prioritized many well-established genes implicated in CAD pathogenesis, including *LDLR, APOB, PCSK9, SORT1, NOS3, VEGFA* and *IL6R* (Supplementary Tables 24 and 25).

**Fig. 4:**
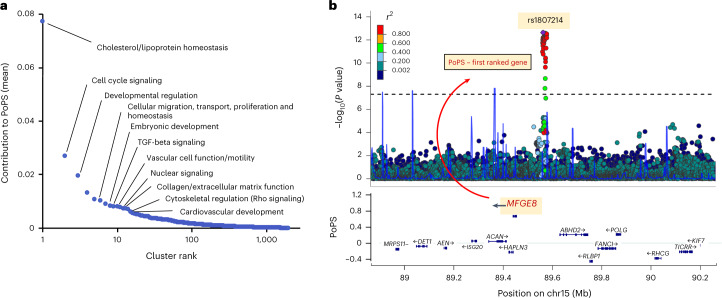
PoPS informs the identification of causal genes for CAD. **a**, Feature clusters contributing to causal gene prioritization. Rank-order plot of 2,852 feature clusters (arising from 19,091 distinct features) contributing to the prioritization of likely causal genes for CAD by PoPS. Similarity-based cluster labels are provided for several top clusters. **b**, Prioritization of *MFGE8* for rs1807214. Regional association plot at chromosome 15 demonstrates the prioritization of *MFGE8* as the likely causal gene for rs1807214, which lies in an intergenic region of chromosome 15. Genes in the region are plotted by their chromosomal position (*x* axis) and PoPS (*y* axis).

Next, we identified features from the PoPS model which were most informative in prioritizing CAD-relevant genes. Hierarchical clustering yielded 2,852 clusters, which we ranked by relative contribution to the PoPS scores of prioritized genes (Fig. 4a). The highest-ranking cluster contained features indicating homeostatic regulation of blood lipids (Supplementary Table 26). Other top clusters were related to vascular cell function, migration and proliferation; the structure and function of the extracellular matrix and metabolic pathways including those in adipose tissue controlling thermoregulation, all well-established mechanisms in CAD pathogenesis^39–41^. Additional high-ranking clusters highlighted early developmental processes and cell cycle signaling pathways as less recognized, but important, mediators of CAD risk.

We then examined a locus where the PoPS method facilitated the prioritization of a putative causal gene. Lead variant rs1807214 lies in an intergenic region of chromosome 15 at which no causal gene has been established^7,8^. Data from GTEx and STARNET identified *cis*-eQTLs for *ABHD2, MFGE8* and *HAPLN3* (Supplementary Tables 27 and 28). Prior locus-based algorithms have prioritized the nearest gene, *ABHD2*, located 65 kb downstream of the sentinel variant^5,38^. However, PoPS prioritized *MFGE8*, located 108 kb upstream of the sentinel (Fig. 4b). *MFGE8* encodes lactadherin, an integrin-binding glycoprotein implicated in vascular smooth muscle cell (VSMC) proliferation and invasion, and the secretion of proinflammatory molecules^42,43^. In vitro deletion of this intergenic region by CRISPR–Cas9 increases *MFGE8* expression—with no change to *ABHD2* expression—and *MFGE8* knock-down reduces coronary artery (CA)-VSMC and monocyte (THP-1) proliferation, lending functional support to *MFGE8* as a likely causal mediator of the CAD association in this region^44^.

### Systematic prioritization of putative causal genes

**Fig. 5:**
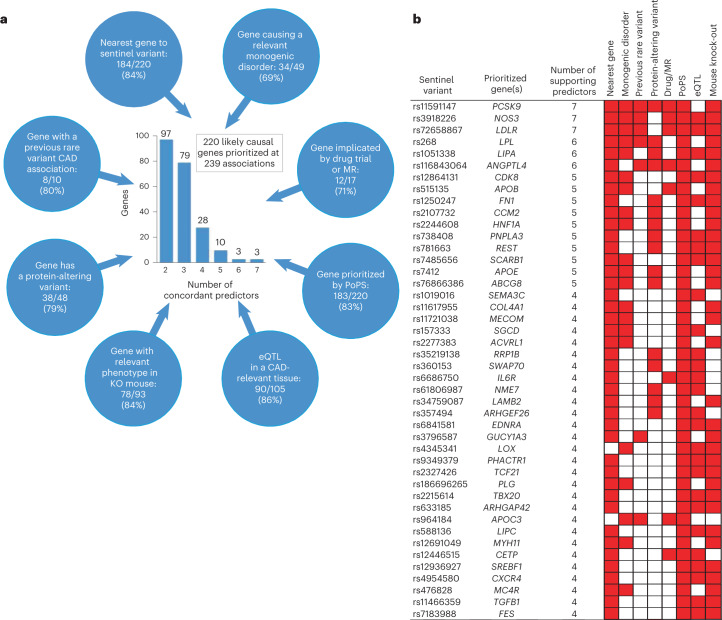
Integrating eight gene prioritization predictors to identify most likely causal genes. **a**, Prioritization of 220 likely causal genes using eight predictors. Blue circles represent the eight predictors used to prioritize causal genes, which are as follows: (1) a gene in the region harbors a variant that ClinVar classifies as having evidence for being pathogenic for a cardiovascular-relevant monogenic disorder (Supplementary Table 34); (2) a gene in the region has been implicated by an effective drug targeting the protein and/or a positive MR study suggesting a causal effect of the protein on CAD (Supplementary Table 31); (3) either of the two top prioritized genes in the region from PoPS (Supplementary Table 24); (4) a gene in the region has an eQTL in a CAD-relevant tissue from GTEx or STARNET for which the lead eSNP is in high linkage disequilibrium (LD) (*r*^*2*^ ≥ 0.8) with the CAD sentinel variant (Supplementary Tables 27 and 28); (5) a gene for which a mouse knock-out has a cardiovascular-relevant phenotype (Supplementary Table 35); (6) a gene in the region harbors a protein-altering variant that is in high LD (*r*^*2*^ ≥ 0.8) with the CAD sentinel variant (Supplementary Table 31); (7) a gene in the region has been shown to have a rare variant association with CAD in a previous WES or genotyping study (Supplementary Table 31); (8) the nearest gene to the CAD sentinel variant. Numbers in the blue circles indicate, firstly, the number of genes for which this predictor agreed with the most likely causal gene, secondly, the number of genes for which this predictor provided evidence for at least one gene, and in parentheses, the percentage agreement (that is, the first number as a percentage of the second). The central histogram shows the number of agreeing predictors that supported the 220 prioritized genes by the number of genes. **b**, Predictors for 44 most likely causal genes strongly prioritized by at least four agreeing predictors. The matrix denotes predictors that supported the most likely causal gene (colored red) for each of the 44 most likely causal genes with at least four predictors that supported the gene. Genes are ordered by number of agreeing predictors. The sentinel variant for the association with the smallest *P* value for CAD is shown for each gene. Full details of the causal gene prioritization evidence for all 279 genome-wide associations are presented in Supplementary Table 31 and the 79 most likely causal genes with three agreeing predictors are displayed in the same format in Supplementary Fig. 1.

We developed and applied a consensus-based prioritization framework involving eight similarity-based or locus-based predictors to systematically prioritize likely causal genes for all 279 genome-wide associations (Online Methods and Fig. 5a). Most likely causal genes were selected based on the highest (unweighted) number of the eight predictors. To test this framework, we generated an *a priori* set of 30 ‘positive control’ genes with well-established causal roles in CAD and assessed the accuracy of each predictor (Supplementary Table 29). Twenty-eight of the 30 positive control genes were correctly prioritized as the most likely causal gene based on the highest number of concordant predictors with a median of four concordant predictors per gene (Supplementary Table 30). All predictors demonstrated high accuracy, including nearest gene (90%), PoPS (90%), eQTL (85%) and mouse knock-outs (100%; Supplementary Table 30).

**Extended Data Fig. 6:**
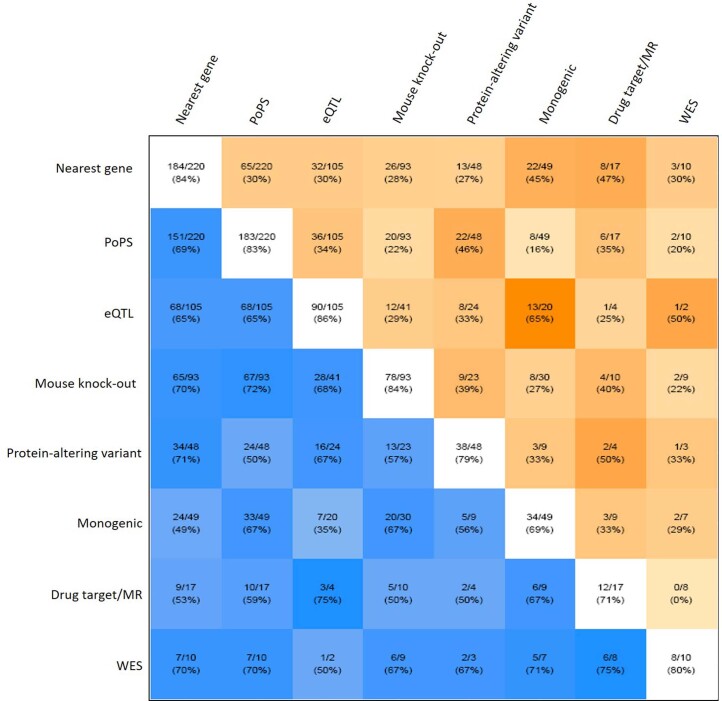
Pairwise concordance of eight gene-prioritization predictors to identify most likely causal genes. White squares lying on the diagonal contain the number of genes for which that predictor provided evidence (denominator) and the number of times for which that predictor prioritized the most likely causal gene at the locus (numerator). For example, eQTL data provided evidence for 105 causal genes, of which 90 (86%) were also the most likely causal gene at the locus. Blue squares below the diagonal show the concordance between pairs of predictors and contain the number of genes for which both predictors provided evidence (denominator) and the number of times for which the prioritized causal gene was the same (numerator). For example, the nearest gene and the presence of a protein-altering variant in high LD (*r*^2^ > 0.8) with the CAD sentinel both provided evidence for a causal gene at 48 loci, of which they were concordant (that is prioritized the same causal gene) at 34 (71%). Darker blue squares show higher levels of concordance. Orange squares above the diagonal show the discordance between pairs of predictors and contain the number of genes for which both predictors provided evidence (denominator) and the number of times for which the prioritized causal gene was the different (numerator). For example, the nearest gene and the presence of a protein-altering variant in high LD (*r*^2^ > 0.8) with the CAD sentinel both provided evidence for a causal gene at 48 loci, of which they were discordant (that is prioritized a different causal gene) at 13 (27%). Darker orange squares show higher levels of discordance. See Fig. 5a for descriptions of the eight predictors used to prioritize causal genes.

We were able to prioritize a likely causal gene at 239 (85.7%) of the genome-wide associations based on having two or more concordant predictors, resulting in the prioritization of 220 genes (Supplementary Table 31). We considered 123 of these genes *strongly* prioritized (three or more concordant predictors; Fig. 5b and Supplementary Fig. 1). For 21 genes, the prioritized gene was not the nearest gene to the sentinel variant, including *APOC3*, *PLTP* and *LOX*. Agreement (the proportion of times that a predictor prioritized the same gene as the most likely causal gene) was high across predictors, including nearest gene (84%), PoPS (83%) and eQTLs (86%; Fig. 5a). Concordance (the proportion of times a pair of predictors both provided evidence for the consensus-based causal gene) was more variable (Extended Data Fig. 6); nearest gene and the presence of a protein-altering variant were typically concordant (71%), whereas monogenic genes and eQTLs were much less concordant (35%).

### Candidate loci with converging lines of evidence

**Extended Data Fig. 7:**
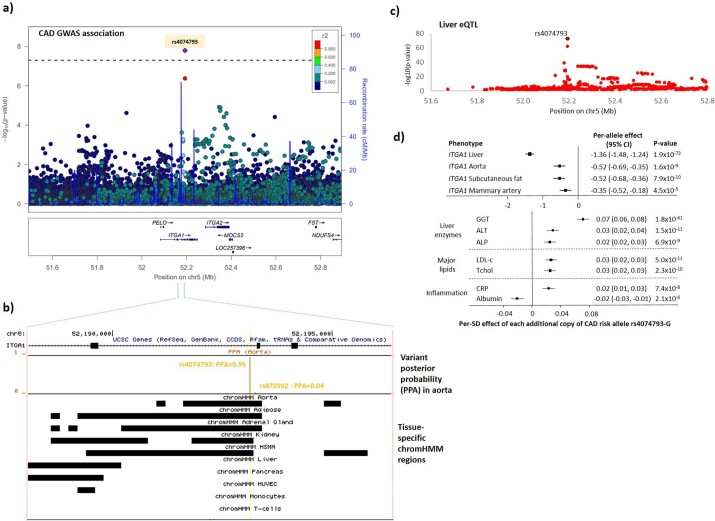
Prioritizing the likely causal variant, gene and pathway at the *ITGA1* locus. **a**, Regional association plot from the primary CAD meta-analysis for the *ITGA1* region. Colored dots represent the position (*x*-axis) in GRCh37 coordinates and –log_10_(meta-analysis *P* value) (*y*-axis) of each variant in the region. Dots are shaded to represent the *r*^2^ with the lead CAD variant (rs4074793), estimated using a random sample of 5,000 European-ancestry participants from UK Biobank. Recombination peaks are plotted in blue based on estimates of recombination from 1000 Genomes European-ancestry individuals. **b**, Tissue-specific imputed chromHMM states at the two credible set variants in the *ITGA1* region. The top track shows the position on chromosome 5 (GRCh37) with respect to the *ITGA1* gene. The second track shows as a vertical orange line the posterior probability (*y*-axis) for each variant in the region from the FGWAS fine-mapping, identifying rs4074793 (PPA = 0.95) as the likely causal variant. The third track indicates as a black box the position of an enhancer state in each of the ten CAD-relevant tissues, using custom imputed chromHMM states based on epigenomic data from the NIH Roadmap Epigenomics Consortium project. The yellow vertical line indicates the position of the likely causal variant (rs4074793) with respect to the chromHMM states. rs4074793 is annotated to a chromHMM state for all five tissues that show enrichment in the region. HSMM, human skeletal muscle cells; HUVEC, human umbilical vein endothelial cells; PPA, posterior probability of being the causal variant. **c**, Effect of rs4074973 on *ITGA1* expression in liver in the STARNET study. The plot shows the position (*x*-axis) in GRCh37 coordinates and –log_10_(*P* value) (*y*-axis) of each variant in the region. The likely causal CAD variant rs4074973 is circled in black. Only variants with *P* < 0.01 are displayed. **d**, Associations of rs4074973 with *ITGA1* expression and phenotypes from a phenome-wide association study. The per-allele association of rs40747973-G (the CAD risk allele) measured in s.d. units is plotted for each phenotype. The box indicates the point estimate and the horizontal bars represent the 95% confidence intervals. The top panel shows the association estimates for *ITGA1* expression from the STARNET study. The bottom panel shows associations from UK Biobank (liver enzymes and inflammatory markers) and the literature (lipids^46^). ALP, alkaline phosphatase; ALT, alanine aminotransferase; CRP, C-reactive protein; GGT, gamma glutamyltransferase; LDL-c, low-density lipoprotein cholesterol; Tchol, total cholesterol.

Several newly identified CAD risk loci had strong variant- and gene-level evidence supporting their candidacy for functional interrogation. For example, we identified a CAD-associated region that was most strongly enriched in the aorta (Supplementary Table 3), with an intronic variant (rs4074793) in *ITGA1* having a PPA of 0.95 (Extended Data Fig. 7a,b). Lead variant rs4074793 lies in a region annotated as a likely enhancer in several tissues and is the lead variant for a strong *cis*-eQTL for *ITGA1* in liver among CAD patients from STARNET (*P* = 1.8 × 10^−73^; Extended Data Fig. 7c). This eQTL was also seen in aorta, subcutaneous fat and mammary artery (Extended Data Fig. 7d). No other gene expression signals were seen at this locus, while PoPS also strongly prioritized *ITGA1* as the likely causal gene (Supplementary Table 31). *ITGA1* encodes integrin subunit alpha-1, a widely expressed protein that forms a heterodimer with integrin beta-1 and acts as a cell surface receptor for extracellular matrix components, such as collagens and laminins. The CAD risk allele (rs4074793-G), or strong proxies, were associated with elevated liver enzymes^45^, C-reactive protein and LDL cholesterol^46^, highlighting the influence of altered *ITGA1* expression in the liver on lipid pathways as a likely causal pathway to CAD.

**Fig. 6:**
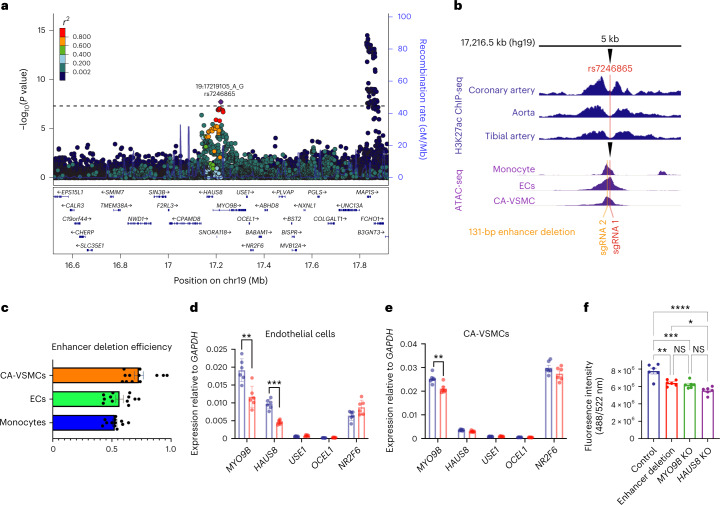
Experimental interrogation of a new CAD locus near *MYO9B*. **a**, Regional association plot from the primary CAD meta-analysis for the new gene-dense region around *MYO9B*. Colored dots represent the position (*x* axis) in GRCh37 coordinates and –log_10_(meta-analysis *P* value) (*y* axis) of each variant in the region. Dots are shaded to represent the *r*^2^ with the lead CAD variant (rs7246865), estimated using a random sample of 5,000 European ancestry participants from the UK Biobank. Recombination peaks are plotted in blue based on estimates of recombination from 1000 Genomes European ancestry individuals. **b**, Identification of a noncoding enhancer in the region around the CAD association signal. The plot shows an inset of a 5-kb window surrounding the lead CAD variant (rs7246865). The top three tracks (blue) show H3K27Ac ChIP-seq of human CA, aorta and tibial artery, identifying a vascular tissue enhancer element overlying rs7246865. The bottom three tracks (purple) show ATAC-seq of human monocytes, immortalized human aortic ECs and CA-VSMCs, identifying a region of open chromatin in all three cell types around rs7246865. The plot also shows the location of the sgRNAs used for deletion of the noncoding enhancer. **c**, Efficiency of CRISPR editing in primary human cells. The Cas9-sgRNA ribonucleoprotein nucleofection method resulted in noncoding enhancer deletion efficiency (*x*-axis) of greater than 0.5 by densitometry and was comparable across monocytes, ECs and CA-VSMCs. Points indicate enhancer deletion efficiency for each of the 12 replicates. Horizontal bars indicate mean enhancer deletion efficiency, and whiskers indicate 95% CIs. **d**, Relative expression of nearby genes after enhancer deletion in ECs. The *y* axis shows mean expression of five local genes expressed in ECs compared to expression levels of a control gene (*GAPDH*). Blue bars indicate gene expression with Cas9–control sgRNA. Red bars indicate expression with tandem enhancer-deleting guides as identified in **b**. Points indicate gene expression levels for each of the six replicates. Vertical bars indicate mean expression levels and whiskers indicate 95% CIs. Gene expression was quantified by qPCR. Expression levels were compared using an unpaired two-way Student’s *t* test. Reduced expression of *MYO9B* and *HAUS8* was identified after 131-bp enhancer deletion as in **b**. **P = 0.0020; ***P < 0.0001. **e**, Relative expression of nearby genes after enhancer deletion in CA-VSMCs. The *y*-axis shows mean expression of five local genes expressed in CA-VSMCs compared to expression levels of a control gene (*GAPDH*). Blue bars indicate gene expression with Cas9–control sgRNA. Red bars indicate expression with tandem enhancer-deleting guides as identified in **b**. Points indicate gene expression levels for each of the six biological replicates. Vertical bars indicate mean expression levels and whiskers indicate 95% CIs. Gene expression was quantified by qPCR. Expression levels were compared using an unpaired two-way Student’s *t* test. Reduced expression of *MYO9B* was identified after 131-bp enhancer deletion as in **b**. **P = 0.0044. **f**, In vitro endothelial wound healing with enhancer and gene deletions. The *y*-axis indicates fluorescence intensity, a read-out for endothelial wound healing and a composite of migration and proliferation. ECs with CRISPR–Cas9 genome editing for enhancer deletion (red) or single-gene knock-outs exhibited diminished wound healing relative to nontargeting control with no deletions (blue). Dots indicate endothelial wound healing for each of the six replicates. Vertical bars indicate mean wound-healing levels and whiskers indicate 95% CIs. Levels of wound healing were compared by one-way ANOVA. *P = 0.0464; **P = 0.0013; ***P = 0.0003; ****P < 0.0001; NS, not significant.

We also identified a new association with CAD at a gene-dense region enriched for epigenetic annotations in adipose, liver, monocytes and skeletal muscle myoblasts (Fig. 6a and Supplementary Table 20). FGWAS prioritized rs7246865 as the putative causal variant (PPA = 0.71). Among 30 genes within 500 kb of rs7246865, PoPS prioritized *MYO9B* (Supplementary Table 24), which encodes unconventional myosin-IXb, a myosin protein with Rho-GTPase signaling activity involved in cell migration^47^. Evidence for the involvement of *MYO9B* was also provided by a *cis*-eQTL in tibial artery in GTEx (*P* = 5.3 × 10^−8^), with the CAD risk allele exhibiting lower *MYO9B* expression (Supplementary Table 27).

### Experimental interrogation of a new CAD locus

We proceeded to investigate the functional significance of the *MYO9B* locus with respect to CAD risk. This genomic region is contained within a vascular tissue enhancer, as identified by a strong H3K27ac ChIP-seq signal in coronary artery, aorta and tibial artery (Fig. 6b). Using ATAC-seq of primary vascular cells, we identified open chromatin at rs7246865 in the following three cell types of relevance to CAD: immortalized human aortic endothelial cells (ECs), CA-VSMCs and monocytes (Fig. 6b).

We used CRISPR–Cas9 to delete the enhancer sequence in these cell types (Fig. 6b), achieving 53–72% effective deletion of a 131-bp segment within the enhancer (Fig. 6c). We measured the transcriptional effect of enhancer deletion on all genes expressed in these cell types within a 250-kb window surrounding rs7246865. The enhancer deletion resulted in reduced *MYO9B* and *HAUS8* expression in ECs (Fig. 6d) and reduced *MYO9B* expression in CA-VSMCs (Fig. 6e), compatible with vascular GTEx eQTLs. There was no change in the expression of any other genes in the region in either cell type or of any gene in monocytes.

Finally, we sought to understand whether the enhancer is associated with a cellular phenotype of relevance to CAD. Given the cytoskeletal functions of *MYO9B* and *HAUS8* in other cell types^47,48^, we assessed the effects of these genes in a monolayer wound-healing assay, a composite of cell migration and proliferation^49^. We observed that ECs with the enhancer deletion exhibited impaired wound healing, as did ECs with knock-outs of either *MYO9B* or *HAUS8*, suggesting that the regulatory effect of the enhancer contributes to CAD risk through impaired wound healing in ECs (Fig. 6f). We did not observe any effect on migration with deletion of the noncoding enhancer or *MYO9B* in CA-VSMCs.

## Discussion

In a discovery analysis involving >200,000 cases of CAD and >1 million controls, we identified 279 genome-wide significant associations, including 82 reported here for the first time. We objectively prioritized likely causal variants and effector genes across all associations using functionally informed fine-mapping, a recently developed genome-wide gene prioritization method (PoPS), and systematic integration of locus-based and similarity-based predictors, with several tailored specifically to cardiovascular disease. Finally, informed by our prioritization framework, we experimentally interrogated a new CAD signal to establish a putative, mechanistic link between this genomic region and risk of CAD.

The large sample size enabled detection of more than 80 new genetic associations with CAD, predominantly common weak-effect variants. Our findings suggest that future, larger GWAS—at least those in European ancestry populations—are unlikely to discover many more large-effect common variants (that is, those with ORs greater than 1.05) associated with CAD. In fact, additional associations contributing to the long polygenic tail of CAD risk are likely to arise from the ~650 predominantly weak-effect signals among associations that reached the 1% FDR threshold, which in aggregate explained ~36% of the heritability of CAD. Notably, we identified 38 new loci when we incorporated recently published GWAS results based on only 29,000 CAD cases from Biobank Japan, demonstrating that future multi-ancestry analyses should enhance the yield of genetic discovery for CAD.

Consistent with previous studies, we demonstrated that a genome-wide PRS derived from this GWAS strongly predicts both incident and recurrent CAD^50–53^. Notably, our new PRS demonstrated improved ability to discern those at higher and lower risk of CAD as compared to a widely used PRS derived from an earlier GWAS of ~61,000 CAD cases^52^. While the new PRS provides an improved tool for genetic risk prediction of CAD in the setting of primary and secondary prevention, our findings suggest that further increases in European-ancestry GWAS sample size may only modestly improve the predictive ability of the CAD PRS. More substantive improvements in polygenic risk prediction may arise from methodological developments, such as approaches that model interactions between variants or incorporate functional information^54,55^. Moreover, further investigations are required to understand the extent to which genetic discovery analyses that include more non-European ancestry participants will improve the portability of PRS across ancestries, and whether this will result in improved prediction across all ancestry groups^56^.

The weak effects of most CAD-associated variants do not preclude their contribution to important etiological insights with therapeutic implications, as the effects of pharmacologically perturbing identified targets are typically much stronger than those of naturally occurring genetic variants that are common in the population. For example, we uncovered common variant associations of weak effect at *HMGCR* and *NPC1L1*, which encode the targets of HMG-CoA reductase inhibitors (statins) and ezetimibe, respectively, two of the most effective and commonly prescribed medications for the prevention and management of CAD through lowering blood lipid levels. However, the translation of statistical associations into actionable biology and potential therapeutic targets requires elucidation of causal genes and mechanisms, which has lagged behind the rapid growth in genetic association discoveries.

Here we implemented strategies to enhance the identification of putative causal variants, genes and biological pathways. By incorporating epigenomic enrichment in disease-relevant tissues—a previously shown approach to improve fine-mapping over broader, disease-agnostic approaches^29^—we prioritized likely causal variants that were not always those with the strongest statistical associations. Using a recently developed similarity-based tool (PoPS) that exploits the full genome-wide data to identify disease-enriched features, we prioritized >200 likely causal genes. Support for the validity of the genes prioritized by PoPS comes from the high ranking of features of known relevance to atherosclerosis (for example, lipid metabolism, extracellular matrix processes) from more than 50,000 tested features; the correct assignment of the most likely causal gene at several well-established lipid and nonlipid CAD loci; selection of the likely-correct causal gene over several other candidates in a region, including those in closer proximity to the sentinel (for example, *MFGE8*); and corroborating evidence at many loci from orthogonal gene prioritization methods, such as eQTLs in disease-relevant tissues.

As support from multiple, orthogonal lines of evidence increases the likelihood of prioritizing the correct causal gene, we propose an integrative, consensus-based prioritization framework that incorporates eight complementary predictors. By applying this framework to all 279 genome-wide associations, we systematically enhance the level of evidence around both known and new risk loci for CAD to arrive at 123 genes strongly prioritized on the basis of having three or more concordant predictors. Although distance from the sentinel variant has been shown to be a reasonable predictor of causal genes across many phenotypes^14,38^, our integrative approach prioritized a gene that was not the nearest gene for 15% of associations. Also, at several newly identified associations, such as those nearest *ITGA1* and *MYO9B*, we provide complementary lines of in silico evidence to nominate potential causal variants, genes and mechanistic pathways. Finally, we leveraged genome-editing and cell-based assays to interrogate the new association signal at chromosome 19, validating the involvement of *MYO9B*, but also implicating another putative causal gene, *HAUS8*. Importantly, these experimental findings substantiate our in silico prioritization of a region with apparent regulatory influence, and our similarity-based prioritization of cell migration pathways, as both *MYO9B* and *HAUS8* may exert their influence on CAD risk through the control of vascular cell cytoskeleton. Furthermore, the findings raise the possibility that two genes at a locus may regulate a common, cellular pathway in coordinated fashion, such as seen for *COL4A1* and *COL4A2* at a well-established CAD risk locus^57^. While experimental evidence is ultimately required to confirm causal mechanisms at all unresolved CAD risk loci, we provide a prioritization framework yielding evidence-based candidates that may be amenable to analogous functional follow-up.

## Methods

### Genetic discovery meta-analysis

Details of the ten de novo studies, including the source of participants, case and control definitions, basic participant characteristics, and ethics approval, are provided in Supplementary Note, Supplementary Table 1 and Extended Data Fig. 1. Study-specific sample and variant filters were applied before additive logistic (or logistic mixed) models were run, with CAD status as the outcome and adjusting for study-specific covariates, including those accounting for potential ancestry effects.

We performed an inverse-variance weighted meta-analysis on the betas and standard errors using METAL^58^, combining the results from the ten de novo studies with previously published summary statistics. Variant-specific sample sizes were maximized by using a combination of summary statistics from prior CAD meta-analyses of the CARDIoGRAMplusC4D consortium, and additional variant filtering was performed, as detailed in Supplementary Note^2,7,10,16^. The final dataset included 20,073,070 variants.

### Joint association analysis

We performed joint association analysis using GCTA software^59^. This approach fits an approximate multiple regression model using summary-level meta-analysis statistics and LD corrections estimated from a reference panel (here the UKBB sample using European ancestry participants only). We adopted a chromosome-wide stepwise selection procedure to select variants and estimate their joint effects at (i) a genome-wide significance level (*P*_Joint_ ≤ 5.0 × 10^−8^) in the meta-analyzed variants that reached genome-wide significance (*n* = 18,348) and (ii) an FDR 1% *P* value cut-off (*P*_Joint_ ≤ 2.52 × 10^−5^) in the 1% FDR variant list (*n* = 47,622). We identified 241 independent variants at the genome-wide significance threshold and 897 independent variants within the 1% FDR list.

### Identifying previously reported regions and associations

To identify regions of the genome previously reported as having associations with CAD, we first collapsed variants reaching genome-wide significance by clumping variants within 500 kb of each other into a single locus. We compared these regions with all variants previously found to be associated with CAD at a genome-wide level of significance (*P* ≤ 5.0 × 10^−8^) from previous large-scale genetic association studies of CAD. Regions were annotated as ‘known’ if they included a previously reported CAD-associated variant. To assess which of our associations were previously reported or new, we examined the pairwise correlation between each of our 279 genome-wide significant sentinel variants and any nearby previously reported variants, defining ‘new’ as having *r*^2^ < 0.2 in UK Biobank European ancestry participants.

### Genetic correlation analysis

Genetic correlation between CAD and conventional risk factors (total cholesterol, LDL cholesterol, HDL cholesterol, triglycerides, body mass index, systolic blood pressure and diastolic blood pressure) and cardiometabolic diseases (type 2 diabetes, ischemic stroke and heart failure) was assessed using LD Score Regression (LDSC)^60^. We used the 1000 Genomes European ancestry LD file comprising ~1.2 million variants available at https://alkesgroup.broadinstitute.org/LDSCORE/.

### PheWAS in UK Biobank

To understand the spectrum of phenotypic consequences of our 279 independent associations with CAD, we conducted a PheWAS in the UK Biobank (see Supplementary Note for complete analysis details). Briefly, we tested for associations with 53 cardiovascular and noncardiovascular diseases and 32 continuous traits, as listed in Supplementary Tables 32 and 33. A genetic variant was considered to be associated with a ‘conventional CAD risk factor’ if the CAD risk-increasing allele exhibited a directionally consistent/positive association with blood lipids (total cholesterol, LDL cholesterol, triglycerides or a diagnosis of hypercholesterolemia); blood pressure (systolic blood pressure, diastolic blood pressure or a diagnosis of hypertension); hyperglycemia (serum glucose, hemoglobin A1c or a diagnosis of type 2 diabetes) or adiposity (body mass index).

### Rare variant analyses

Variant annotation was performed using Variant Effect Predictor (VEP) v96.0 with LOFTEE plugin on version three imputed data and variants with an information score ≥0.8 (refs. ^61,62^). Various gene-based groupings were tested (Supplementary Table 6) and allele frequencies from the entire UK Biobank cohort were used for groupings. Variants (*n* = 64,102) were considered to be in a gene if they fell within the gene coordinates as defined by GENCODE v19. Gene-based association tests were performed in SAIGE-GENE v0.35.8.5 using a white British subset of UK Biobank (28,683 CAD cases and 367,783 controls)^63^. Software defaults were used except in step 0 the number of markers for sparse matrix was 2000, and in step 1, the tolerance for preconditioned conjugate gradient to converge was 0.01 and variance ratios were estimated across MAC categories. Two variants were required in each gene for testing. Covariates in the model included the genotyping array, the first five principal components calculated in the white British subset of samples, birth year, and sex. Burden, SKAT, and SKAT-O tests were performed for each gene. As no strong signals were observed except for the *PCSK9* gene, we did not extend our rare variant testing to other studies.

### Sex-specific analysis

We performed a sex-stratified GWAS analysis in UK Biobank following the same phenotype definition and sample exclusions with the main analysis. We used the SAIGE software and adjusted our single-variant association analysis for the first five genetic principal components and the genotyping array, separately for men and women^64^. Based on promising initial results in UK Biobank, we collated sex-stratified GWAS summary statistics, as available, from other participating studies (Supplementary Table 6). Additional details of sex-specific analyses are provided in Supplementary Note.

### FDR estimation

The FDR following the meta-analysis was assessed using the ‘*q* value’ R package. We generated *q* values for all 20.1 million variants. The *P* value cut-off for a *q* value of 1% was 2.52 × 10^−5^ and there were 47,622 variants reaching that threshold. Joint conditional analysis was performed using GCTA (as described earlier) to identify approximately independent association signals.

### Estimation of heritability explained

Heritability calculations were based on a multifactorial liability-threshold model, implemented in the INDI-V calculator (http://cnsgenomics.com/shiny/INDI-V/), under the assumption of a baseline population risk (*K*) of 0.0719 and a twin heritability (*H*_*L*_^2^) of 0.4 (refs. ^65,66^). Single-variant regression estimates from the meta-analysis summary statistics were used to estimate heritability for the sentinel variants at the 241 conditionally independent genome-wide significant associations and the 897 conditionally independent associations reaching the 1% FDR threshold in the primary meta-analysis. To account for correlation between variants, multiple regression estimates from the GCTA joint association analysis were also used to estimate heritability for both sets of variants.

### Cross-ancestry comparison

For cross-ancestry comparison, we used summary statistics from a recent GWAS of 29,319 CAD cases and 183,134 controls from Biobank Japan^3^. In total, 199 of the 241 sentinel variants from our primary meta-analysis were also found in the Biobank Japan study; after aligning effect alleles, we compared the beta estimates and minor allele frequencies using Pearson’s correlation coefficient. To investigate the effect of outliers on the between-ancestry correlation of beta estimates, we re-estimated the correlation coefficient after excluding three strong outliers (at *ATXN2, FER* and *SLC22A1*). We then performed an inverse-variance weighted meta-analysis on the beta estimates and standard errors, incorporating summary results from Biobank Japan and those from all other studies in our primary meta-analysis. After cross-ancestry meta-analysis, we again dropped variants that were only present in one study or had fewer than 30,000 cases in total from all contributing studies, leaving 23,333,163 variants after filtering. We then collapsed variants reaching genome-wide significance (*P* ≤ 5.0 × 10^−8^) by clumping variants within 500 kb into a single locus, resulting in 38 additional loci that did not contain a previously reported CAD variant.

### Derivation and training of PRSs

PRS were derived using the pruning and thresholding method or the LDpred computational algorithm (LDpred v.1.0), with 503 European ancestry individuals derived from the 1000 Genomes Project study serving as the linkage disequilibrium reference panel^67^. To evaluate the added utility of our GWAS for the prognostication of CAD risk, we compared two sets of scores using effect estimates from either the current meta-analysis or from our previous 1000 Genomes-imputed GWAS of CAD involving ~60,000 cases^7^. For each derivation method and summary statistic, we constructed a range of scores of varying sizes drawing from common genetic variants that overlapped between the current meta-analysis, the earlier 1000 Genomes-imputed CAD GWAS and our training/validation datasets from the MDC Study^68^. Additional details on PRS derivation and training are contained in Supplementary Note.

### Incident event prediction analyses

Cox proportional hazard models were used to assess the time-to-event relationship between each PRS and incident CAD events in the MDC study (see Supplementary Note for study details). Baseline models were adjusted for age and sex only, and then subsequently, for established risk factors for CAD (total cholesterol, HDL cholesterol, systolic blood pressure, body mass index, type 2 diabetes, current smoking status and family history of CAD). Harrell C-statistics were estimated using Cox proportional hazard analysis over a 21-year follow-up period to assess the discrimination of the PRS.

### Recurrent event prediction analyses

The two optimal PRS (2022 PRS and 2015 PRS) were calculated in participants of the genetic substudy of the FOURIER trial (see Supplementary Note for trial details) using the genotype dosage for each allele, multiplied by its weight and then summed across all variants. Patients received a raw score standardized per 1 s.d. (continuous), as well as a percentile score relative to the total cohort. All scoring was performed using PLINK v2.0 (www.cog-genomics.org/plink/2.0/)^69^. Model goodness-of-fit was evaluated using the concordance statistic and Akaike’s Information Criterion. R version 3.6.1 was used for statistical analyses.

The clinical outcome of interest was recurrent major coronary events, defined as myocardial infarction, coronary revascularization or death from CAD (*n*_incident_cases_ = 673). Participants in the genetic cohort were followed for a median of 2.3 years. All endpoints were formally adjudicated by a blinded clinical events committee during the trial. A Cox model was used to determine the HR per 1 s.d. higher level of the PRS and for the extreme deciles compared to the middle 80%. Analyses were adjusted for age, sex and ancestry (using principal components 1–5).

### Identifying protein-altering variants

To identify protein-altering variants among our genome-wide significant associations, we took the 279 sentinel variants and their LD proxies at *r*^2^ ≥ 0.8 as estimated in the European ancestry subset of UK Biobank and annotated them using the Ensembl VEP^62^. We selected for each sentinel variant any proxies identified as having a ‘high’ (that is, stop-gain and stop-loss, frameshift indel, donor and acceptor splice-site and initiator codon variants) or ‘moderate’ (that is, missense, in-frame indel, splice region) consequence and recorded the gene that the variant disrupts.

### Functional GWAS analysis

To fine-map loci and identify credible functional variants, we applied FGWAS software^29^. The software integrates GWAS summary statistics with epigenetic data and we used the ChromHMM-derived states from the NIH Roadmap Epigenomics Consortium on a selection of ten CAD-relevant cell/tissue types (adipose nuclei, aorta and human skeletal muscle myoblasts (HSMM), liver, human umbilical vein endothelial cells (HUVEC), kidney, adrenal gland, pancreatic islets, primary monocytes and T-cells from peripheral blood)^70,71^. To maximize our search space to find functional elements, we prepared a custom state by merging likely functional ChromHMM states (enhancers, transcription start sites, repressed polycomb, transcription at 5′ and 3′ of gene) for each genomic position. We reweighted the GWAS by running a null model and then a model containing the custom annotation for each of the ten tissues. Regions of the genome that showed strong enrichment (>3 s.d. increment in Bayes factor (BF)) and had a genome-wide significant CAD-associated variant (*P* < 5.0 × 10^−8^) were selected. For each region, we identified the tissue that showed maximum increment in BF and then constructed a 95% credible functional set of variants based on the ranked PPA for each variant within a region.

### eQTL analysis in CAD-relevant tissues

To examine whether the CAD associations were driven by changes in gene expression in CAD-relevant tissues and cell types, we interrogated *cis*-eQTLs from CAD-relevant tissues in the STARNET eQTL study and the GTEx study^34,35^. Analysis-specific details are provided in the Supplementary Note.

### Polygenic prioritization of candidate causal genes

We implemented PoPS, a similarity-based gene prioritization method designed to leverage the full genome-wide signal to nominate causal genes independent of methods utilizing GWAS data proximal to the gene^15^. Broadly, PoPS leverages polygenic enrichments of gene features including cell-type-specific gene expression, curated biological pathways and protein–protein interaction networks (Supplementary Table 23) to train a linear model to compute a PoPS for each gene (see Supplementary Note for further details).

### Variants responsible for cardiovascular-relevant monogenic disorders

To identify genes harboring pathogenic variants responsible for cardiovascular-relevant monogenic disorders, we searched the NCBI’s ClinVar database (https://www.ncbi.nlm.nih.gov/clinvar/) on 26 June 2020. Variants were pruned to those within ±500 kb of our CAD sentinel variants; categorized as ‘pathogenic’ or ‘likely pathogenic’; with a listed phenotype; and with either (i) details of the evidence for pathogenicity, (ii) expert review of the gene or (iii) a gene that appears in practice guidelines. We then filtered variants that were annotated with a manually curated set of cardiovascular-relevant phenotype terms, including those related to CAD, CAD risk factors (lipids, metabolism, blood pressure, obesity and platelets), bleeding disorders and relevant cardiac, vasculature or neurological abnormalities (Supplementary Table 34). Where a variant was annotated with multiple genes, both genes were considered as potentially pathogenic.

### Phenotyping knock-out mice

Human gene symbols were mapped to gene identifiers (HGNC) and mouse ortholog genes were obtained using Ensembl (www.ensembl.org). Phenotype data for single-gene knock-out models were obtained from the International Mouse Phenotyping Consortium, data release 10.1 (www.mousephenotype.org), and from the Mouse Genome Informatics database, data from July 2019 (www.informatics.jax.org). For each mouse model, reported phenotypes were grouped using the mammalian phenotype ontology hierarchy into broad categories relevant to CAD as follows: cardiovascular physiology (MP:0001544), cardiovascular morphology (MP:0002127), growth and body weight (MP:0001259), lipid homeostasis (MP:0002118), cholesterol homeostasis (MP:0005278) and lung morphology (MP:0001175). This resulted in mapping from genes to phenotypes in animals (Supplementary Table 35).

### Rare variant associations, MR and drug evidence

To inform prioritization of causal genes within 1-Mb regions around our genome-wide associations, we reviewed the literature for three sources of evidence as follows: (1) rare coding variants previously associated with CAD, either individually or in aggregate gene-based tests, through whole-exome sequencing (WES) or exome array studies; (2) Mendelian randomization (MR) studies of gene expression, protein levels or proximal phenotypes that implicate specific genes as causal effector genes for CAD and (3) drugs proven to be effective for cardiovascular-relevant indications and that target specific proteins encoded by genes.

### Systematic integration of gene prioritization evidence

To systematically prioritize likely causal genes for all 279 genome-wide associations, we integrated the following eight of the aforementioned similarity-based or locus-based predictors of causal genes: (1) the top two prioritized genes from PoPS; (2) genes with eQTLs in CAD-relevant tissues from STARNET or GTEx; (3) genes containing protein-altering variants that are in strong LD (*r*^2^ ≥ 0.8) with the CAD sentinel variant; (4) genes harboring variants responsible for monogenic disorders of cardiovascular relevance according to ClinVar; (5) genes containing rare coding variants that have been associated with CAD risk in previous WES or array-based studies; (6) genes encoding proteins of causal relevance to CAD per MR studies or that are targets for established cardiovascular drugs; (7) genes that display cardiovascular-relevant phenotypes in knock-out mice from the International Mouse Phenotyping Consortium or Mouse Genome Informatics database; and (8) the nearest gene to the CAD sentinel variant (Fig. 5a). We prioritized the most likely ‘causal gene’ for each association using a consensus-based approach, selecting the gene with the highest, unweighted sum of evidence across all eight predictors.

We tested our approach by evaluating whether 30 (positive control) genes with established relevance to CAD were prioritized as the most likely causal genes within their respective genomic regions. Positive control genes were selected by a literature search that sought evidence from engineered mouse models of reduced gene expression (‘knock-out’ or ‘knock-down’ models), MR studies or successful drug targets. In addition, we defined two measures to summarize the relative contributions of individual predictors and pairs of predictors to the consensus-based approach. Specifically, we defined ‘agreement’ as the proportion of times that an individual predictor prioritized the same gene that was nominated as the most likely causal gene by the consensus-based framework. ‘Concordance’ was defined as the proportion of times a pair of predictors both converged on the gene that was nominated as the most likely causal gene by the consensus of the eight predictors.

### CRISPR–Cas9 genome editing in vascular cells

Human coronary artery VSMCs (Lonza CC-2583; culture media CC-31182) were used at passage five or earlier. Endothelial cell experiments were conducted with immortalized human aortic endothelial cells (ATCC CRL-4052; culture media Lifeline Cell Technology LL-0003). Monocyte experiments were conducted with THP-1 monocyte cells (ATCC TIB-202; culture media RPMI ATCC 30-2001, 10% FBS Sigma 12306C-500ML). Genome editing was performed as previously described (Supplementary Note)^72^.

### Gene expression by qPCR

For assessment of gene expression, mRNA was extracted (Qiagen RNAeasy kit; Qiagen, 74106) and DNase I digestion was performed (DNAse I, Thermo Fisher 18068015) before cDNA synthesis (Applied Biosystems, 43-688-14) and qPCR (Applied Biosystems, 4444965). Gene expression was assessed by quantitative PCR with Taqman probes (Invitrogen) for genes of interest (*MYO9B*: Hs00994622_m1; *HAUS8*: Hs00928622_m1*; OCEL1*: Hs00928613_m1; *USE1*: Hs00218426_m1; *NR2F6*: Hs00172870_m1; *GAPDH*: Hs03929097_g1). Data are shown relative to expression of *GAPDH*. Statistical analyses were conducted with unpaired two-way Student’s *t* test.

### Noncoding enhancer characterization

Assay for transposase-accessible chromatin using sequencing (ATAC-seq) data for THP-1 monocytes and CA-VSMCs was previously available. We performed ATAC-seq in human immortalized aortic endothelial cells as previously described^73^. H3K27ac CHIP-seq data were publicly available via ENCODE (coronary artery, ENCFF970RKM; aorta, ENCFF118EKX; tibial artery, ENCFF972ZHA).

### Wound-healing assay

Wound-healing assays were performed as previously described (Platypus Technologies, CMAUFL4)^49^. After genome editing, 15,000 cells per well were plated with well inserts in place in culture media. Inserts were then removed the day after plating. Prior to complete wound healing (48–72 h), cells were stained with Calcein AM dye (Invitrogen, C3099) and wound healing was quantified with a fluorescence plate reader (excitation 488 nm/emission 522 nm). Statistical analyses were conducted with one-way ANOVA between groups. Where specific software tools are not named, we used Stata or R for analyses.

### Reporting summary

Further information on research design is available in the Nature Portfolio Reporting Summary linked to this article.

## Online content

Any methods, additional references, Nature Portfolio reporting summaries, source data, extended data, supplementary information, acknowledgements, peer review information; details of author contributions and competing interests; and statements of data and code availability are available at 10.1038/s41588-022-01233-6.

## Supplementary information

Supplementary Information Supplementary Fig. 1 and Supplementary Note.

Reporting Summary

Supplementary Data 1 Regional association plots for the 241 genome-wide significant signals from the primary CAD GWAS meta-analysis.

Supplementary Tables Supplementary Tables 1–35.

## Data Availability

Summary statistics are available upon publication through the CARDIoGRAMplusC4D website (http://www.cardiogramplusc4d.org/) and the NHGRI-EBI GWAS catalog (https://www.ebi.ac.uk/gwas/, accession codes: GCST90132314 (https://www.ebi.ac.uk/gwas/studies/GCST90132314) and GCST90132315 (https://www.ebi.ac.uk/gwas/studies/GCST90132315)). Interactive searchable Manhattan plots and a locus-specific epigenome annotation browser for functionally enriched loci are available at https://procardis.shinyapps.io/cadgen/. An interactive searchable browser detailing the locus-specific evidence prioritizing causal variants, genes and pathways is available at the Common Metabolic Diseases Knowledge Portal (https://hugeamp.org/method.html?trait=cad&dataset=cardiogram). Other datasets used in this study include the NCBI’s ClinVar database (https://www.ncbi.nlm.nih.gov/clinvar/) on 26 June 2020, a 1000 Genomes European ancestry LD file comprising ~1.2 million variants (https://alkesgroup.broadinstitute.org/LDSCORE/), the GTEx Consortium v7 data release (https://www.gtexportal.org/home/datasets), the Ensembl database (www.ensembl.org), the International Mouse Phenotyping Consortium, data release 10.1 (www.mousephenotype.org) and the Mouse Genome Informatics database, data from www.informatics.jax.org on July 2019.

## Code availability

Custom code for preparing the study-specific GWAS summary statistics files for meta-analysis can be found at https://github.com/cambridge-ceu/cardiogramplusC4D_GWAS. Custom code for PRS analysis using a modified version of Ldpred 1.0 can be found at https://github.com/wavefancy/LDpredChrByChr.
