# Supplementary material for: Discovery and systematic characterization of risk variants and genes for coronary artery disease in over a million participants
Source: Nat Genet. 2022 Dec 6;54(12):1803–15. doi: 10.1038/s41588-022-01233-6 (PMC9729111; doi:10.1038/s41588-022-01233-6)
Supplement: Supplementary file 1 — Supplementary Fig. 1 and Supplementary Note. [file 41588_2022_1233_MOESM1_ESM.pdf]

nature genetics

Article

https://doi.org/10.1038/s41588-022-01233-6

Discovery and systematic characterization of risk variants and genes for coronary artery disease in over a million participants

In the format provided by the authors and unedited

SUPPLEMENTARY NOTE

Genetic discovery meta-analysis

Details of the ten *de novo* studies are presented in Supplementary Table 1 and Extended Data Figure 1. Ethical approval and informed consent were obtained for all participating studies. With the exception of UK Biobank (which used the ThermoFisher UK Biobank Axiom array), studies used Illumina genotyping arrays. Most studies used the Haplotype Reference Consortium v1.1 panel for imputation, but several utilized local whole-genome sequence data for improved imputation.

An inverse variance weighted meta-analysis was performed using METAL,[1] combining the results from the ten *de novo* studies with previously published summary statistics. To maximize the variant-specific sample size, we used summary statistics from either (a) a previous 1000 Genomes-imputed GWAS meta-analysis of up to 60,801 CAD cases and 123,504 CAD-free controls;[2] (b) a meta-analysis of ~79,000 variants in up to 88,192 CAD cases and 162,544 controls, predominantly based on the Illumina CardioMetabochip array;[3] or (c) a meta-analysis ~184,000 variants in up to 42,335 CAD cases and 78,240 controls based on the Illumina Exome array.[4, 5] From each meta-analysis, we dropped variants which were only present in one study or had fewer than 30,000 cases in total from all contributing studies. Where a variant was found in multiple meta-analyses, we kept the result which had the highest total number of 'effective cases' across studies (approximated within each study as the variant-specific number of CAD cases multiplied by the imputation quality score). Finally, to avoid false positive associations driven by an extreme result in a single study, we filtered variants with a meta-analysis p-value$\leq 5.0\times10^{-6}$ that did not have a p-value<0.2 in at least two studies for which the direction of effect was consistent with the overall meta-analyses effect estimate. Our final dataset included 20,073,070 variants after filtering.

Phenome-wide association study (PheWAS) in UK Biobank

We conducted a phenome-wide association study in UK Biobank of our 279 independent associations with CAD against 53 cardiovascular and non-cardiovascular diseases and 32 continuous traits. A complete list of the phenotypes assessed, details on disease definitions, and relevant sample sizes are provided in Supplementary Tables 32 and 33. We limited analyses to UK Biobank participants of European genetic ancestry as defined by principal components analysis, and excluded one individual in each pair with *KING* coefficient > 0.0884, indicating 2nd degree or closer relatedness (n=393,461). For disease phenotypes, we performed logistic regression adjusted for age, sex, genotyping array, and the first five principal components. An association with a disease phenotype was deemed significant at a Bonferroni-corrected threshold of p-value$<3.4\times10^{-6}$ (53 diseases x 279 genetic variants). Continuous phenotypes were residualized after adjusting for age, sex, genotyping array, and the first five principal components; linear regression was performed on residuals following inverse-normal transformation. For analysis of glycemic traits (hemoglobin A1c and serum glucose), participants with type 1 or type 2 diabetes were excluded. An association with a continuous phenotype was deemed significant

at a Bonferroni-corrected threshold of P-value<$5.6x10^{-6}$ (32 continuous traits x 279 genetic variants).

Sex-specific analysis

Sex-stratified GWAS analyses were performed in UK Biobank using SAIGE and adjusting for the first five genetic principal components and the genotyping array, separately for men and women. Analyses were then extended to other participating studies (Supplementary Table 6). All sex-specific summary statistics were checked for quality control (QC) cohort-wise to exclude poorly imputed variants (info<0.4), improbable betas (>|4|) and significant deviations from Hardy-Weinberg Equilibrium (p-value<$1.0x10^{-9}$). Cohort-wise sex-specific q-q plots were generated and inspected and the genomic inflation statistic (λ) was also calculated. Association summary statistics from all 17 studies were combined via inverse-variance weighted meta-analysis in GWAMA.[6, 7] We implemented three different types of meta-analysis: a) a sex-specific meta-analysis, where summary statistics were combined separately for men and women; b) a sex-combined meta-analysis, where effect estimates from men and women were combined assuming no between-sex heterogeneity; and c) a sex-differentiated meta-analysis, where sex-specific estimates were combined while allowing for heterogeneity between men and women. We excluded genetic variants that had a minor allele count < 10 or minor allele frequency < 0.01, were only present in one study, or had a sample size below the median sample size in the sex-combined meta-analysis. To identify significant sex-differentiated genetic variants, we considered variants that had a p-value≤$5.0x10^{-8}$ from the sex-differentiated meta-analysis and a sex-heterogeneity p-value≤0.01. Among the significantly associated genetic variants we then applied a 500kb pruning to identify the sex-differentiated CAD loci.

Derivation and training of polygenic risk scores

Polygenic risk scores (PRS) were derived using one of two methods – pruning and thresholding or the LDpred computational algorithm. European ancestry individuals (N=503) from the 1000 Genomes project were employed for the linkage disequilibrium panel. Two sets of PRS were created using either results from the current meta-analysis or from our previous 1000 Genomes-imputed GWAS of CAD involving ~60,000 cases. A spectrum of scores were constructed of varying sizes for each score derivation method and set of summary statistics. Specifically, pruning and thresholding-based scores were created using a combination of p-value (1, 0.5, 0.05, $5x10^{-3}$, $5x10^{-4}$, $5x10^{-5}$, $5x10^{-6}$, $5x10^{-7}$, $5x10^{-8}$) and r^2 (0.2, 0.4, 0.6, 0.8, 0.99) thresholds, yielding 45 distinct PRS for each of the two sets of GWAS summary statistics utilized (90 total pruning and thresholding-based scores). LDpred-based scores were constructed incorporating all available SNPs, HapMap3 SNPs (gs://gnomad-public/resources/grch37/hapmap/hapmap_3.3.b37.vcf.bgz), or a soft LD-clumping approach using combinations of p-value (0.05, 0.5, 1) and r^2 (0.2, 0.4, 0.6, 0.8, 0.99) thresholds (17 total sets of input variants). Additionally, we employed a tuning parameter (ρ) for LDpred, which represents the fraction of causal variants, and tested all LDpred-based scores across a range of ρ parameters (1, 0.3, 0.1, 0.03, 0.01, 0.003, 0.001 and an infinitesimal model), yielding 136 distinct PRS per set of summary statistics utilized (272 total LDpred-based PRS).

PRS were computed using variants with high-quality imputation results available in the Malmö Diet and Cancer Study (MDC), defined by information score (INFO) > 0.3. For each participant, the raw PRS was generated by multiplying the genotype dosage for each risk-increasing allele by its respective weight and then summing across all variants in the score using PLINK2 software. To permit adjustment for genetic ancestry, principal components of ancestry were computed using the EIGENSOFT software package. The calculated raw PRS was ancestry-adjusted by taking the residual of a linear regression model that predicted PRS using the first ten principal components.

We trained all pruning and thresholding and LDpred PRS (362 total scores) in a subset of the MDC (n=22,872; $n_{incident\_cases}$=3,307). Cox proportional hazard models were used to assess the time-to-event relationship between each PRS and incident CAD, with or without adjustment for age and sex. Bootstrapping analysis was performed (100 iterations) and the mean hazard ratio (HR) and mean area under the receiver operator characteristic curve (AUC; as calculated by Harrell's C-statistic) were reported as performance metrics to rank scores within each of four categories as classified by the PRS derivation method (pruning and thresholding; LDpred) and effect estimates utilized (2015 CAD GWAS; Current meta-analysis). Metrics for the top-performing PRS in each category were compared by Wilcoxon rank-sum test based on results of bootstrapping analyses.

Malmö Diet and Cancer study (MDC)

The Malmö Diet and Cancer Study is a prospective, population-based cohort that enrolled 30,447 participants between 1991 and 1996 ranging in age from 44 to 73 years. Baseline information on lifestyle and clinical factors was collected using a detailed questionnaire as previously described.[8] From the total study population, 28,556 participants (94%) who had genetic data available and were free of CAD at time of enrollment were analyzed. A subset of 5685 randomly selected participants, that comprised the Malmö Diet and Cancer Cardiovascular Cohort, had blood cholesterol concentrations recorded. Incident cases of CAD had either fatal or nonfatal myocardial infarction, coronary artery bypass graft surgery, percutaneous coronary angioplasty or death due to CAD. Incident event adjudication was available through December 31, 2016. Genotyping was performed using the Illumina GSA v1 genotyping array. Of 29,304 samples which underwent genotyping and were free from CAD at baseline, 28 556 (97%) were retained after quality control procedures that removed low-quality samples (discordance between reported and genetically inferred sex, low call rate (<90%), and sample duplicates). With respect to genetic variants, quality control was performed with removal of those not in Hardy-Weinberg equilibrium (p-value$<1\times10^{-15}$). Imputation was then performed using the Haplotype Reference Consortium reference panel.

The FOURIER trial (and genetic subset)

The FOURIER trial was a multinational, randomized, double-blind, placebo-controlled trial of the efficacy of evolocumab in patients with clinically evident atherosclerotic cardiovascular disease.[9] The key inclusion criteria for the trial were age between 40 and 85 years, LDL cholesterol of 70 mg/dl or greater or non-HDL-C of 100 mg/dl or greater, and a history of either myocardial infarction, non-hemorrhagic stroke, or symptomatic peripheral artery disease. The genetic sub-study included all participants in FOURIER who provided consent for genetic analyses at

enrollment into the trial and had genotyped data that passed quality control (QC), and were of European ancestry. The final genetic cohort comprised 14,298 unrelated European-ancestry participants, of whom 7,135 were in the placebo arm of the trial. There were no clinically important differences between the overall trial participants and the participants in the genetic subset.

Expression eQTL analysis in CAD-relevant tissues

We examined *cis*-eQTLs from CAD-relevant tissues in the Stockholm-Tartu Atherosclerosis Reverse Network Engineering Task (STARNET) eQTL study and the Genotype-Tissue Expression (GTEx) study to determine whether our CAD associations were driven by changes in gene expression in CAD-relevant tissues and cell types. For STARNET, we used *cis*-eQTL associations from seven tissues (atherosclerotic aortic root [AOR], atherosclerotic-lesion-free internal mammary artery [MAM], blood [BLD], liver [LIV], subcutaneous fat [SF], skeletal muscle [SKLM], and visceral abdominal fat [VAF]) taken from 600 CAD patients as previously described. We cross-referenced the sentinel CAD variants and their proxies ($r^2 \geq 0.8$) with STARNET eQTLs reaching a 5% FDR for all tissues. To ensure the CAD association and eQTL are likely to be driven by the same causal variant, we retained only those eQTLs where the CAD-associated variant and the lead eQTL variant had $r^2 \geq 0.8$ among European ancestry participants from UK Biobank. For GTEx we followed the same procedure using the v7 data release (https://www.gtexportal.org/home/datasets) and restricted to *cis*-eQTLs reaching a 5% FDR from eight tissues (adipose [subcutaneous, visceral omentum], adrenal gland, artery [aorta, coronary, tibial], liver and whole blood).

Polygenic prioritization of candidate causal genes (PoPS)

PoPS is a similarity-based gene prioritization method that utilizes the full genome-wide signal to nominate causal genes that is in contrast to more locus-based methods that focus on GWAS data proximal to the gene.[10] PoPS leverages polygenic enrichments of gene features including cell-type specific gene expression, curated biological pathways, and protein-protein interaction networks to compute a polygenic priority score (PoPS) for each gene (additional details in Supplementary Methods). Specifically, PoPS was used to train a linear model to predict gene-level association scores from gene features. First, MAGMA was applied to GWAS summary statistics using the 1000 Genomes Project reference panel,[11] which outputs gene-phenotype association p-values derived from the mean chi-square statistic of variants within the gene body. The gene p-values were converted to z-scores $z_g = F^{-1}(1 - p_g)$, where F^{-1} is the probit function. This yielded a roughly normally distributed variable that reflects the strength of the variant-phenotype association localized to variants within each gene body. However, these scores are contaminated by the effects of linkage disequilibrium, and are imperfect metrics to determine causal gene-phenotype relationships. Thus, PoPS incorporates additional biologically-relevant information by fitting a matrix of gene features to the MAGMA scores. In total, 57,543 features were considered for analysis, including data on gene expression, protein-protein interaction networks, and biological pathways (Supplementary Table 23). PoPS performs marginal feature selection (i.e. removes all features with marginal correlation > 0.05) in order to reduce the dimensionality of the regression, then performs a generalized least squares with L2 regularization to learn the coefficients of a linear model. L2 regularization is adjusted such that the leave-one-out cross-validation error is minimal. The predicted values, a kind of reconstituted MAGMA score,

are the PoP scores for each gene, with higher scores reflecting stronger evidence for a causal gene-phenotype relationship.

CRISPR-Cas9 genome editing in vascular cells

Human coronary artery vascular smooth muscle cells (Lonza CC-2583; culture media CC-31182) were used at passage five or earlier. Endothelial cell experiments were conducted with immortalized human aortic endothelial cells (ATCC CRL-4052; culture media Lifeline Cell Technology LL-0003). Monocyte experiments were conducted with THP-1 monocyte cells (ATCC TIB-202; culture media RPMI ATCC 30-2001, 10% FBS Sigma 12306C-500ML). Genome editing was performed as described previously [12]. Briefly, control guide (5'- gcactaccagagctaactca-3') or targeting guides are complexed with Cas9 for 15-20 minutes while preparing a cell suspension. 150-200k cells were suspended in supplemented SG solution and electroporated in nucleofector (V4XC-3032) with 50pMol sgRNA per sgRNA and 20pMol Cas9 per reaction (Synthego). Nucleofector programs were CA-210 for ECs, CM-137 for CA-SMCs and FF-100 for monocytes. Cells from each reaction were immediately split for DNA and RNA specimens. Cells were simultaneously harvested in a subconfluent state for DNA or RNA after 24-48 hours to check genome editing efficiency and gene expression. To generate 131bp enhancer deletion at rs7246865, paired guides were used (upstream guide 5'- accgggggucugacucacag-3'; downstream guide 5'- cuugagccaaauccacguca-3') and a 769bp amplicon was generated to access enhancer deletion (Forward primer 5'-ACACAGTTTCTGGGGGTGAG-3'; Reverse Primer 5'-AAAAGCTCAGTGGCTGCAAT-3'). Efficiency of single guide indels was determined with Sanger sequencing and TIDE analysis. Editing efficiency of enhancer deletion was assessed with PCR and agarose gel electrophoresis and densitometry using ImageJ (NIH).[13] Pooled guides were used to generate knockout of *MYO9B* (5'-caccaaauguuaugugcugg-3', 5'-ggugcugcuauggccccggc-3', 5'-cugcguugcgcuccugcagc-3'; assessed with amplicon of 450bp Forward primer 5'-GAGTGTGAAAGAGGCAGGCA-3' & Reverse Primer 5'-AGGTCATCAAAGTCCGCCTG-3) and *HAUS8* (5'-cccaucucccuccaggugga-3'; 5'-caggacauucgccacggccc-3'; 5'-cgagaugcucagccccuucg-3'; assessed with amplicon of 438bp Forward Primer: 5'-TGCCCAAGGTTCAAATCCCA-3' & Reverse Primer 5'-CGCTGTATCTTAACCCTGC-3') with confirmation of knockout by PCR and electrophoresis. Control guides were commercially available (Synthego). DNA was harvested (QIAgen DNAeasy blood and tissue) and regions were amplified by PCR (Qiagen HotStart Taq Polymerase) and assessed on 1-2% agarose gels stained with 1:250,000 ethidium bromide or eGels stained with Sybr Gold (Invitrogen).

1) Willer, C.J., Li, Y., and Abecasis, G.R., *METAL: fast and efficient meta-analysis of genomewide association scans.* Bioinformatics, 2010. **26**(17): p. 2190-2191.

2) Nikpay, M., et al., *A comprehensive 1000 Genomes–based genome-wide association meta-analysis of coronary artery disease.* Nat Genet, 2015. **47**(10): p. 1121-1130.

3) Howson, J.M., et al., *Fifteen new risk loci for coronary artery disease highlight arterial-wall-specific mechanisms.* Nat Genet, 2017. **49**(7): p. 1113-1119.

4) Webb, T.R., et al., *Systematic evaluation of pleiotropy identifies 6 further loci associated with coronary artery disease.* J Am Coll Card, 2017. **69**(7): p. 823-836.

5) Myocardial Infarction Genetics and CARDIoGRAM Exome Consortia Investigators, *Coding variation in ANGPTL4, LPL, and SVEP1 and the risk of coronary disease.* New Eng J Med, 2016. **374**(12): p. 1134-1144.

6) Magi, R., Lindgren, C.M., and Morris, A.P., *Meta-analysis of sex-specific genome-wide association studies.* Genet Epidemiol, 2010. **34**(8): p. 846-853.

7) Mägi, R. and Morris, A.P., *GWAMA: software for genome-wide association meta-analysis.* BMC Bioinf, 2010. **11**(1): p. 288.

8) Berglund, G., et al. *The Malmo Diet and Cancer study. Design and feasibility*. J Intern Med. 1993. **233**(1): p. 45–51.

9) Sabatine, M.S., et al., *Rationale and design of the Further cardiovascular OUtcomes Research with PCSK9 Inhibition in subjects with Elevated Risk trial.* Am Heart J, 2016. **173**: p. 94-101.

10) Weeks, E.M., et al., *Leveraging polygenic enrichments of gene features to predict genes underlying complex traits and diseases.* medRxiv, 2020: p. 2020.09.08.20190561.

11) 1000 Genomes Project Consortium, et al., *A global reference for human genetic variation.* Nature, 2015. **526**(7571): p. 68-74.

12) Atri, D.S., et al., *CRISPR-Cas9 genome editing of primary human vascular cells in vitro.* Curr Protoc, 2021. **1**(11): p. e291.

13) Gough, W., et al., *A quantitative, facile, and high-throughput image-based cell migration method is a robust alternative to the scratch assay*. J Biomol Screen. 2011. **16**(2): p. 155-63.

EPIC-CVD

EPIC-CVD is a case-cohort study nested within the European Prospective Investigation into Cancer and Nutrition (EPIC) cohort (1). The EPIC cohort consists of 366,521 women and 153,457 men, aged between 35 and 70 years at baseline, recruited from the population at 23 centres across 10 European countries (Denmark, France, Germany, Greece, Italy, The Netherlands, Norway, Spain, Sweden and the UK) between 1992 and 2000. More than 93% of participants were of European ancestry. For EPIC-CVD, a representative subcohort of 17,634 participants was selected by simple random sampling, stratified by centre, from participants who had available stored blood and buffy coat samples (n=385,747) (2, 3).

For this GWAS analysis, prevalent or incident coronary artery disease cases were compared with non-cases from the subcohort. Coronary artery disease was defined as myocardial infarction, chronic ischaemic heart disease or angina using ICD9 (410-414) or ICD10 (I20-I25) codes. Methods used in the recruitment centres to determine first non-fatal CHD and stroke events included self-report and linkage with morbidity or hospital registries. For most centres, non-fatal events were further validated by a review of medical records and/or linkage with registries. Fatal events were generally determined through mortality registries (3).

EPIC complies with the Declaration of Helsinki, and all participants gave written informed consent before participating in this study. The study was approved by the local ethics committees of the participating centres and the Institutional Review Board of the International Agency for Research on Cancer (IARC, Lyon). A list of the EPIC-CVD Principal Investigators can be found at the end of this Supplement. To avoid overlapping participants, EPIC-CVD was removed from the meta-analyses based on the CardioMetabochip and Exome array.

deCODE

The aim of deCODE genetics in Reykjavik, Iceland, is to find associations between variations in the sequence of the genome and human phenotypes. Subjects with a broad range of phenotypes, their relatives, and control subjects have been recruited continuously since 1996 through a variety of research programs, including cardiovascular (4). We assigned coronary artery disease case status based on the relevant ICD-9 and ICD-10 codes and NCSP procedure codes from Landspitali – The National University Hospital, the only tertiary hospital in Iceland, and from the Causes of Death Register. Case status is updated annually. The control group consisted of individuals free of coronary artery disease.

The study was approved by the Data Protection Authority of Iceland and the National Bioethics Committee of Iceland (Approvals No. VSNb2015080003-03.01 and VSNb2015030022-03.01 with amendments). All participants donating samples signed informed consents. Personal identities of those contributing phenotypes and biological samples were encrypted with a third party system, provided by the Data Protection Authority of Iceland. To avoid overlapping participants, deCODE was removed from the meta-analysis based on the CardioMetabochip array.

GerMIFS

The GerMIFS V cases consist of patients referred for coronary angiography, classified as MI cases, from Germany (5). Control samples were recruited as part of the Cooperative Health Research in the Augsburg Region, a population-based study to assess the health status of the population in Augsburg and the surrounding area. The GerMIFS VI cases consist of patients referred for coronary angiography, classified as CAD or MI cases based on the coronary angiogram, with a stenosis diameter of more than 50% in at least one coronary vessel from Germany (6). Control samples consist of patients referred for coronary angiography with exclusion of coronary artery disease and no history of atrial fibrillation. The GerMIFS VII cases consist of patients referred for coronary angiography, classified as CAD or MI cases, with a stenosis diameter of more than 50% in at least one coronary vessel, with a disease manifestation at young age, multivessel disease, history of previous MI/coronary artery bypass graft surgery and/or absence of traditional risk factors from Germany. Control samples were recruited as CAD-free individuals of the Heinz-Nixdorf-Recall Study which recruited samples in the area of Essen, Northrhine-Westfalia of a prospective cardio-vascular focused population-based cohort. The studies were approved by the local ethics committee and participants gave written informed consent before participating in this study, and the studies were conducted in accordance with the Declaration of Helsinki.

Greek Coronary Disease Cohort (GCC)

GCC is a case-control study conducted in Greece. CAD cases included participants hospitalized for acute coronary syndrome or diagnosed with Left Main CAD during hospitalization. Controls were drawn from the TEENs of Attica: Gene and Environment study (TEENAGE) (7) and from the Non-alcoholic Fatty Liver Disease study (NAFLD) case-control study. The GCC study had obtained local ethics approval and all patients had provided written informed consent prior to enrolment.

HUNT study

The Nord-Trøndelag Health Study (HUNT) is a population-based health survey conducted in the county of Nord-Trøndelag, Norway, where the entire county's population aged 20 years or older were invited to participate (8). Four waves of the survey have been conducted, HUNT1 [1984-1986], HUNT2 [1995-1997], HUNT3 [2006-2008], and HUNT4 [2017-2019], enrolling more than 123,000 individuals at one or more HUNT surveys with participation rates of 89%, 70%, 54%, and 54% respectively. We used a combination of hospital, out-patient, and emergency room discharge diagnoses (ICD-9 and ICD-10) to identify 7,710 CAD cases and 58,577 controls with genotype data. The 'intermediate CAD' phenotype was defined in HUNT as myocardial infarction (I21-I24, 410), chronic IHD (I25.1, I25.2, I25.5, I25.6, I25.7, I25.8, I25.9, 411, 412, 414.0, 414.8, 414.9), or self-reported history of CABG, which was used in the absence of ICD codes for CABG, triple heart bypass or PTCA. Angina cases (I20, 413) were excluded from controls. As a prospective study, we identify prevalent and incident cases. The genotyped cohort has 32748 males and 36887 females. Participation in the HUNT Study is based on informed consent, and the study has been approved by the Data Inspectorate and the Regional Ethics Committee for Medical Research in Norway. To avoid overlapping participants, HUNT was removed from the meta-analysis based on the Exome array.

Mass General Brigham Biobank

The Mass General Brigham Biobank is a large research data and sample repository comprising more than 100,000 participants that is embedded within the framework of the Mass General Brigham integrated health care system (9). Participants are prospectively enrolled in the context of outpatient visits, inpatient stays, and emergency department encounters. The Mass General Brigham Biobank contains banked samples (plasma, serum, DNA and buffy coats), genomic data, and other health information, including data from the electronic health record (EHR) at hospitals affiliated with the Mass General Brigham healthcare system – primarily the Massachusetts General Hospital and the Brigham and Women's Hospital. Array-based genotyping was performed using either the Illumina Multi-Ethnic Genotyping Array, Expanded Multi-Ethnic Genotyping Array, or the Multi-Ethnic Global BeadChip Array (Illumina, Inc., San Diego, CA). We studied the first 13,667 genotyped participants from the Mass General Brigham Biobank with relevant clinical data available.

TIMI

Three cardiovascular outcomes trials from the TIMI Study Group were included in the GWAS analysis: PEGASUS-TIMI 54, ENGAGE AF-TIMI 48, and SAVOR-TIMI 53. These trials contributed a combined 26,737 patients (17,887 cases and 8,850 controls).

The PEGASUS-TIMI 54 trial was a multinational, randomized, double-blind, placebo-controlled trial of the efficacy of ticagrelor among patients with prior MI (10). The inclusion criteria were age of at least 50 years old with one additional high-risk feature: 65 years or older, diabetes, a second MI, multivessel coronary artery disease, or renal dysfunction. Exclusion criteria included known bleeding disorder, gastrointestinal bleeding within 6 months, history of ischemic stroke, intracranial bleeding, central nervous system abnormality, surgery within 30 days, or planned use of $P2Y_{12}$ receptor antagonist, dipyridamole, cilostazol, or an anticoagulant. Patients were randomized 1:1:1 to either ticagrelor 90 mg twice daily, 60 mg twice daily, or placebo and followed for a median of 2.8 years. The mean age of the study population was 65 years old and 76% were men. Comorbidities included smoking (17%), hypertension (78%), diabetes (32%), prior PCI (83%), and prior CABG (60%). All 10,607 patients who consented for genetic analysis, passed QC, and were of European ancestry were included in this GWAS analysis.

ENGAGE AF-TIMI 48 was a 3-arm multinational, randomized, double-blind, placebo-controlled trial comparing two doses of the Xa inhibitor edoxaban to warfarin in patients with atrial fibrillation (11). The inclusion criteria were age of at least 21 years old with atrial fibrillation, a $CHADS_2$ score of 2 or higher, and on anticoagulation. Exclusion criteria included atrial fibrillation with reversible etiology, kidney dysfunction, high risk of bleeding, use of DAPT, significant mitral stenosis, or recent cardiovascular events. Patients were randomized 1:1:1 to either warfarin, low dose edoxaban, or high dose edoxaban and followed for a median of 2.8 years. The mean age of the study population was 72 years old and 62% were men. Comorbidities included diabetes (38%), stroke (28%), and heart failure (57%). All 9,647 patients who consented for genetic analysis, passed QC, and were of European ancestry were included in this GWAS analysis.

The SAVOR-TIMI 53 trial was a multinational, randomized, double-blind, placebo-controlled trial of the DPP4 inhibitor saxagliptin in diabetics (12). The inclusion criteria were age of at least 40 years with type 2 diabetes mellitus, a glycated hemoglobin of 6.5% to 12.0%, and either established cardiovascular disease or multiple cardiovascular risk factors. Key exclusion criteria included end-stage renal disease, kidney transplantation, or incretin-based therapy within 6 months. Patients were randomized 1:1 to receive saxagliptin or placebo and followed for a median of 2.9 years. The mean age of the study population was 65 years old and 67% were men. Comorbidities included atherosclerosis (78%) and HTN (81%). All 6,483 patients who consented for genetic analysis, passed QC, and were of European ancestry were included in this GWAS analysis.

UK Biobank

UK Biobank (UKB) is a prospective study of approximately 500,000 individuals comprising members of the UK population aged 40-69 collected from multiple sites across the United Kingdom from 2006-2010 (14). For GWAS we utilized version 3 of the UK Biobank genotype data which was imputed to 1000 Genomes, UK10K, and Haplotype Reference Consortium panels (https://doi.org/10.1038/s41586-018-0579-z, 10.1038/ng.3679) for 472,335 individuals of European ancestry that had not withdrawn consent for study as of April, 2018. 33,941 cases were defined as prevalent or incident MI or intervention (CABG, PTCA) ascertained from Hospital Episode Statistics or self-report; or IHD death ascertained from Office of National Statistic. 438,394 controls were taken from the remaining population considered to be CAD and angina free. The minimum MAF for testing was $1x10^{-6}$.

1) E. Riboli, K. Hunt, N. Slimani, P. Ferrari, T. Norat et al., *European Prospective Investigation into Cancer and Nutrition (EPIC): study populations and data collection,* Public Health Nutrition, vol.59, issue.6b, pp.1113-1137, 2002.
2) Langenberg C, Sharp S, Forouhi NG, Franks PW, Schulze MB, et al. (2011) *Design and cohort description of the InterAct Project: an examination of the interaction of genetic and lifestyle factors on the incidence of type 2 diabetes in the EPIC Study*. Diabetologia 54: 2272–2282.
3) Danesh J, Saracci R, Berglund G et al. *EPIC-Heart: the cardiovascular component of a prospective study of nutritional, lifestyle and biological factors in 520,000 middle-aged participants from 10 European countries*. Eur J Epidemiol 2007;22:129–41.
4) Helgadottir A, Thorleifsson G, Manolescu A, et al. *A common variant on chromosome 9p21 affects the risk of myocardial infarction.* Science. 2007;316(5830):1491-1493.
5) Brænne I, Zeng L, Willenborg C, et al. *Genomic correlates of glatiramer acetate adverse cardiovascular effects lead to a novel locus mediating coronary risk*. PLoS One. 2017 12(8):e0182999.
6) Roselli C, Chaffin MD, Weng L-C, et al. *Multi-ethnic genome-wide association study for atrial fibrillation*. Nat Genet. 2019 50(9):1225-1233.
7) Ntalla I, Giannakopoulou M, Vlachou P, et al. *Body composition and eating behaviours in relation to dieting involvement in a sample of urban Greek adolescents from the TEENAGE (TEENs of Attica: Genes & Environment) study.* Public Health Nutr. 2014 17(3):561-8.
8) Krokstad, S. et al. *Cohort Profile: The HUNT Study, Norway.* Int. J. Epidemiol. 42, 968–977 (2013).
9) Gainer VS, Cagan A, Castro VM, et al. *The Biobank Portal for Partners Personalized Medicine: A Query Tool for Working with Consented Biobank Samples, Genotypes, and Phenotypes Using i2b2*. J Pers Med. 2016 26;6(1):11.
10) Bonaca MP, Bhatt DL, Cohen M, et al. *Long-Term Use of Ticagrelor in Patients with Prior Myocardial Infarction.* N Engl J Med 2015;372:1791-1800.
11) Giugliano RP, Ruff CT, Braunwald E, et al. *Edoxaban versus Warfarin in Patients with Atrial Fibrillation*. N Engl J Med 2013; 369:2093-2104.
12) Scirica BM, Bhatt DL, Braunwald E, et al. *Saxagliptin and Cardiovascular Outcomes in Patients with Type 2 Diabetes Mellitus.* N Engl J Med 2013;369:1317-1326.
13) Sudlow C, Gallacher J, Allen N, et al. *UK Biobank: an open access resource for identifying the causes of a wide range of complex diseases of middle and old age*. PLoS Med. 2015 12(3):e1001779.
14) Grace C, Farrall M, Watkins H, Goel A. *Manhattan++: displaying genome-wide association summary statistics with multiple annotation layers*. BMC Bioinformatics. 2019 20(1):610.

1) CARDIoGRAMplusC4D Consortium Membership 2022 (alphabetical order).
Affiliations at time of contribution to CARDIoGRAMplusC4D

**Krishna G Aragam**	Cardiovascular Research Center, Massachusetts General Hospital; Center for Genomic Medicine, Massachusetts General Hospital, Boston MA USA; Cardiovascular Disease Initiative, Broad Institute of MIT and Harvard; Program in Medical and Population Genetics Broad Institute of MIT and Harvard Cambridge MA USA
**David O Arnar**	deCODE genetics/Amgen, Inc. Reykjavik, Iceland; Faculty of Medicine, University of Iceland, Reykjavik, Iceland, Department of Internal Medicine, Division of Cardiology, Landspitali – National University Hospital of Iceland, Reykjavik, Iceland
**Themistocles L Assimes**	Department of Medicine, Stanford University School of Medicine, Stanford, California, USA
**Bjørn O Åsvold**	K.G. Jebsen Center for Genetic Epidemiology, Department of Public Health and Nursing, Norwegian University of Science and Technology, NTNU Trondheim, Norway; HUNT Research Centre, Norwegian University of Science and Technology, Levanger Norway; Department of Endocrinology, Clinic of Medicine, St. Olavs Hospital, Trondheim, Norway
**Deepak S Atri**	Divisions of Cardiovascular Medicine and Genetics, Brigham and Women's Hospital, Harvard Medical School, Broad Institute of MIT and Harvard, Cardiovascular Disease Initiative, Broad Institute of MIT and Harvard, Cambridge, MA, USA
**Aris Baras**	Regeneron Genetics Center, Regeneron Pharmaceuticals, Tarrytown, NY, USA
**Johan LM Björkegren**	Clinical Gene Networks AB, Stockholm, Sweden; Department of Genetics & Genomic Sciences, Institute of Genomics and Multiscale Biology, Icahn School of Medicine at Mount Sinai, New York, USA; Integrated Cardio Metabolic Centre, Karolinska Institutet, Karolinska Universitetssjukhuset, Huddinge, Sweden
**Stefan Blankenberg**	University Heart Center Hamburg, Clinic for general and interventional Cardiology, Hamburg, Germany
**Bernhard O Boehm**	Division of Endocrinology and Diabetes, Department of Internal Medicine, Ulm University Medical Centre, Ulm, Germany
**Eric Boerwinkle**	Human Genetics Center, Department of Epidemiology, Human Genetics, and Environmental Sciences, School of Public Health, The University of Texas Health Science Center at Houston, Houston, TX, USA; Human Genome Sequencing Center, Baylor College of Medicine, Houston, TX, USA
**Michael R Brown**	Human Genetics Center, Department of Epidemiology, Human Genetics, and Environmental Sciences, School of Public Health, The University of Texas Health Science Center at Houston, Houston, TX, USA
**Ben Brumpton**	K.G. Jebsen Center for Genetic Epidemiology, Department of Public Health and Nursing, Norwegian University of Science and Technology, NTNU, Trondheim, Norway; HUNT Research Centre, Norwegian University of Science and Technology, Levanger, Norway
**Noël P Burtt**	Program in Medical and Population Genetics, Broad Institute of MIT and Harvard, Cambridge, MA, USA
**Adam S Butterworth**	BHF Cardiovascular Epidemiology Unit, Department of Public Health and Primary Care, University of Cambridge, Cambridge, UK; Health Data Research UK Cambridge, Wellcome Genome Campus and University of Cambridge, Cambridge, UK; British Heart Foundation Centre of Excellence, Division of Cardiovascular Medicine, Addenbrooke's Hospital, Cambridge, UK; National Institute for Health Research Cambridge Biomedical Research Centre, Cambridge University Hospitals, Cambridge,

UK; The National Institute for Health Research Blood and Transplant Unit (NIHR BTRU) in Donor Health and Genomics at the University of Cambridge, Cambridge, UK

John C Chambers Epidemiology and Biostatistics, Imperial College London, London, UK

Robert Clarke CTSU - Nuffield Department of Population Health, Medical Sciences Division, University of Oxford, Oxford, UK

Rory Collins CTSU - Nuffield Department of Population Health, Medical Sciences Division, University of Oxford, Oxford, UK

Maria C Costanzo Program in Medical and Population Genetics, Broad Institute of MIT and Harvard, Cambridge, MA, USA

John Danesh BHF Cardiovascular Epidemiology Unit, Department of Public Health and Primary Care, University of Cambridge; Health Data Research UK, Wellcome Genome Campus and University of Cambridge; The National Institute for Health Research Blood and Transplant Unit (NIHR BTRU) in Donor Health and Genomics at the University of Cambridge, Cambridge, UK; Human Genetics, Wellcome Sanger Institute, Saffron Walden, UK; National Institute for Health Research Cambridge Biomedical Research Centre, Cambridge University Hospitals, Cambridge, UK; British Heart Foundation Centre of Excellence, Division of Cardiovascular Medicine, Addenbrooke's Hospital, Cambridge, UK

Paul S de Vries Human Genetics Center, Department of Epidemiology, Human Genetics, and Environmental SciencesSchool of Public Health, The University of Texas Health Science Center at Houston, Houston, TX, USA

George Dedoussis School of Health Science & Education, Department of Nutrition-Dietetics, Harokopio University, Athens, Greece

Panos Deloukas William Harvey Research Institute, Barts and the London School of Medicine and Dentistry, Queen Mary University of London, London, U; KPrincess Al-Jawhara Al-Brahim Centre of Excellence in Research of Hereditary Disorders (PACER-HD) King Abdulaziz University, Jeddah, Saudi Arabia; Princess Al-Jawhara Al-Brahim Centre of Excellence in Research of Hereditary Disorders (PACER-HD) King Abdulaziz University, Jeddah, Saudi Arabia

Patrick T Ellinor Cardiovascular Research Center, Massachusetts General Hospital, Boston, MA, USA; Cardiovascular Disease Initiative, Broad Institute of MIT and Harvard, Cambridge, MA, USA

Jeanette Erdmann Institute for Cardiogenetics University of Lübeck, Lübeck, Germany; DZHK (German Research Center for Cardiovascular Research), partner site Hamburg-Lübeck-Kiel, Germany

Martin Farrall Radcliffe Department of Medicine, Division of Cardiovascular Medicine, University of Oxford; Wellcome Centre for Human Genetics, University of Oxford, Oxford, UK

Hilary K Finucane Program in Medical and Population Genetics, Broad Institute of MIT and Harvard, Cambridge, MA, USA; Analytic and Translational Genetics Unit, Massachusetts General Hospital, Boston, Massachusetts, USA; Stanley Center for Psychiatric Research, Broad Institute of MIT and Harvard, Cambridge, MA, USA

Jason Flannick Division of Genetics and Genomics, Boston Children's Hospital, Boston, MA, USA

Paul W Franks Department of Clinical Sciences, Genetic & Molecular Epidemiology Unit, Lund University Diabetes Center, Skåne University Hosptial, Malmö, Sweden Department of Public Health & Clinical Medicine, Genetic Epidemiology & Clinical Research Group, Section for Medicine, Umeå University, Umeå, Sweden; Department of Nutrition, Harvard School of Public Health, Boston, MA USA

**Olga Giannakopoulou**	William Harvey Research Institute, Barts and the London School of Medicine and Dentistry, Queen Mary University of London, London, UK
**Panagiota Giardoglou**	School of Health Science & Education, Department of Nutrition-Dietetics, Harokopio University, Athens, Greece
**Anuj Goel**	Radcliffe Department of MedicineDivision of Cardiovascular MedicineUniversity of Oxford; Wellcome Centre for Human Genetics, University of Oxford, Oxford, UK
**Christopher Grace**	Radcliffe Department of Medicine, Division of Cardiovascular Medicine, University of Oxford; Wellcome Centre for Human Genetics, University of Oxford, Oxford, UK
**Dongfeng Gu**	Department of Population Genetics and Prevention, Fu Wai Hospital & Cardiovascular Institute, Chinese Academy of Medical Sciences & Peking Union Medical College, Beijing, China
**Daniel F Gudbjartsson**	deCODE genetics/Amgen, Inc. Reykjavik Iceland; School of Engineering and Natural Sciences, University of Iceland, Reykjavik, Iceland
**Ulrich Güldener**	German Heart Centre Munich, Department of Cardiology, Technical University of Munich, Munich, Germany
**Rajat M Gupta**	Divisions of Cardiovascular Medicine and Genetics, Brigham and Women's Hospital, Harvard Medical School, Boston, MA, USA; Cardiovascular Disease Initiative, Broad Institute of MIT and Harvard, Cambridge, MA, USA; Program in Medical and Population Genetics, Broad Institute of MIT and Harvard, Cambridge, MA, USA
**Syed M. Ijlal Haider**	Institute for Cardiogenetics, University of Lübeck, Lübeck, Germany
**Anders Hamsten**	Atherosclerosis Research Unit, Department of Medicine Solna, Karolinska Institutet, Stockholm, Sweden Center for Molecular Medicine and Department of Cardiology, Karolinska University Hospital, Stockholm, Sweden
**Anna Helgadottir**	deCODE genetics/Amgen, Inc.Reykjavik, Iceland
**George Hindy**	Department of Population Medicine, Qatar University College of Medicine, Doha, Qatar
**Hilma Holm**	deCODE genetics/Amgen, Inc. Reykjavik, Iceland
**Jemma C Hopewell**	CTSU - Nuffield Department of Population Health, Medical Sciences Division, University of Oxford, Oxford, UK
**G Kees Hovingh**	Department of Vascular Medicine, Academic Medical Center, Amsterdam, The Netherlands
**Kristian Hveem**	K.G. Jebsen Center for Genetic Epidemiology, Department of Public Health and Nursing, Norwegian University of Science and Technology, NTNU, Trondheim, Norway; HUNT Research Centre, Norwegian University of Science and Technology, Levanger, Norway
**Maysson Ibrahim**	CTSU - Nuffield Department of Population Health, Medical Sciences Division, University of Oxford, Oxford, UK
**Erik Ingelsson**	Department of Medical Epidemiology and Biostatistics, Karolinska Institutet, Stockholm, Sweden
**Kazuyoshi Ishigaki**	Laboratory for Statistical and Translational Genetics, RIKEN Center for Integrative Medical Sciences,Yokohama Kanagawa, Japan
**Kaoru Ito**	Laboratory for Cardiovascular Genomics and Informatics, RIKEN Center for Integrative Medical Sciences, Yokohama Kanagawa, Japan
**Dong-Keun Jang**	Program in Medical and Population Genetics, Broad Institute of MIT and Harvard, Cambridge, MA, USA
**Tao Jiang**	BHF Cardiovascular Epidemiology Unit, Department of Public Health and Primary Care, University of Cambridge, Cambridge UK

**Frederick K Kamanu**	TIMI Study Group, Division of Cardiovascular Medicine, Brigham and Women's Hospital, Harvard Medical School, Boston, Massachusetts, USA
**Yoichiro Kamatani**	Department of Computational Biology and Medical Sciences, Graduate School of Frontier Sciences, The University of Tokyo, Tokyo, Japan
**Stavroula Kanoni**	William Harvey Research Institute, Barts and the London School of Medicine and Dentistry, Queen Mary University of London, London, UK
**Adnan Kastrati**	German Heart Centre Munich, Department of Cardiology, Technical University of Munich; German Research Center for Cardiovascular Research (DZHK e.V.), partner site Munich Heart Alliance,Munich Germany
**Sekar Kathiresan**	Verve Therapeutics, Cambridge, MA, USA; Cardiovascular Research Center and Center for Human Genetic Research, Massachusetts General Hospital, Boston, Massachusetts, USA; Broad Institute, Cambridge, Massachusetts, USA; Department of Medicine, Harvard Medical School, Boston, Massachusetts, USA
**Thorsten Kessler**	German Heart Centre Munich, Department of Cardiology, Technical University of Munich; German Research Center for Cardiovascular Research (DZHK e.V.), partner site Munich Heart Alliance,Munich Germany
**Amit V Khera**	Center for Genomic Medicine, Massachusetts General Hospital, Boston, MA, USA; Cardiovascular Disease Initiative, Broad Institute of MIT and Harvard, Cambridge, MA, USA; Program in Medical and Population Genetics, Broad Institute of MIT and Harvard, Cambridge MA, USA
**Bong-Jo Kim**	Division of Structural and Functional Genomics Center for Genome Science, Korea National Institute of Health, Korea
**Issei Komuro**	Department of Cardiovascular Medicine, The University of Tokyo, Tokyo, Japan
**Tomasz Konopka**	William Harvey Research Institute, Barts and the London School of Medicine and Dentistry, Queen Mary University of London, London, UK
**Jaspal S Kooner**	National Heart and Lung Institute (NHLI), Imperial College London, Hammersmith Hospital, London, UK.
**Satoshi Koyama**	Laboratory for Cardiovascular Genomics and Informatics, RIKEN Center for Integrative Medical Sciences, Yokohama Kanagawa, Japan
**Iftikhar J Kullo**	Department of Cardiovascular Medicine, Mayo Clinic, MN, USA
**Theodosios Kyriakou**	Wellcome Centre for Human Genetics, University of Oxford, Oxford, UK
**Terho Lehtimäki**	Department of Clinical Chemistry, Fimlab Laboratories, Tampere University Hospital, Tampere, Finland; Department of Clinical Chemistry, University of Tampere School of Medicine, Tampere, Finland
**Ling Li**	German Heart Centre Munich, Department of Cardiology, Technical University of Munich, Munich, Germany
**Luca A Lotta**	Regeneron Genetics Center, Regeneron Pharmaceuticals, Tarrytown, NY, USA
**Lijiang Ma**	Department of Genetics and Genomic Science, Icahn Institute for Genomics and Multiscale Biology, Icahn School of Medicine at Mount Sinai; The Zena and Michael A. Wiener Cardiovascular Institute, Icahn School of Medicine at Mount Sinai, New York, NY, USA
**Nicholas A Marston**	TIMI Study Group, Division of Cardiovascular Medicine, Brigham and Women's Hospital, Harvard Medical School, Boston, Massachusetts, USA
**Winfried März**	Synlab Academy, Mannheim, Germany; Medical Clinic V (Nephrology, Hypertensiology, Endocrinology, Diabetology, Rheumatology), Medical Faculty of Mannheim, University of Heidelberg, Mannheim, Germany

**Ruth McPherson**	Department of Medicine, Division of Cardiology, University of Ottawa Heart Institute, Ottawa, Canada
**Thomas Meitinger**	Institute of Human Genetics, Helmholtz Zentrum München, German Research Center for Environmental Health, Neuherberg, Germany; Klinikum rechts der IsarInstitute of Human Genetics, Technical University of Munich, Munich, Germany, German Research Center for Cardiovascular Research (DZHK e.V.), partner site Munich Heart Alliance, Munich, Germany
**Olle Melander**	Department of Clinical Sciences in Malmö, Lund University, Malmo, Sweden
**Andres Metspalu**	Estonian Genome Center, University of Tartu, Tartu, Estonia; Institute of Molecular and Cell Biology, University of Tartu, Tartu, Estonia
**Alanna C Morrison**	Human Genetics Center, Department of Epidemiology, Human Genetics, and Environmental Sciences, School of Public Health, The University of Texas Health Science Center at Houston, Houston, TX, USA
**Sören Mucha**	Institute for Cardiogenetics, University of Lübeck, Lübeck, Germany
**Loreto Muñoz Venegas**	Institute for Cardiogenetics, University of Lübeck; DZHK (German Research Center for Cardiovascular Research), partner site Hamburg-Lübeck-Kiel, Lübeck, Germany
**Matthias Munz**	Institute for Cardiogenetics, University of Lübeck, Lübeck, Germany
**Federico Murgia**	CTSU - Nuffield Department of Population Health, Medical Sciences Division, University of Oxford, Oxford, UK
**Christopher P Nelson**	Department of Cardiovascular Sciences and NIHR Leicester Biomedical Research Centre, University of Leicester, Leicester UK
**Jonas B Nielsen**	Department of Internal Medicine, CardiologyUniversity of Michigan, Ann Arbor, Michigan, US; K.G. Jebsen Center for Genetic Epidemiology, Department of Public Health and Nursing, Norwegian University of Science and Technology, NTNU, Trondheim, Norway
**Markku S Nieminen**	Division of Cardiology, Department of Medicine, Helsinki University Central Hospital (HUCH), Helsinki, Finland
**Markus M Nöthen**	School of Medicine & University Hospital Bonn, Institute of Human Genetics, University of Bonn, Bonn, Germany
**Christopher O'Donnell**	Framingham Heart Study, National Heart, Lung and Blood Institute, Framingham, MA 01702, USA
**Marju Orho-Melander**	Department of Clinical Sciences in Malmö, Lund University, Malmo, Sweden
**Colin N A Palmer**	Medical Research Institute, University of Dundee, Ninewells Hospital and Medical School, Dundee, UK
**Shichao Pang**	German Heart Centre Munich, Department of Cardiology, Technical University of Munich, Munich, Germany
**Markus Perola**	Department of Chronic Disease Prevention, National Institute for Health and Welfare, Helsinki, Finland
**Loukianos S Rallidis**	Second Department of Cardiology, Medical School, National and Kapodistrian University of Athens, University General Hospital Attikon, Athens, Greece
**Muredach P Reilly**	Cardiovascular Institute, Perelman School of Medicine at the University of Pennsylvania, Philadelphia, USA
**Tobias Reinberger**	Institute for Cardiogenetics, University of Lübeck, Lübeck, Germany
**Samuli Ripatti**	Department of Chronic Disease Prevention, National Institute for Health and Welfare, Helsinki FIN-00271, Finland; Wellcome Trust Sanger Institute, Hinxton, UK
**Robert Roberts**	Cardiovascular Genomics & Genetics, University of Arizona, College of Medicine, Phoenix, Arizona, USA; University of Ottawa Heart Institute, Cardiovascular Research

	Methods Centre and Ruddy Canadian Cardiovascular Genetics Centre, Ontario, Canada
**Carolina Roselli**	Cardiovascular Disease Initiative, Broad Institute of MIT and Harvard, Cambridge, MA, USA
**Christian T Ruff**	TIMI Study Group, Division of Cardiovascular Medicine, Brigham and Women's Hospital, Harvard Medical School, Boston, Massachusetts, USA
**Arno Ruusalepp**	Department of Cardiac Surgery, Tartu University Hospital and Institute of Clinical Medicine, Tartu University, Tartu, Estonia
**Marc S Sabatine**	TIMI Study Group, Division of Cardiovascular Medicine, Brigham and Women's Hospital, Harvard Medical School, Boston, Massachusetts, USA
**Danish Saleheen**	Center for Non-Communicable Diseases, Karachi, Pakistan; Department of Public Health and Primary Care, University of Cambridge, Cambridge, UK; Department of Medicine, University of Pennsylvania, Philadelphia, Pennsylvania, USA
**Nilesh J Samani**	Department of Cardiovascular Sciences and NIHR Leicester Biomedical Research Centre, University of Leicester, Leicester, UK
**Manjinder S Sandhu**	MRC Epidemiology Unit, Institute of Metabolic Science, Addenbrooke's Hospital, Cambridge, UK; Wellcome Trust Sanger Institute, Hinxton, UK
**Gavin Schnitzler**	Cardiovascular Disease Initiative, Broad Institute
**Stefan Schreiber**	Institut für Klinische Molekularbiologie, Christian-Albrechts Universität, Kiel, Germany
**Heribert Schunkert**	German Heart Centre Munich, Department of Cardiology, Technical University of Munich, Munich, Germany; German Research Center for Cardiovascular Research (DZHK e.V.), partner site Munich Heart Alliance, Munich, Germany
**Paul Sherliker**	Medical Research Council Population Health Research Unit, CTSU - Nuffield Department of Population Health, Medical Sciences Division, University of Oxford, Oxford, UK
**Agneta Siegbahn**	Uppsala Clinical Research Center, Uppsala University, Uppsala, Sweden
**Damian Smedley**	William Harvey Research Institute, Barts and the London School of Medicine and Dentistry, Queen Mary University of London, London, UK
**Kari Stefansson**	deCODE genetics/Amgen, Inc. Reykjavik, Iceland; Faculty of Medicine, University of Iceland, Reykjavik, Iceland
**Ida Surakka**	Department of Internal Medicine, Cardiology, University of Michigan, Ann Arbor, Michigan USA
**Gudmundur Thorgeirsson**	deCODE genetics/Amgen, Inc., Faculty of Medicine, University of Iceland; Department of Internal Medicine, Division of Cardiology, Landspitali – National University Hospital of Iceland, Reykjavik, Iceland
**Gudmar Thorleifsson**	deCODE genetics/Amgen, Inc. Reykjavik, Iceland
**Unnur Thorsteinsdottir**	deCODE genetics/Amgen, Inc.; Faculty of Medicine, University of Iceland, Reykjavik, Iceland
**Jacob K Ulirsch**	Program in Medical and Population Genetics, Broad Institute of MIT and Harvard, Cambridge, MA, USA; Analytic and Translational Genetics Unit, Massachusetts General Hospital, Boston, Massachusetts, USA; Program in Biological and Biomedical Sciences, Harvard Medical School, Boston, Massachusetts, USA;
**Moritz von Scheidt**	German Heart Centre Munich, Department of Cardiology, Technical University of Munich; German Research Center for Cardiovascular Research (DZHK e.V.), partner site Munich Heart Alliance, Munich, Germany

**Minxian Wang**	Cardiovascular Disease Initiative, Broad Institute of MIT and Harvard; Program in Medical and Population Genetics Broad Institute of MIT and Harvard, Cambridge MA USA
**Hugh Watkins**	Radcliffe Department of Medicine, Division of Cardiovascular Medicine, University of Oxford; Wellcome Centre for Human Genetics, University of Oxford, Oxford, UK
**Thomas R Webb**	Department of Cardiovascular Sciences and NIHR Leicester Biomedical Research Centre, University of Leicester, Leicester, UK
**Elle M Weeks**	Program in Medical and Population Genetics, Broad Institute of MIT and Harvard,Cambridge MA USA
**Cristen J Willer**	Department of Internal Medicine, Cardiology, University of Michigan, Ann Arbor, Michigan, USA; Department of Computational Medicine & Bioinformatics, University of Michigan, Ann Arbor, Michigan, USA; Department of Human Genetics, University of Michigan, Ann Arbor, Michigan, USA
**Brooke N Wolford**	Department of Computational Medicine & Bioinformatics, University of Michigan, Ann Arbor Michigan USA
**Pierre A Zalloua**	Khalifa University, College of Medicine and Health Sciences, Abu Dhabi, UAE; Harvard T.H.Chan School of Public Health, Boston, MA, USA
**Wei Zhou**	Program in Medical and Population Genetics, Broad Institute of MIT and Harvard, Cambridge MA USA; Analytic and Translational Genetics Unit, Massachusetts General Hospital, Boston, Massachusetts, USA; Stanley Center for Psychiatric Research Broad Institute of MIT and Harvard, Cambridge, MA, USA; Department of Computational Medicine & Bioinformatics, University of Michigan, Ann Arbor, Michigan, USA

2) EPIC-CVD Consortium

Principal investigators of the EPIC-CVD Consortium include the following:

Kim Overvad, Christina Dahm, Anne Tjønneland, Marie-Christine Boutron, Rudolf Kaaks, Matthias Schulze, Pietro Ferrari, Giovanna Masala, Vittorio Krogh, Salvatore Panico, Rosario Tumino, Giuseppe Matullo, Carlotta Sacerdote, Jolanda Boer, Yvonne van der Schouw, Guri Skeie, J. Ramon Quiros, María-José Sánchez Pérez, Conchi Moreno-Iribas, María Dolores Chirlaque, Pilar Amiano, Liher Imaz, Olle Melander, Patrik Wennberg, Nicholas J. Wareham, Timothy J. Key, Elisabete Weiderpass, Elio Riboli, Adam S. Butterworth and John Danesh.

The coordination of EPIC is financially supported by International Agency for Research on Cancer (IARC) and also by the Department of Epidemiology and Biostatistics, School of Public Health, Imperial College London which has additional infrastructure support provided by the NIHR Imperial Biomedical Research Centre (BRC). The national cohorts are supported by: Danish Cancer Society (Denmark); Ligue Contre le Cancer, Institut Gustave Roussy, Mutuelle Générale de l'Education Nationale, Institut National de la Santé et de la Recherche Médicale (INSERM) (France), German Cancer Aid, German Cancer Research Center (DKFZ), German Institute of Human Nutrition Potsdam-Rehbruecke (DIfE), Federal Ministry of Education and Research (BMBF) (Germany); Associazione Italiana per la Ricerca sul Cancro-AIRC-Italy, Compagnia di SanPaolo and National Research Council (Italy); Dutch Ministry of Public Health, Welfare and Sports (VWS), Netherlands Cancer Registry (NKR), LK Research Funds, Dutch Prevention Funds, Dutch ZON (Zorg Onderzoek Nederland), World Cancer Research Fund (WCRF), Statistics Netherlands (The Netherlands); Health Research Fund (FIS) - Instituto de Salud Carlos III (ISCIII), Regional Governments of Andalucía, Asturias, Basque Country, Murcia and Navarra, and the Catalan Institute of Oncology - ICO (Spain); Swedish Cancer Society, Swedish Research Council and County Councils of Skåne and Västerbotten (Sweden); Cancer Research UK (14136 to EPIC-Norfolk; C8221/A29017 to EPIC-Oxford), Medical Research Council (1000143 to EPIC-Norfolk; MR/M012190/1 to EPIC-Oxford) (United Kingdom).

We thank all EPIC participants and staff for their contribution to the study, the laboratory teams at the Medical Research Council Epidemiology Unit for sample management, Cambridge Genomic Services for genotyping, Matthew Walker and Sarah Spackman (BHF Cardiovascular Epidemiology Unit) for data management and the team at the EPIC-CVD Coordinating Centre for study coordination and administration.

3) BioBank Japan Project Consortium

Koichi Matsuda.
Laboratory of Genome Technology, Human Genome Center, Institute of Medical Science, The University of Tokyo, Tokyo, Japan.
Laboratory of Clinical Genome Sequencing, Graduate School of Frontier Sciences, The University of Tokyo, Tokyo, Japan.
kmatsuda@edu.k.u-tokyo.ac.jp

Yuji Yamanashi.
Division of Genetics, The Institute of Medical Science, The University of Tokyo, Tokyo, Japan.
yyamanas@ims.u-tokyo.ac.jp

Yoichi Furukawa.
Division of Clinical Genome Research, Institute of Medical Science, The University of Tokyo, Tokyo, Japan.
furukawa@ims.u-tokyo.ac.jp

Takayuki Morisaki.
Division of Molecular Pathology IMSUT Hospital, Department of Internal Medicine Project Division of Genomic Medicine and Disease Prevention The Institute of Medical Science The University of Tokyo, Tokyo, Japan.
morisaki@ims.u-tokyo.ac.jp

Yoshinori Murakami.
Department of Cancer Biology, Institute of Medical Science, The University of Tokyo, Tokyo, Japan.
ymurakam@ims.u-tokyo.ac.jp

Kaori Muto.
Department of Public Policy, Institute of Medical Science, The University of Tokyo, Tokyo, Japan.
krmt@ims.u-tokyo.ac.jp

Akiko Nagai.
Department of Public Policy, Institute of Medical Science, The University of Tokyo, Tokyo, Japan.
akikongi@ims.u-tokyo.ac.jp

Wataru Obara.
Department of Urology, Iwate Medical University, Iwate, Japan.
watao@iwate-med.ac.jp

Ken Yamaji.
Department of Internal Medicine and Rheumatology, Juntendo University Graduate School of Medicine, Tokyo, Japan.
k.yamaji@juntendo.ac.jp

Kazuhisa Takahashi.
Department of Respiratory Medicine, Juntendo University Graduate School of Medicine, Tokyo, Japan.
kztakaha@juntendo.ac.jp

Satoshi Asai.
Division of Pharmacology, Department of Biomedical Science, Nihon University School of Medicine, Tokyo, Japan.
Division of Genomic Epidemiology and Clinical Trials, Clinical Trials Research Center, Nihon University. School of Medicine, Tokyo, Japan.
asai.satoshi@nihon-u.ac.jp

Yasuo Takahashi.
Division of Genomic Epidemiology and Clinical Trials, Clinical Trials Research Center, Nihon University School of Medicine, Tokyo, Japan.
takahashi.yasuo@nihon-u.ac.jp

Takao Suzuki.
Tokushukai Group, Tokyo, Japan.
takao.suzuki@tokushukai.jp

Nobuaki Sinozaki.
Tokushukai Group, Tokyo, Japan.
nobuaki.shinozaki@tokushukai.jp

Hiroki Yamaguchi.
Departmentof Hematology, Nippon Medical School, Tokyo, Japan.
y-hiroki@fd6.so-net.ne.jp

Shiro Minami.
Department of Bioregulation, Nippon Medical School, Kawasaki, Japan.
shirom@nms.ac.jp

Shigeo Murayama.
Tokyo Metropolitan Geriatric Hospital and Institute of Gerontology, Tokyo, Japan.
smurayam@bbarjp.net

Kozo Yoshimori.
Fukujuji Hospital, Japan Anti-Tuberculosis Association, Tokyo, Japan.

yoshimorik@fukujuji.org

Satoshi Nagayama.
The Cancer Institute Hospital of the Japanese Foundation for Cancer Research, Tokyo, Japan.
snagayama2009@hotmail.co.jp

Daisuke Obata.
Center for Clinical Research and Advanced Medicine, Shiga University of Medical Science, Shiga, Japan.
dobata@belle.shiga-med.ac.jp

Masahiko Higashiyama.
Department of General Thoracic Surgery, Osaka International Cancer Institute, Osaka, Japan.
higashiyama-m@higashiosaka-hosp.jp

Akihide Masumoto.
IIZUKA HOSPITAL, Fukuoka,Japan.
amasumotoh1@aih-net.com

Yukihiro Koretsune.
National Hospital Organization Osaka National Hospital, Osaka, Japan.
koretune318@hotmail.co.jp

Yukihide Momozawa.
Laboratory for Genotyping Development, RIKEN Center for Integrative Medical Sciences, Yokohama, Japan.
momozawa@riken.jp

Chikashi Terao.
Laboratory for Statistical and Translational Genetics, RIKEN Center for Integrative Medical Sciences, RIKEN, Yokohama, Japan.
chikashi.terao@riken.jp

SUPPLEMENTARY FIGURE 1 Predictors for most likely causal genes strongly prioritized by three agreeing predictors.

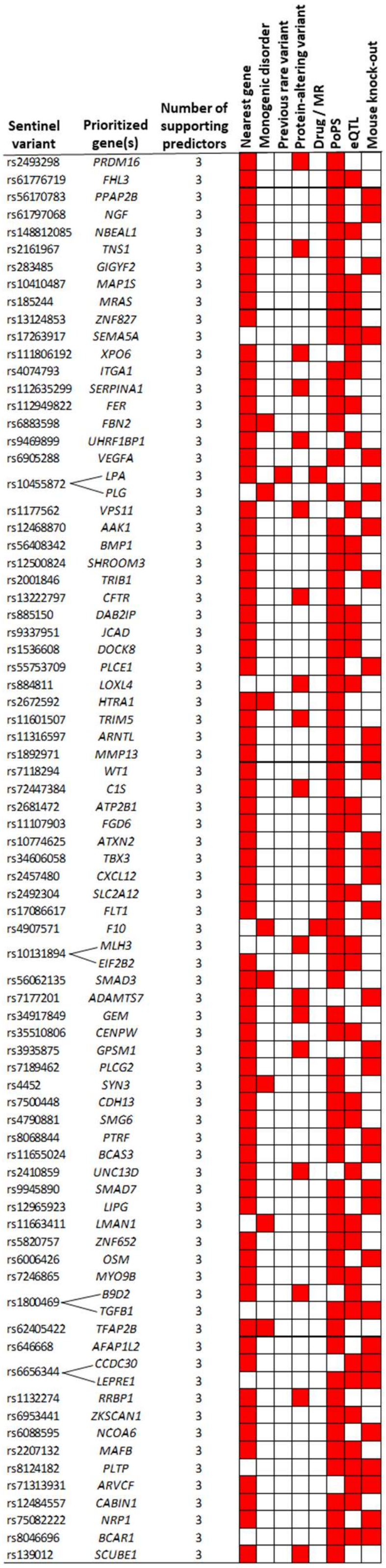

The matrix denotes predictors that supported the mostly likely causal gene (colored red) for each of 125 most likely causal genes with at least three predictors that supported the gene. Genes are ordered by number of agreeing predictors. Lines denote three associations for which two genes were tied for the highest number of agreeing predictors. The sentinel variant for the association with the smallest P-value for CAD is shown for each gene.

Full details of the causal gene prioritization evidence for all 279 genome-wide associations are presented in Supplementary Table 31.
