# Supplementary material for: Discovery and systematic characterization of risk variants and genes for coronary artery disease in over a million participants
Source: Nat Genet. 2022 Dec 6;54(12):1803–15. doi: 10.1038/s41588-022-01233-6 (PMC9729111; doi:10.1038/s41588-022-01233-6)
Supplement: Supplementary file 2 — Reporting Summary [file 41588_2022_1233_MOESM2_ESM.pdf]

nature research

Corresponding author(s): Krishna Aragam & Adam Butterworth

Last updated by author(s): Jul 26, 2022

Reporting Summary

Nature Research wishes to improve the reproducibility of the work that we publish. This form provides structure for consistency and transparency in reporting. For further information on Nature Research policies, see our Editorial Policies and the Editorial Policy Checklist.

Statistics

For all statistical analyses, confirm that the following items are present in the figure legend, table legend, main text, or Methods section.

n/a	Confirmed	
☐	☒	The exact sample size (*n*) for each experimental group/condition, given as a discrete number and unit of measurement
☐	☒	A statement on whether measurements were taken from distinct samples or whether the same sample was measured repeatedly
☐	☒	The statistical test(s) used AND whether they are one- or two-sided<br>*Only common tests should be described solely by name; describe more complex techniques in the Methods section.*
☐	☒	A description of all covariates tested
☐	☒	A description of any assumptions or corrections, such as tests of normality and adjustment for multiple comparisons
☐	☒	A full description of the statistical parameters including central tendency (e.g. means) or other basic estimates (e.g. regression coefficient) AND variation (e.g. standard deviation) or associated estimates of uncertainty (e.g. confidence intervals)
☐	☒	For null hypothesis testing, the test statistic (e.g. *F*, *t*, *r*) with confidence intervals, effect sizes, degrees of freedom and *P* value noted<br>*Give P values as exact values whenever suitable.*
☒	☐	For Bayesian analysis, information on the choice of priors and Markov chain Monte Carlo settings
☒	☐	For hierarchical and complex designs, identification of the appropriate level for tests and full reporting of outcomes
☐	☒	Estimates of effect sizes (e.g. Cohen's *d*, Pearson's *r*), indicating how they were calculated

Our web collection on statistics for biologists contains articles on many of the points above.

Software and code

Policy information about availability of computer code

Data collection

Data were all collected by the individual studies prior to this project.

Data analysis

Software used for data analysis, as described in the Online Methods, includes:

PLINK v2.0: www.cog-genomics.org/plink/2.0/
SNPTEST v2.5.2: https://mathgen.stats.ox.ac.uk/genetics_software/snptest/snptest.html
METAL v1: https://genome.sph.umich.edu/wiki/METAL
GCTA v1.93: https://cnsgenomics.com/software/gcta/
Ensembl Variant Effect Predictor v102: https://www.ensembl.org/info/docs/tools/vep/index.html
SAIGE and SAIGE-GENE v0.44: https://github.com/weizhouUMICH/SAIGE
GWAMA v2.2: https://genomics.ut.ee/en/tools/gwama
qvalue R package: https://www.bioconductor.org/packages/release/bioc/html/qvalue.html
INDI-V: http://cnsgenomics.com/shiny/INDI-V
LDpred v.1.0.11: https://bitbucket.org/bjarni_vilhjalmsson/ldpred/
EIGENSOFT v7.2.1: https://www.hsph.harvard.edu/alkes-price/software/
FGWAS v0.3.6: https://github.com/joepickrell/fgwas
PoPS v0.2: https://github.com/FinucaneLab/pops
Manhattan++: https://rdrr.io/cran/manhplot/

Other analyses were conducted using standard statistical software:
R: https://www.r-project.org/
STATA: https://www.stata.com/

Custom scripts are available on reasonable request to the corresponding authors.

For manuscripts utilizing custom algorithms or software that are central to the research but not yet described in published literature, software must be made available to editors and reviewers. We strongly encourage code deposition in a community repository (e.g. GitHub). See the Nature Research guidelines for submitting code & software for further information.

Data

Policy information about availability of data

All manuscripts must include a data availability statement. This statement should provide the following information, where applicable:

- Accession codes, unique identifiers, or web links for publicly available datasets
- A list of figures that have associated raw data
- A description of any restrictions on data availability

Summary statistics are available upon publication through the CARDIoGRAMplusC4D website (http://www.cardiogramplusc4d.org/) and the NHGRI-EBI GWAS Catalog (https://www.ebi.ac.uk/gwas/) and polygenic risk score weights are available in the Polygenic Score (PGS) Catalog (https://www.pgscatalog.org/). Interactive searchable Manhattan plots and a locus-specific epigenome annotation browser for functionally enriched loci are available at: https://procardis.shinyapps.io/cadgen/. An interactive searchable browser detailing the locus-specific evidence prioritizing causal variants, genes and pathways is available at the Common Metabolic Diseases Knowledge Portal (beta version available at: https://hugeamp.org/method.html?trait=cad&dataset=cardiogram).

Other datasets used in this study include the NCBI's ClinVar database (https://www.ncbi.nlm.nih.gov/clinvar/) on 26th June 2020, a 1000 Genomes European ancestry LD file comprising ~1.2 million variants available at https://alkesgroup.broadinstitute.org/LDSCORE/, the GTEx Consortium v7 data release (https://www.gtexportal.org/home/datasets), the Ensembl database (www.ensembl.org). the International Mouse Phenotyping Consortium, data release 10.1 (www.mousephenotype.org), and the Mouse Genome Informatics database, data from July 2019 (www.informatics.jax.org).

Field-specific reporting

Please select the one below that is the best fit for your research. If you are not sure, read the appropriate sections before making your selection.

☒ Life sciences ☐ Behavioural & social sciences ☐ Ecological, evolutionary & environmental sciences

For a reference copy of the document with all sections, see nature.com/documents/nr-reporting-summary-flat.pdf

Life sciences study design

All studies must disclose on these points even when the disclosure is negative.

Sample size

Details of the ten de novo studies used for the discovery GWAS, including the source of participants, case and control definitions and basic participant characteristics are presented in Supplementary Table 1. We used summary statistics from either (a) a previous 1000 Genomes-imputed GWAS meta-analysis of up to 60,801 CAD cases and 123,504 CAD-free controls;[7] (b) a meta-analysis of ~79,000 variants in up to 88,192 CAD cases and 162,544 controls, predominantly based on the Illumina CardioMetabochip array;[2] or (c) a meta-analysis ~184,000 variants in up to 42,335 CAD cases and 78,240 controls based on the Illumina Exome array.[10, 16] We then combined these results with summary statistics from the CARDIoGRAMplusC4D Consortium, achieving a total sample of 181,522 CAD cases among 1,165,690 study participants.
For trans-ethnic comparison, we used summary statistics from a recent GWAS of 29,319 CAD cases and 183,134 controls from the Biobank Japan.
For the polygenic risk score analyses, we used independent datasets from the Malmo Diet and Cancer Study for primary prevention (n=28,556, including 4,122 incident cases) and the FOURIER trial for secondary prevention (n=7,135, including 673 recurrent events).

No a priori power calculations were conducted to determine the necessary sample size for this study, but as this is the largest sample size for a GWAS of coronary artery disease to date, it has the greatest power to detect associations.

Data exclusions

Details of study-specific variant and sample exclusions can be found in Supplementary Table 1.

Replication

To maximise the power for discovery of genetic associations, we did not split the CAD GWAS dataset into discovery and replication datasets. However, we did assess the associations in an independent dataset of East Asian ancestry (Biobank Japan), which showed strong correlation of effect sizes. We did not conduct a formal replication in this population.
We did, however, try to replicate 216 previously reported CAD loci in our study. We successfully replicated 188 of these signals, with non-replicating signals being primarily due to different ancestries.
For the polygenic score analyses, we split the Malmo Diet and Cancer study into testing and training datasets of non-overlapping participants.

Randomization

As this is an observational genetic association study with no intervention, randomization is not relevant here.

Blinding

As this is an observational genetic association study with no intervention, randomization is not relevant here.

Reporting for specific materials, systems and methods

We require information from authors about some types of materials, experimental systems and methods used in many studies. Here, indicate whether each material, system or method listed is relevant to your study. If you are not sure if a list item applies to your research, read the appropriate section before selecting a response.

Materials & experimental systems

n/a	Involved in the study
☒	☐ Antibodies
☐	☒ Eukaryotic cell lines
☒	☐ Palaeontology and archaeology
☒	☐ Animals and other organisms
☐	☒ Human research participants
☒	☐ Clinical data
☒	☐ Dual use research of concern

Methods

n/a	Involved in the study
☒	☐ ChIP-seq
☒	☐ Flow cytometry
☒	☐ MRI-based neuroimaging

Eukaryotic cell lines

Policy information about cell lines

Cell line source(s)	Human coronary artery vascular smooth muscle cells (Lonza CC-2583; culture media CC-31182); immortalized human aortic endothelial cells (ATCC CRL-4052; culture media Lifeline Cell Technology LL-0003); THP-1 monocyte cells (ATCC TIB-202; culture media RPMI ATCC 30-2001, 10% FBS Sigma 12306C-500ML).
Authentication	None of the cell lines were authenticated.
Mycoplasma contamination	None of the cell lines were tested for mycoplasma contamination.
Commonly misidentified lines (See ICLAC register)	We did not use any commonly misidentified cell lines.

Human research participants

Policy information about studies involving human research participants

Population characteristics	Participants in our de novo CAD GWAS meta-analysis were largely (>95%) of European ancestry (predominantly from Europe or the US) and 46% were female. Study-specific details can be found in Supplementary Table 1.
Recruitment	Our analysis includes many studies, including case-control studies, general population biobanks, hospital-based biobanks and clinical trials.
Ethics oversight	All studies had appropriate ethical approval from relevant ethics committees and all participants gave informed consent for their participation.

Note that full information on the approval of the study protocol must also be provided in the manuscript.
