# Supplementary figures and images for: Discovery and systematic characterization of risk variants and genes for coronary artery disease in over a million participants
Source: Nat Genet. 2022 Dec 6;54(12):1803–15. doi: 10.1038/s41588-022-01233-6 (PMC9729111; doi:10.1038/s41588-022-01233-6)

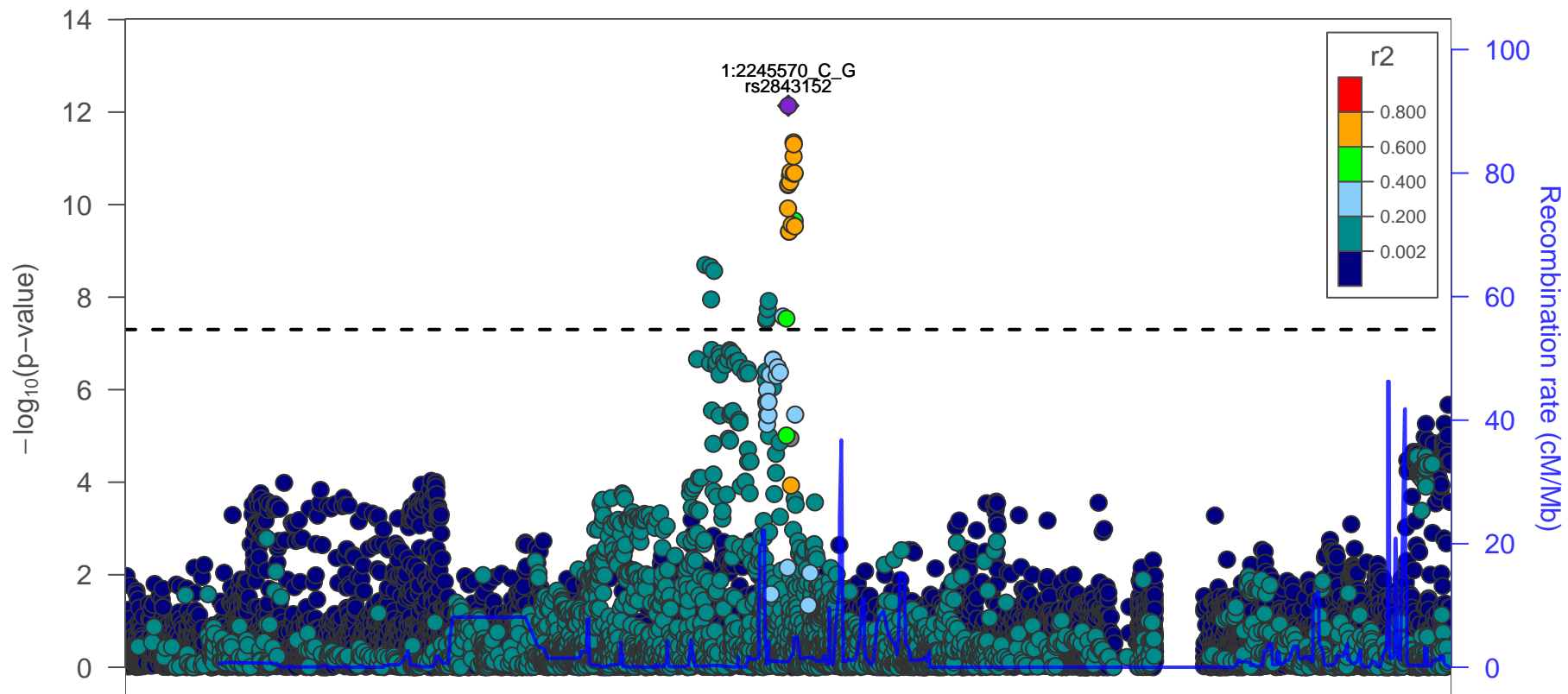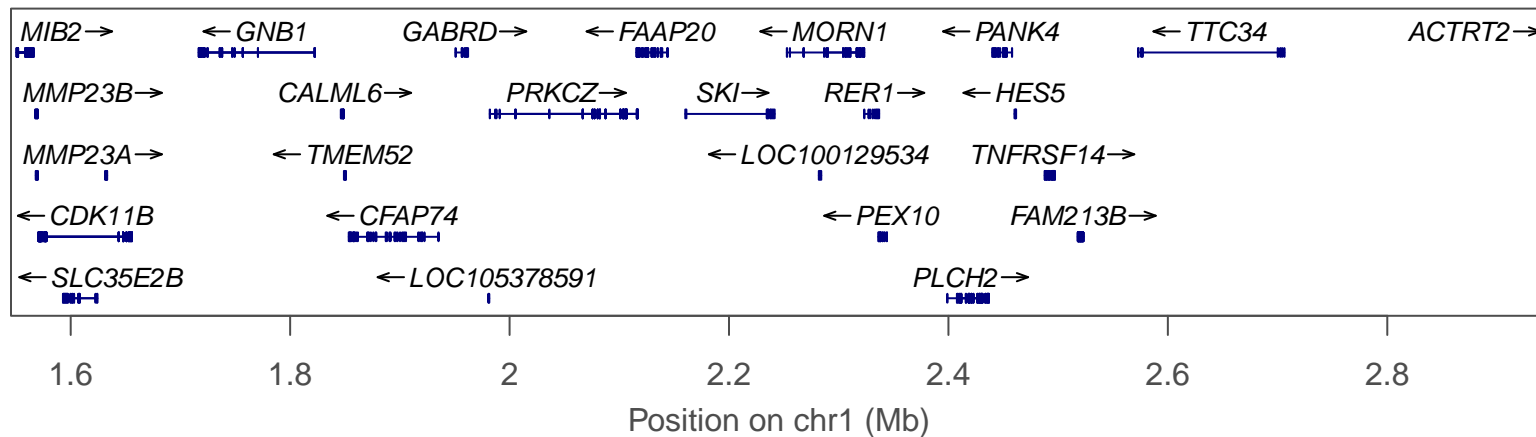

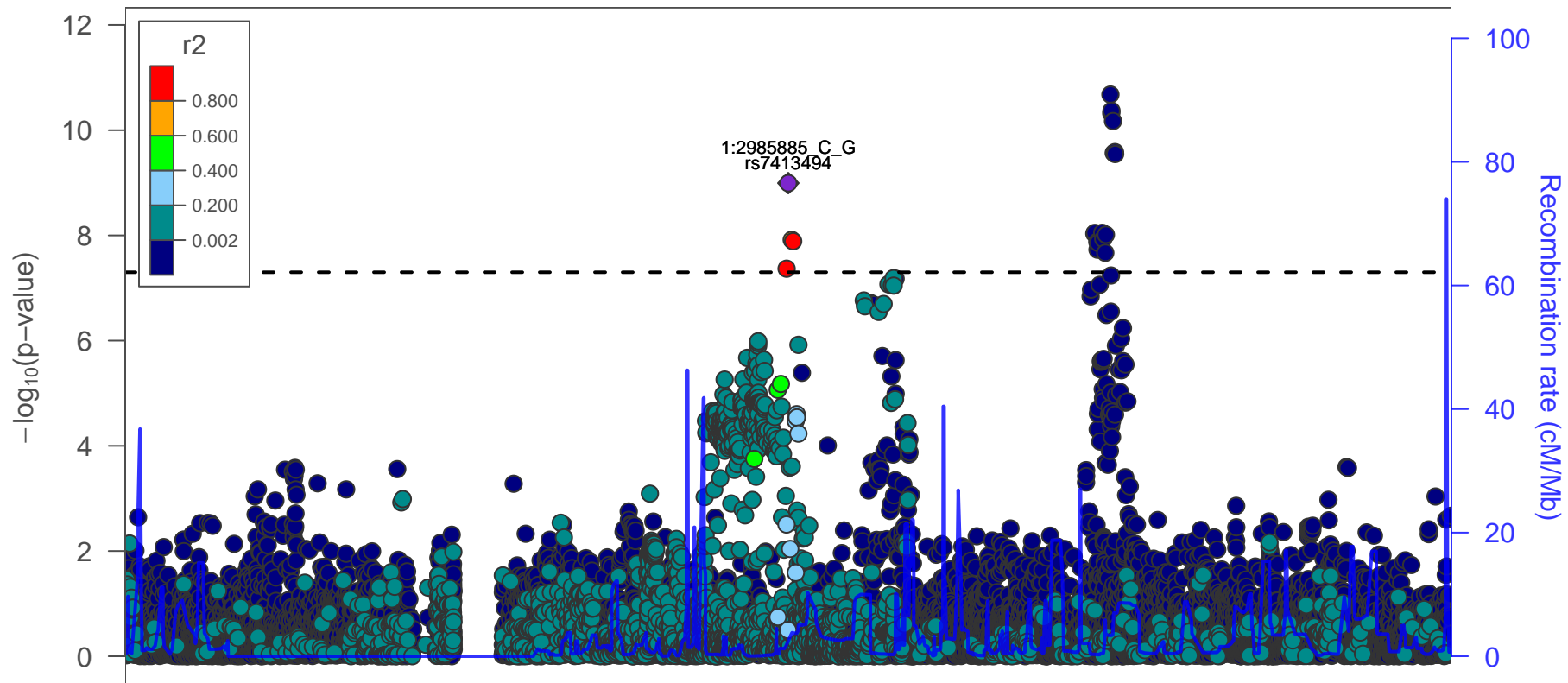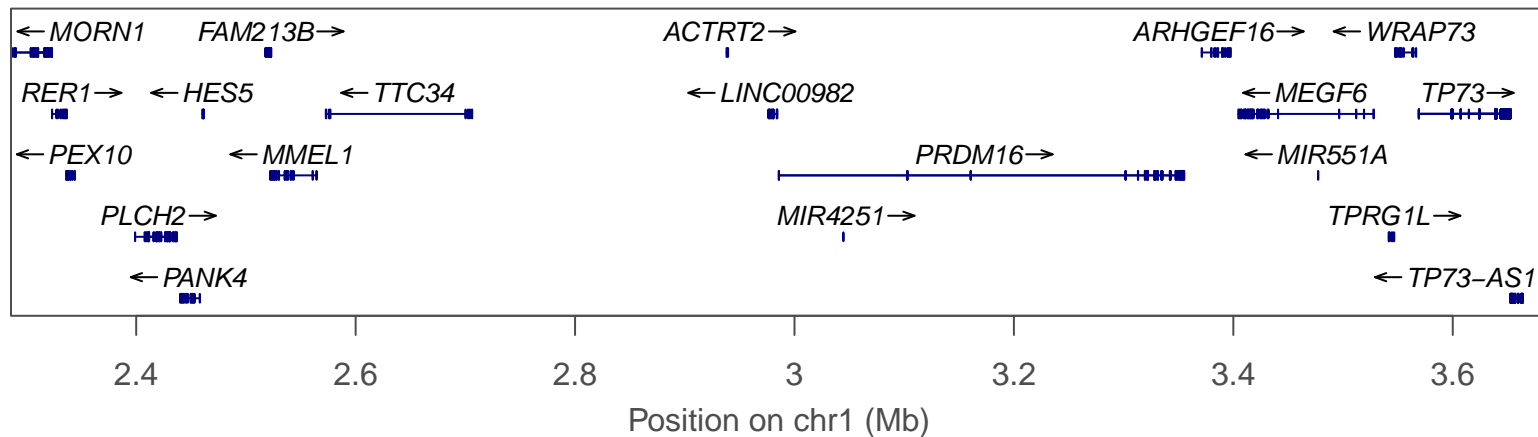

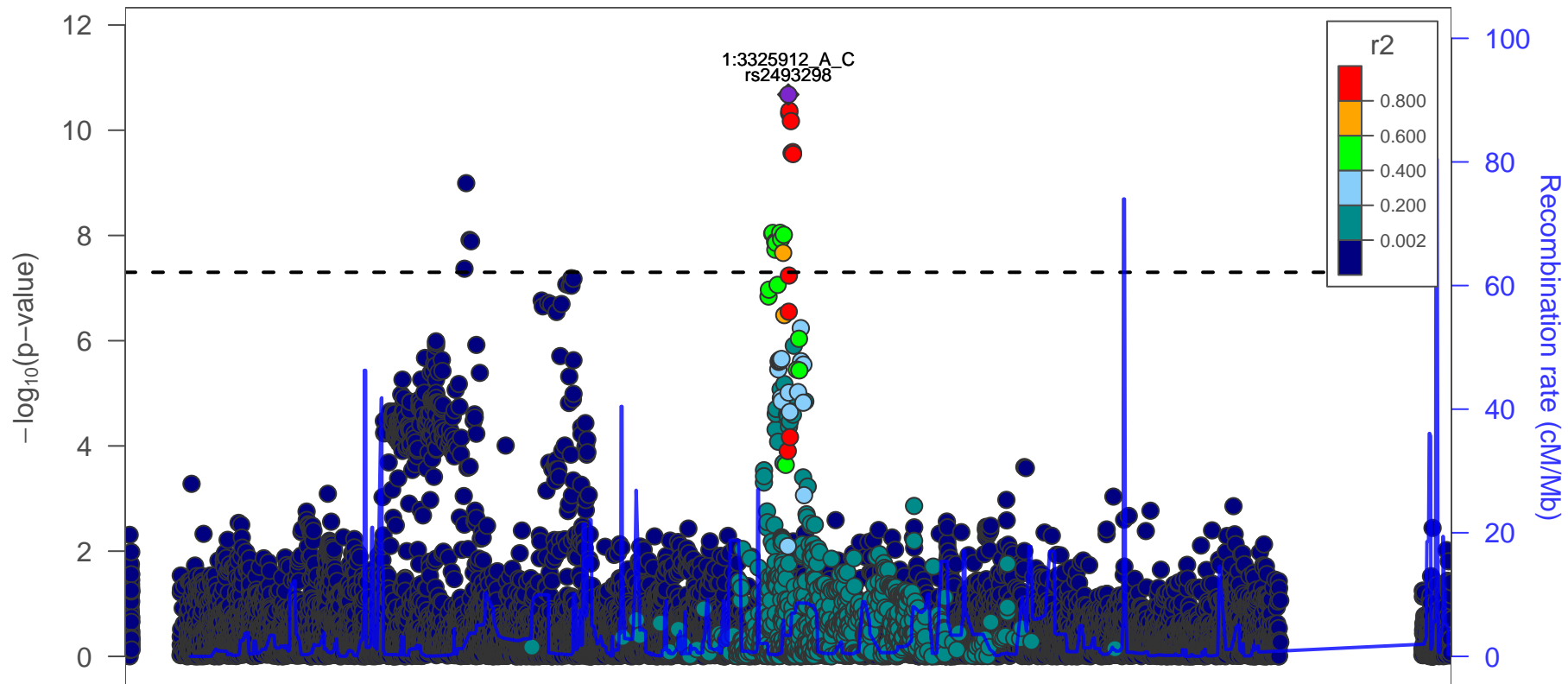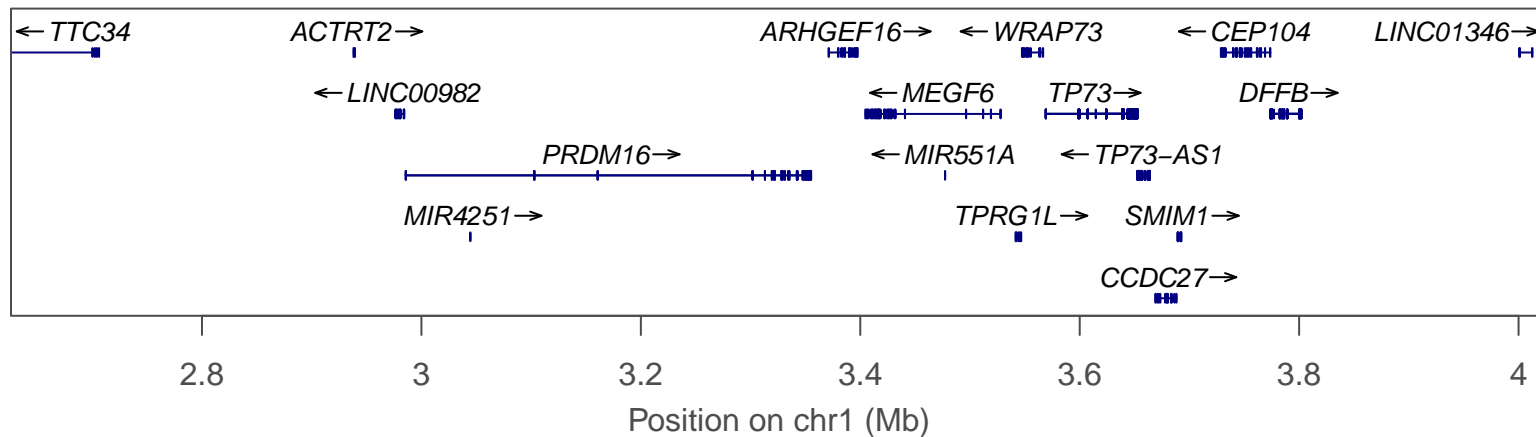

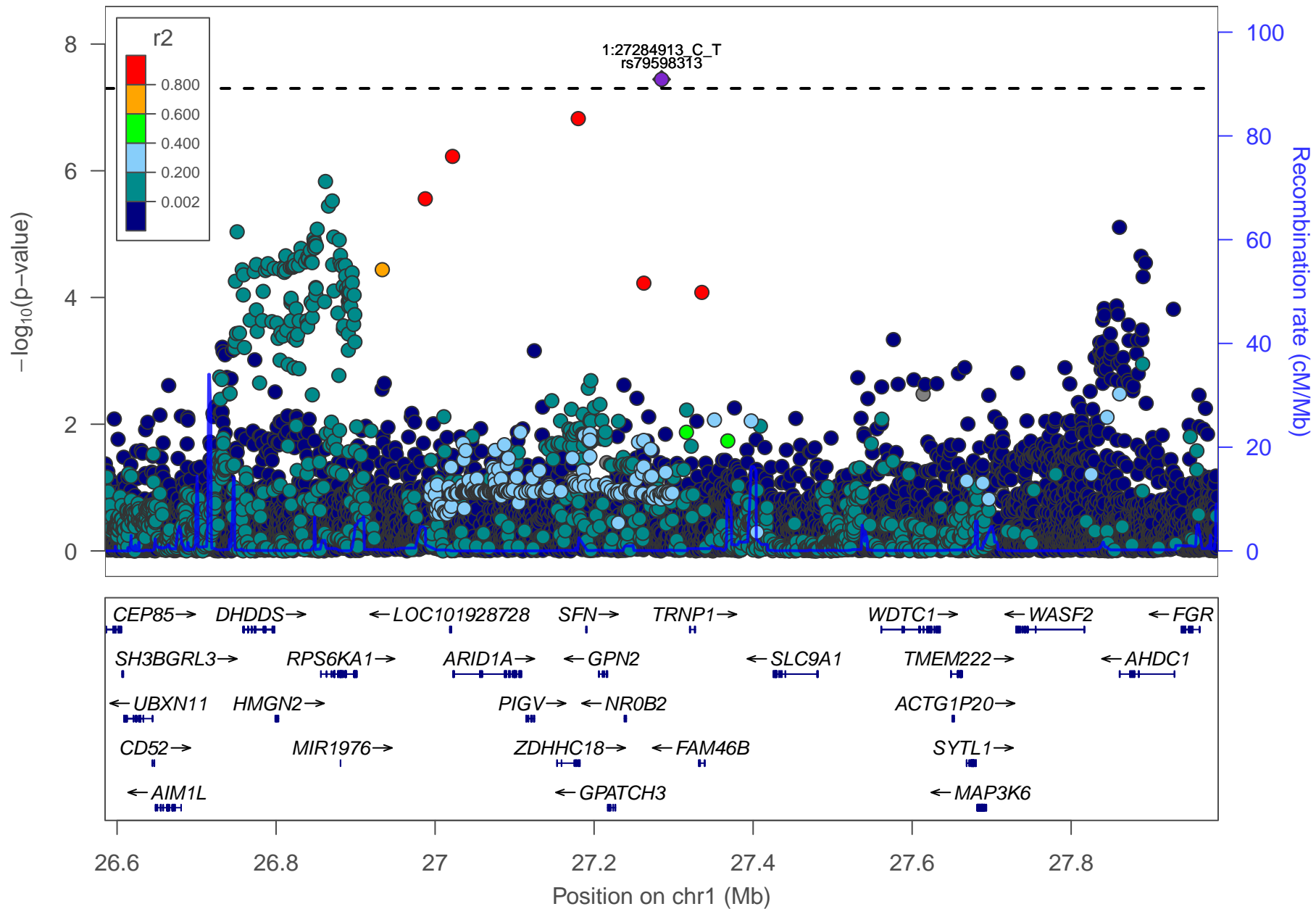

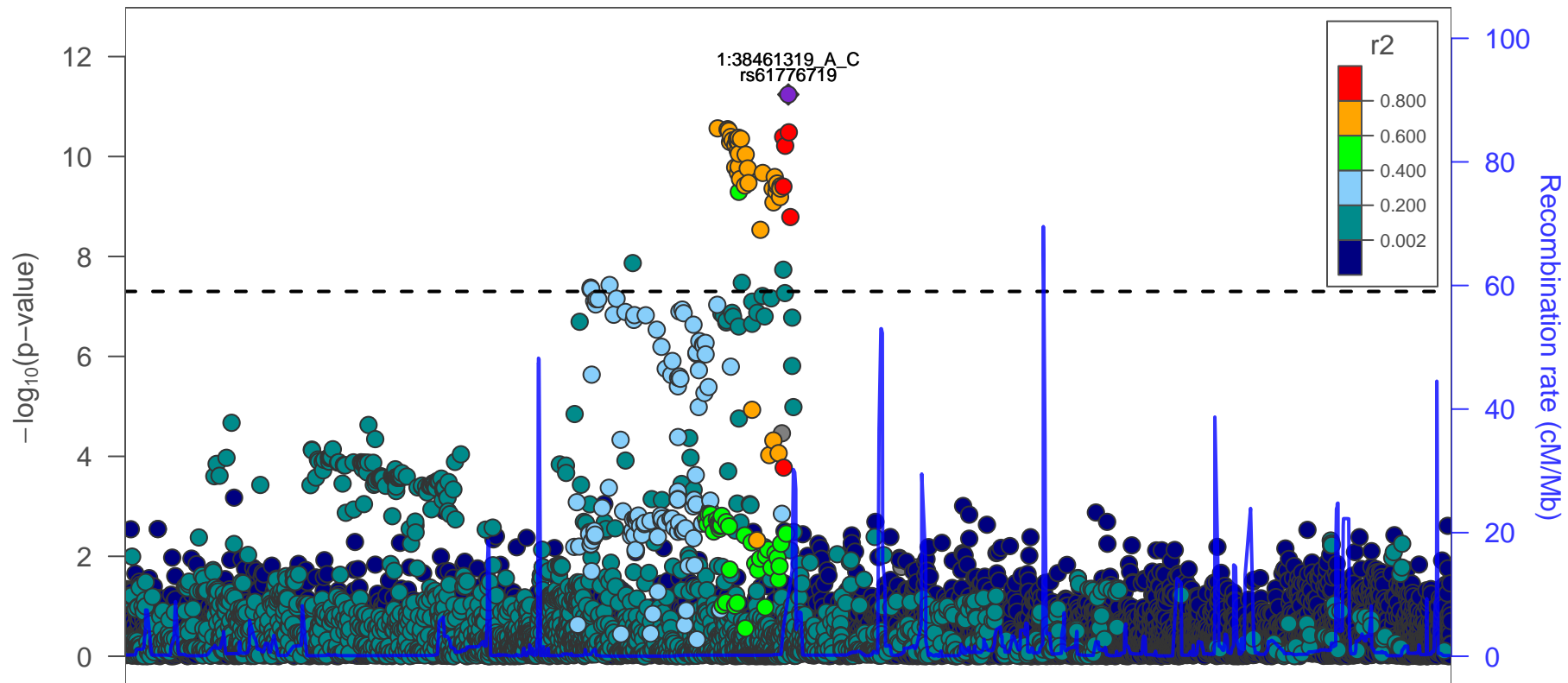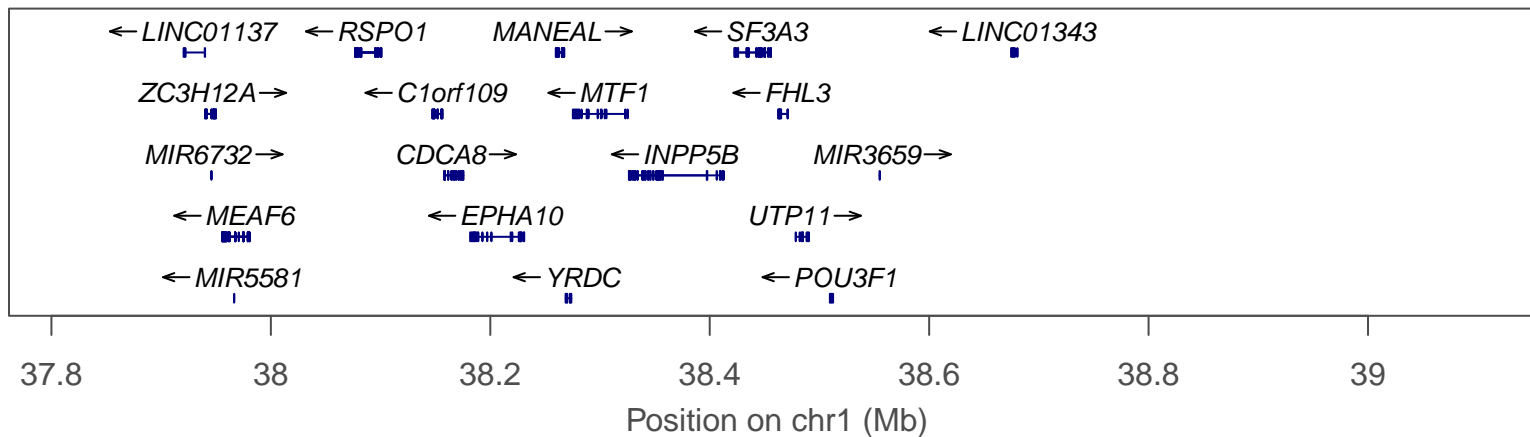

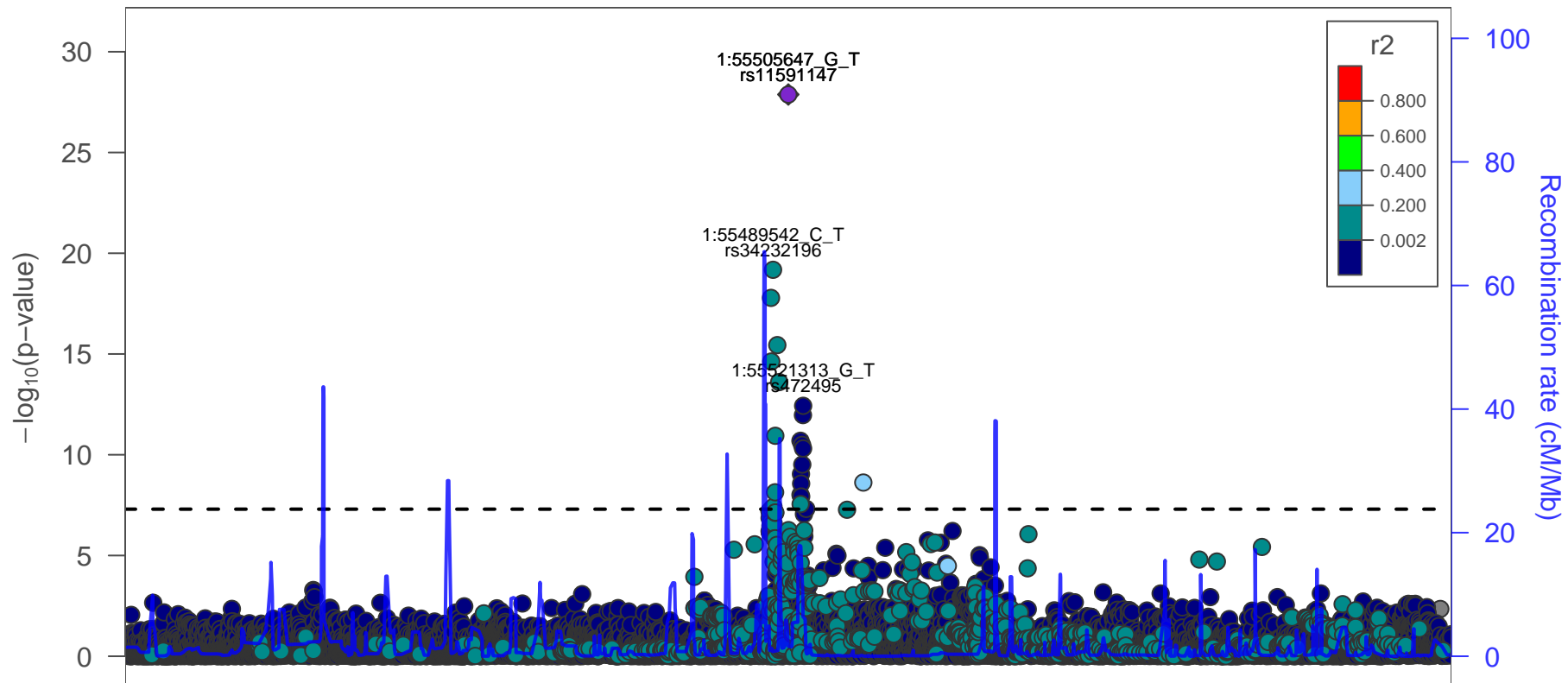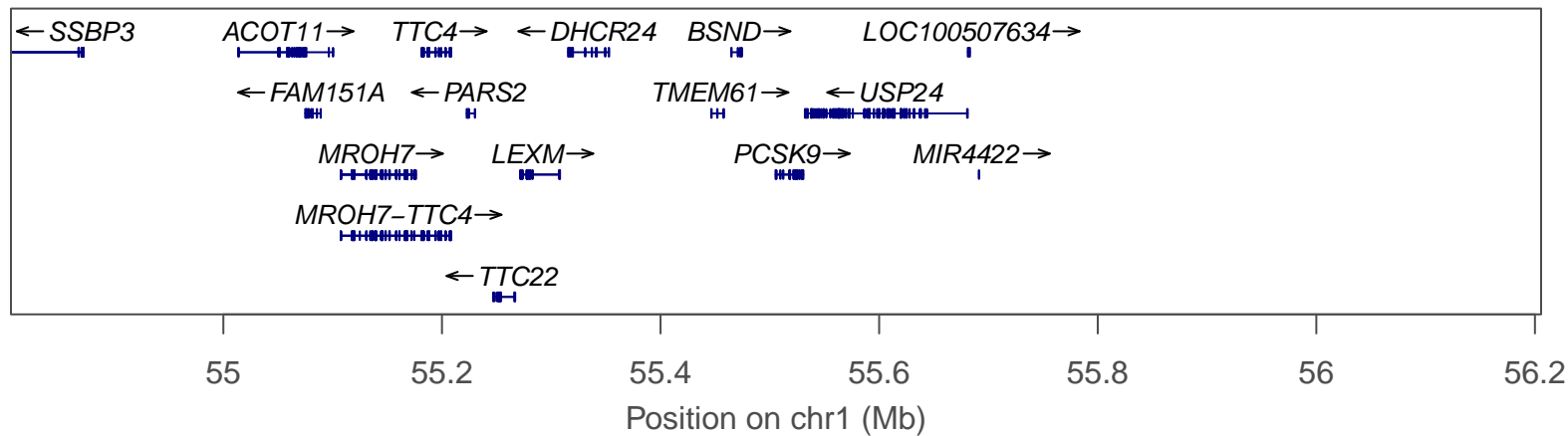

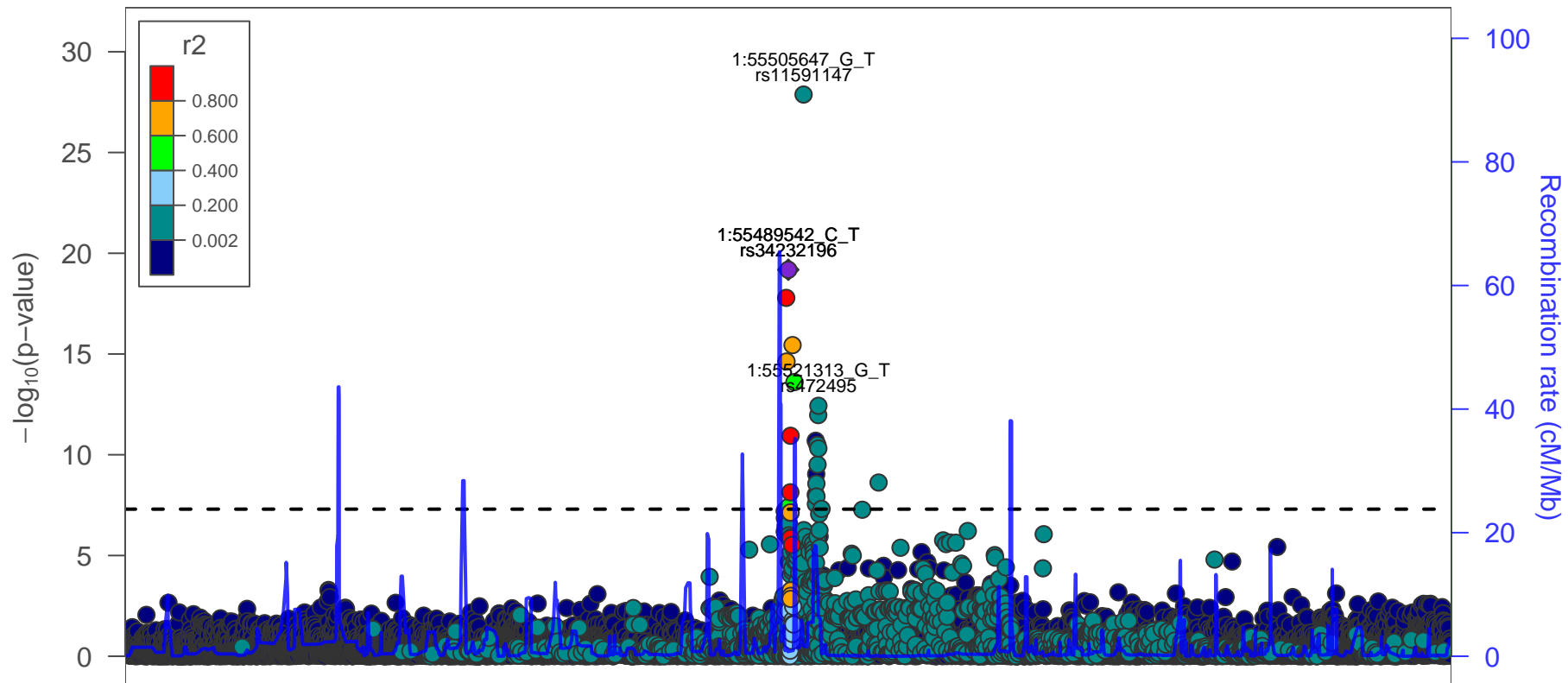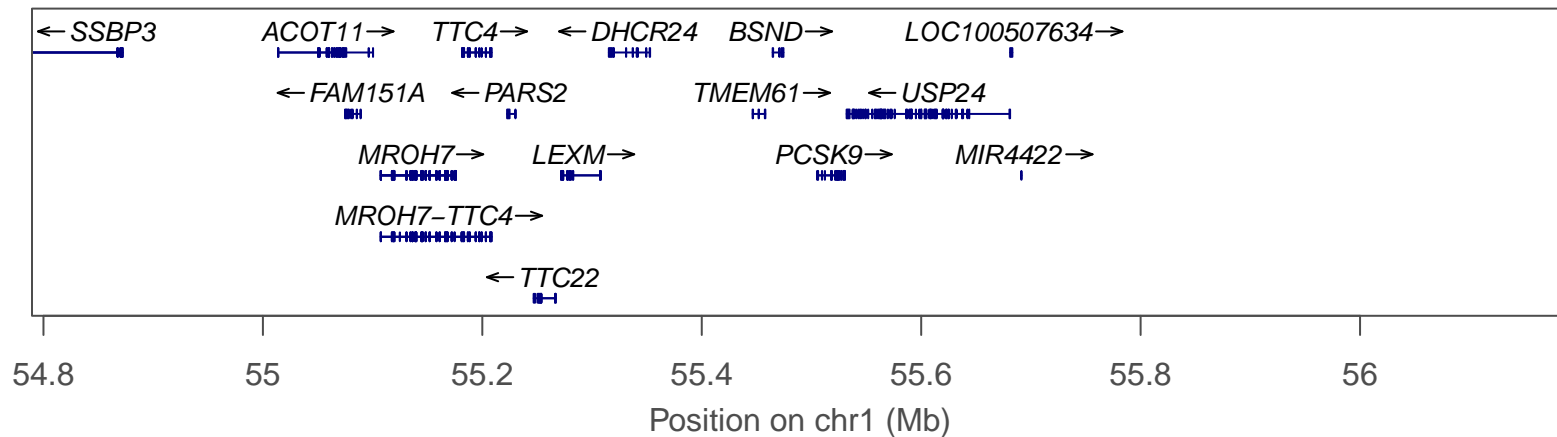

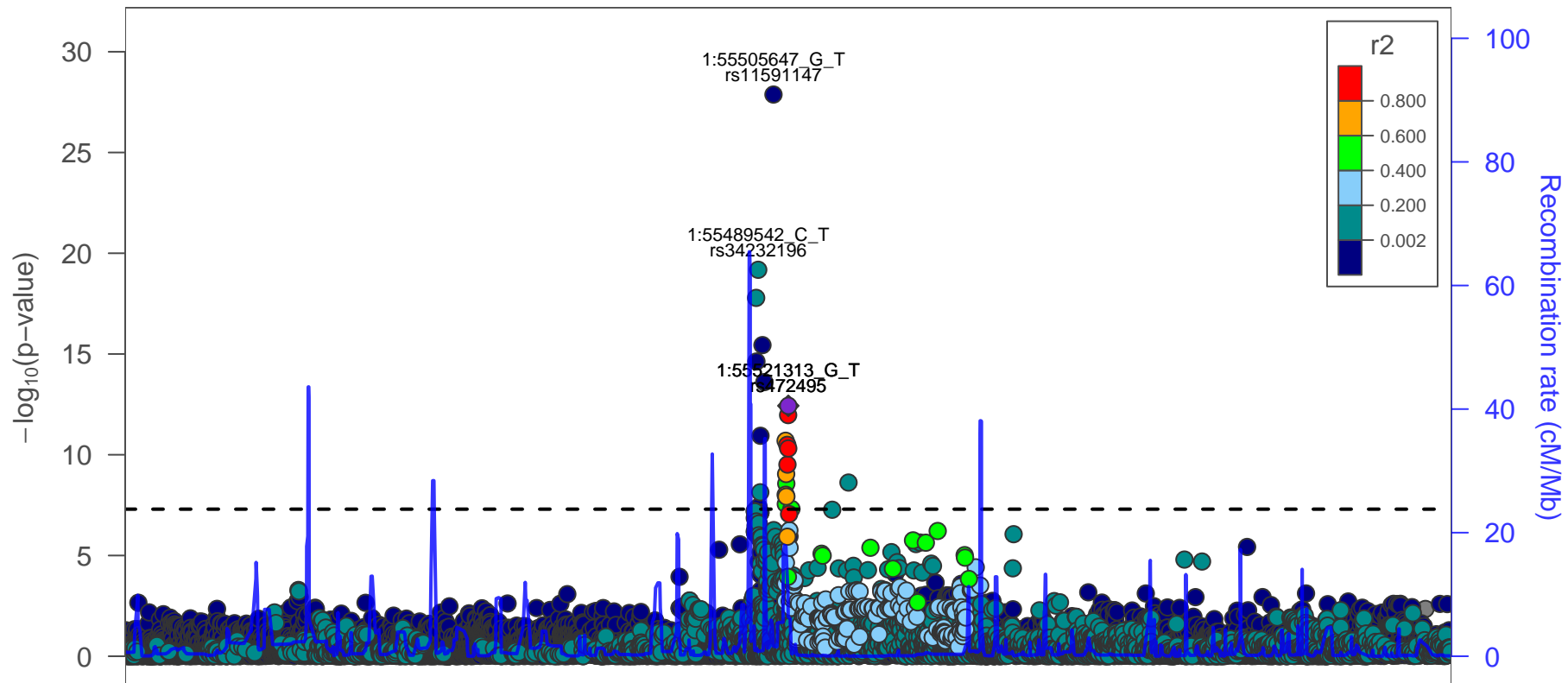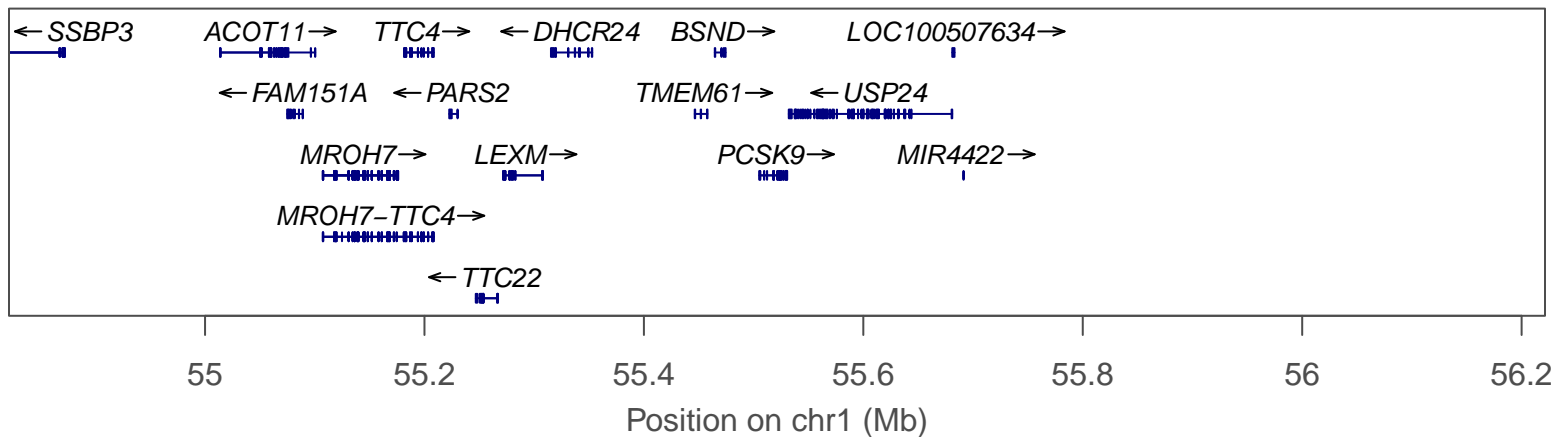

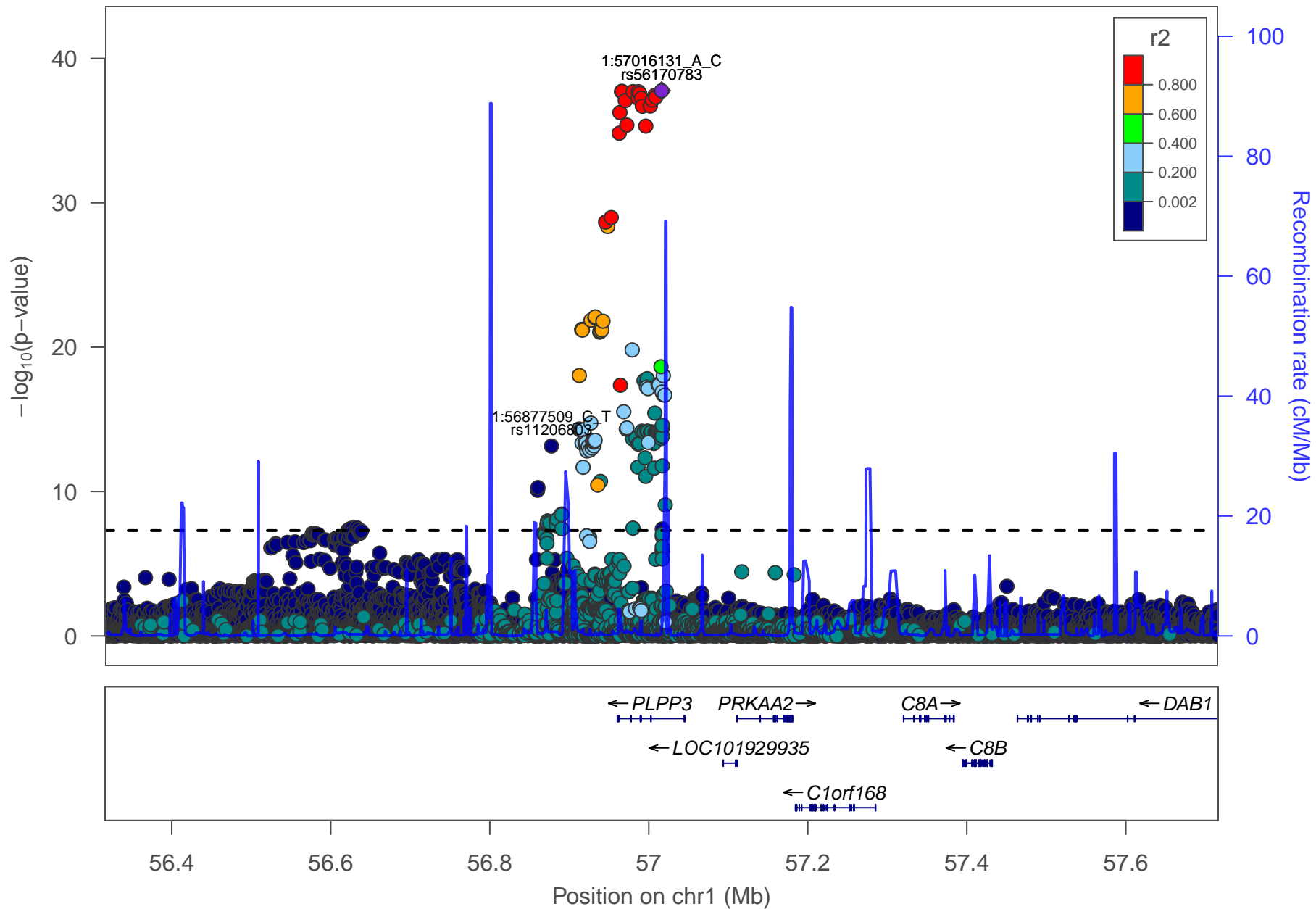

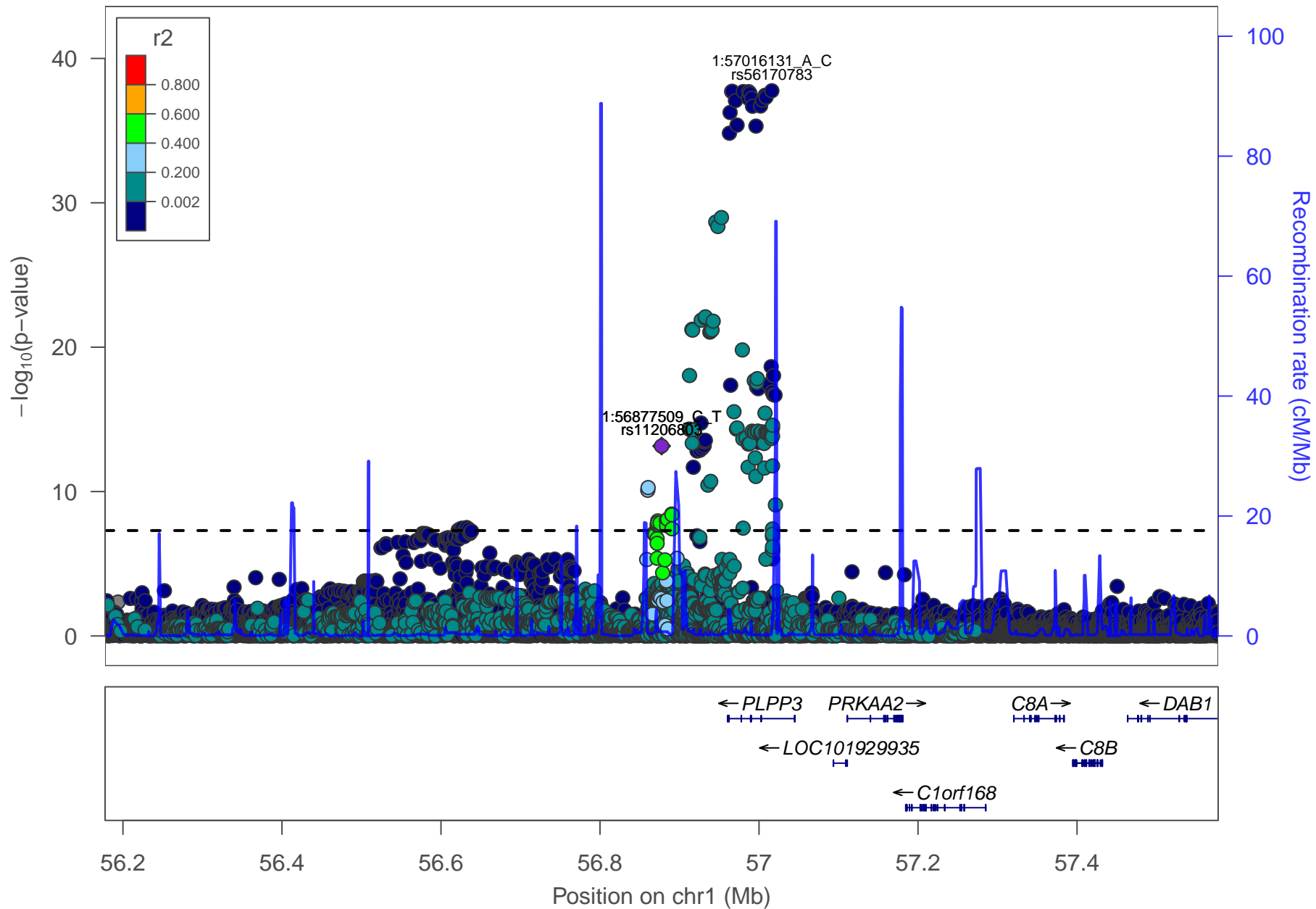

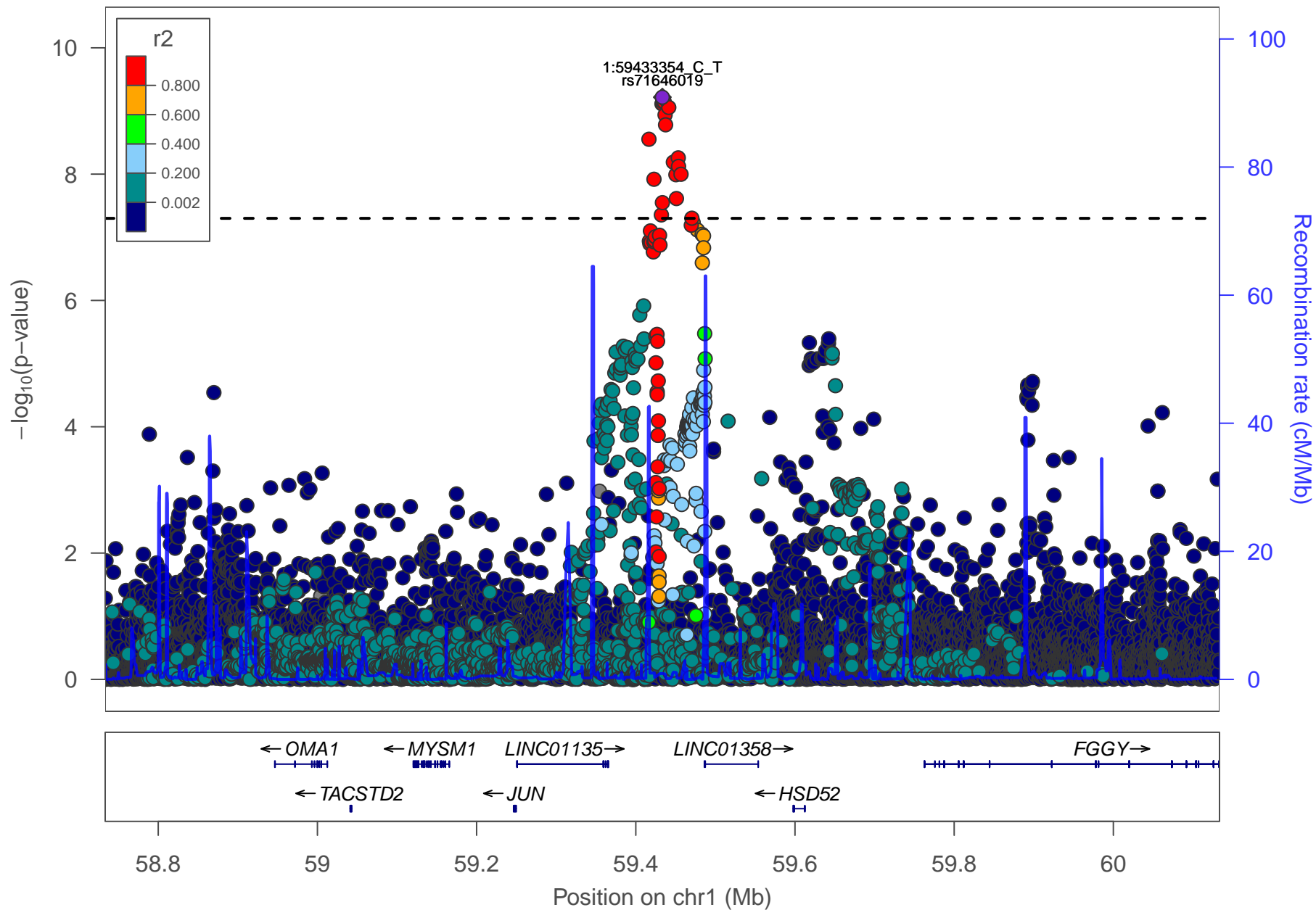

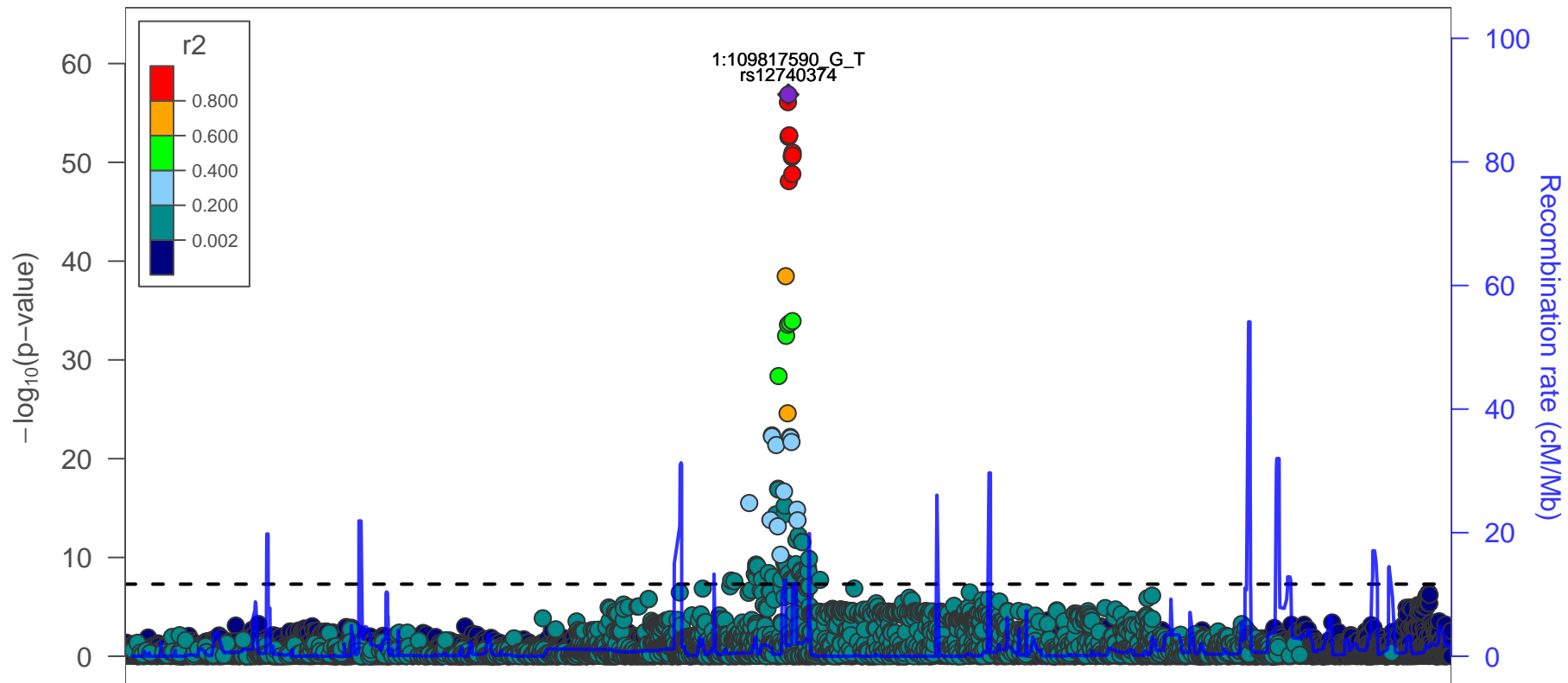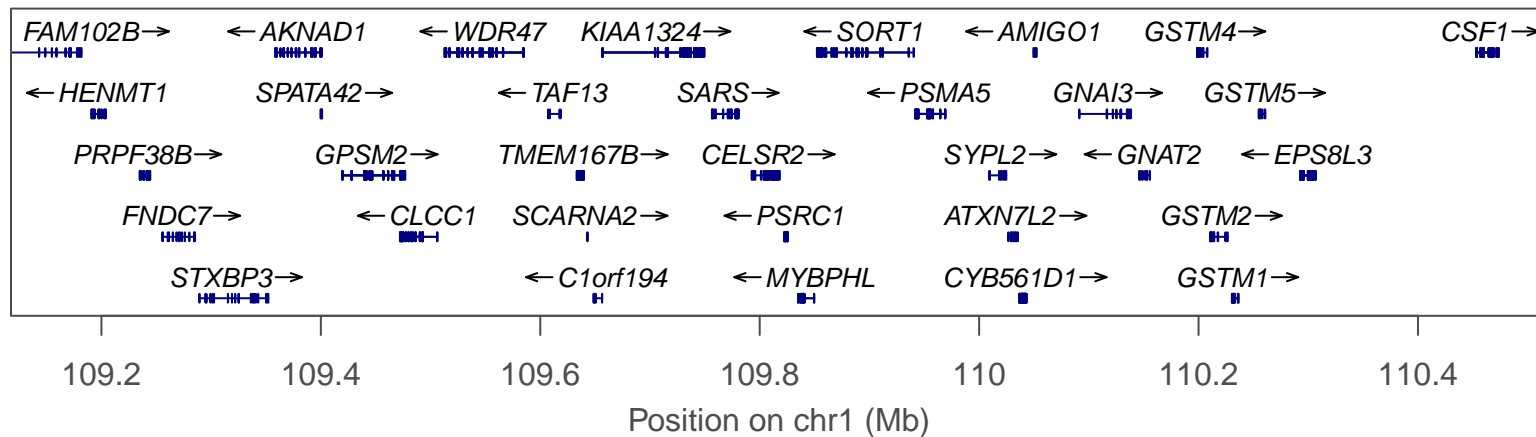

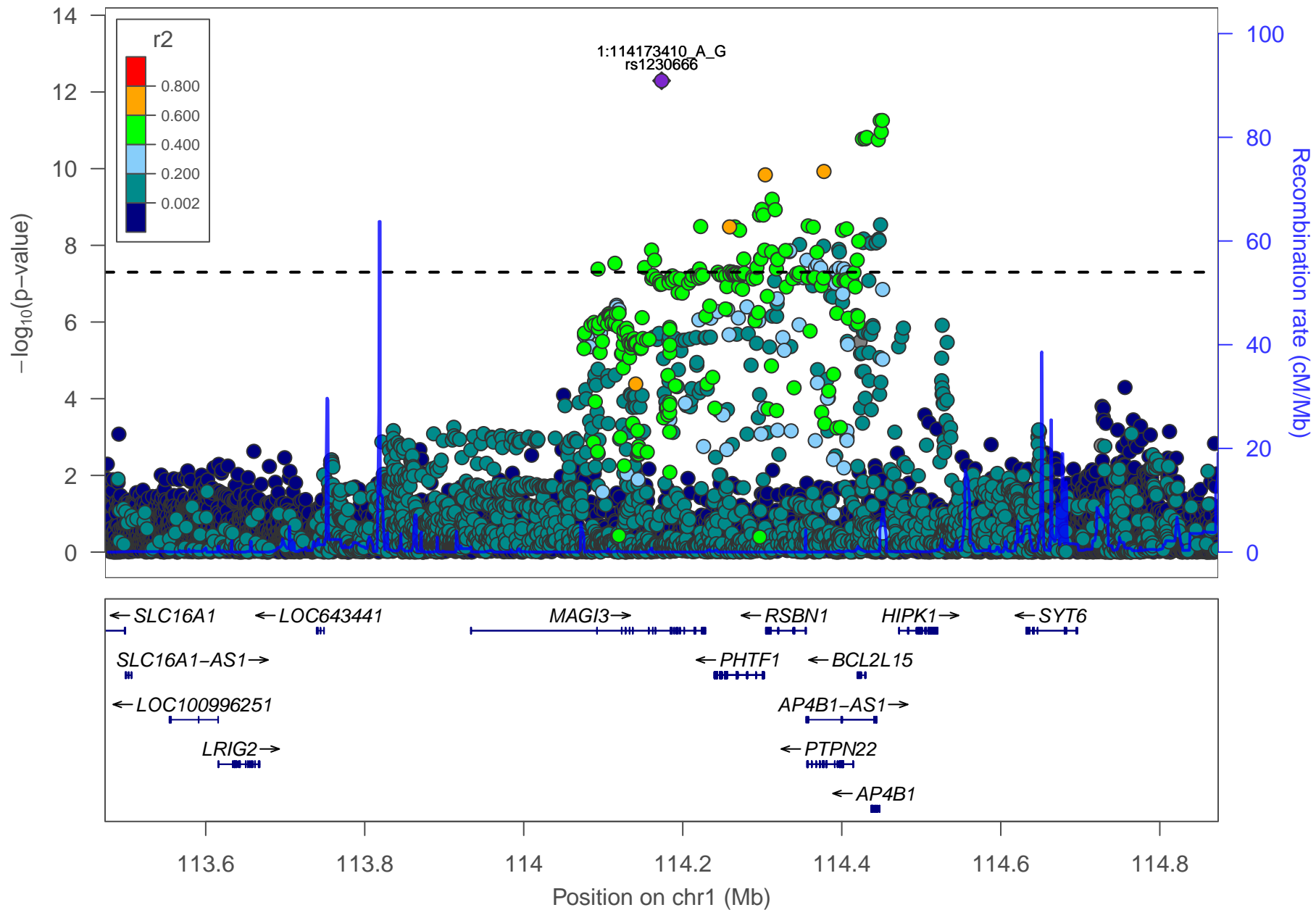

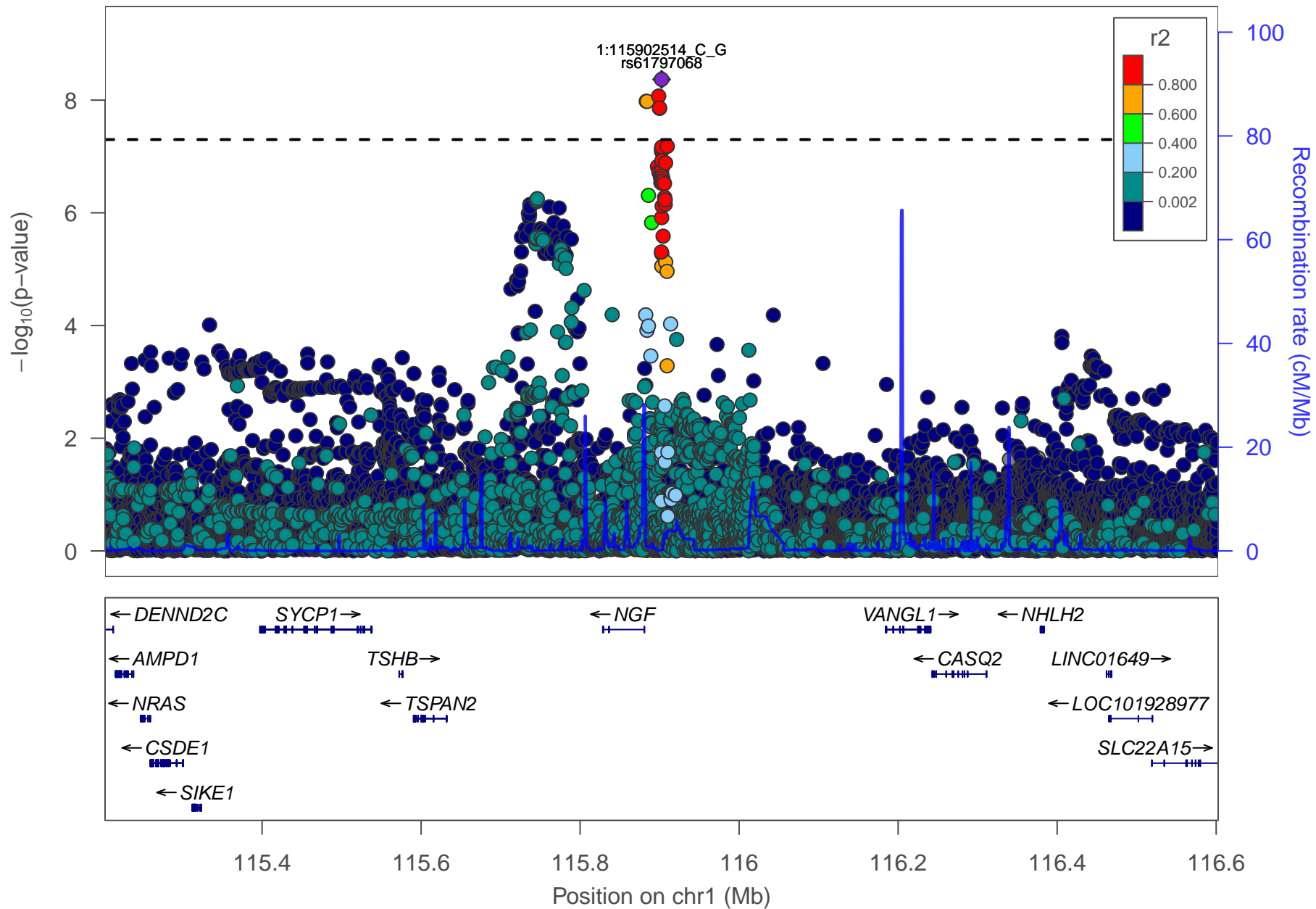

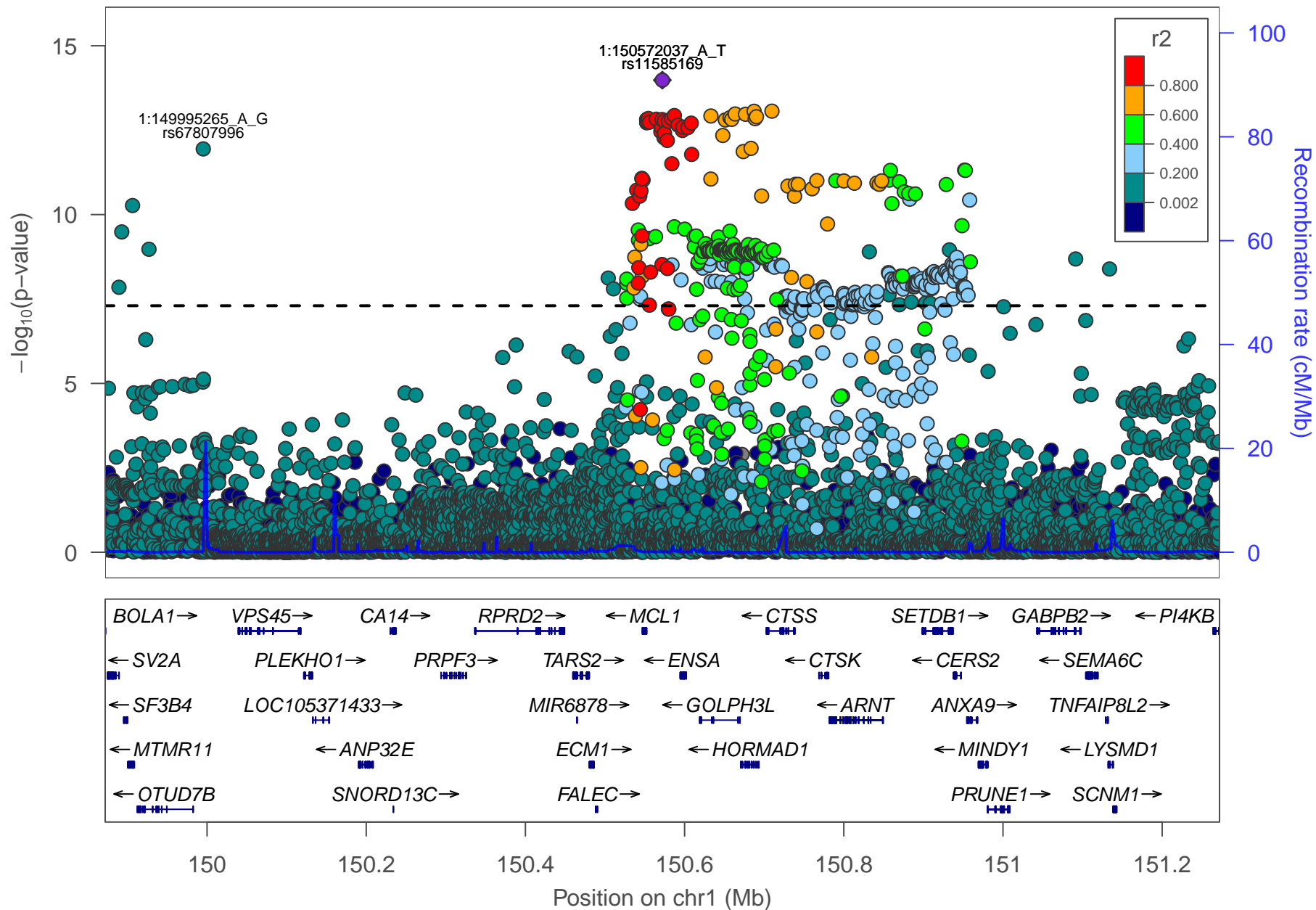

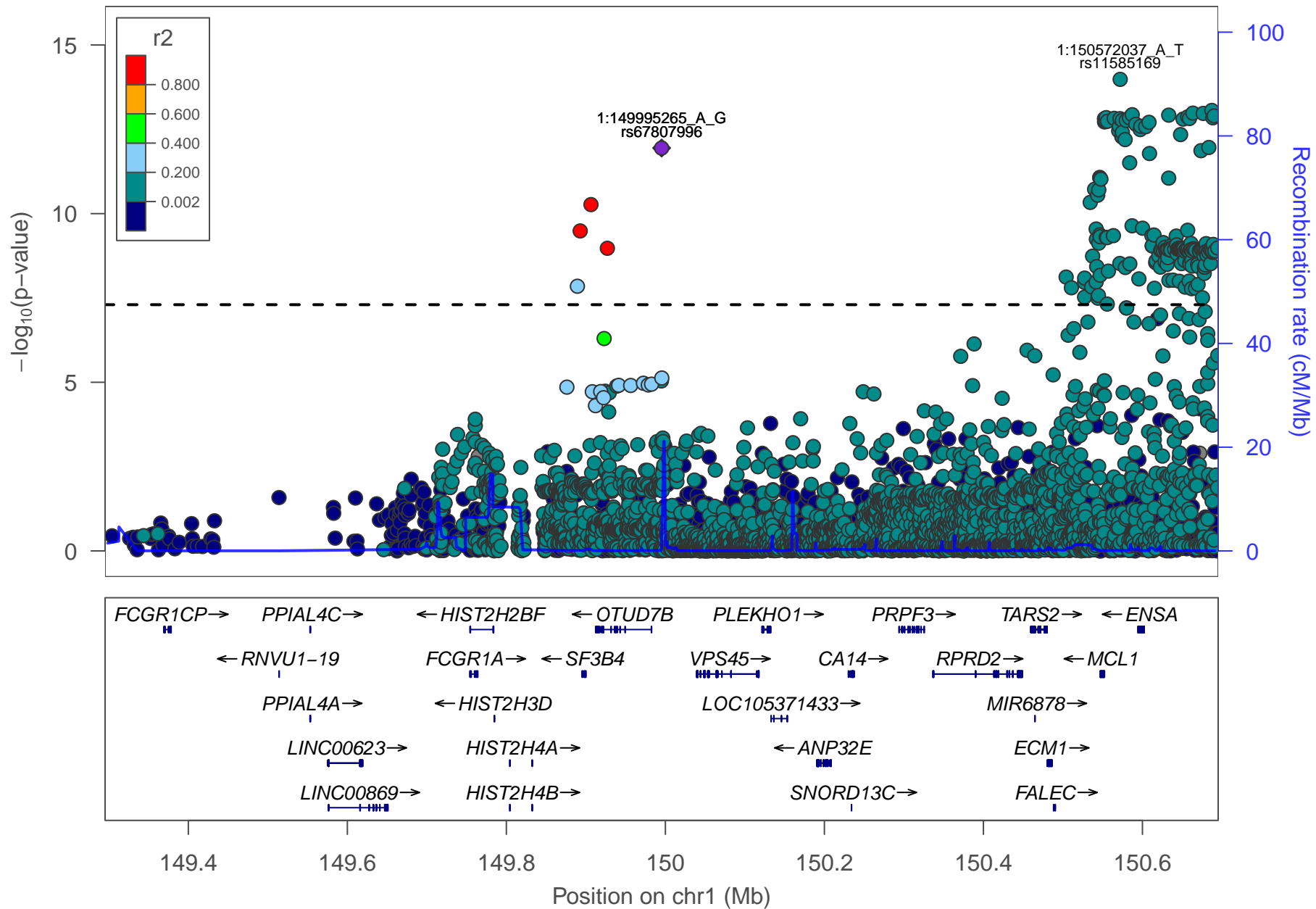

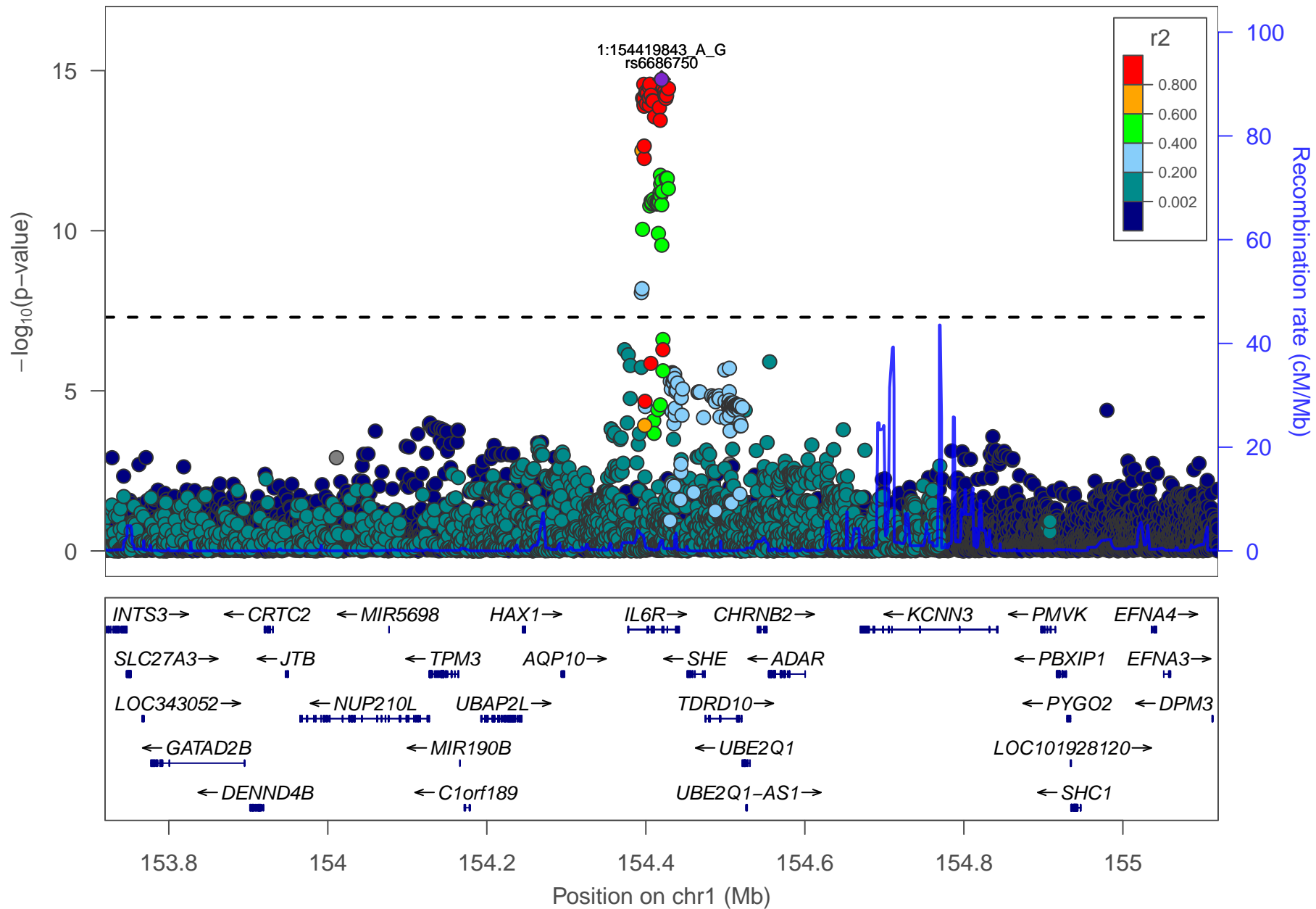

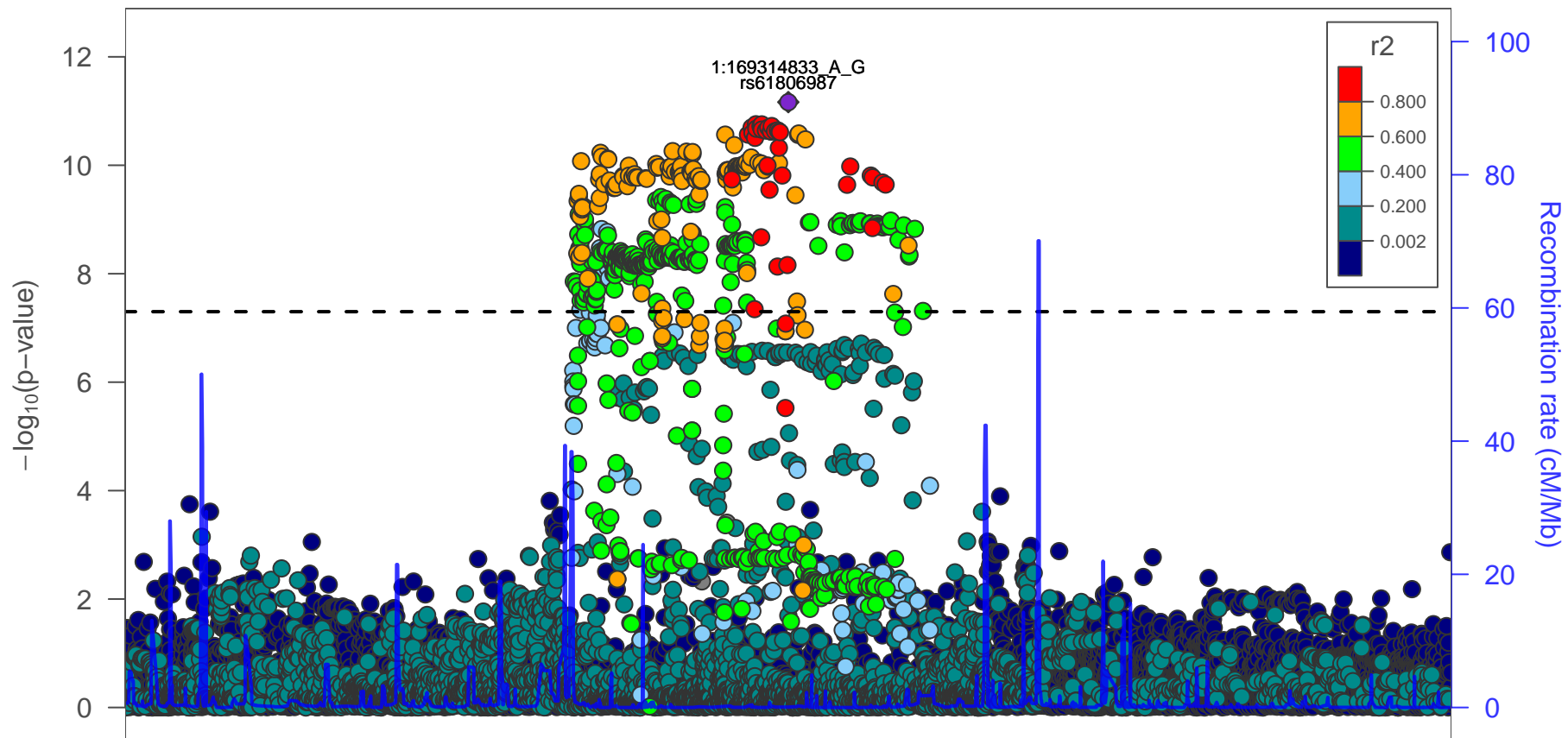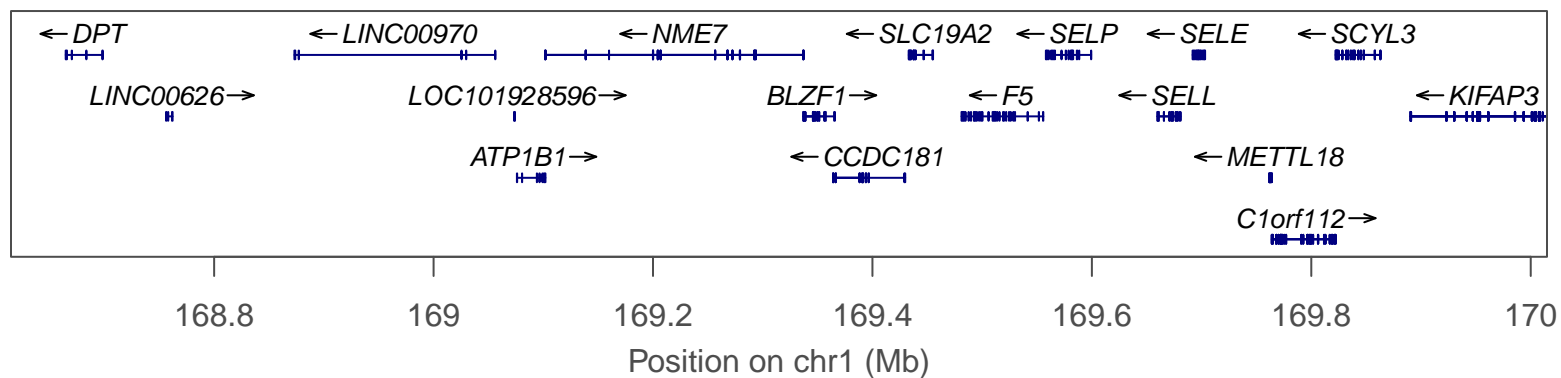

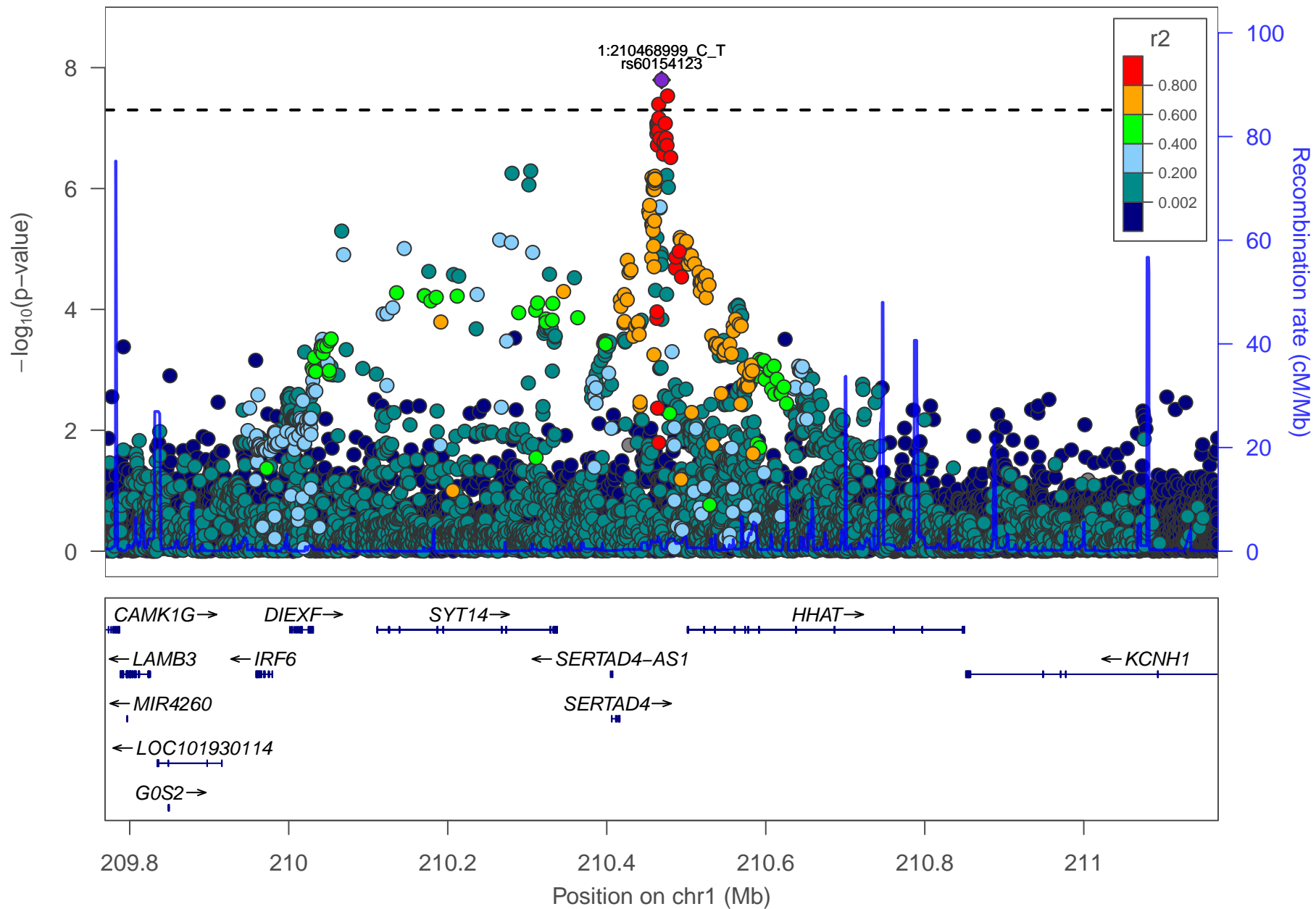

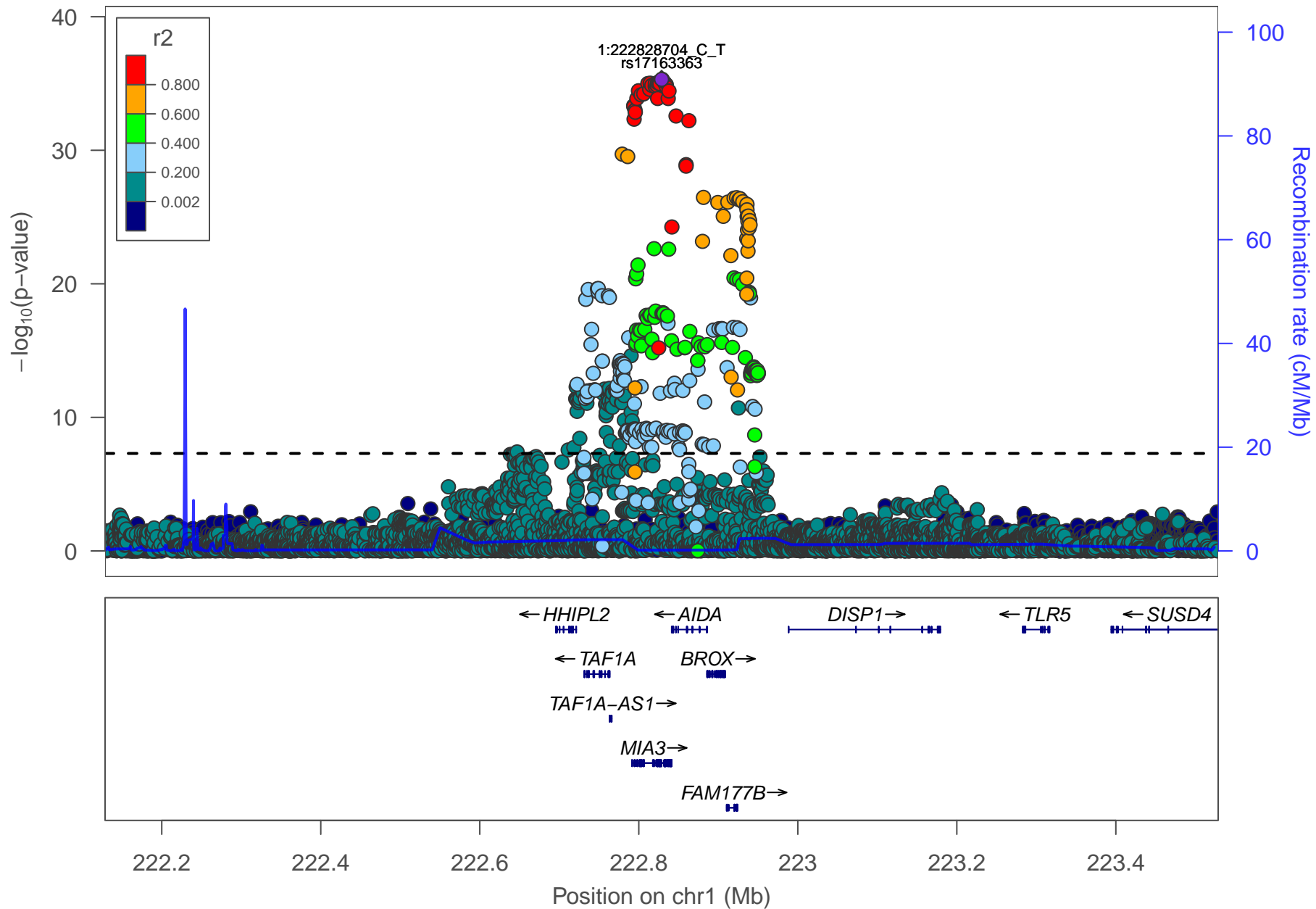

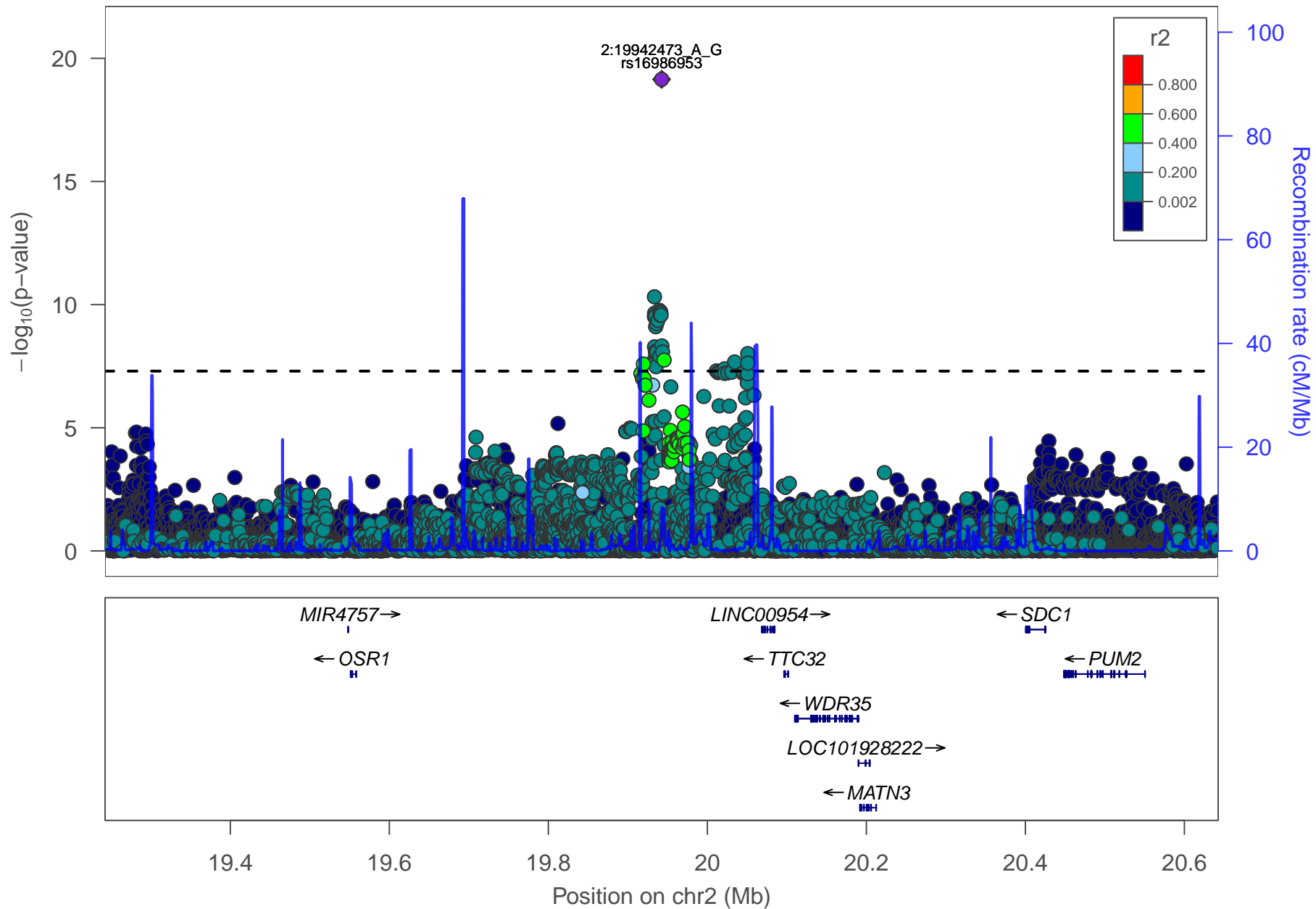

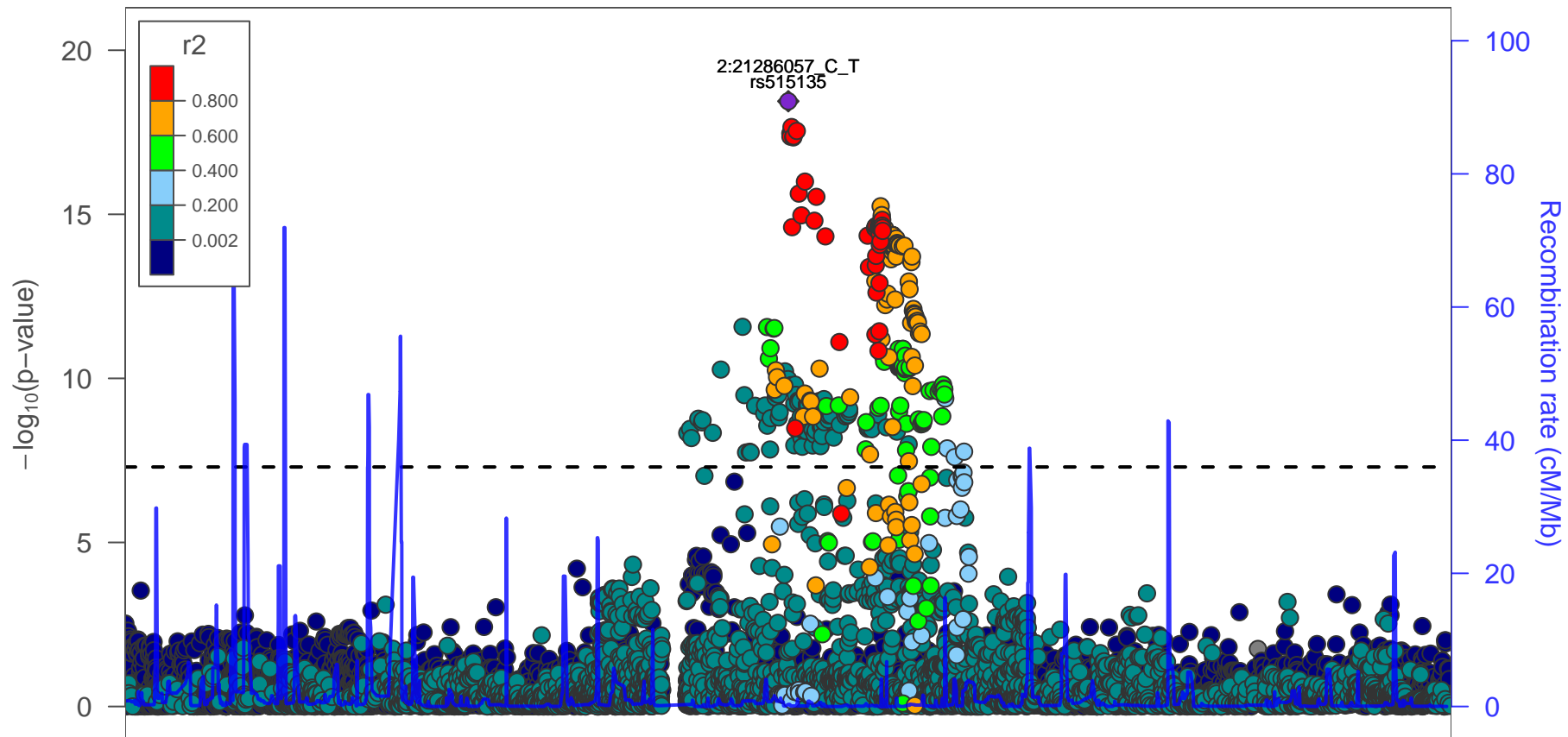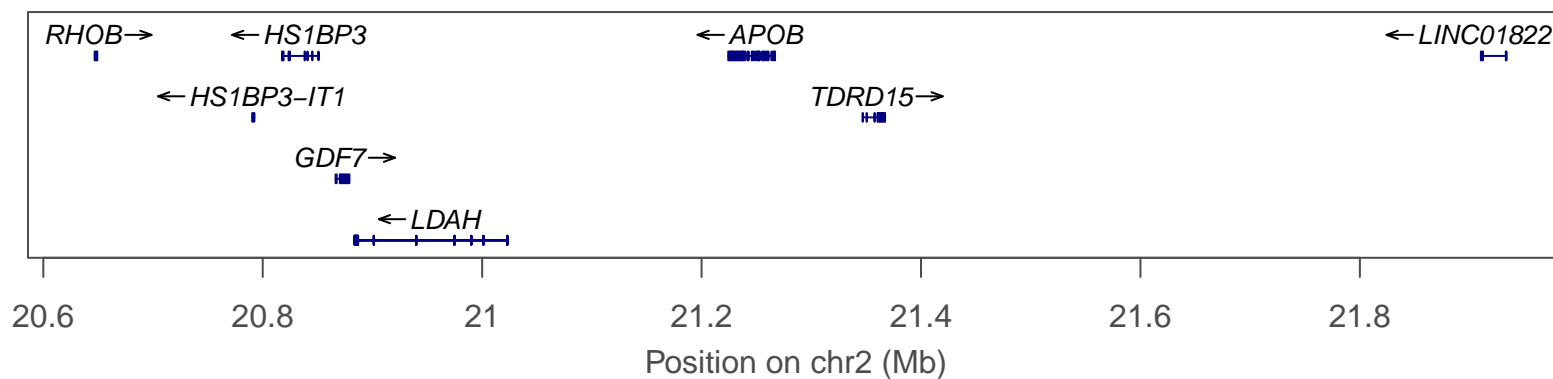

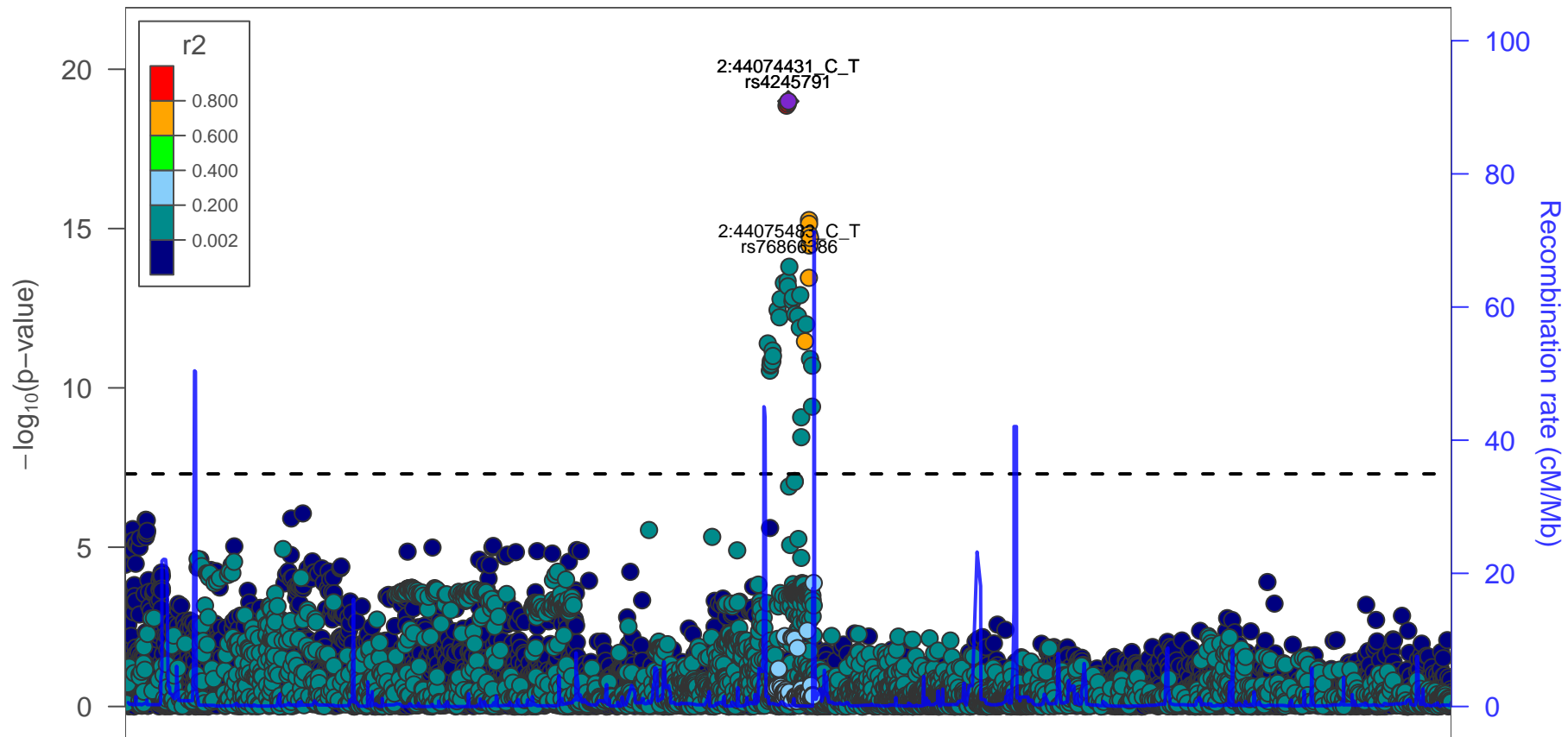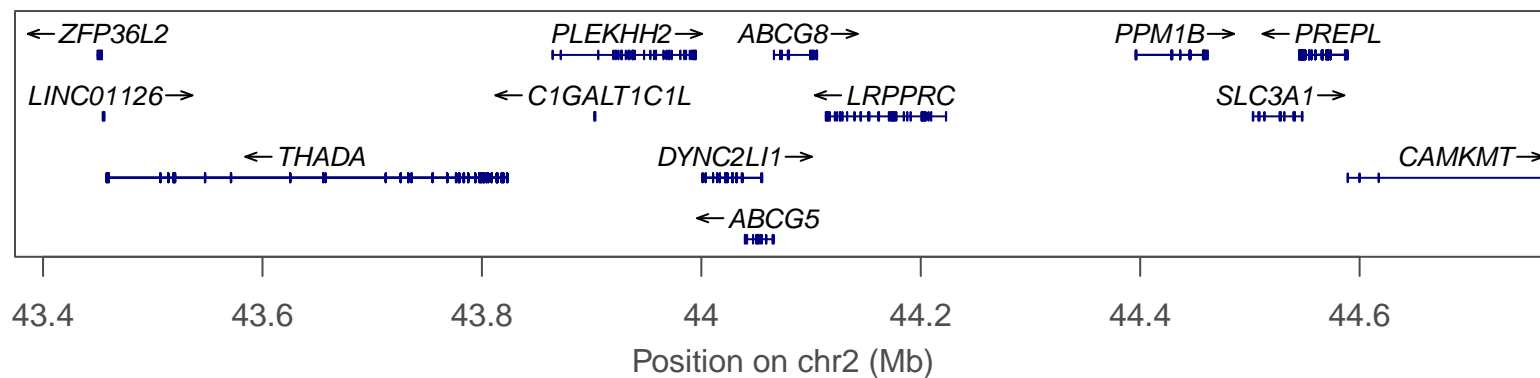

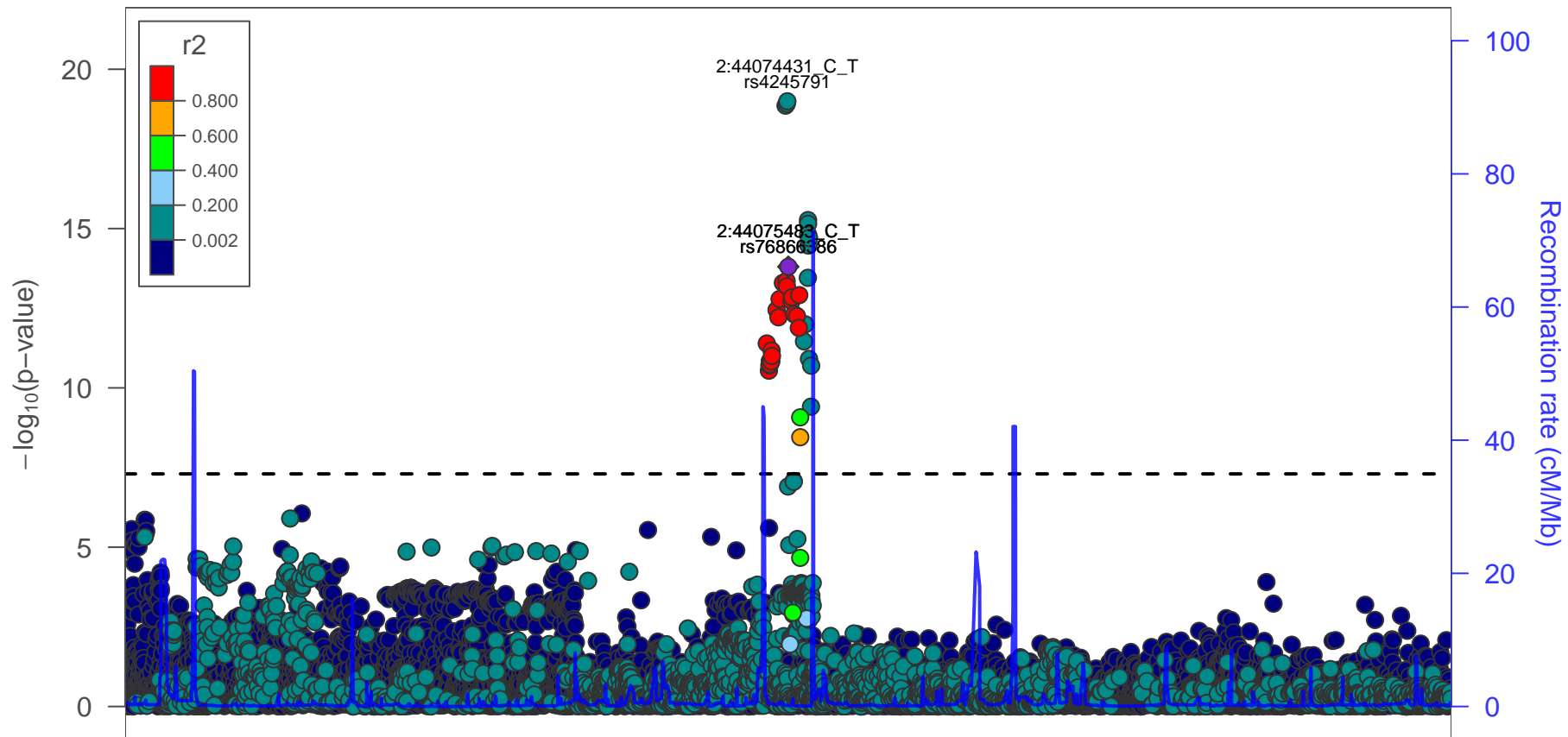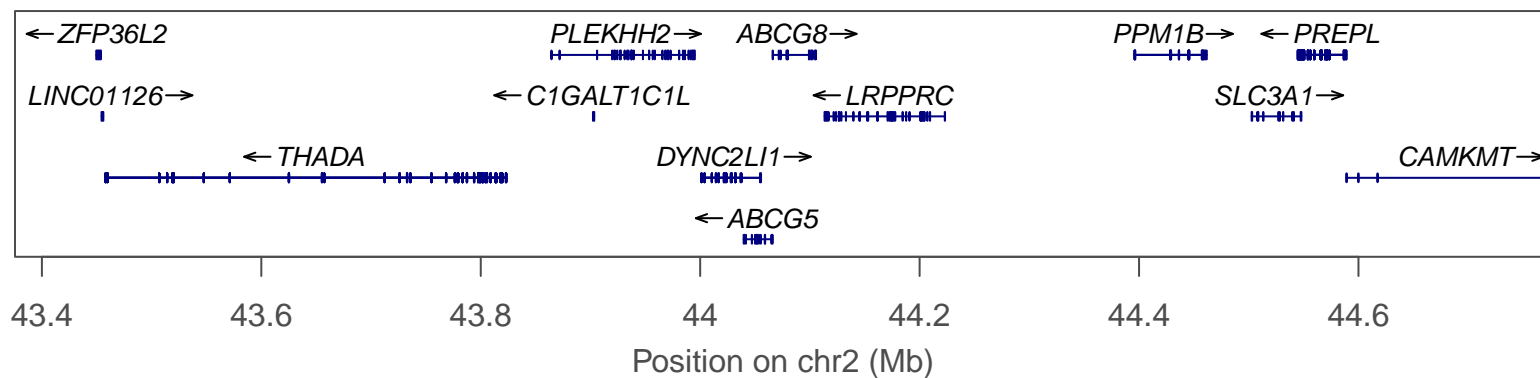

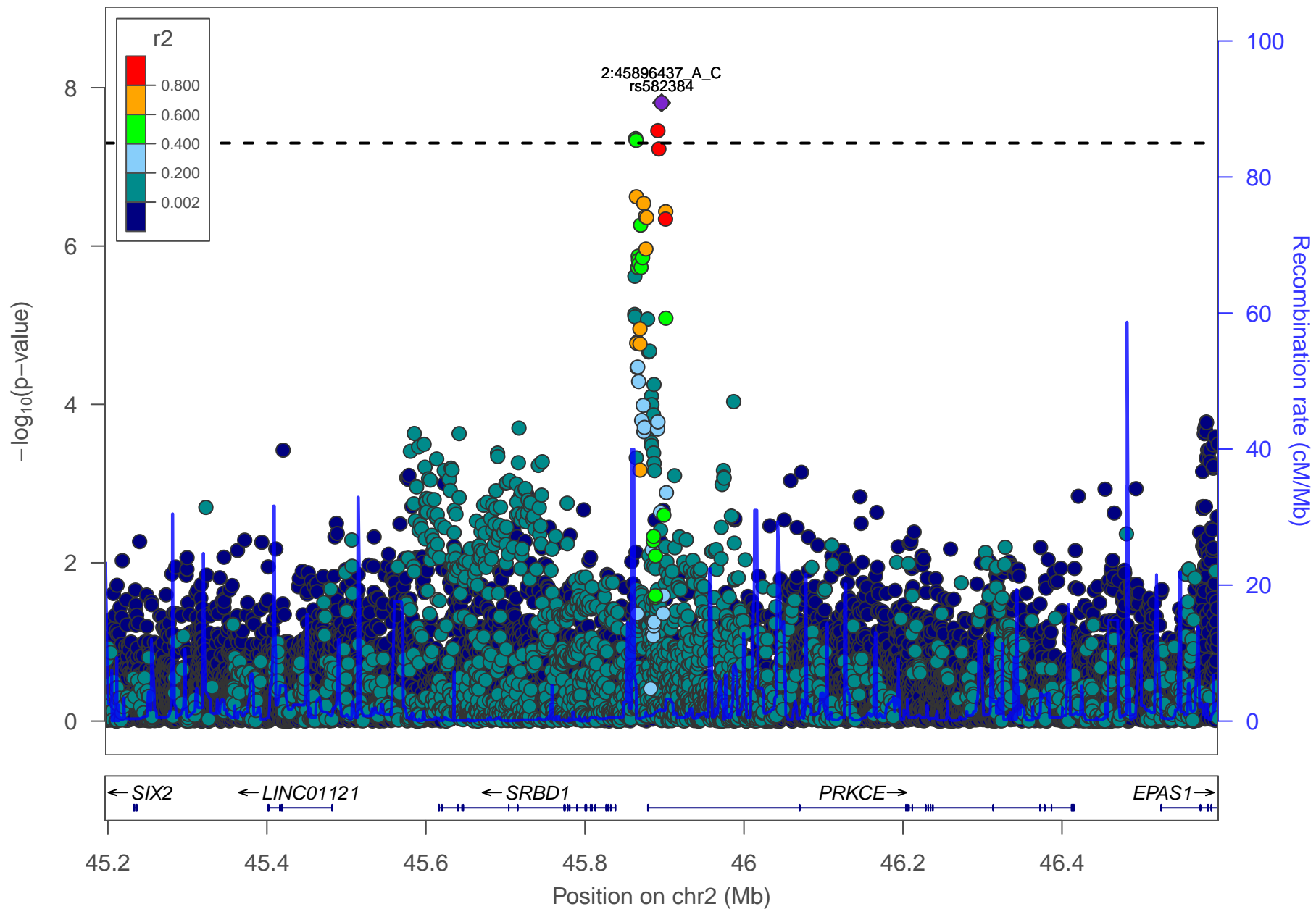

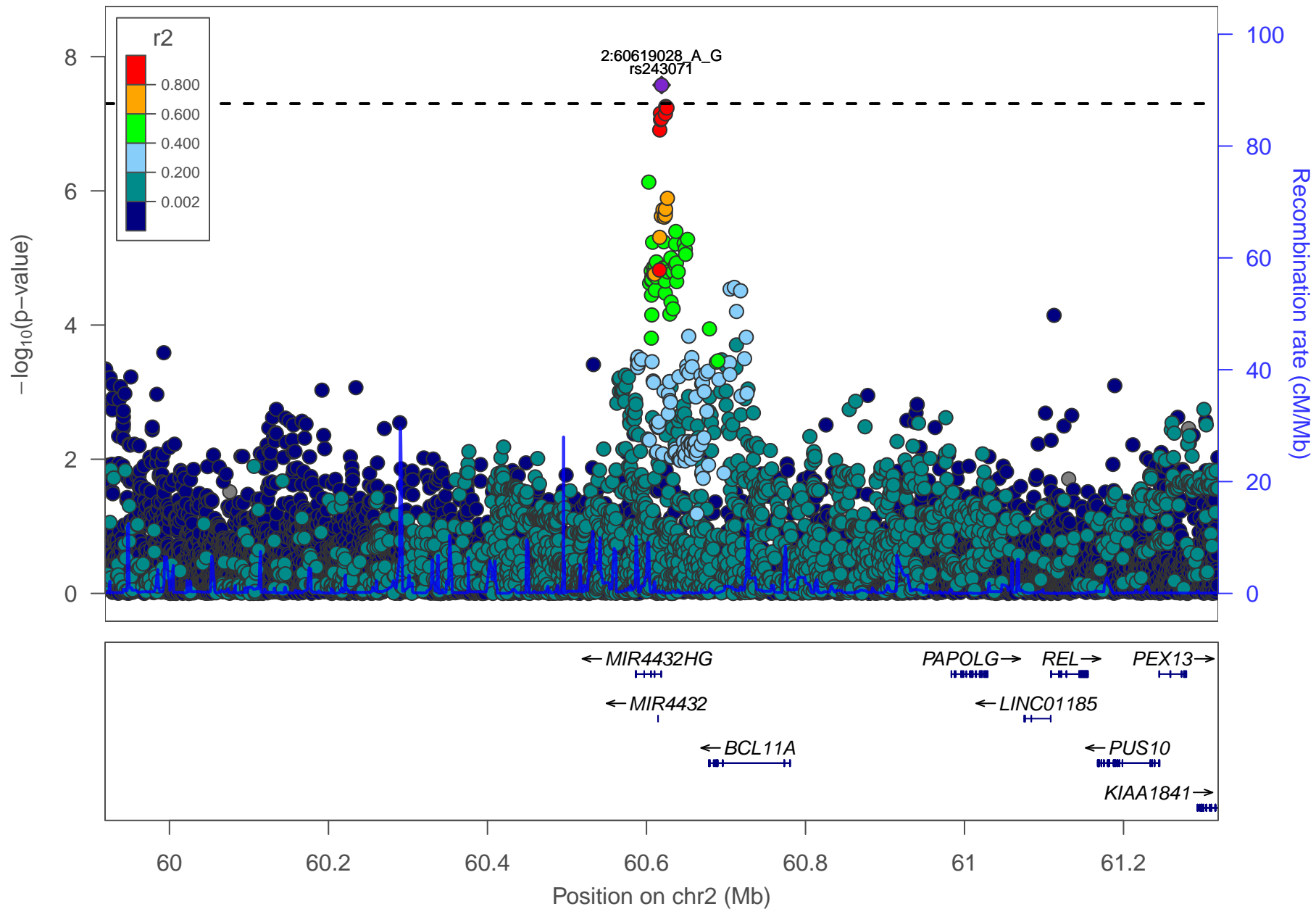

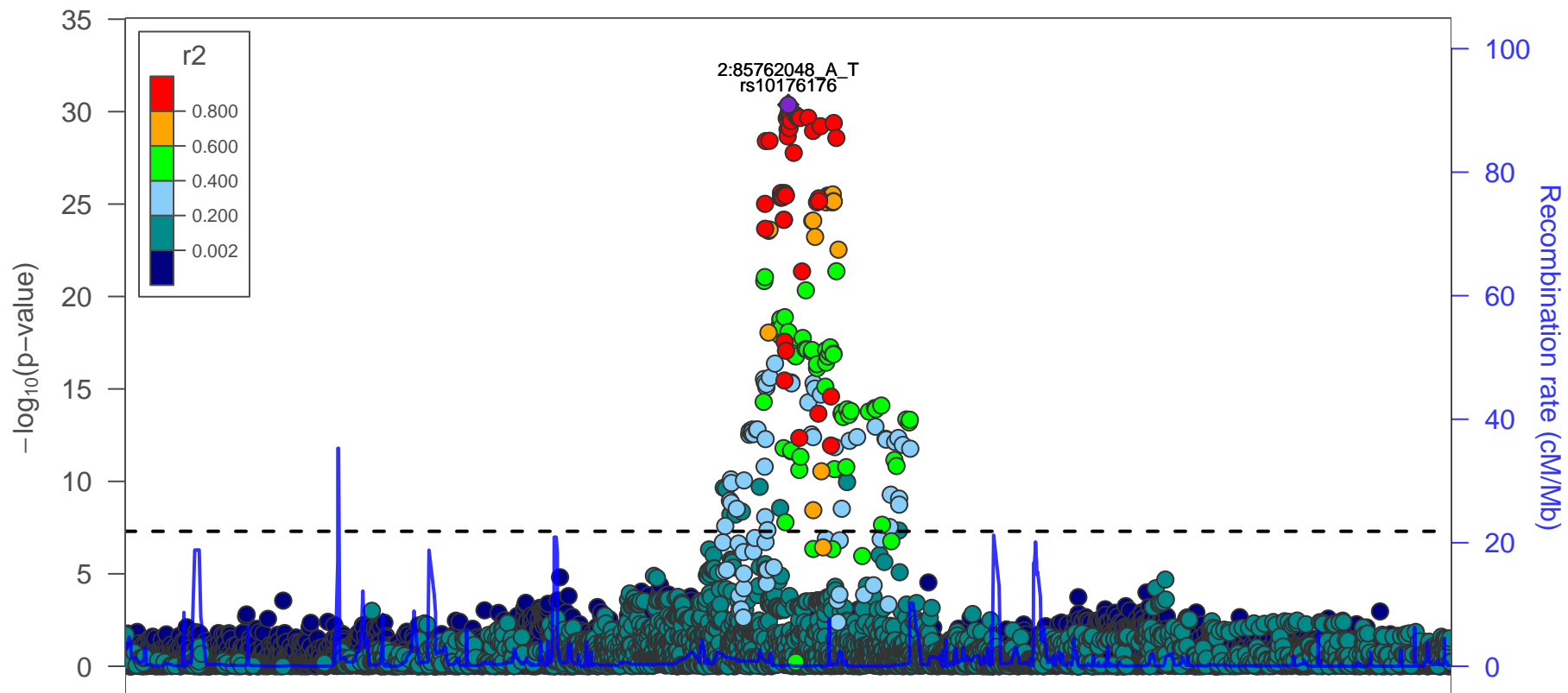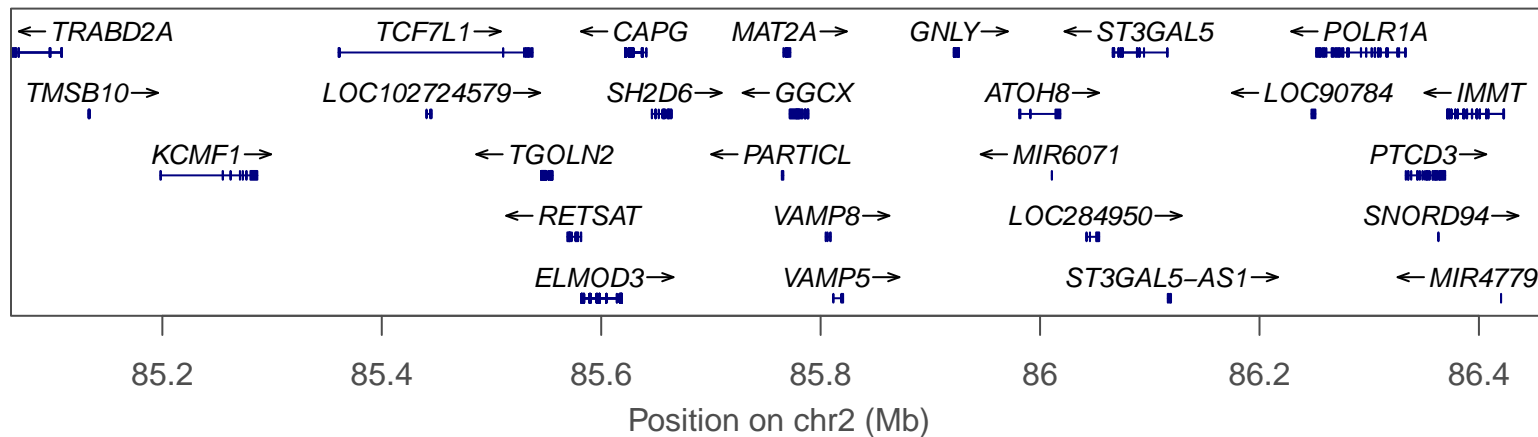

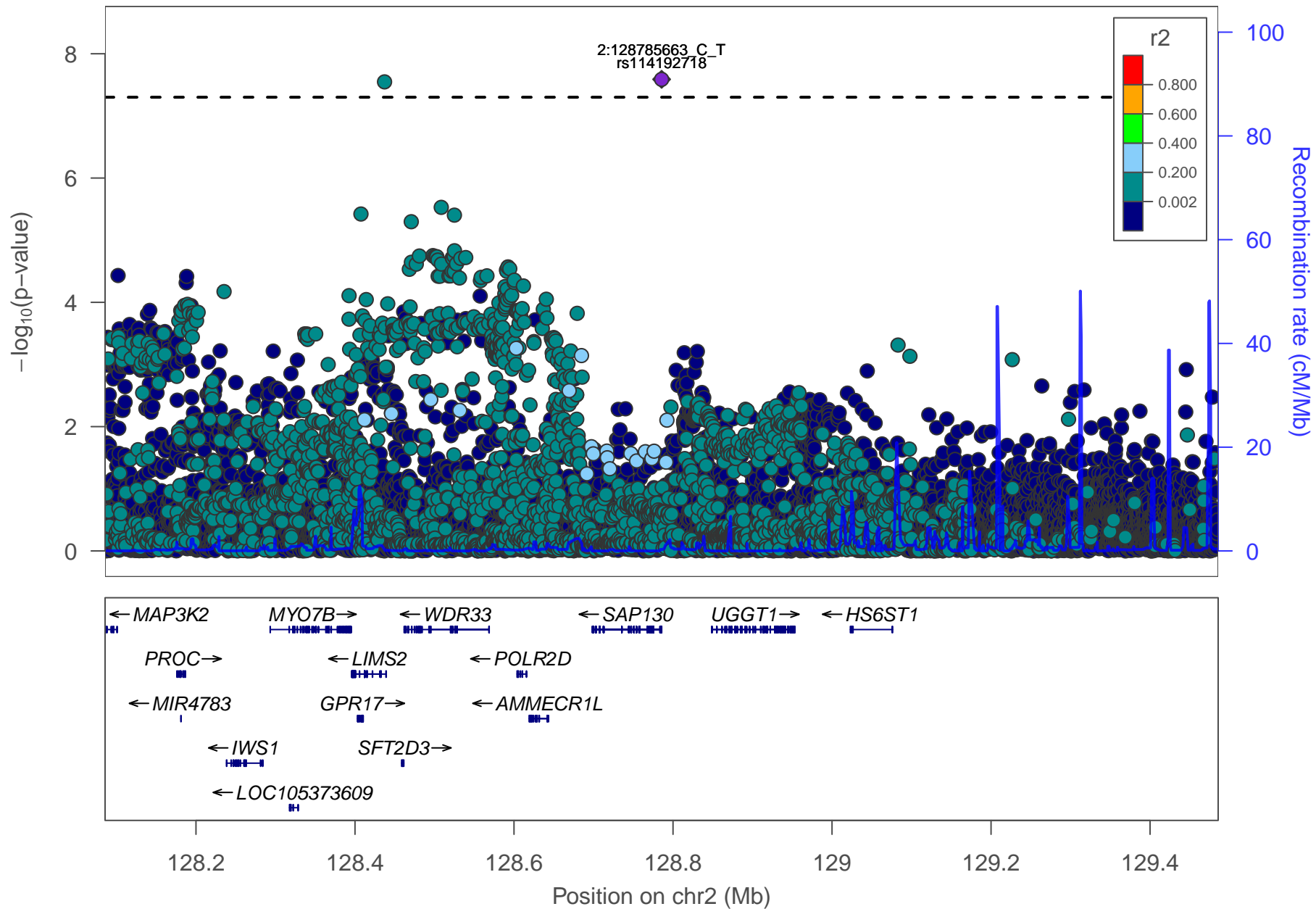

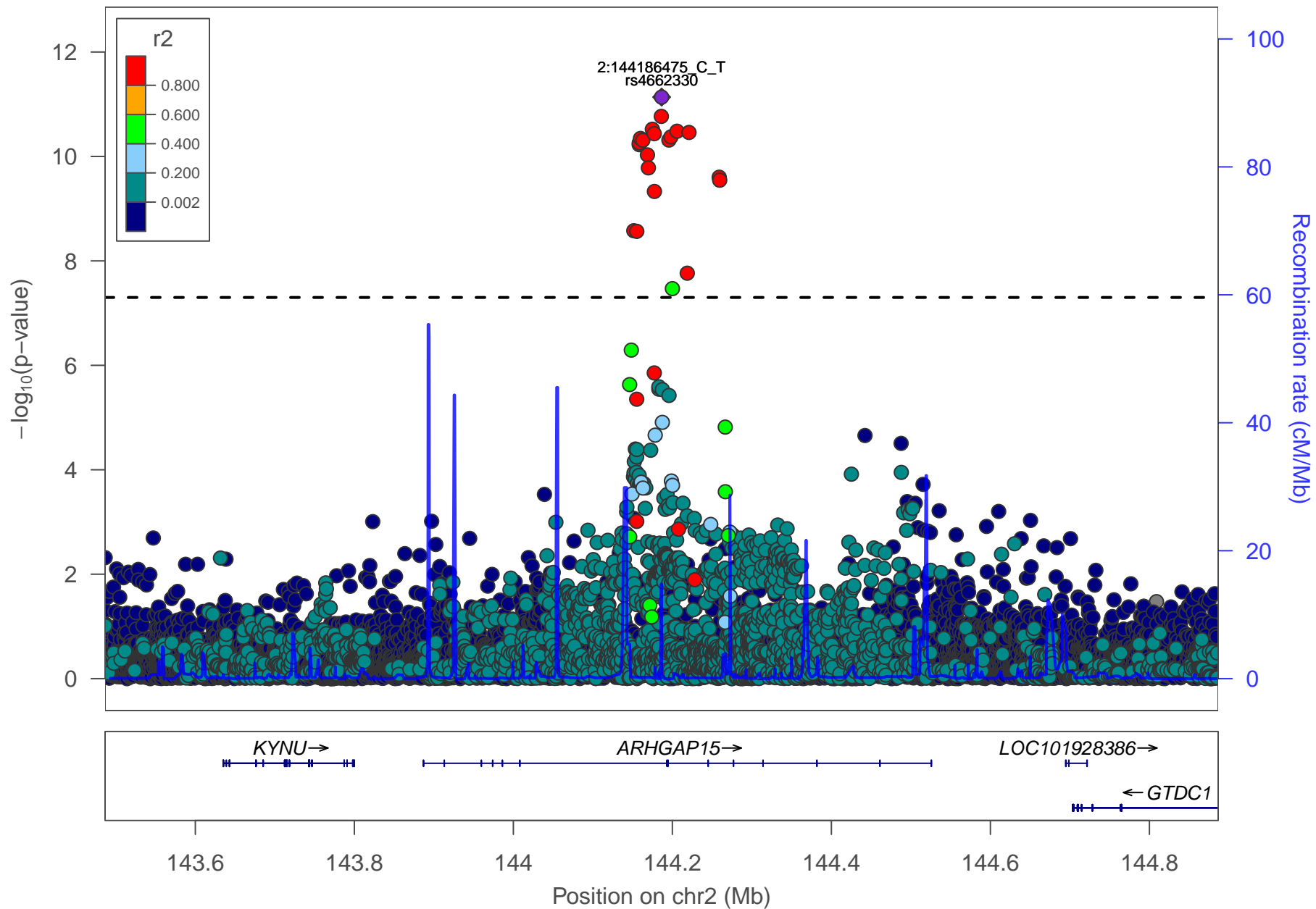

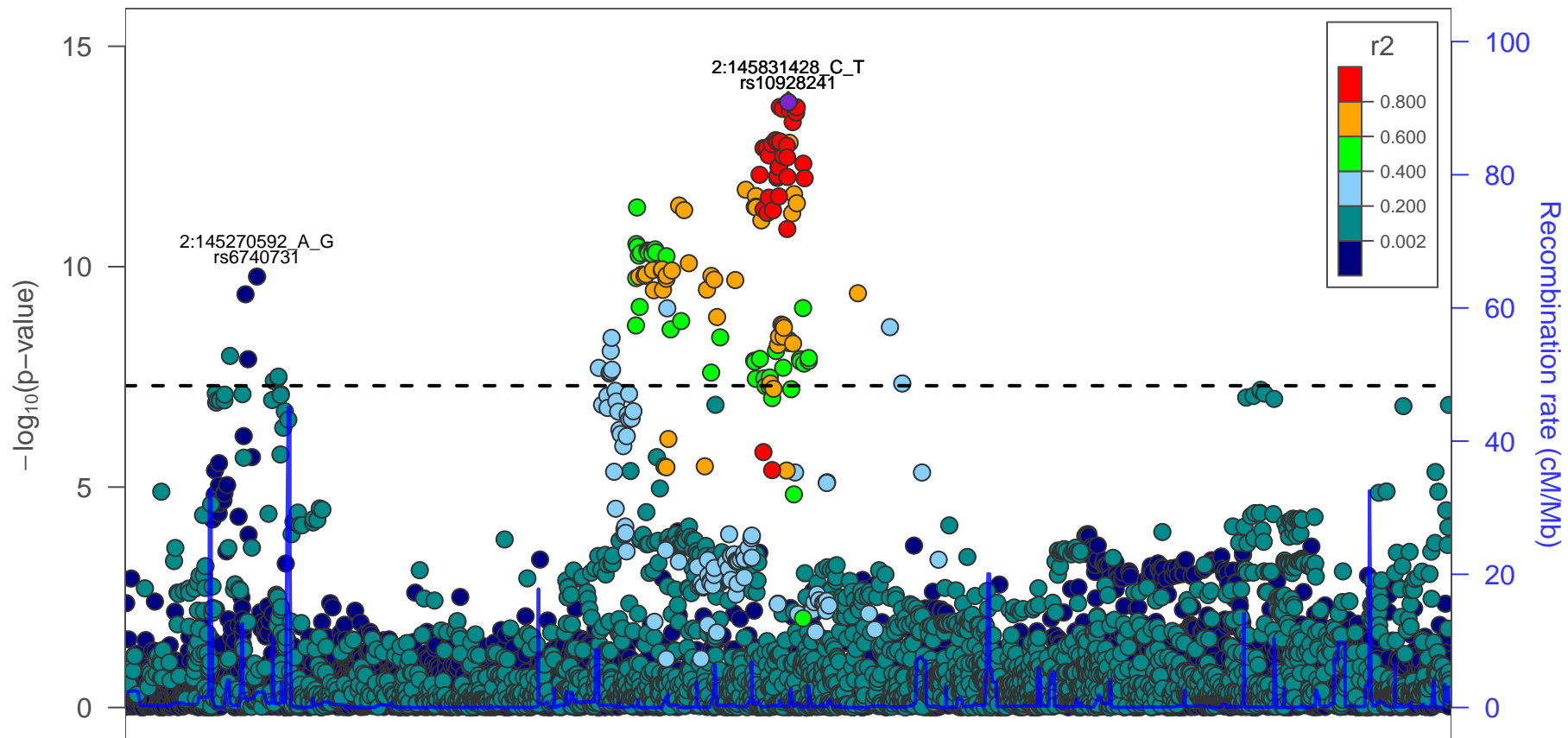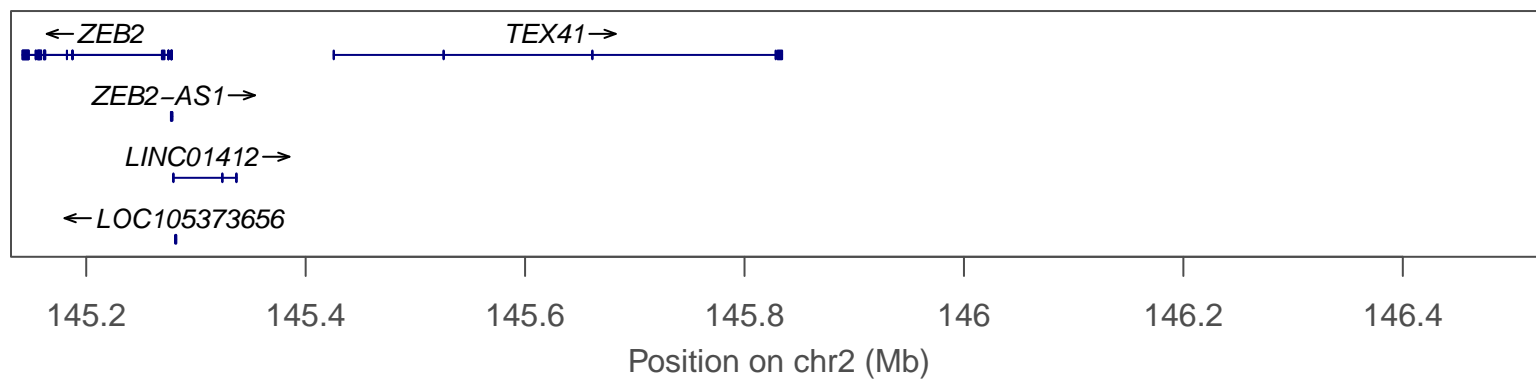

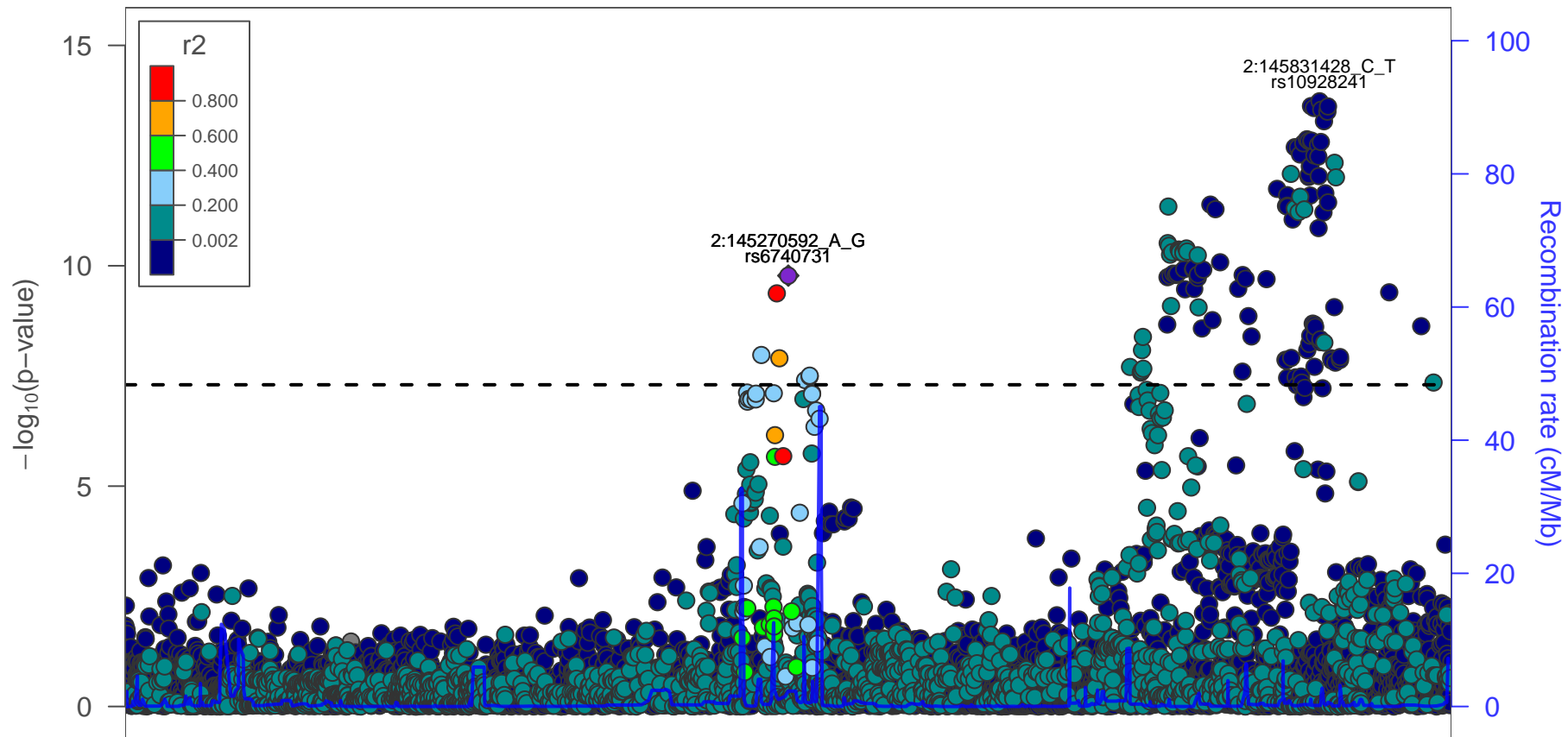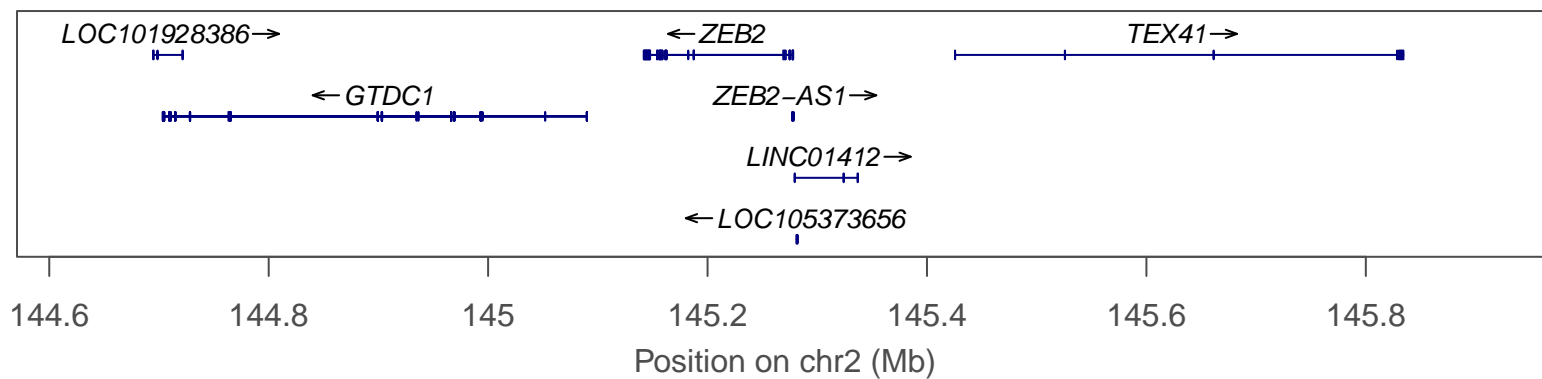

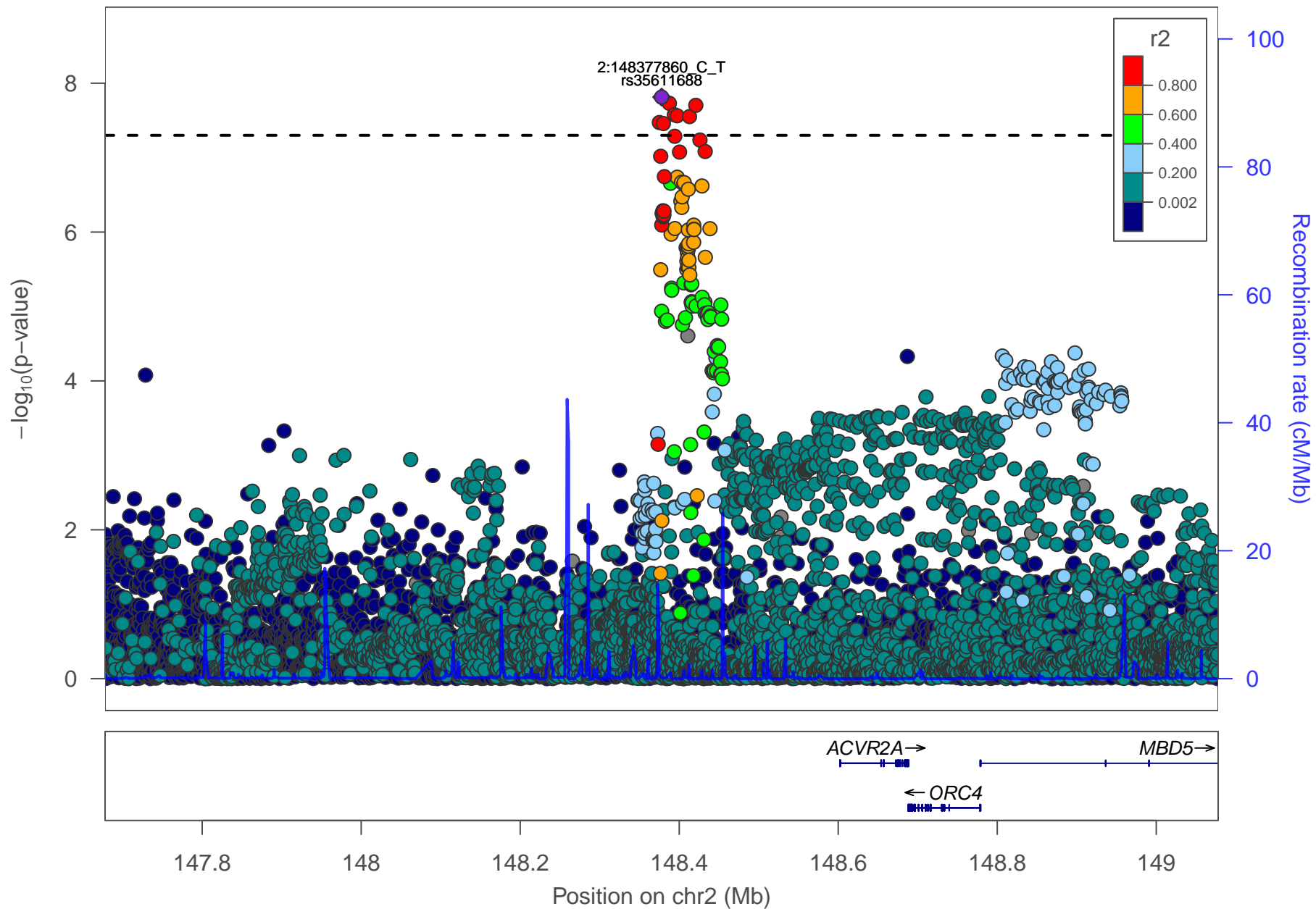

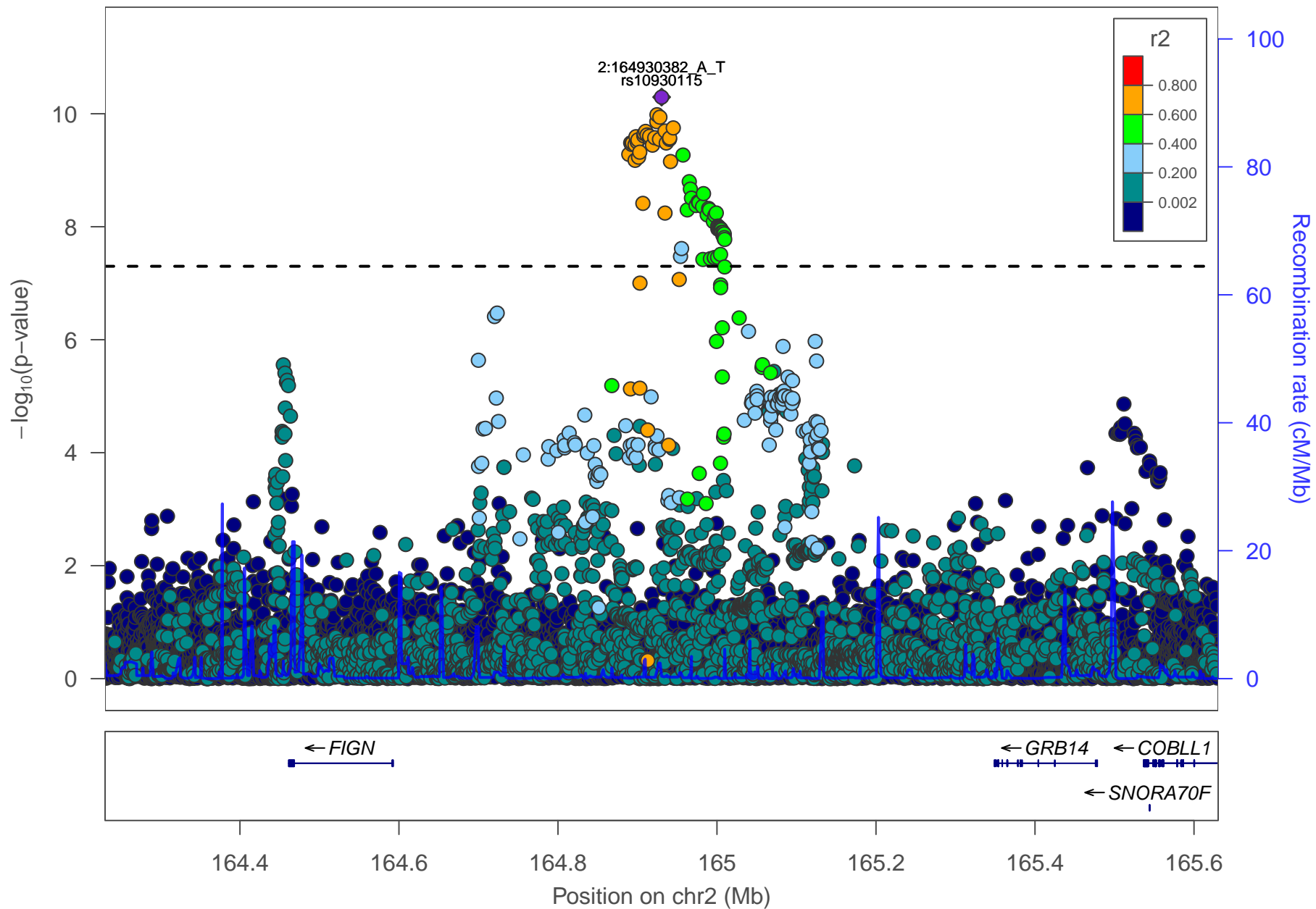

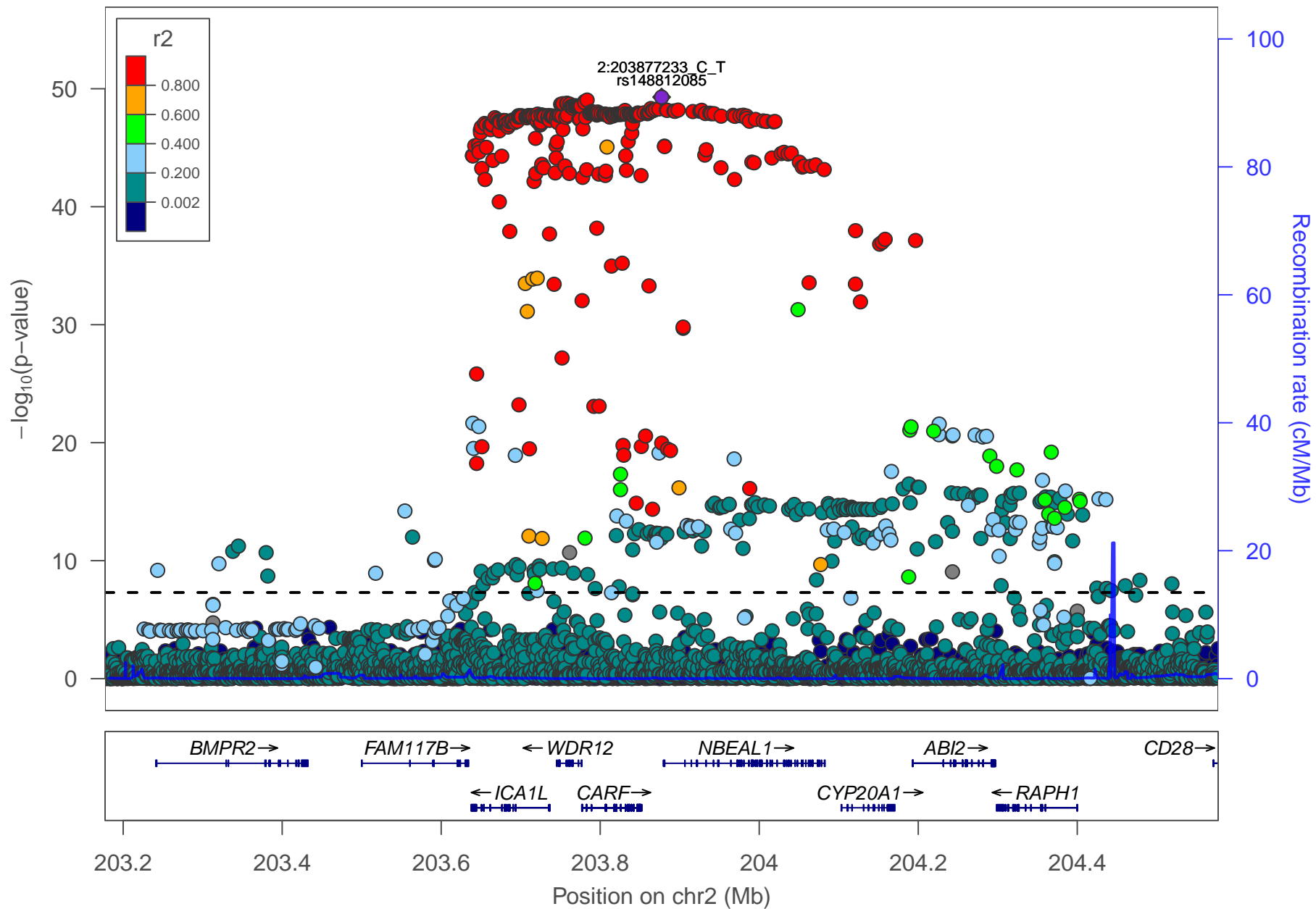

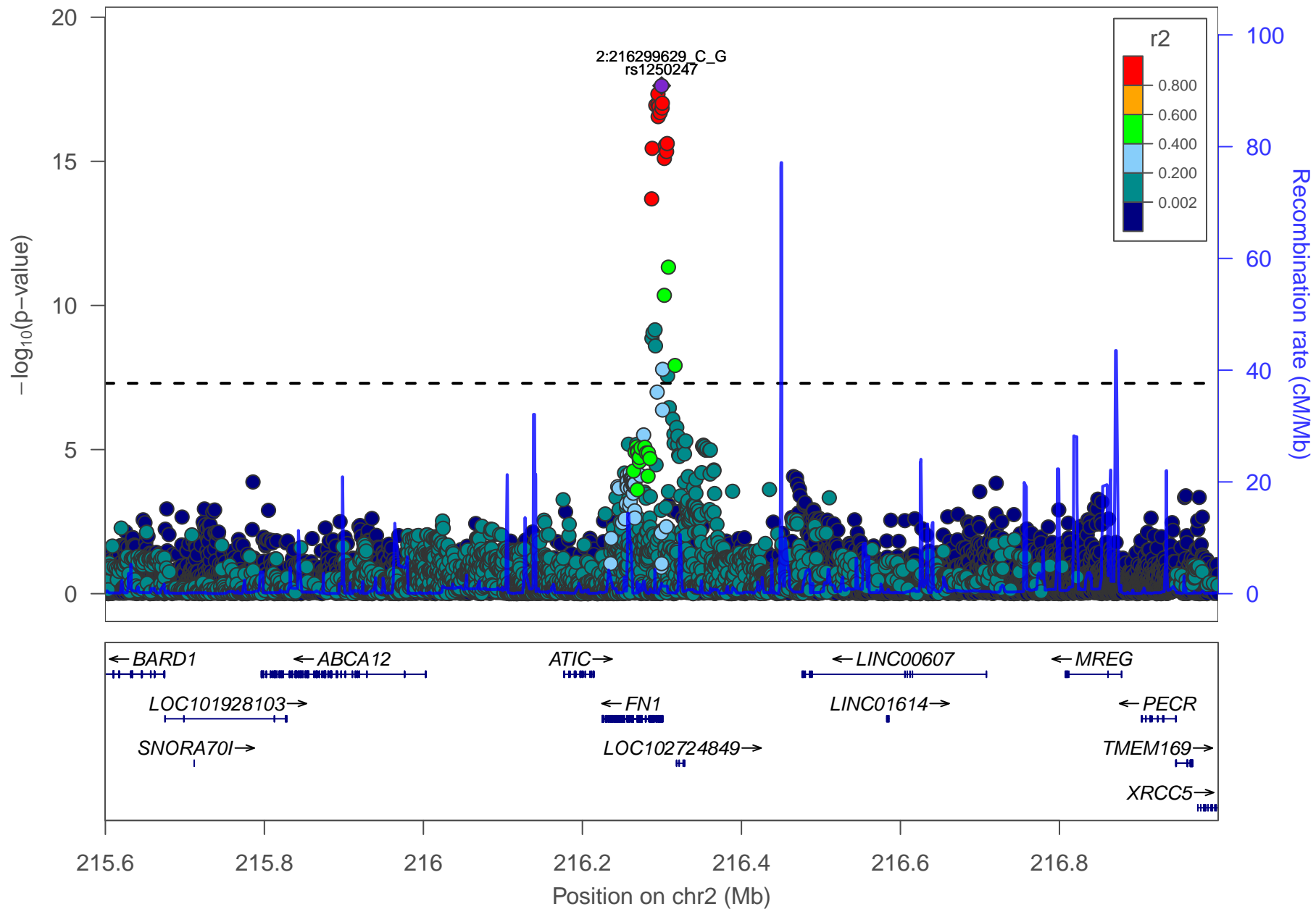

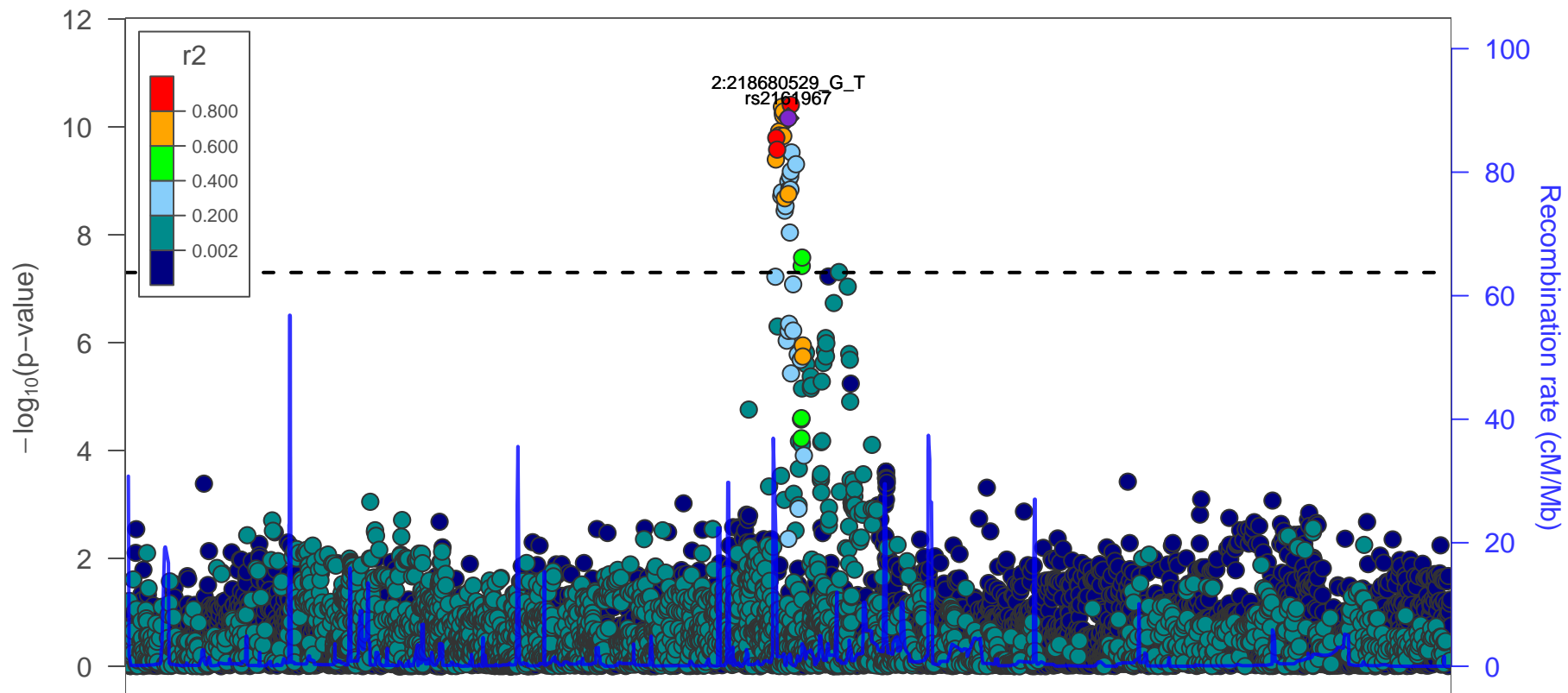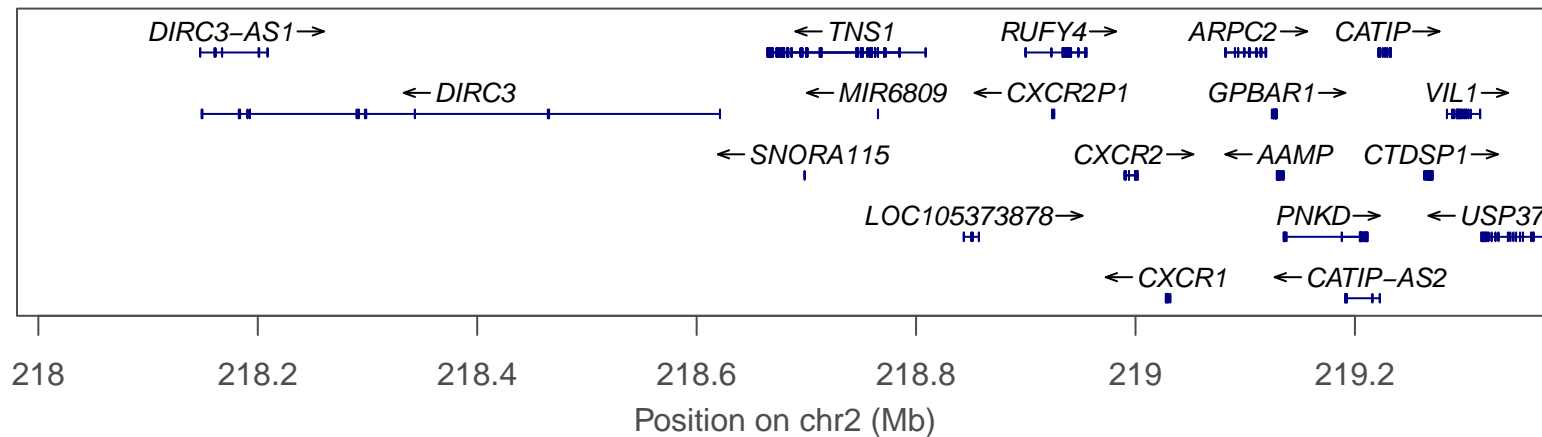

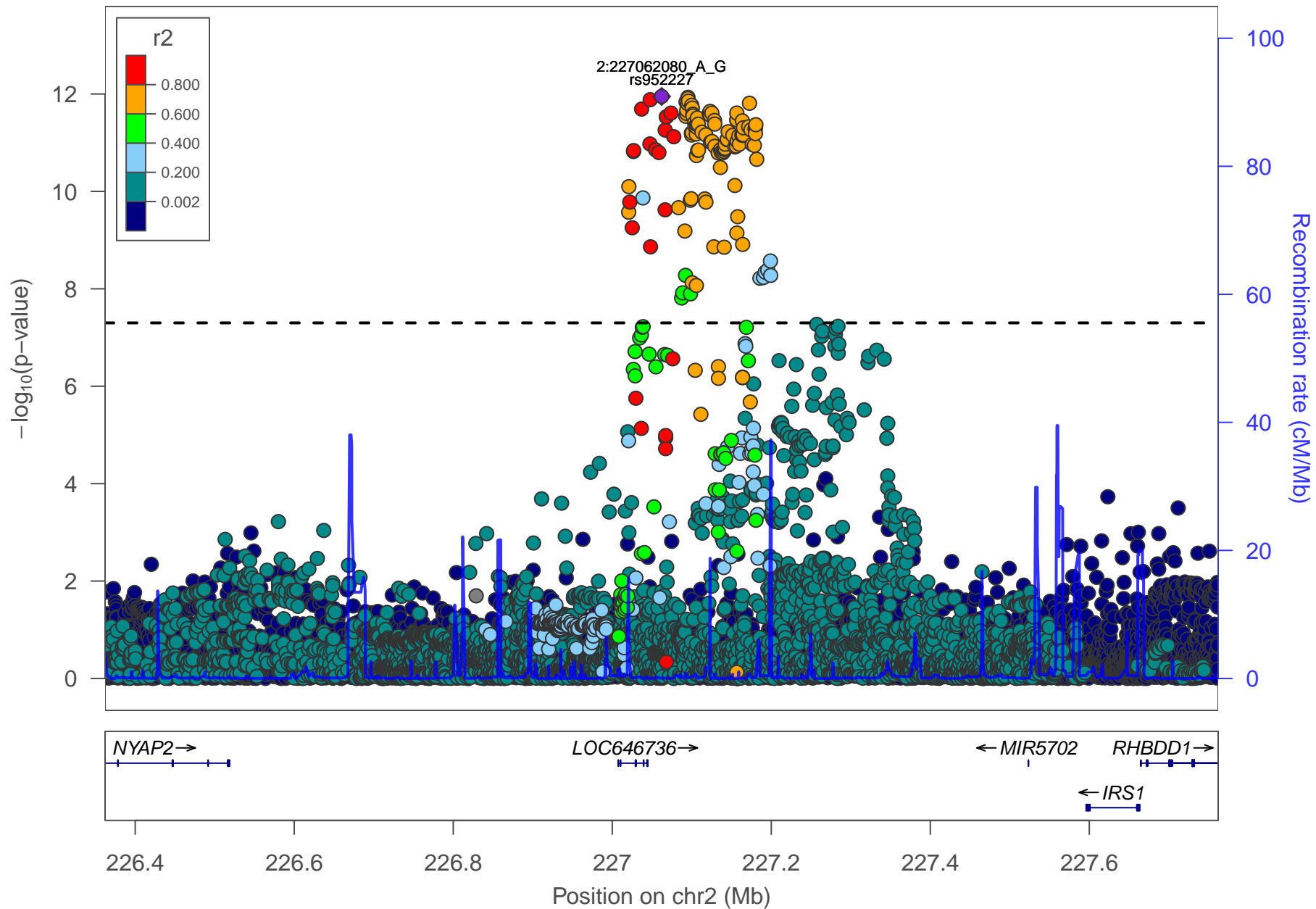

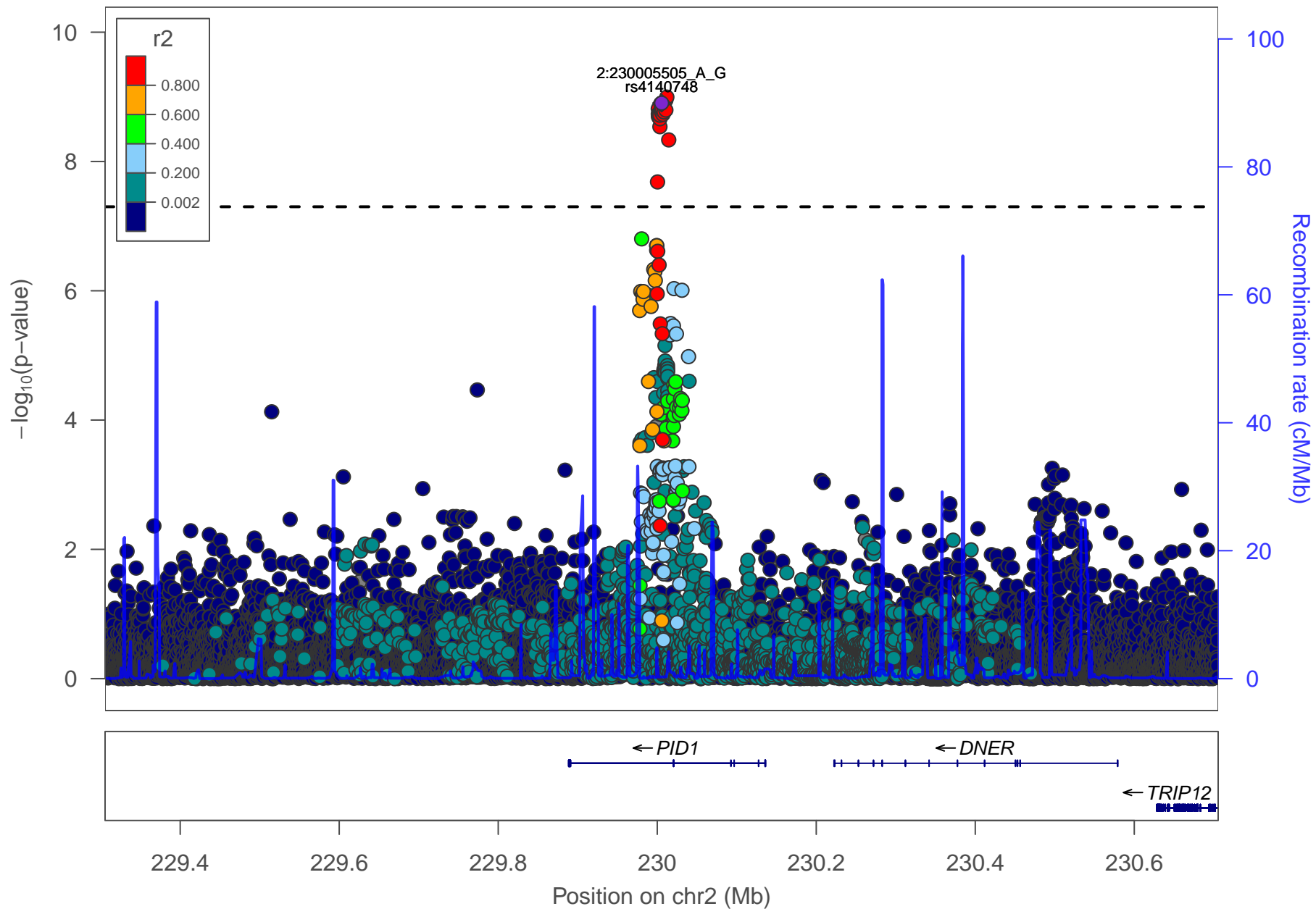

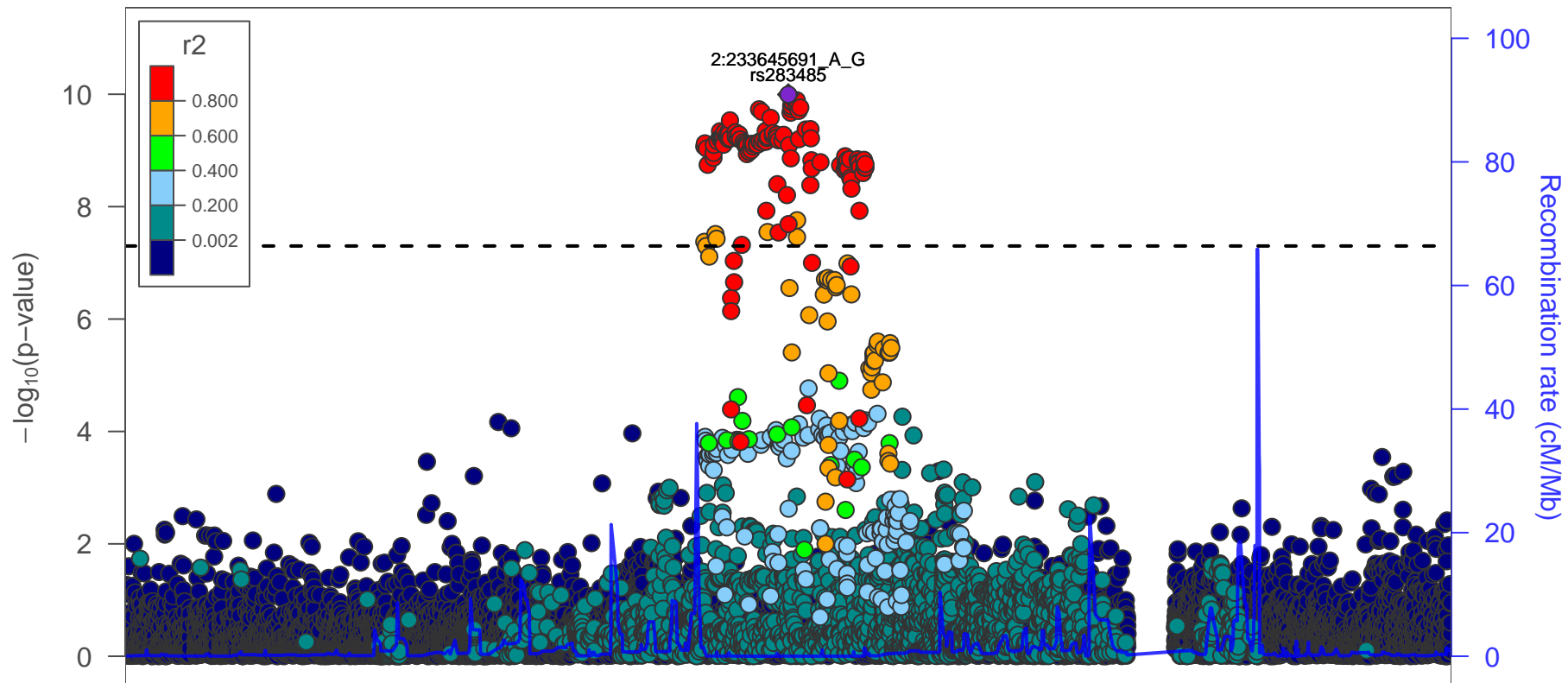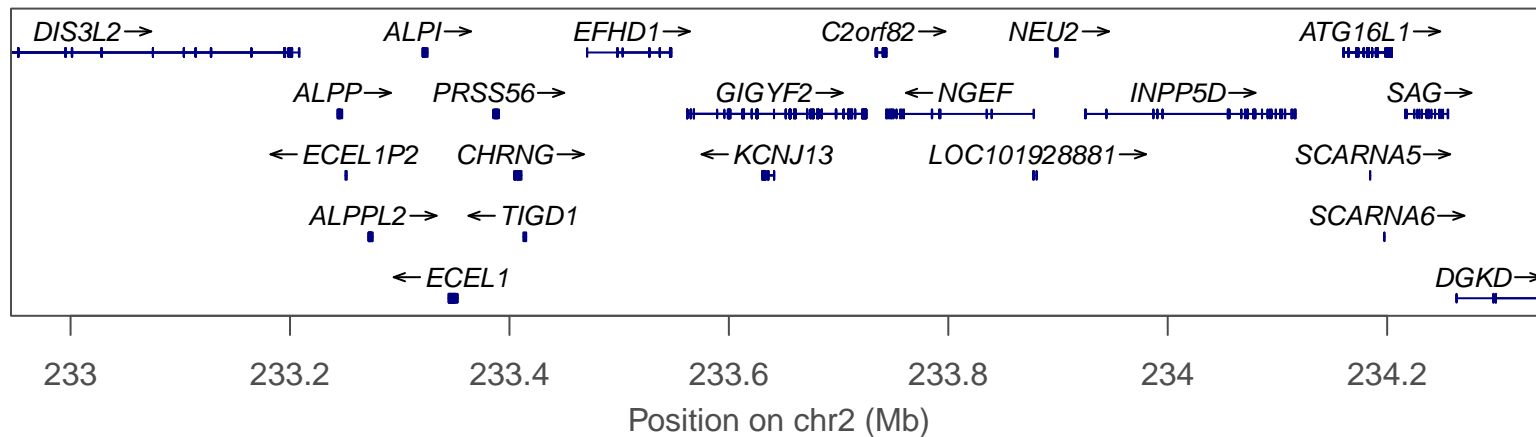

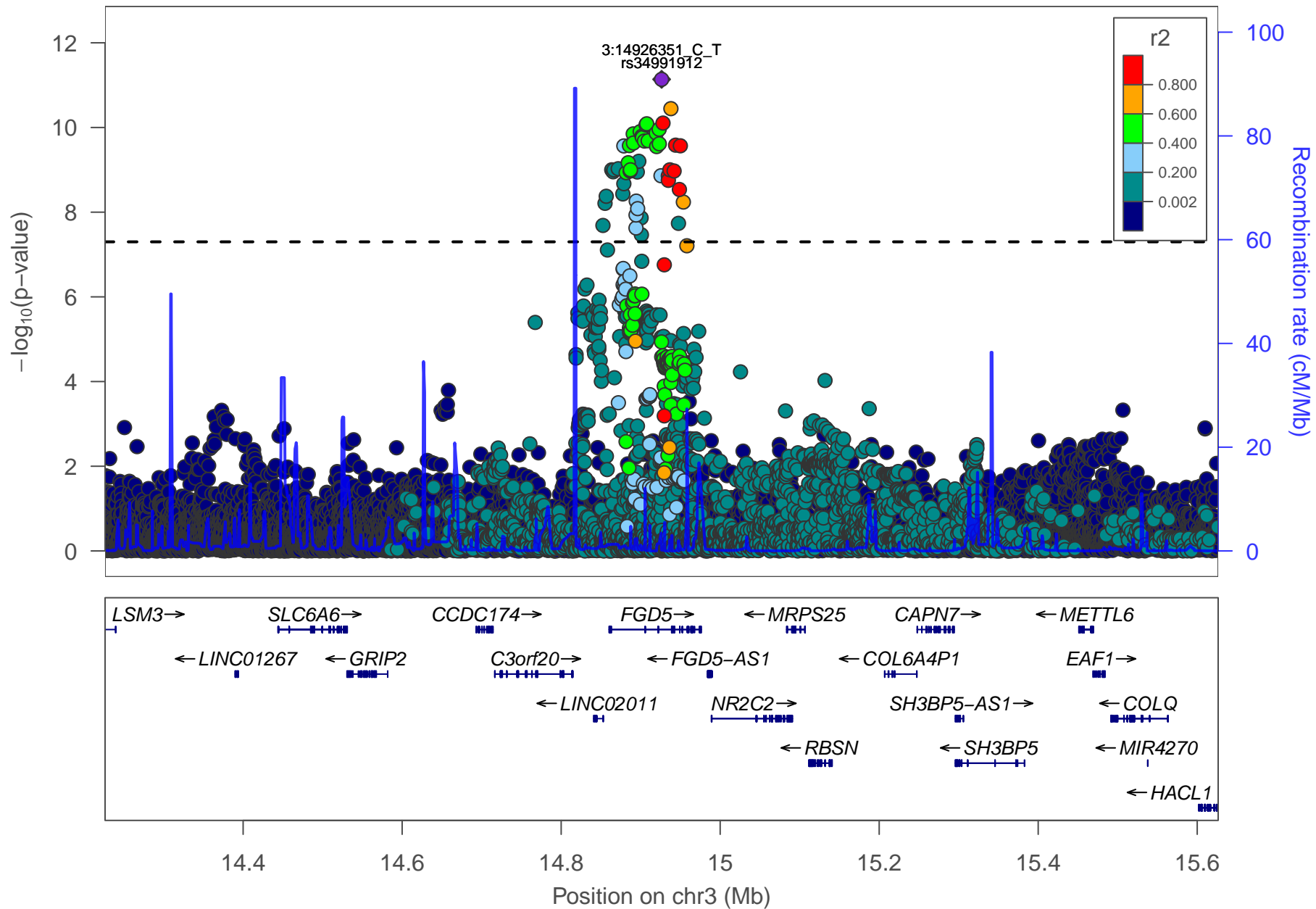

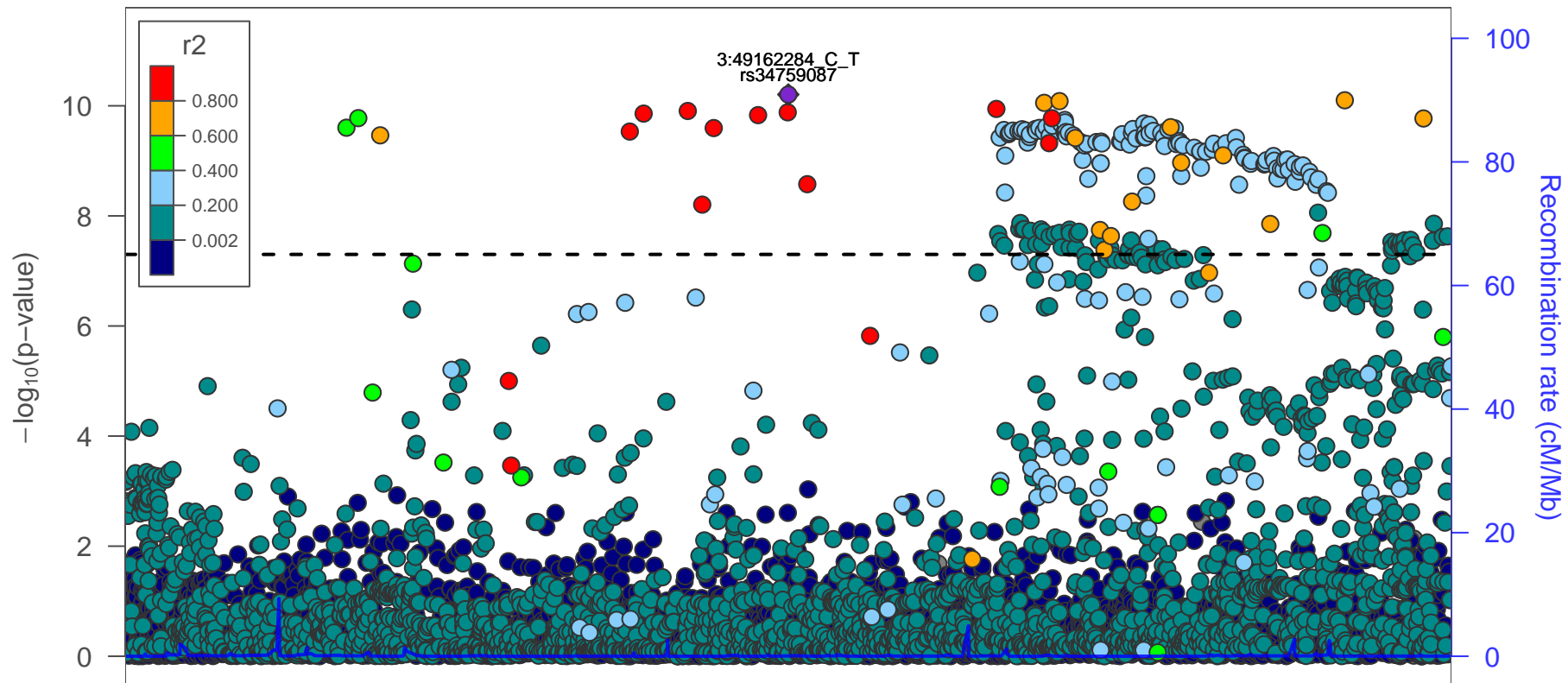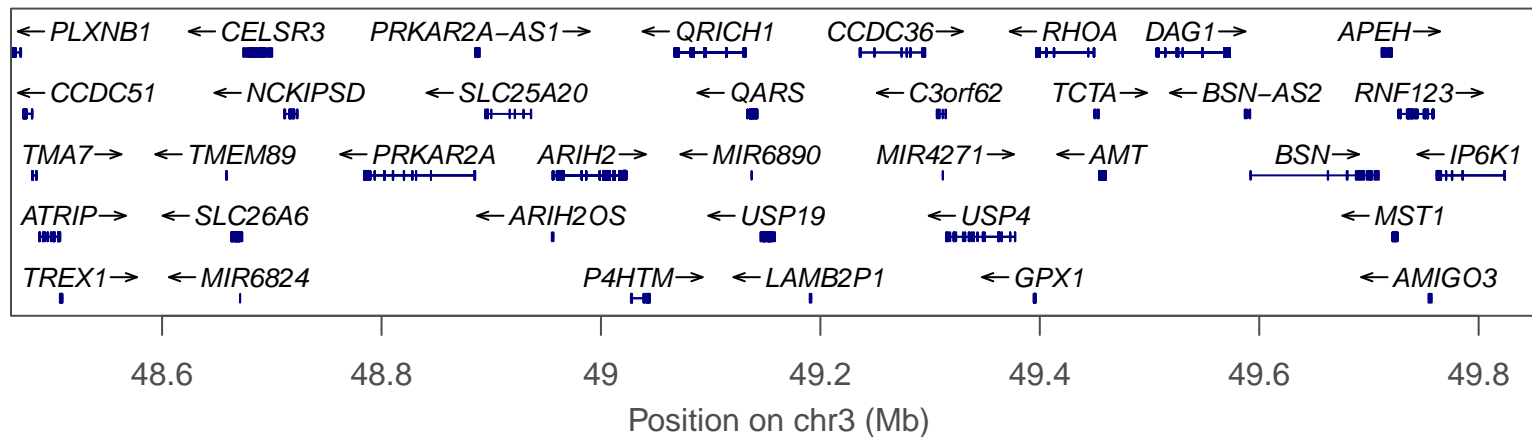

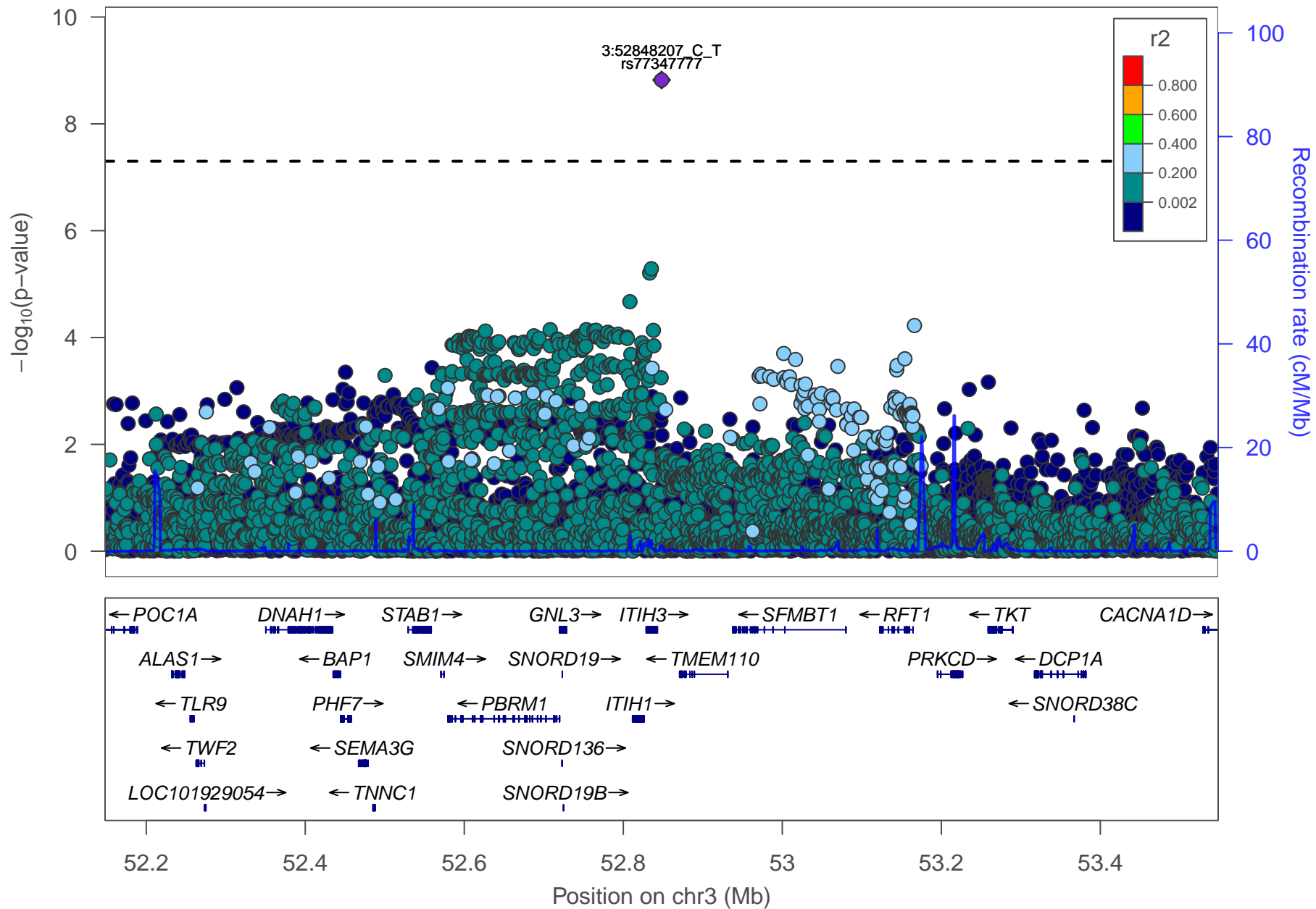

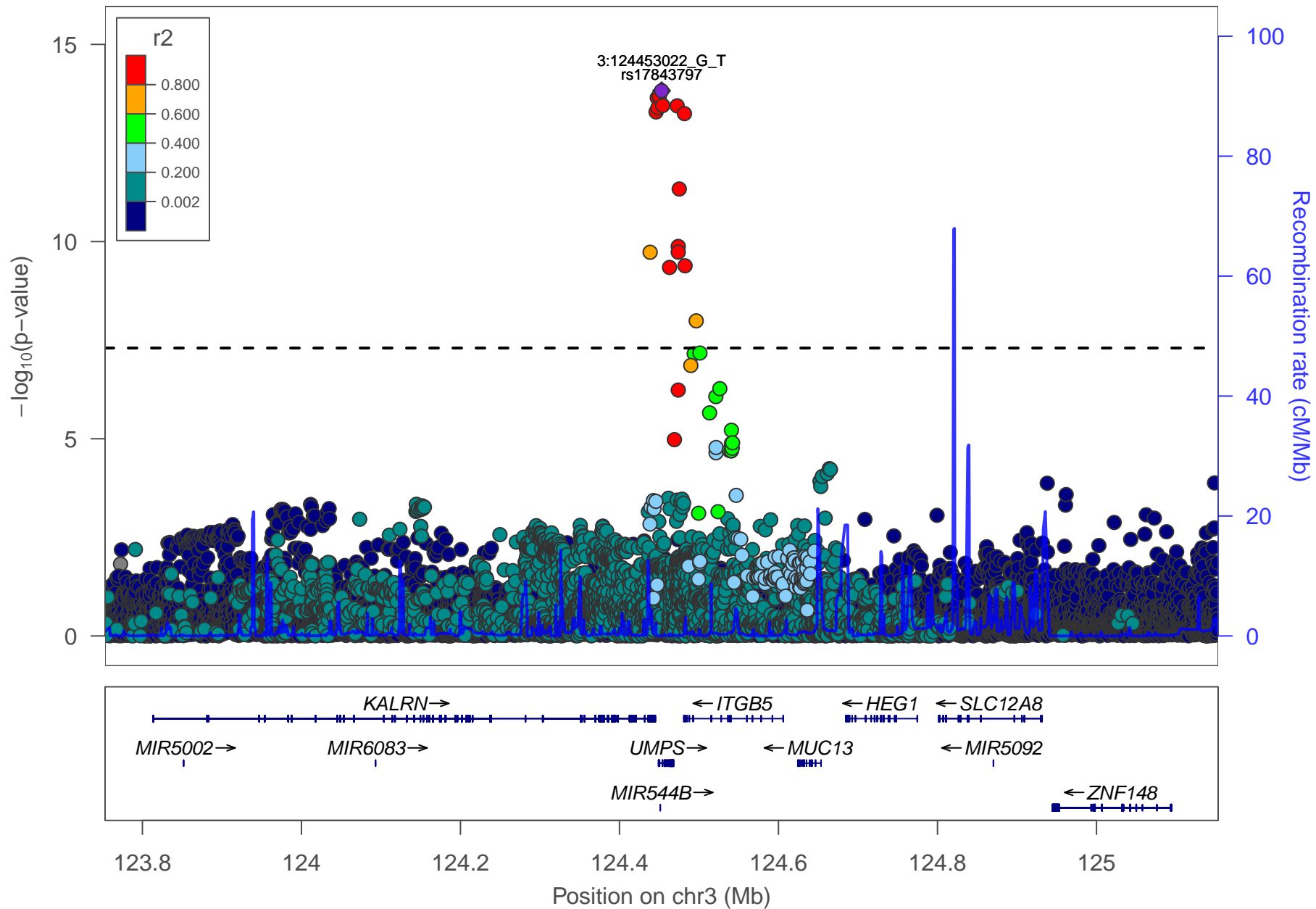

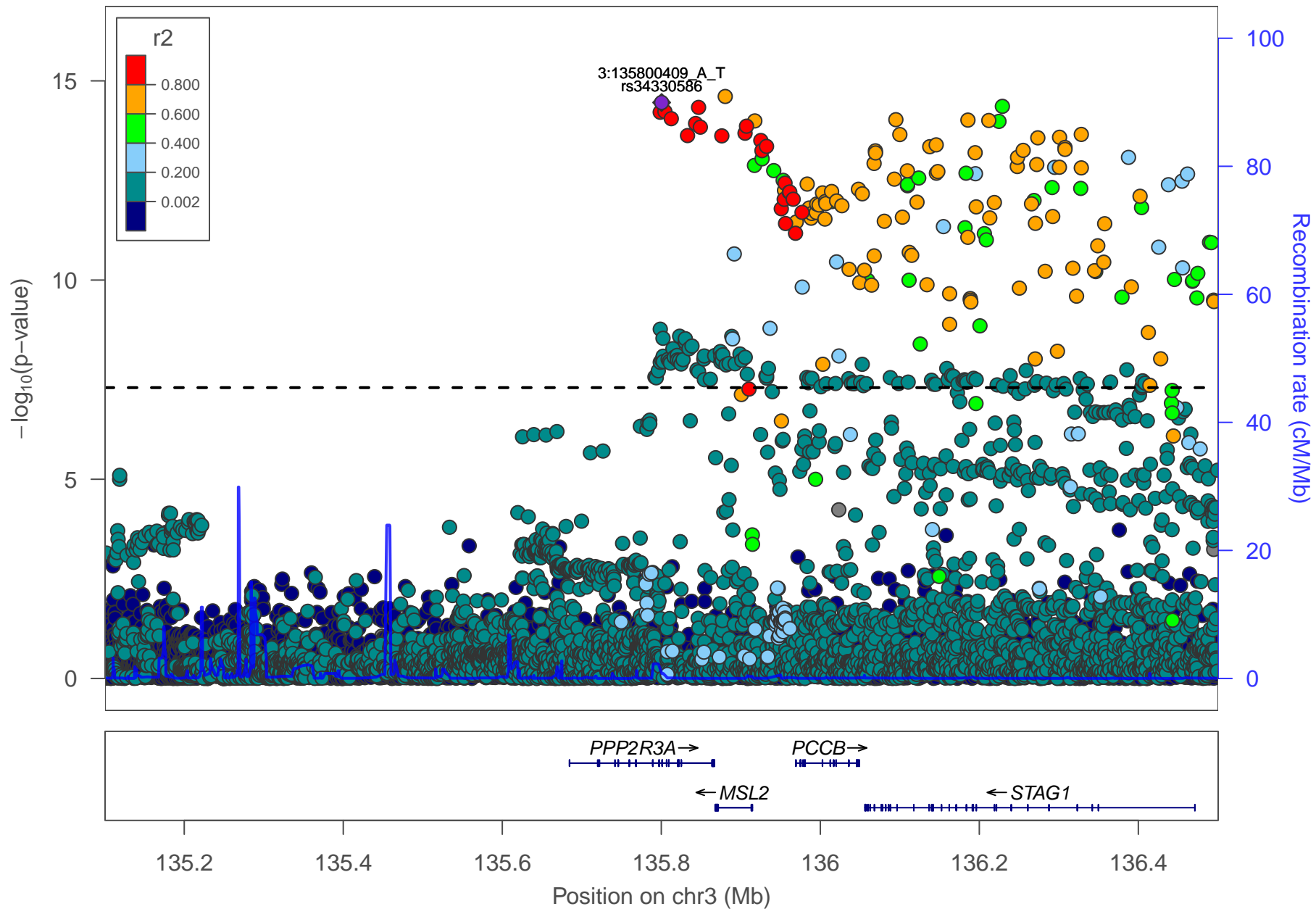

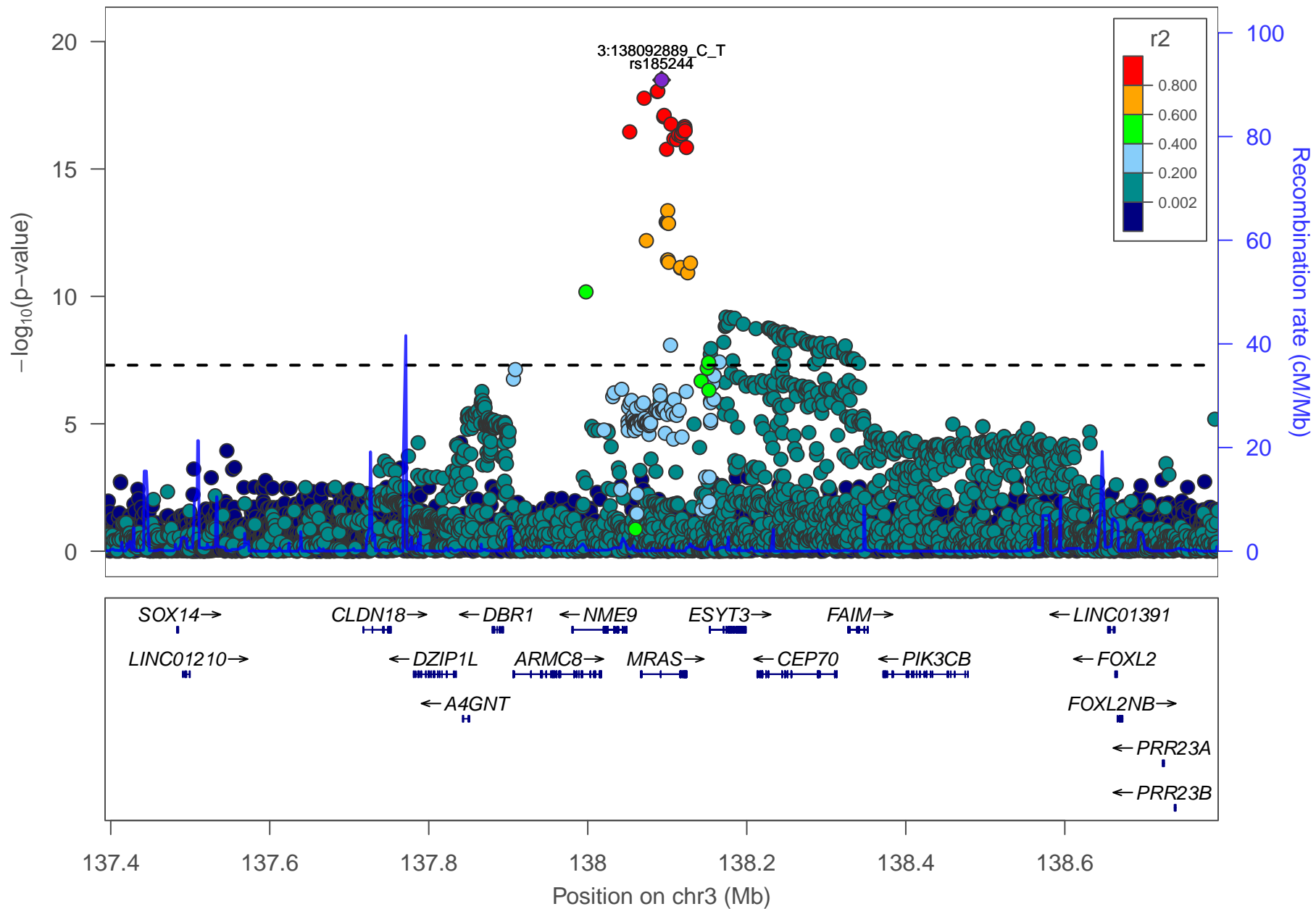

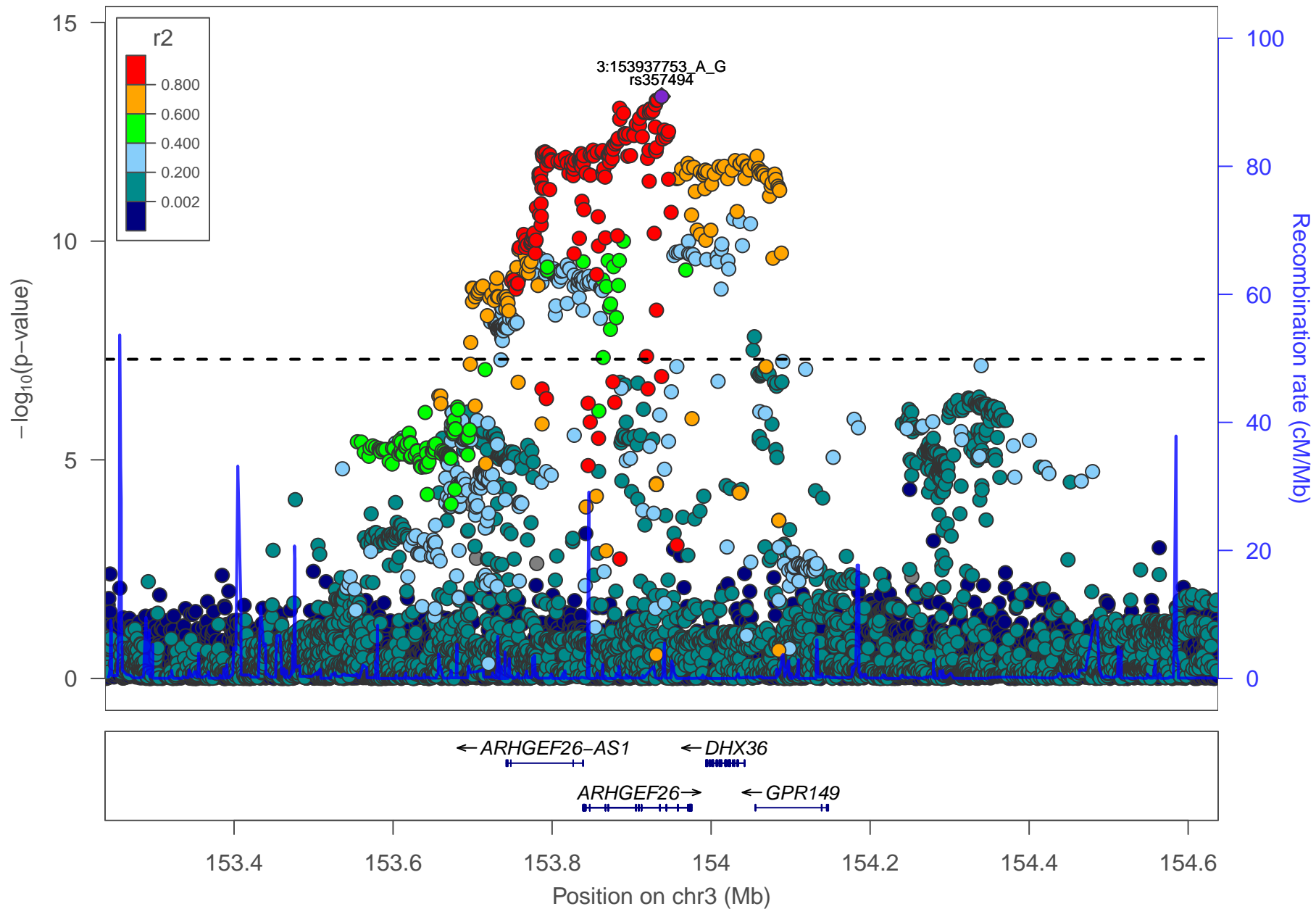

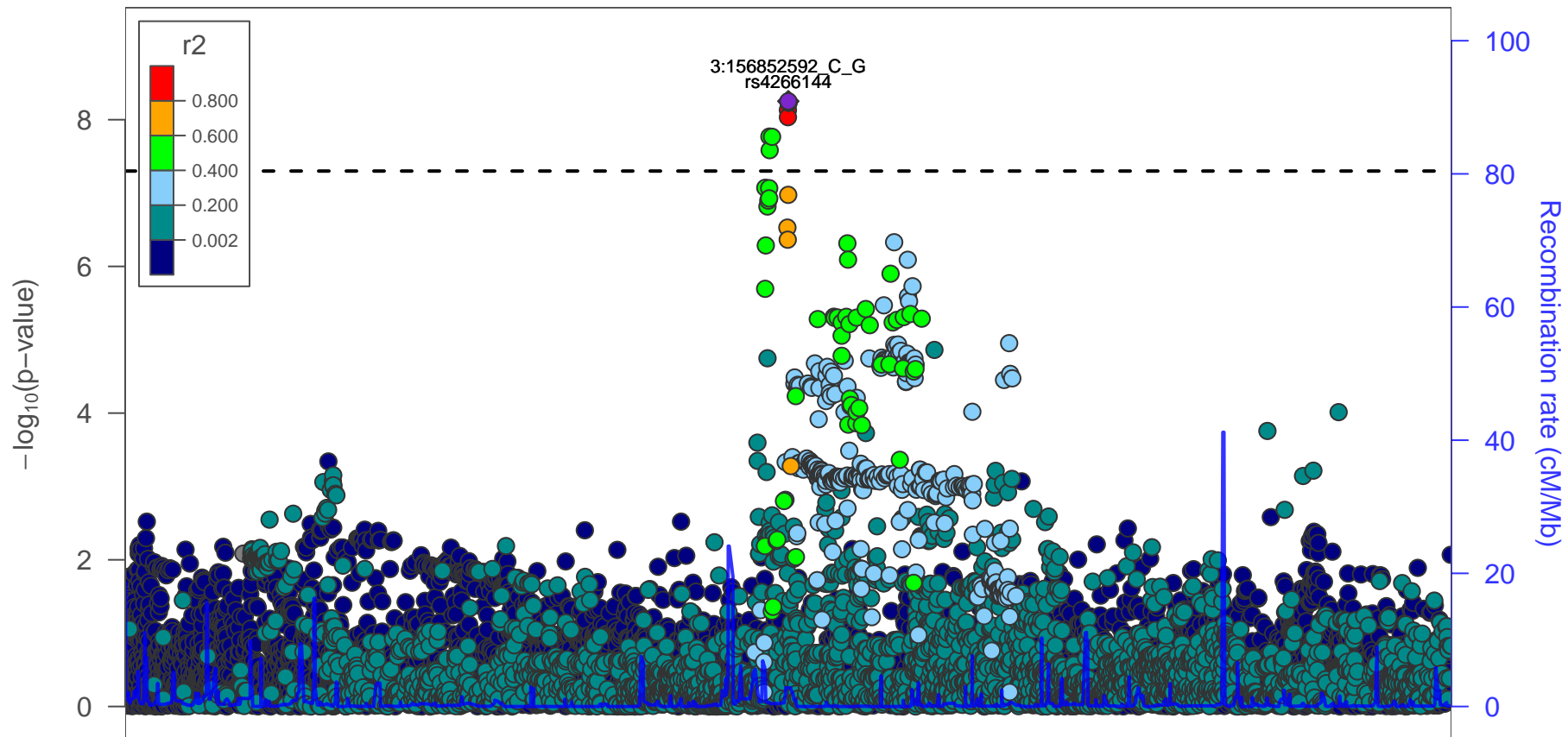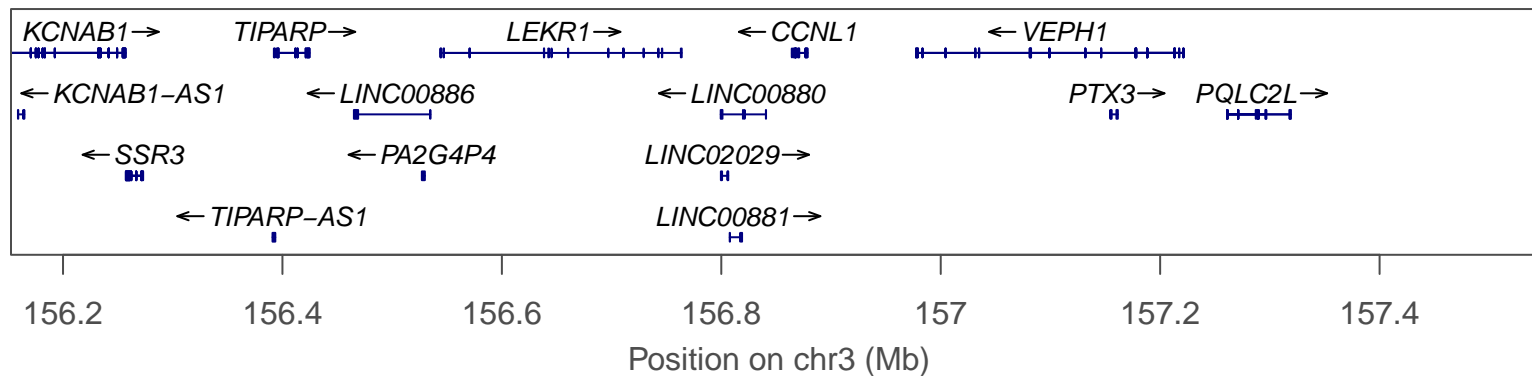

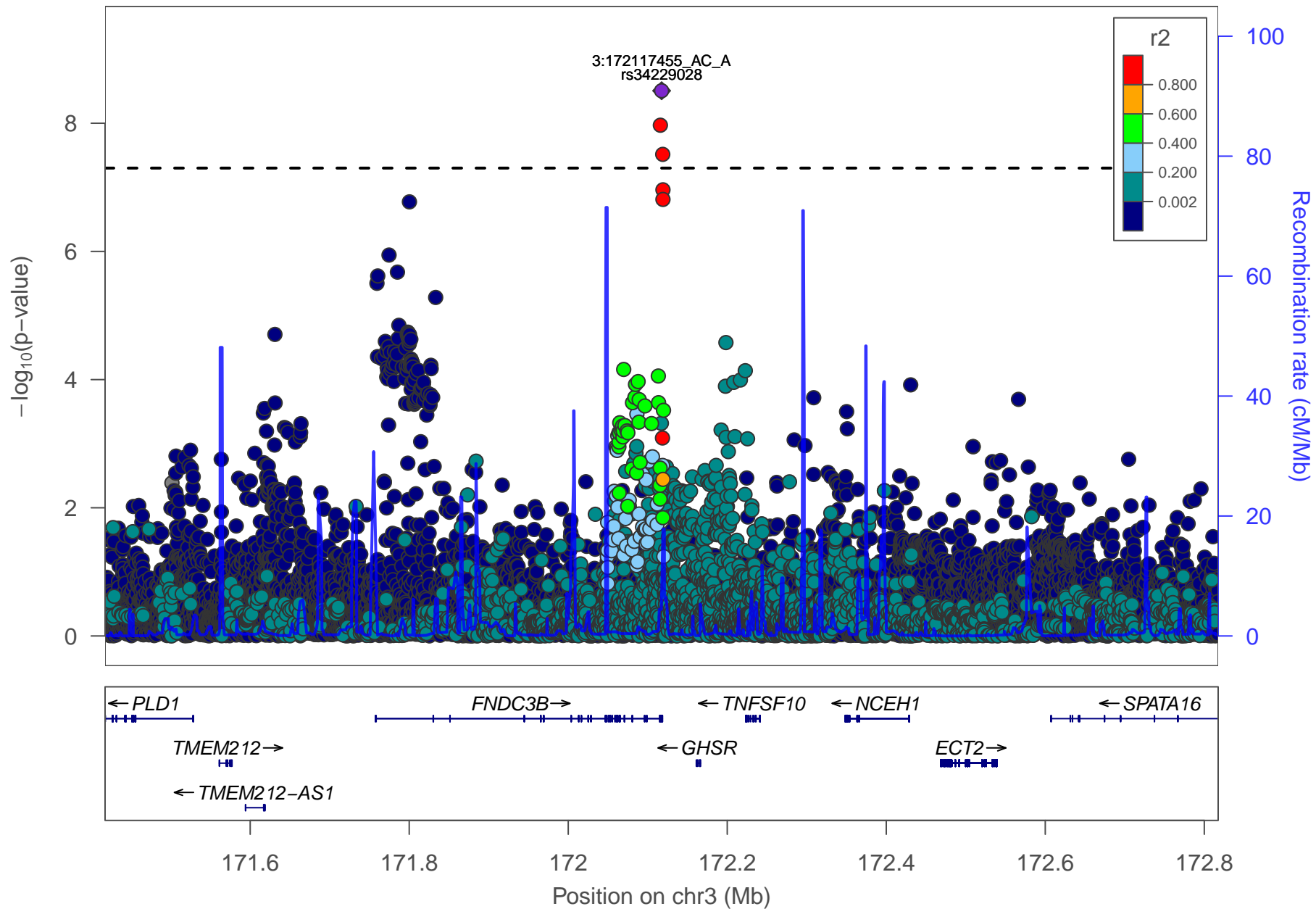

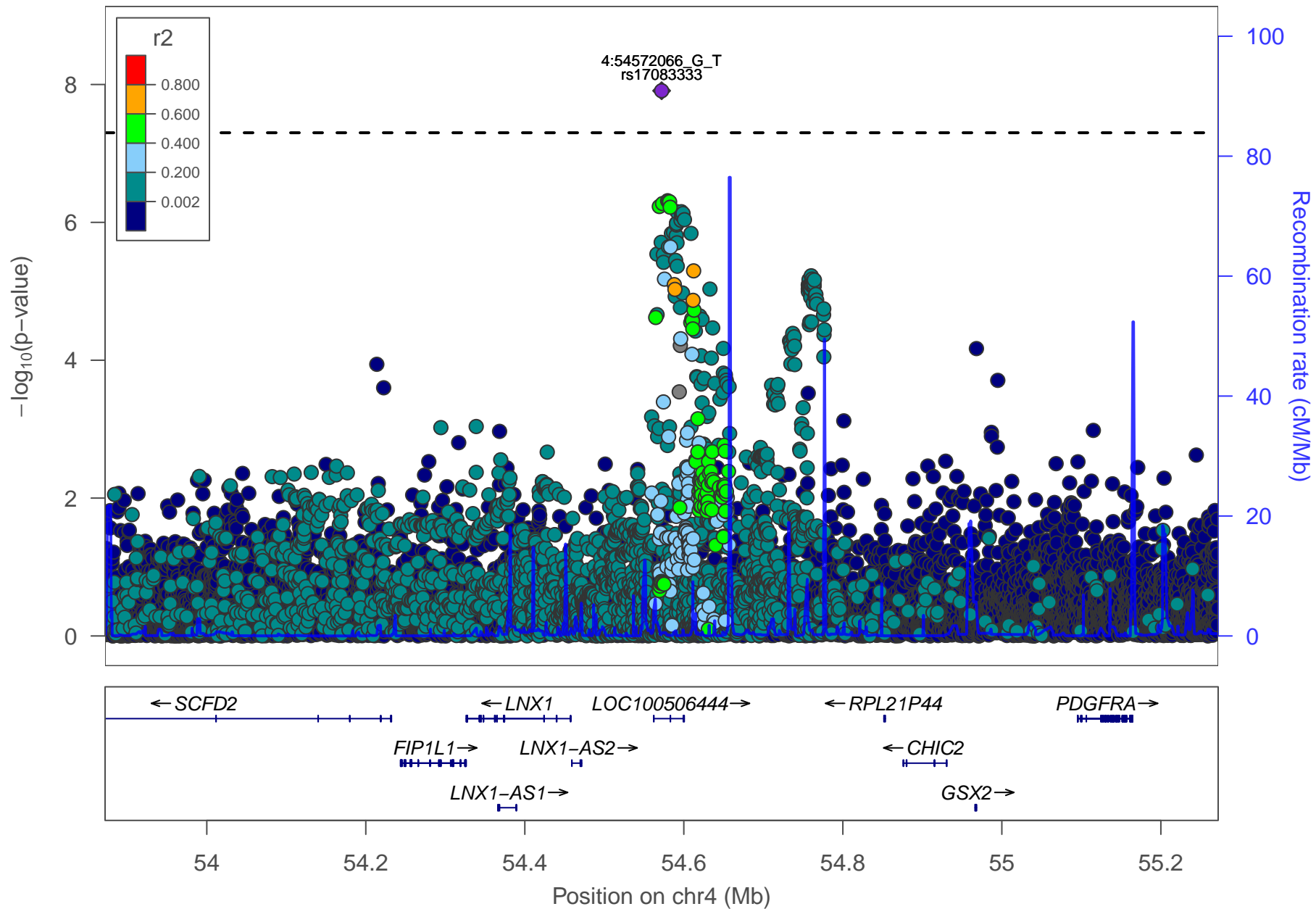

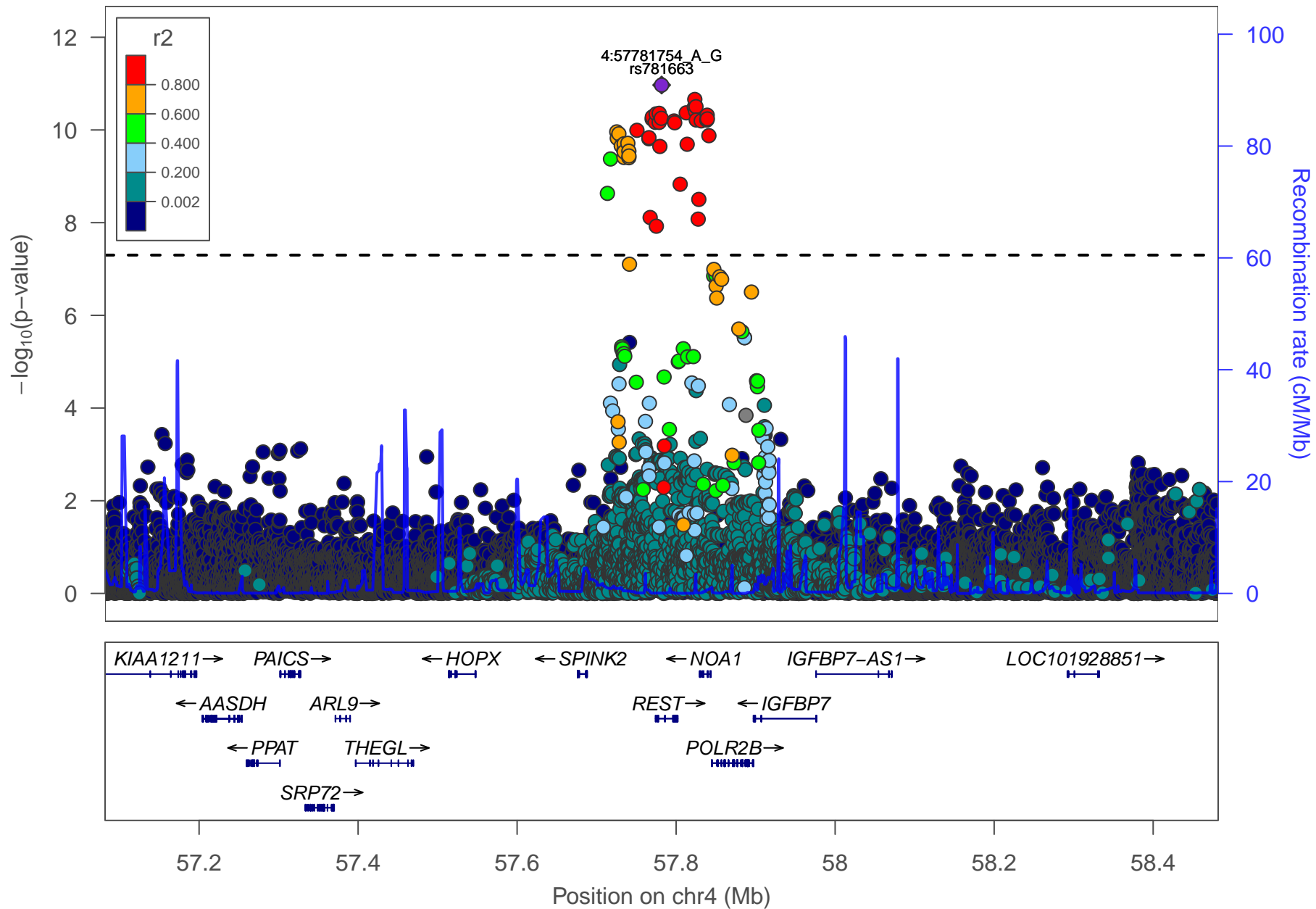

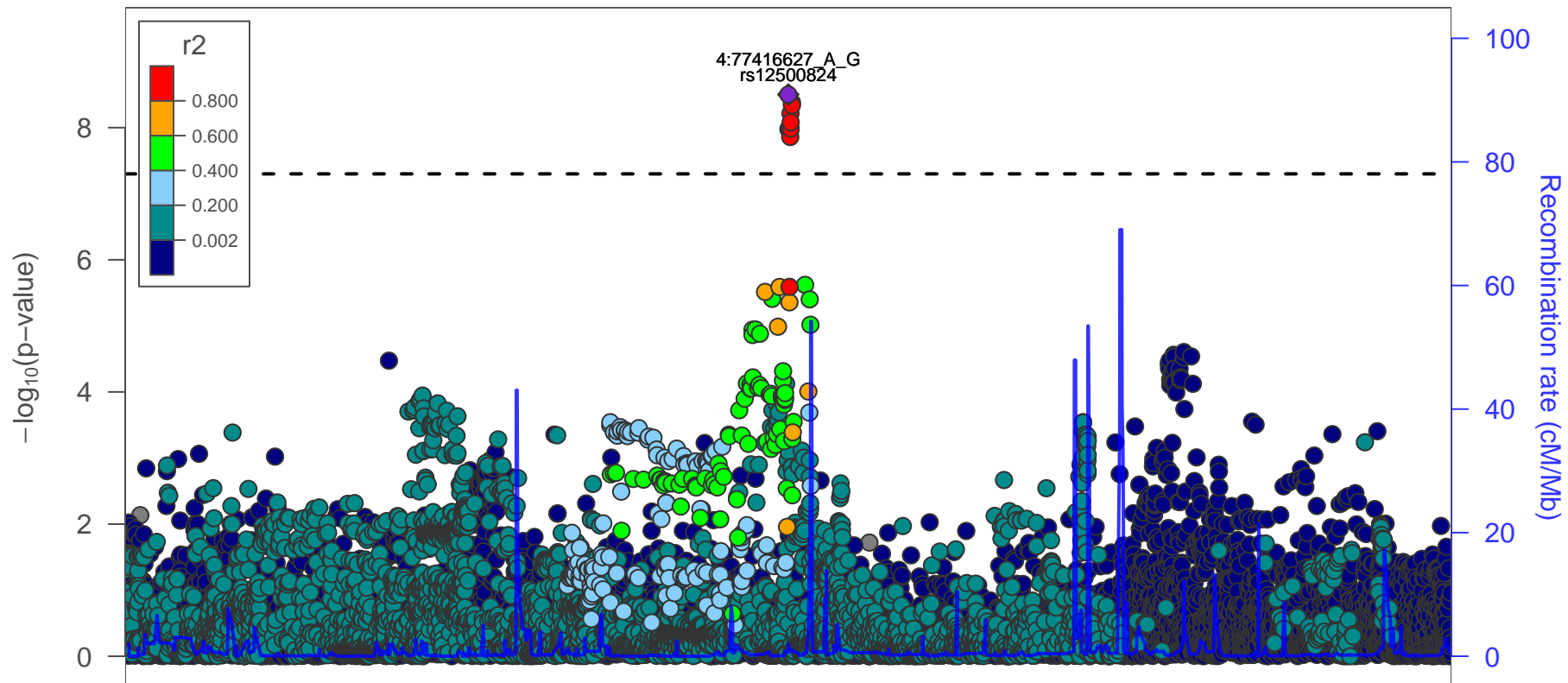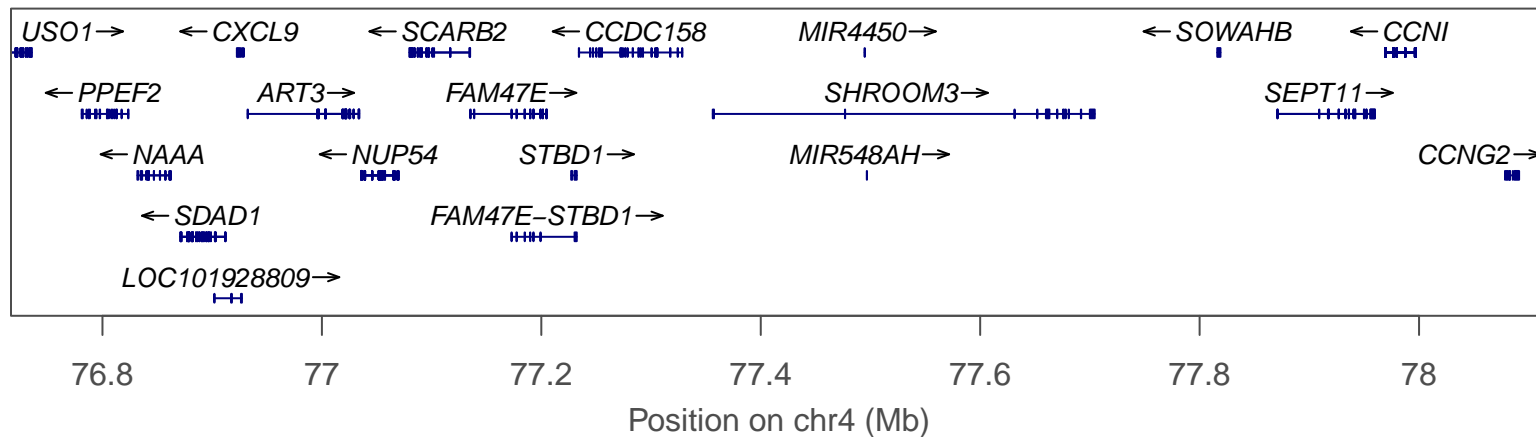

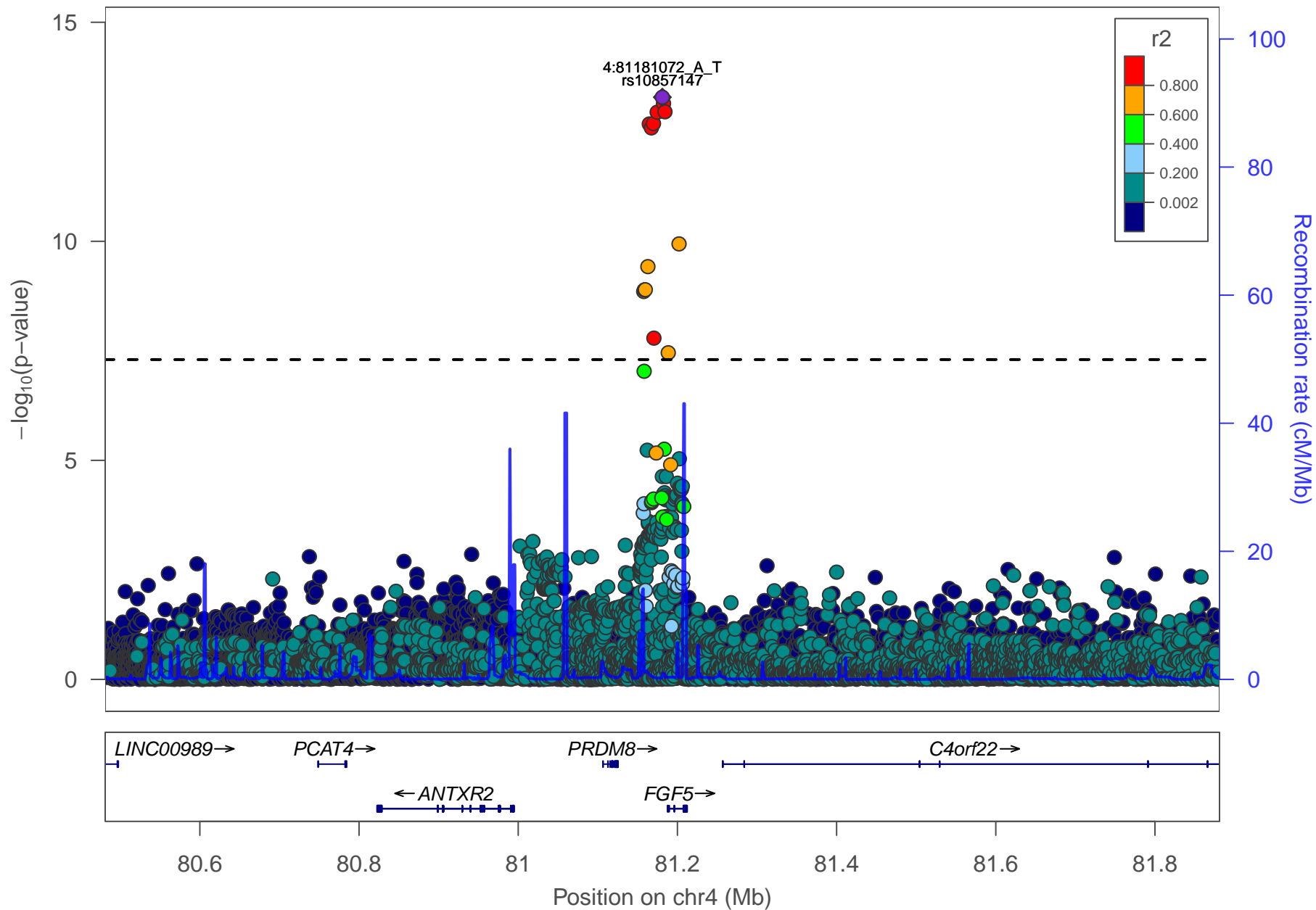

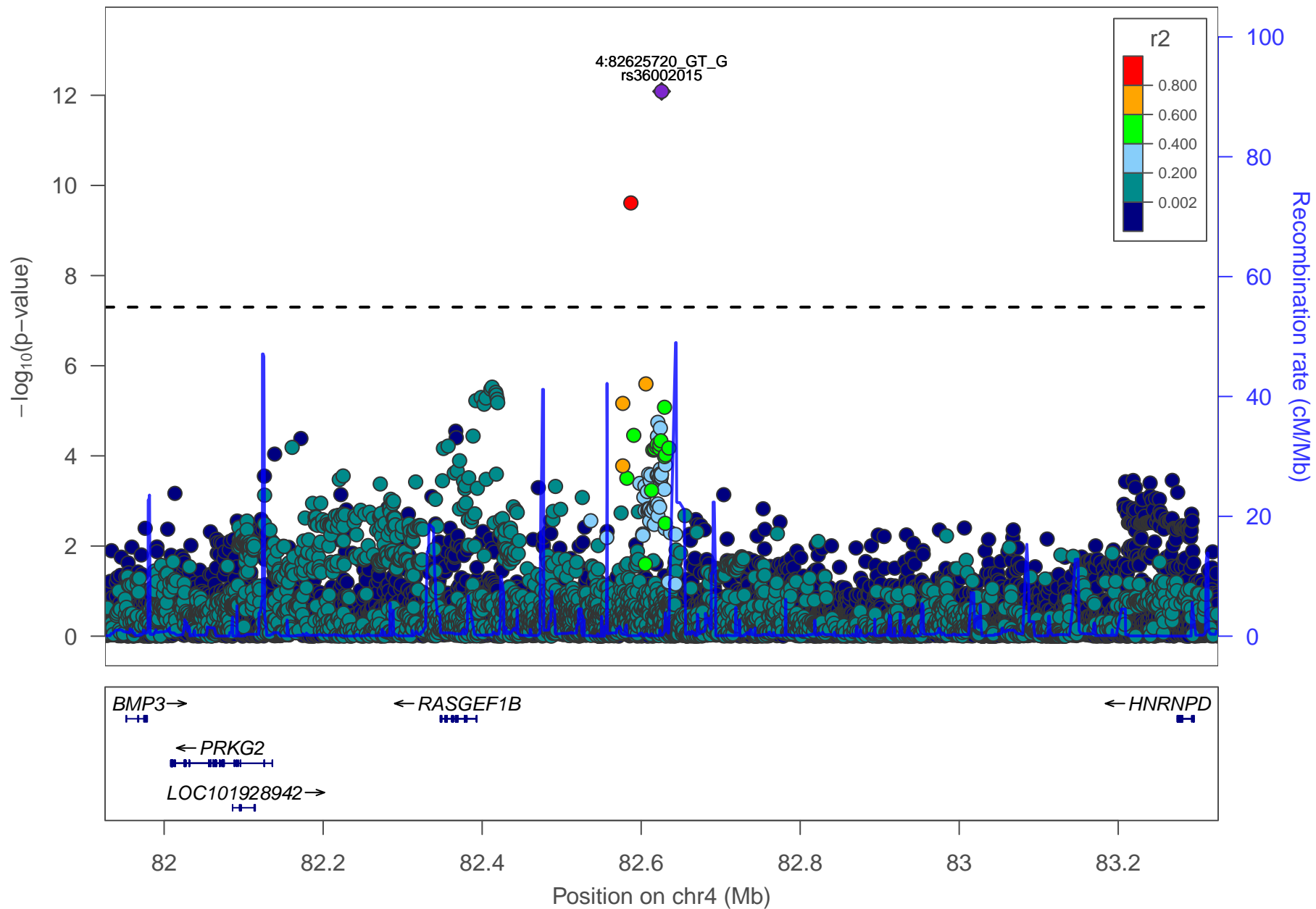

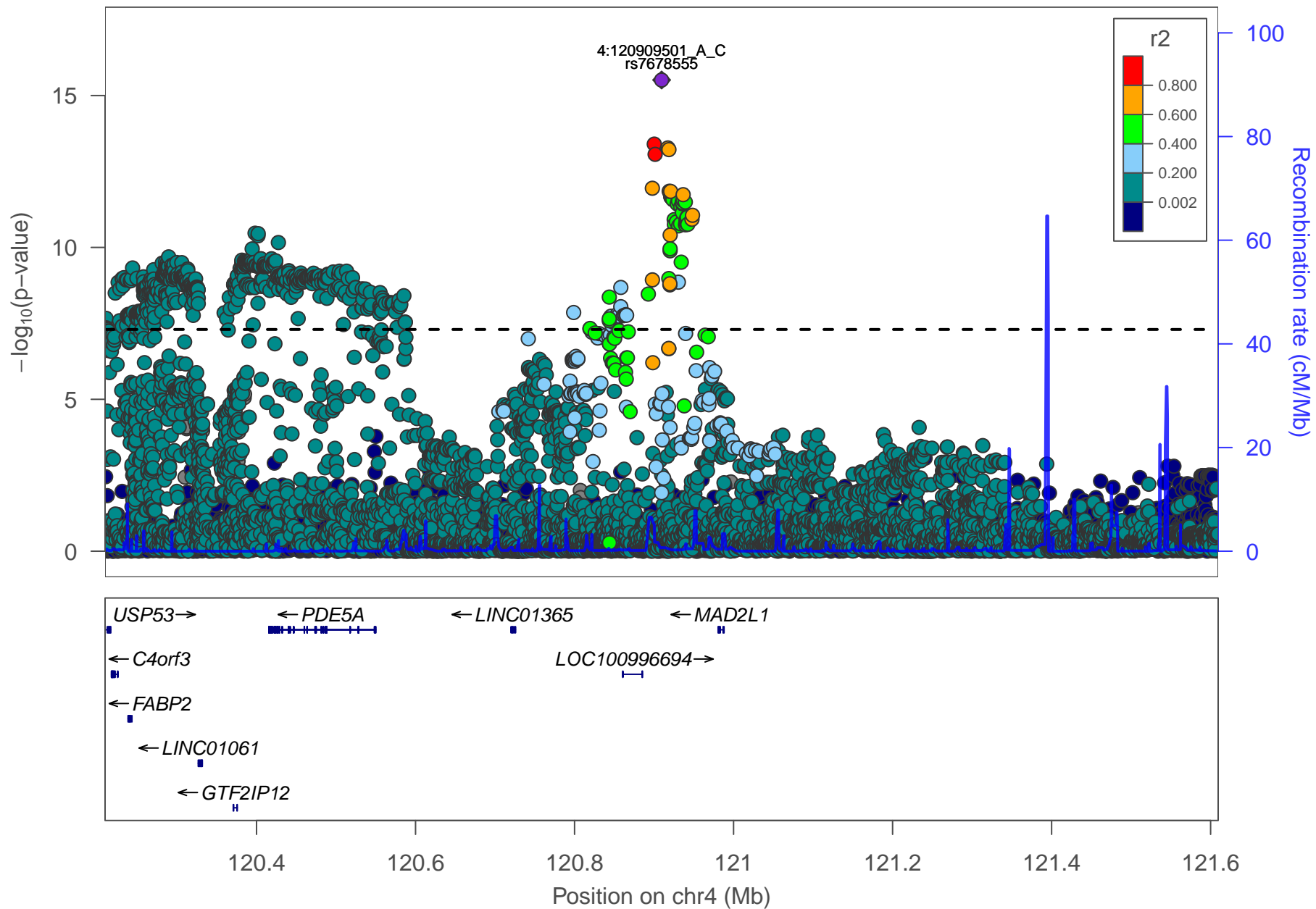

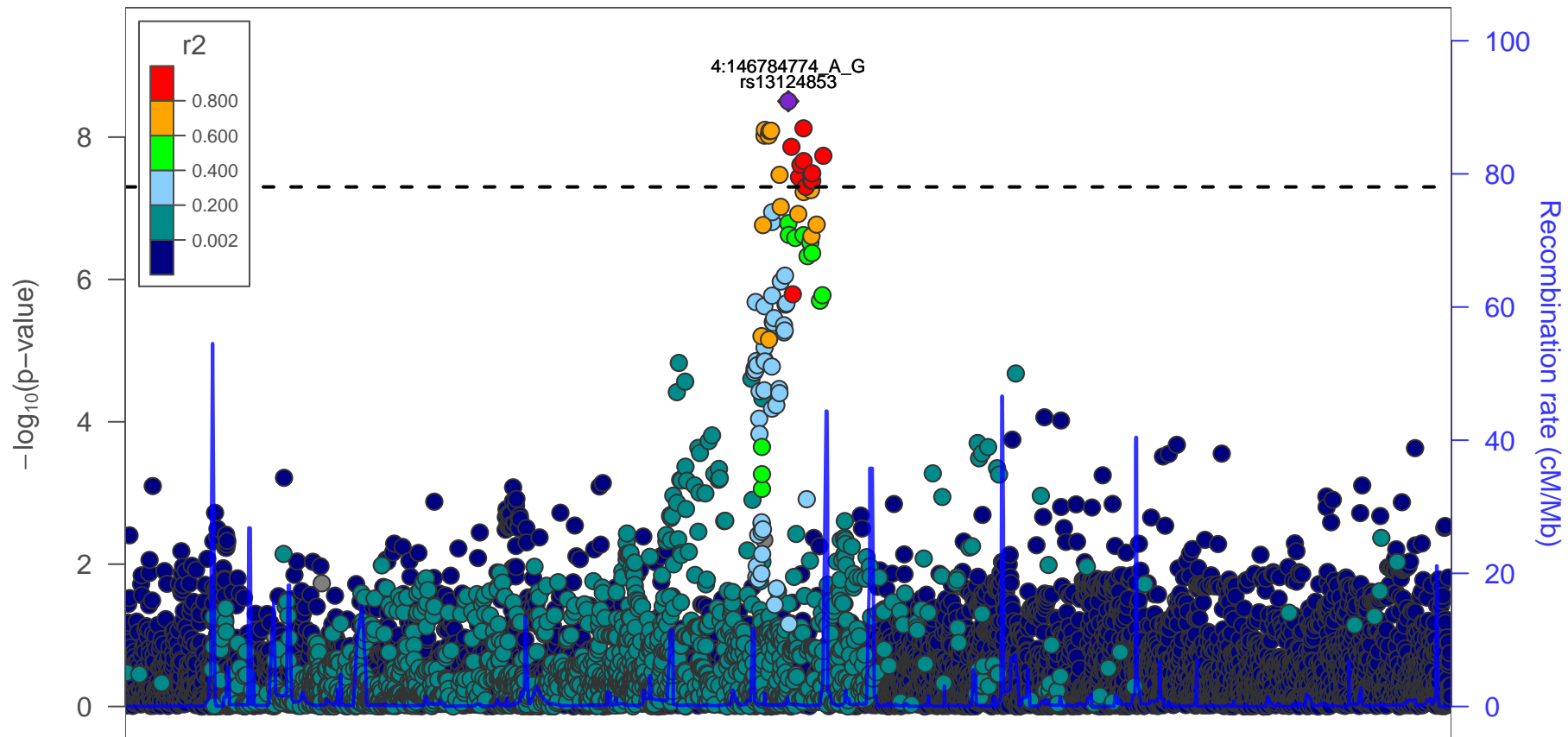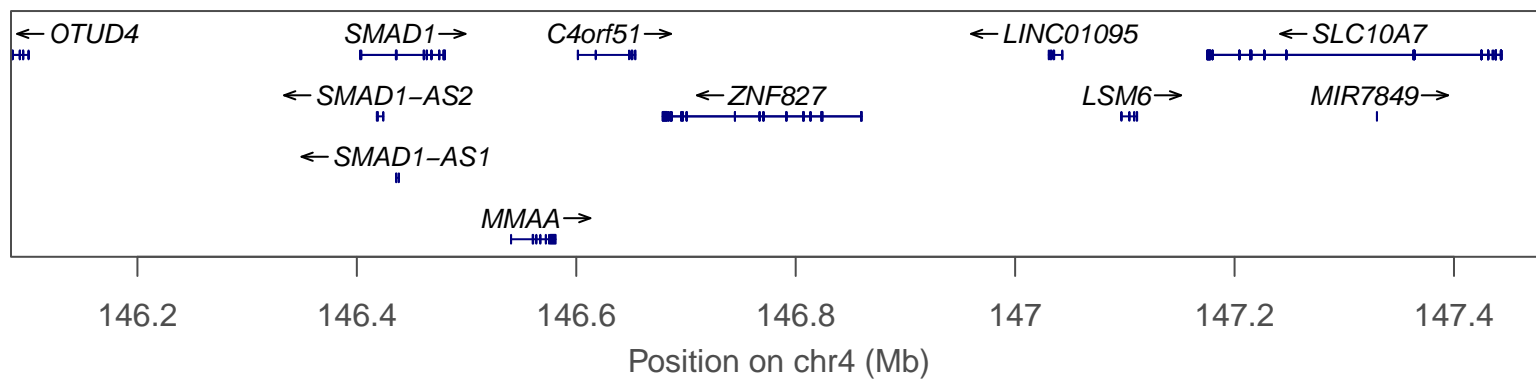

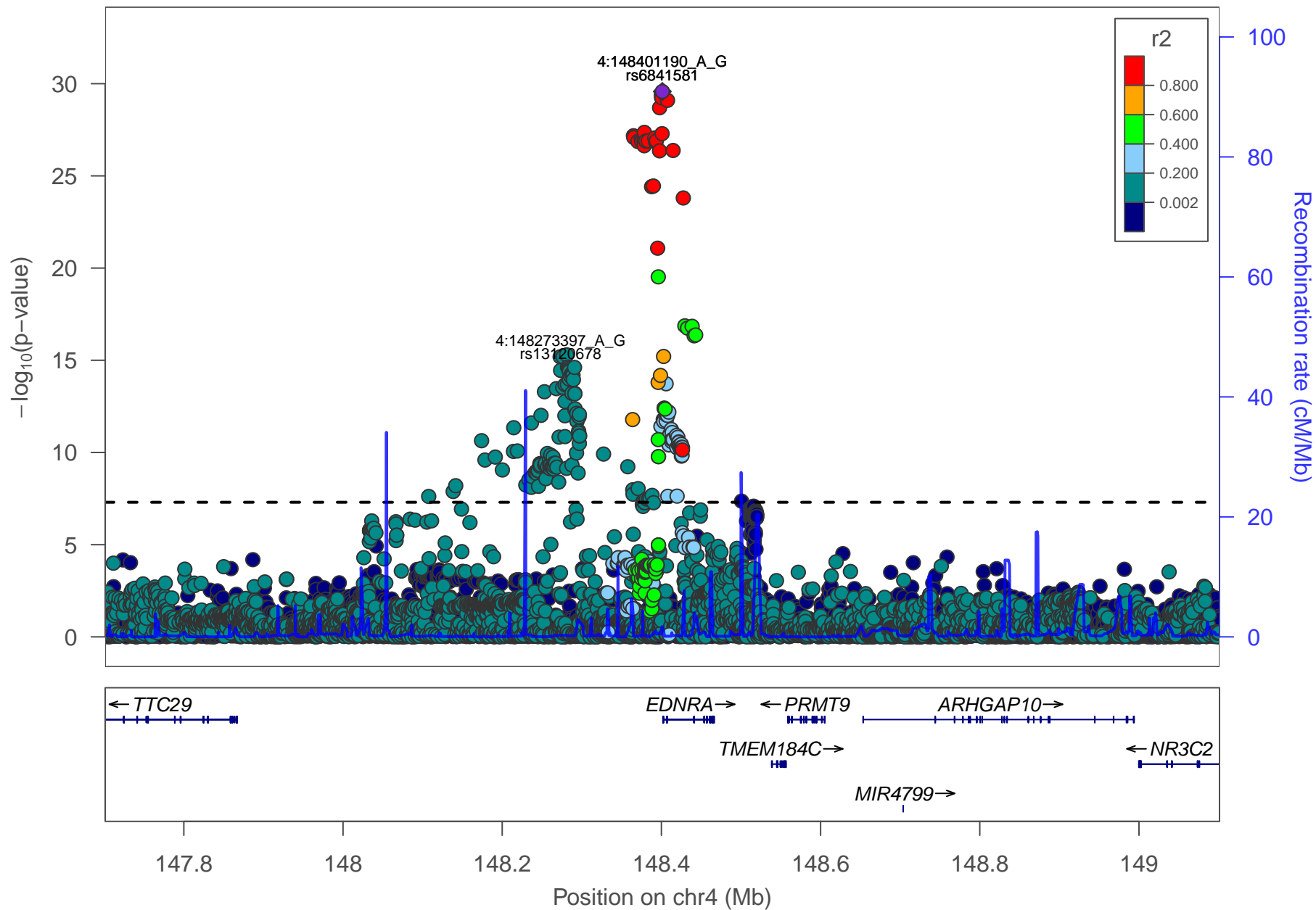

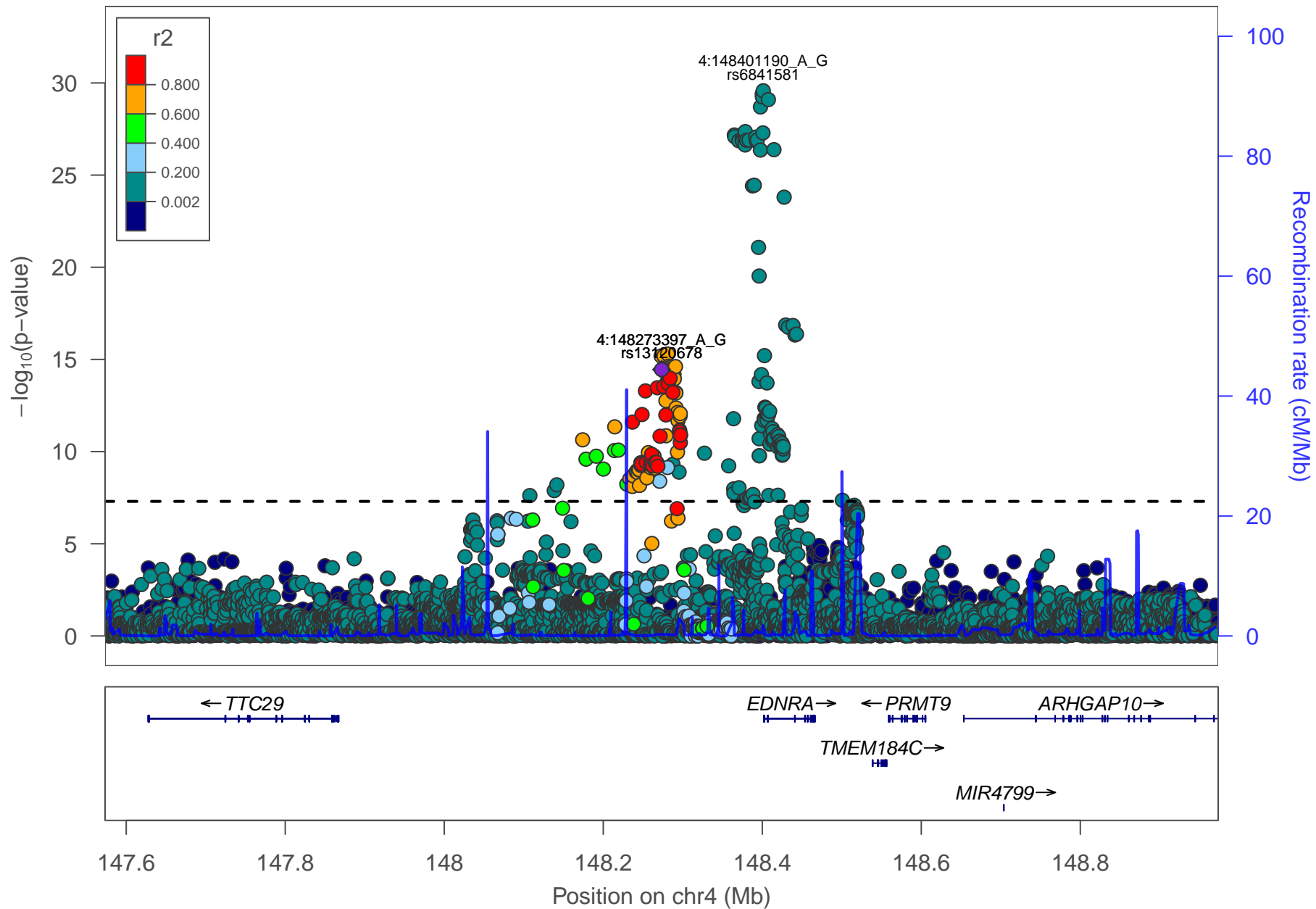

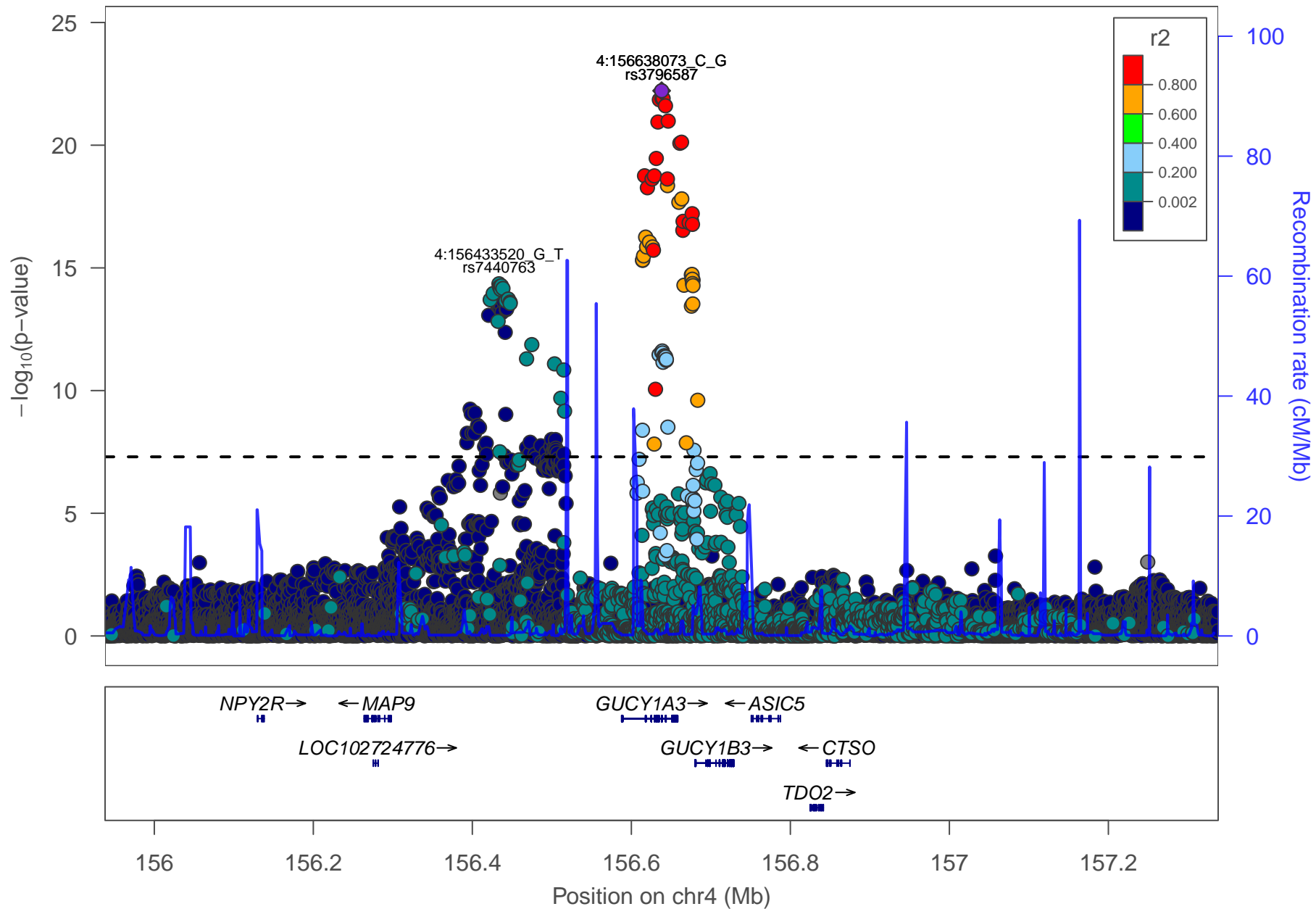

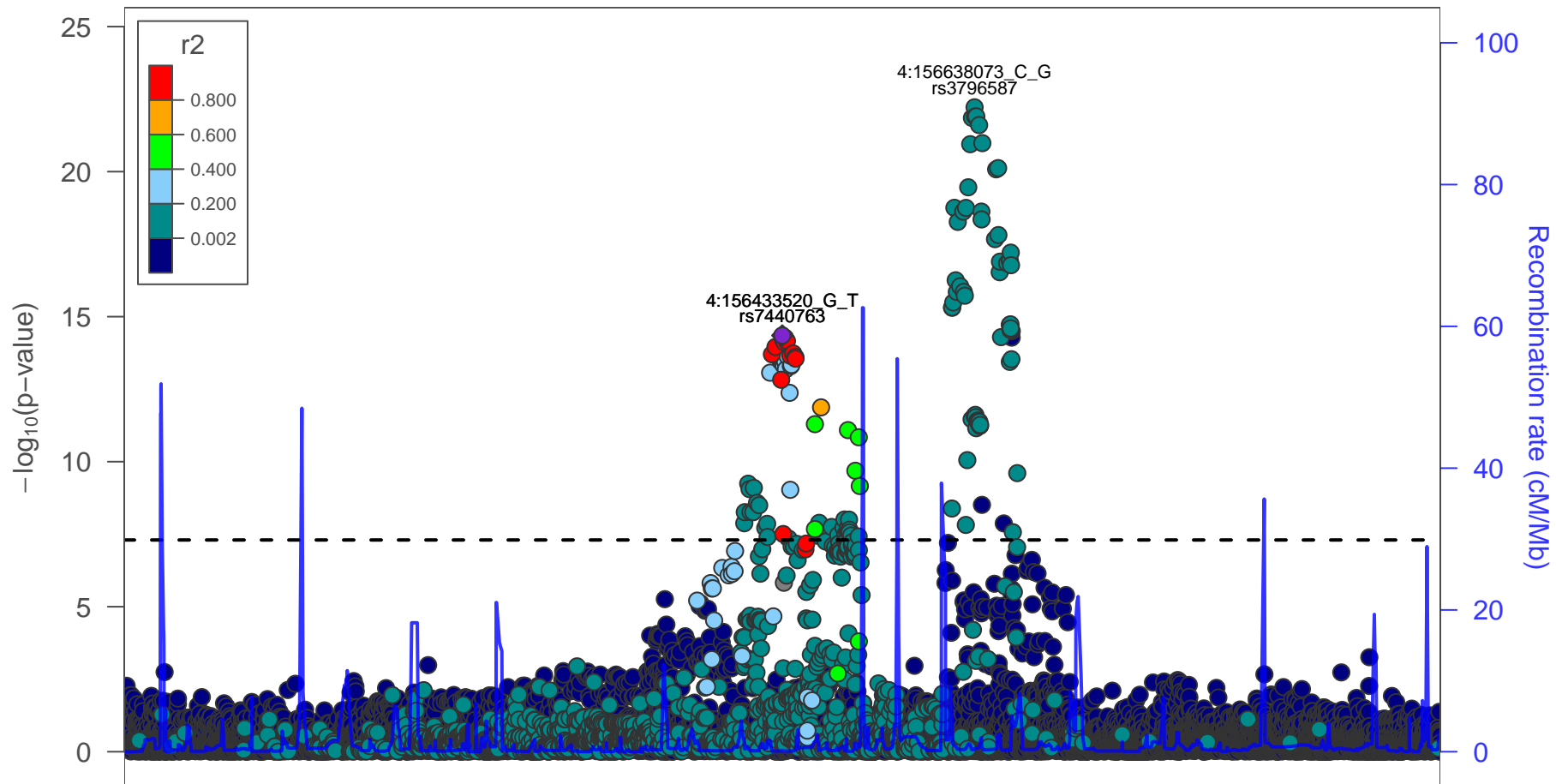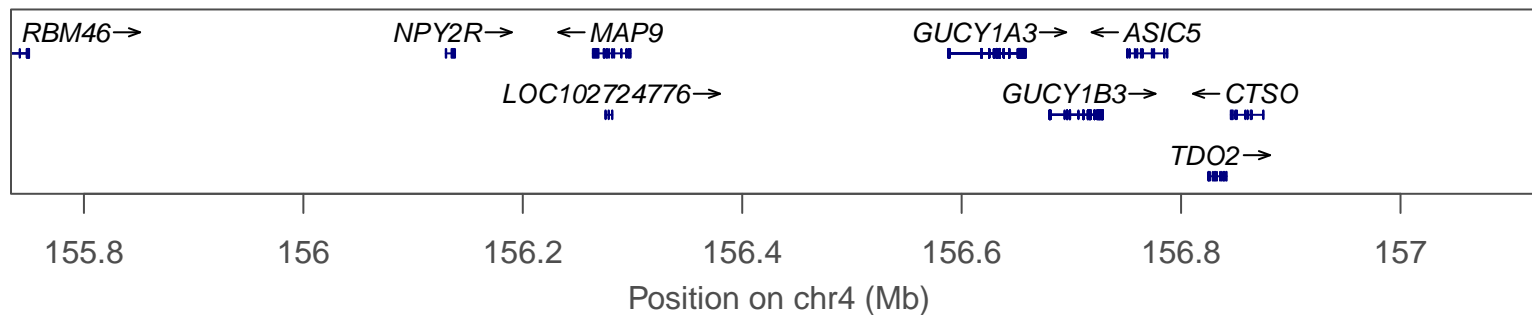

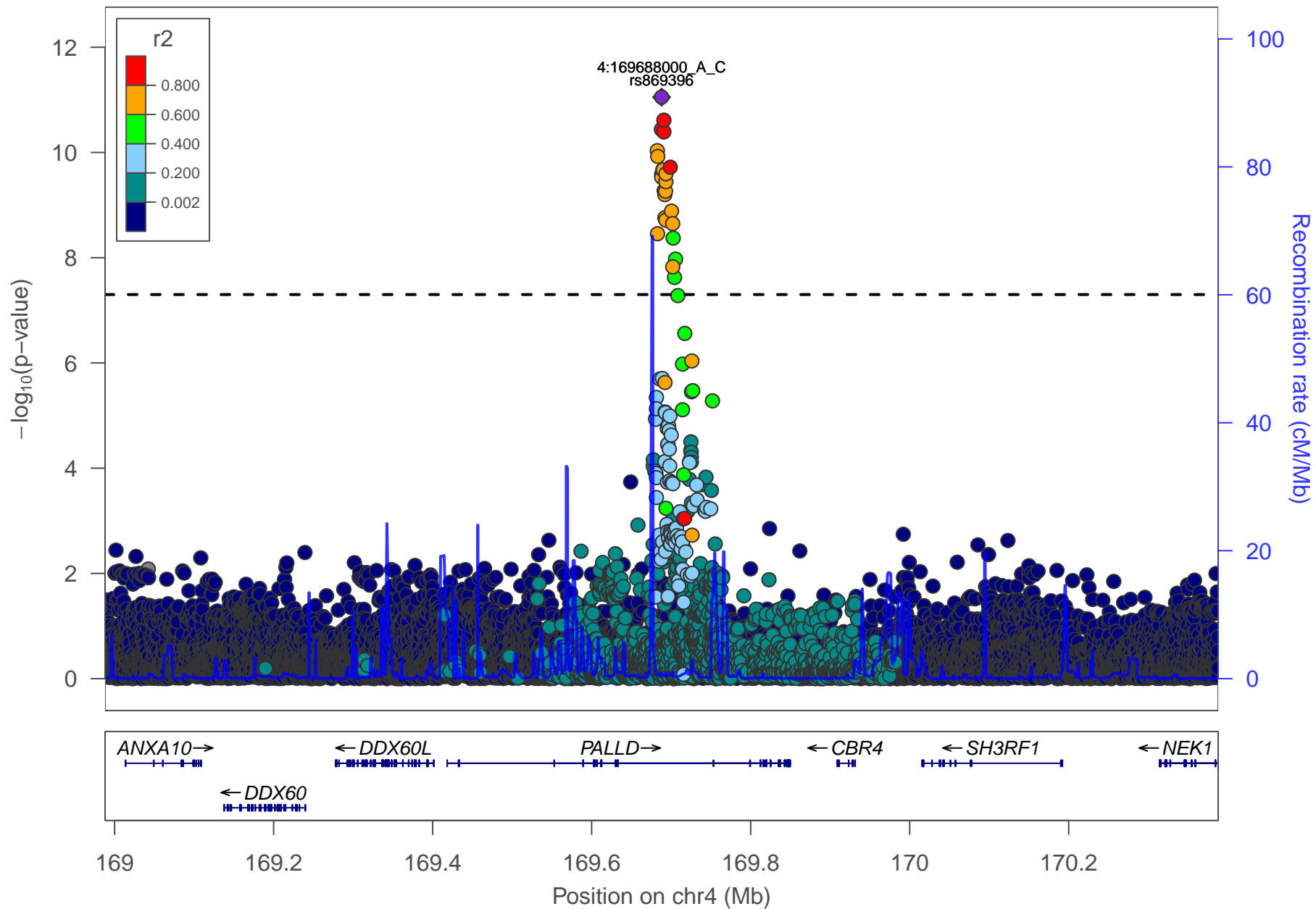

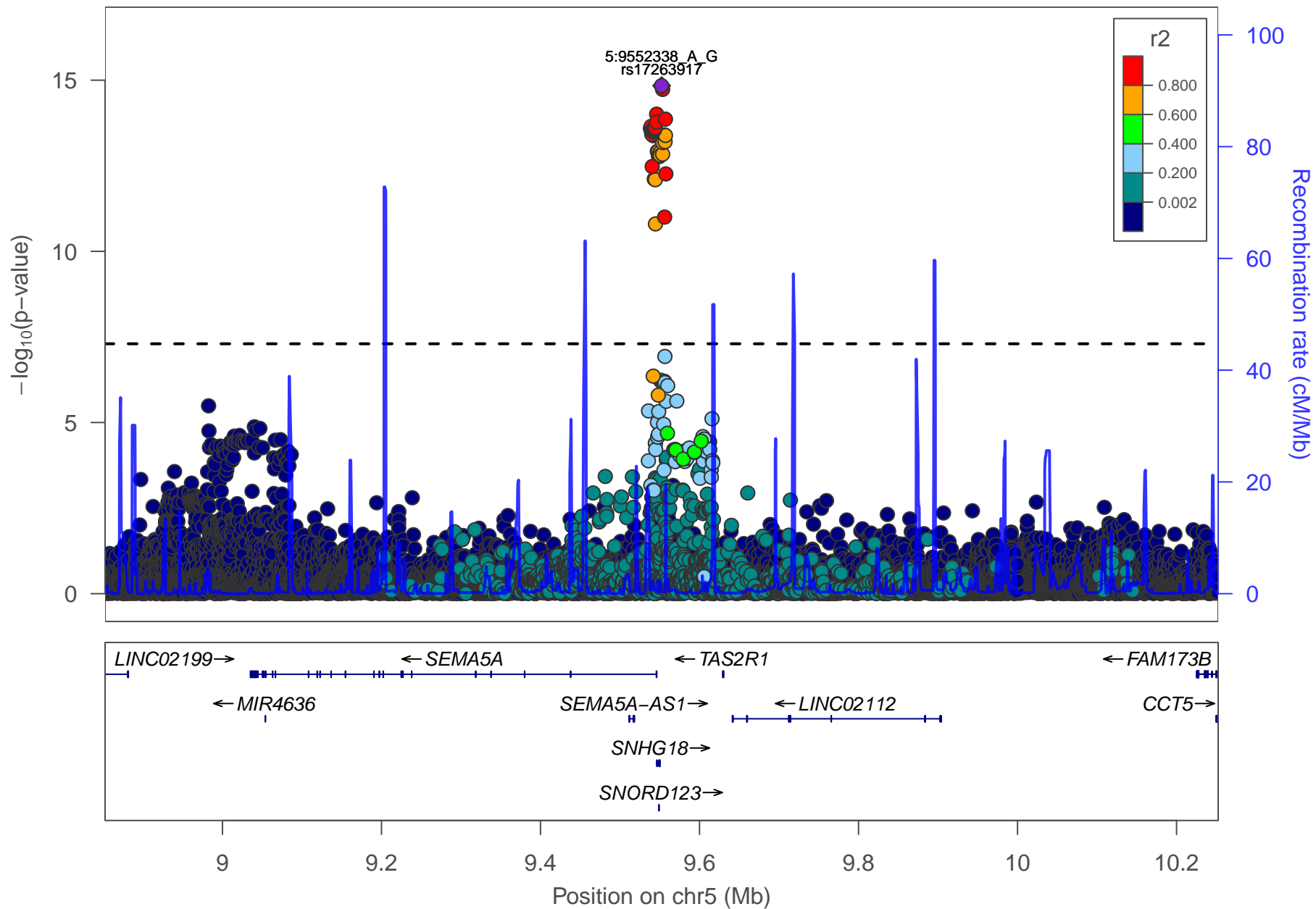

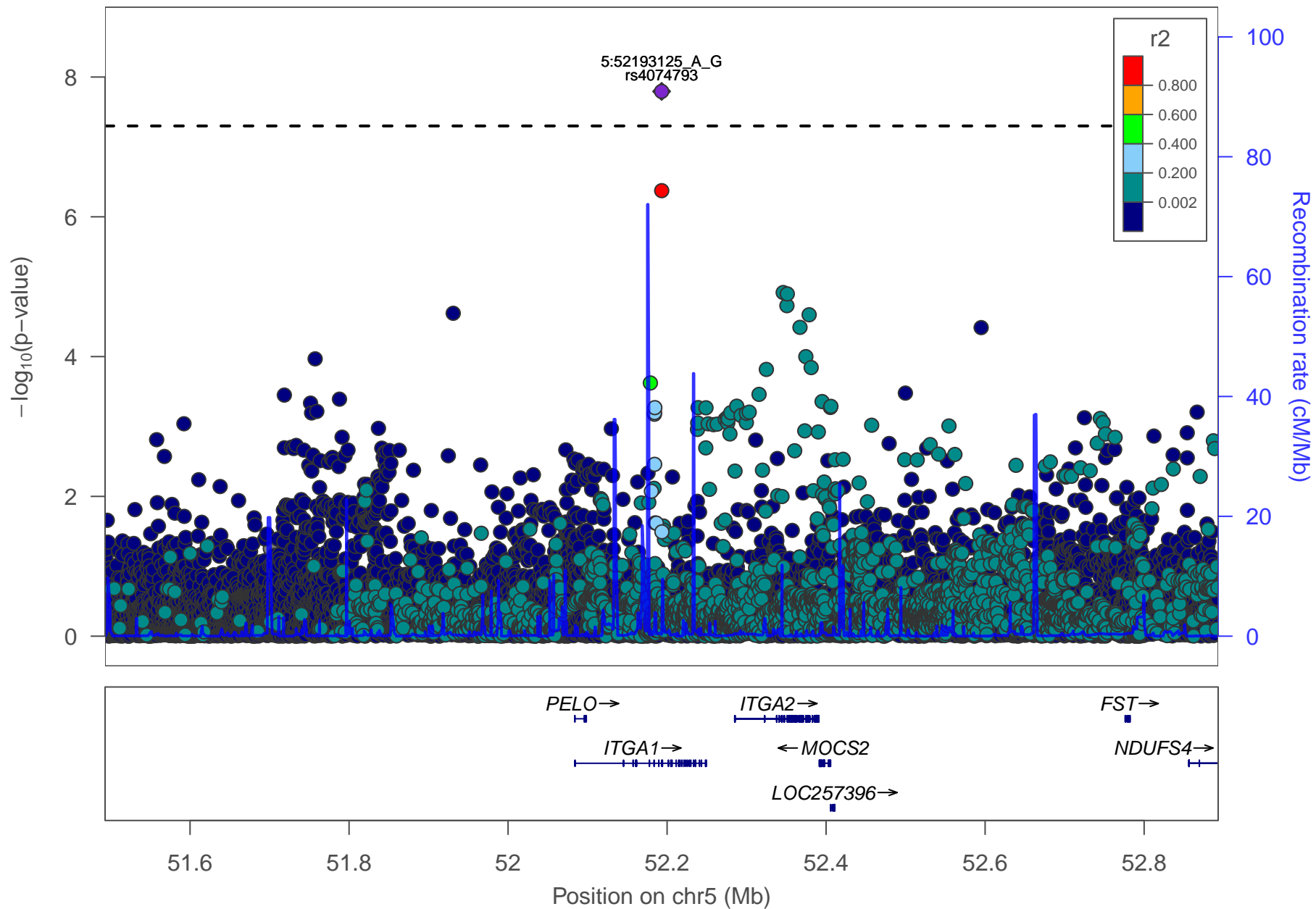

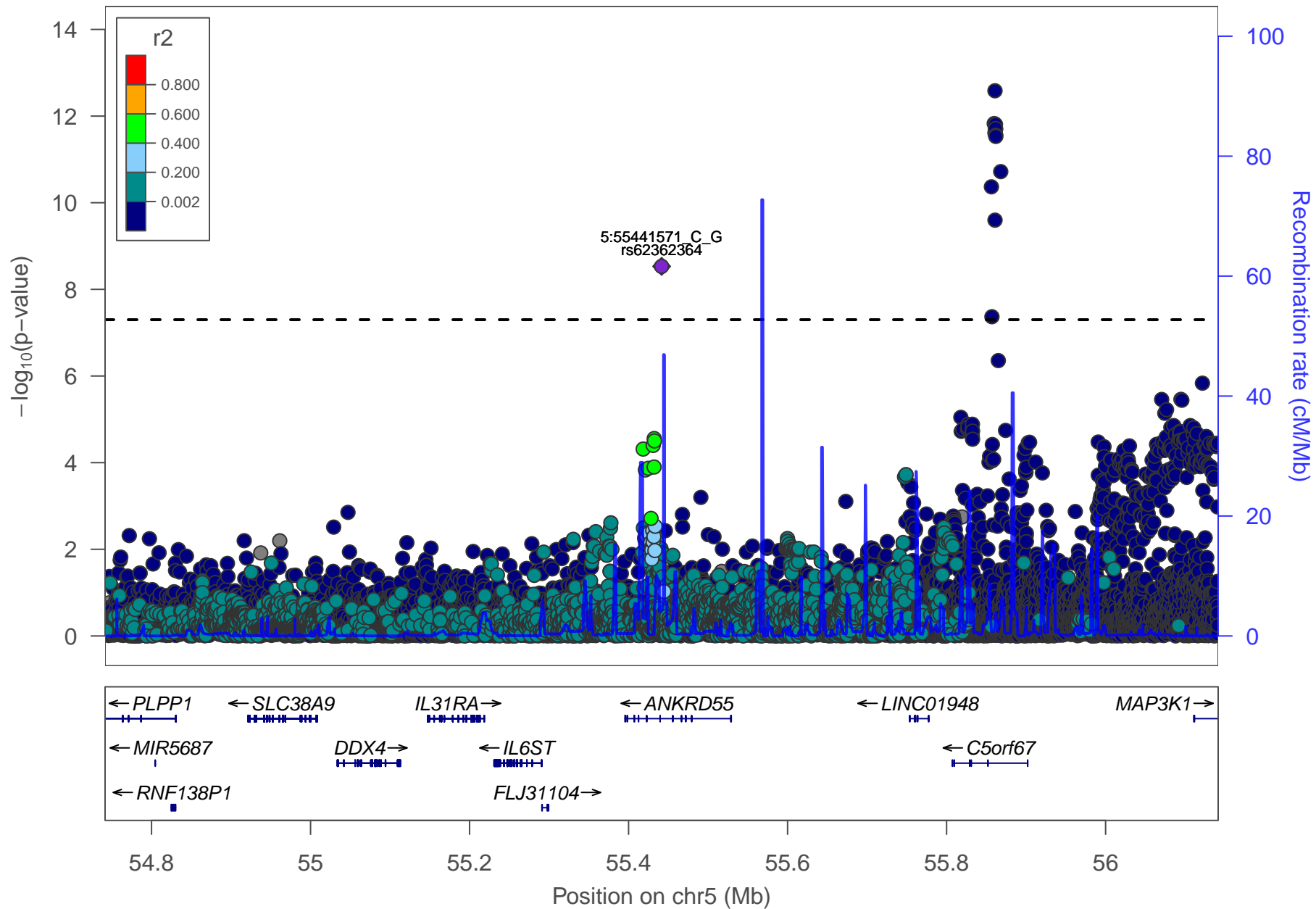

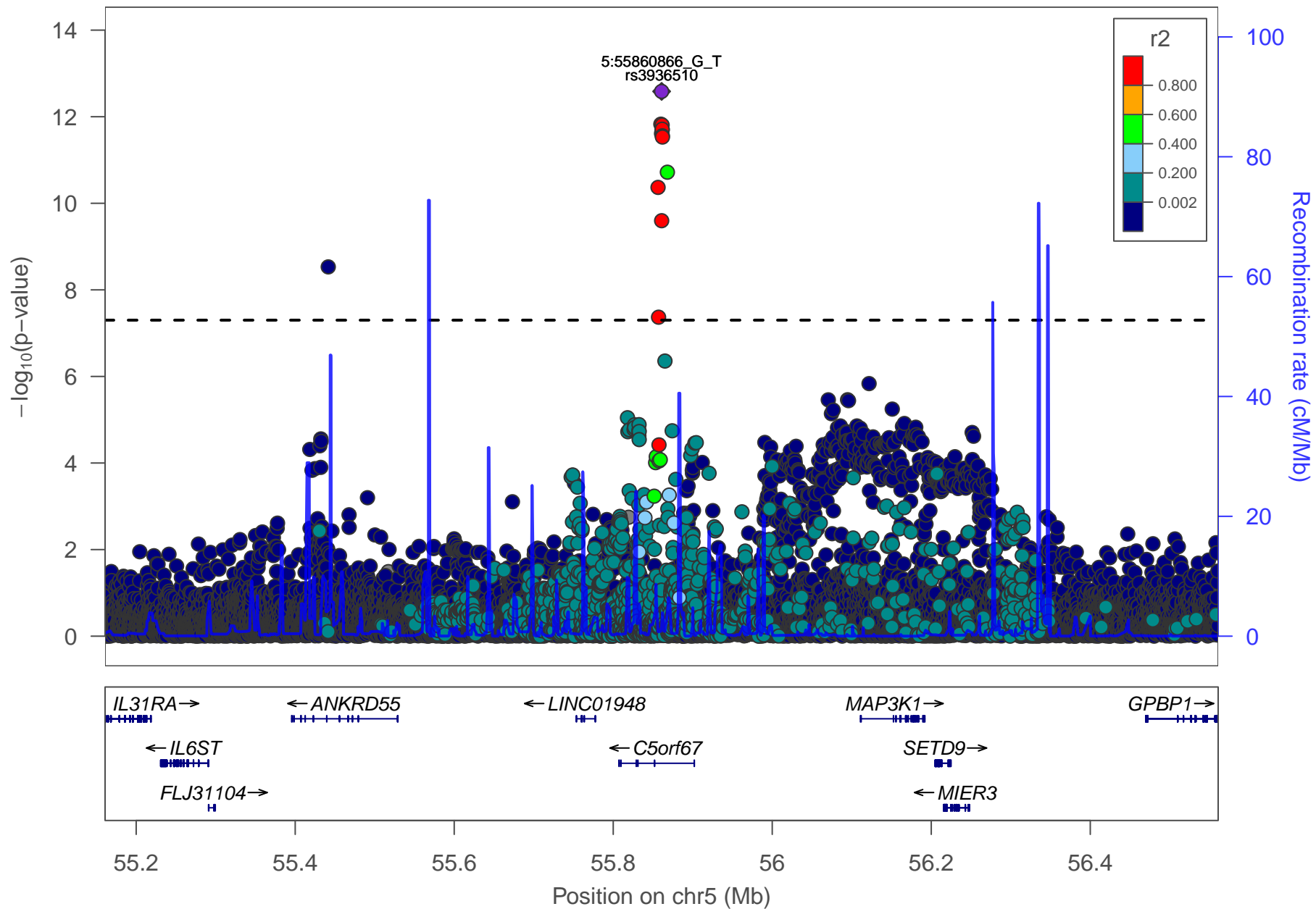

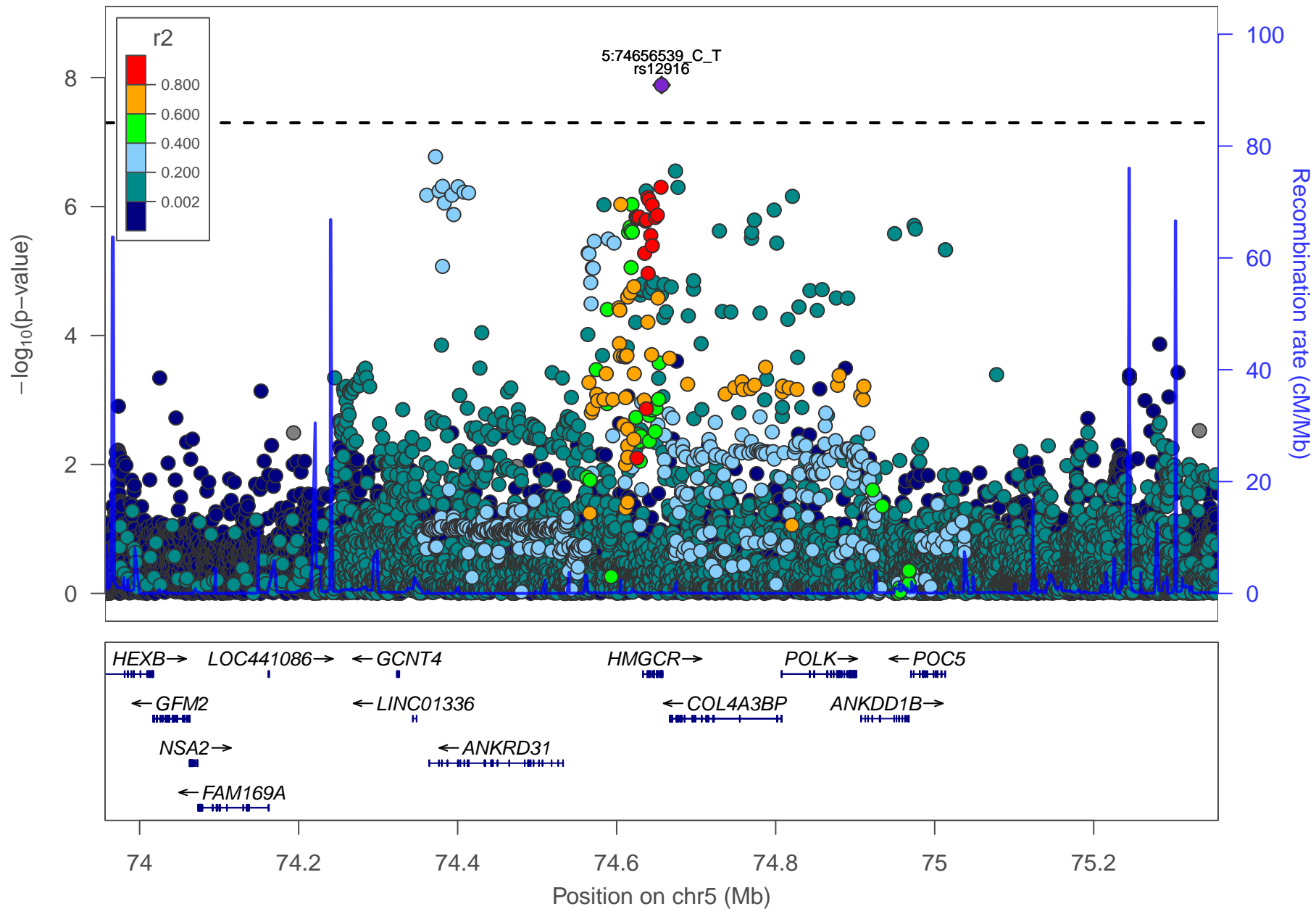

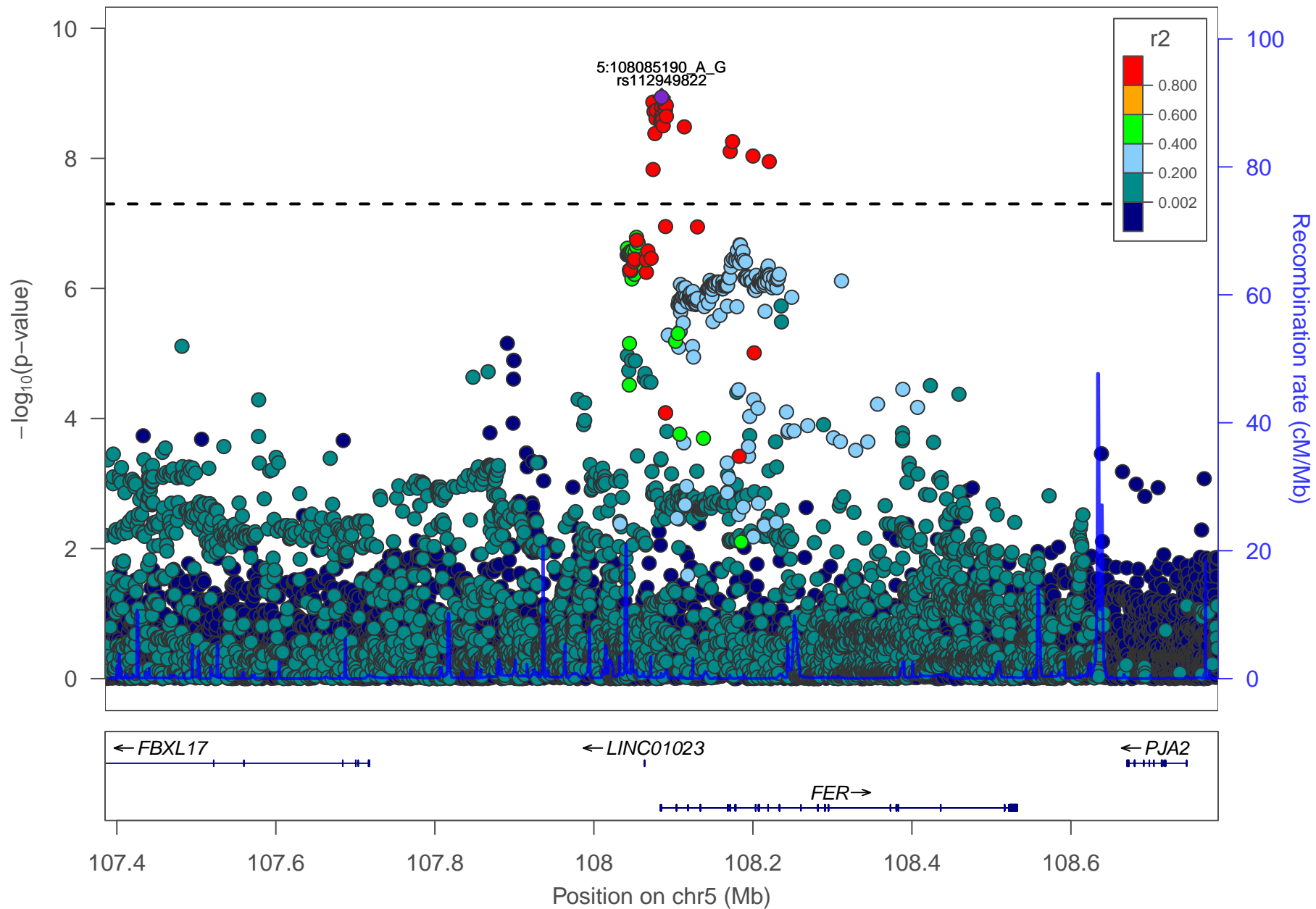

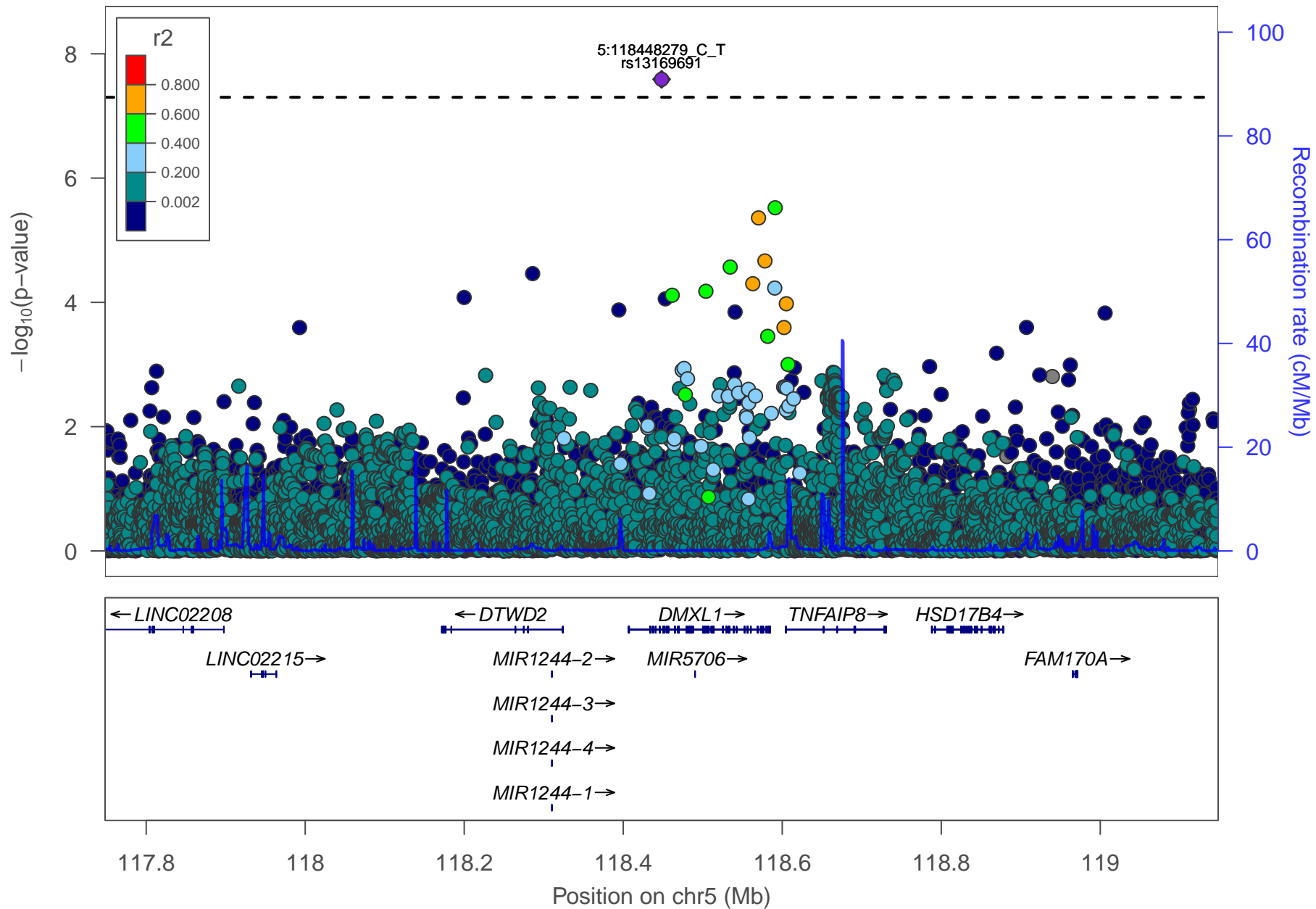

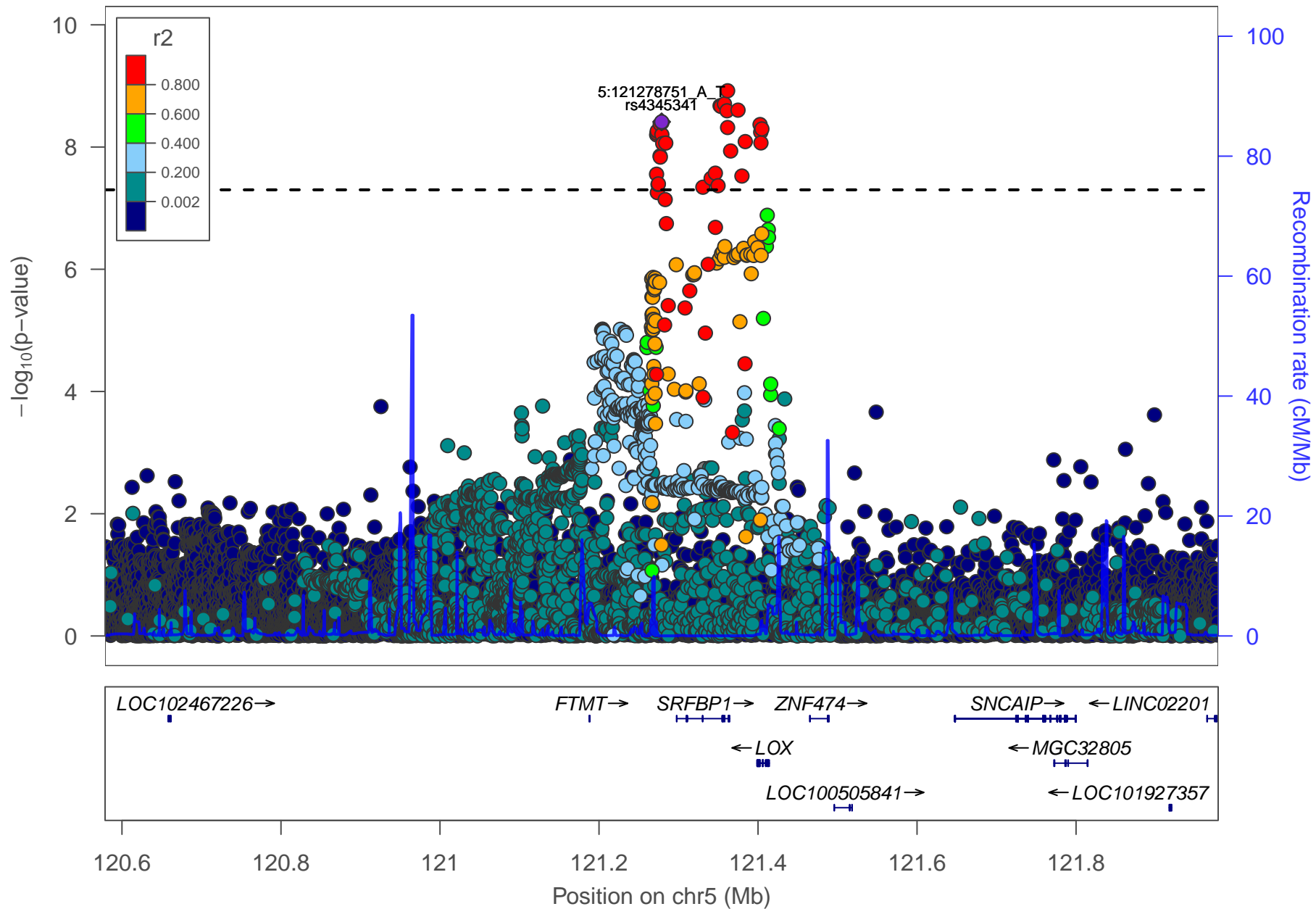

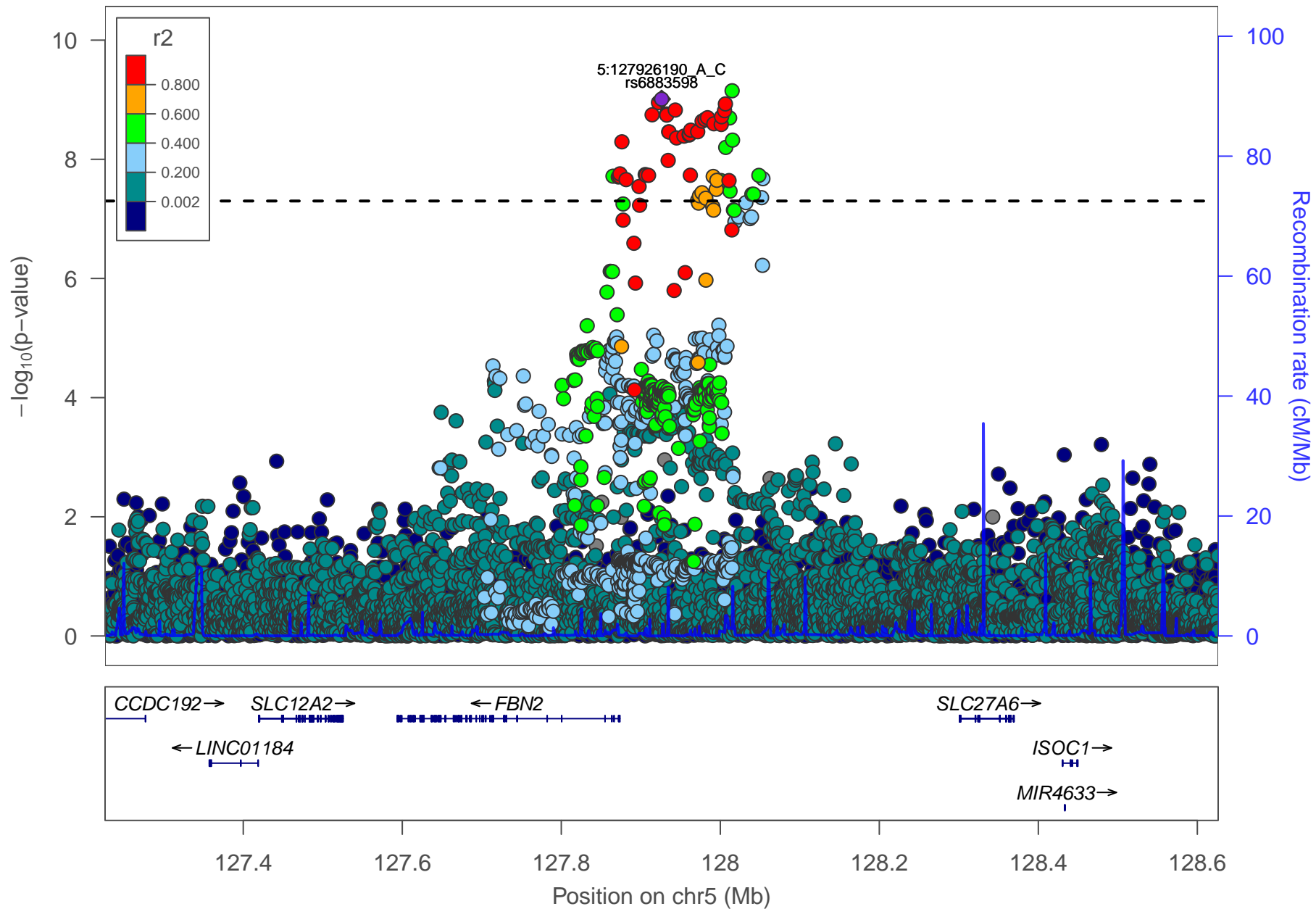

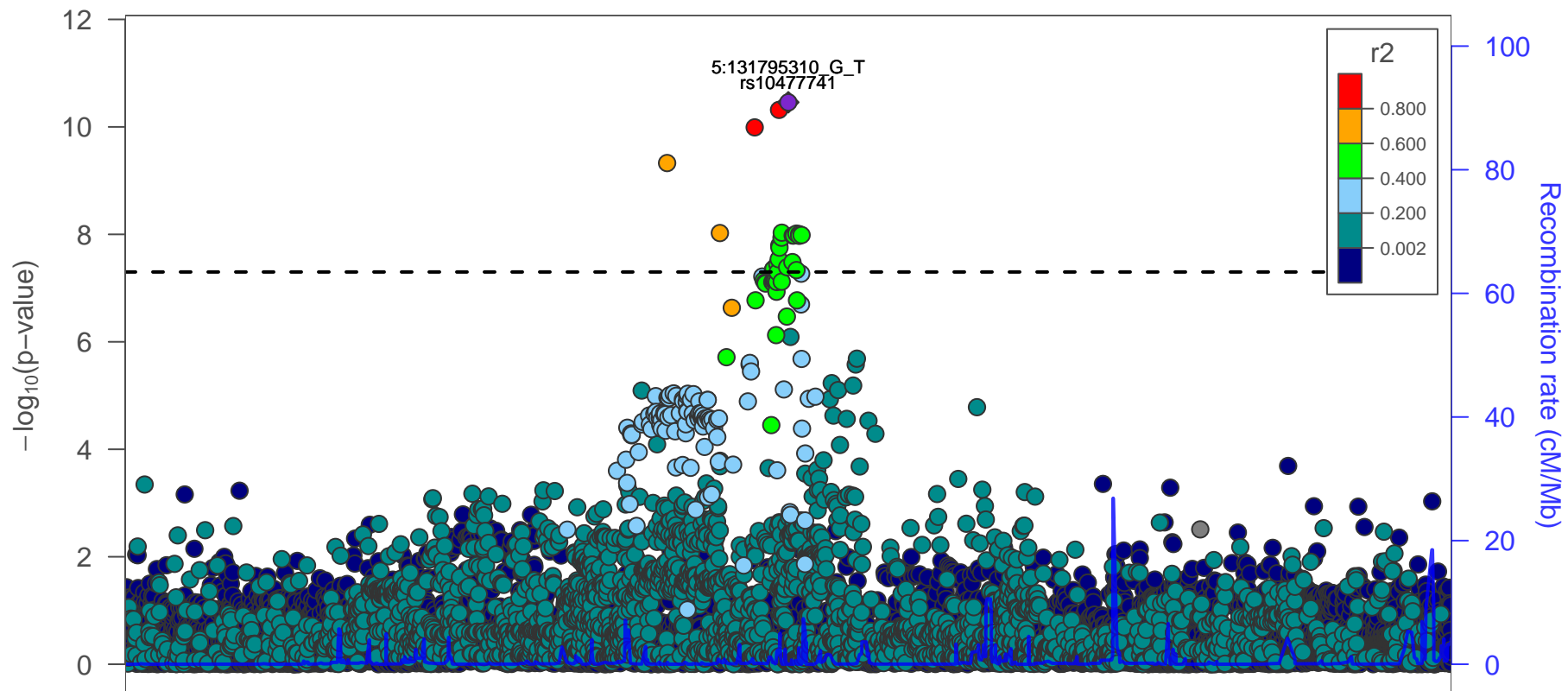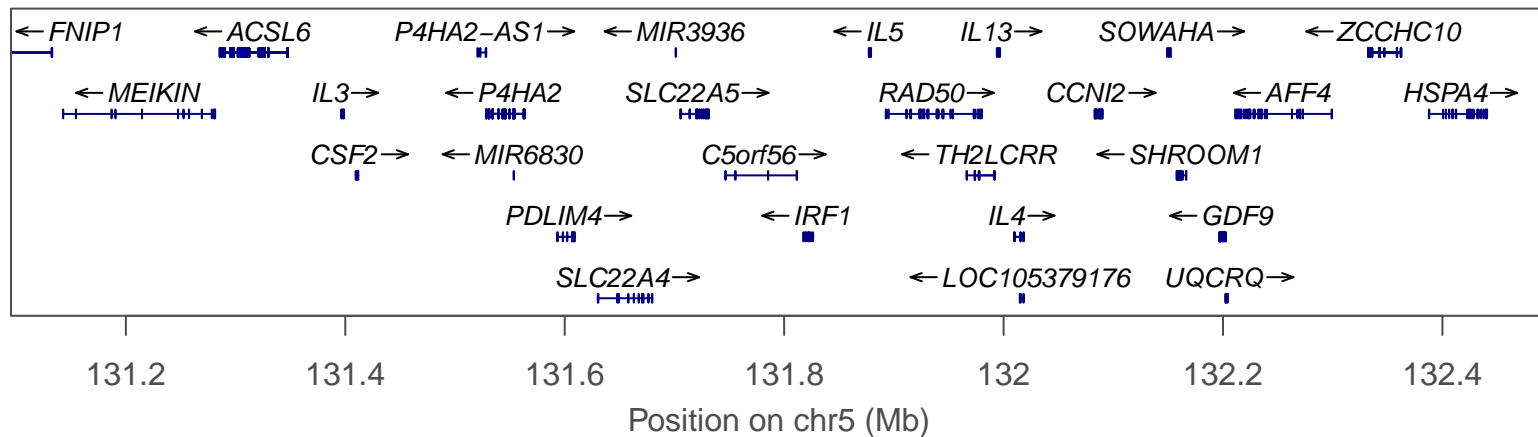

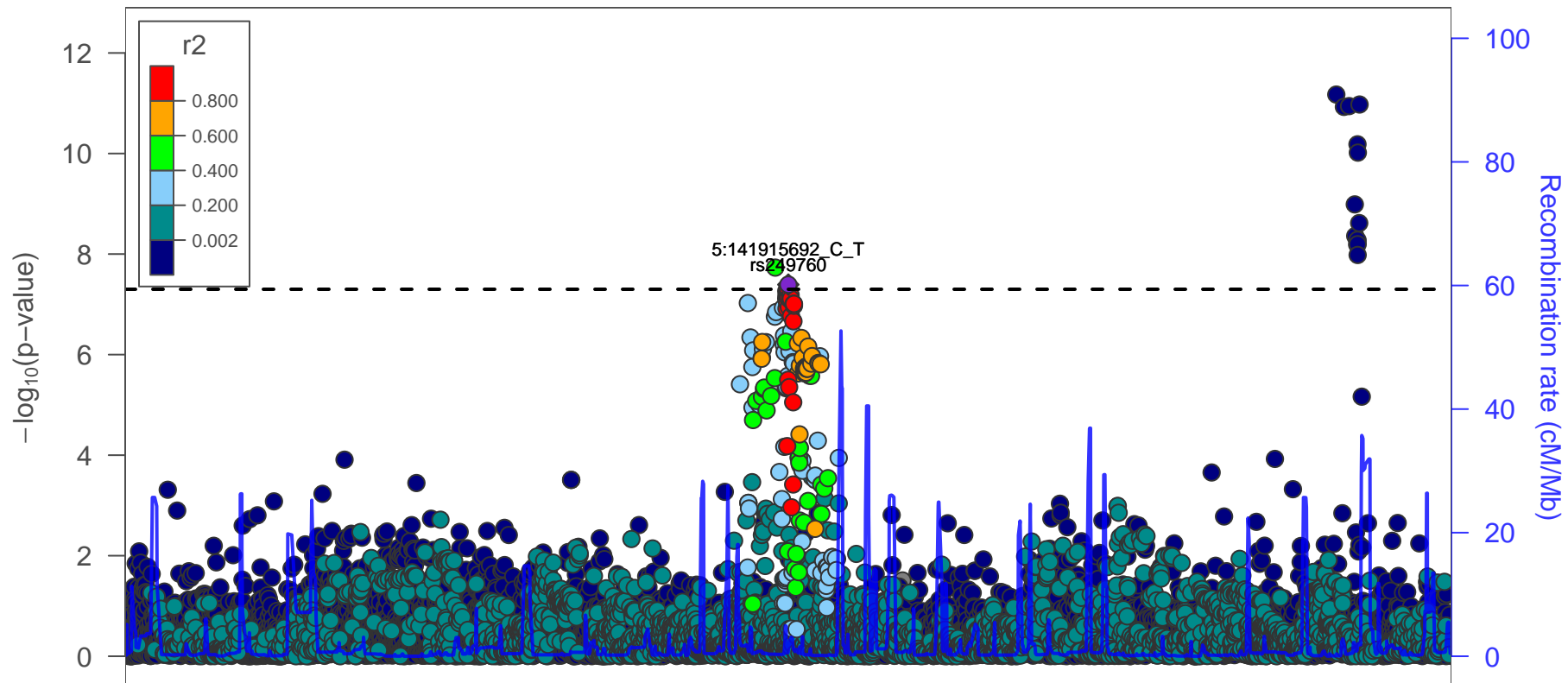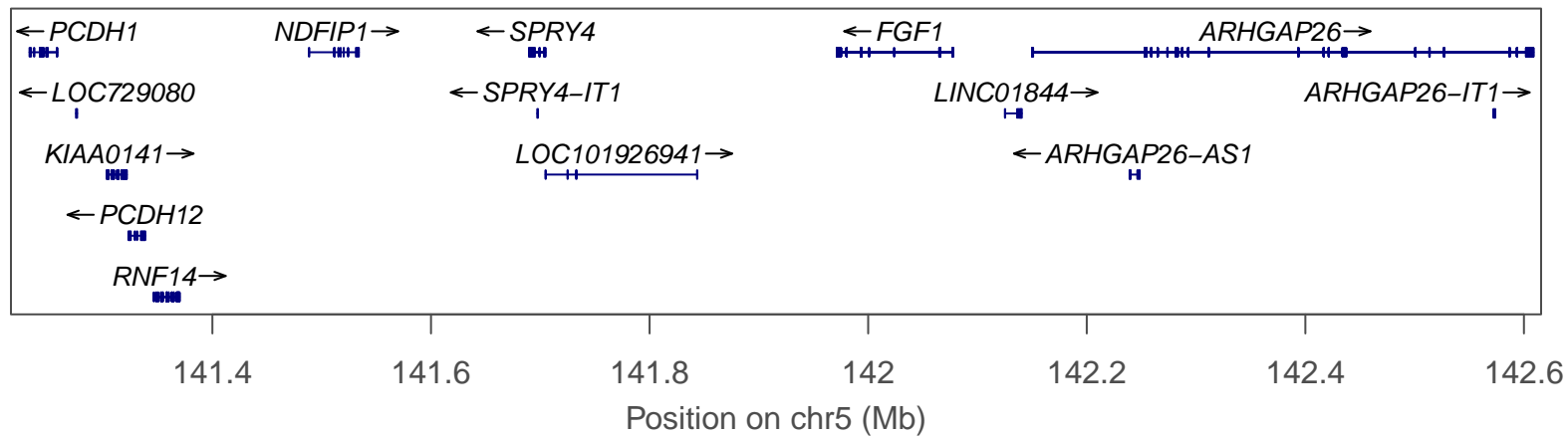

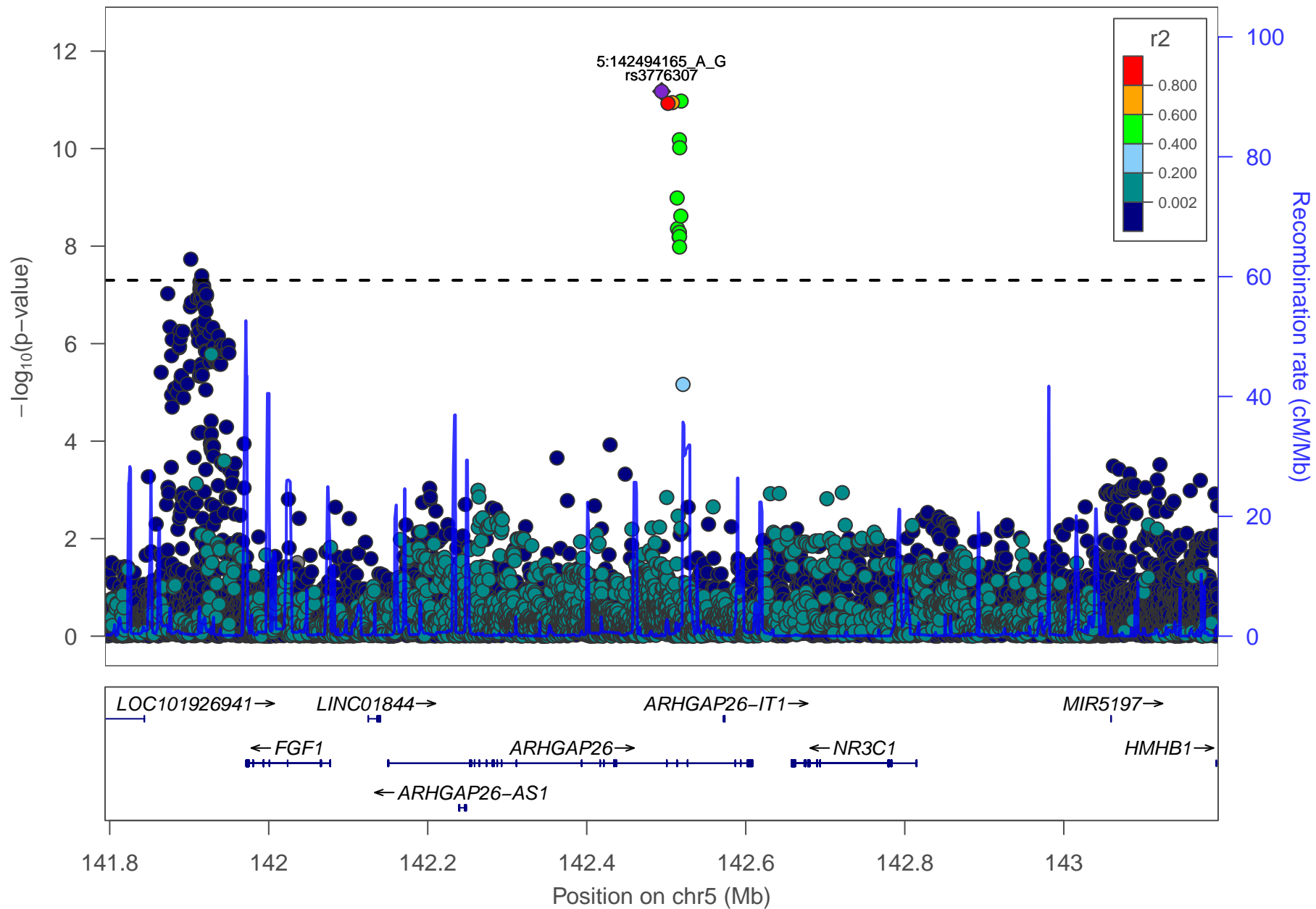

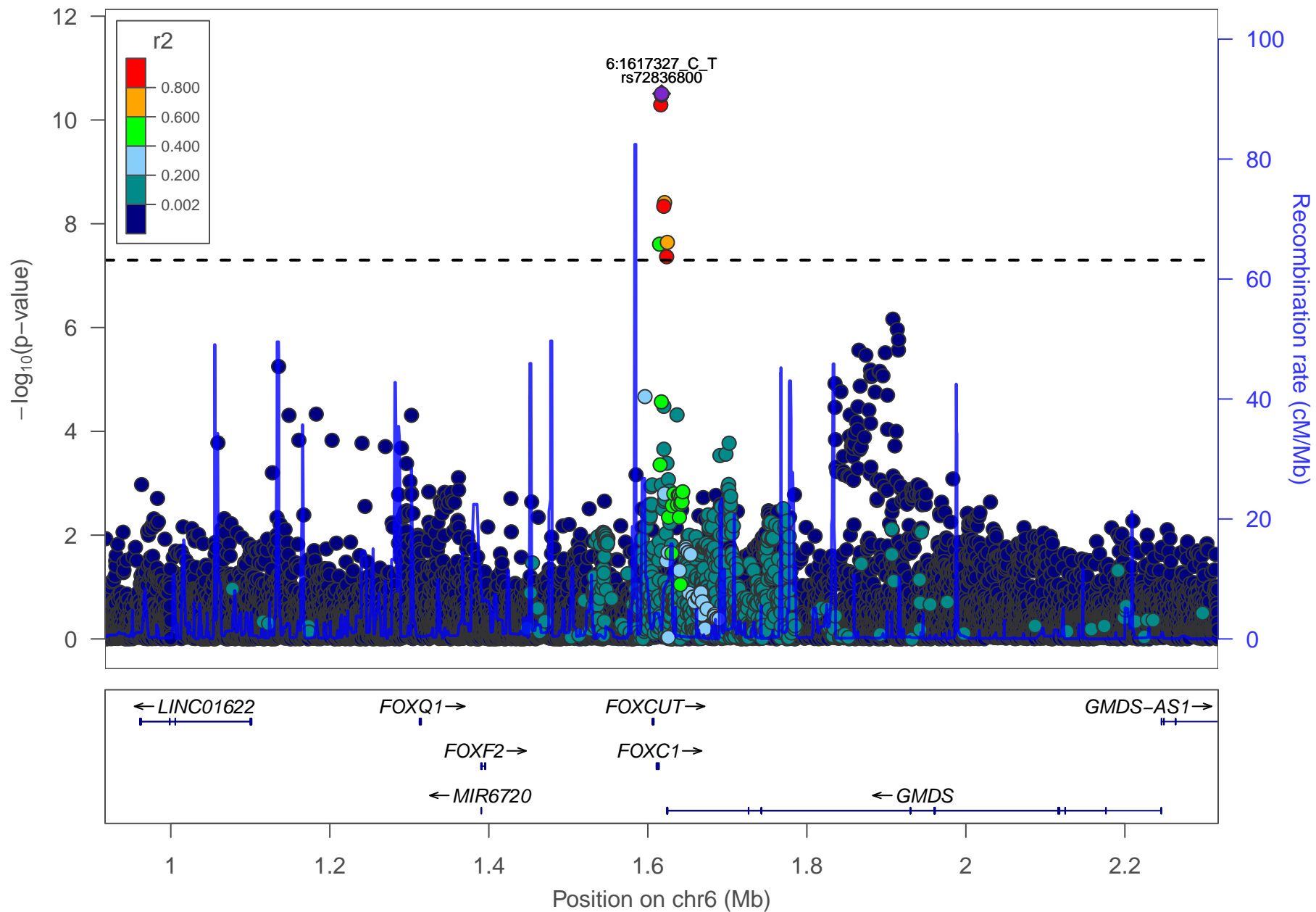

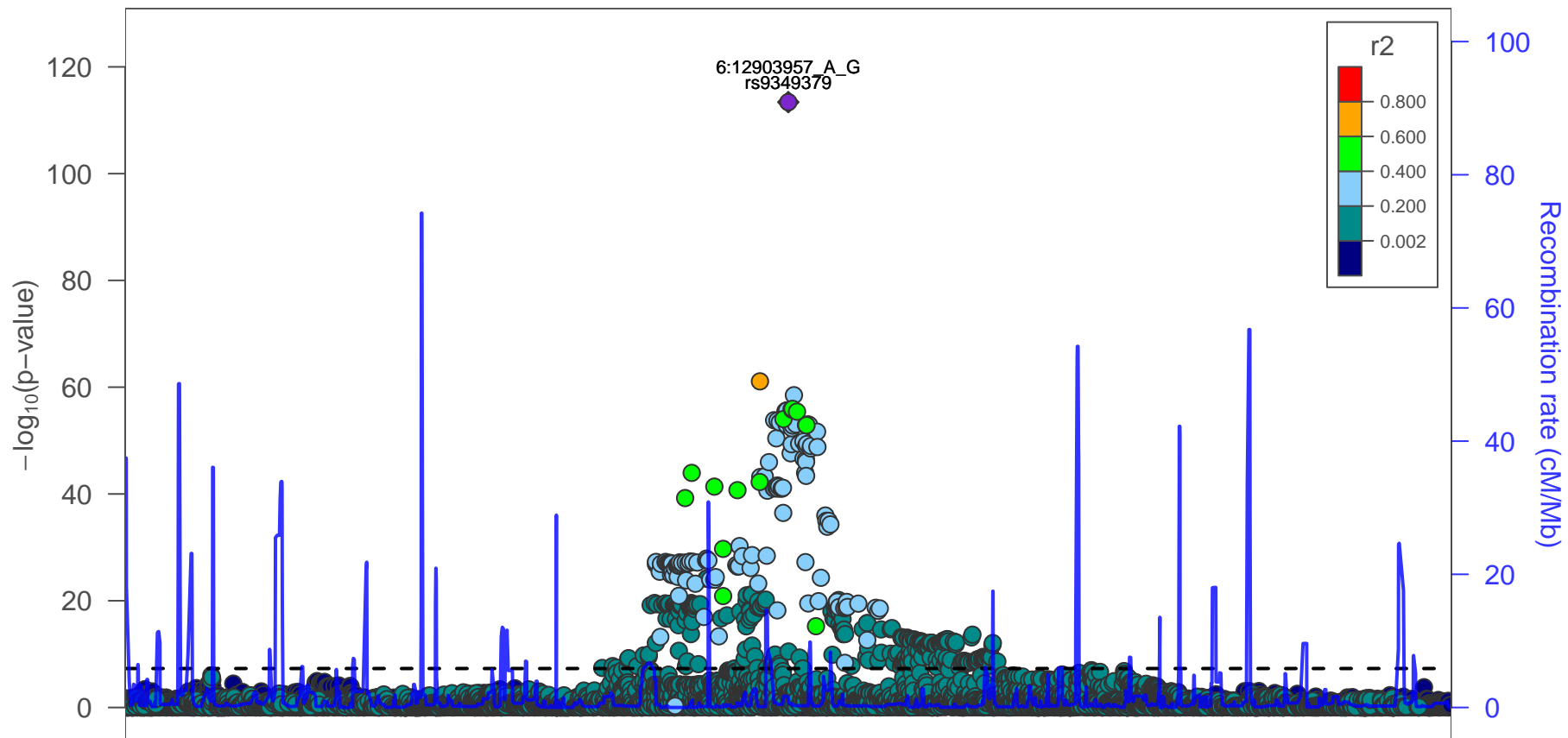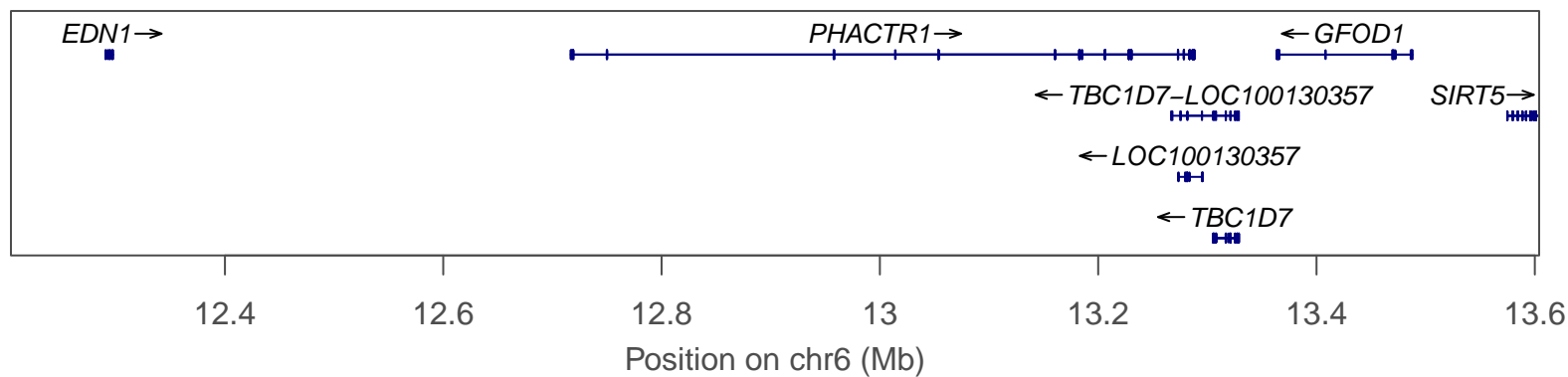

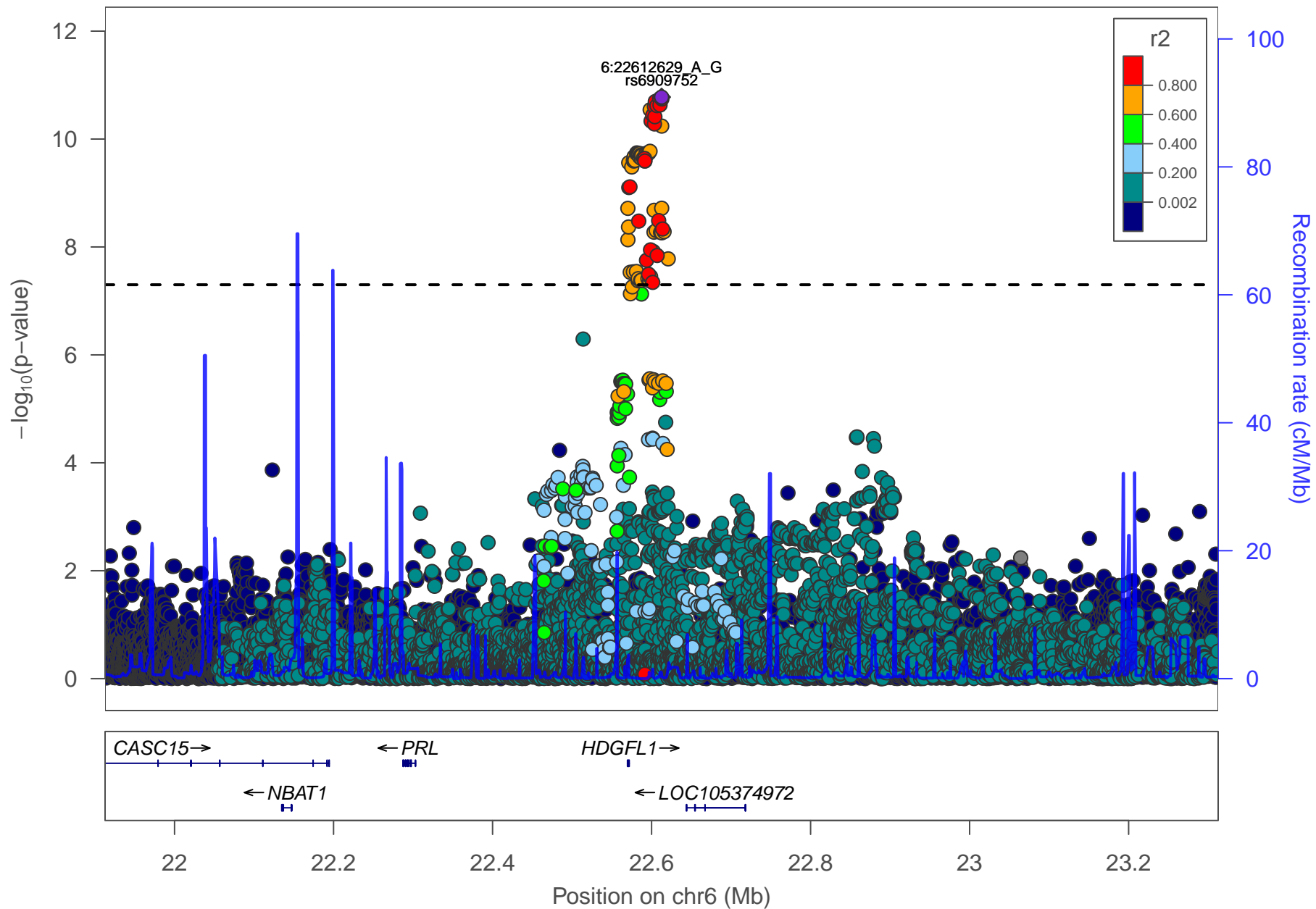

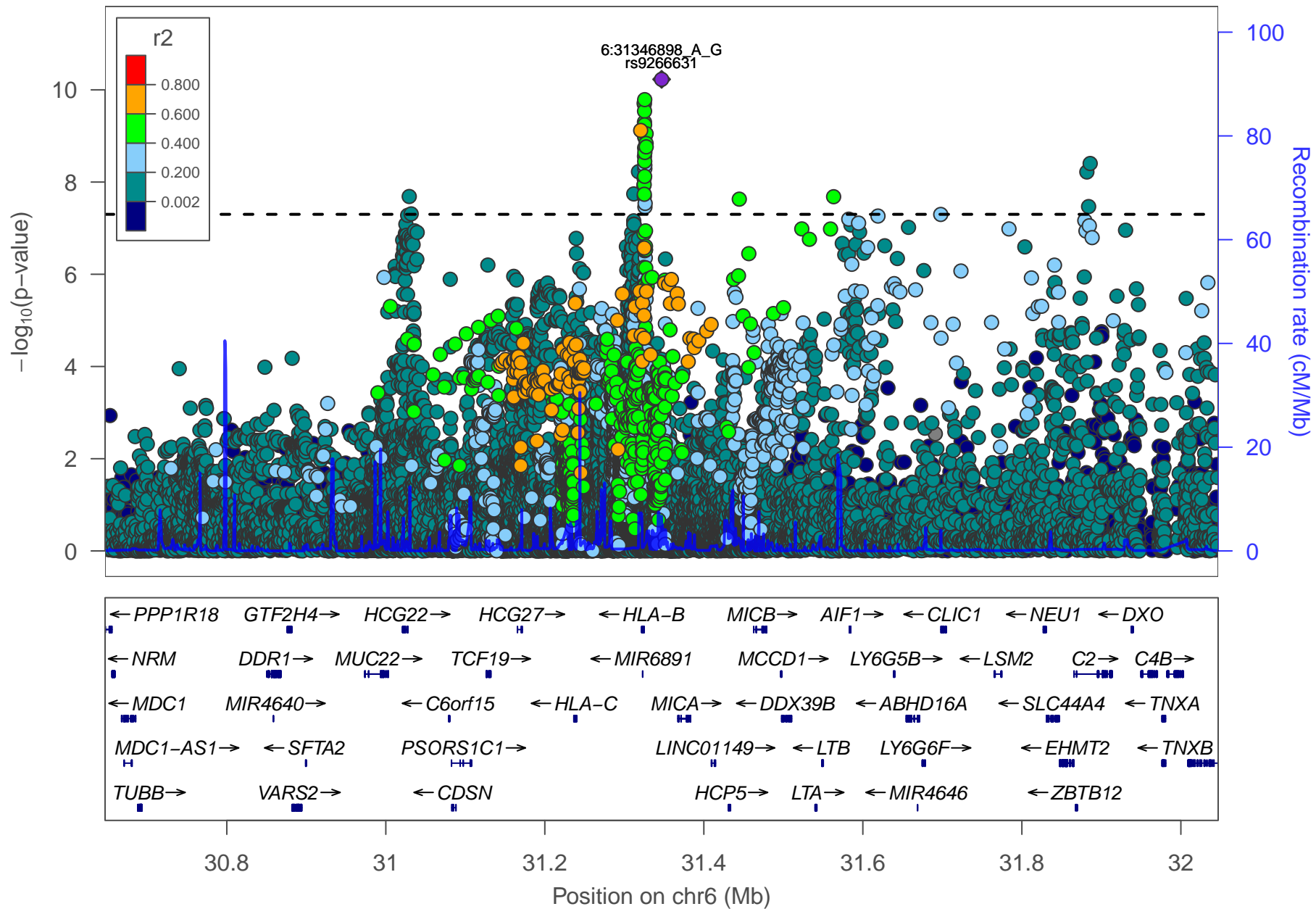

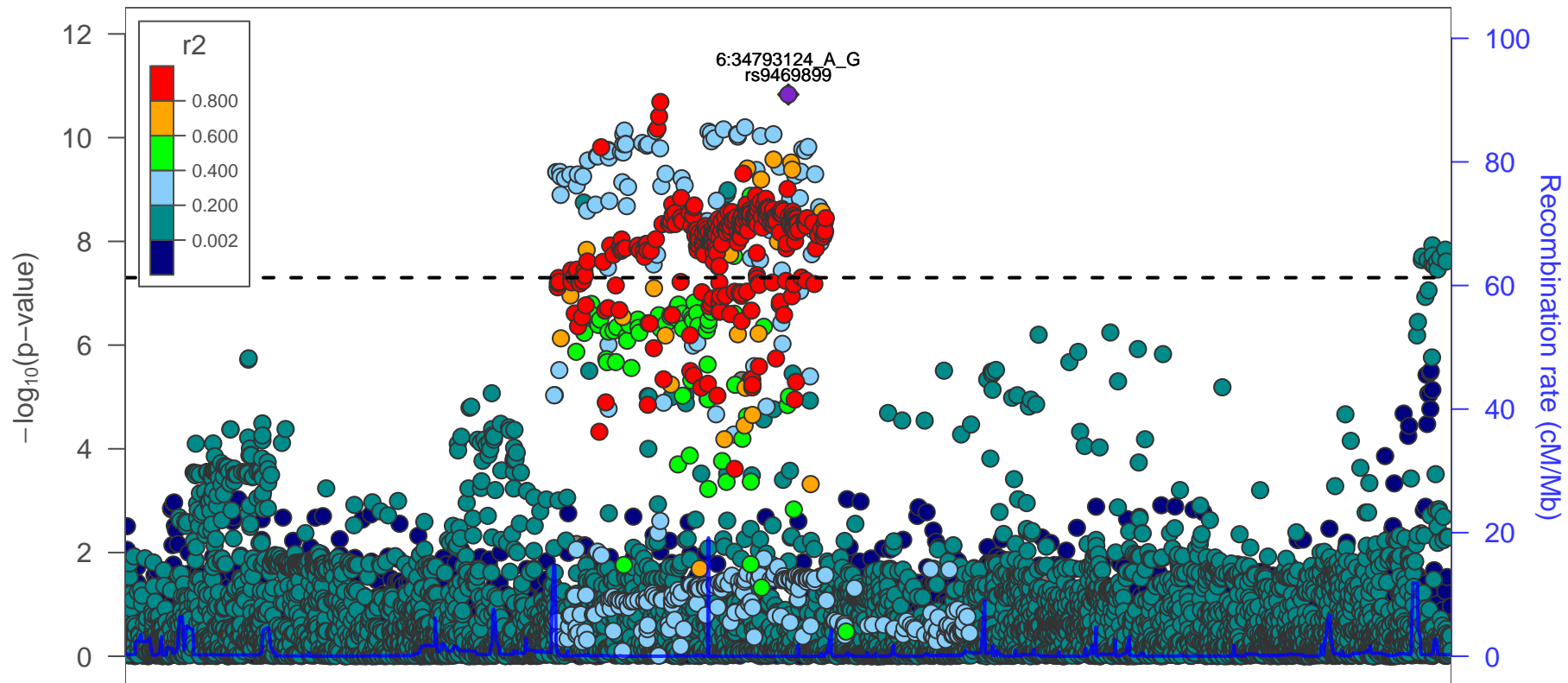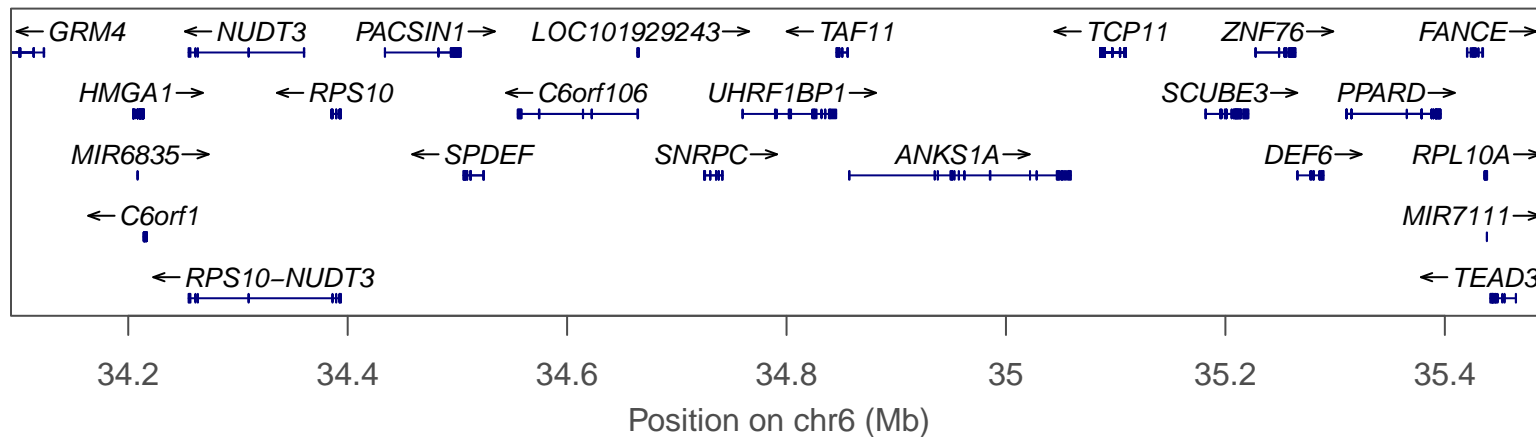

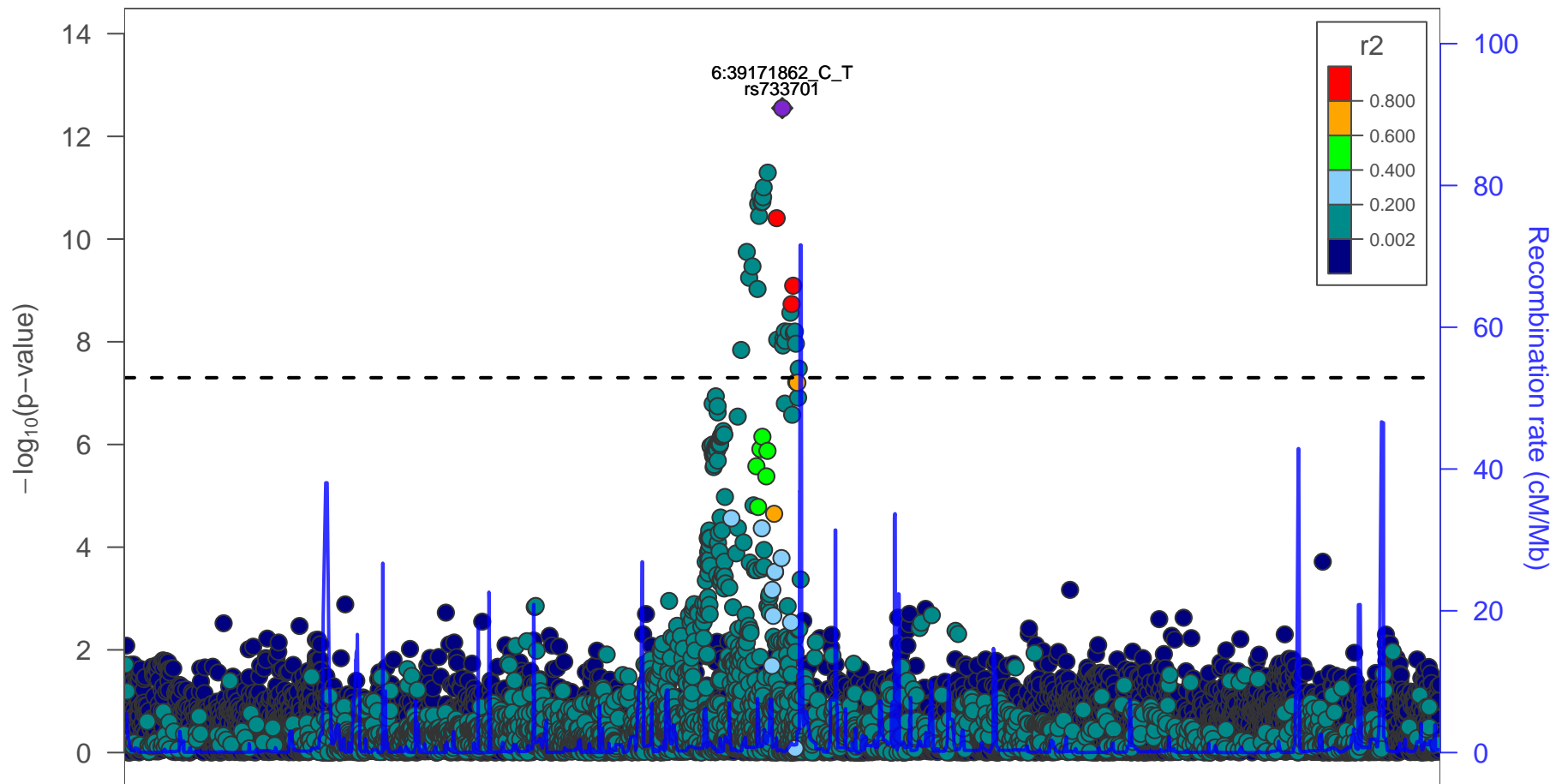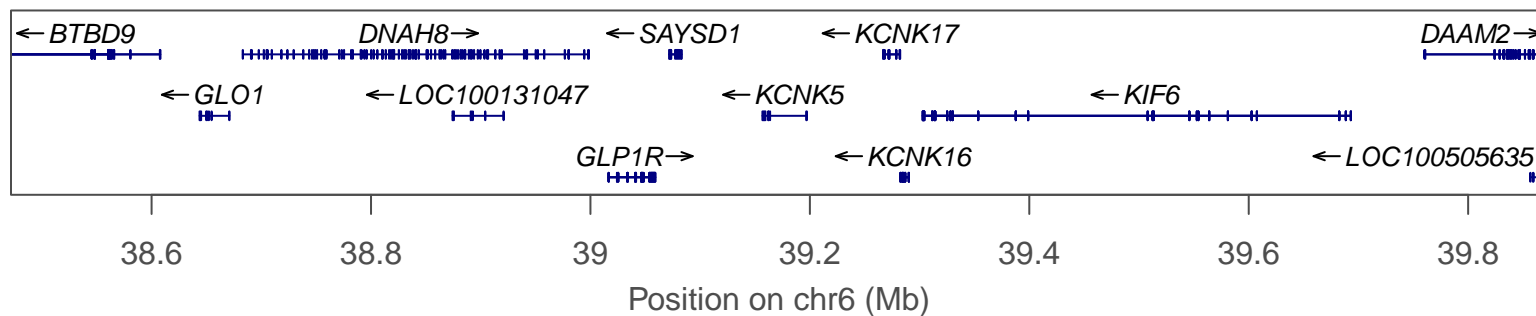

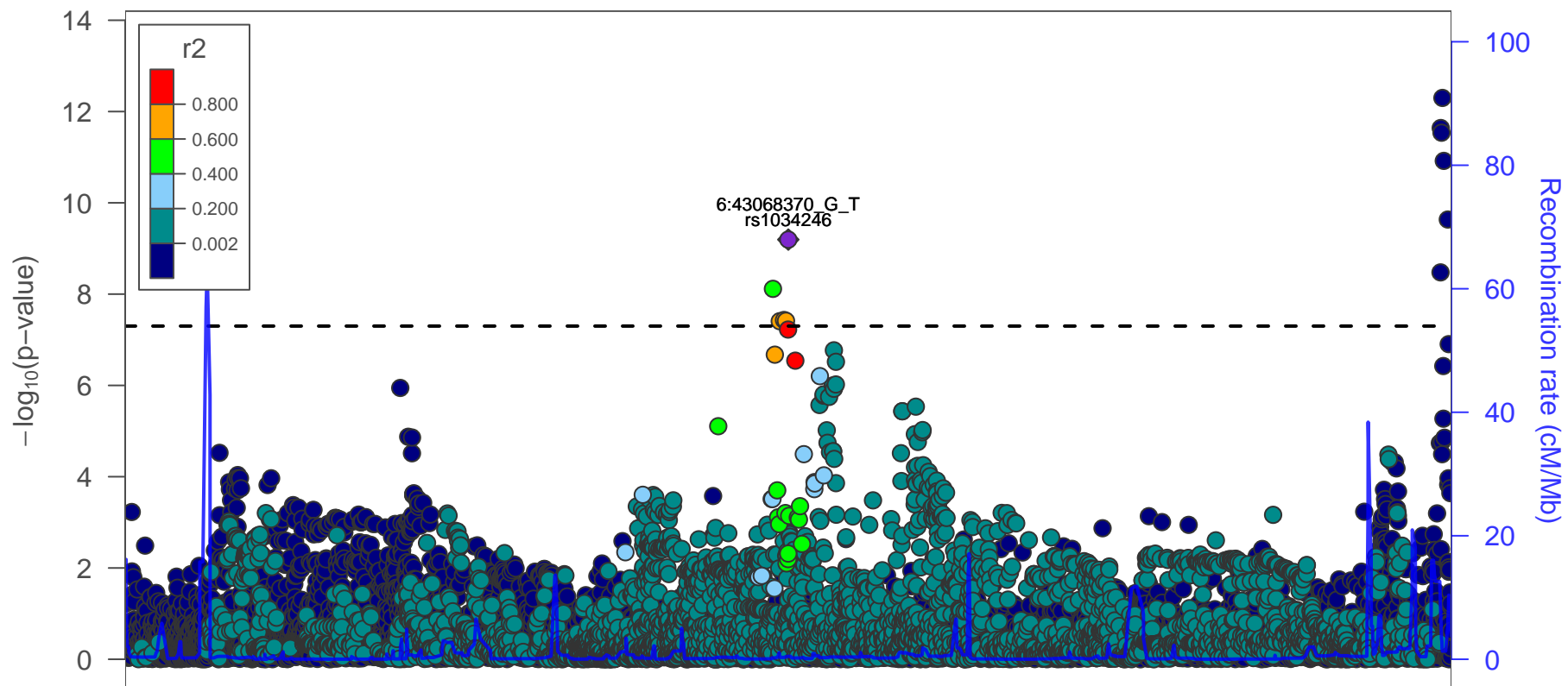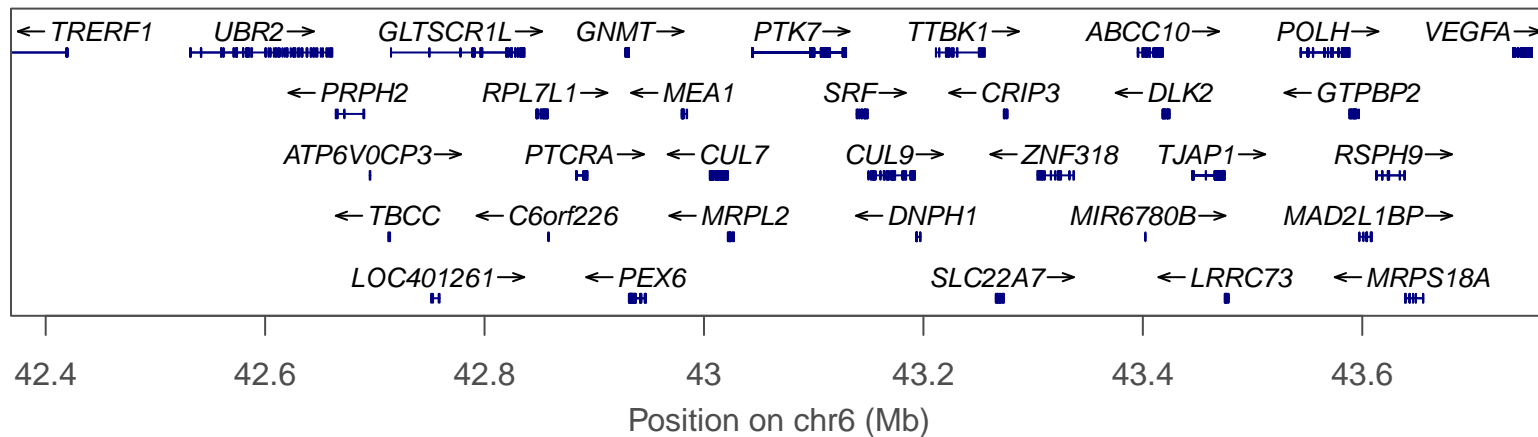

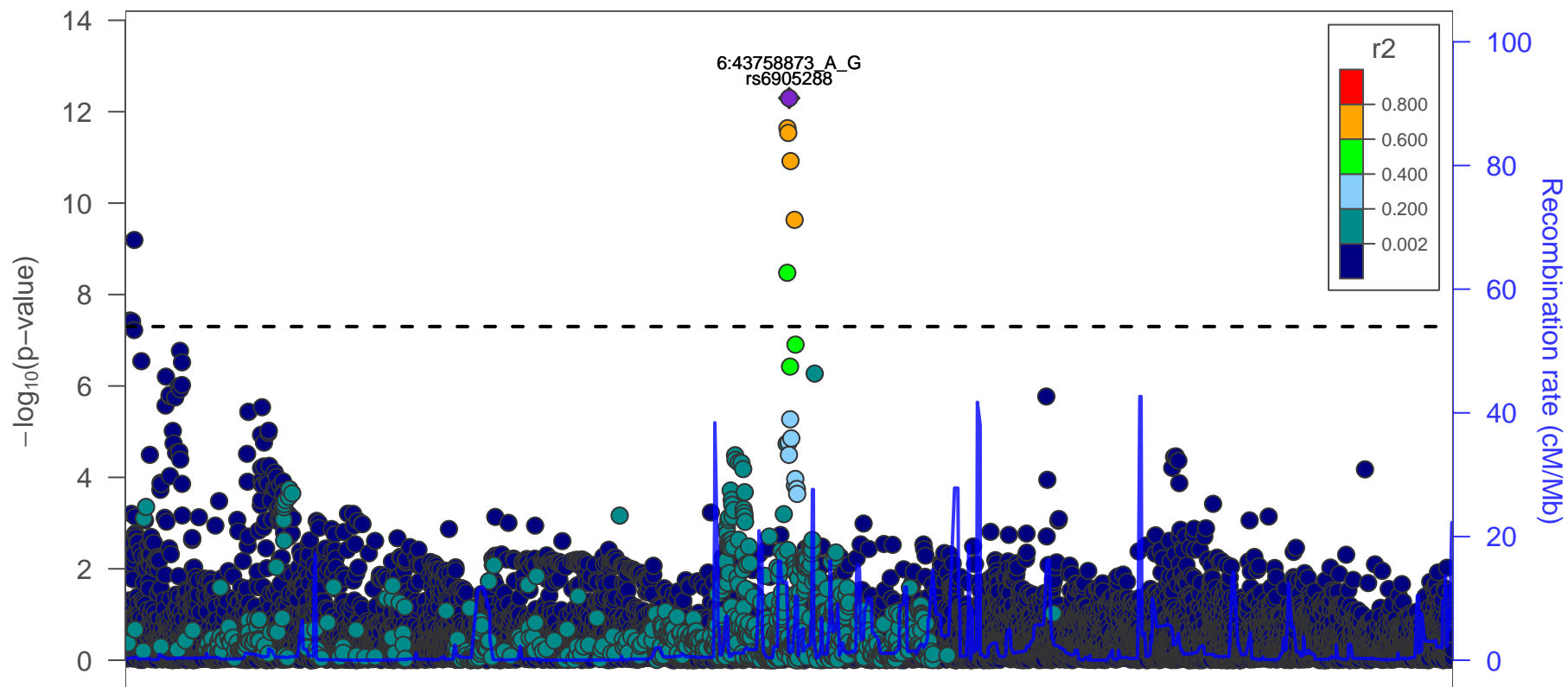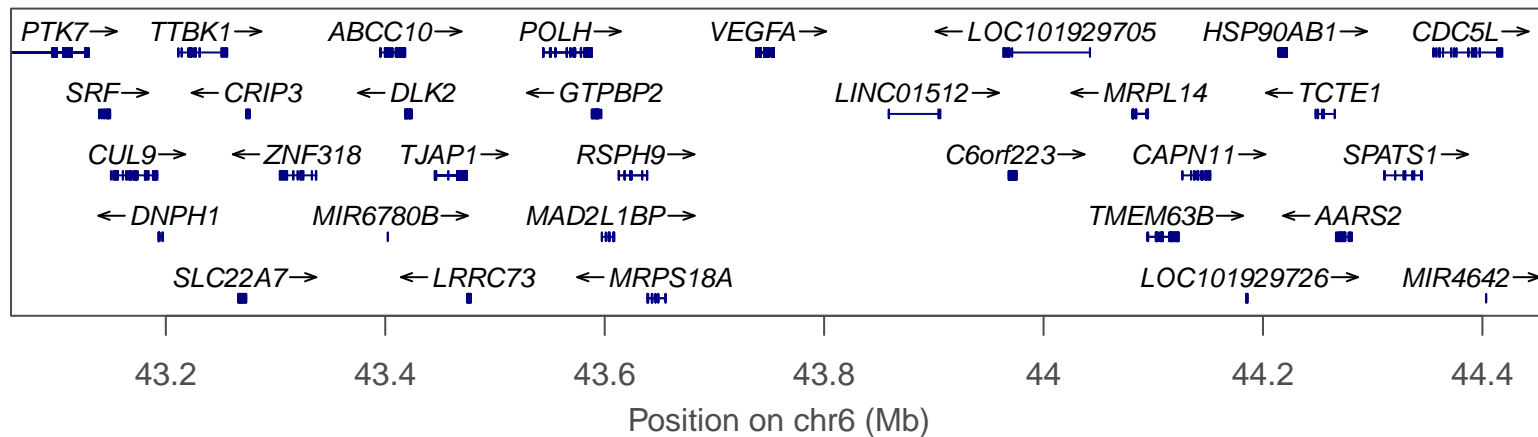

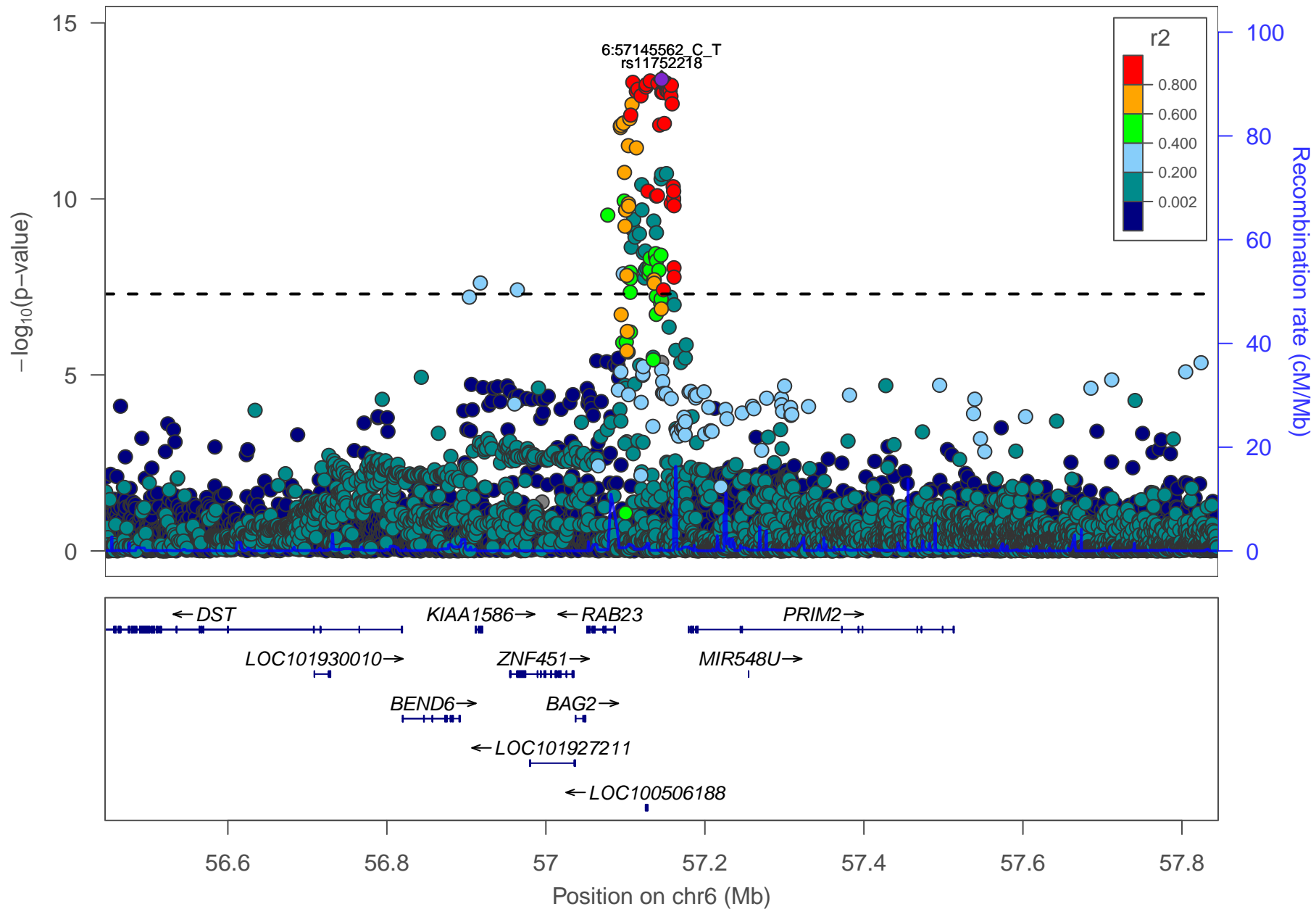

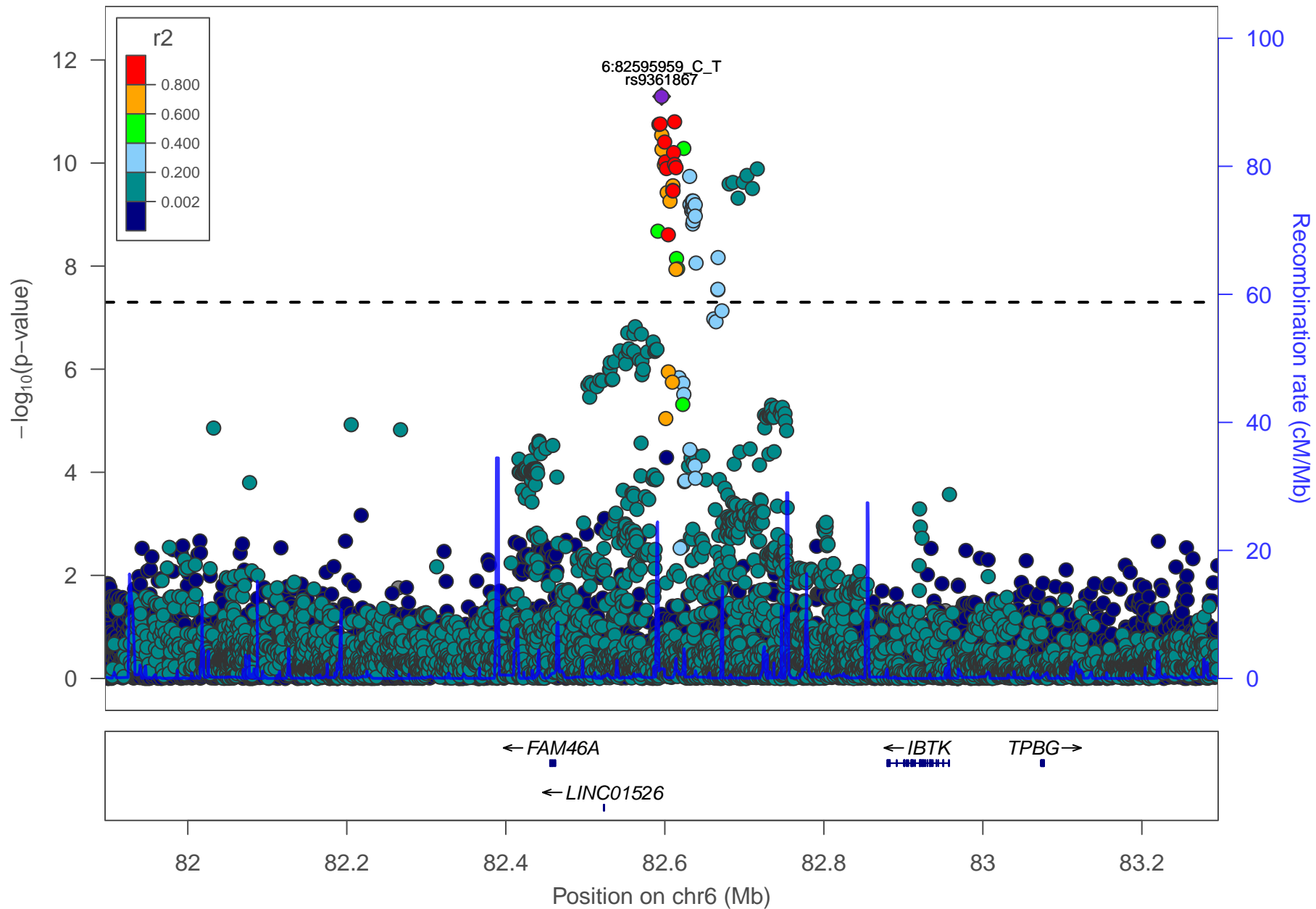

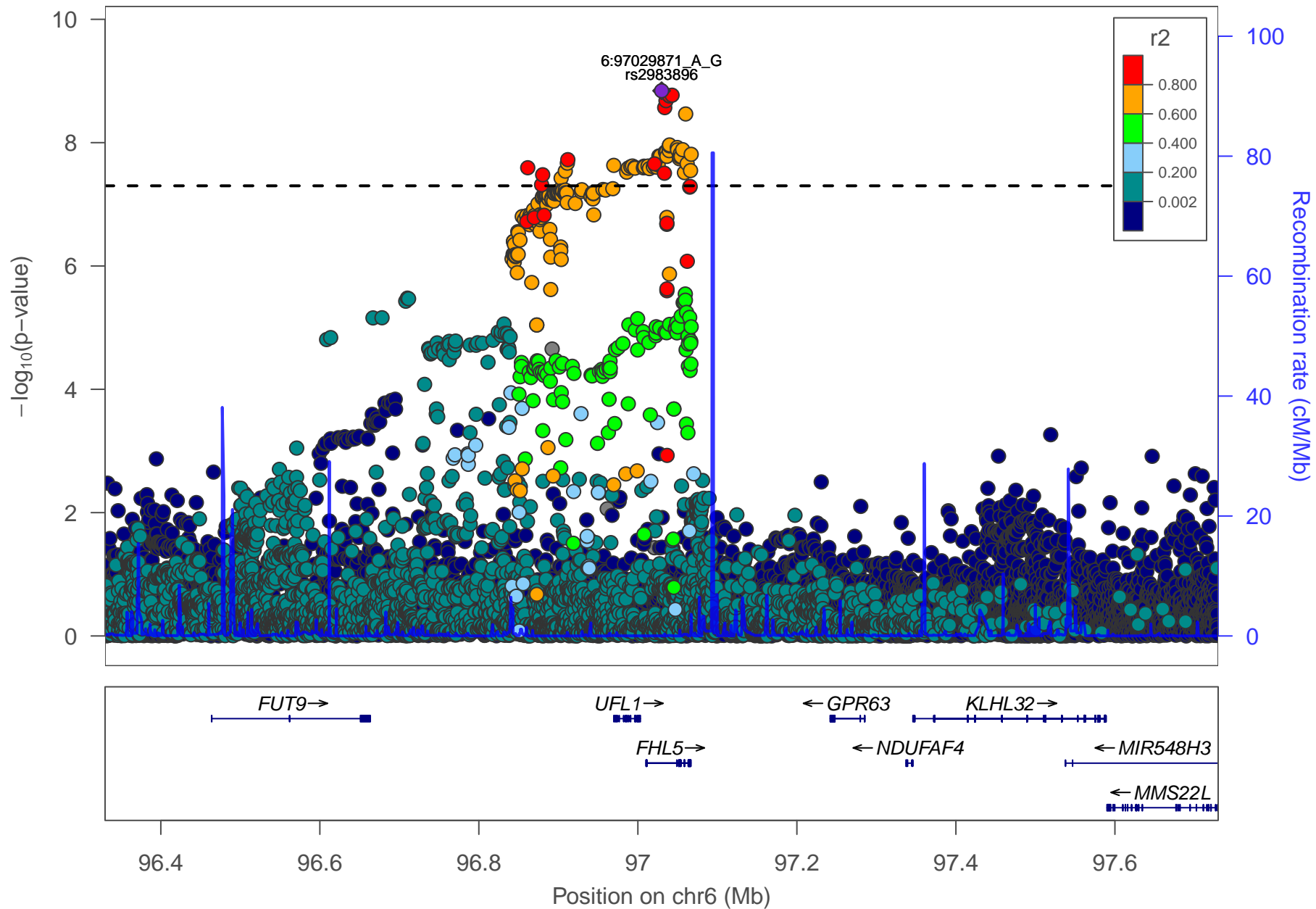

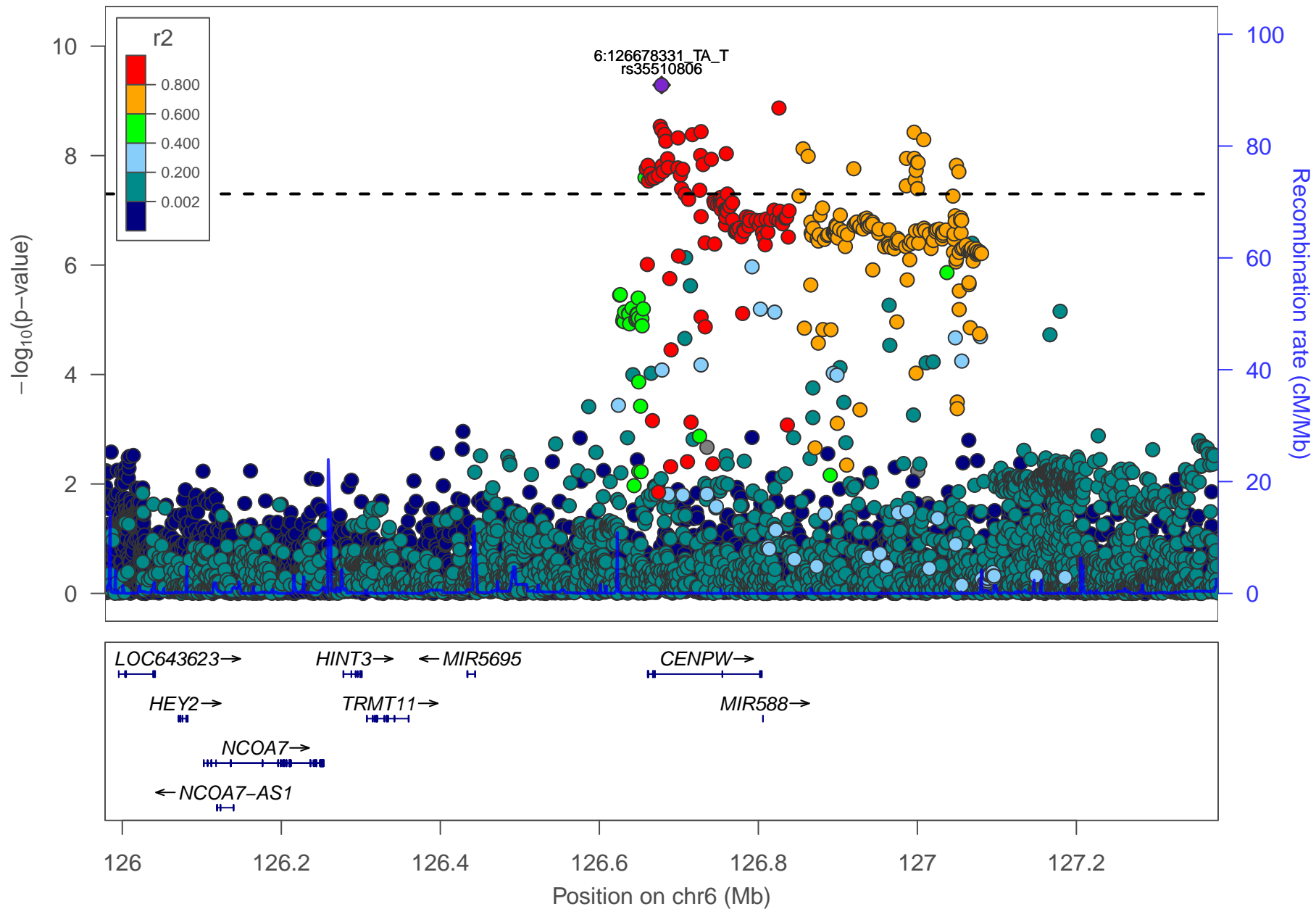

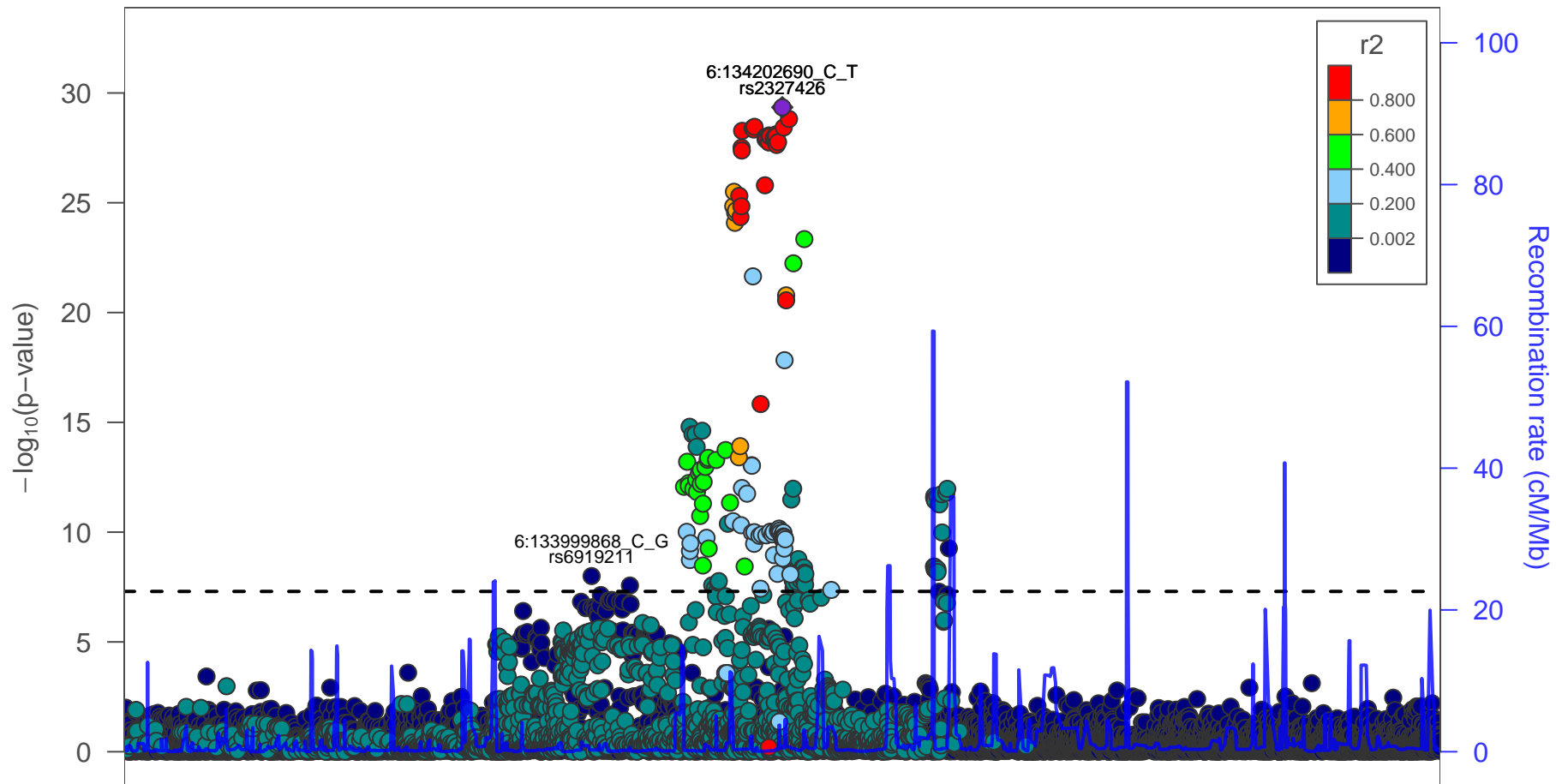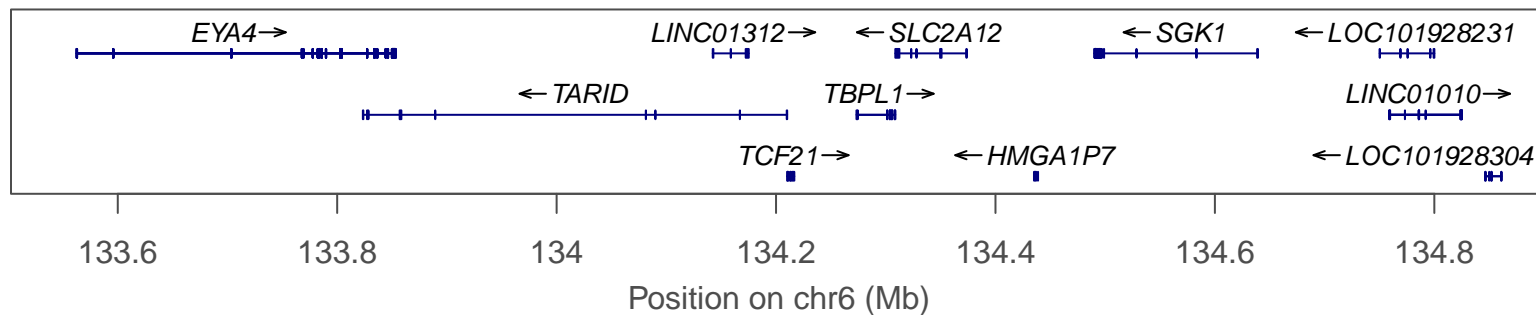

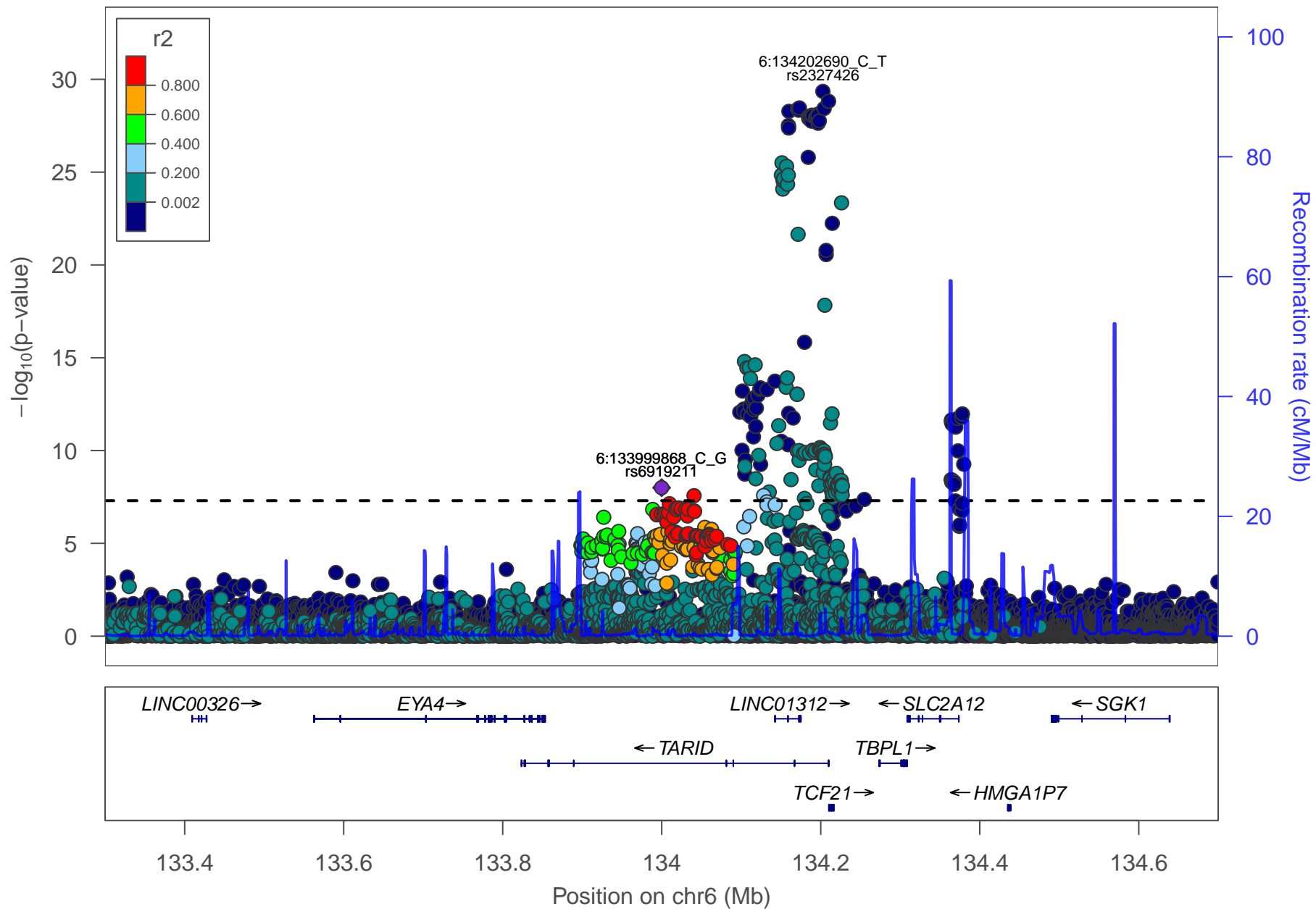

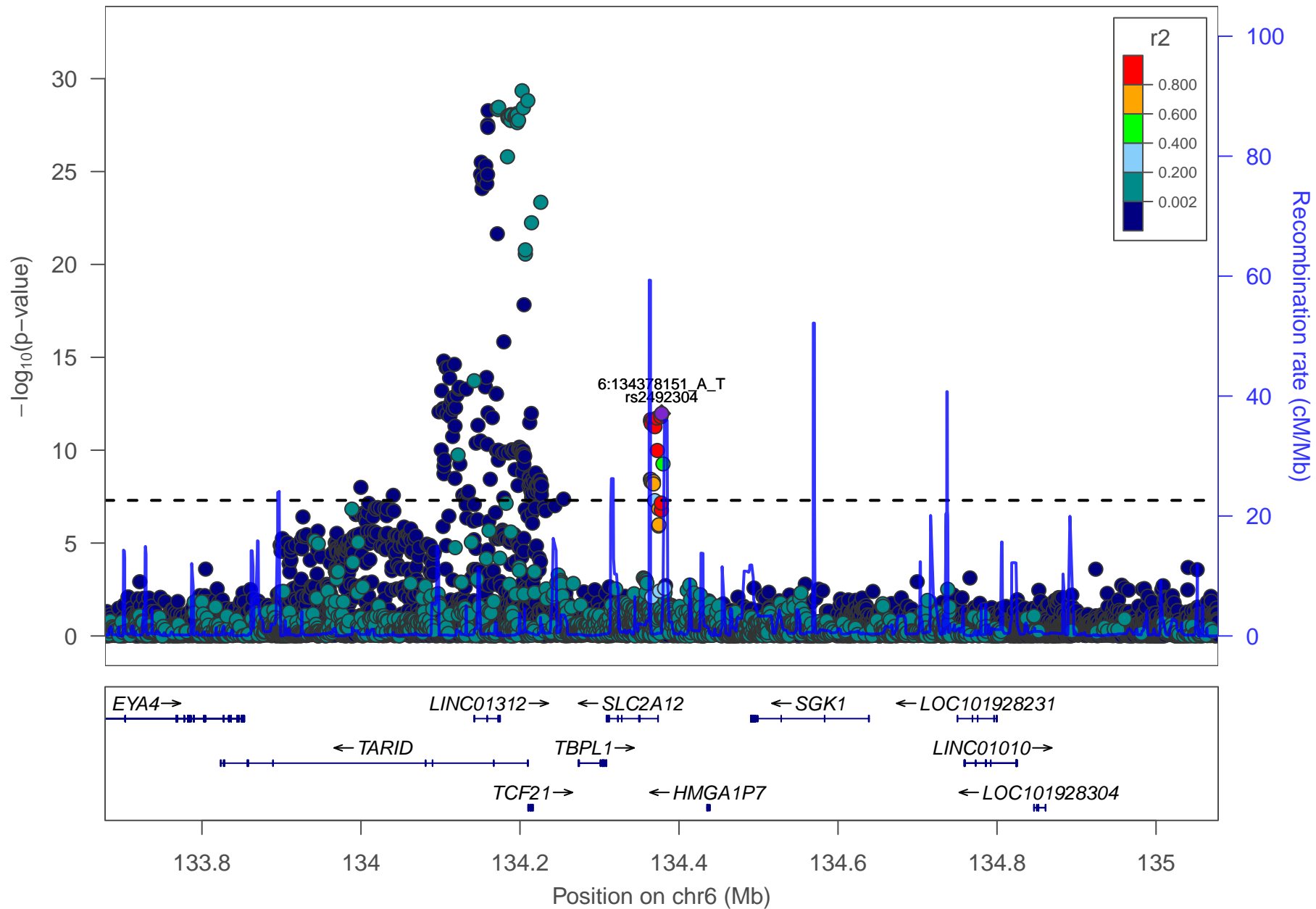

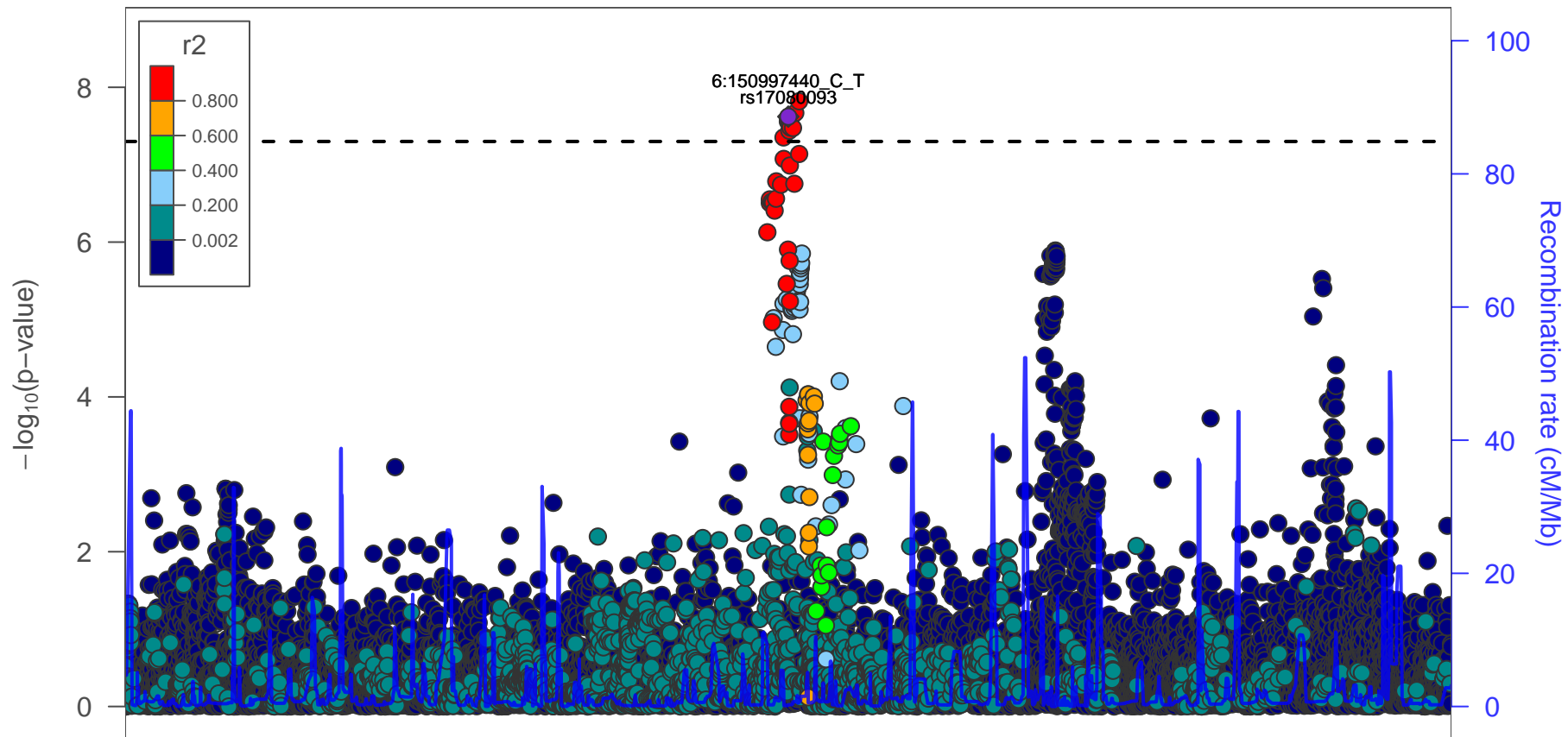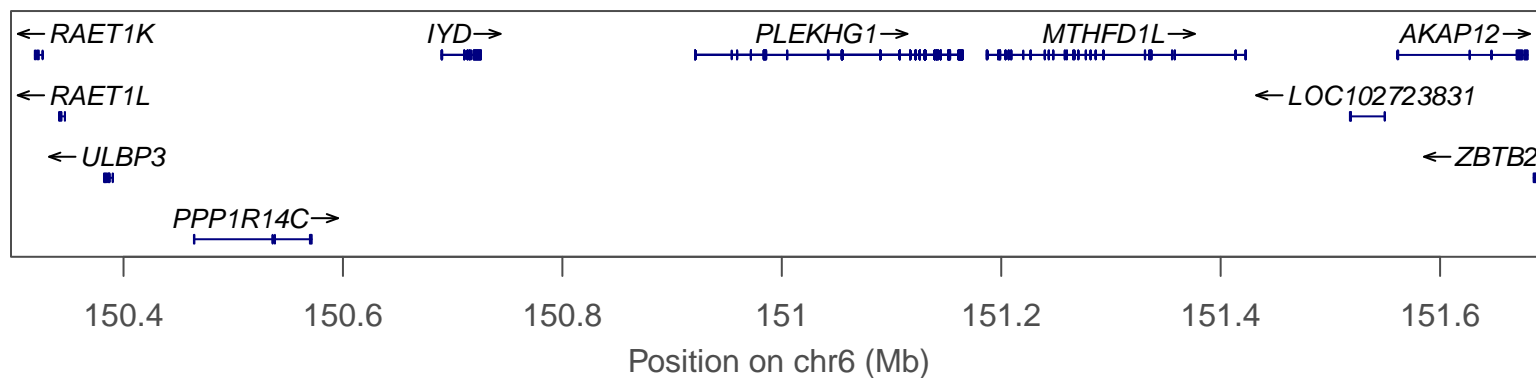

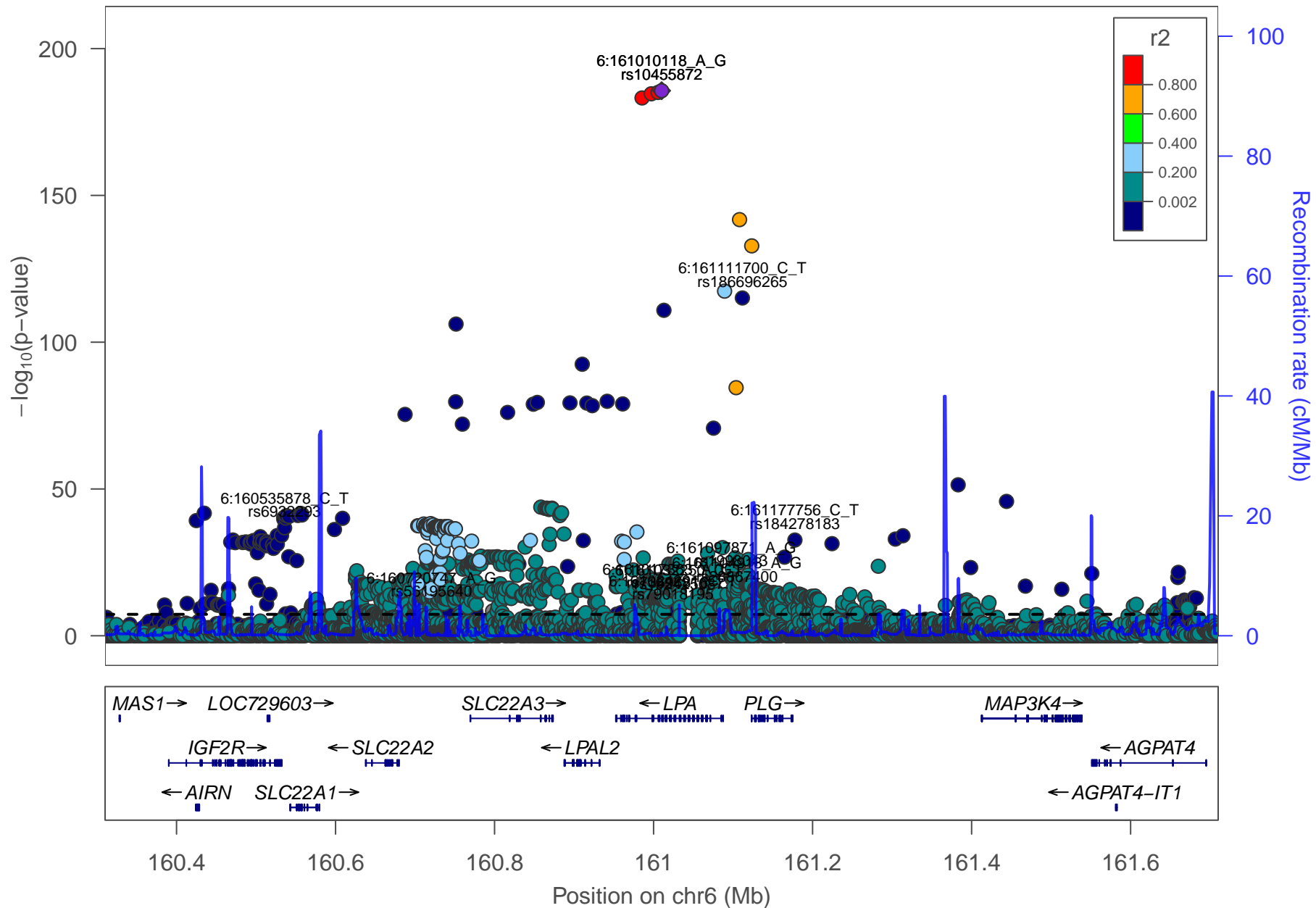

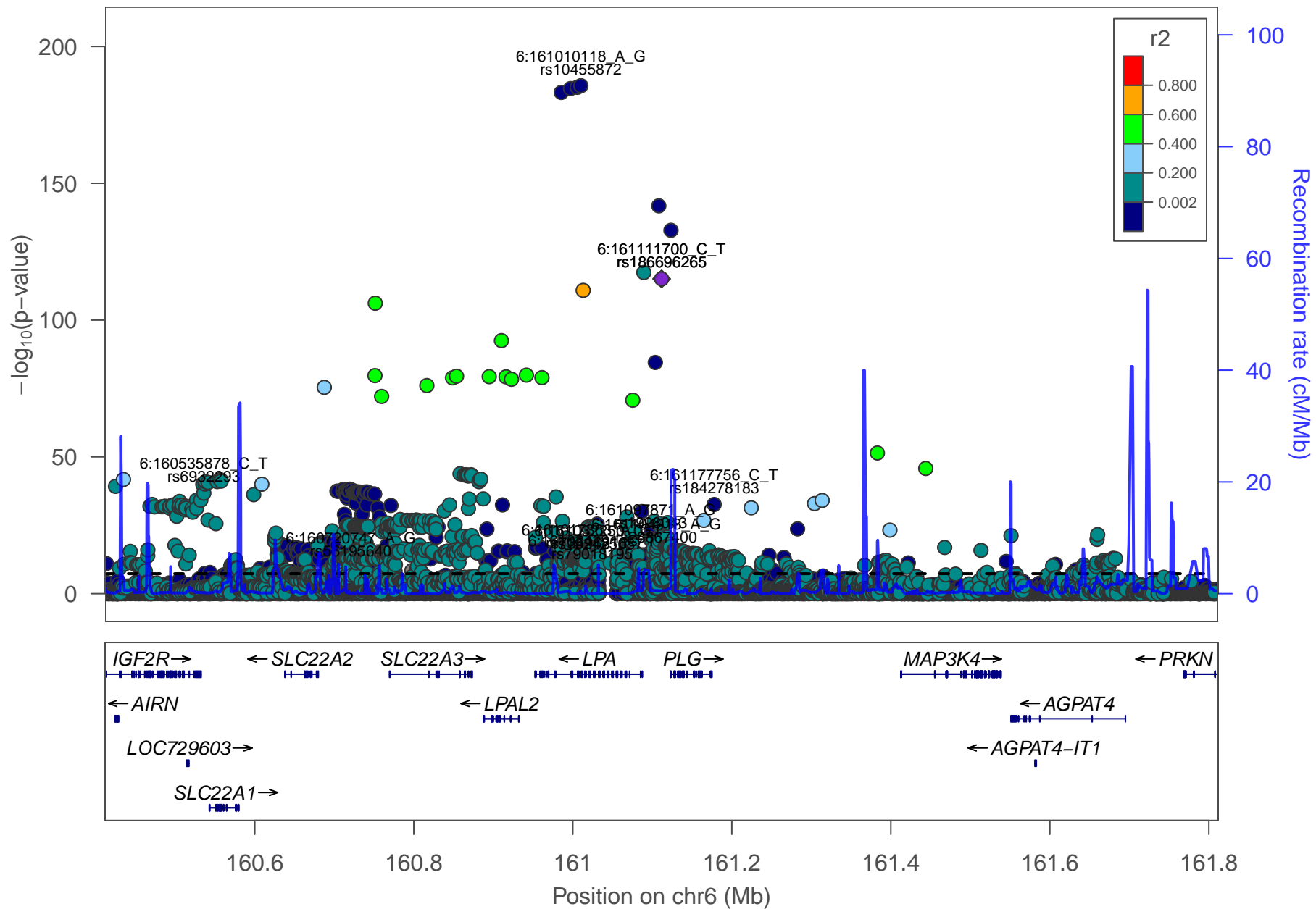



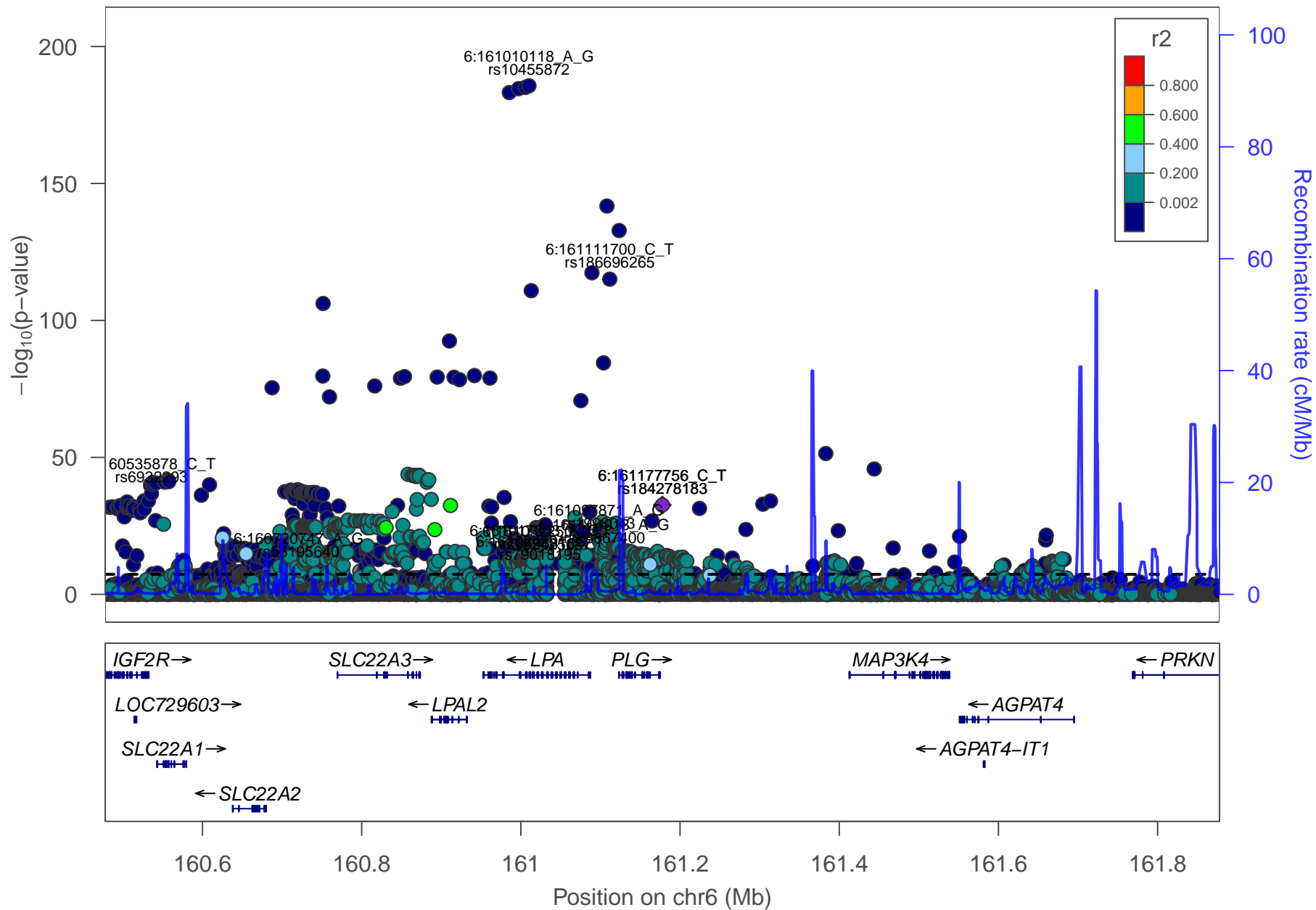



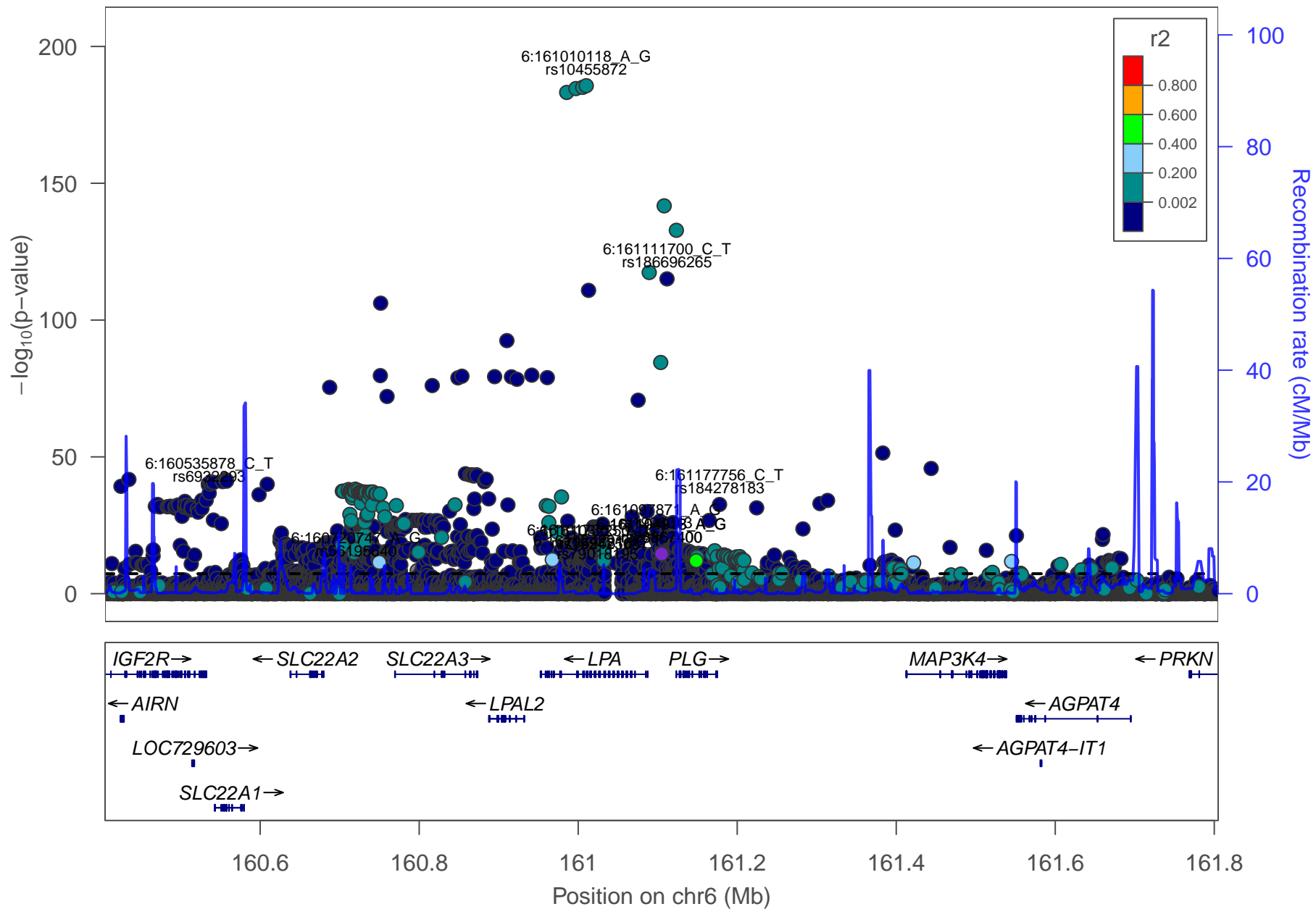



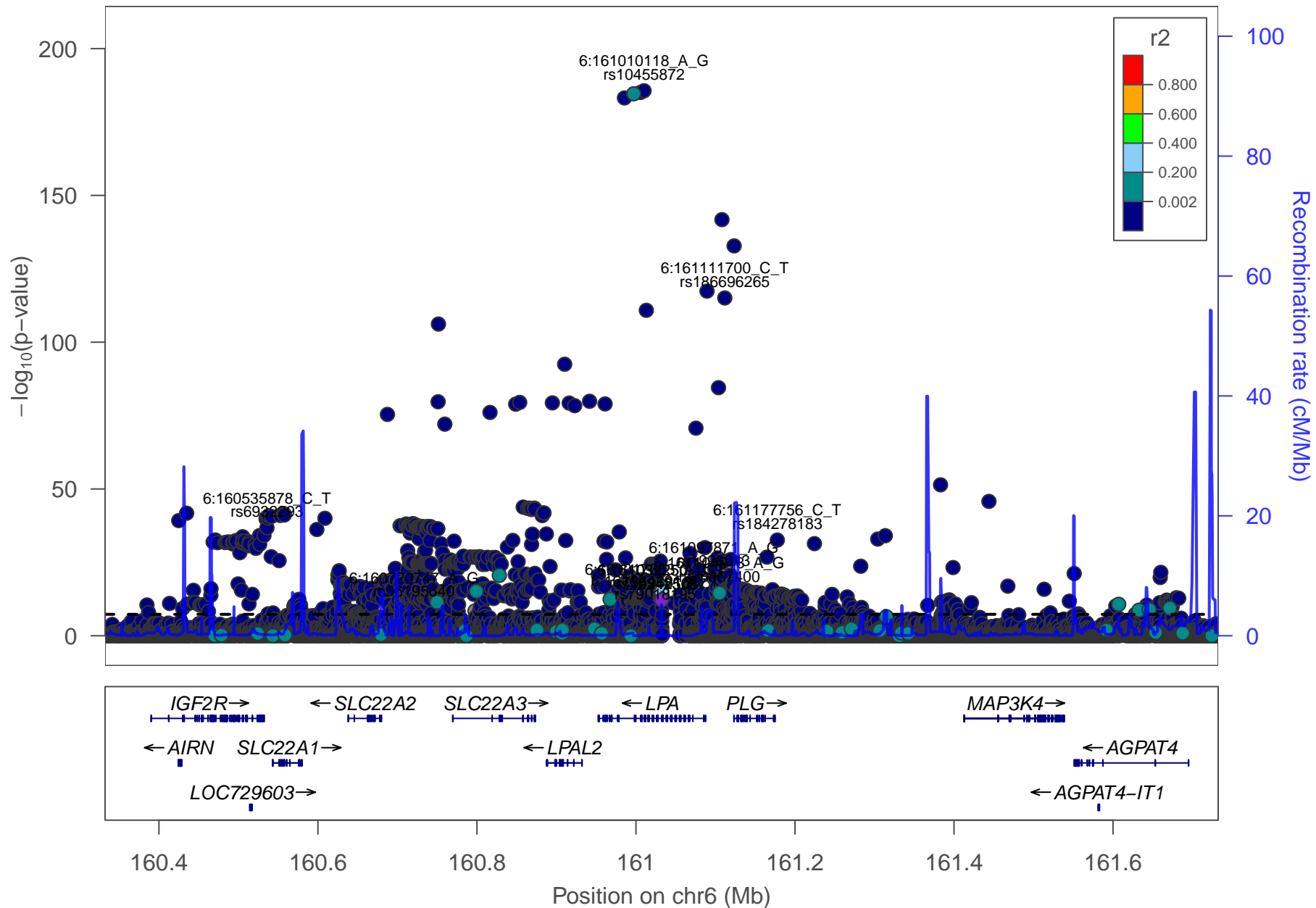



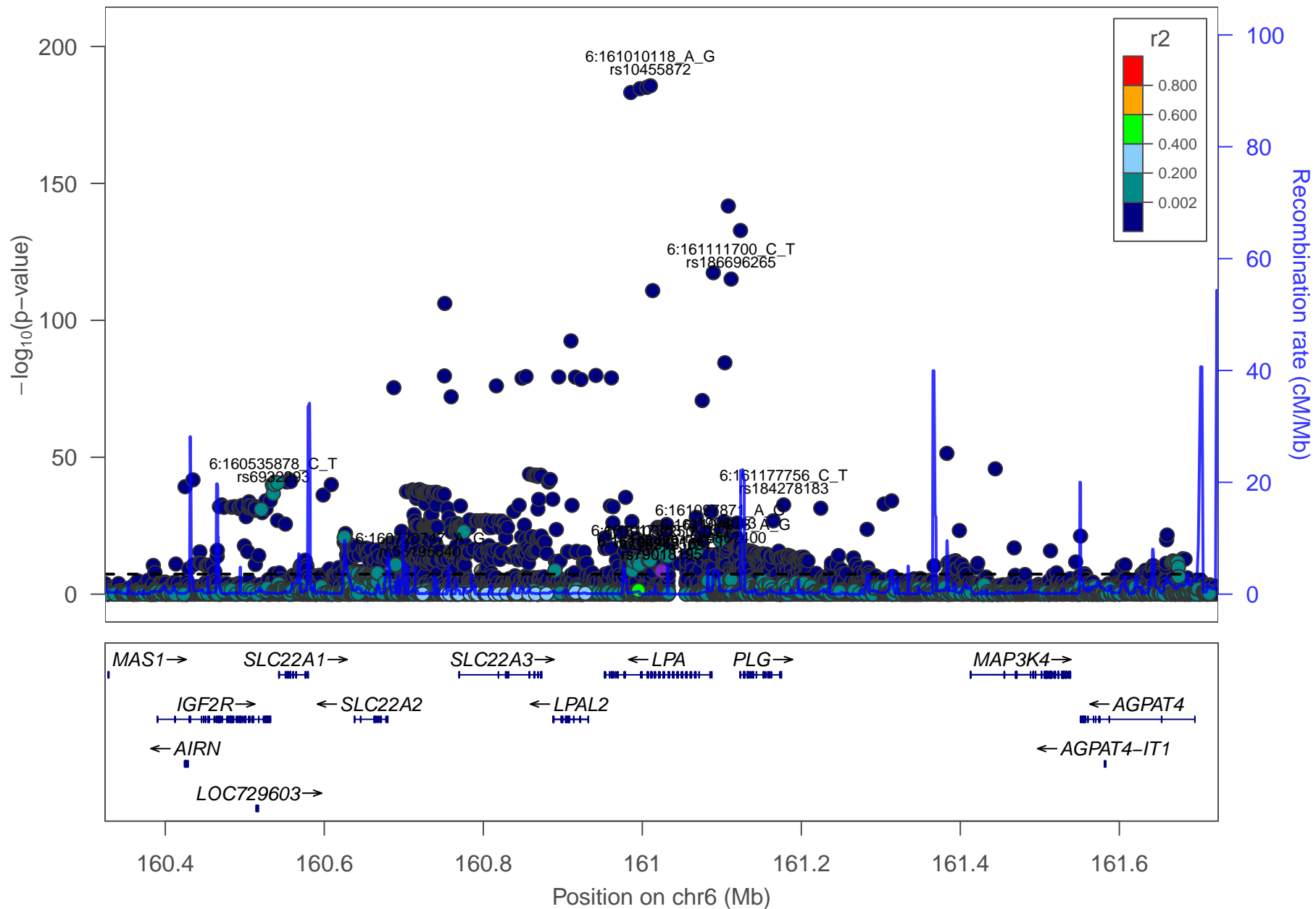

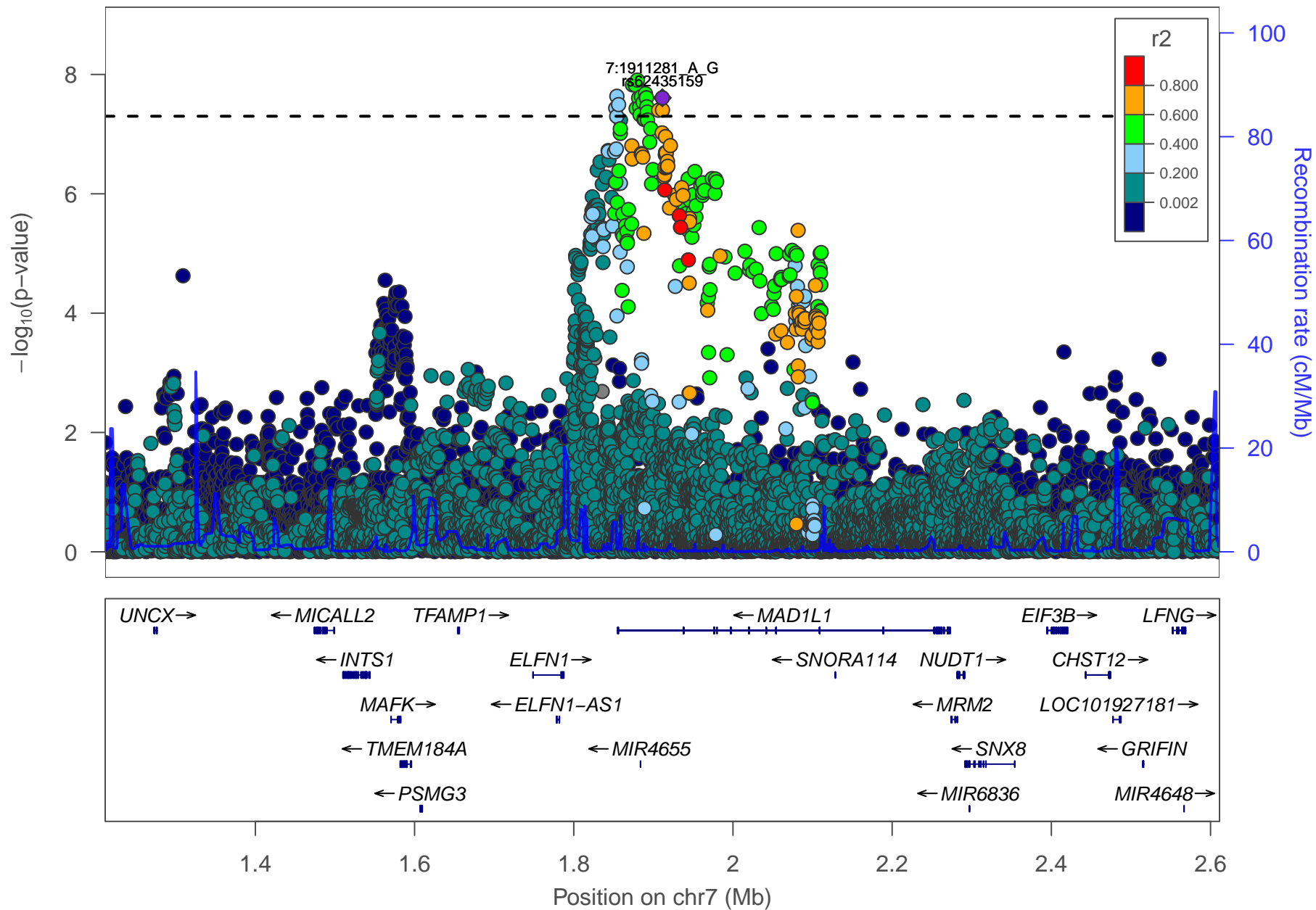

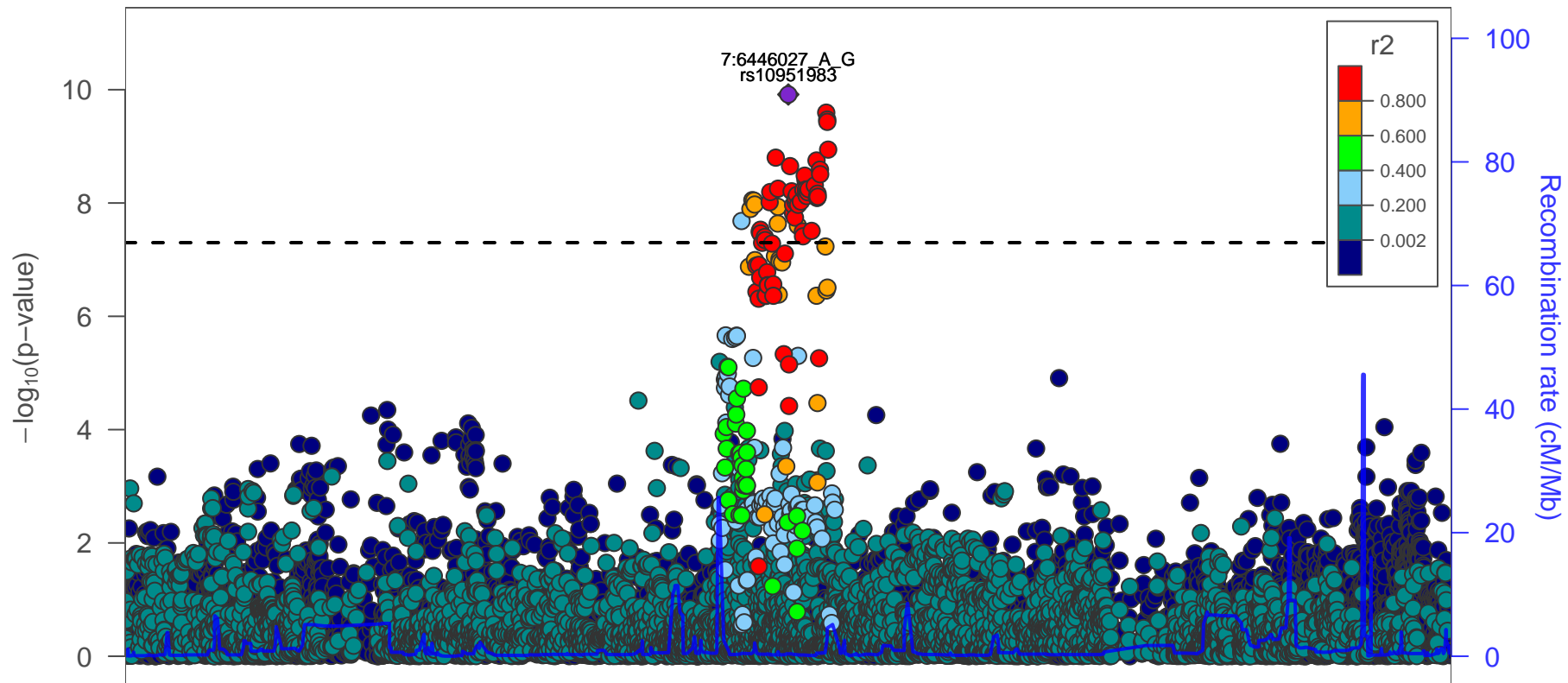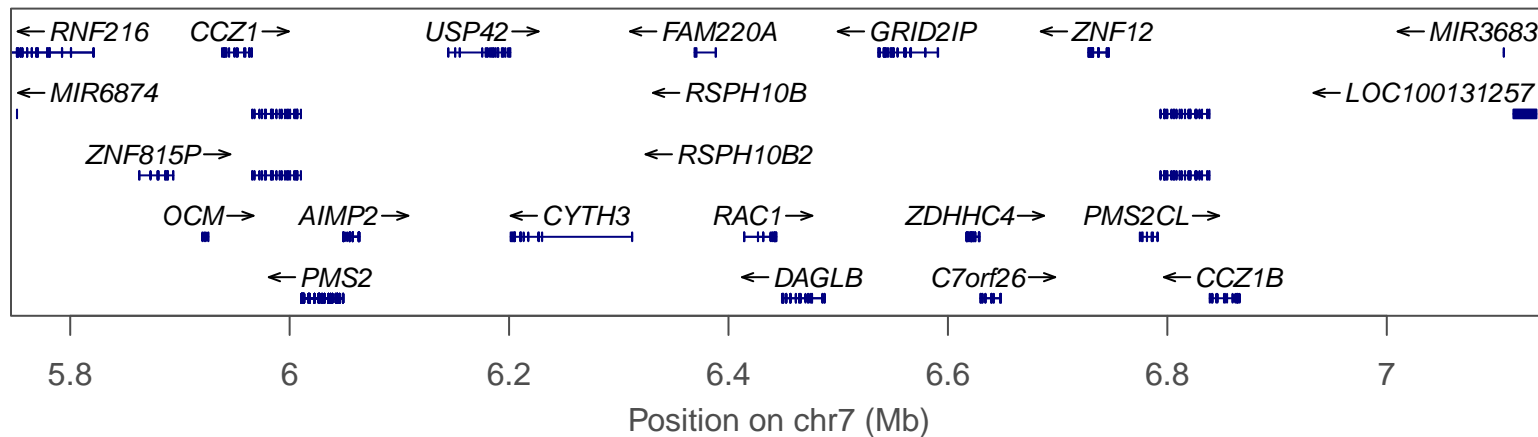

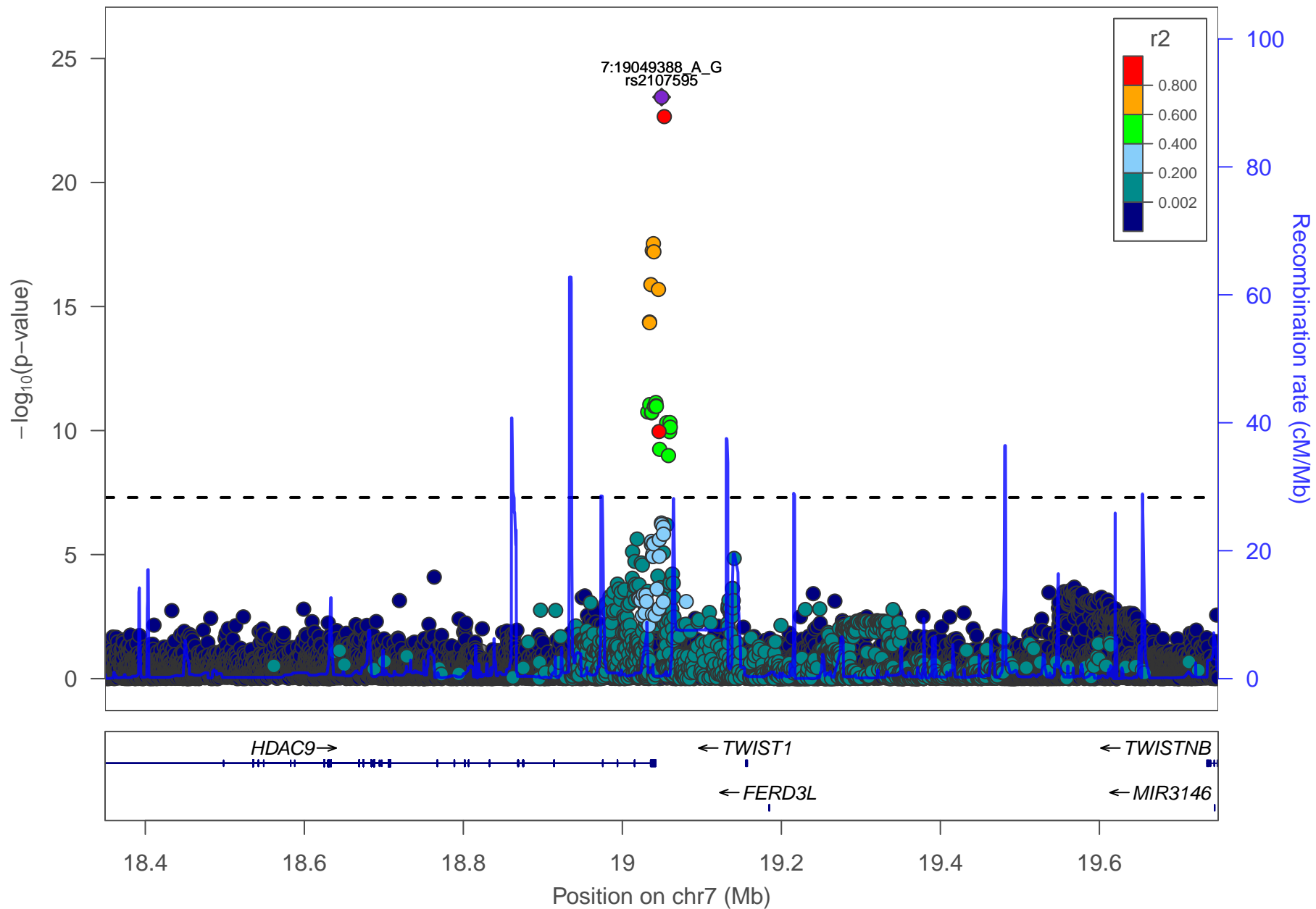

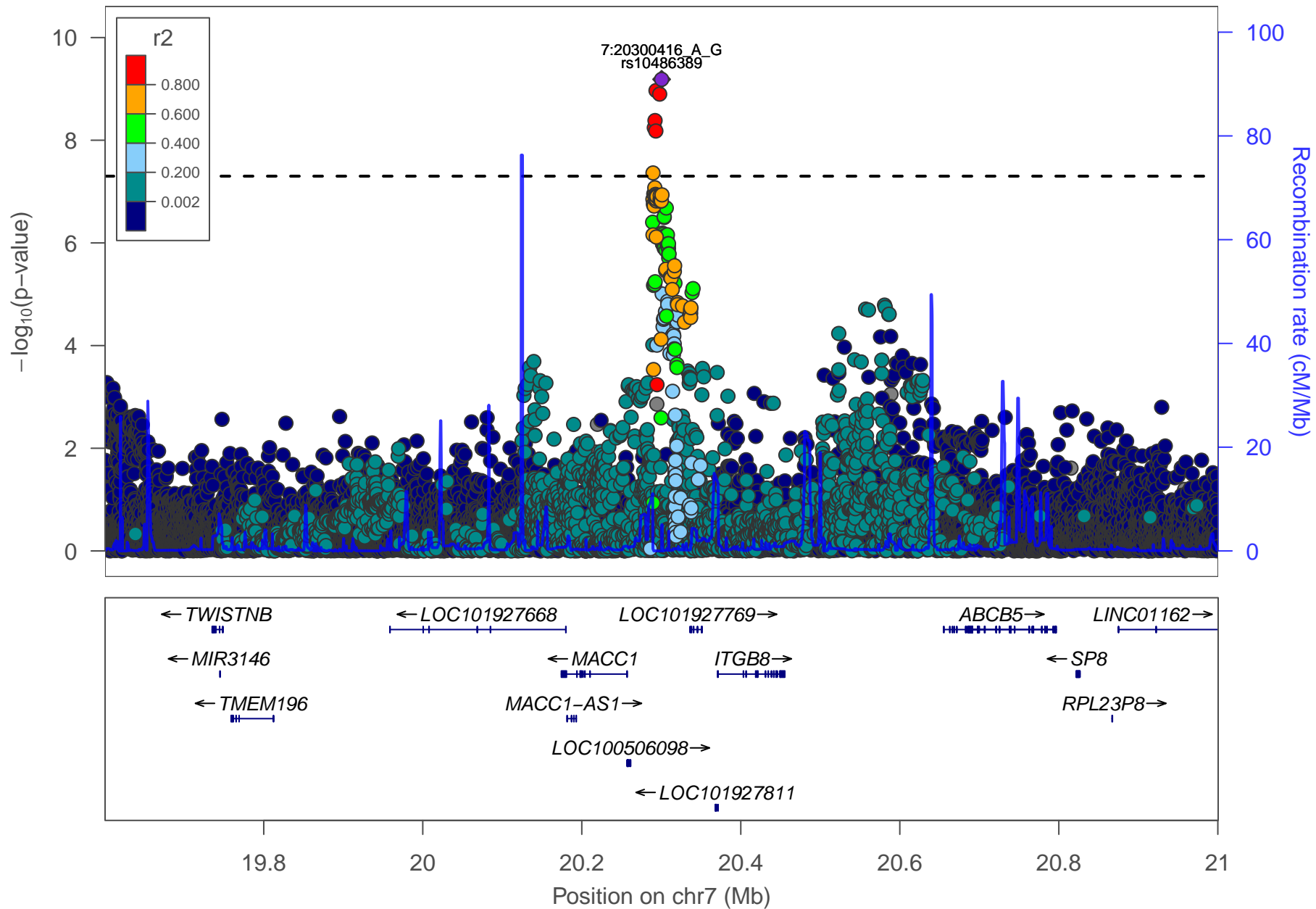

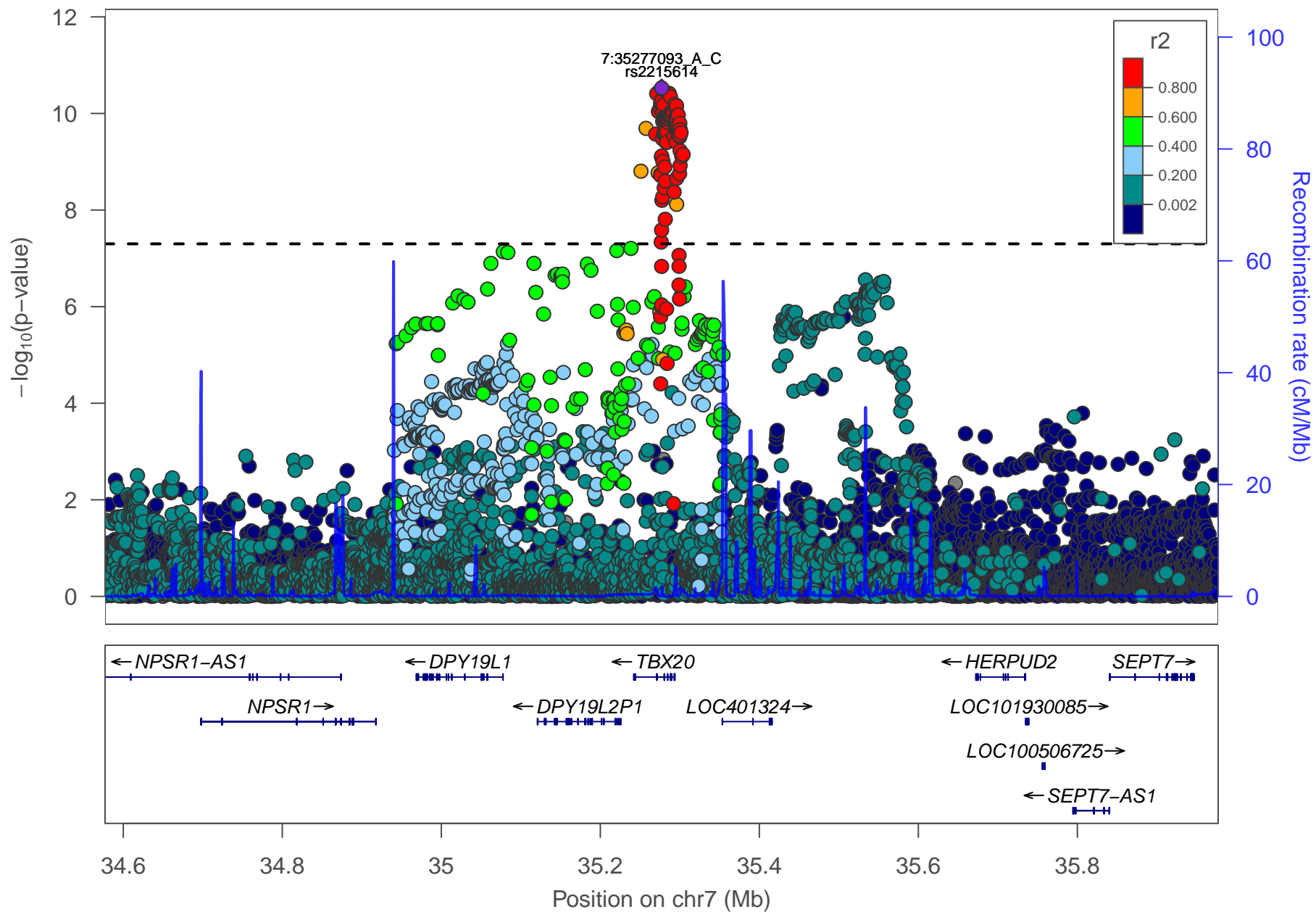

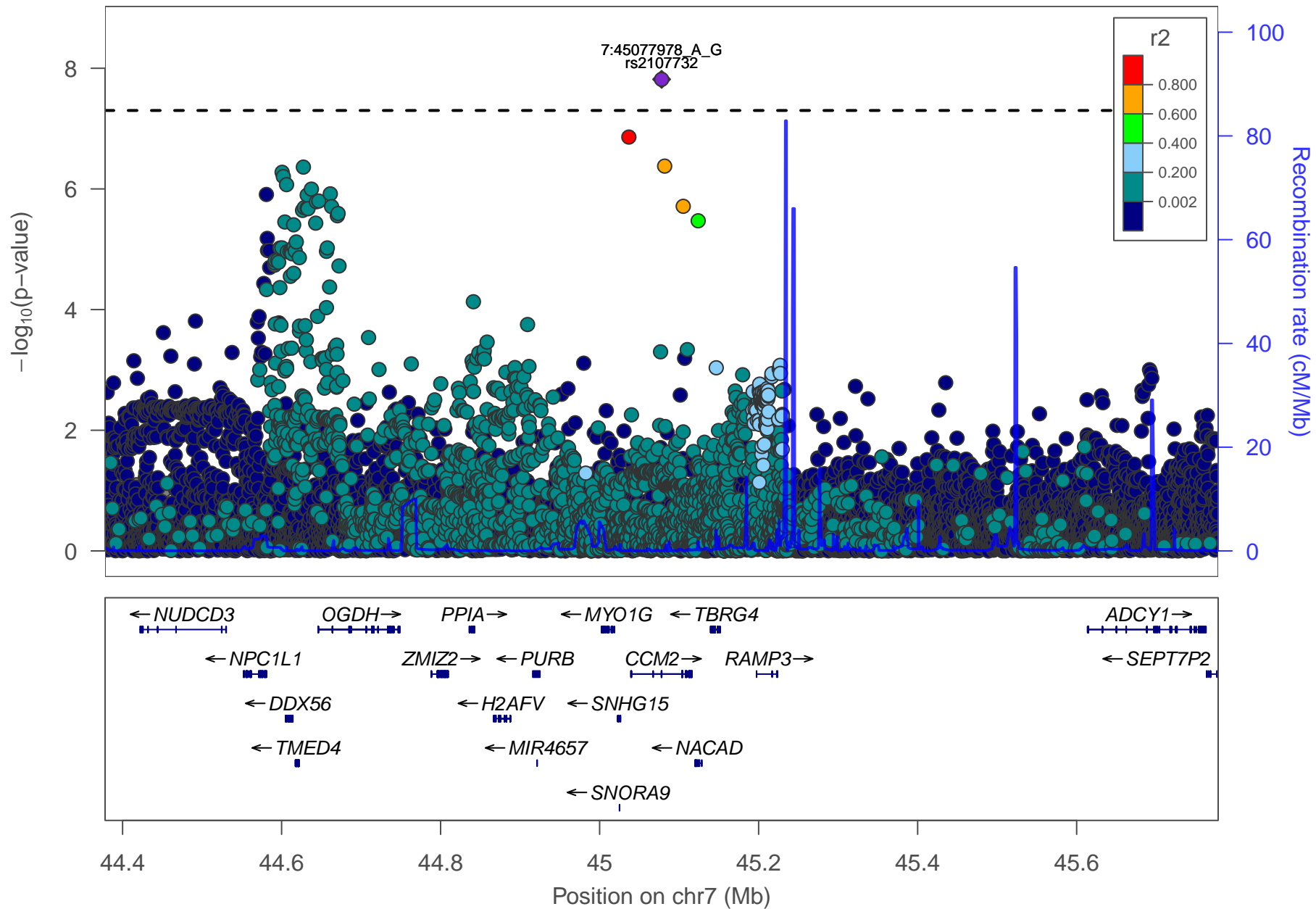

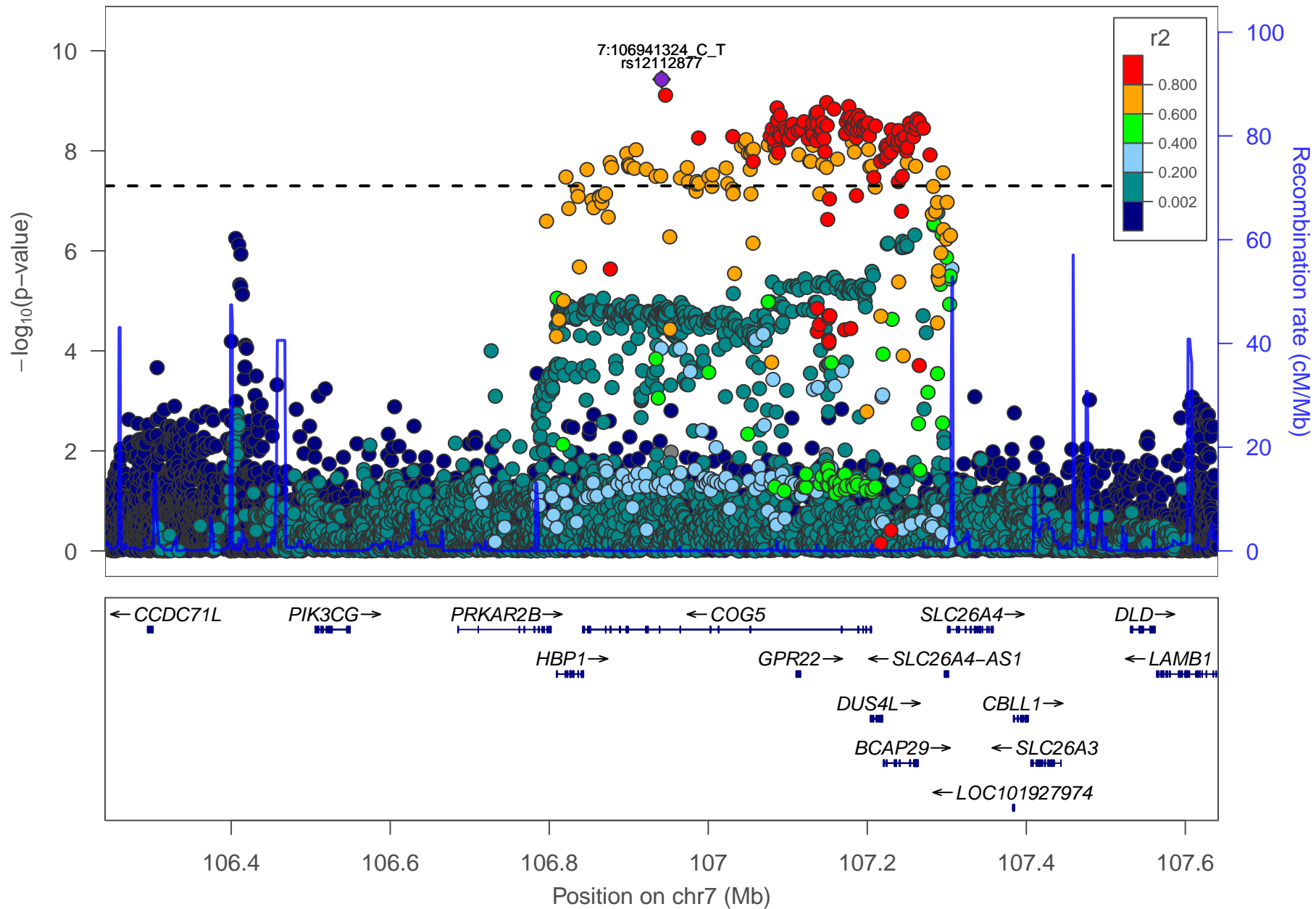

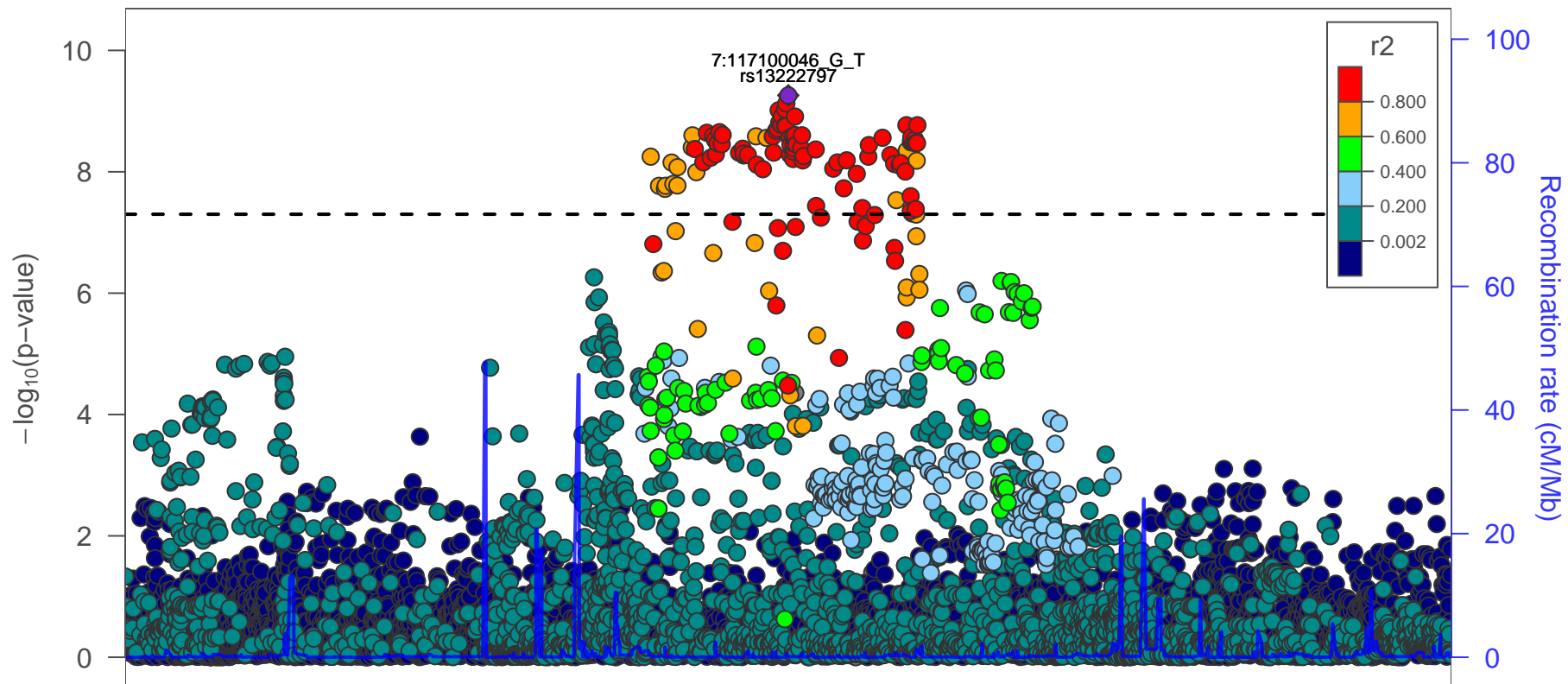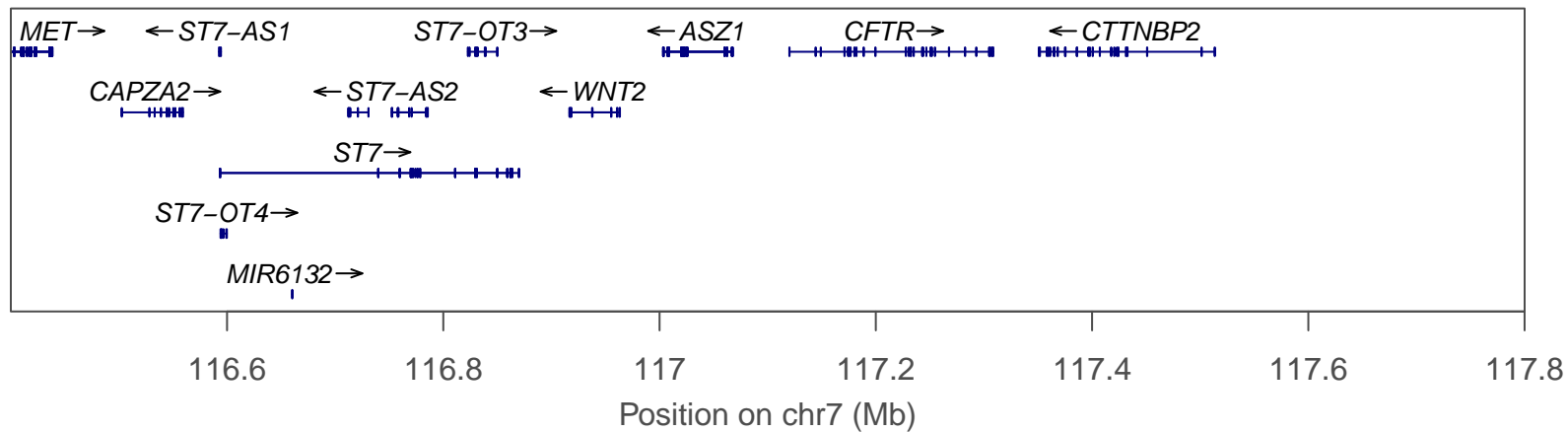

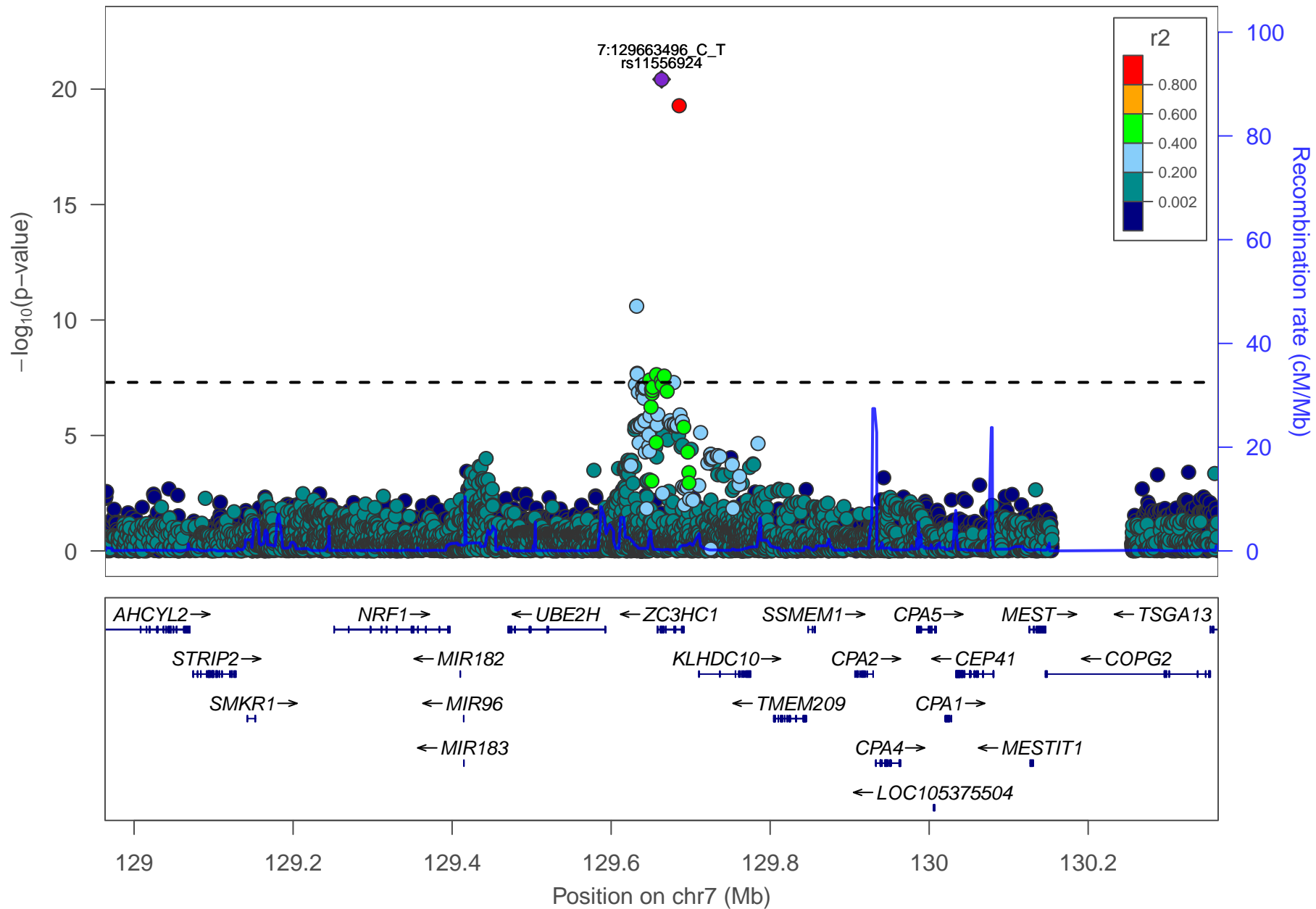

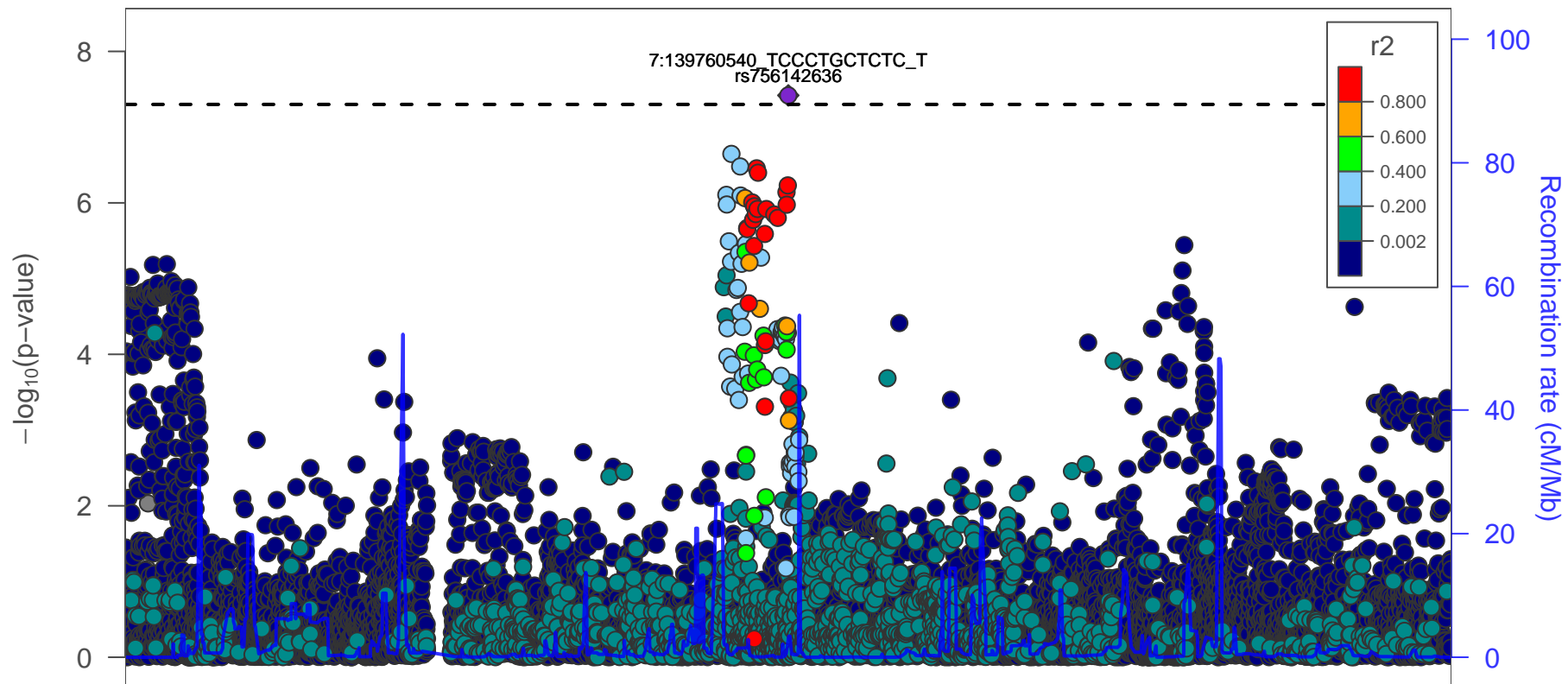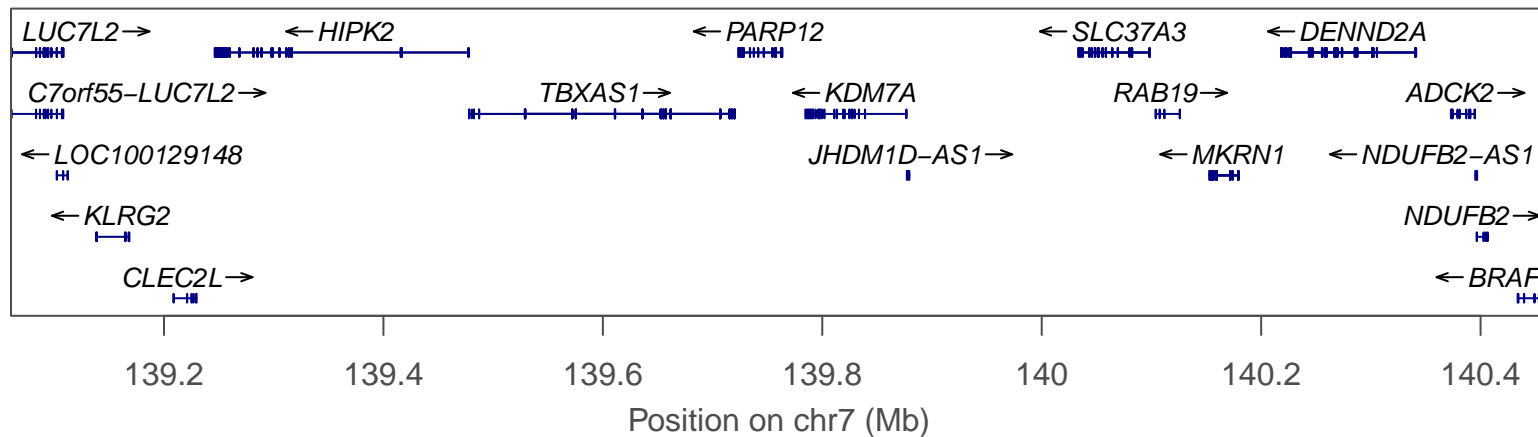

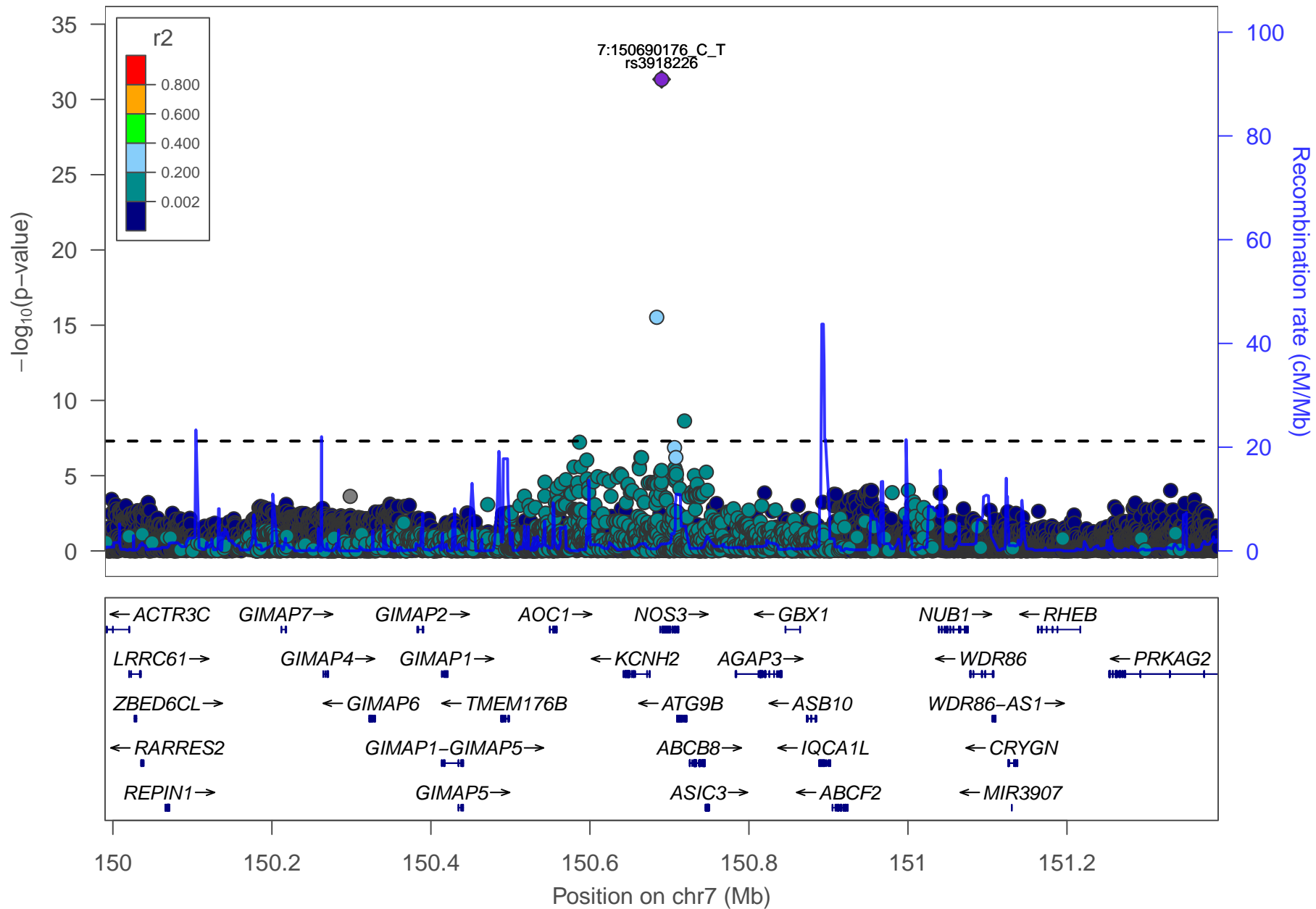

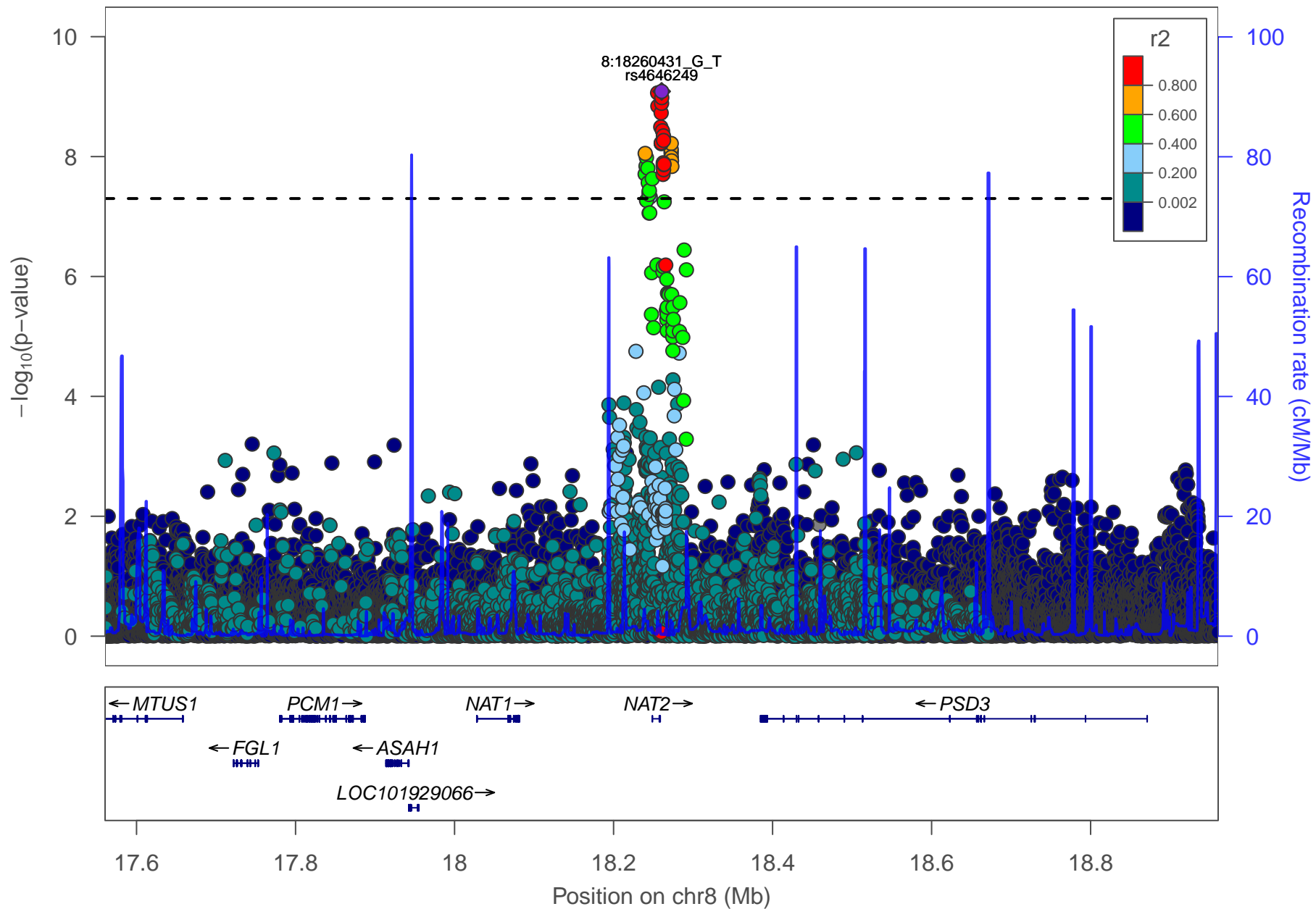

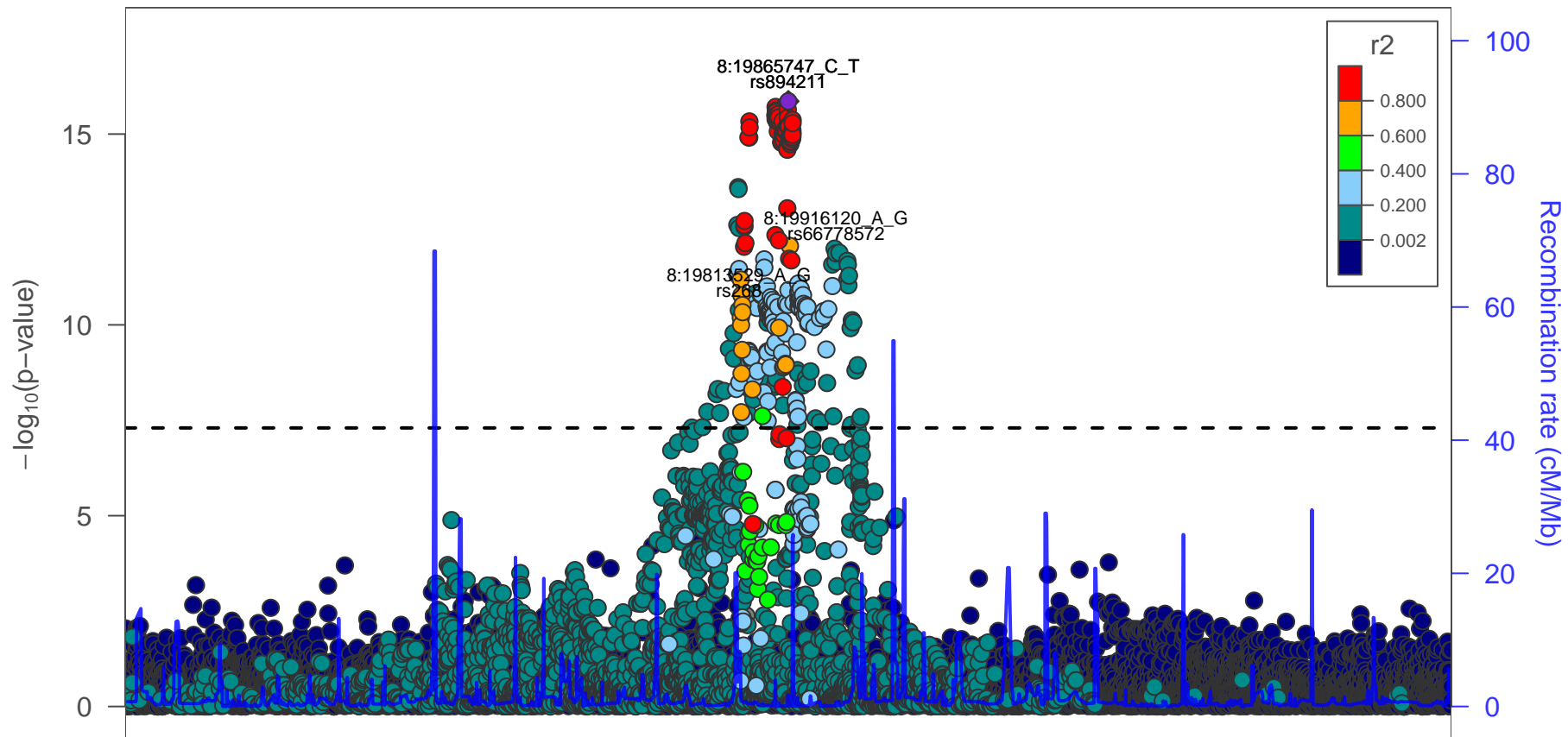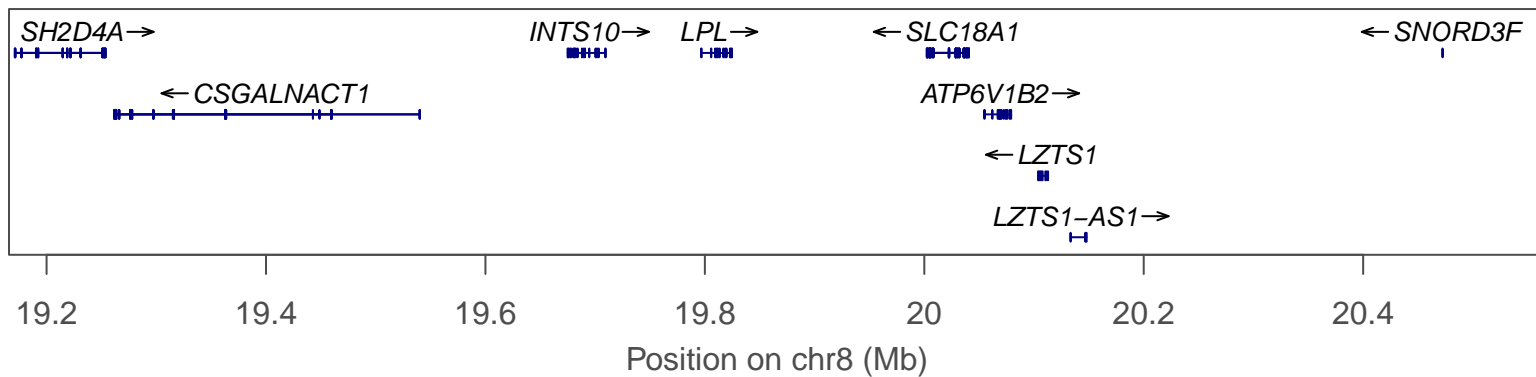

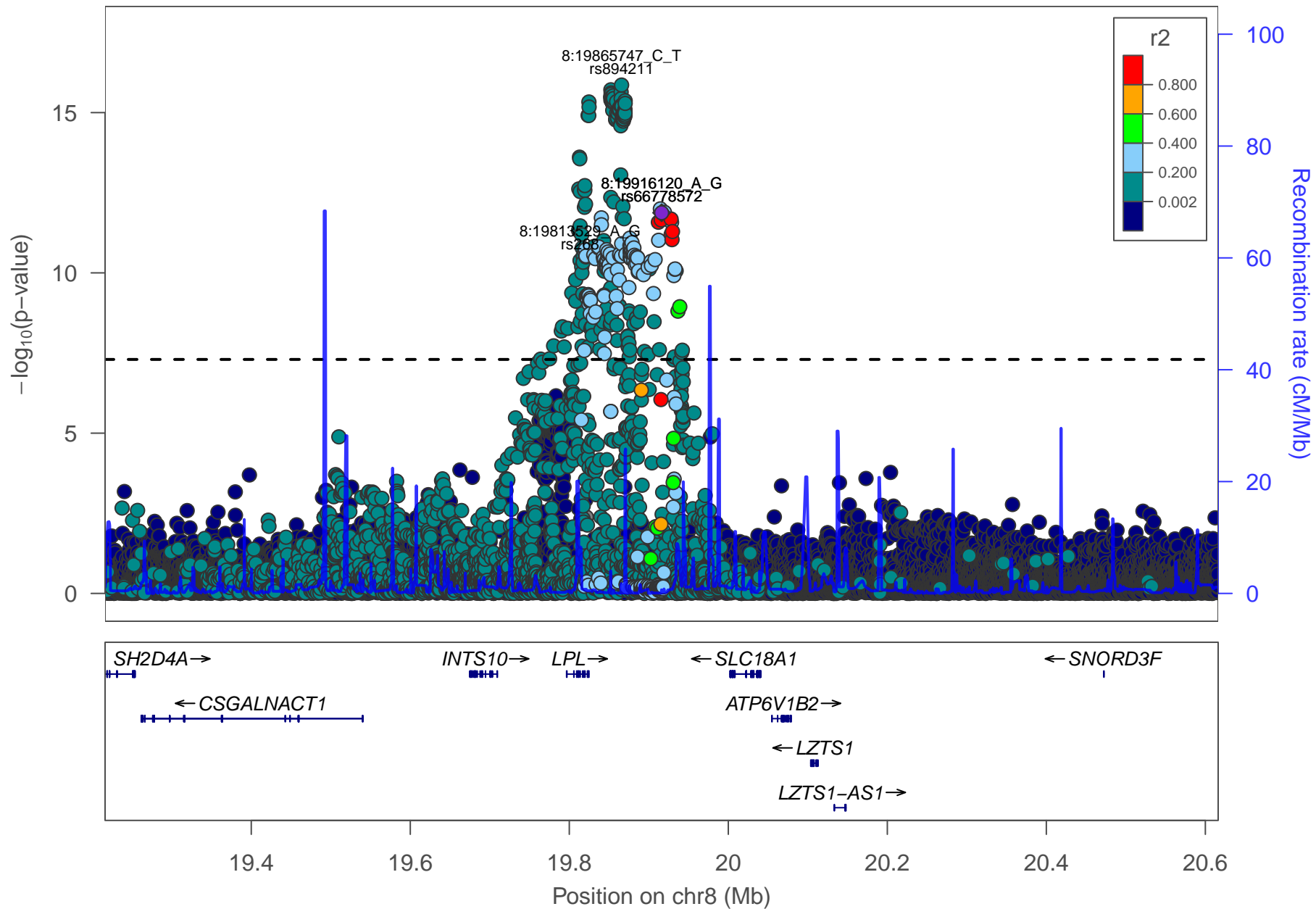

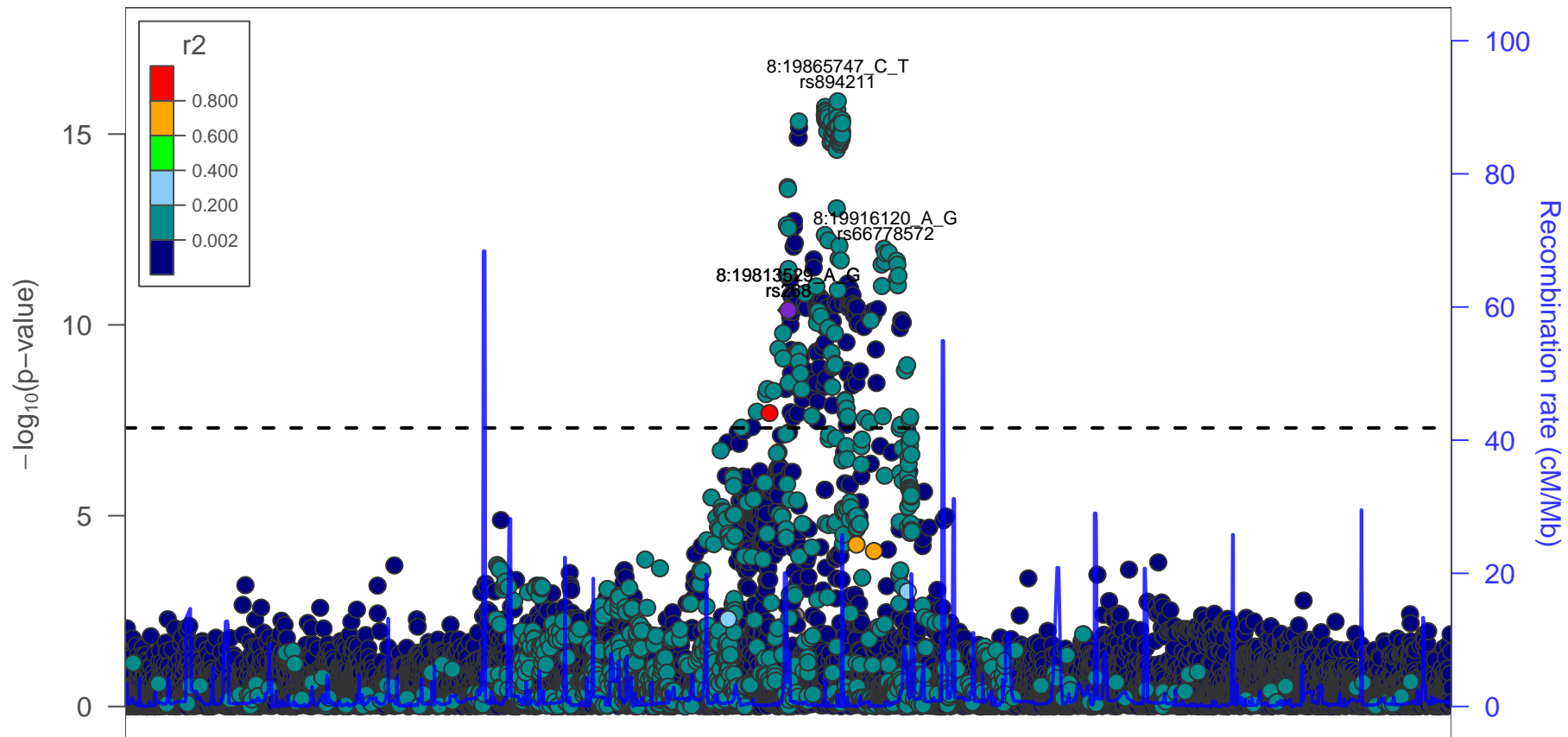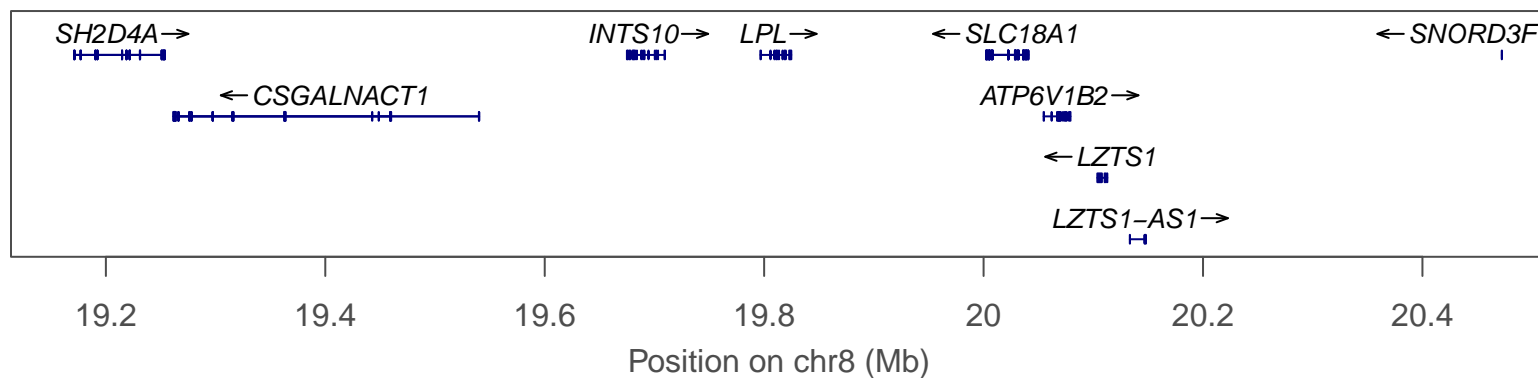

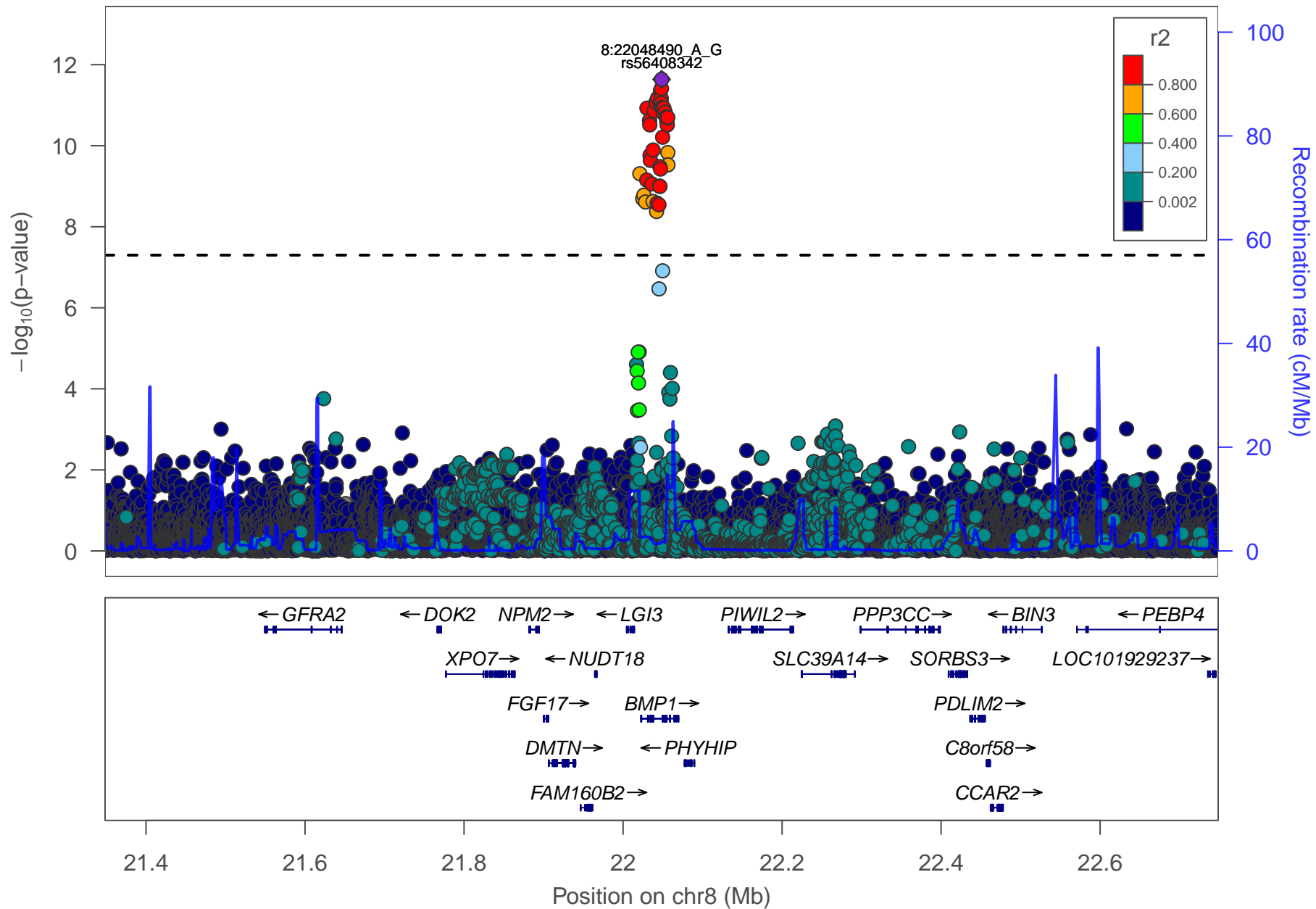

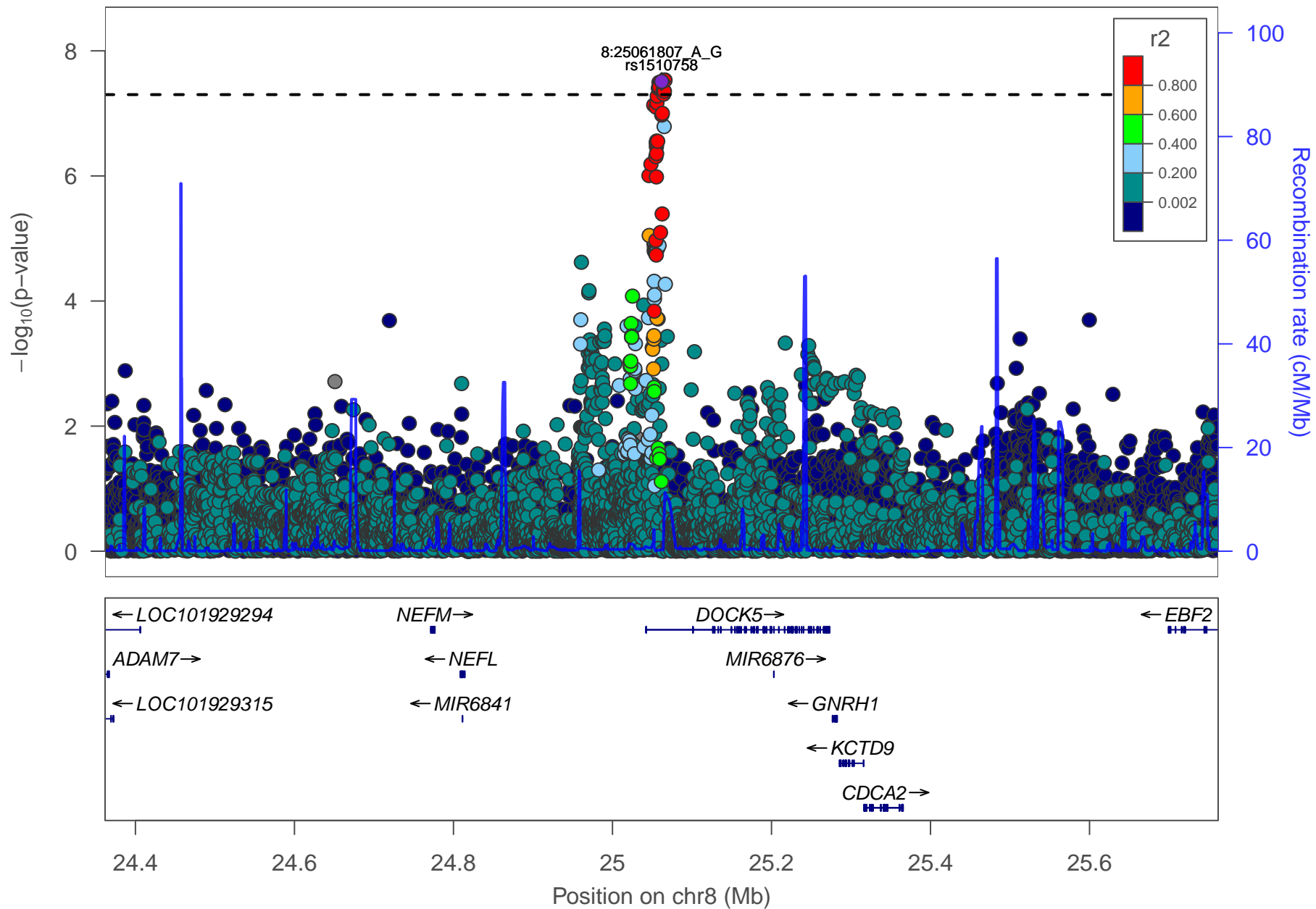

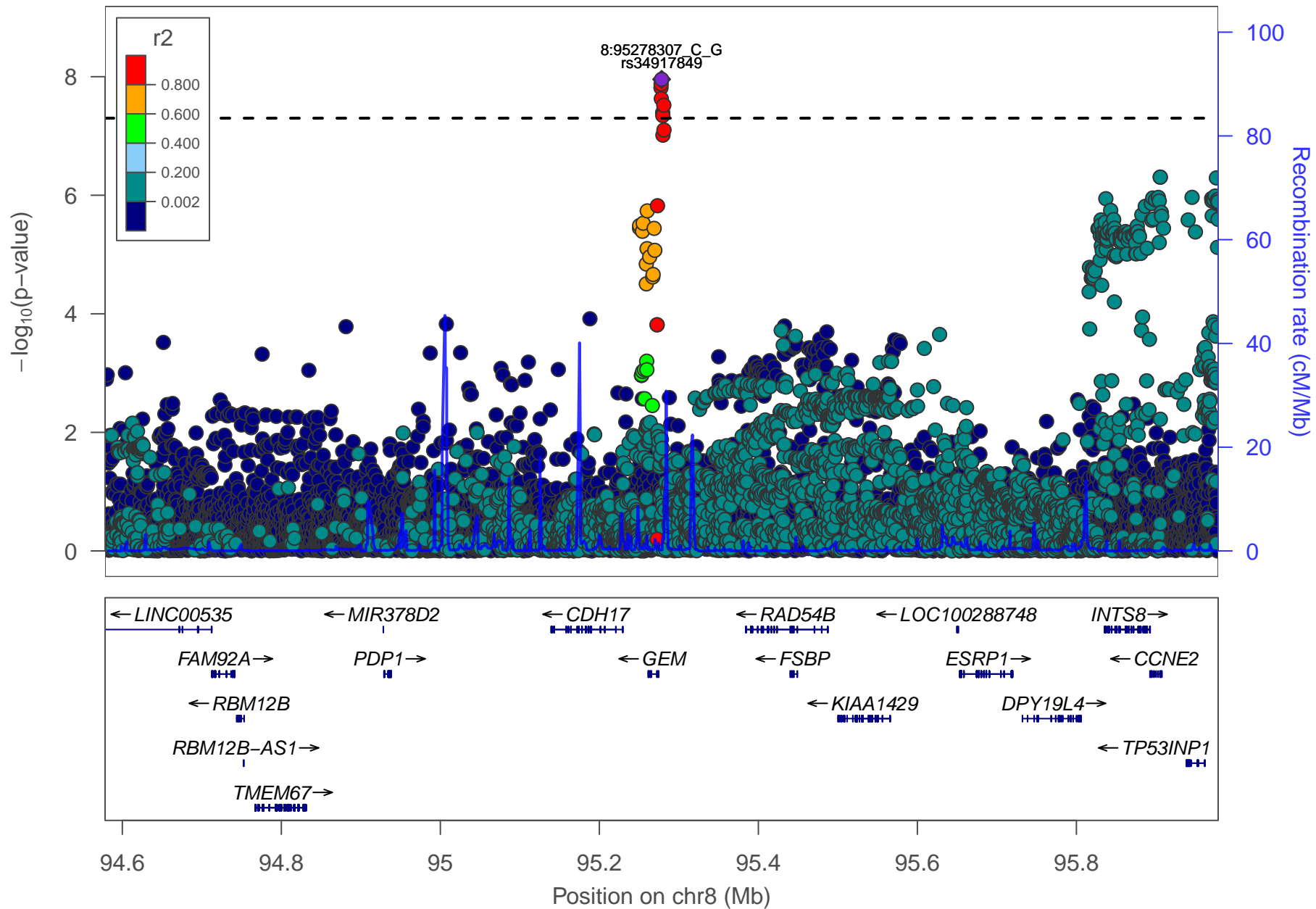

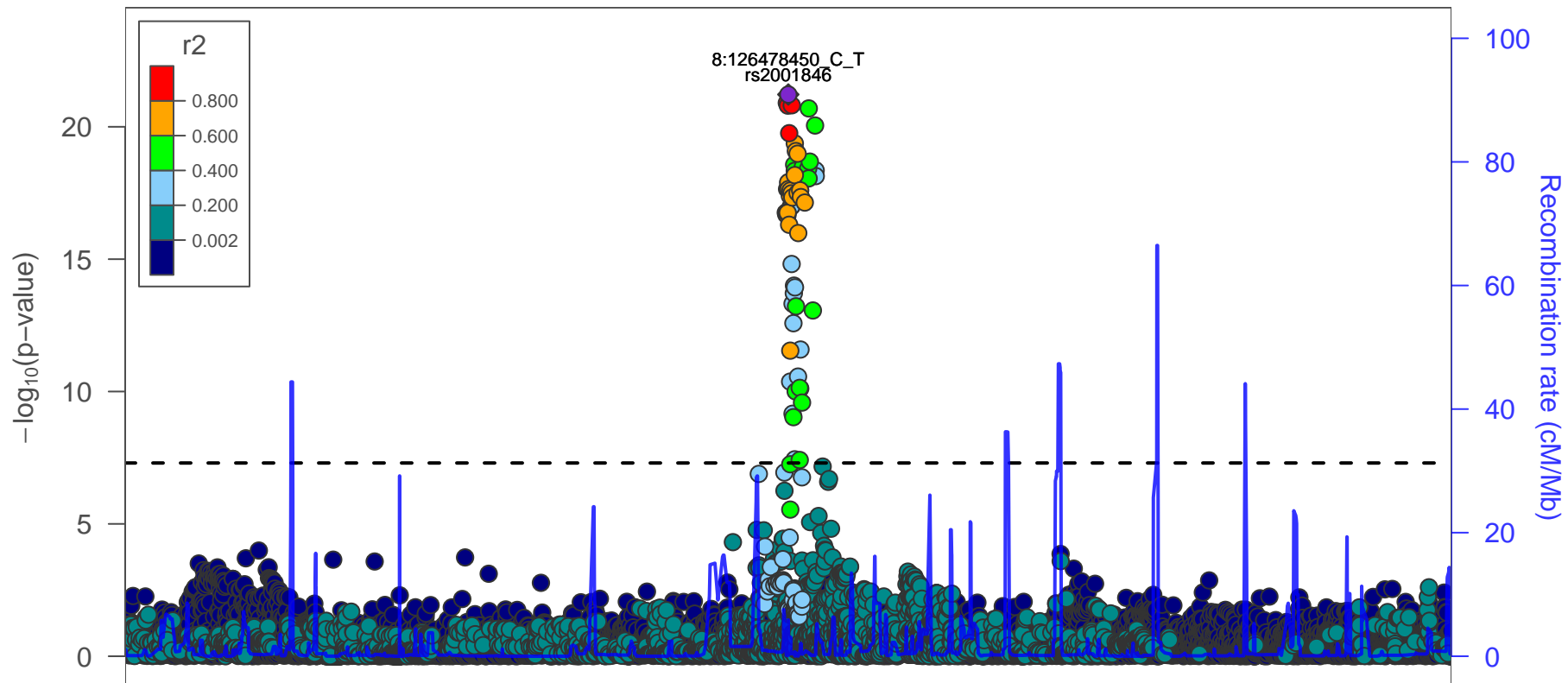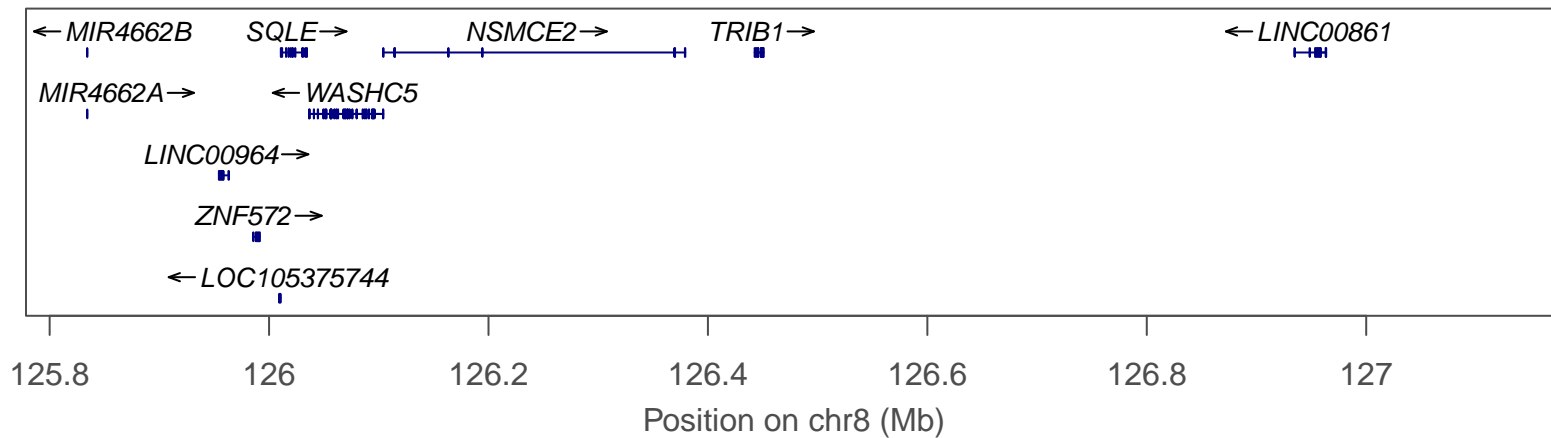

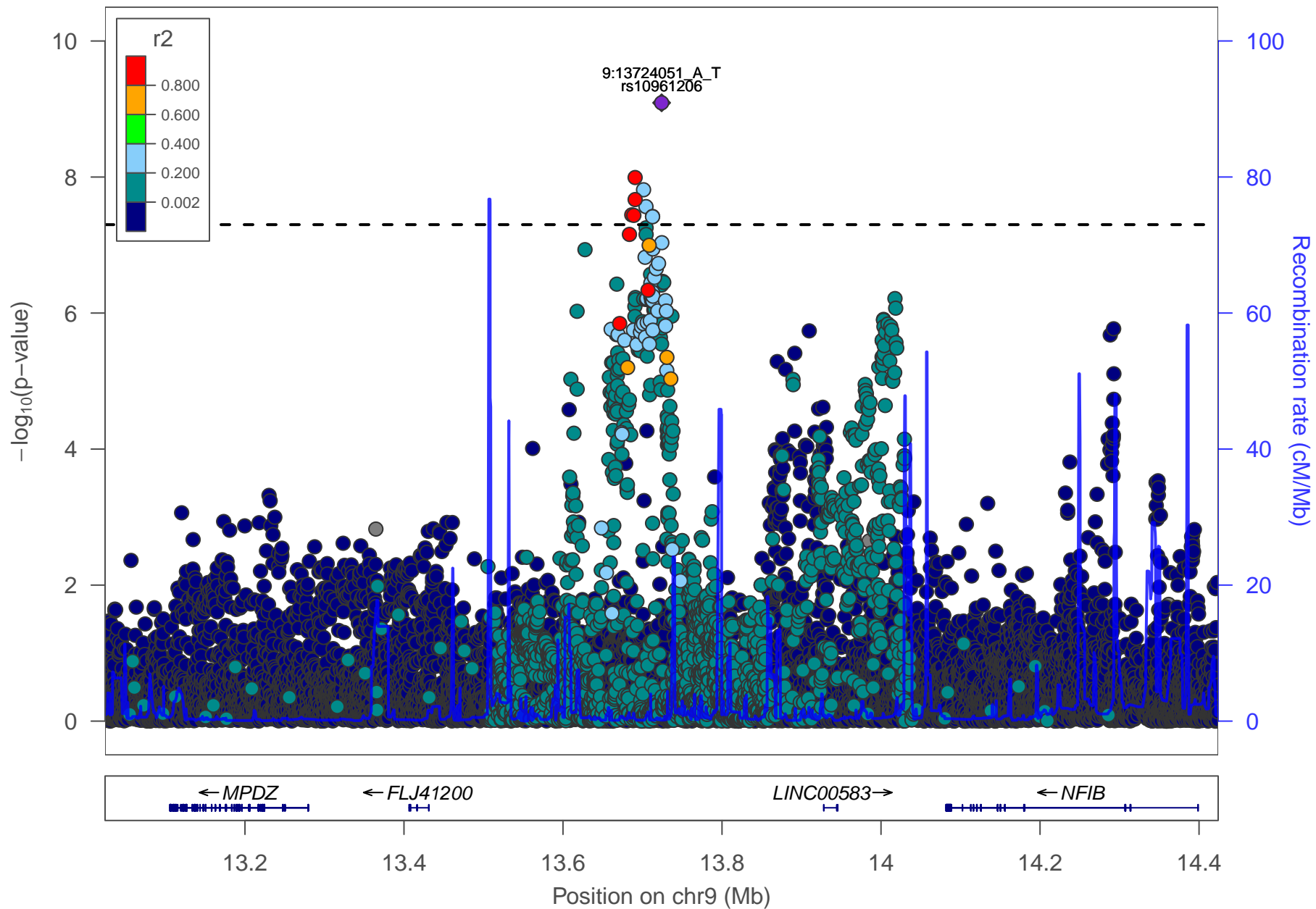

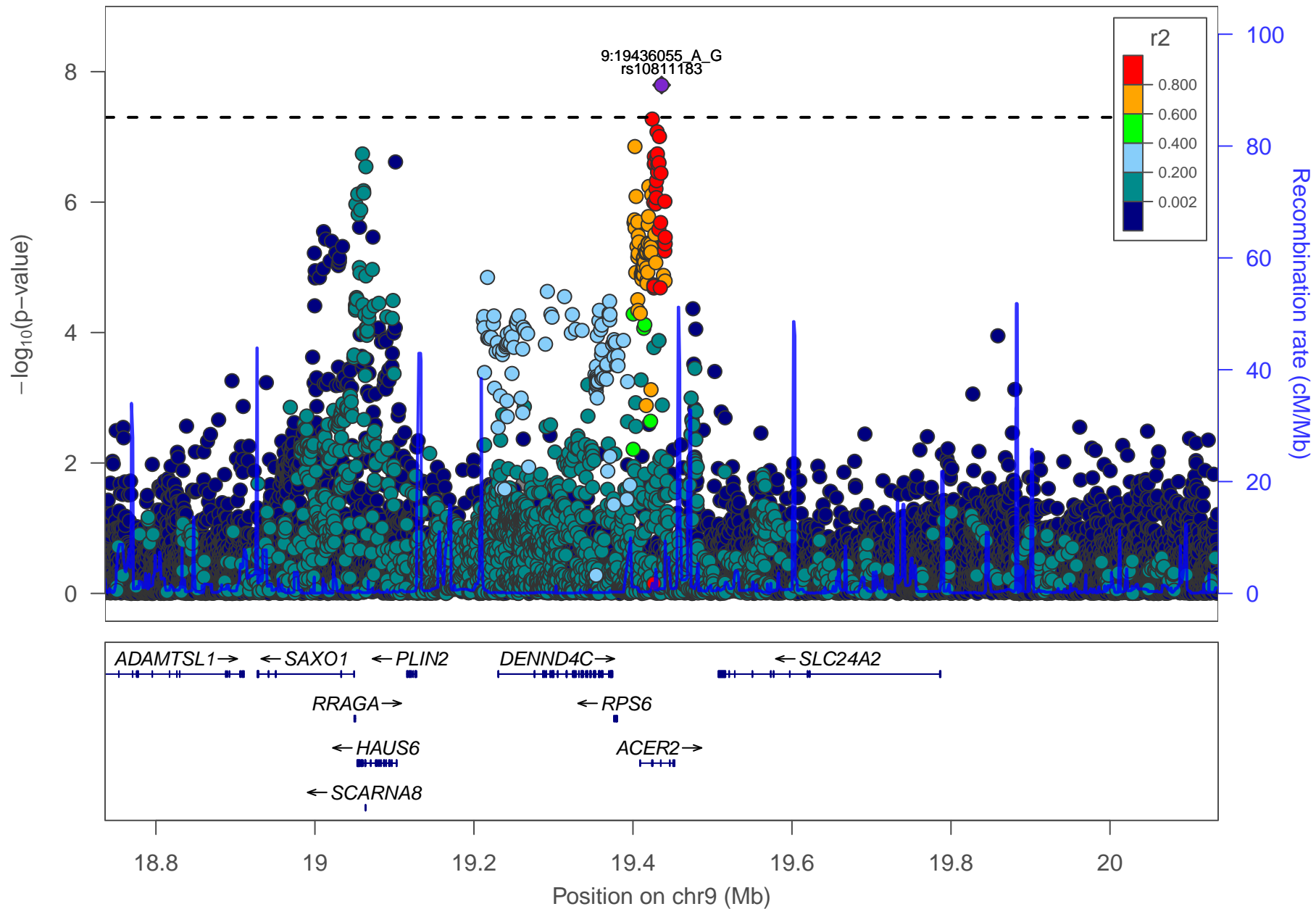

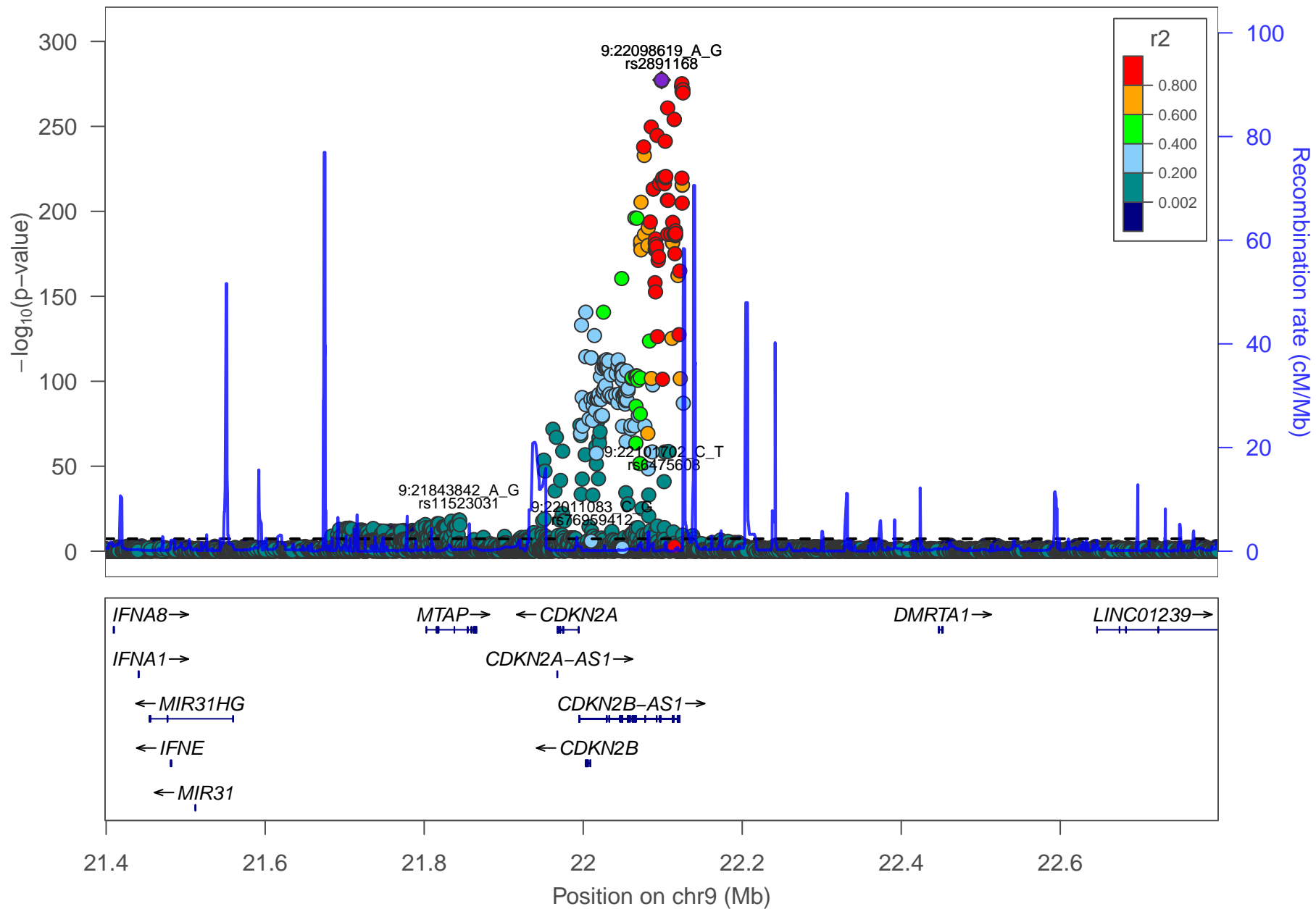

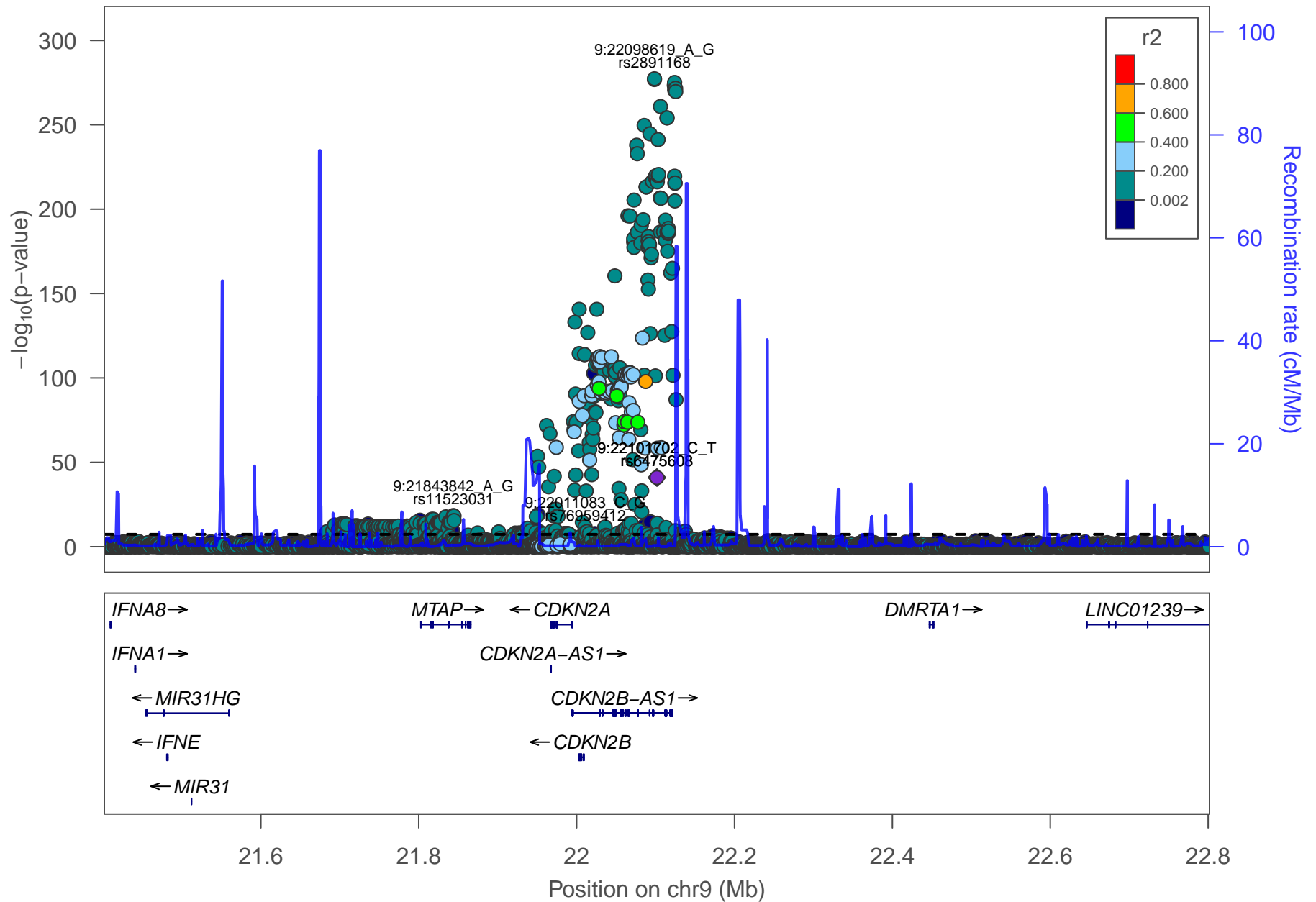

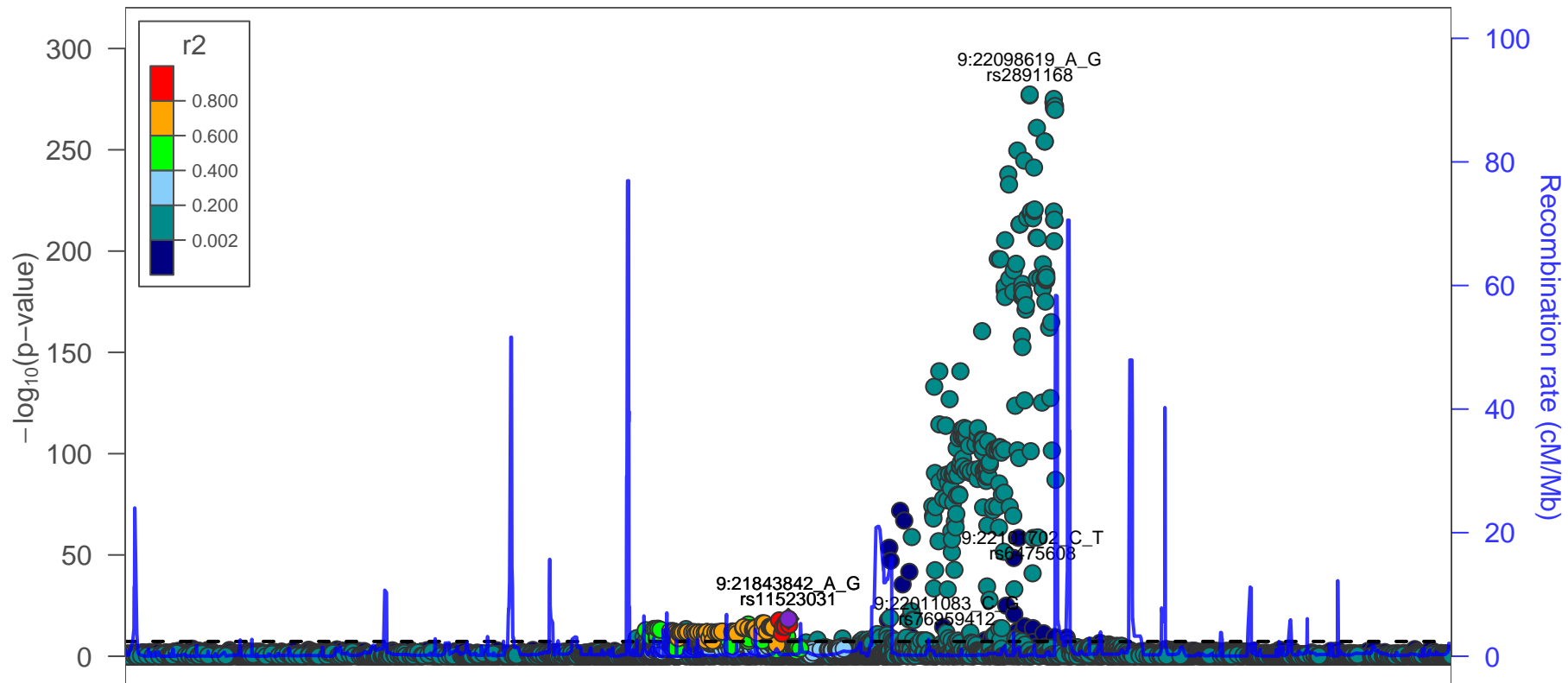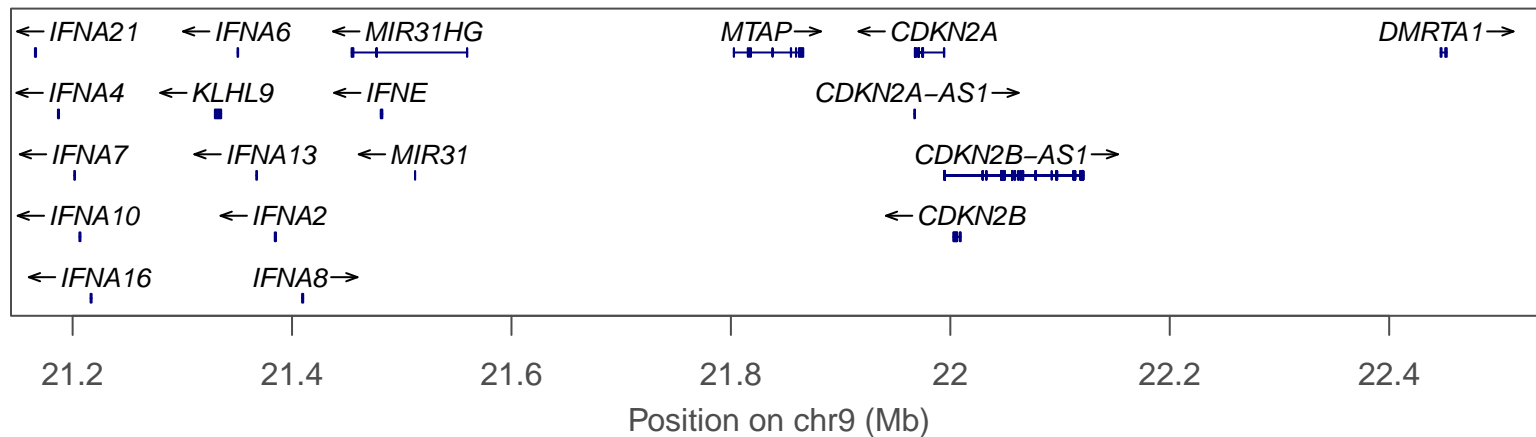

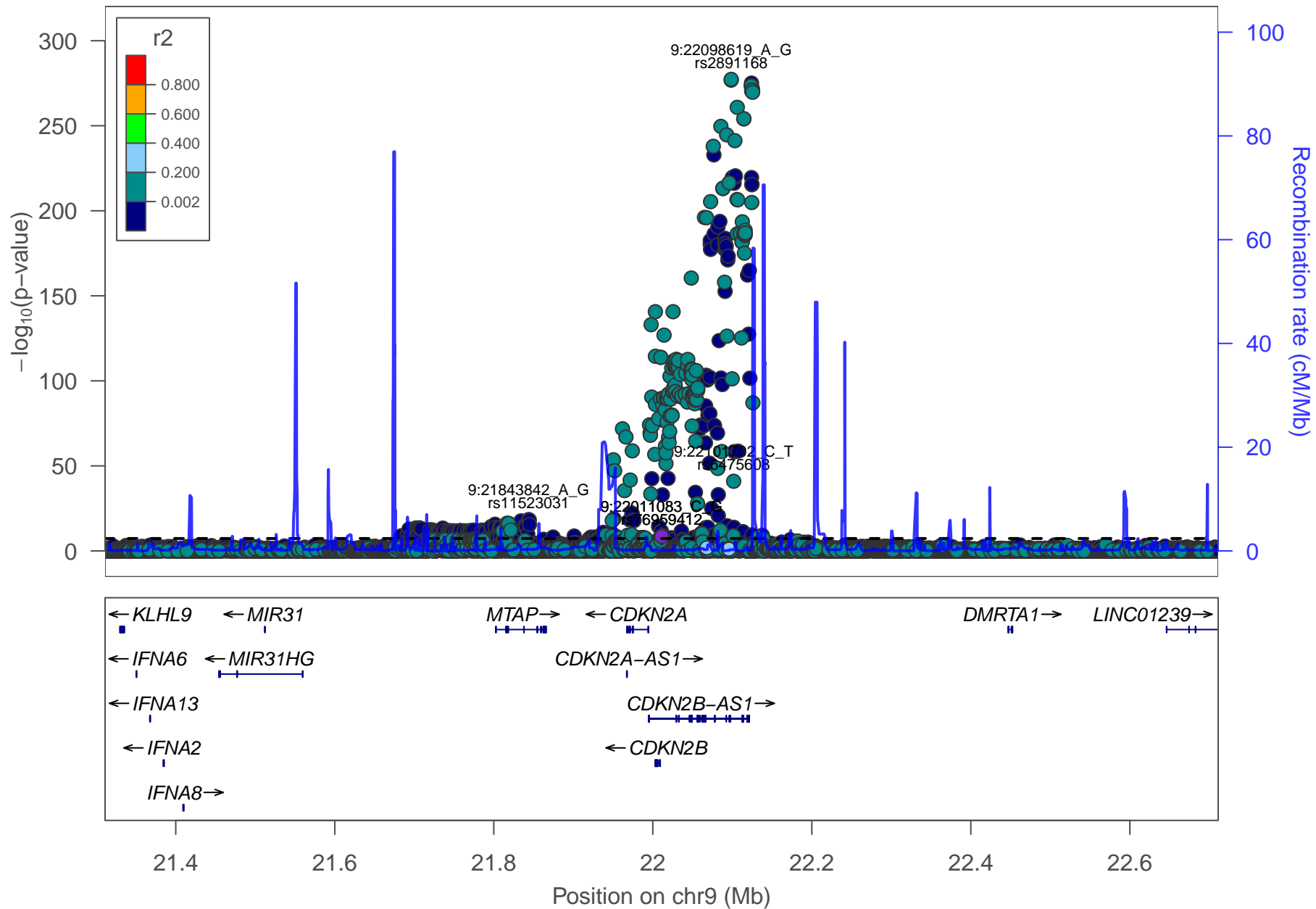

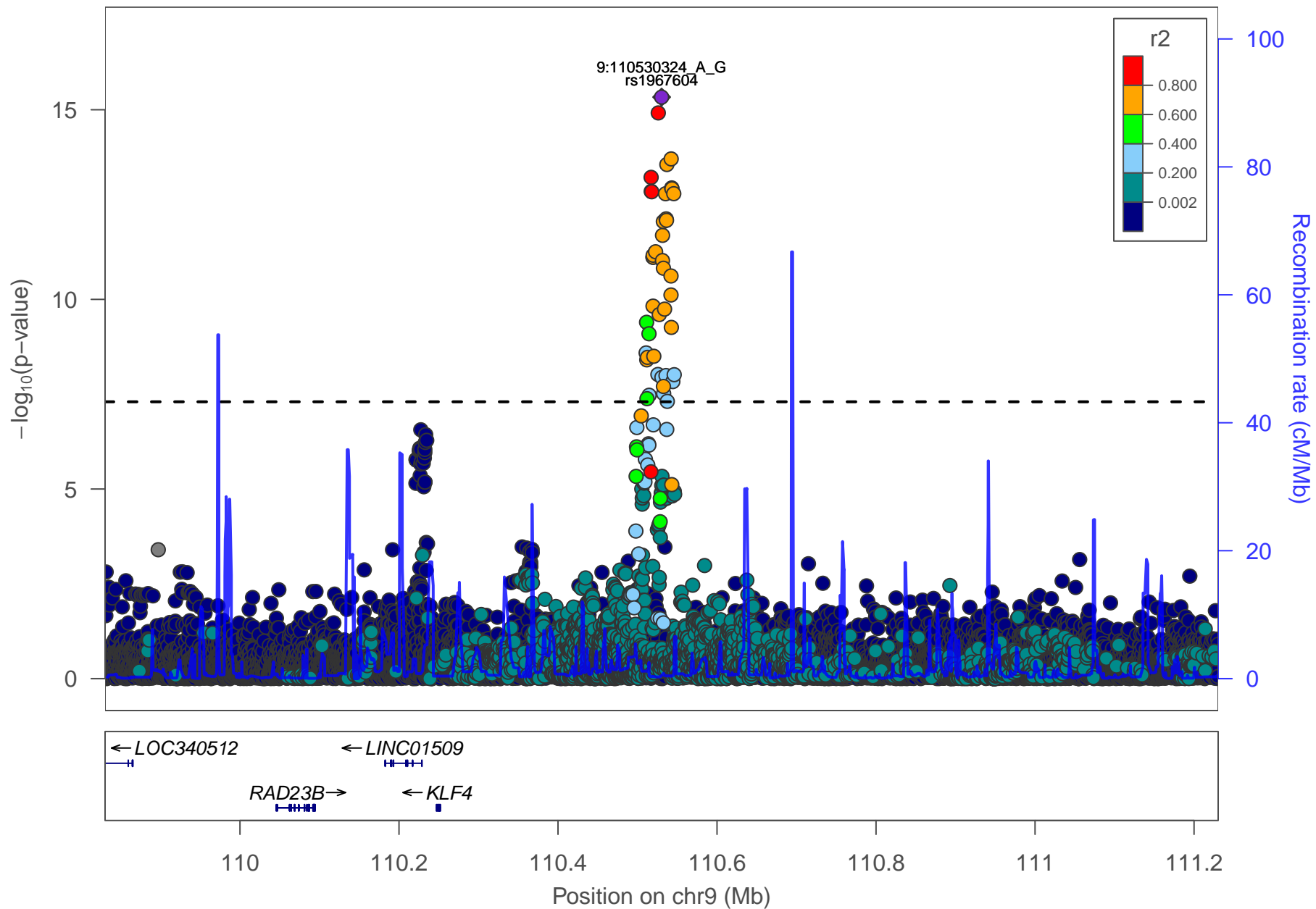

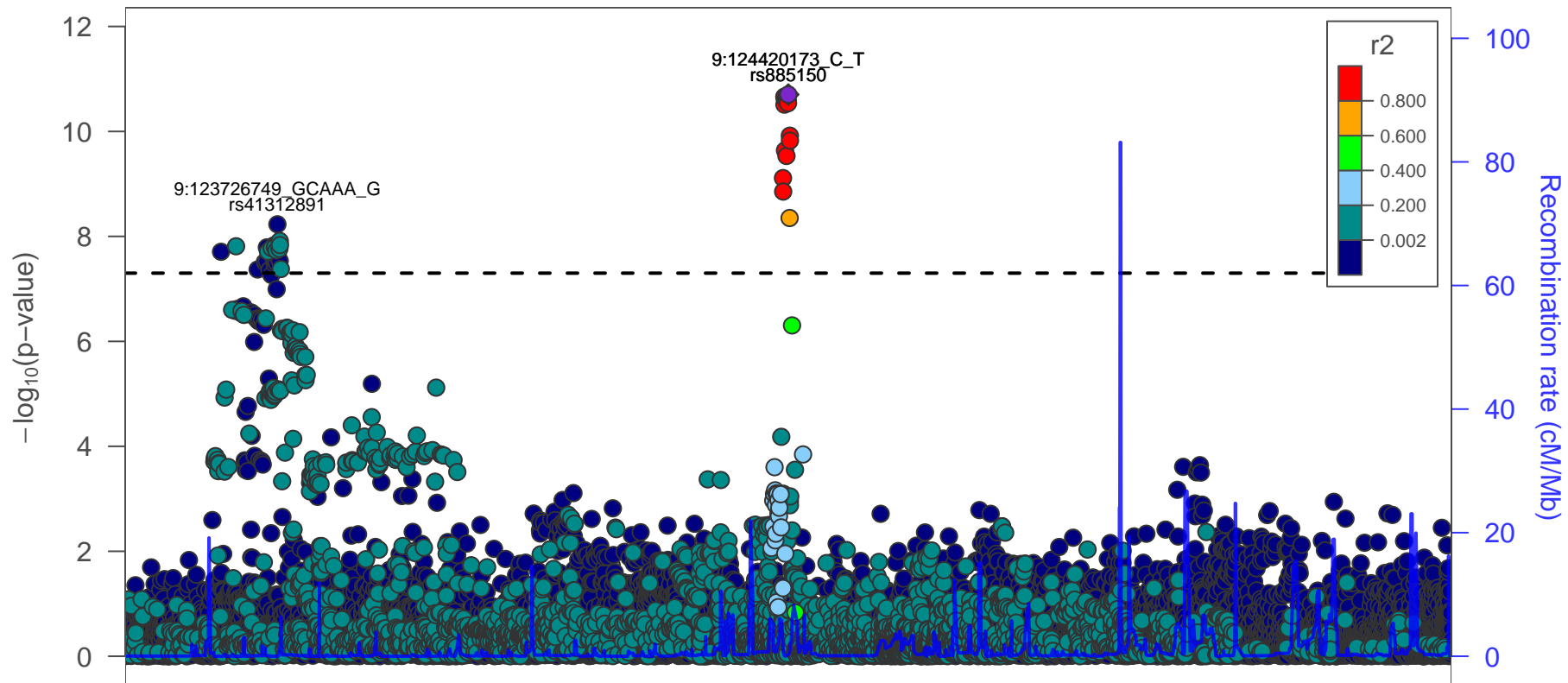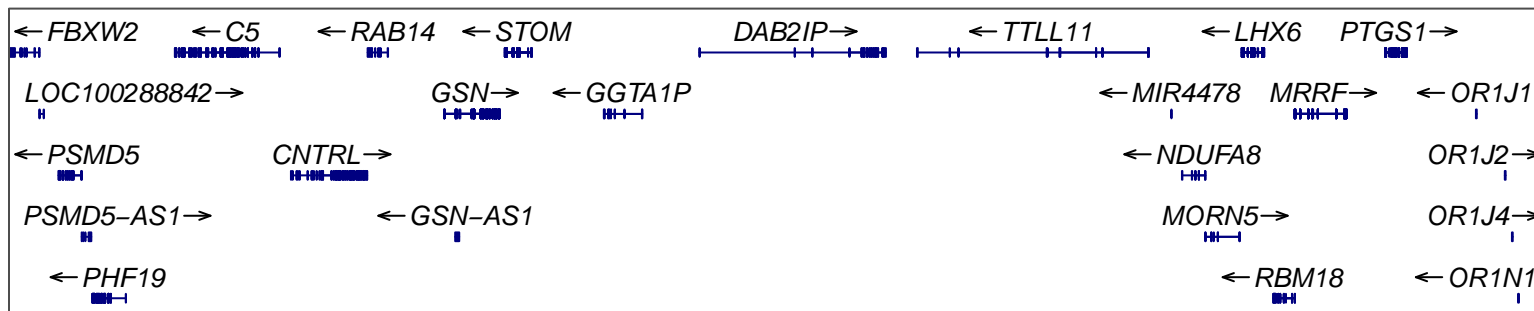

124

124.5

125

Position on chr9 (Mb)

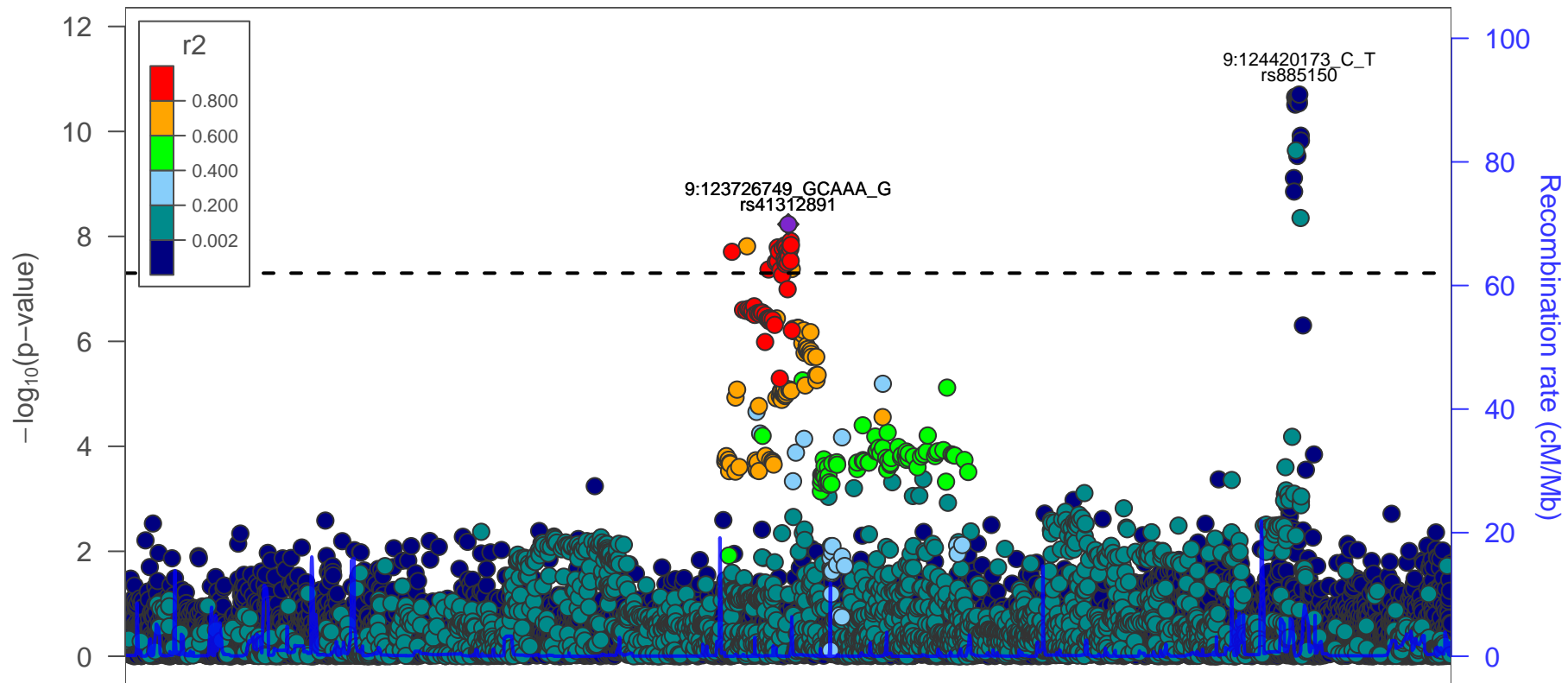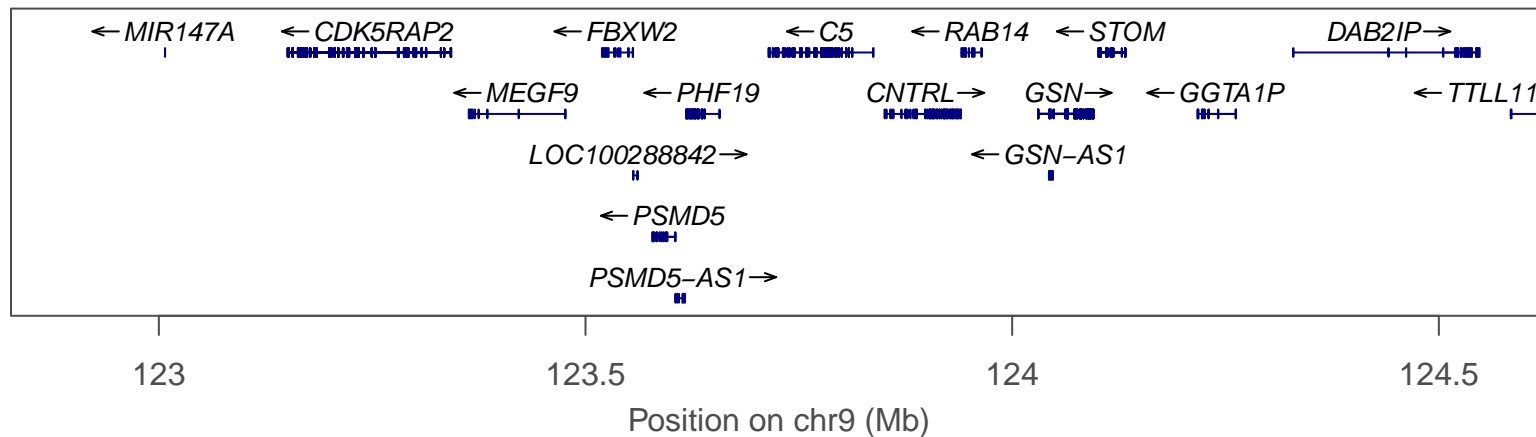

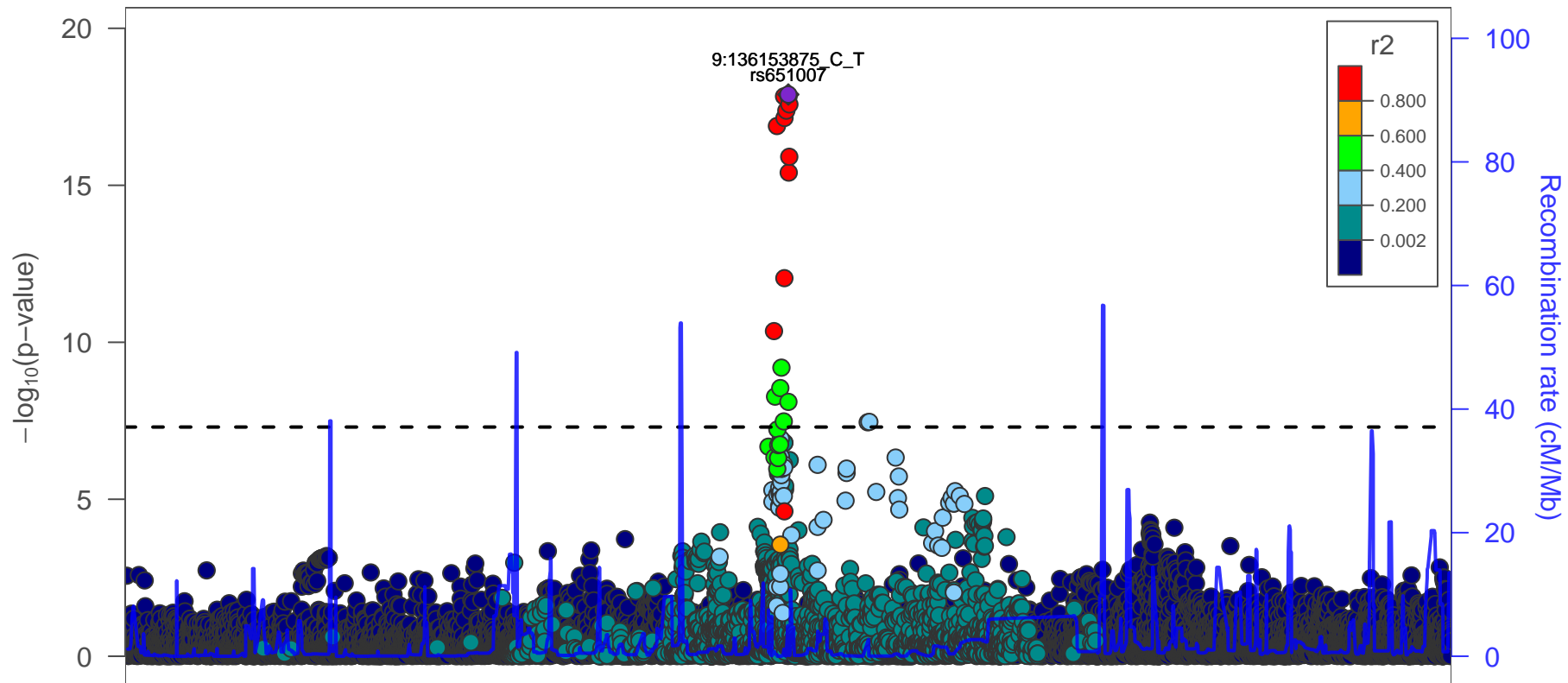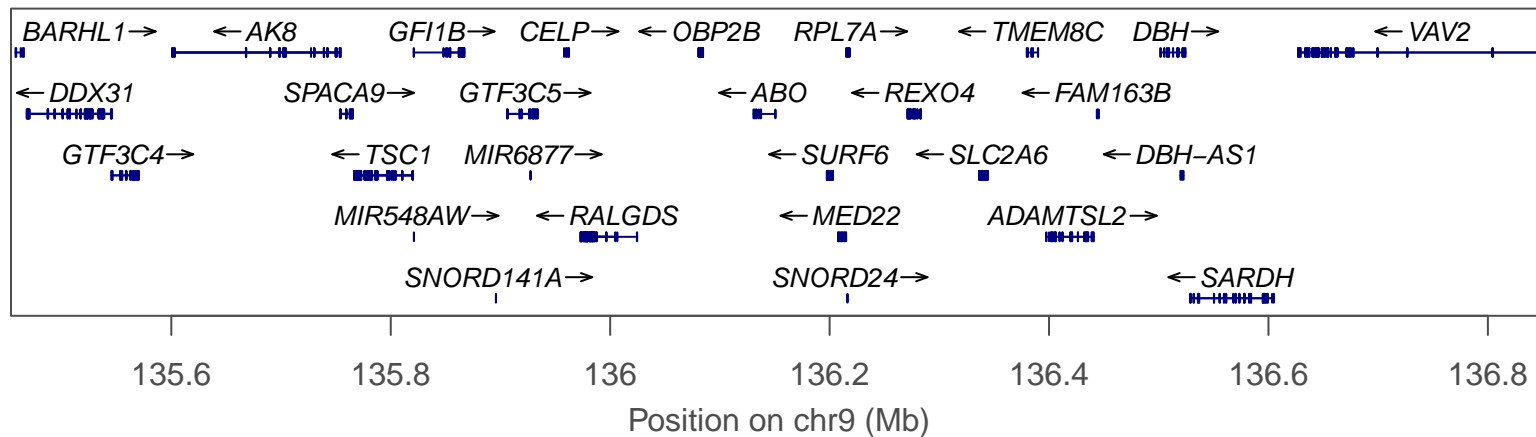

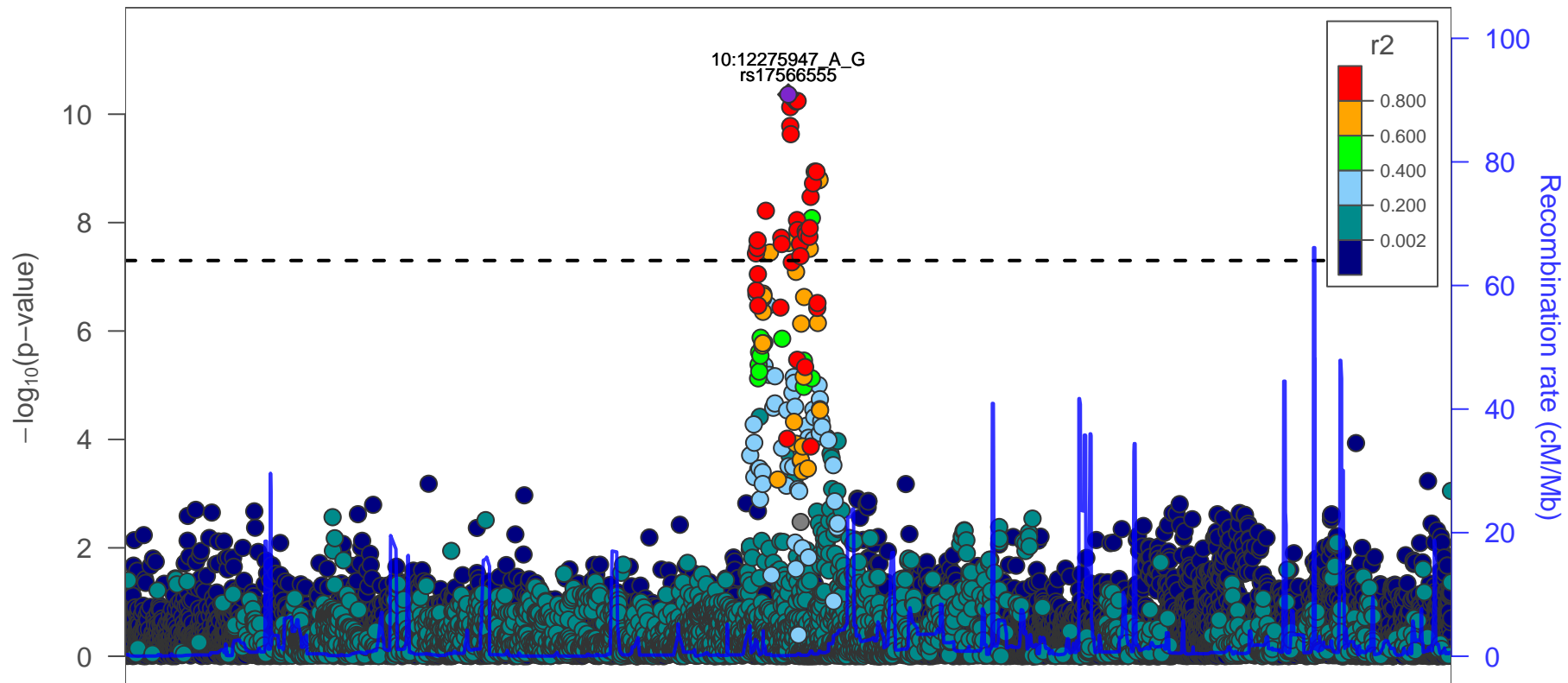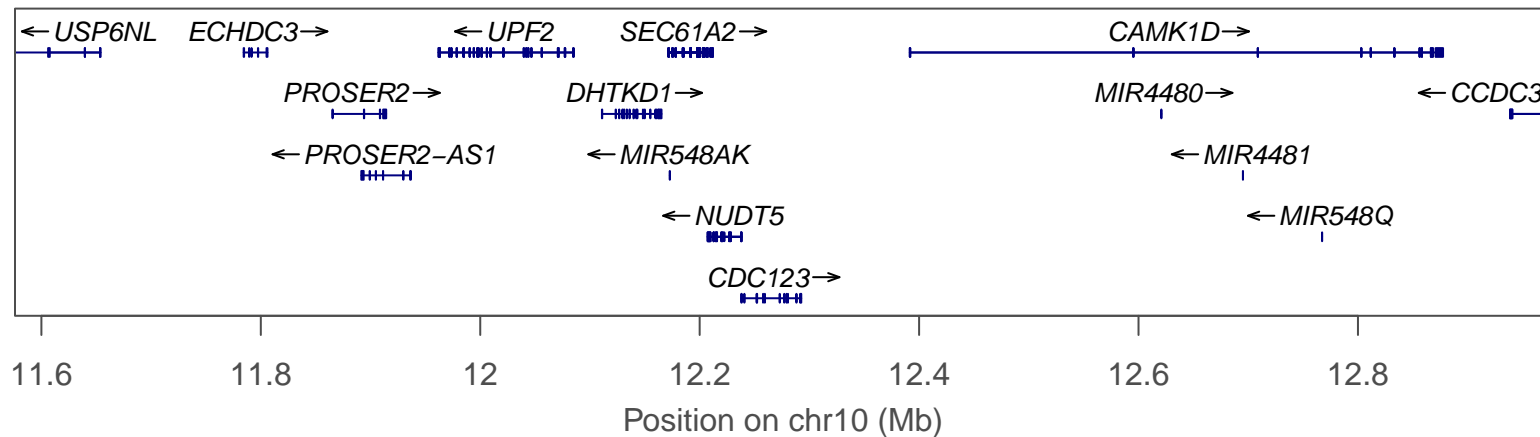

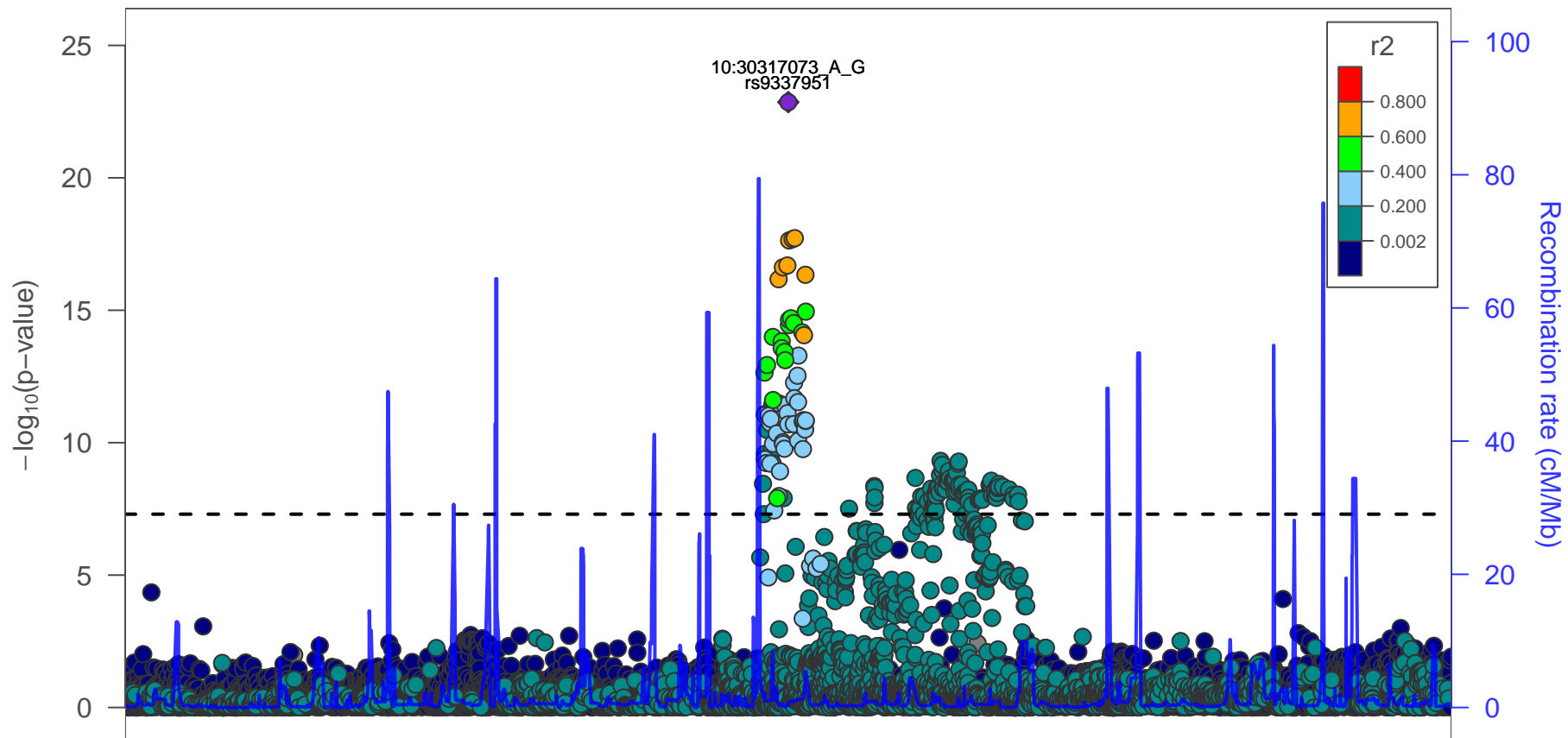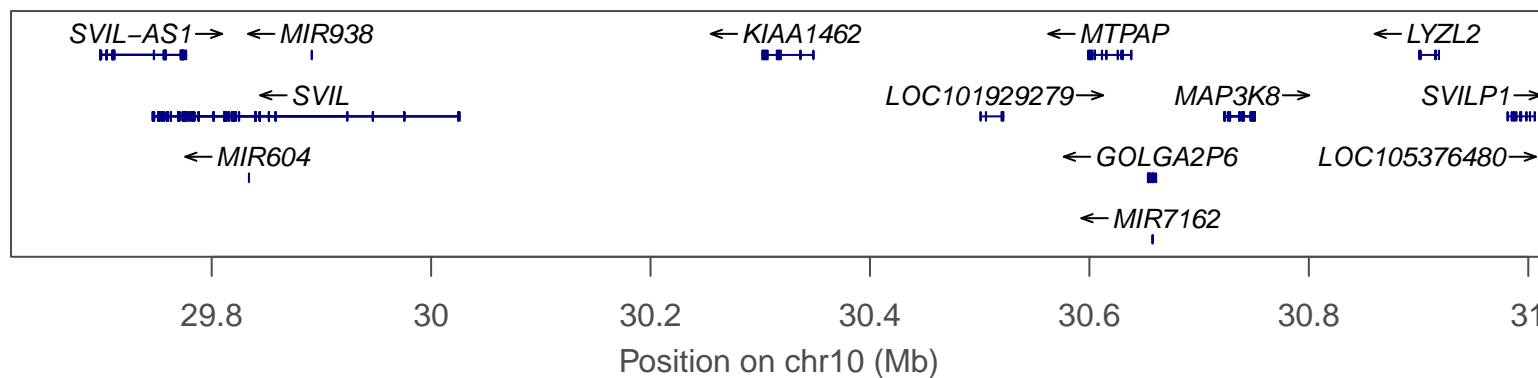

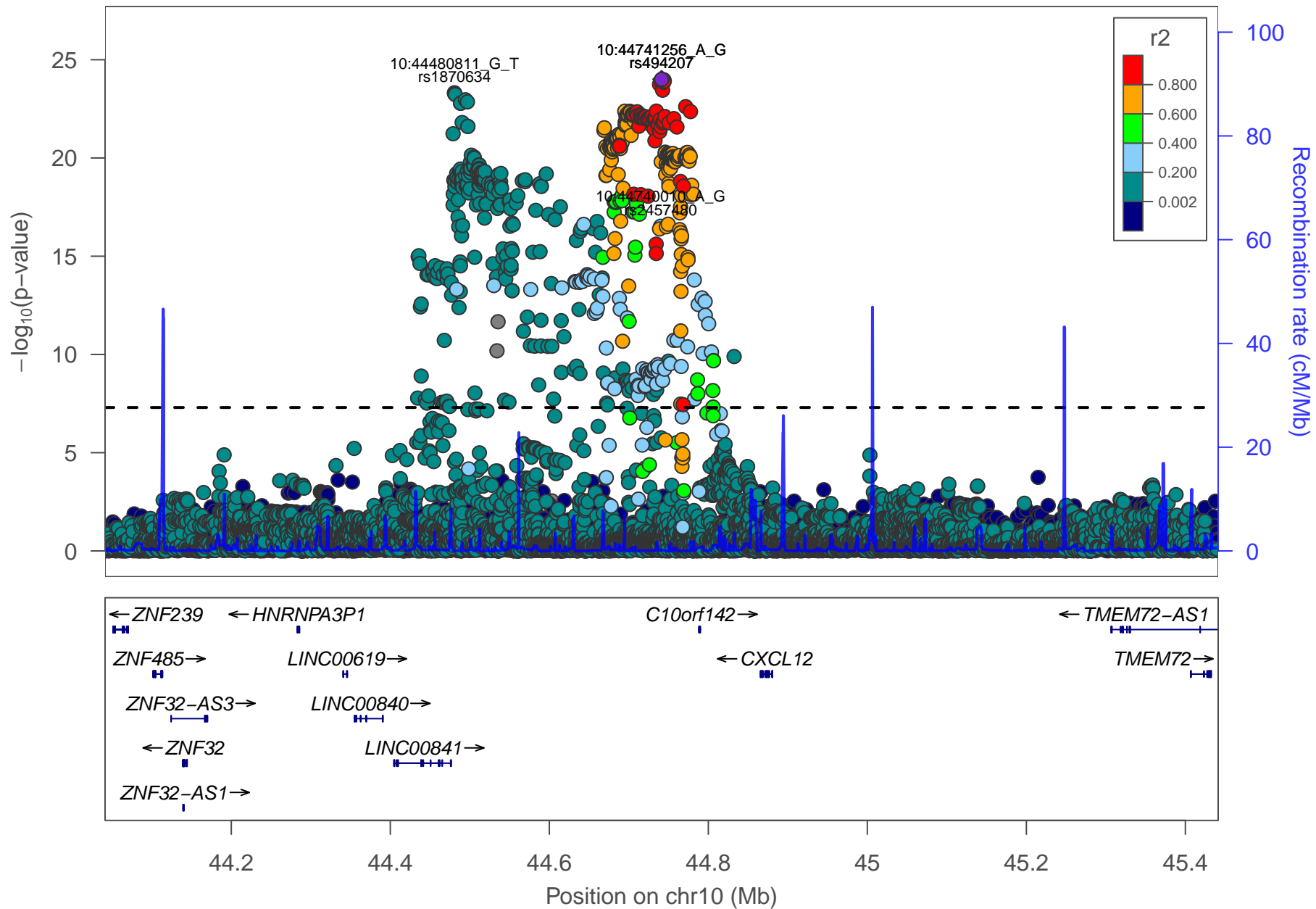

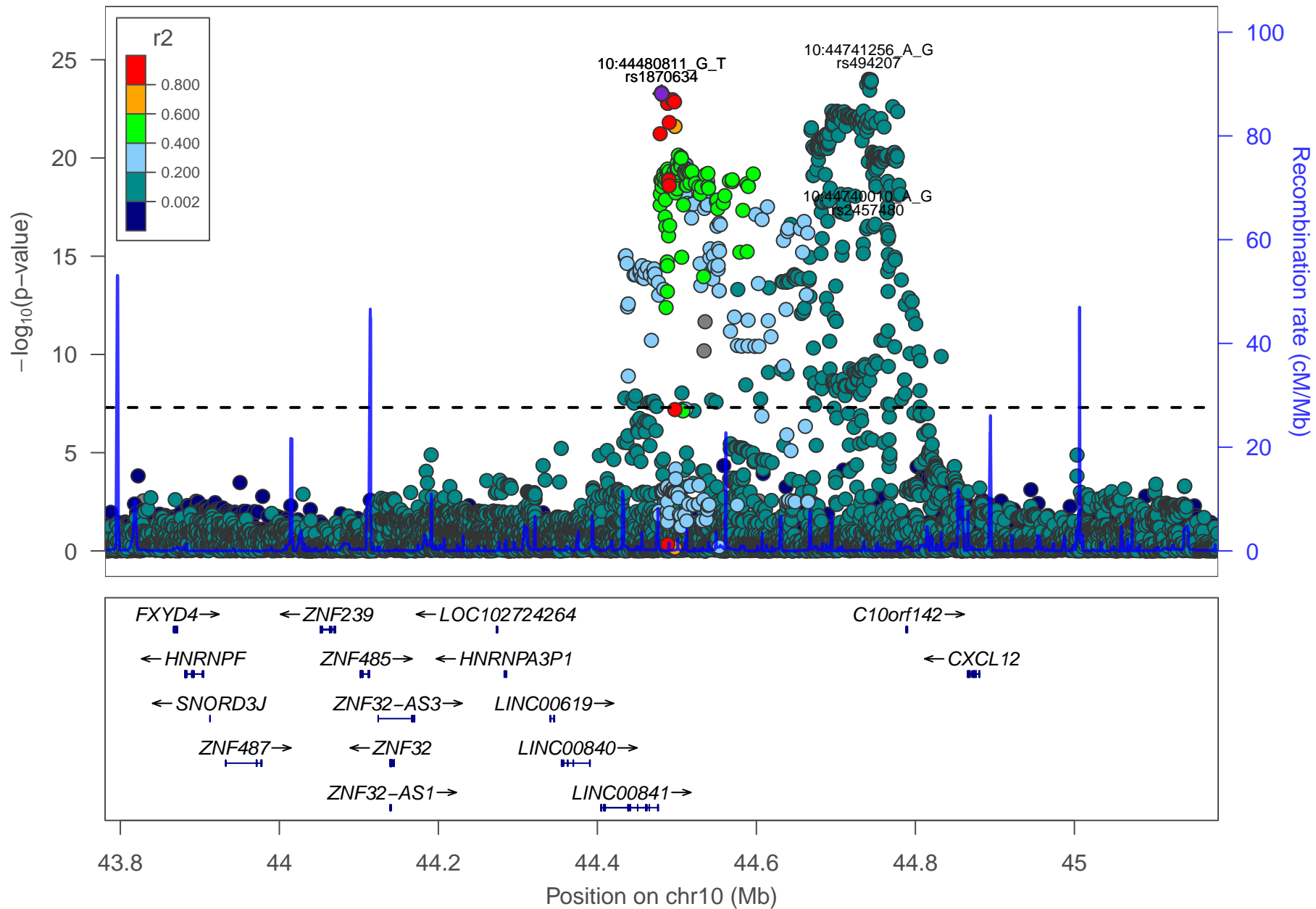

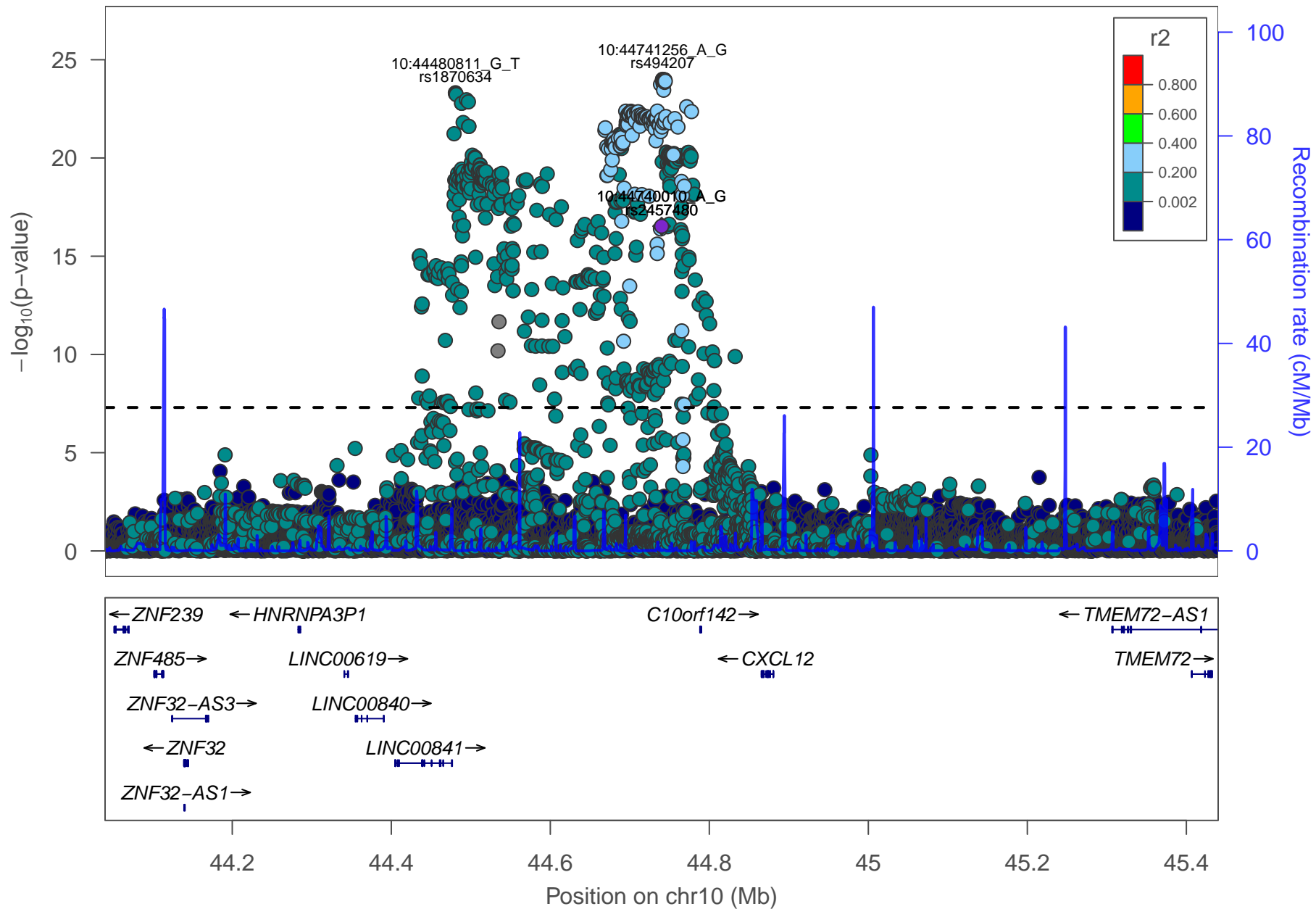

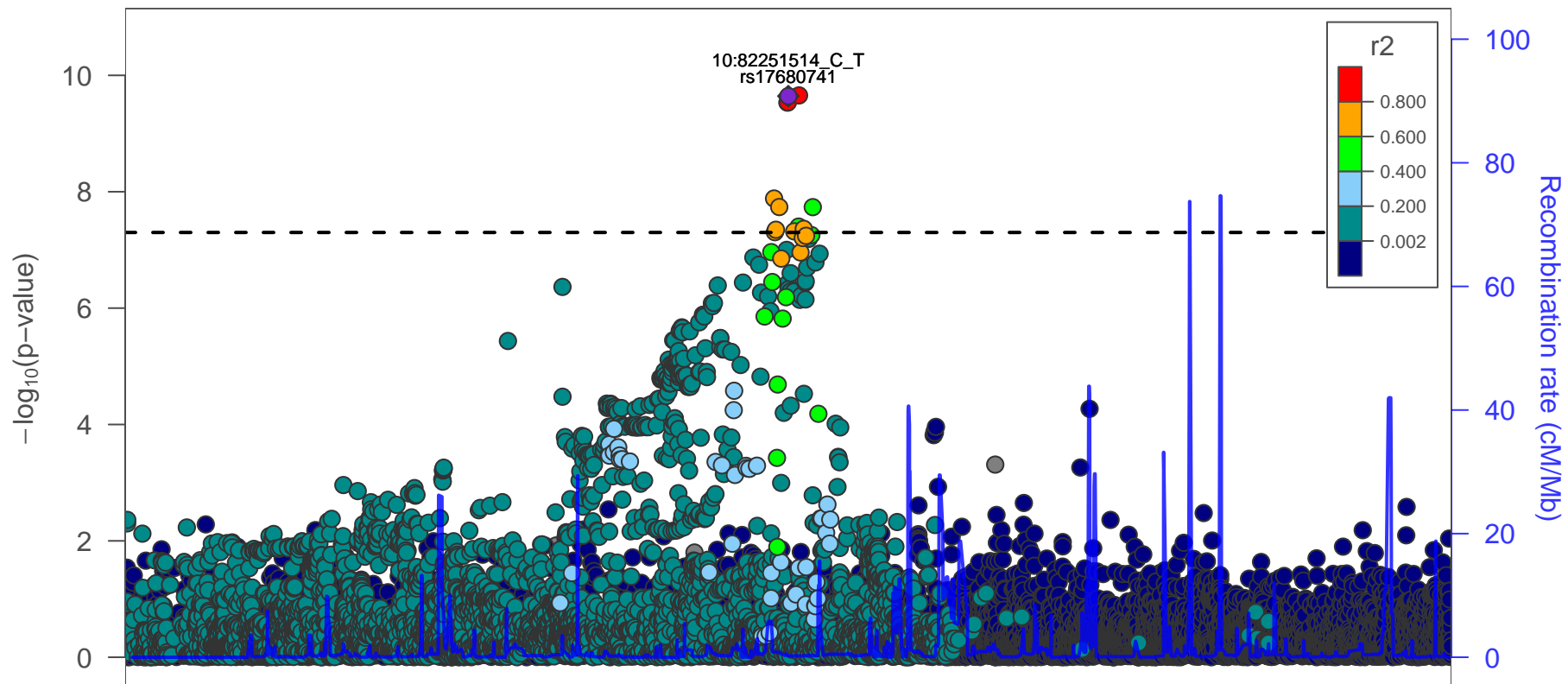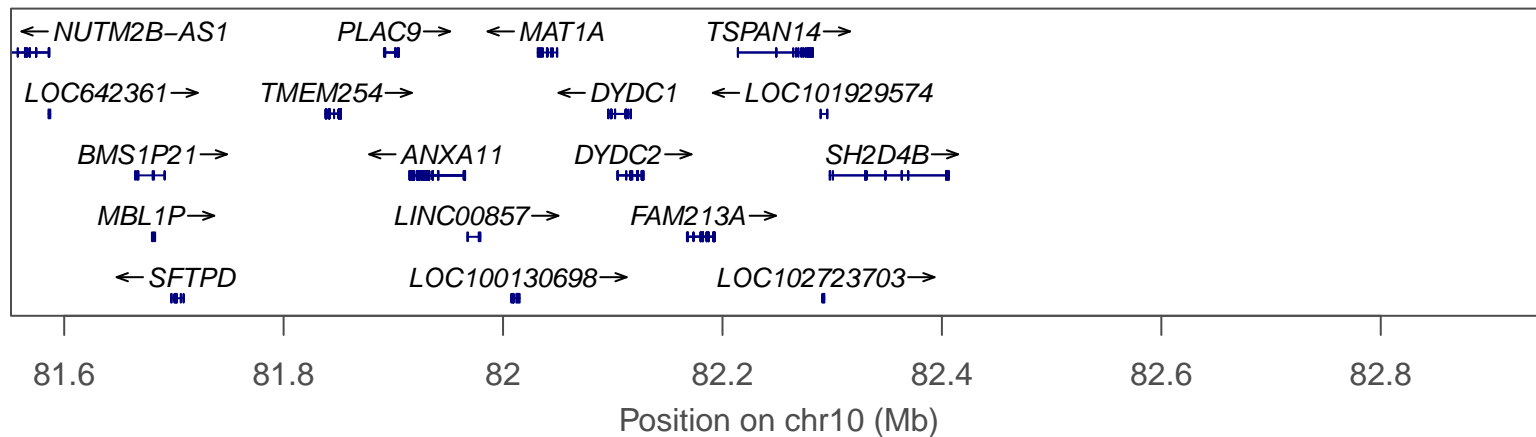

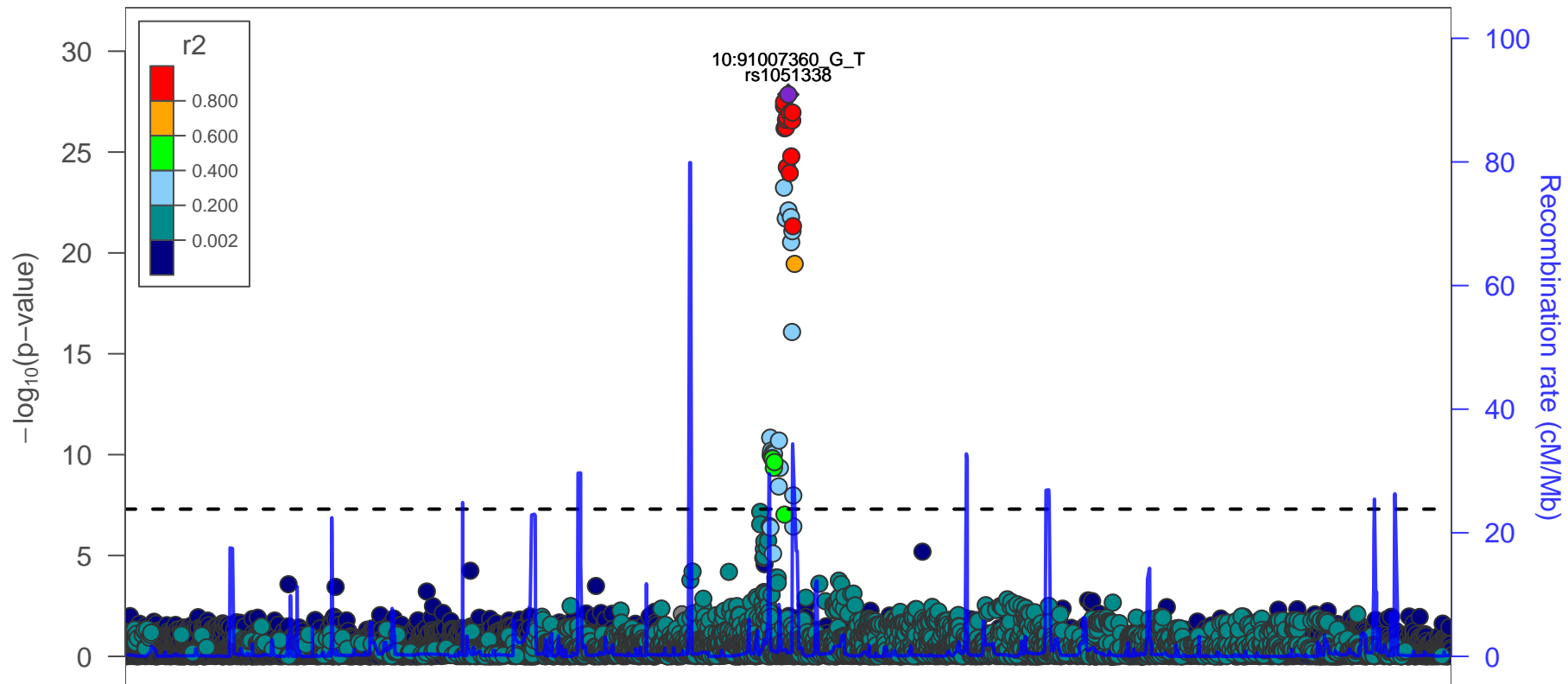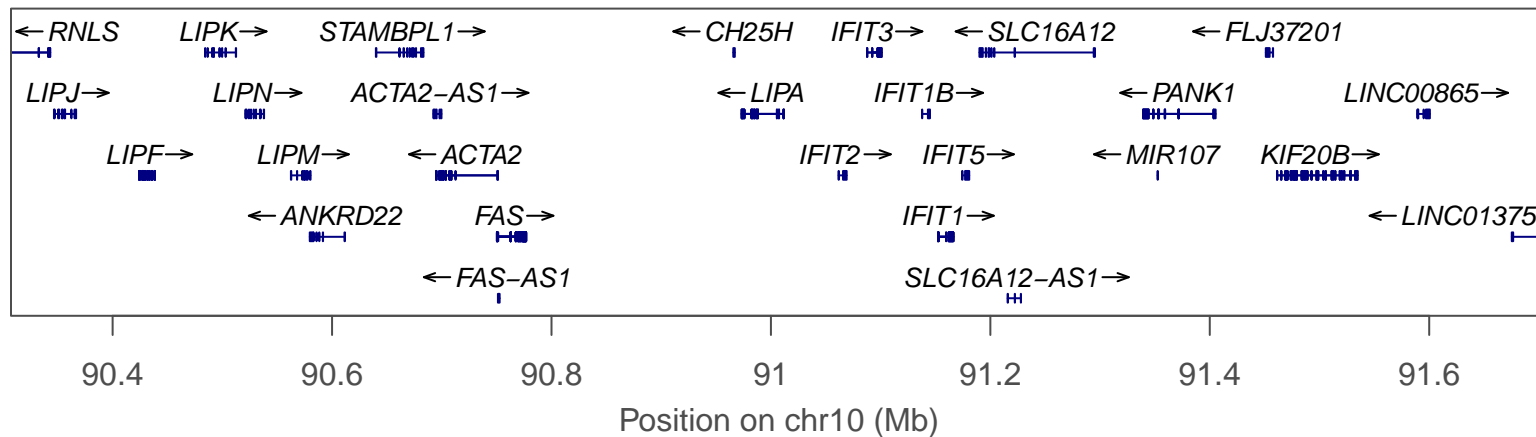

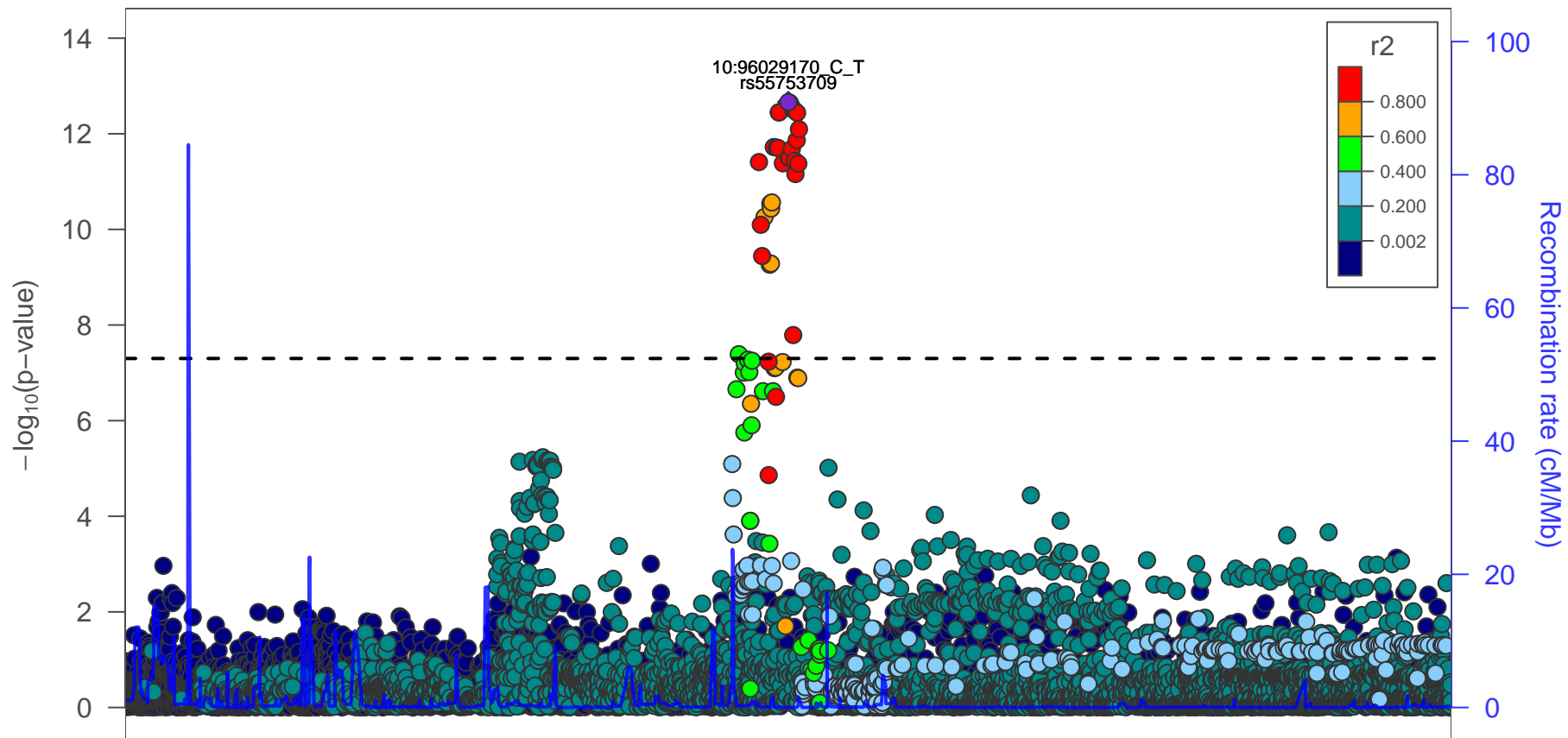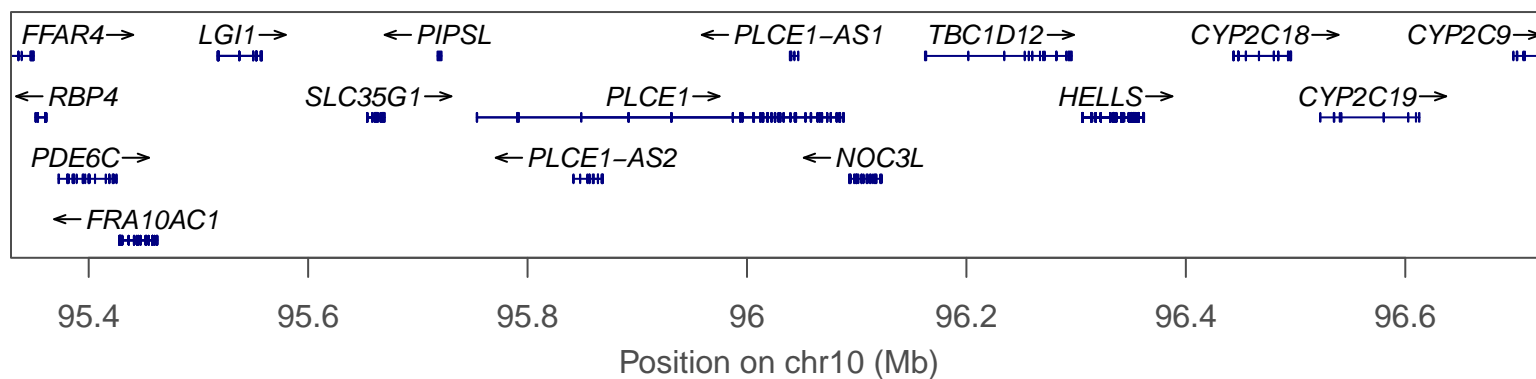

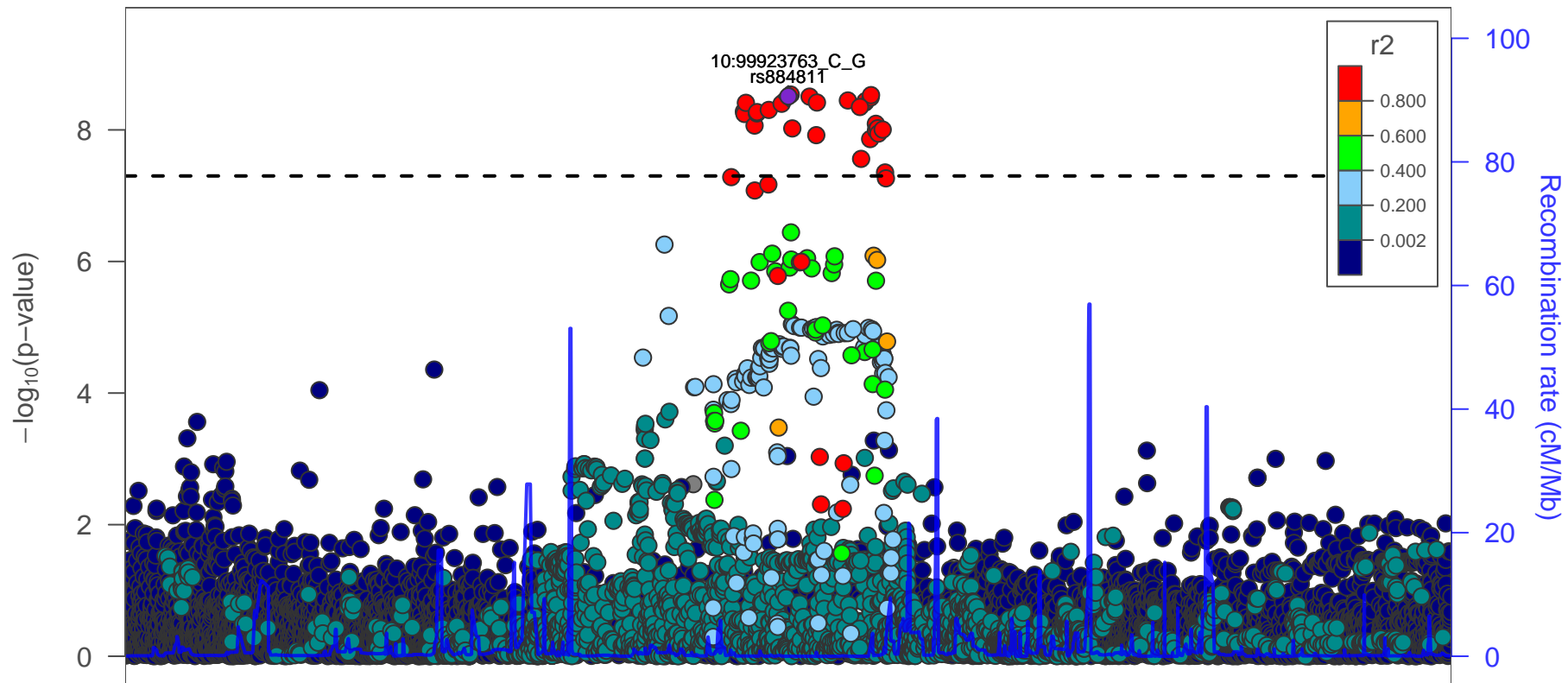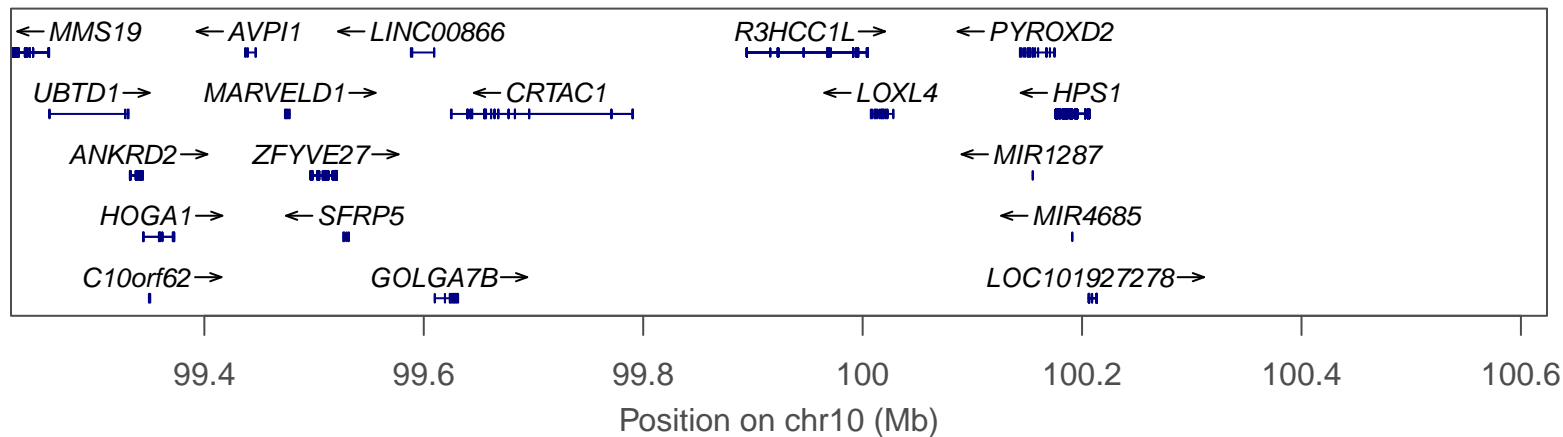

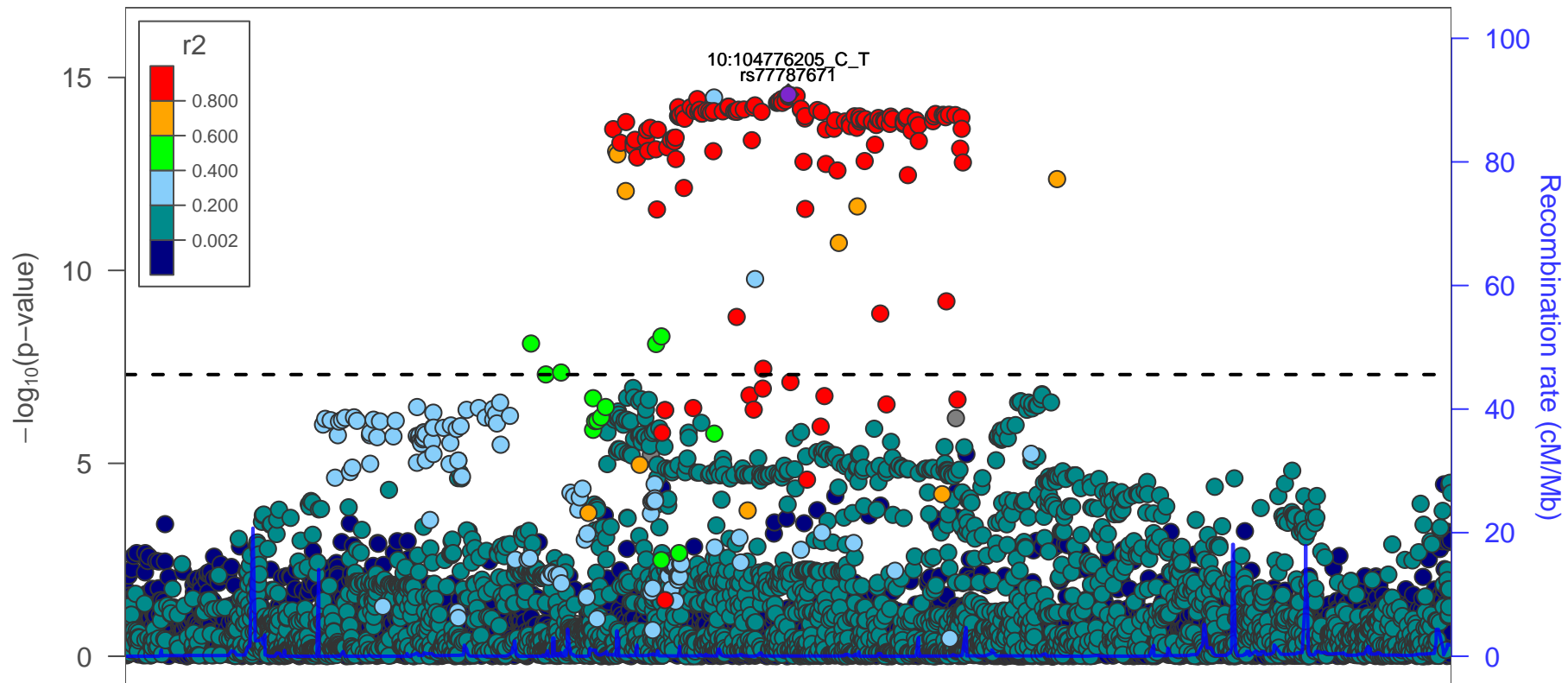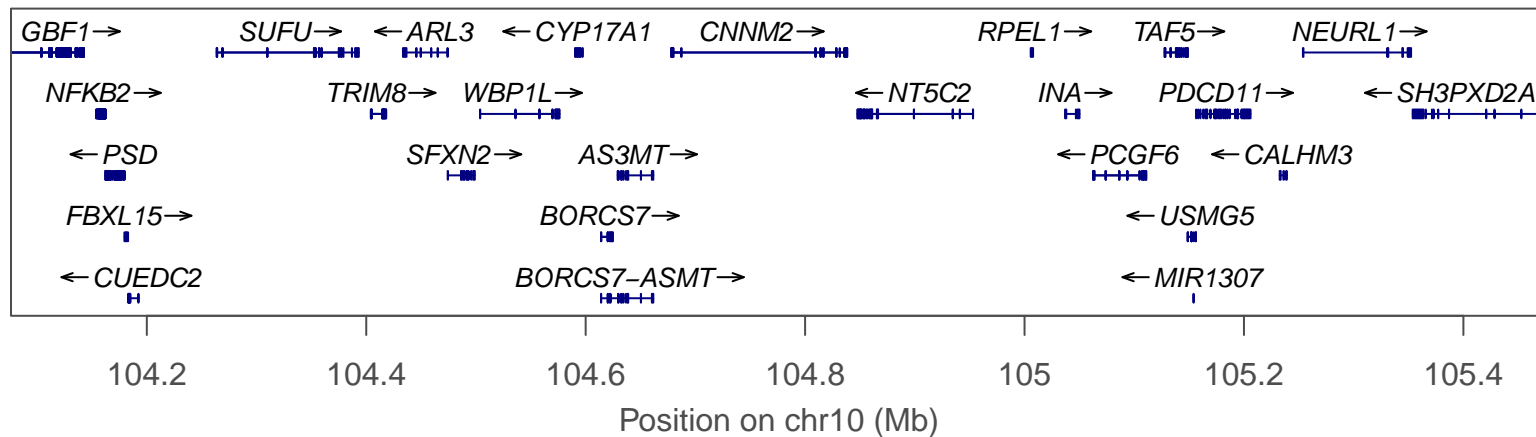

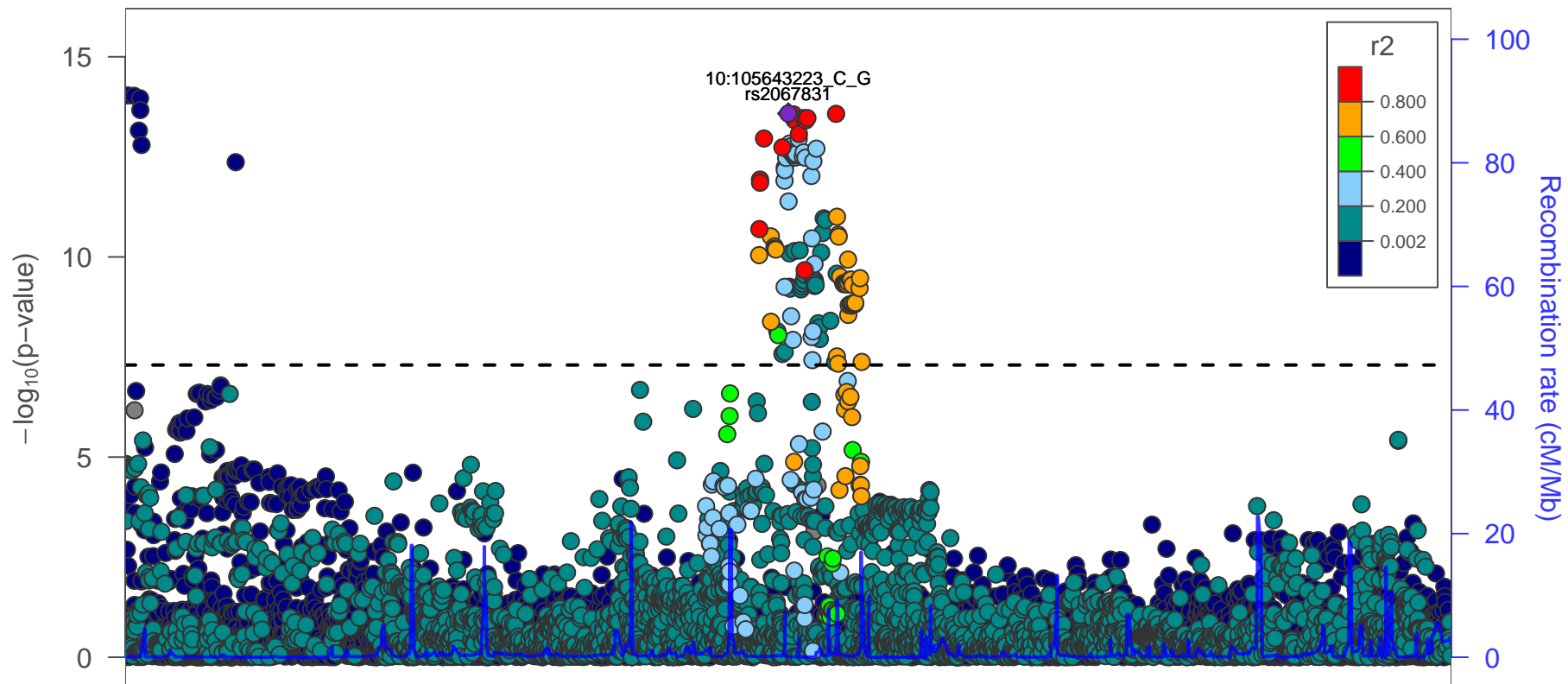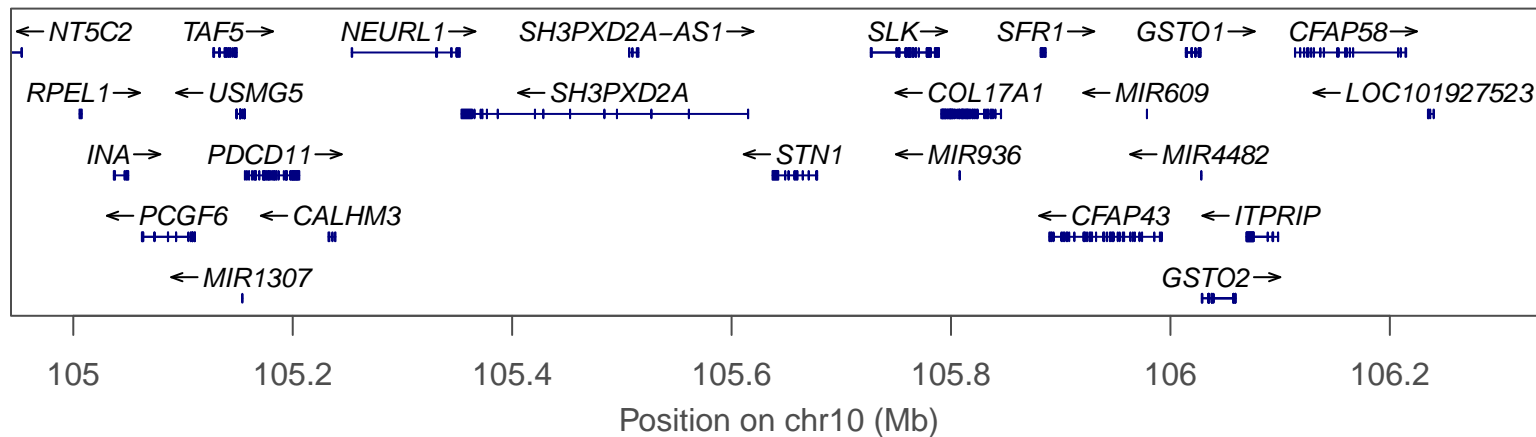

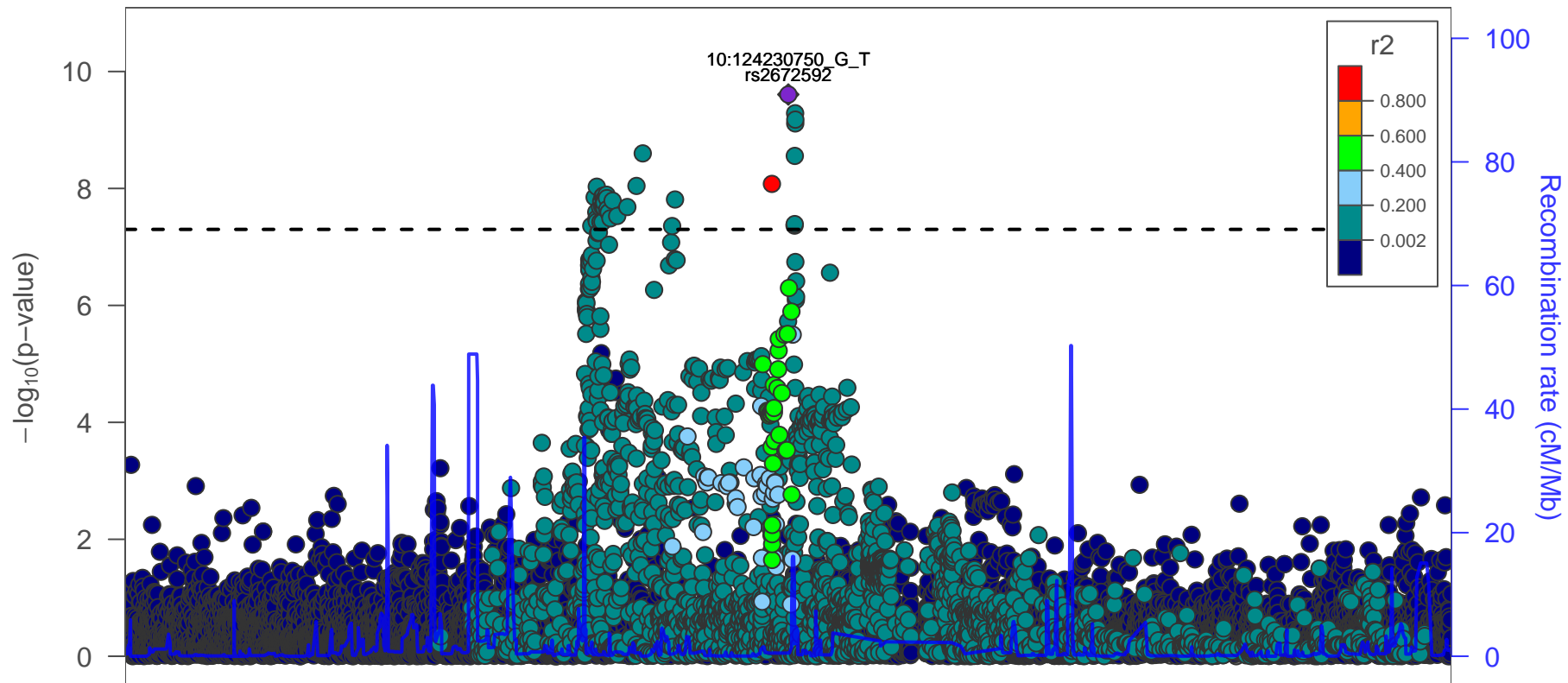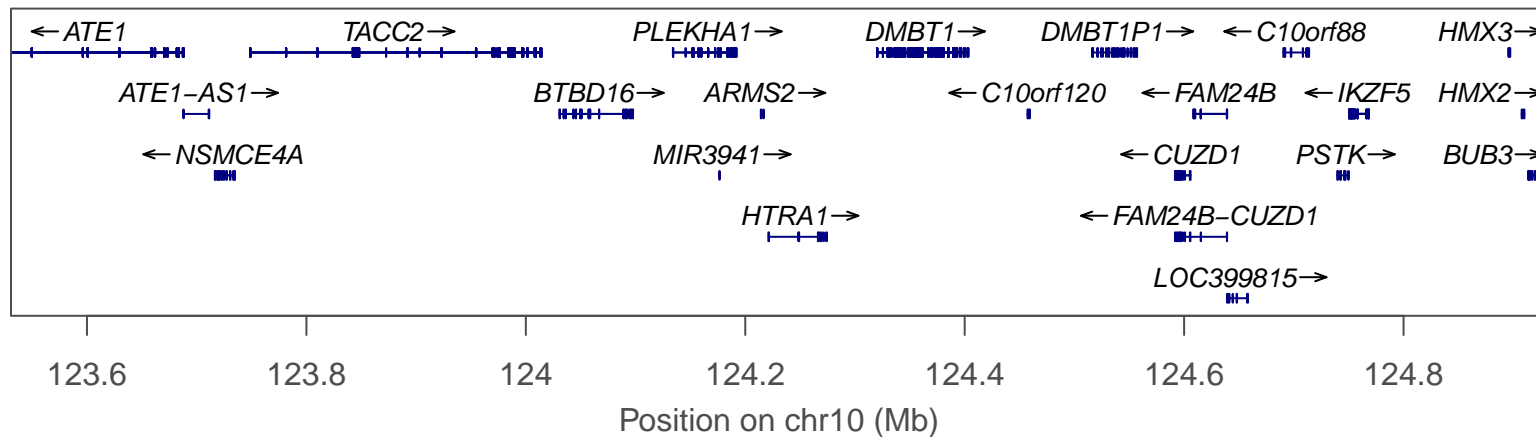

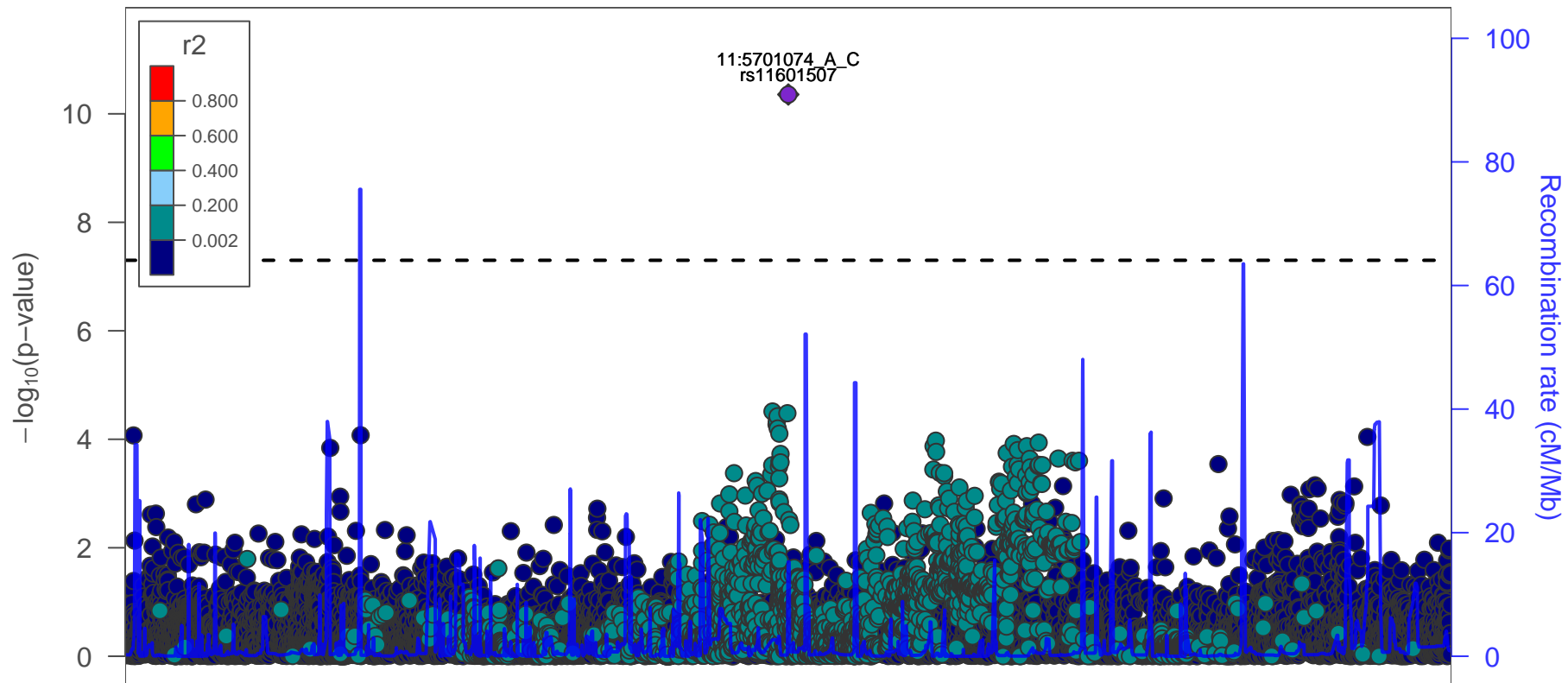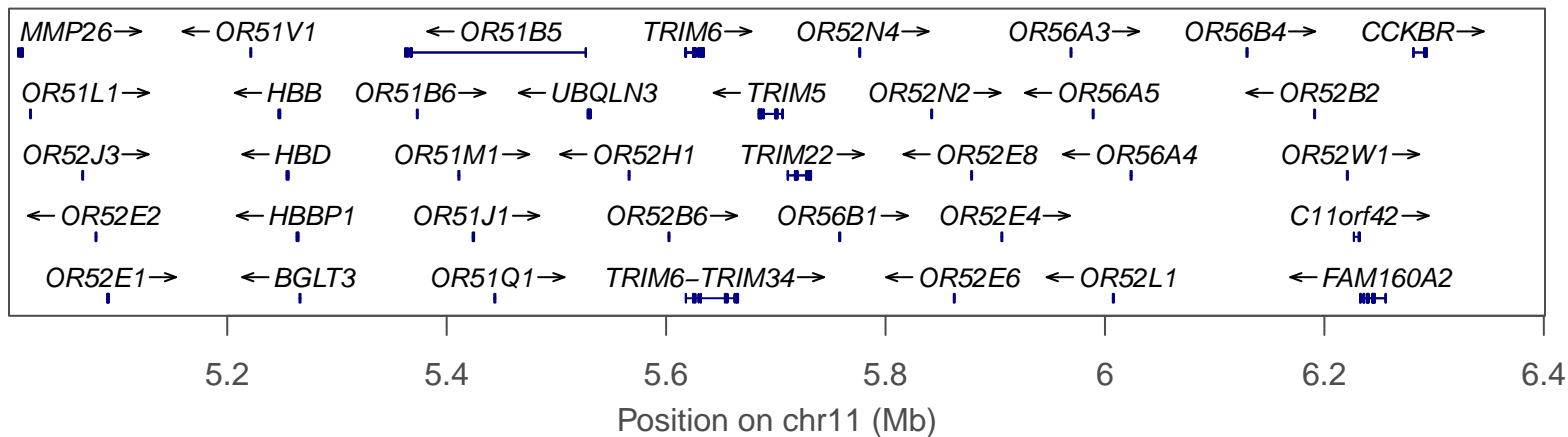

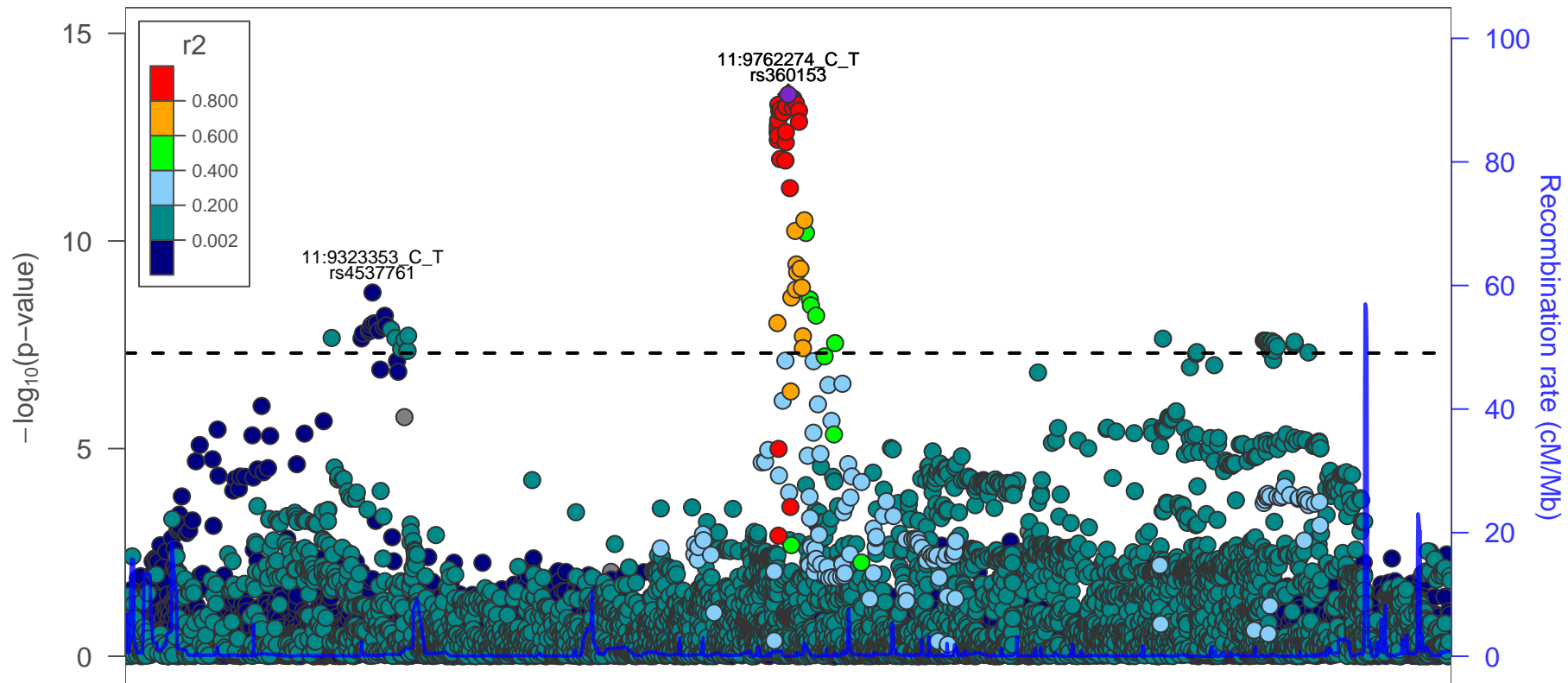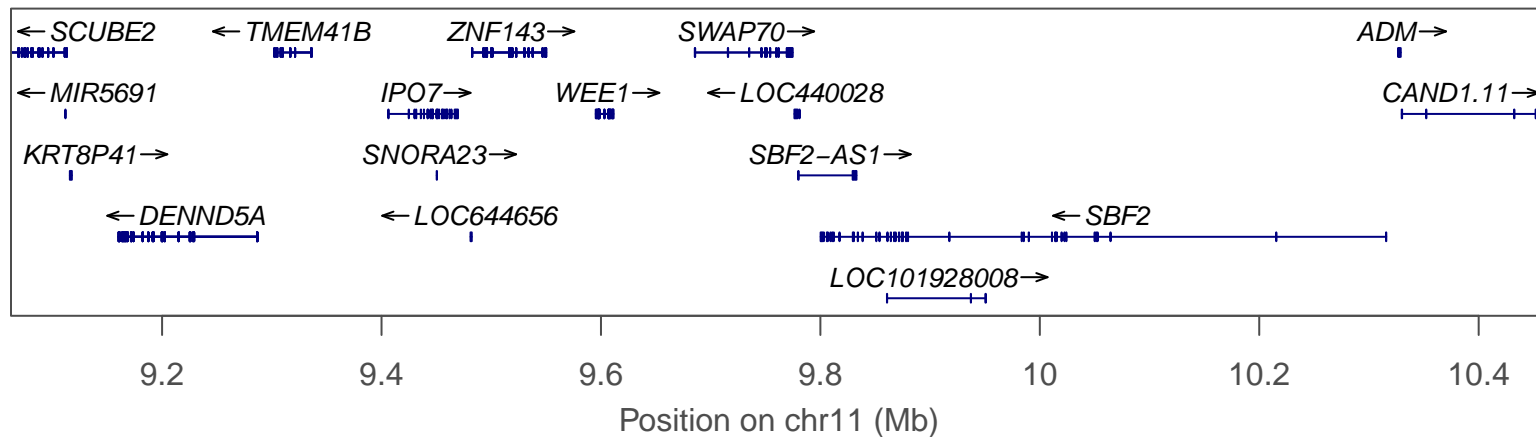

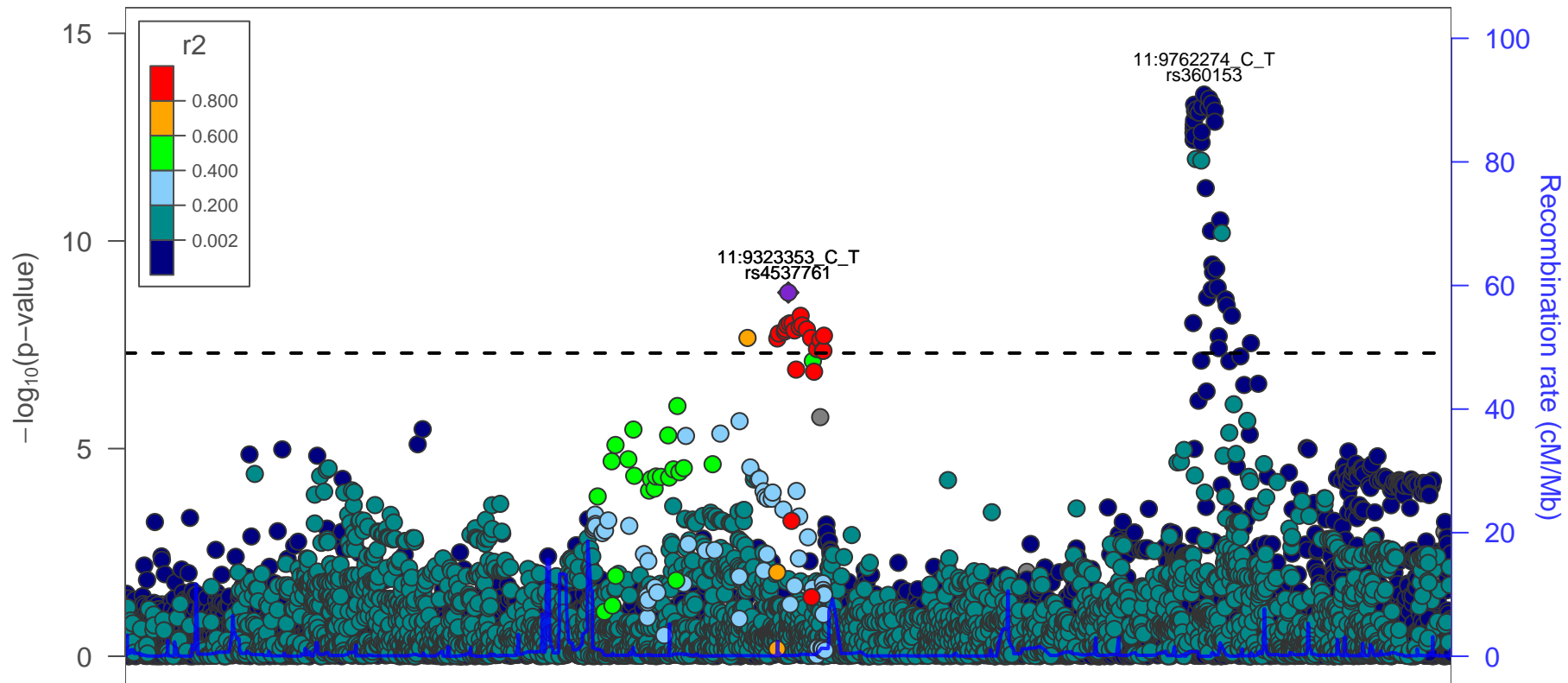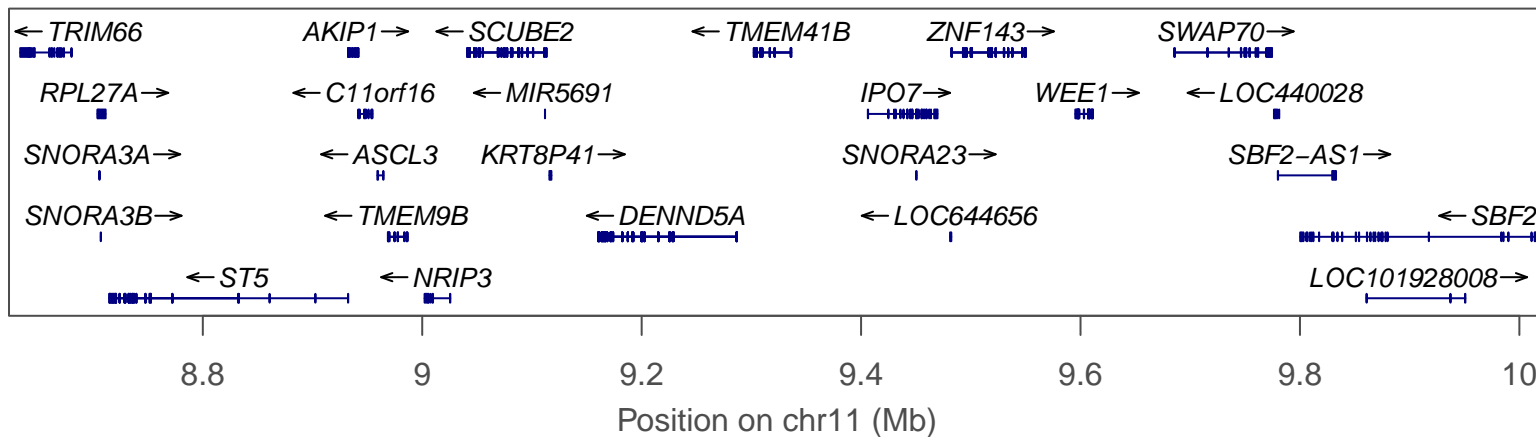

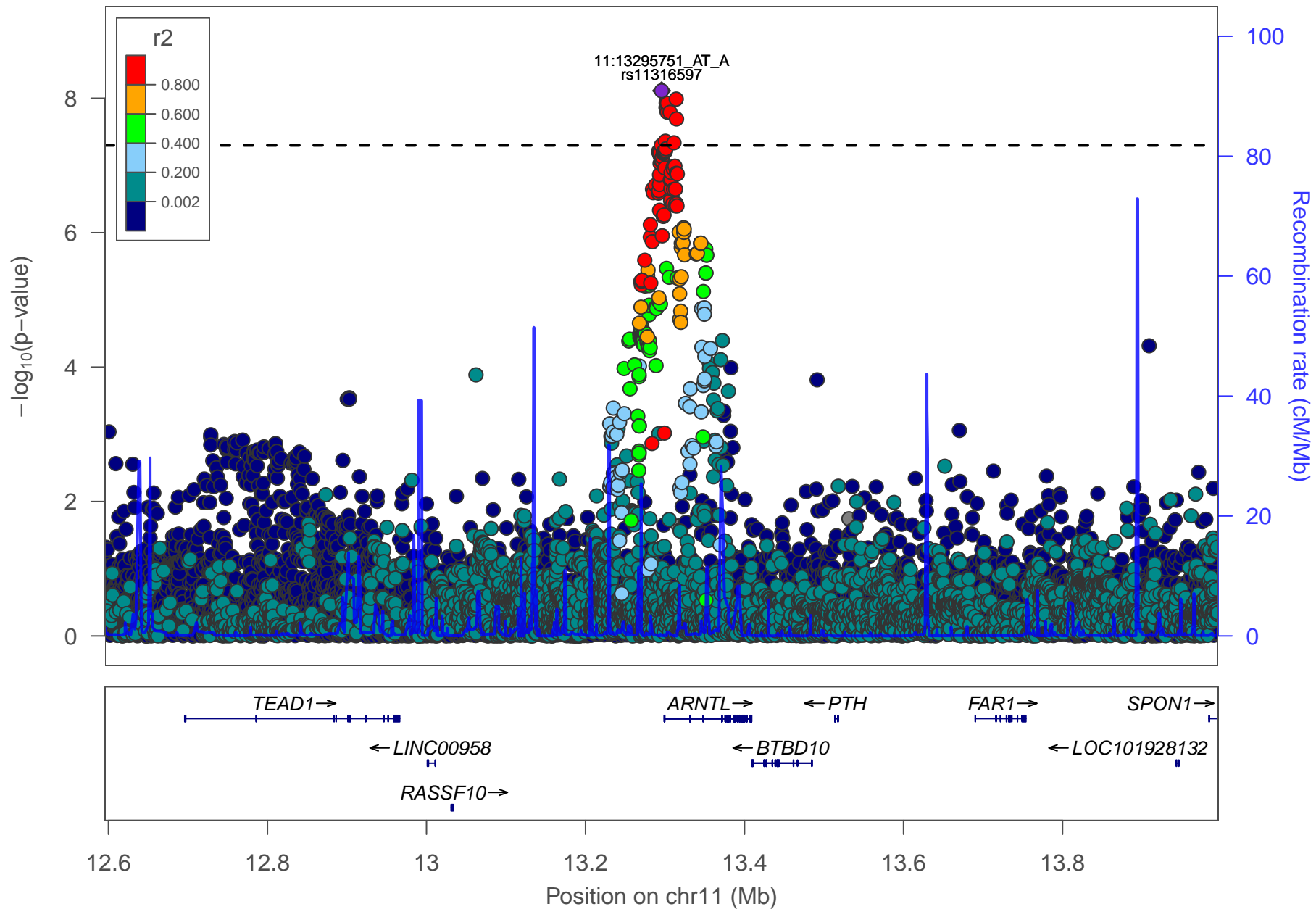

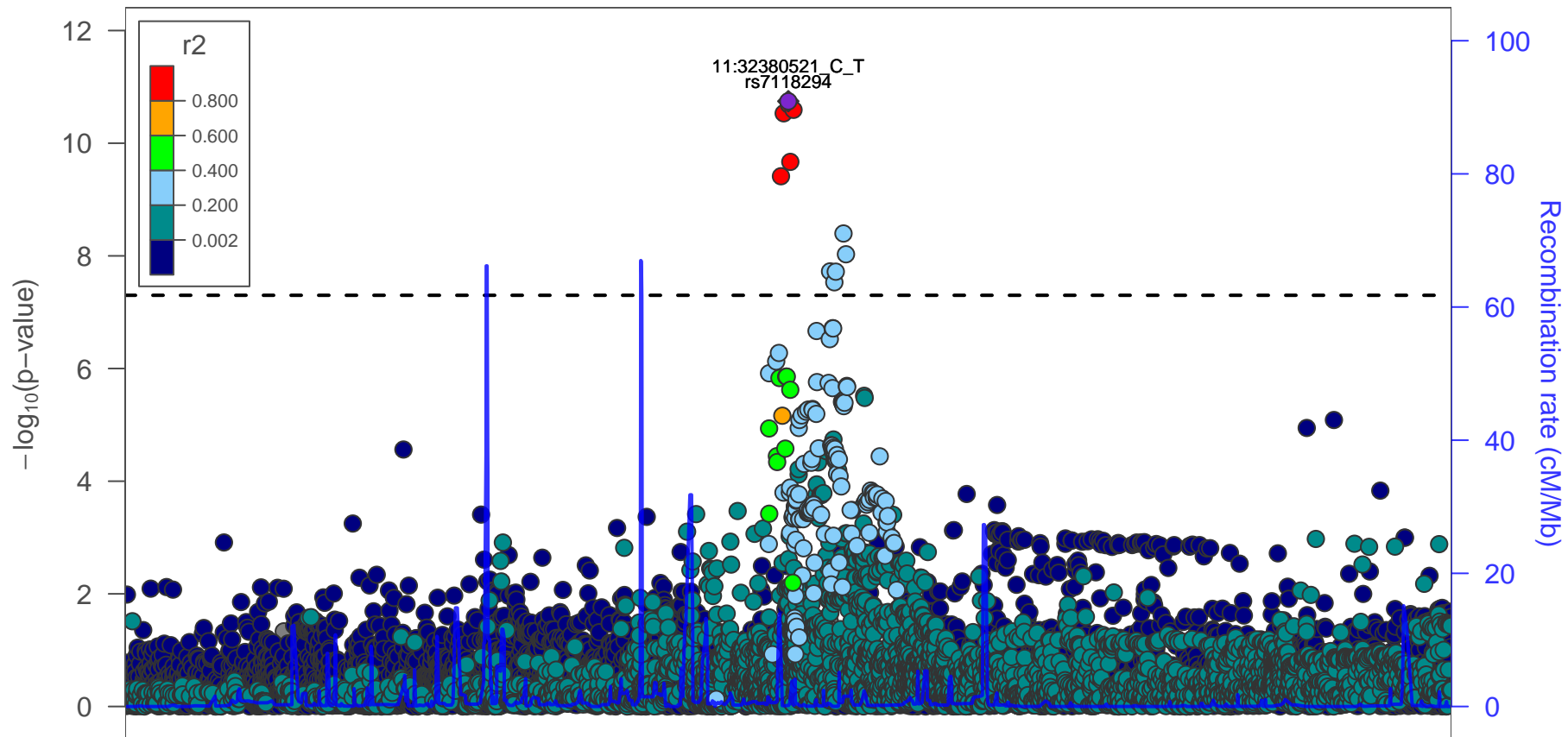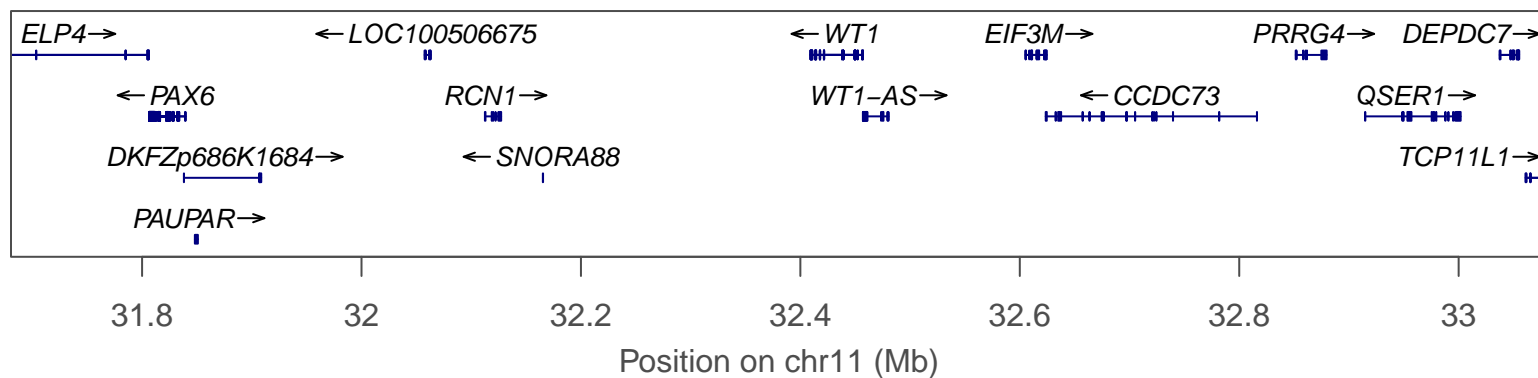

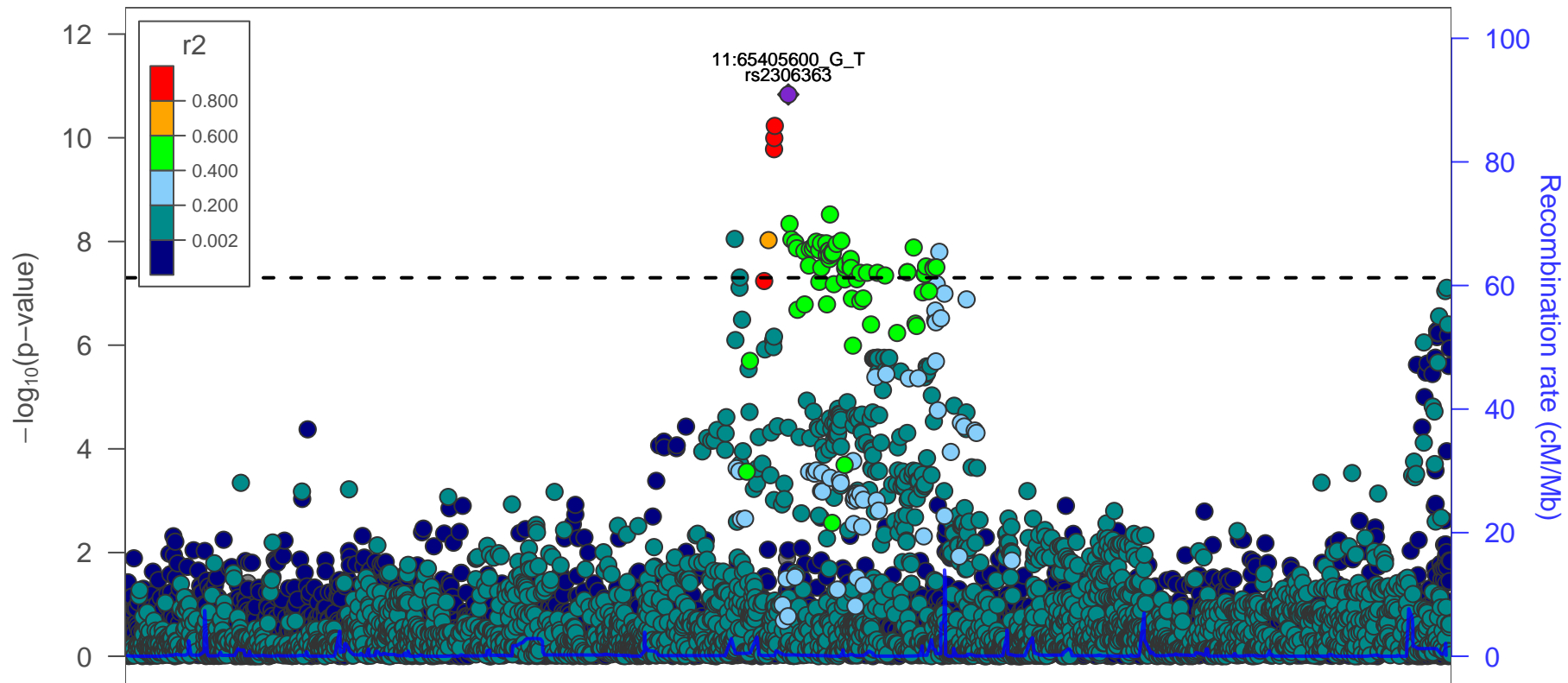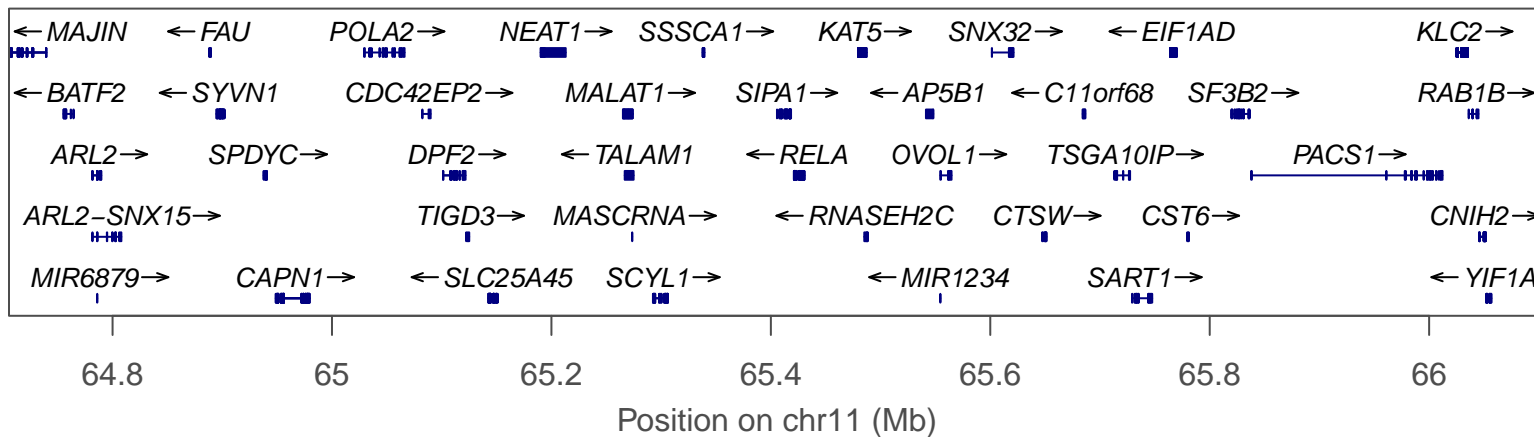

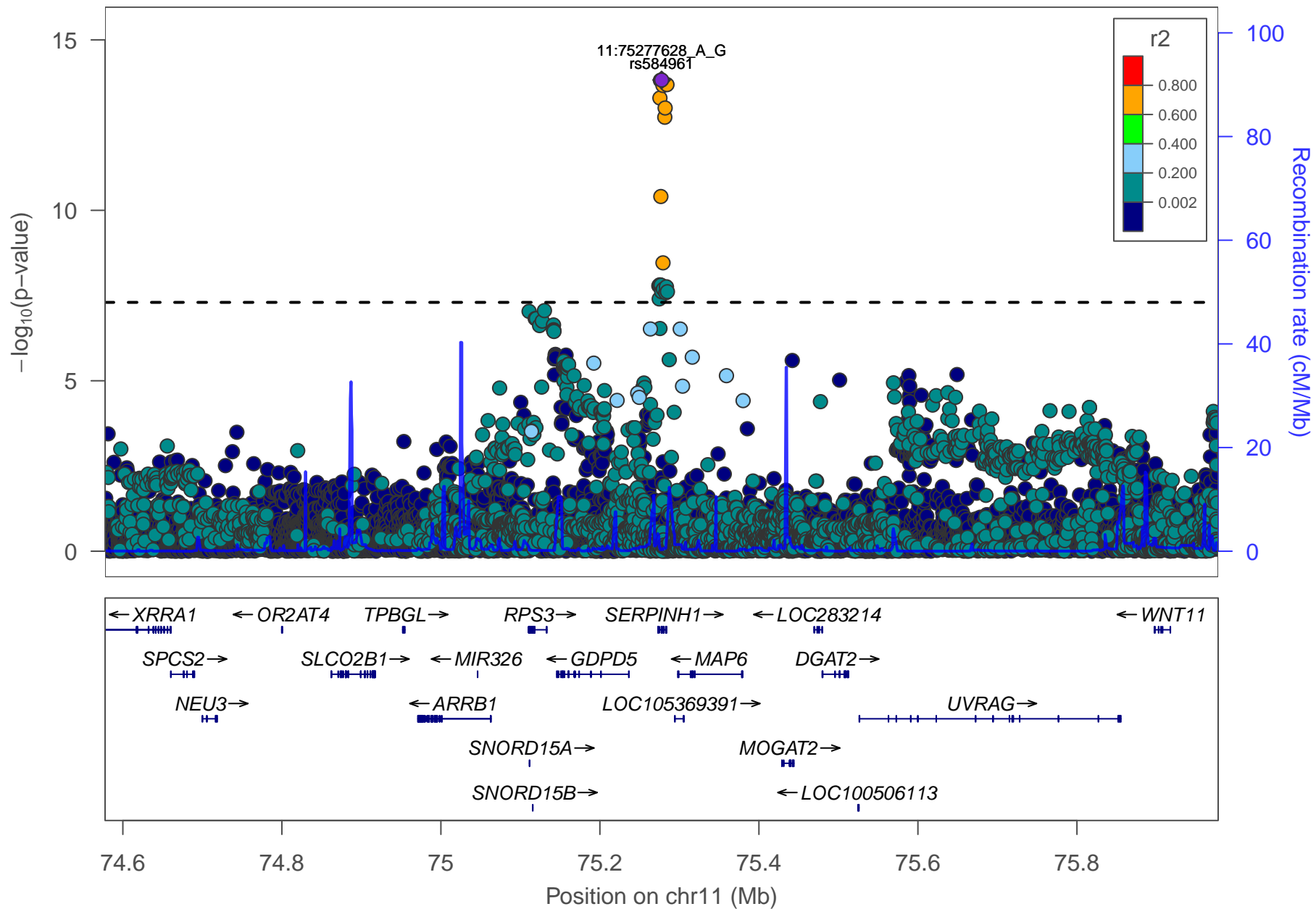

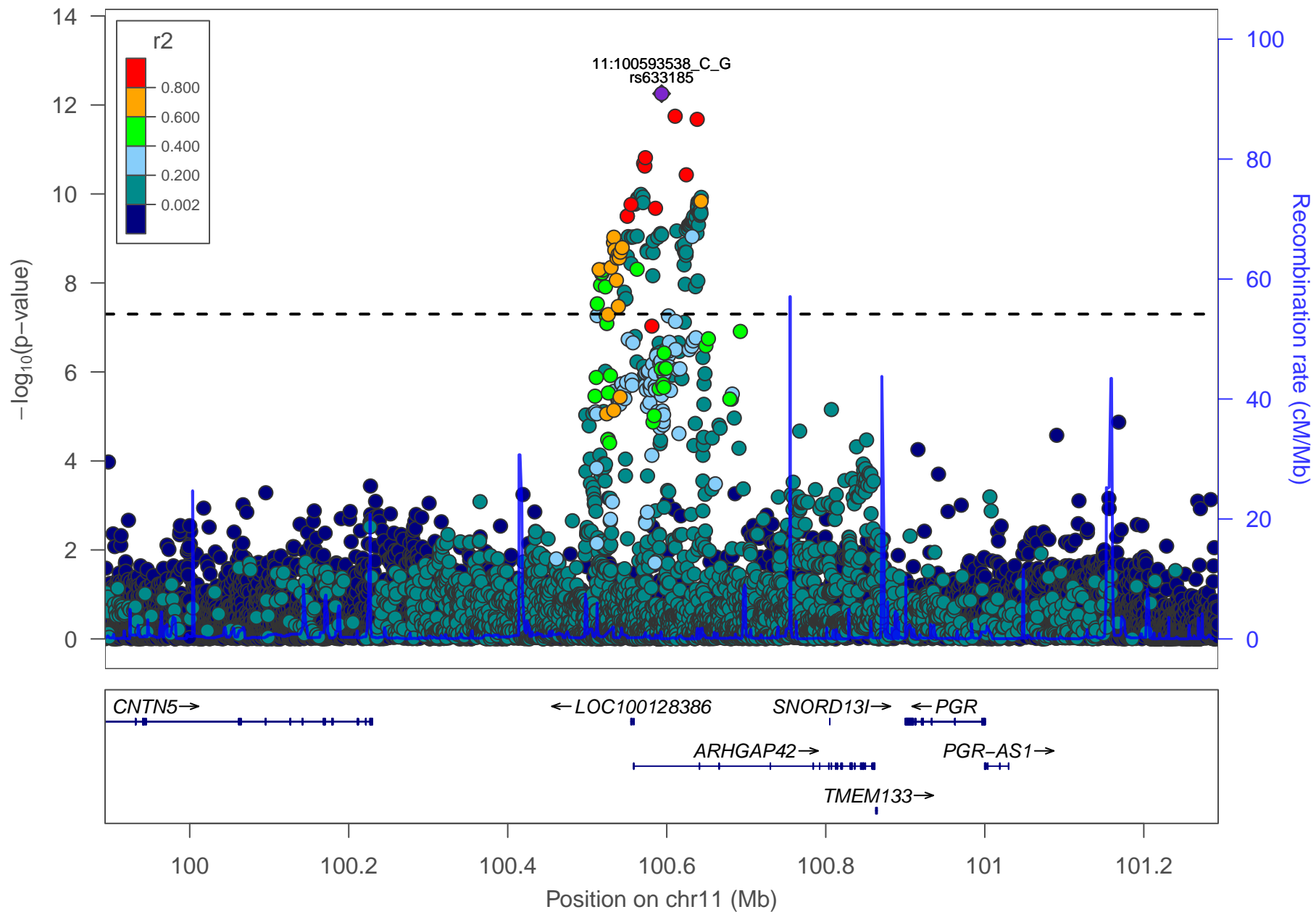

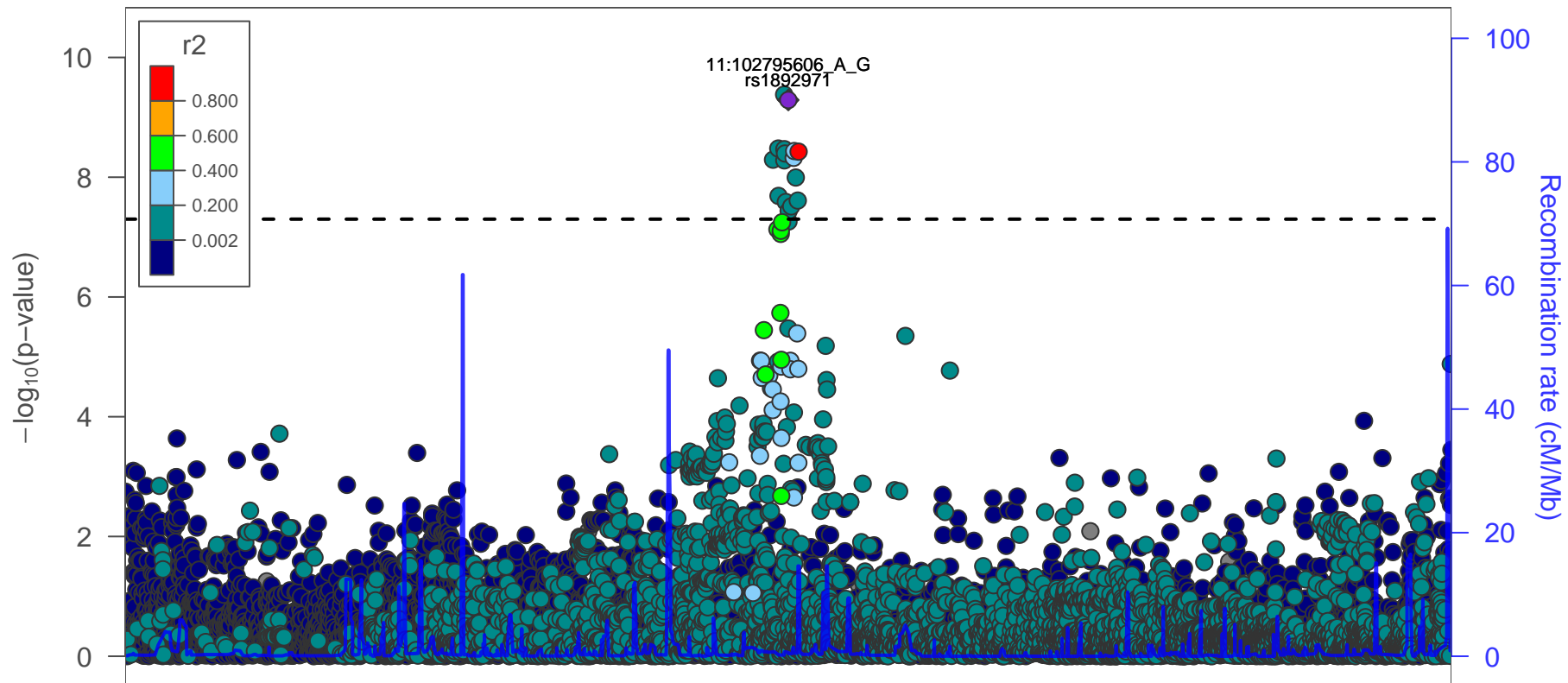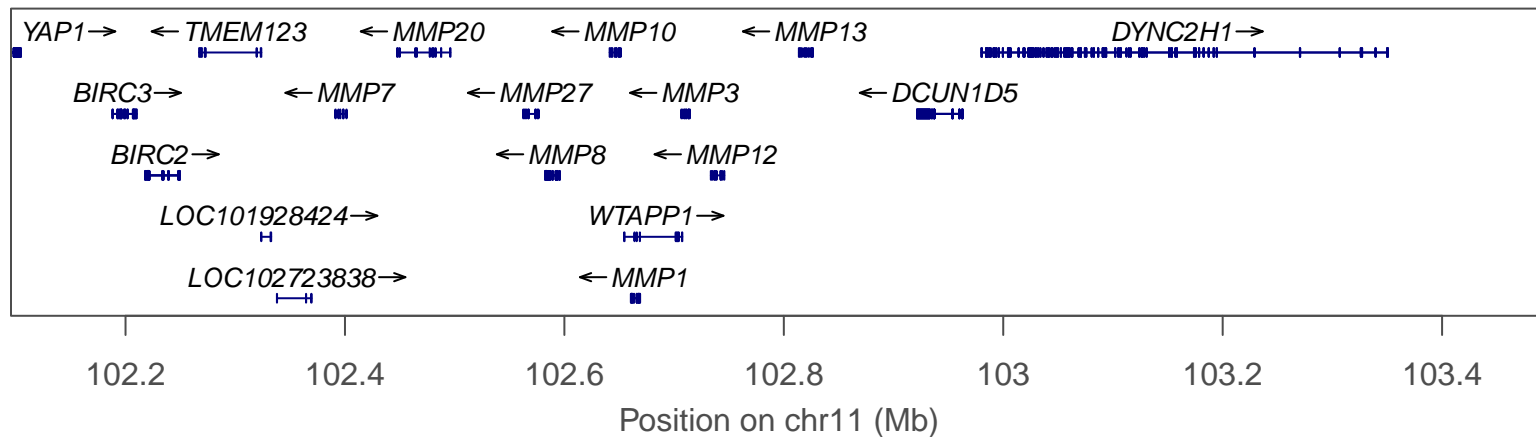

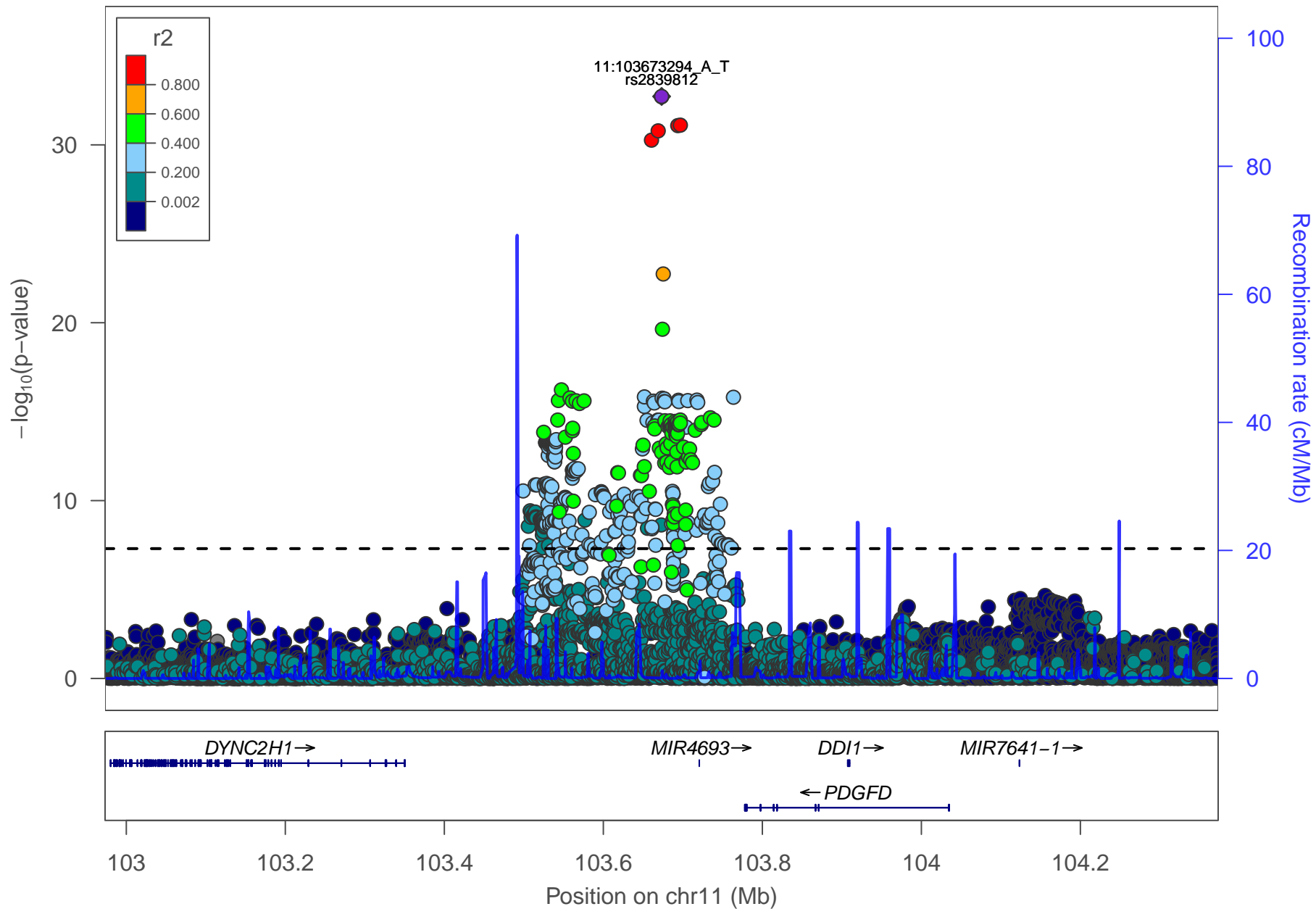

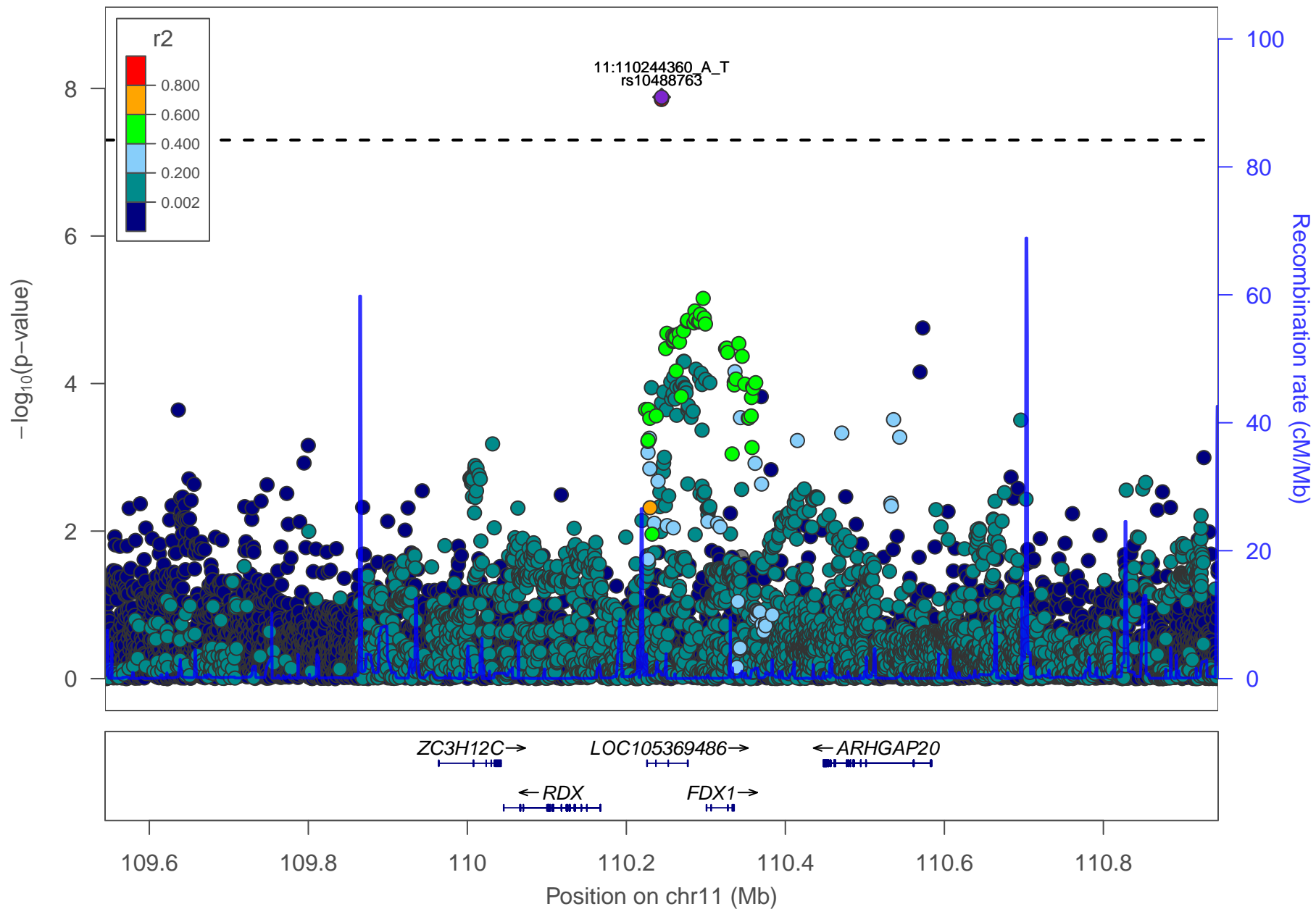

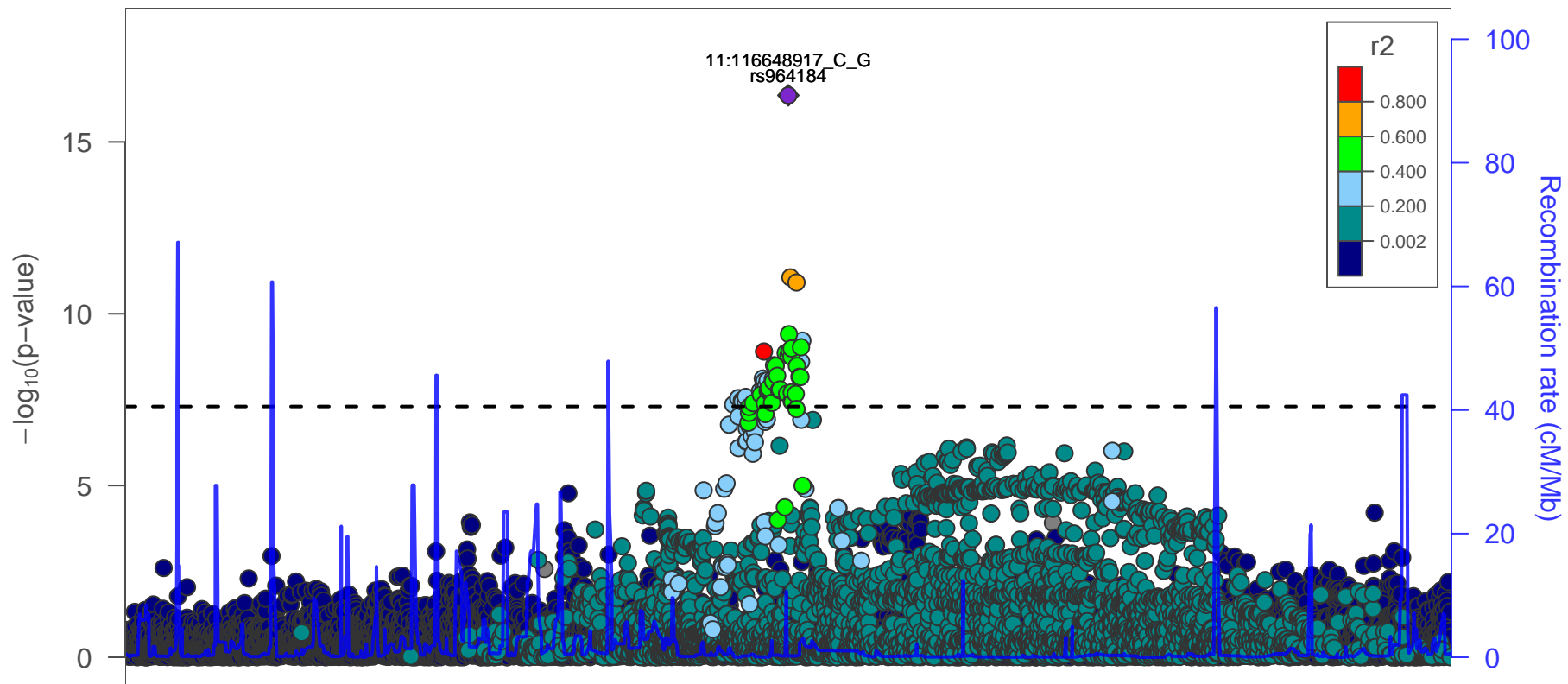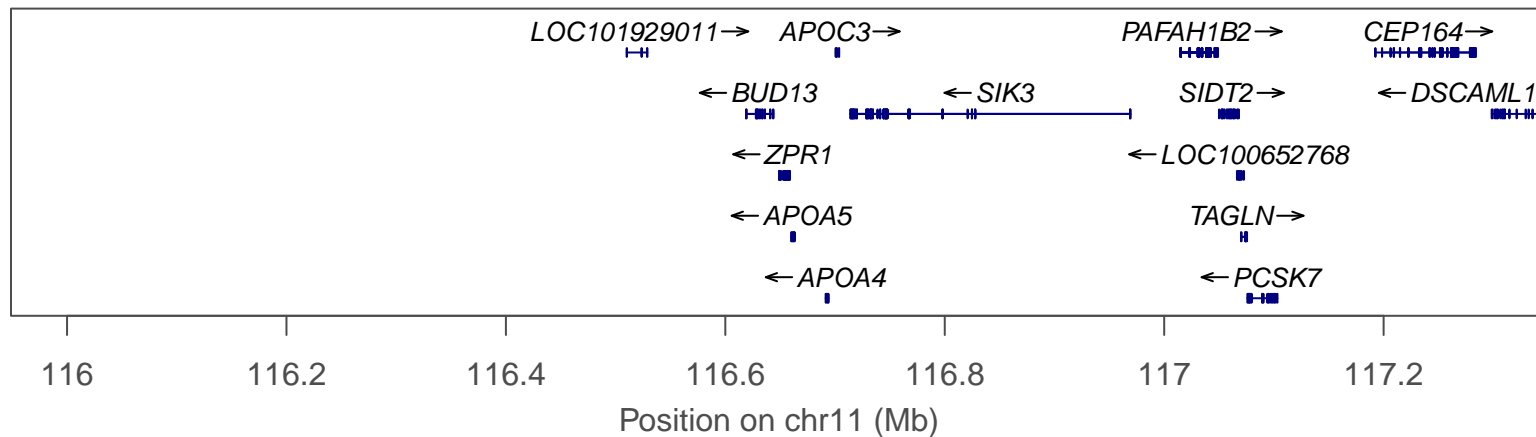

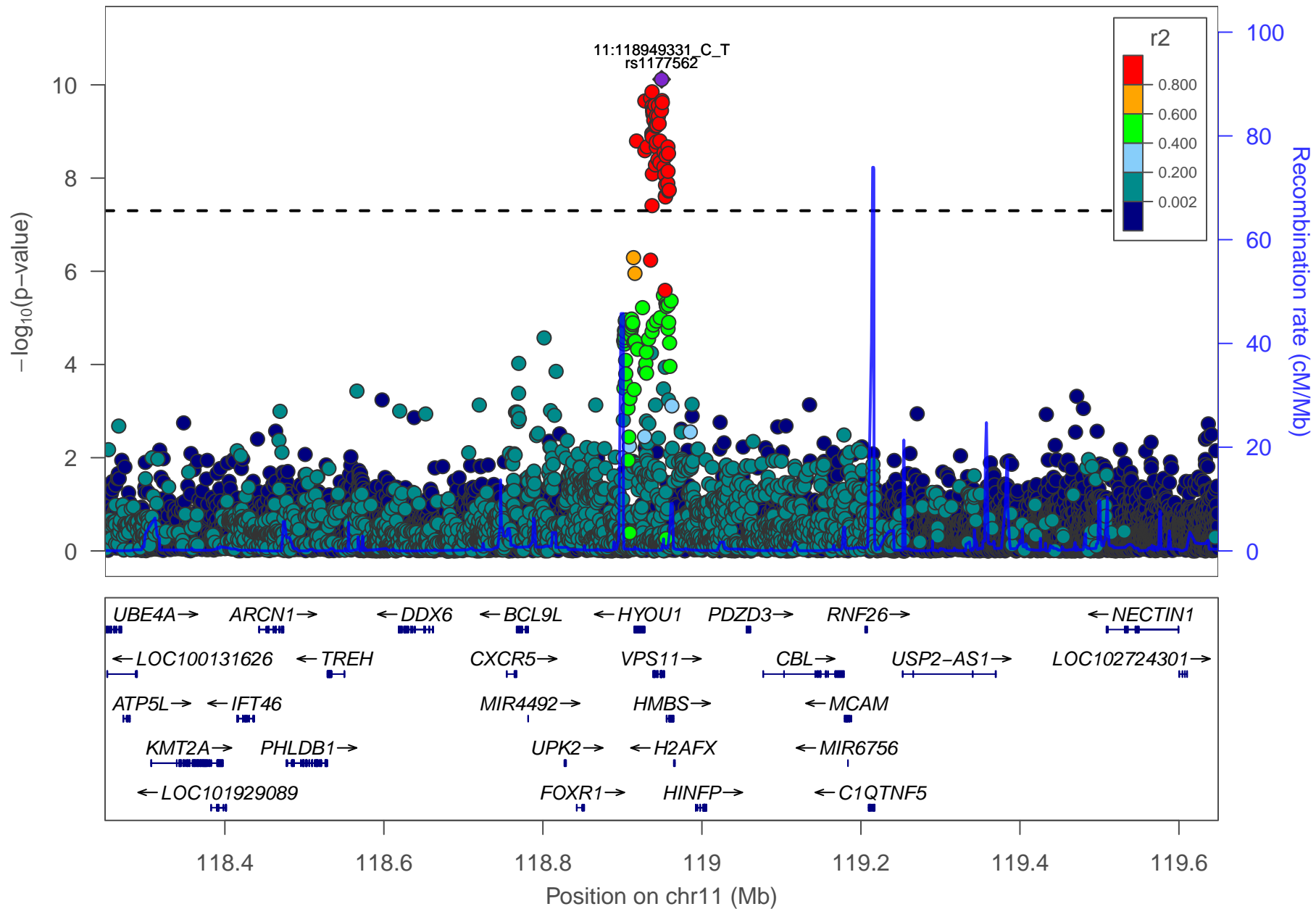

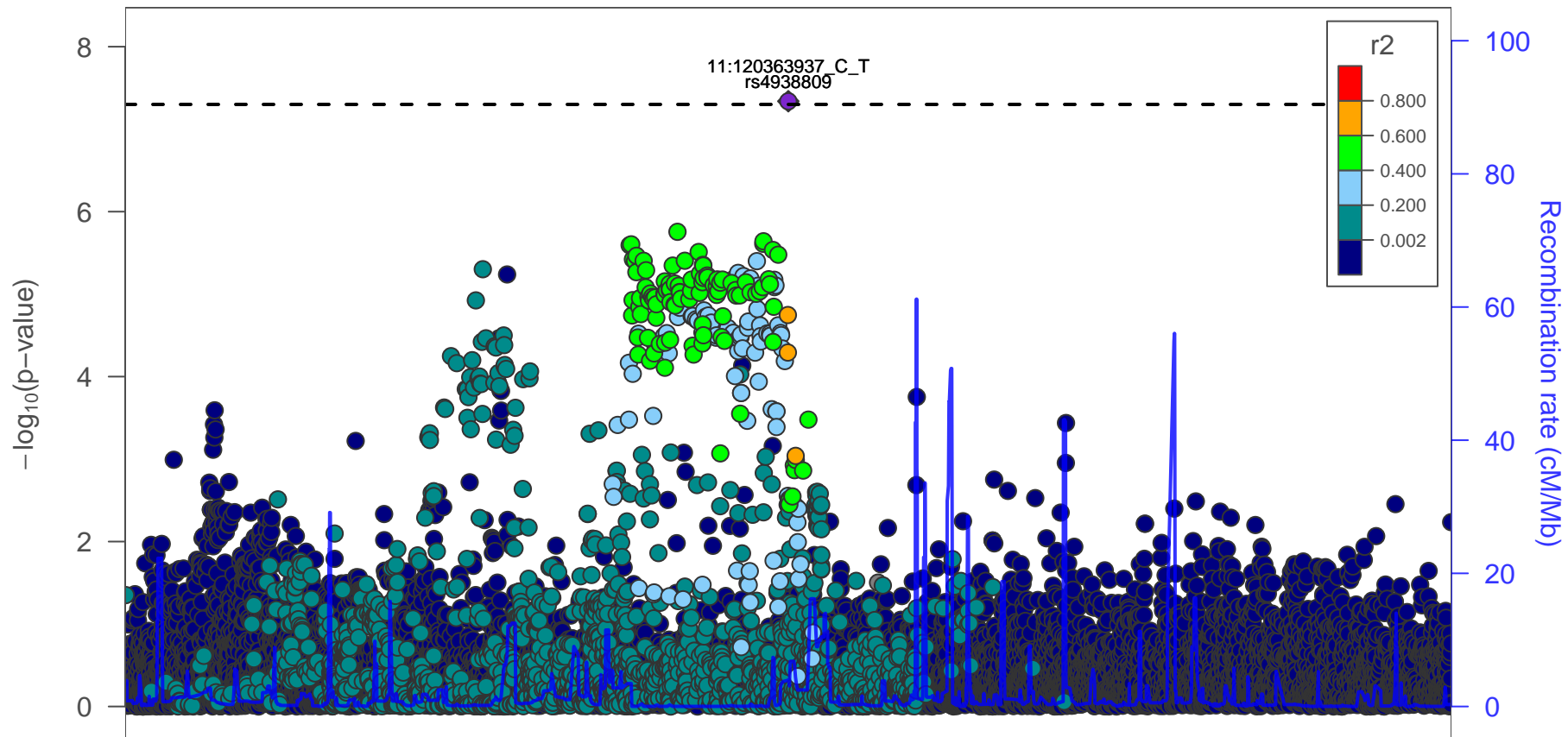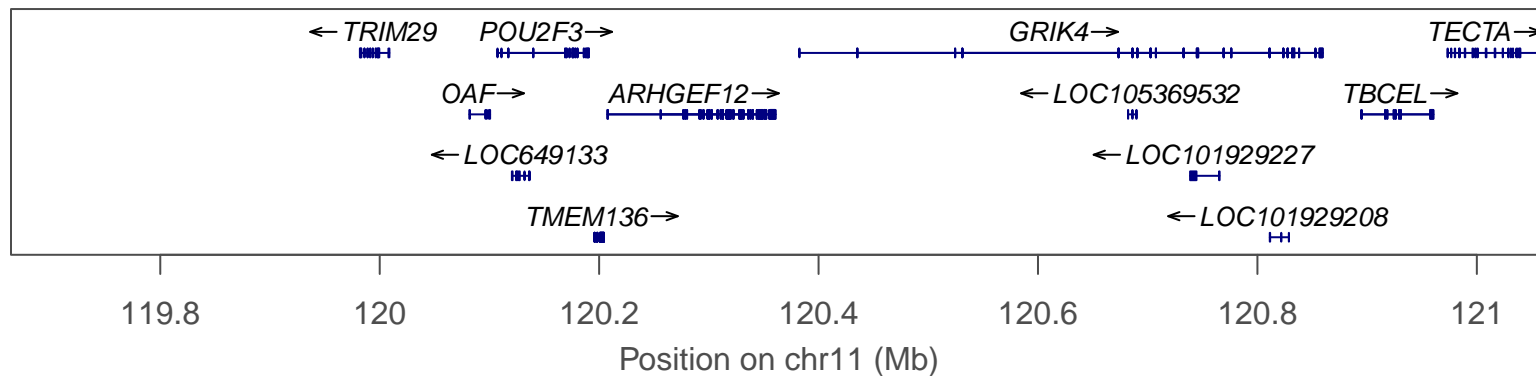

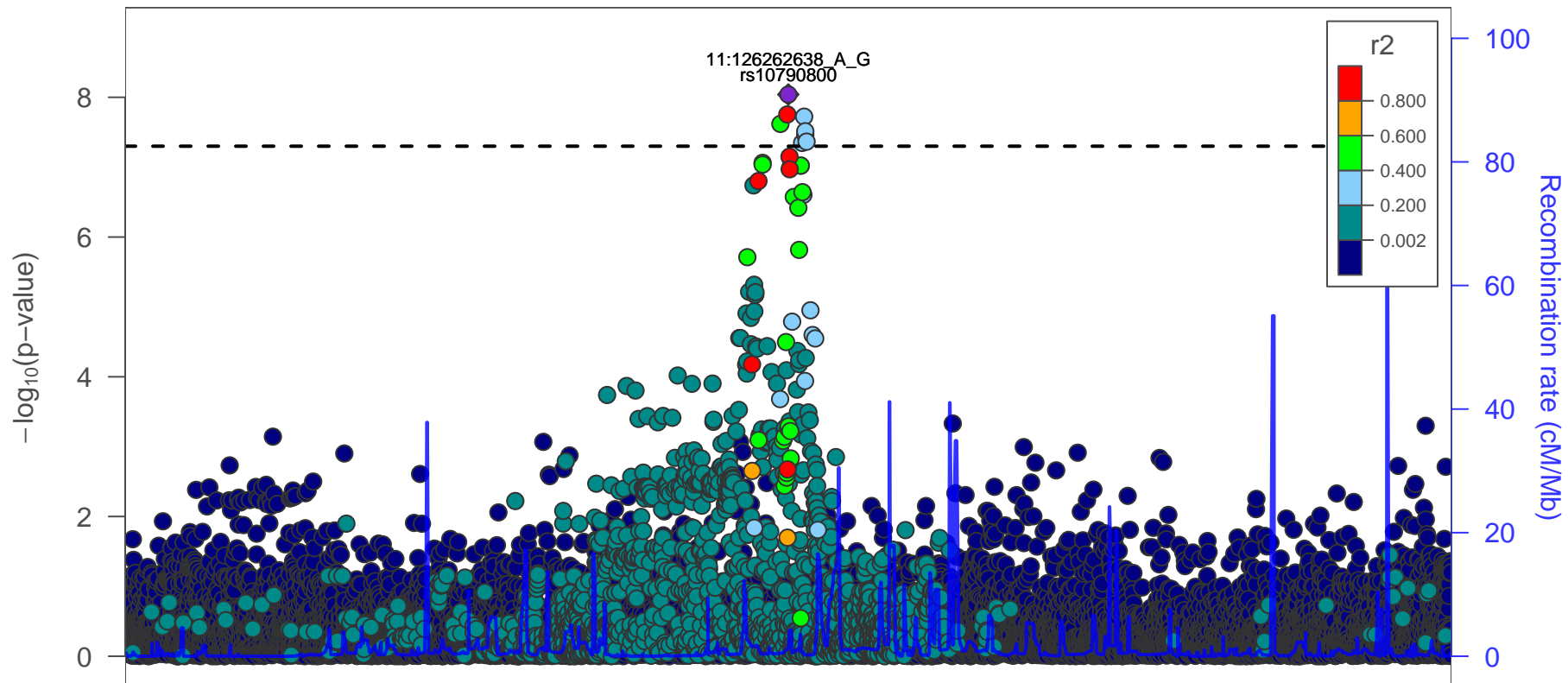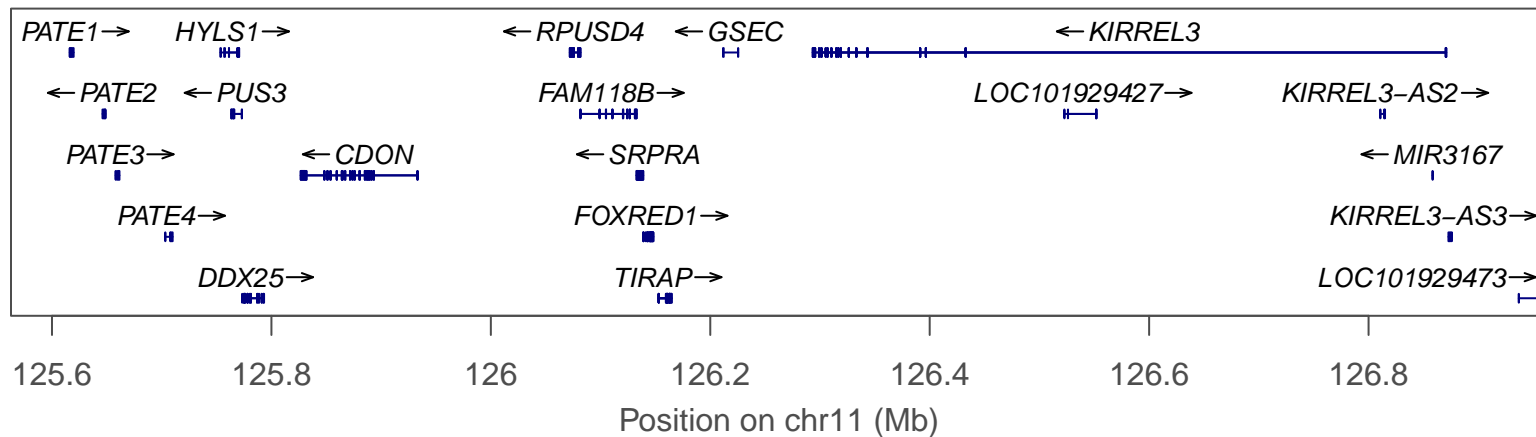

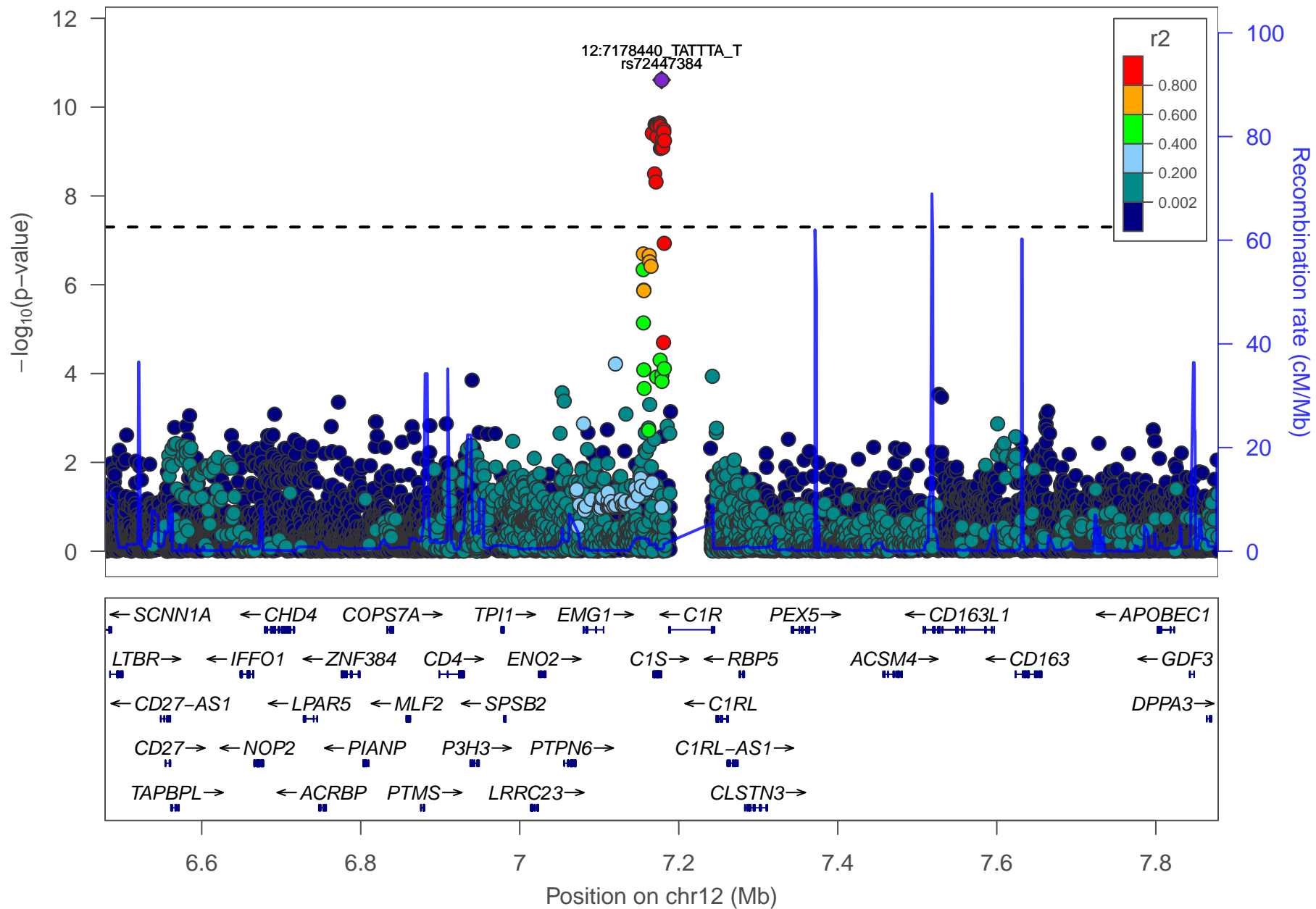

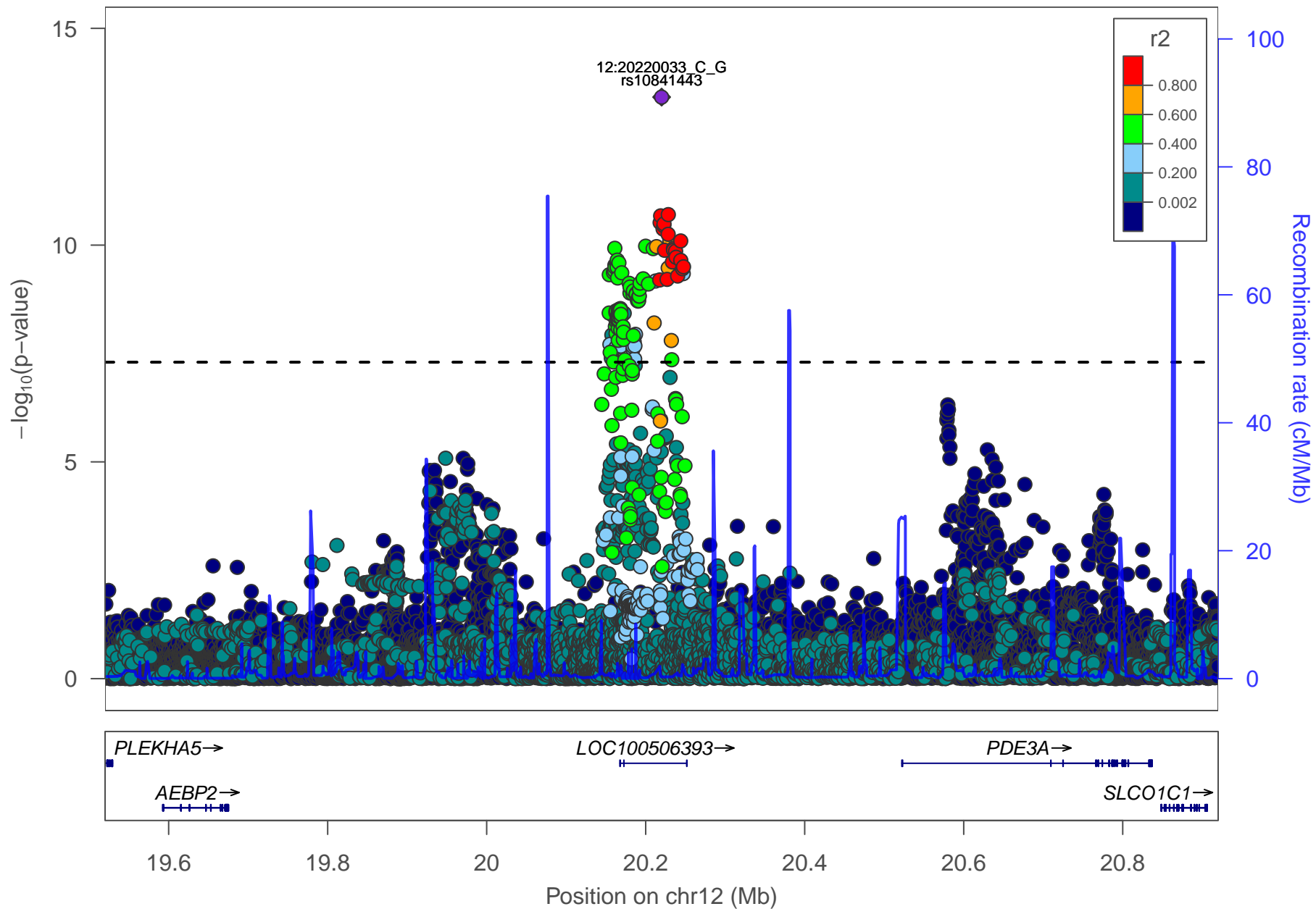

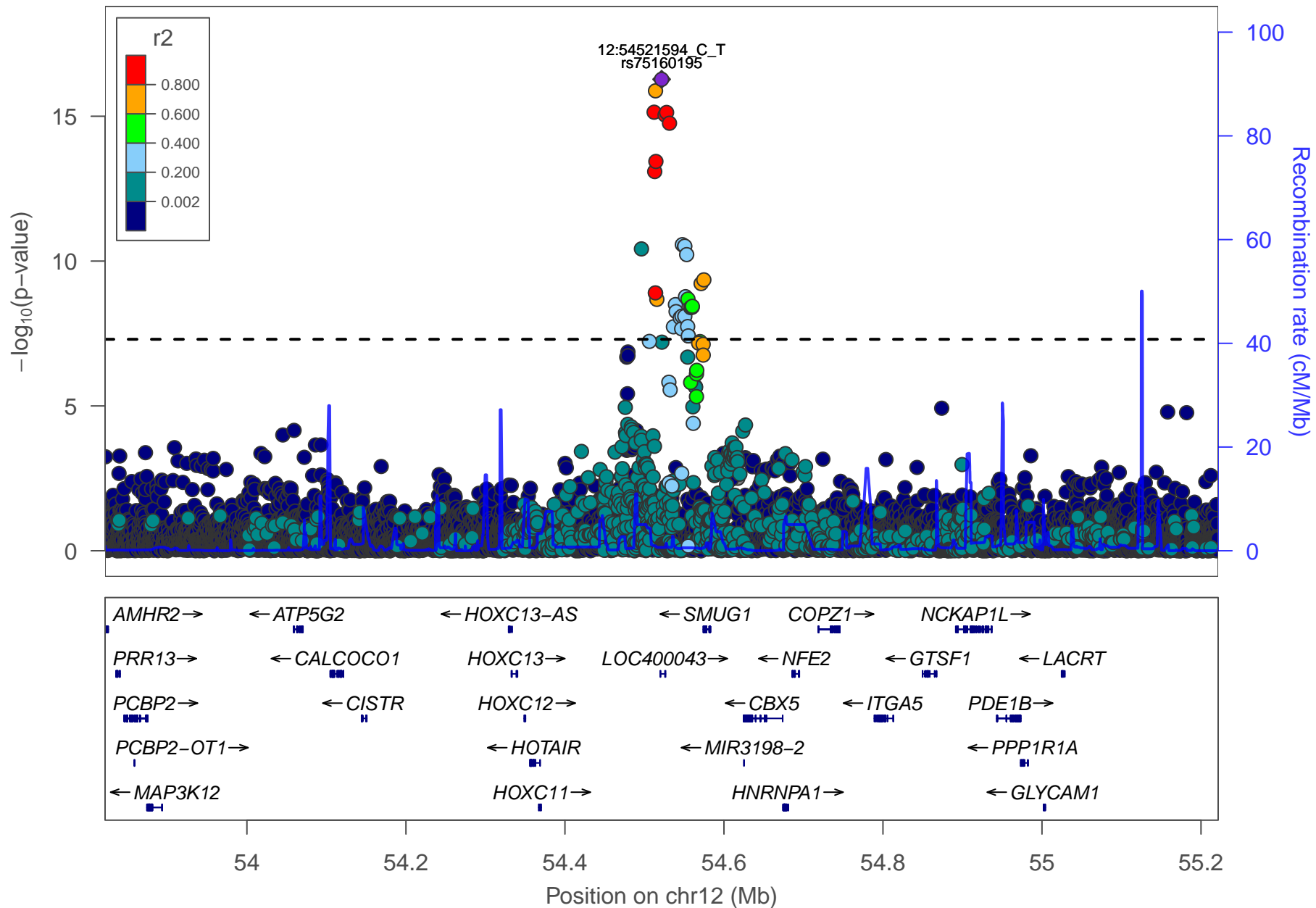

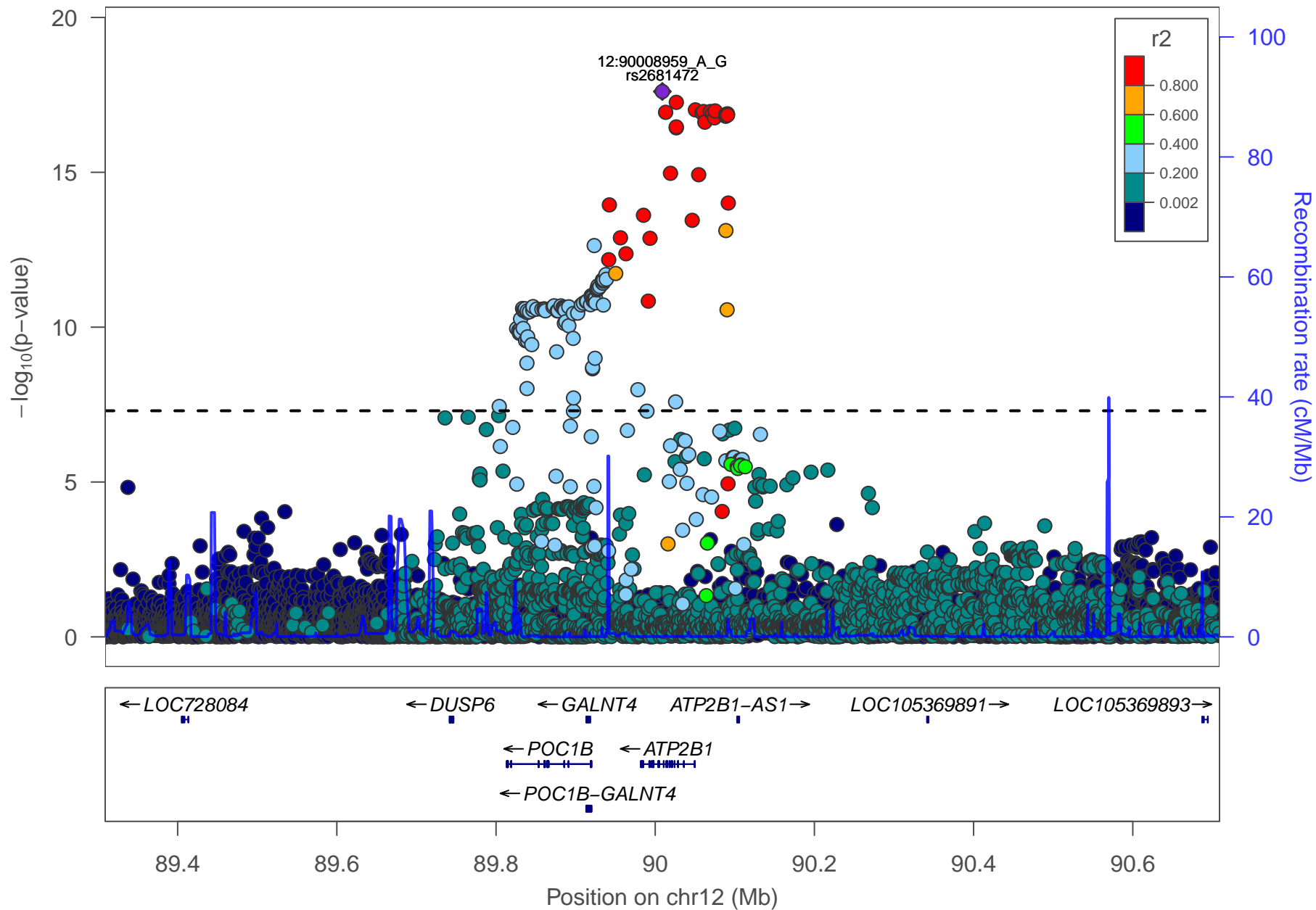

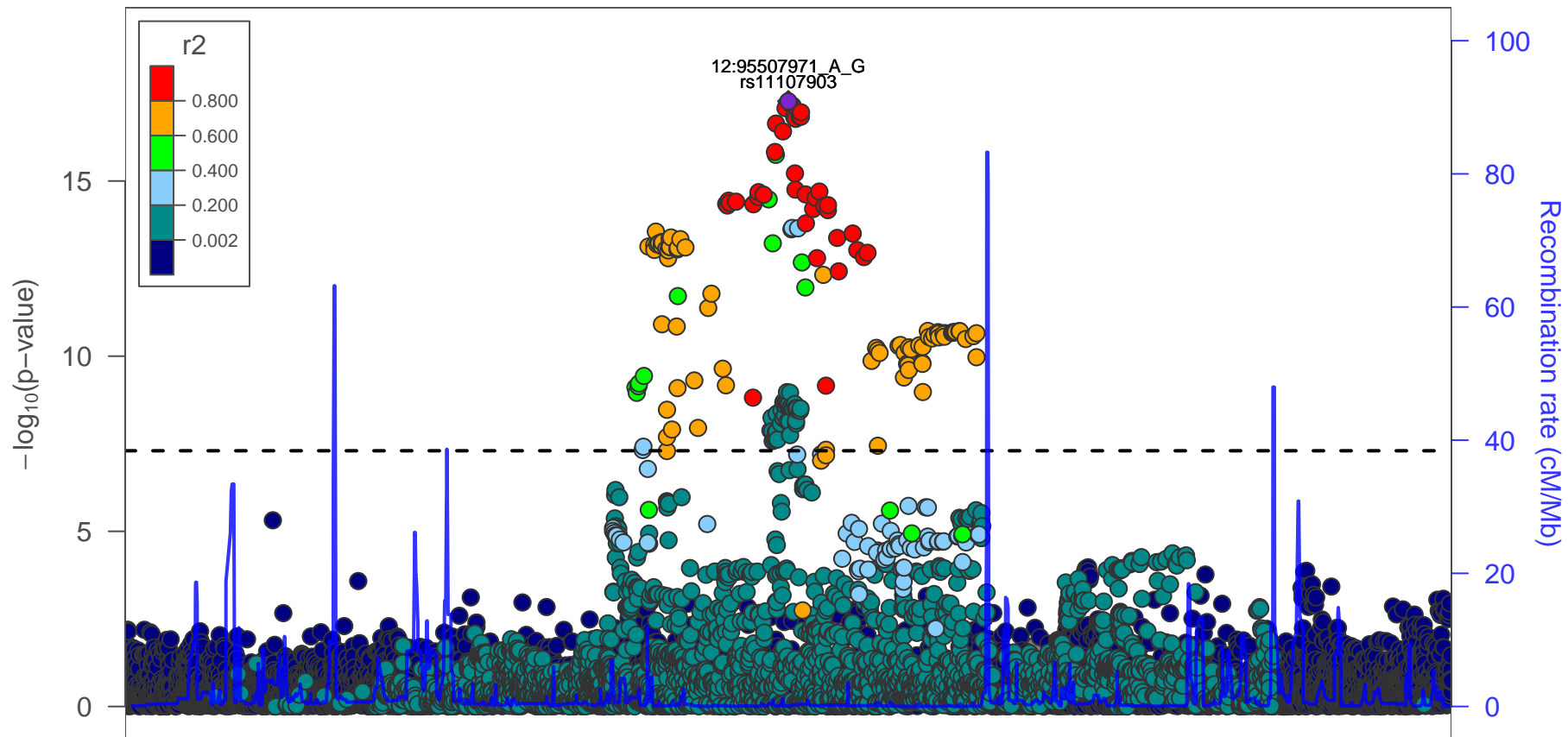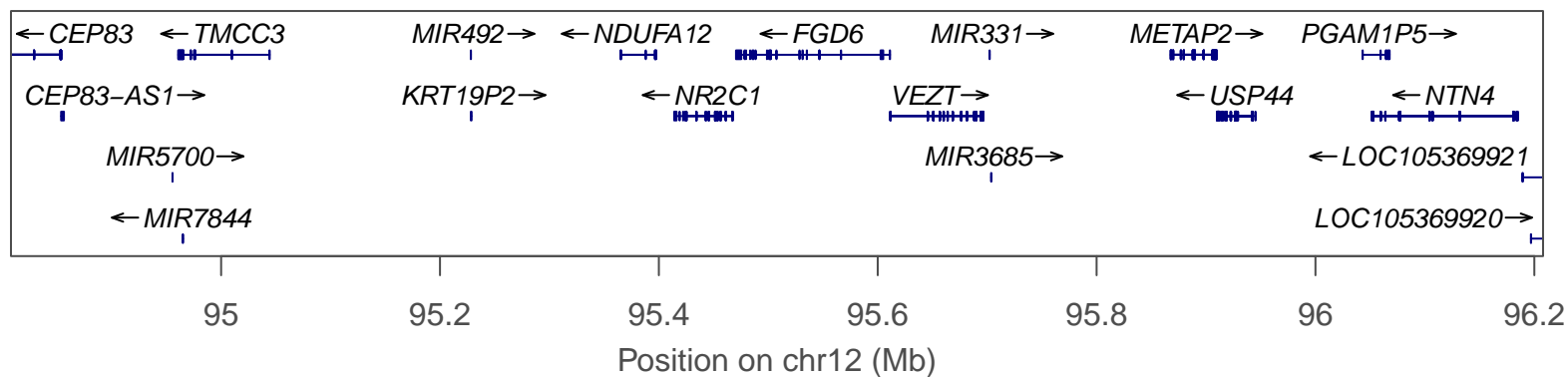

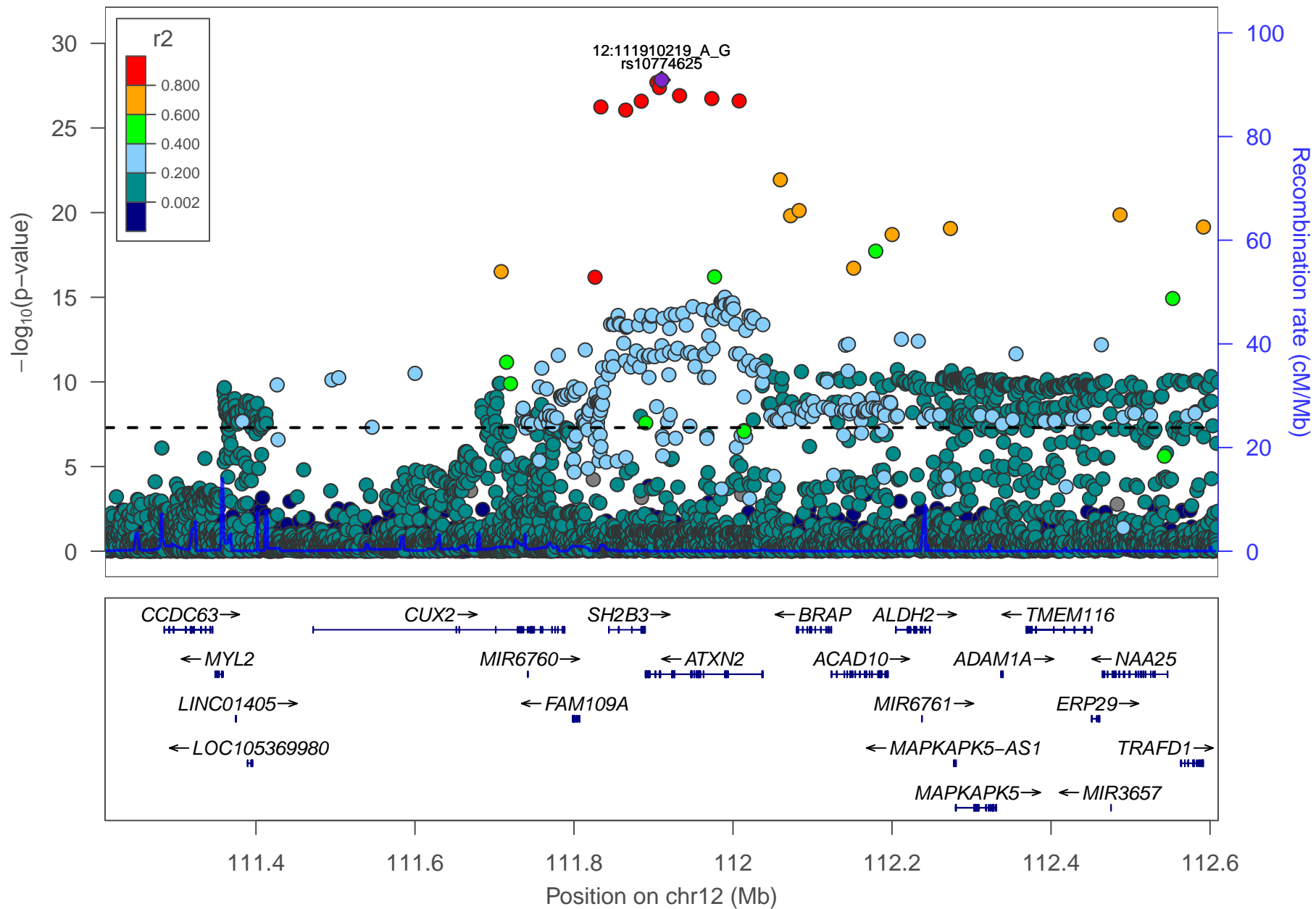

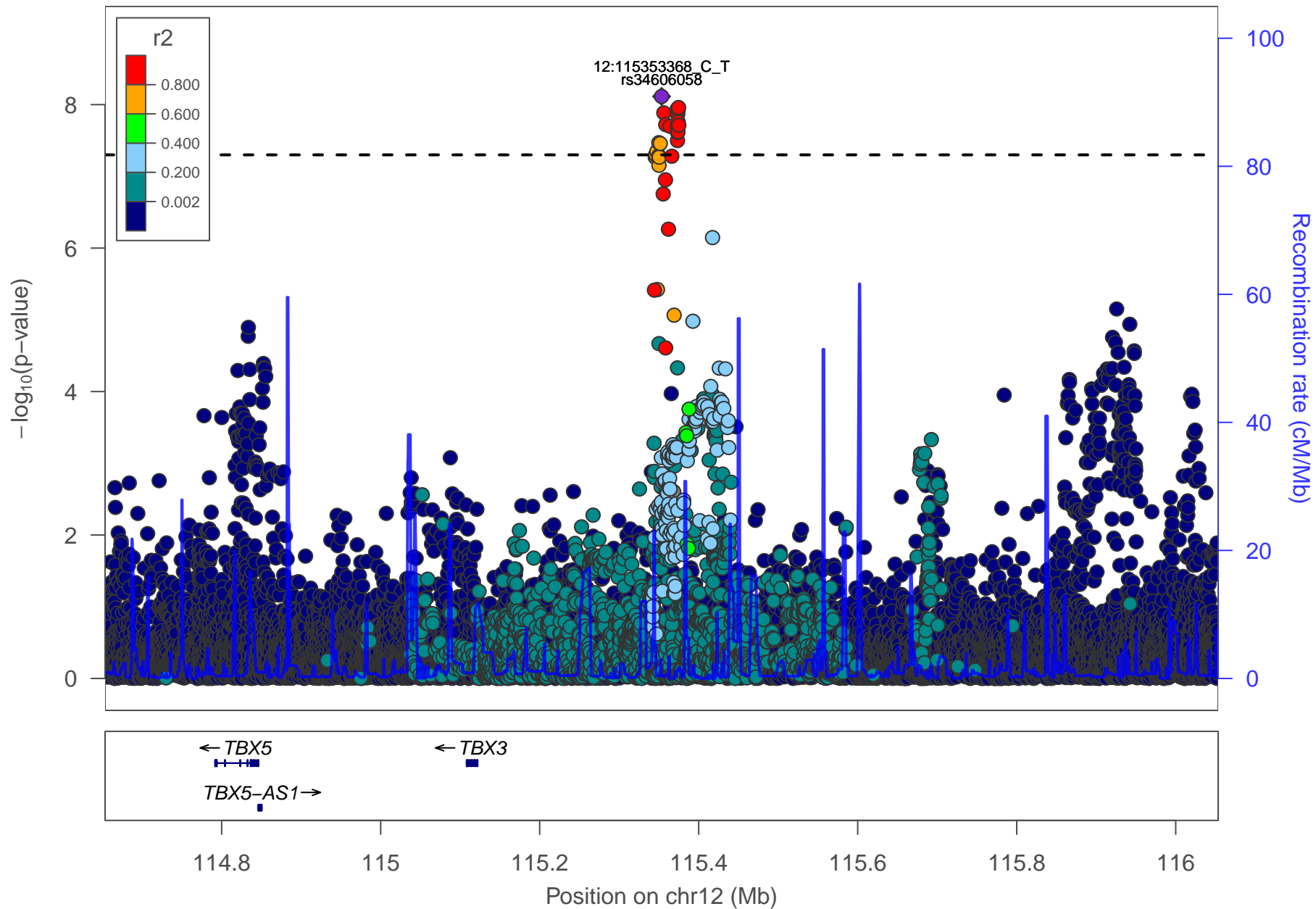

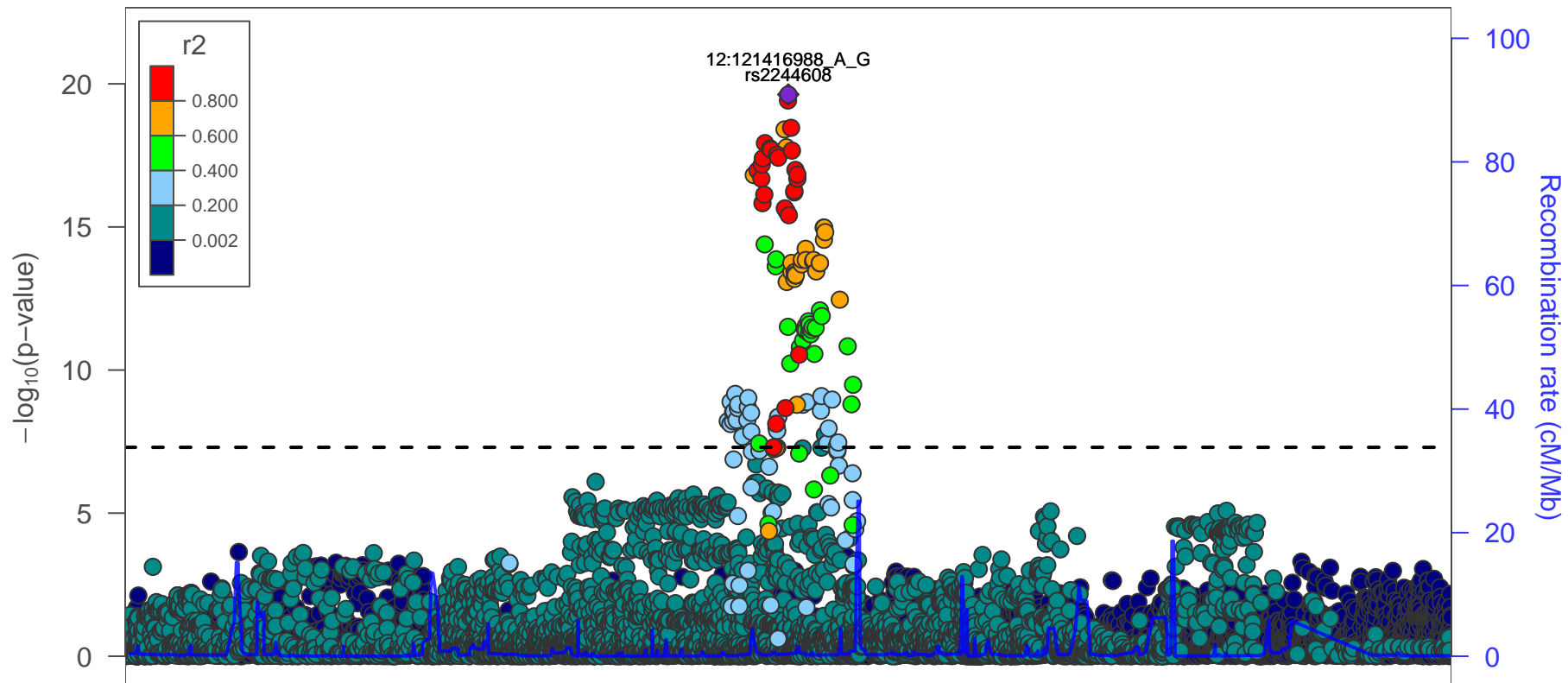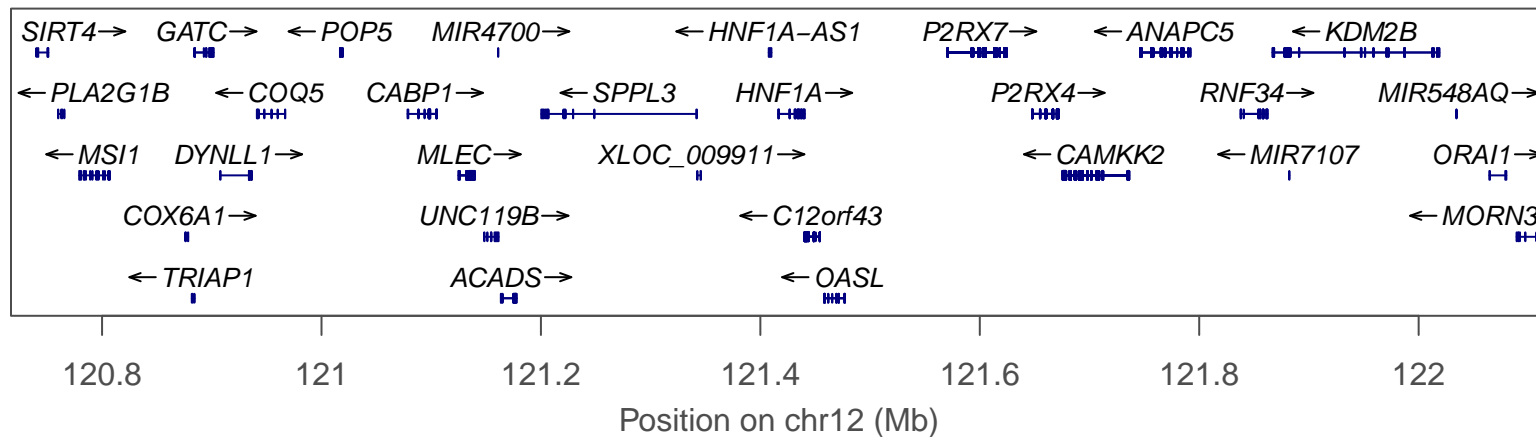

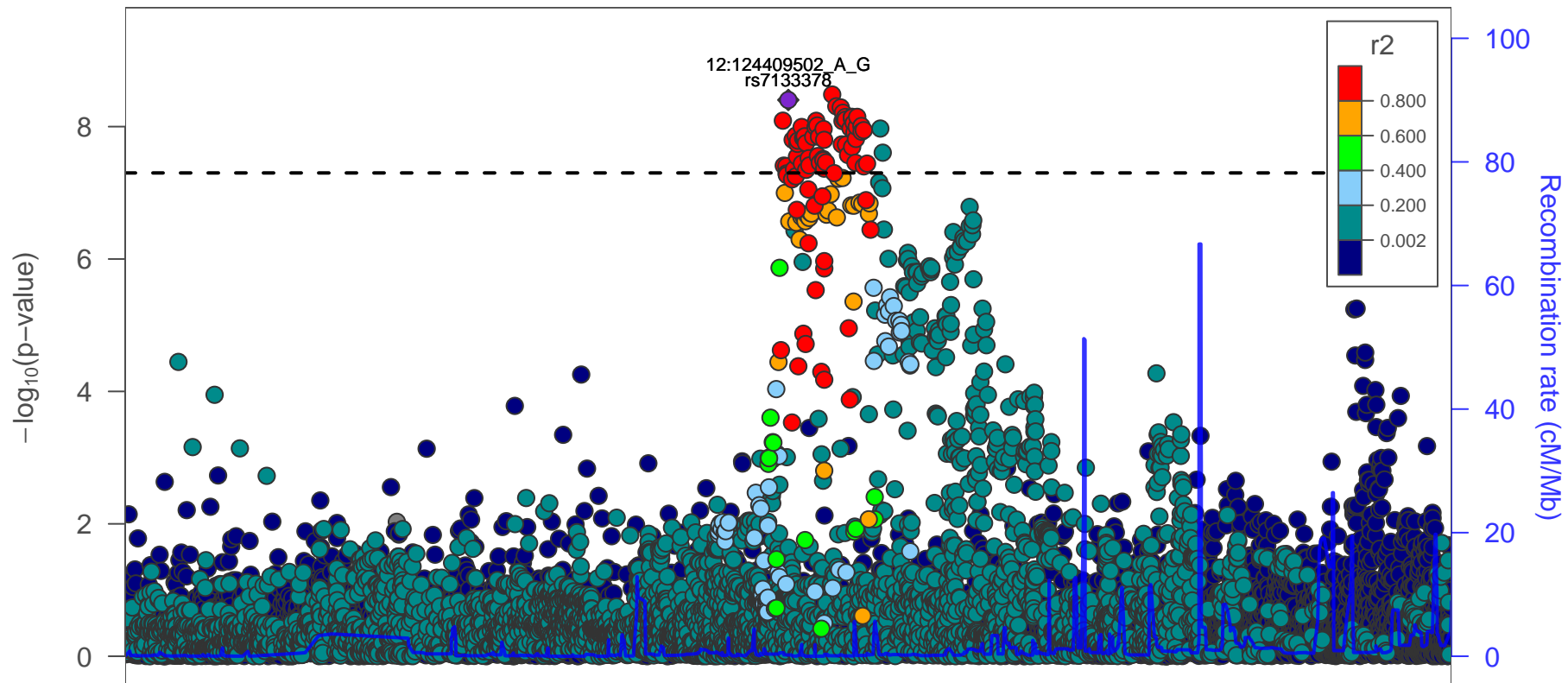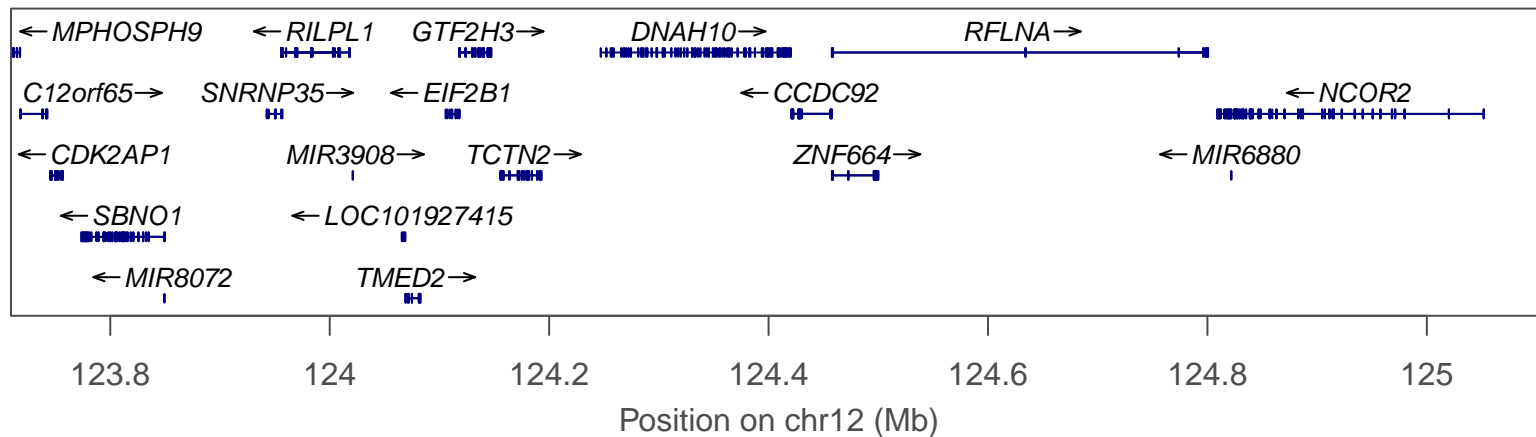

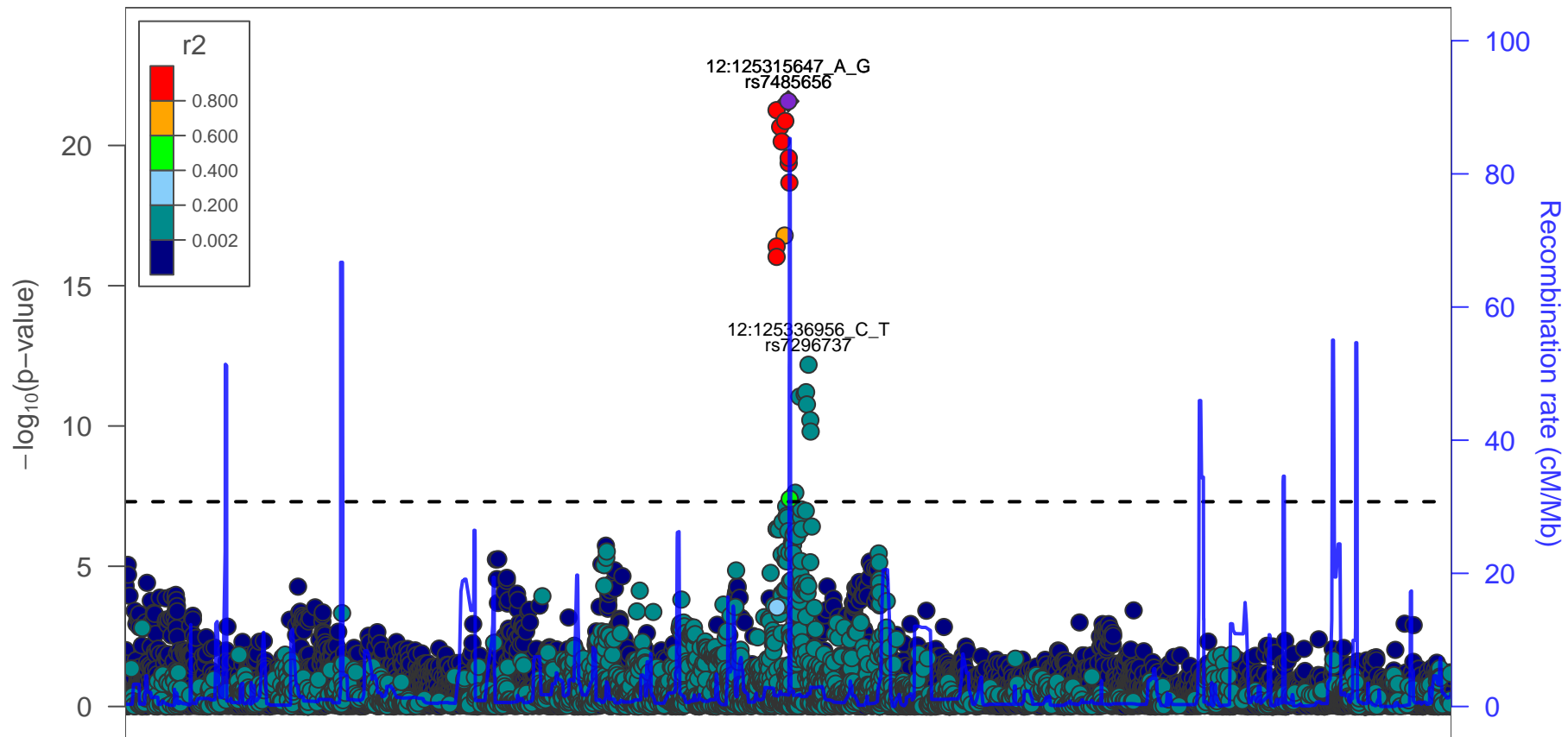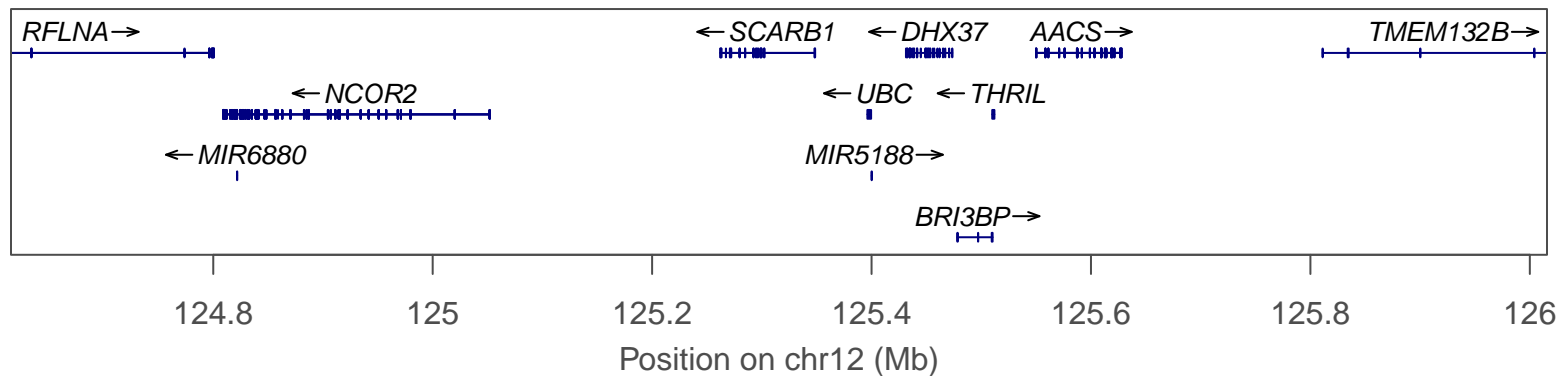

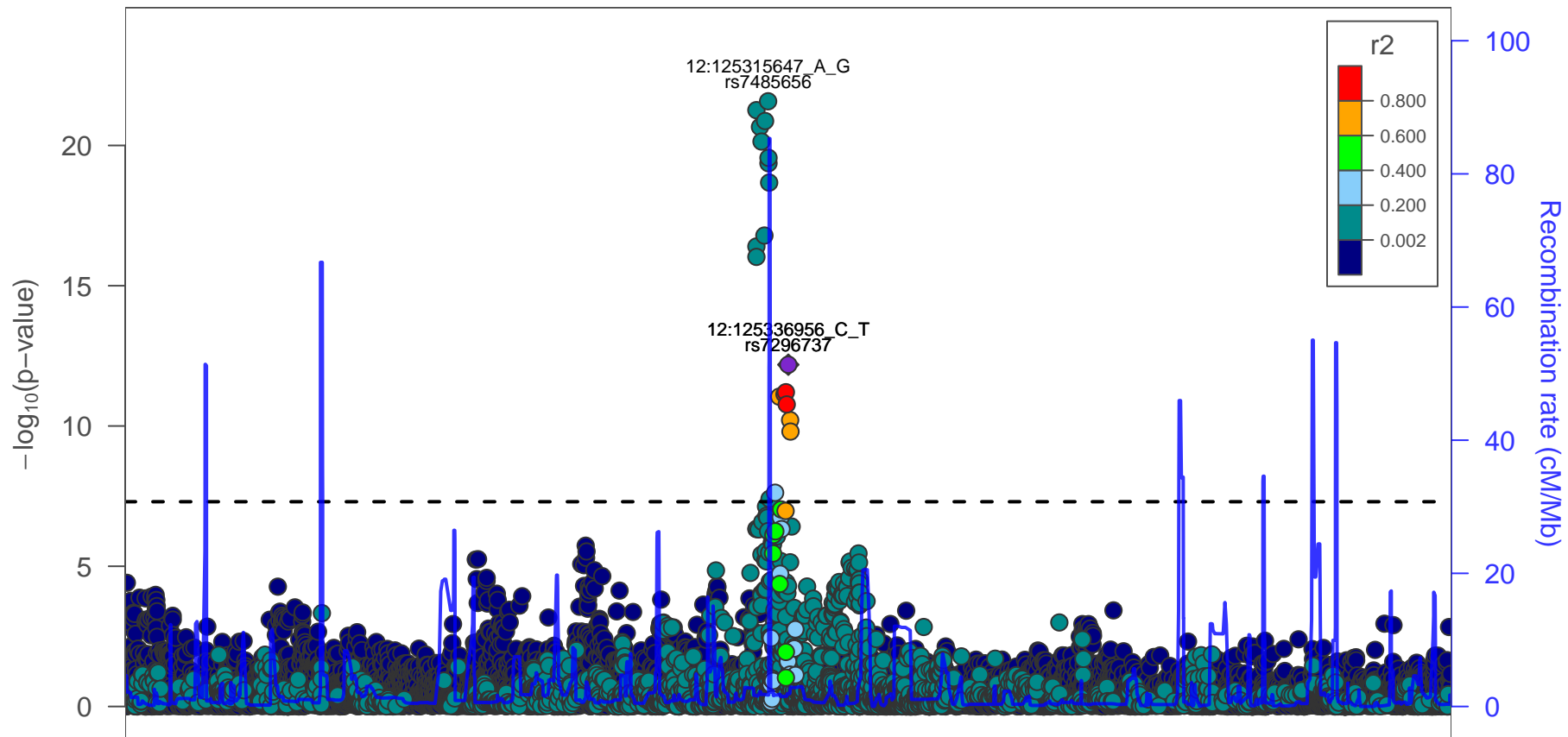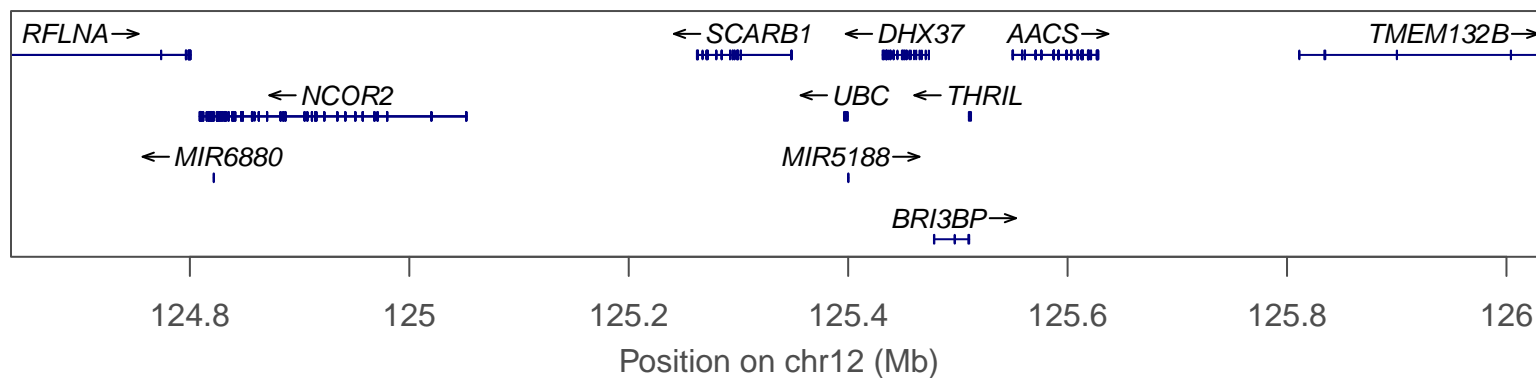

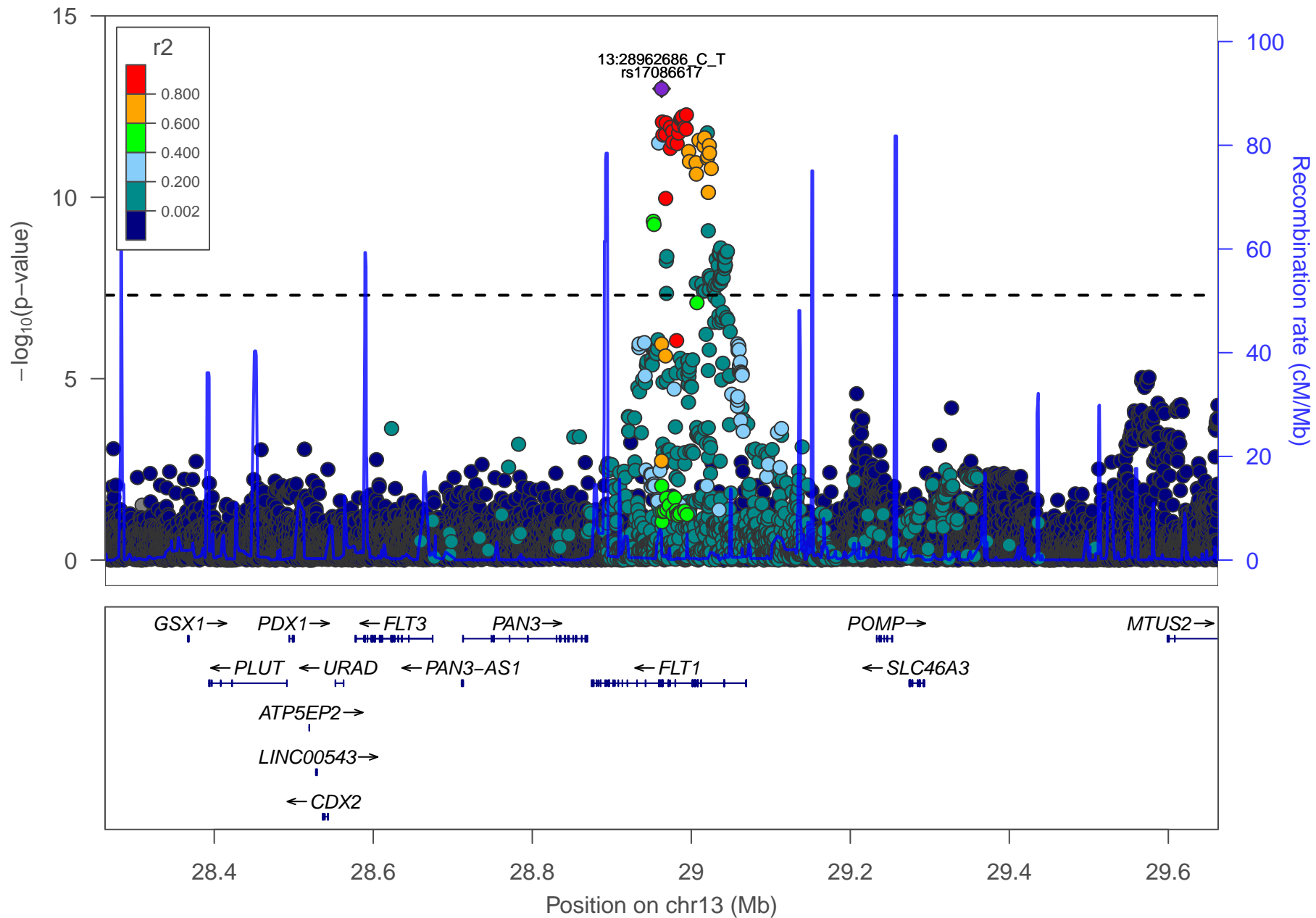

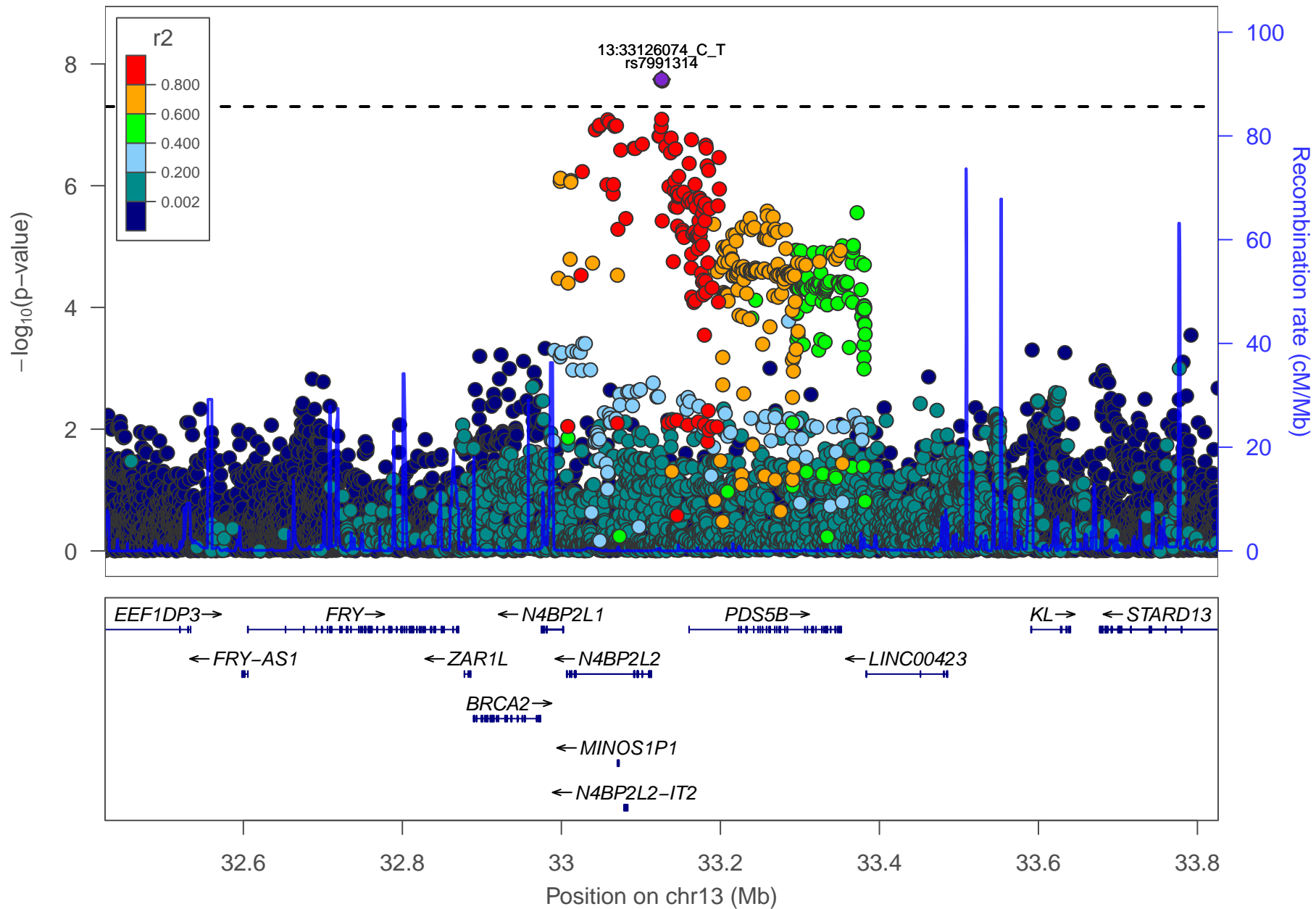

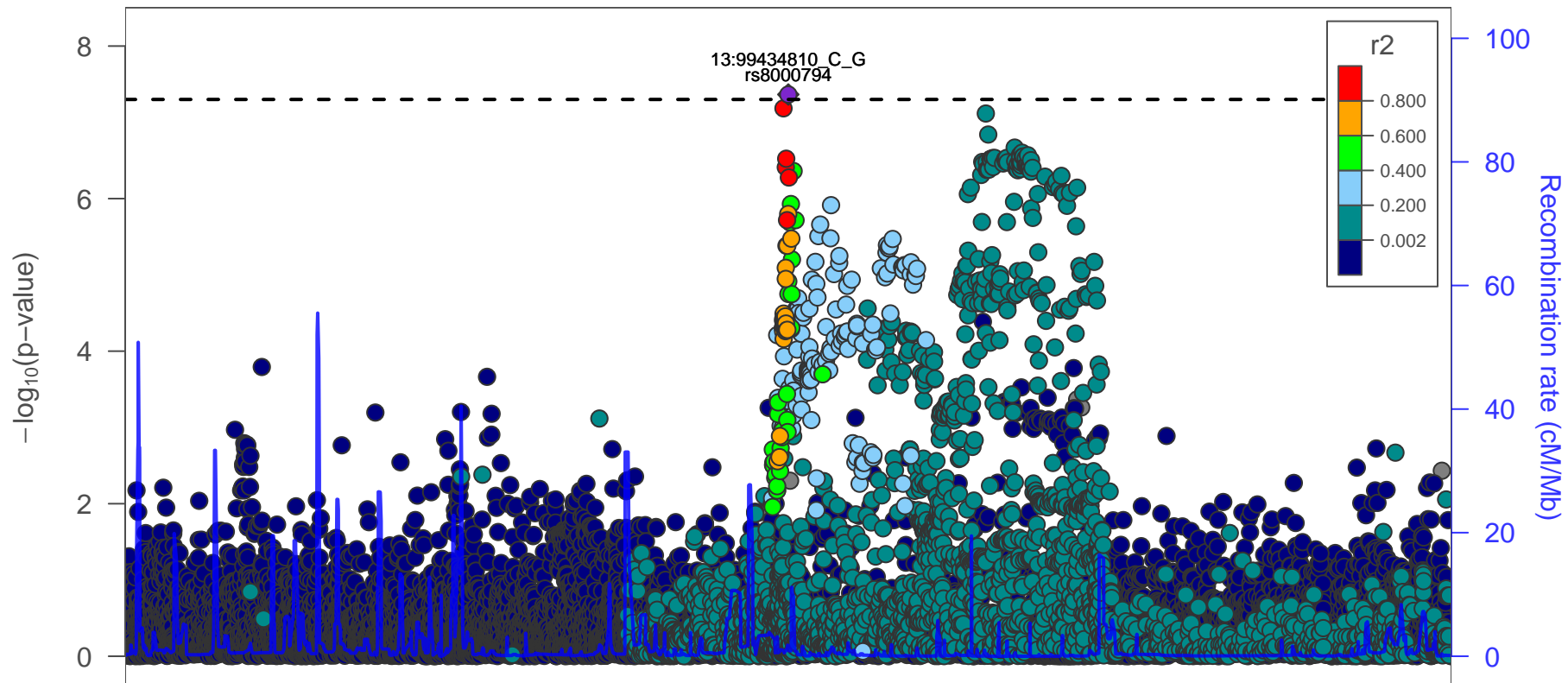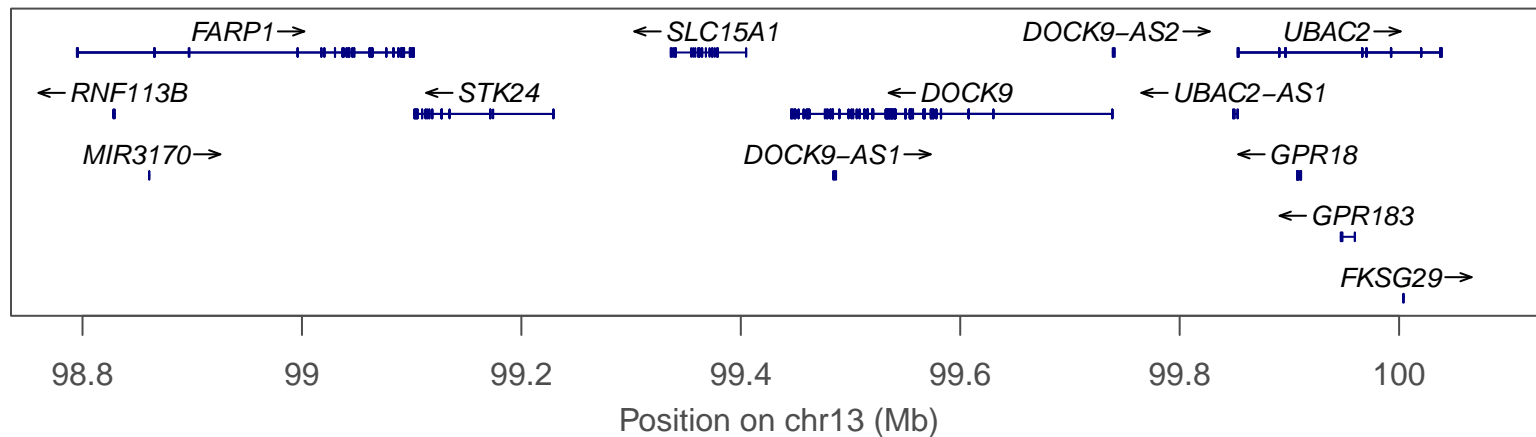

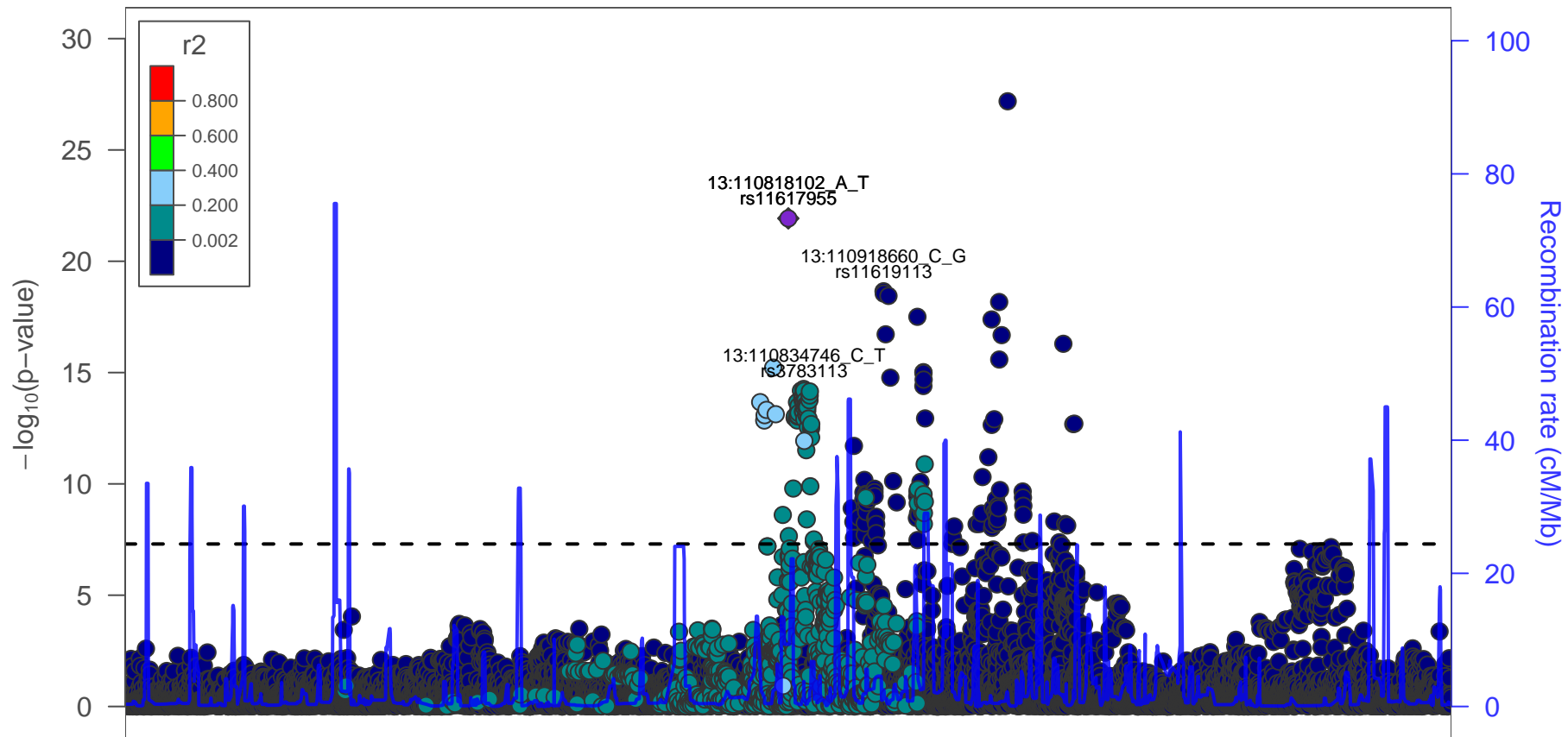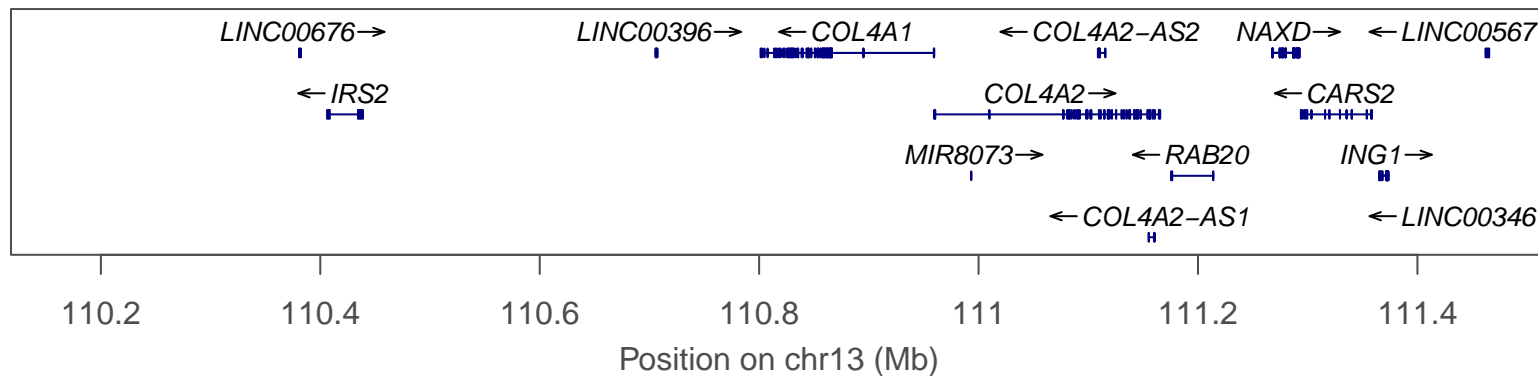

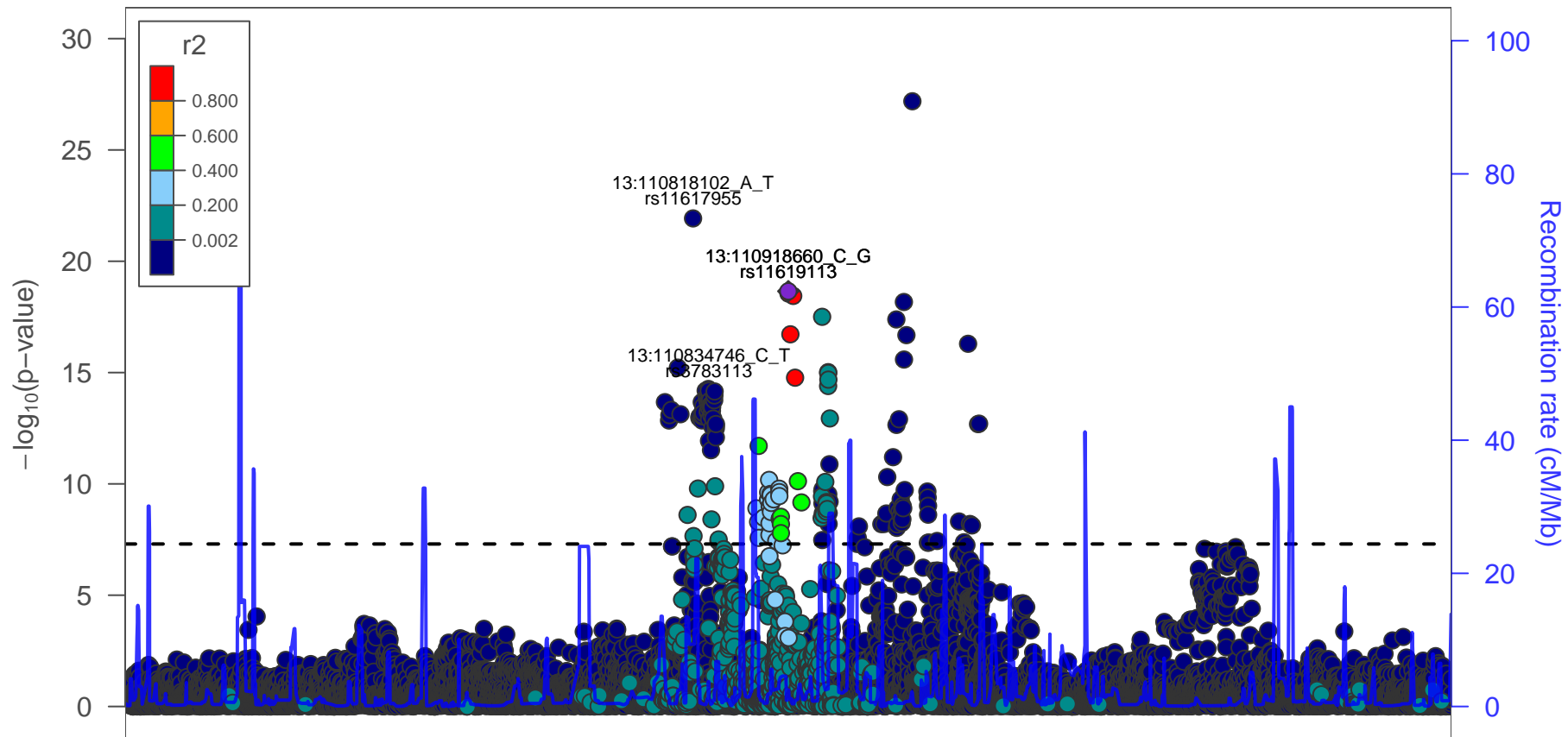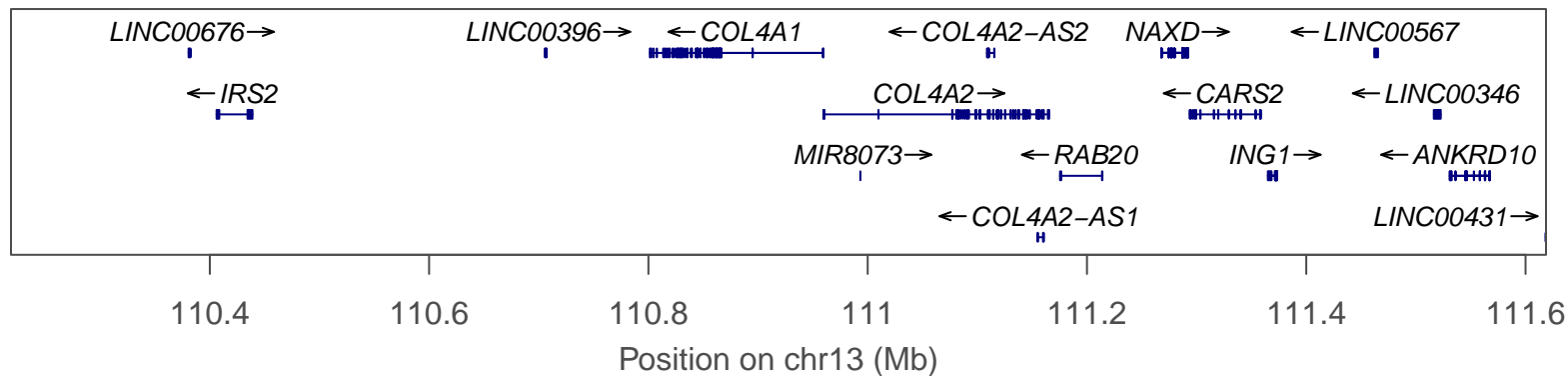

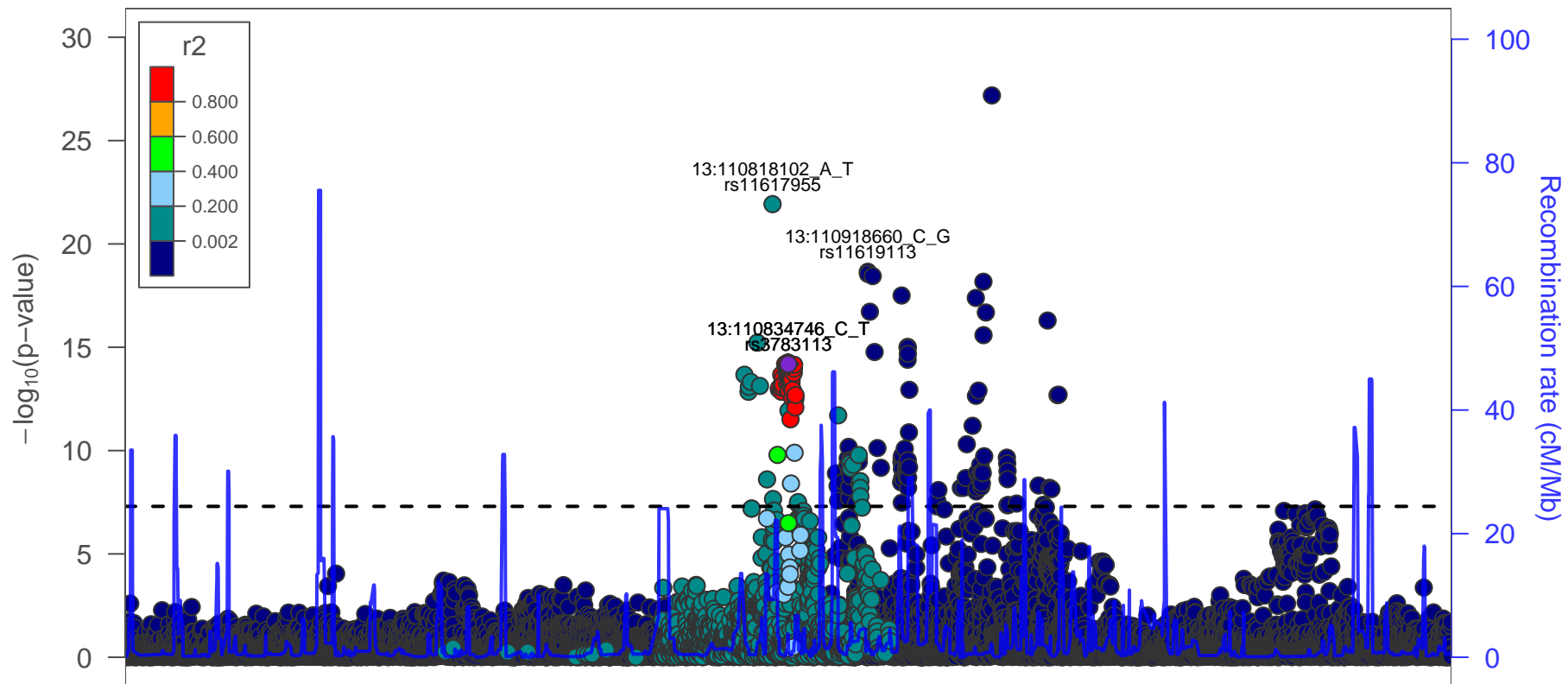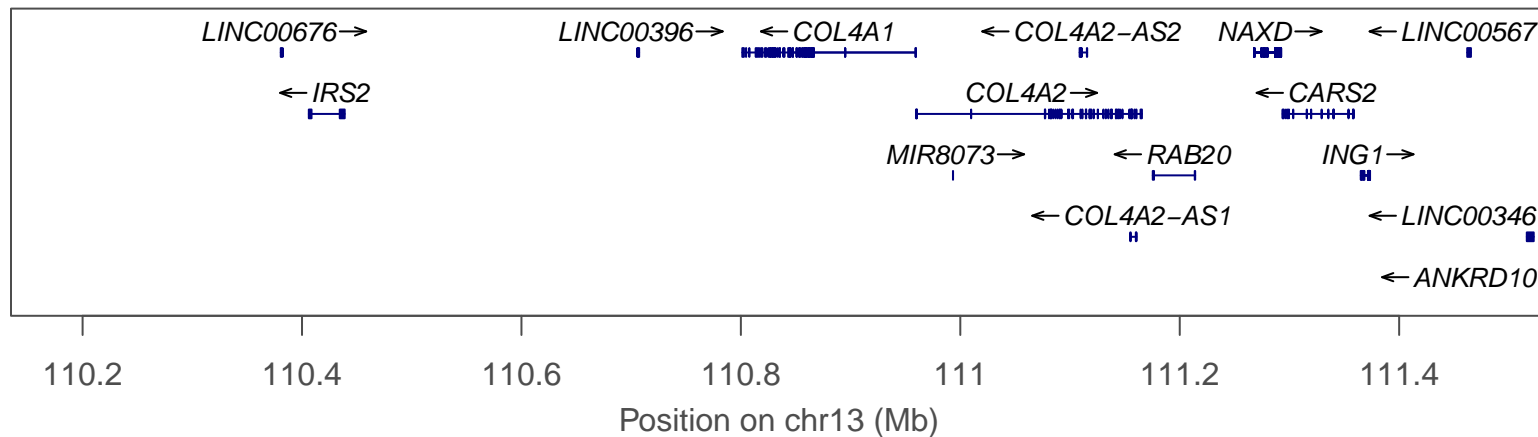

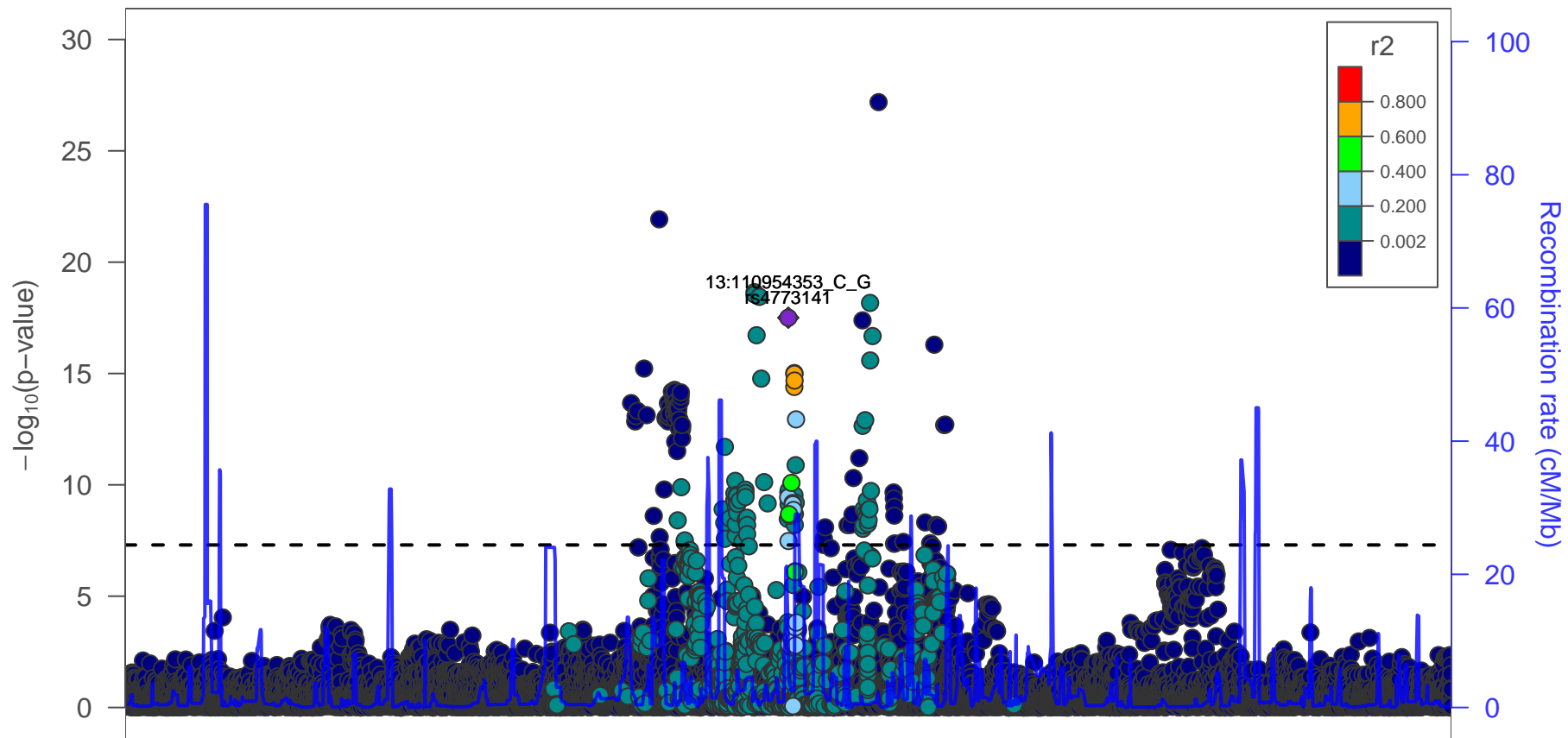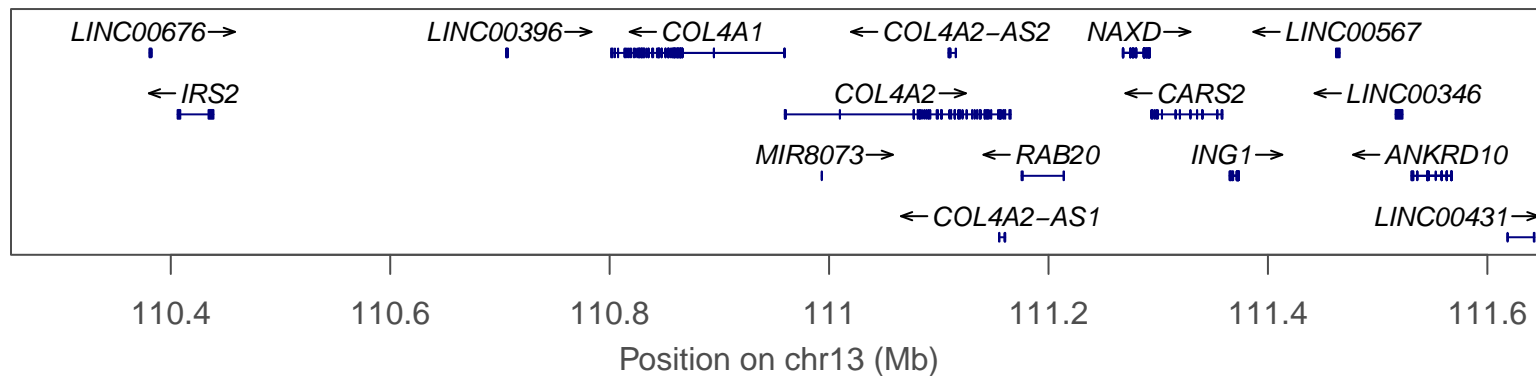

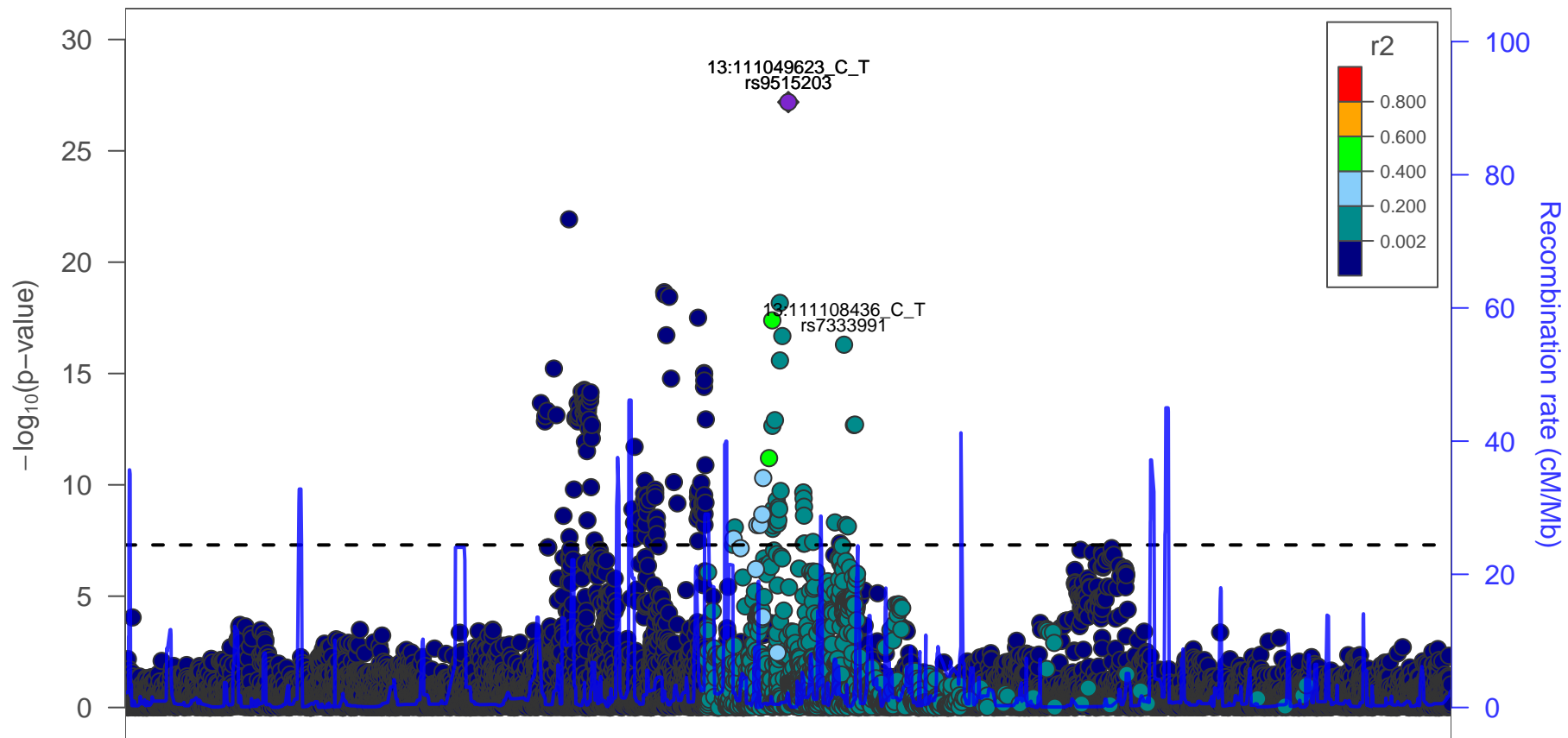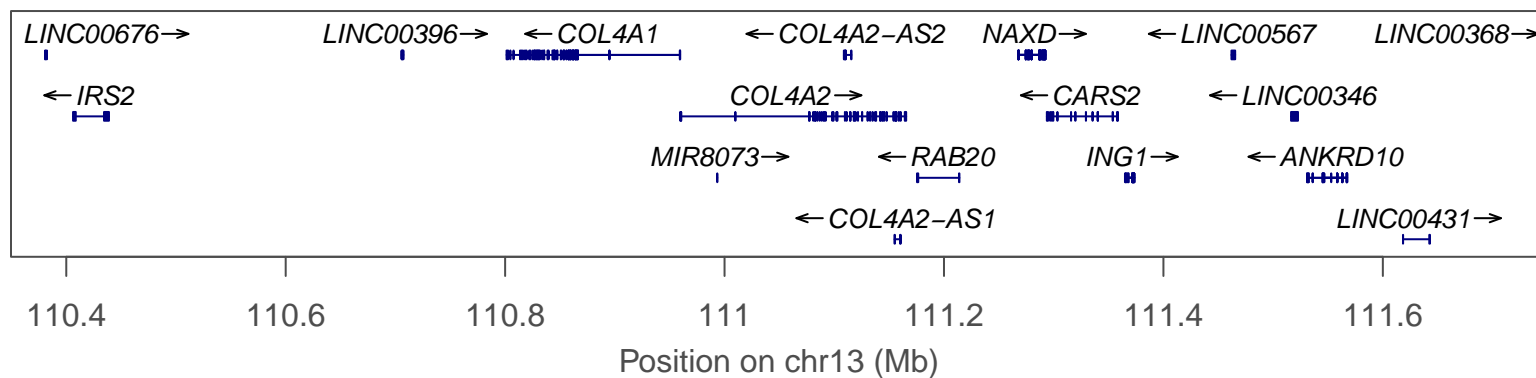

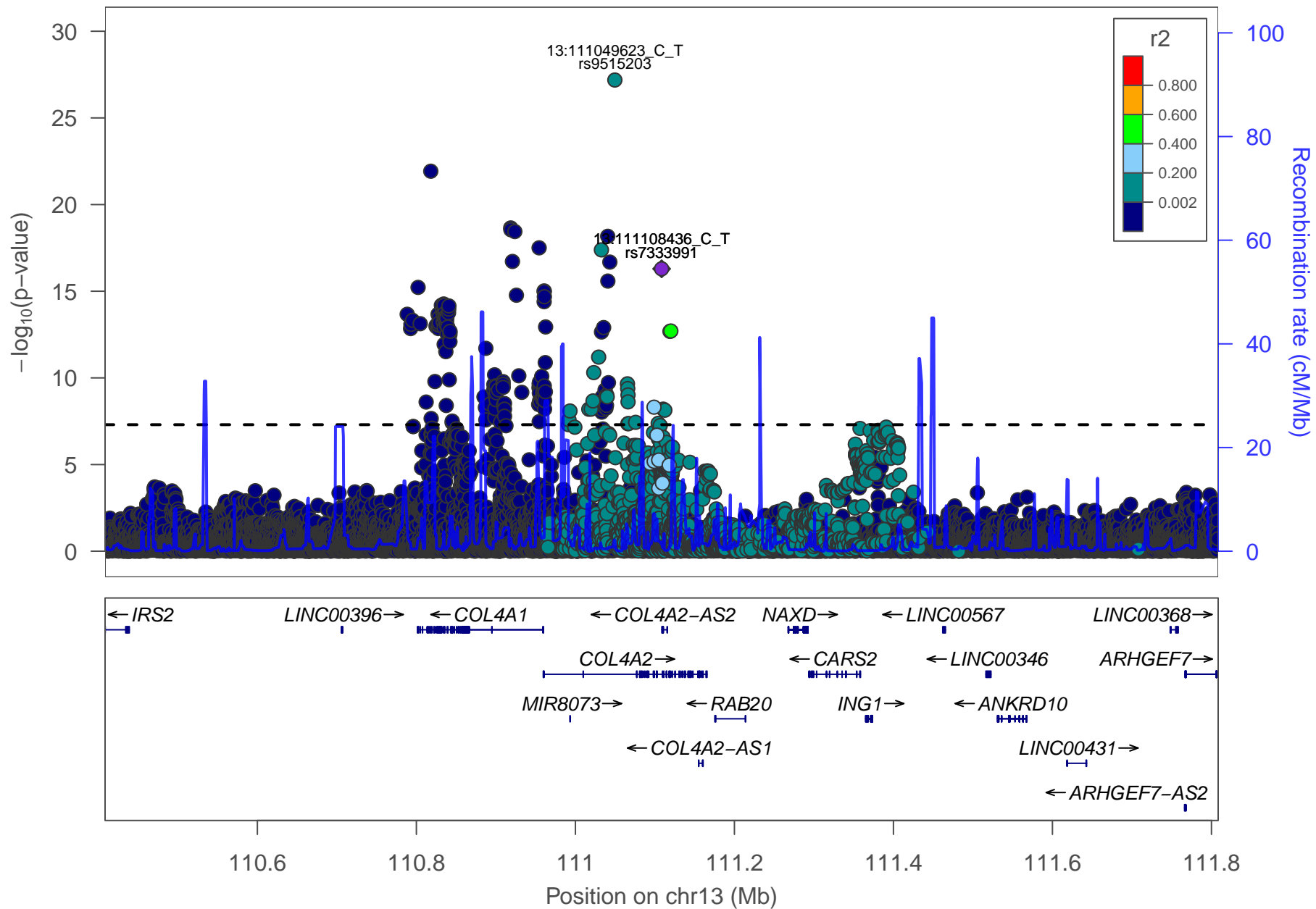

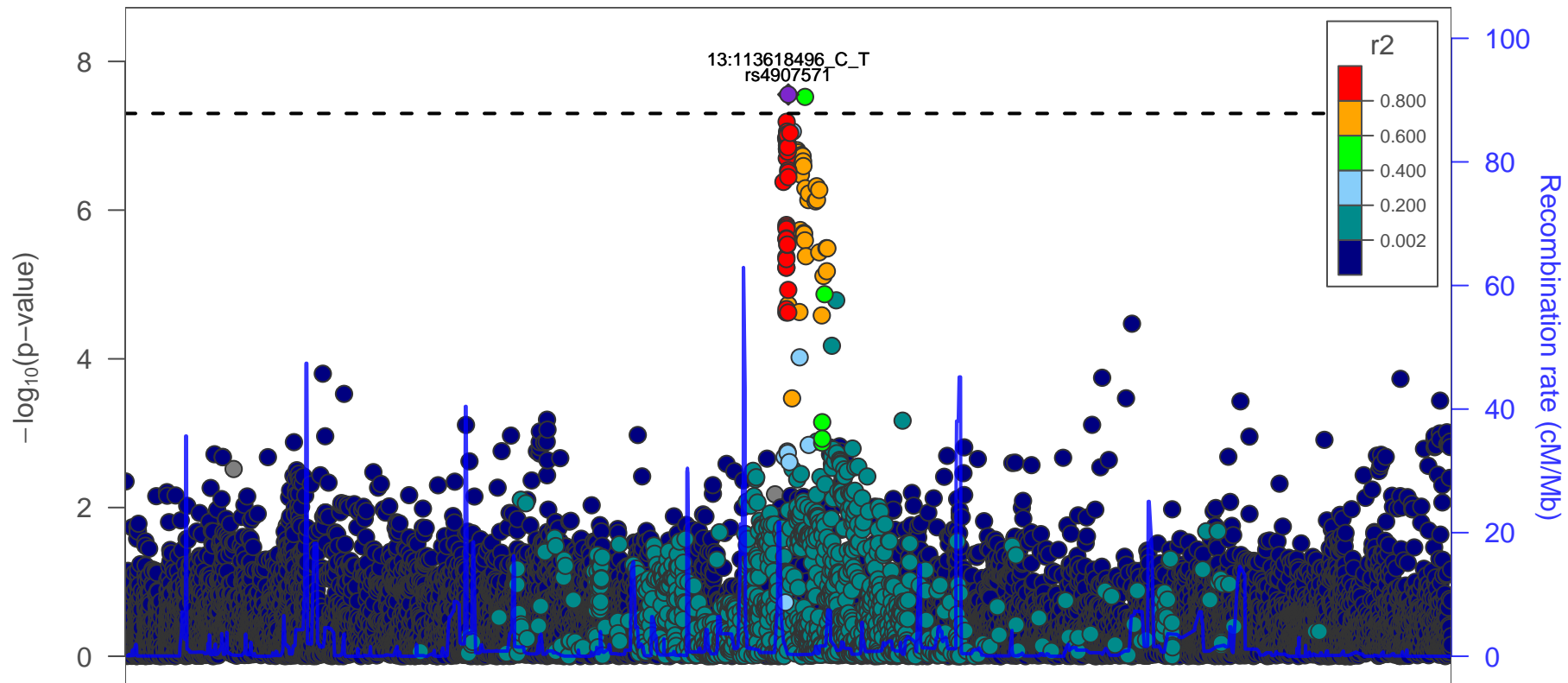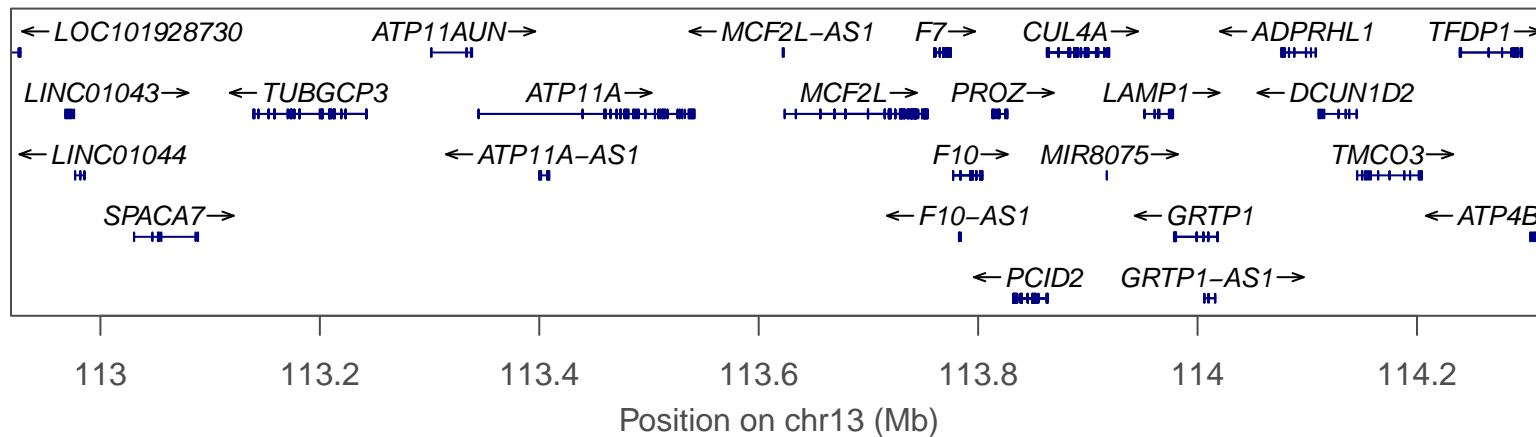

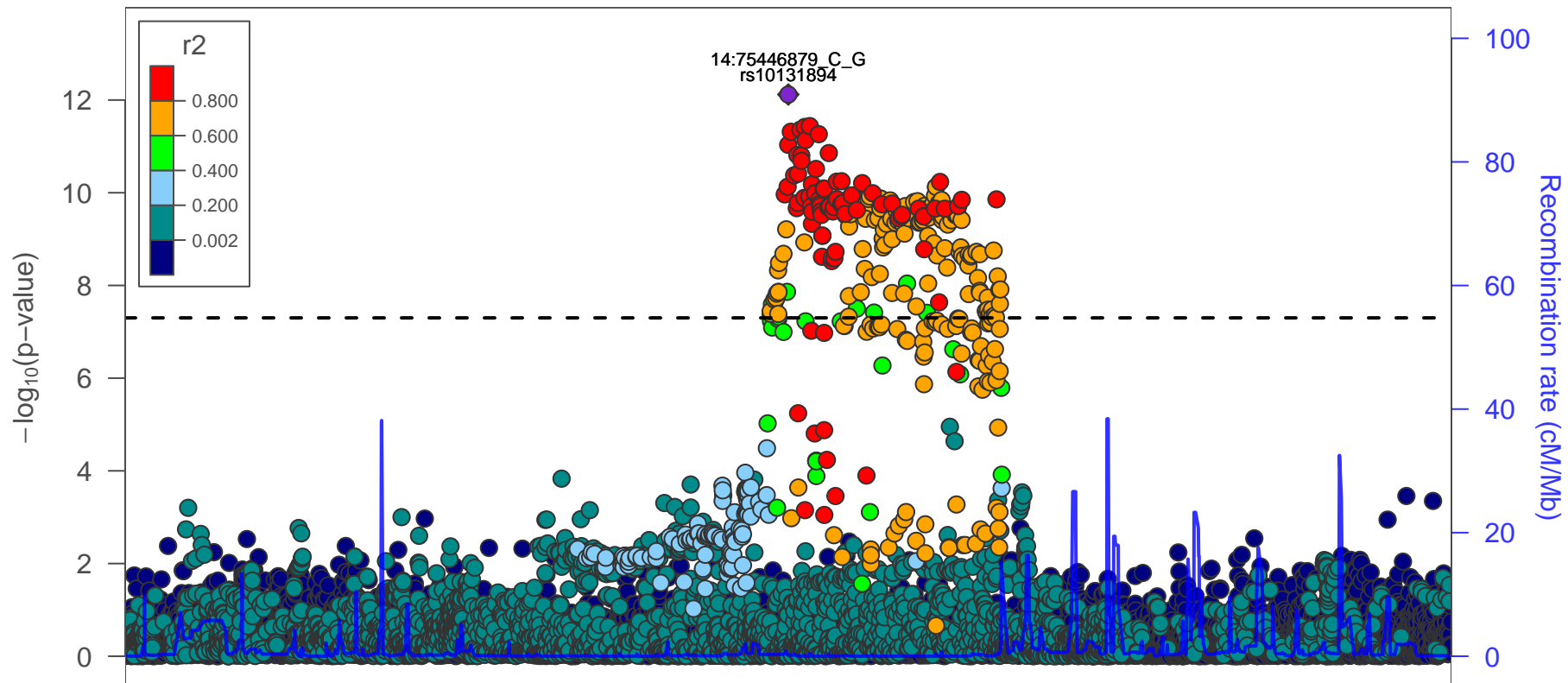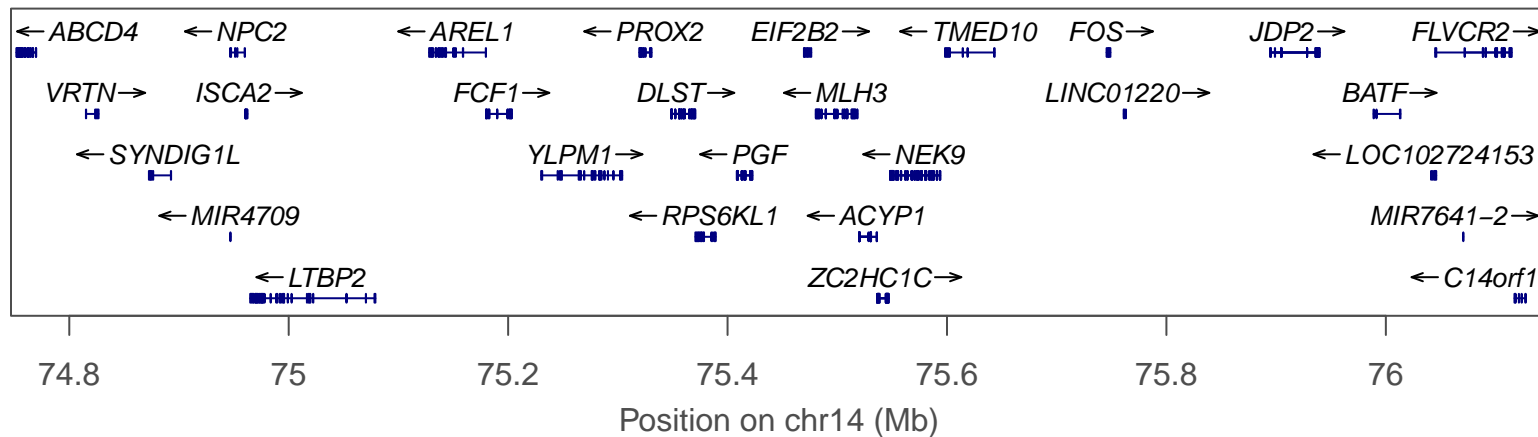

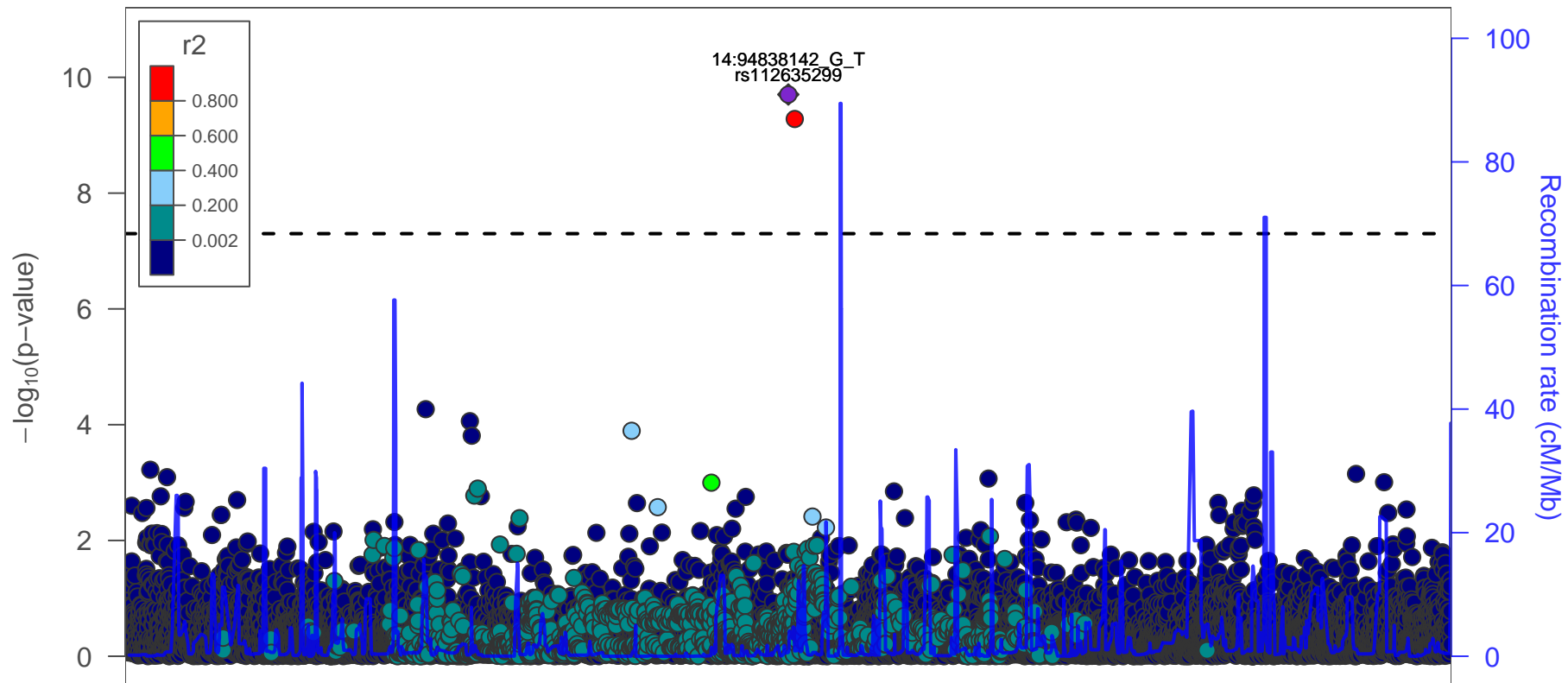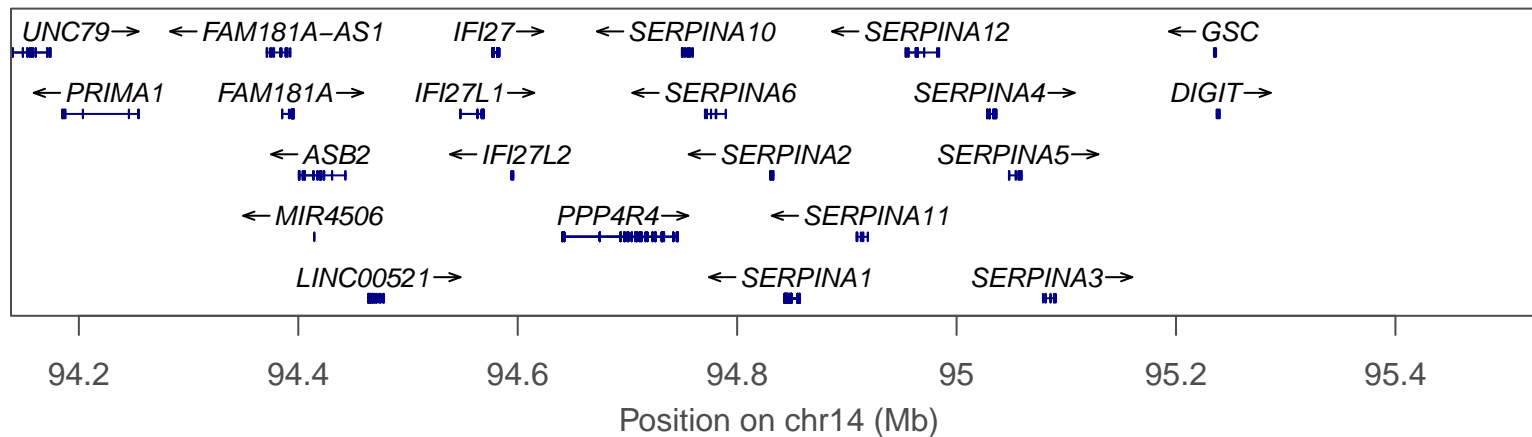

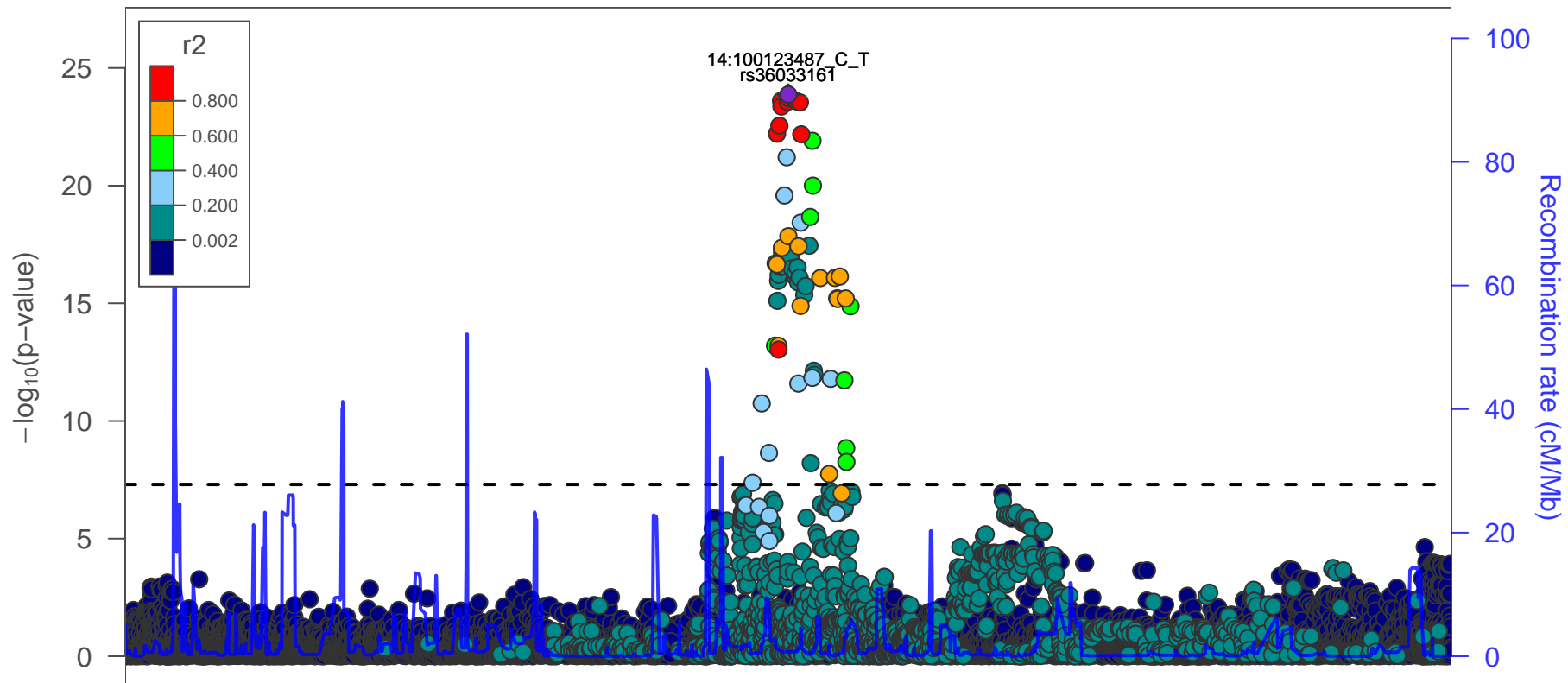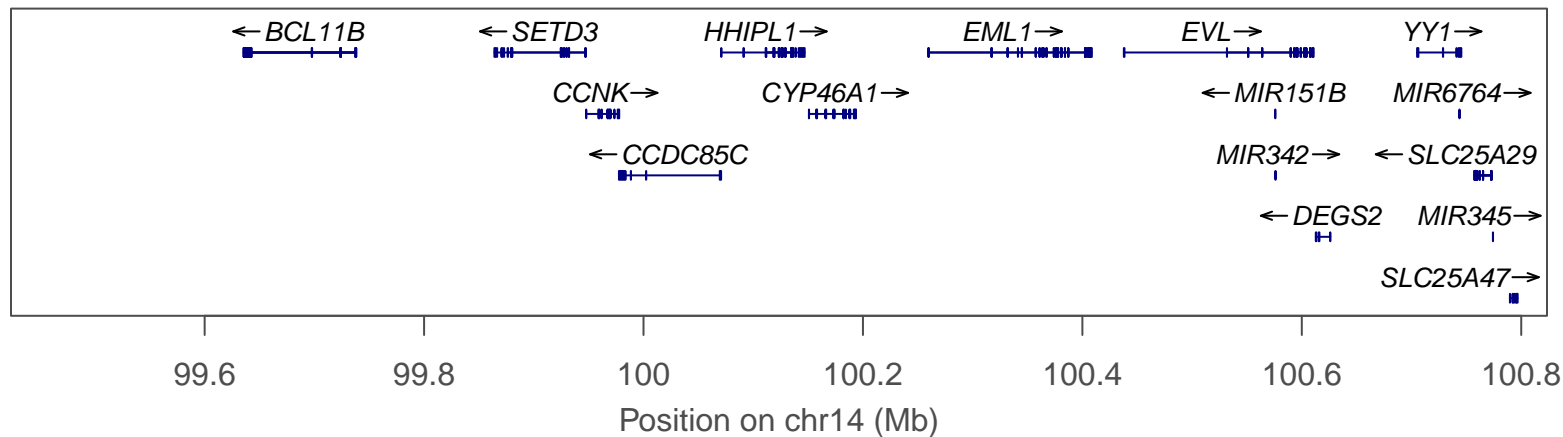

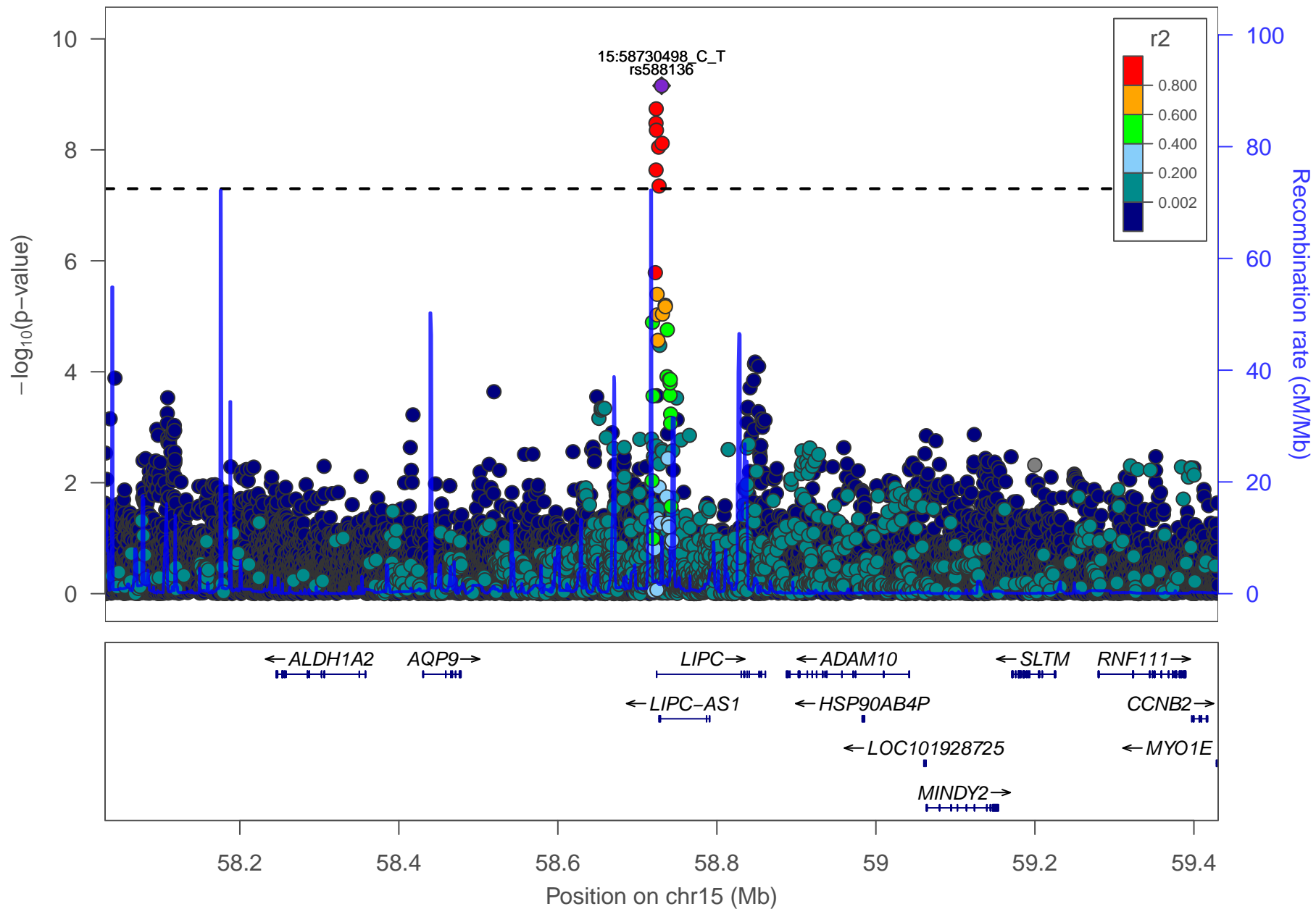

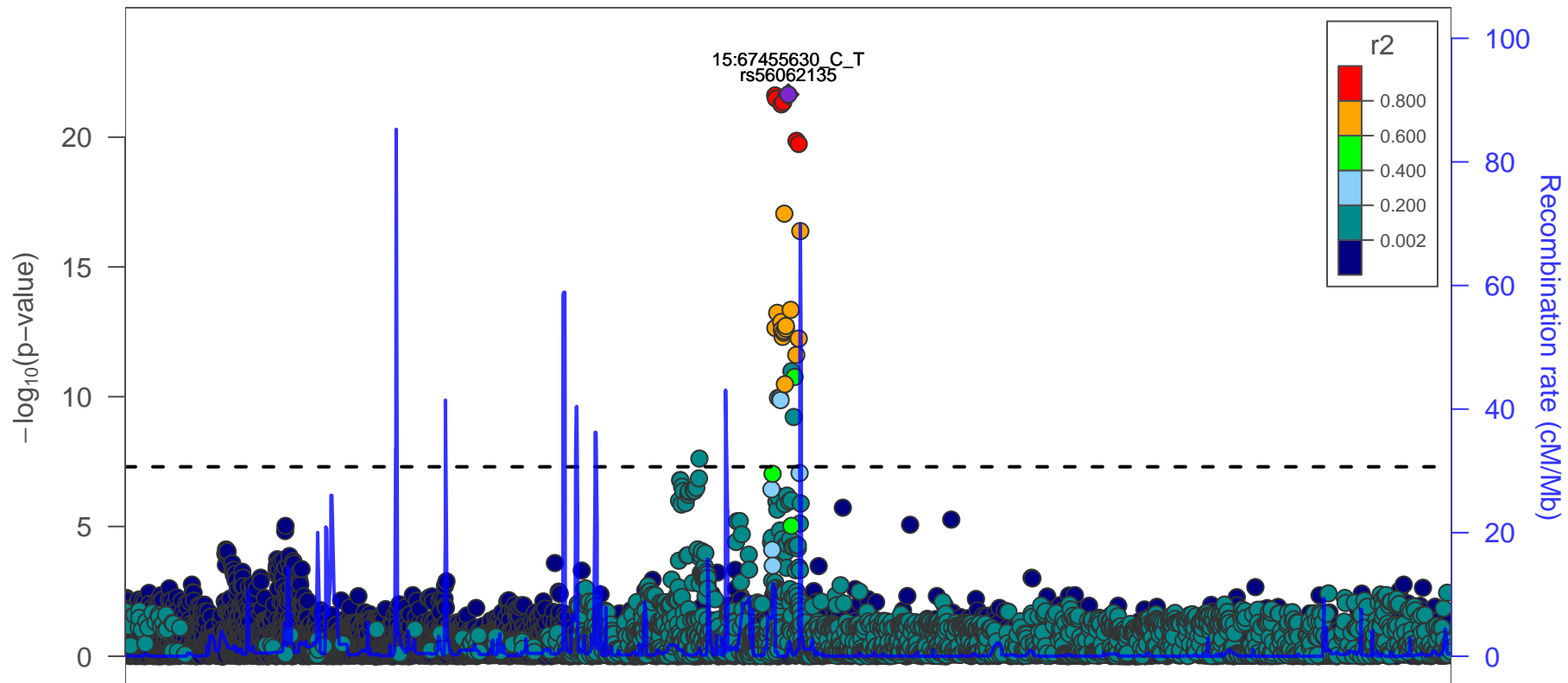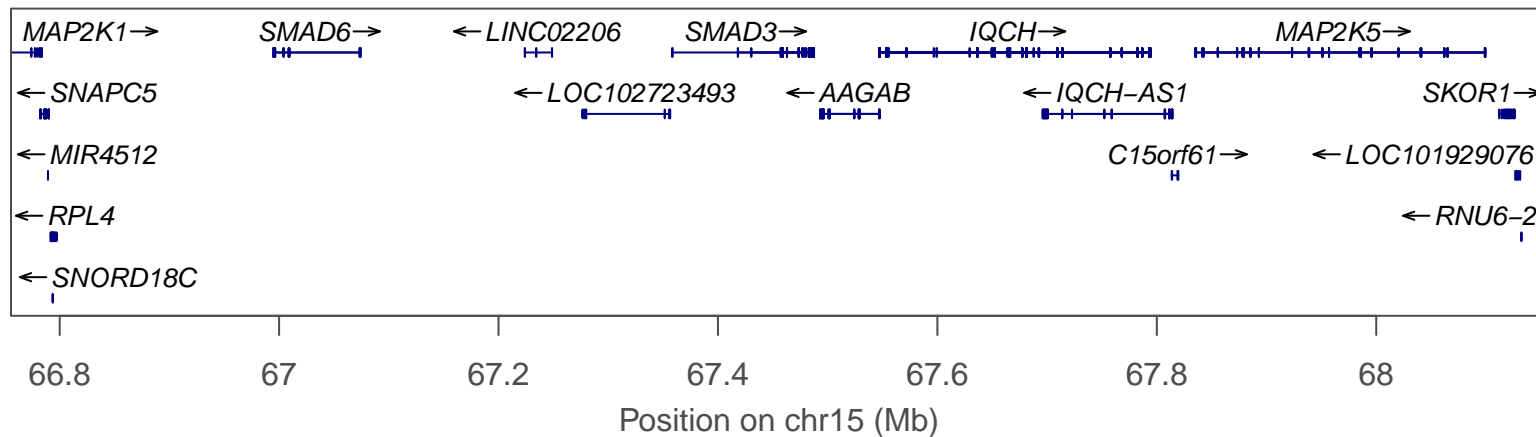

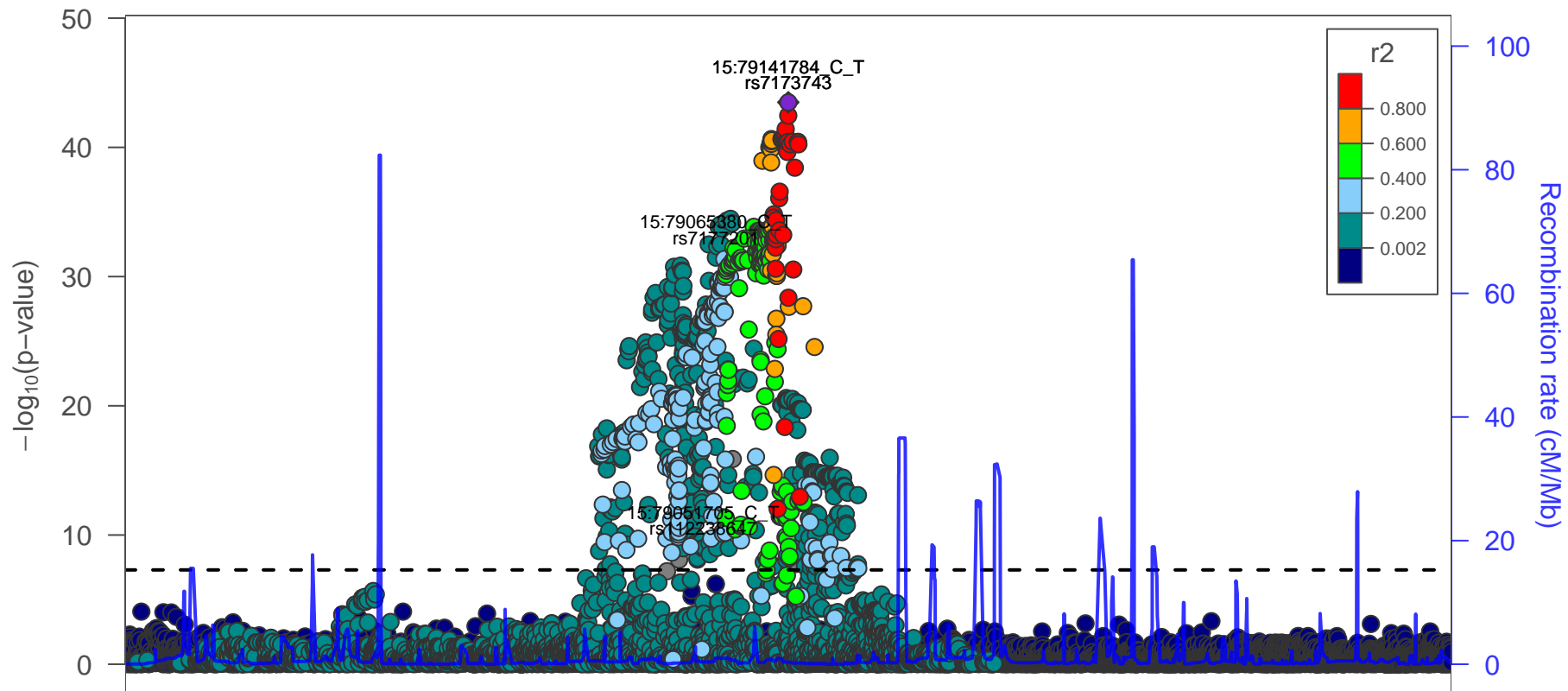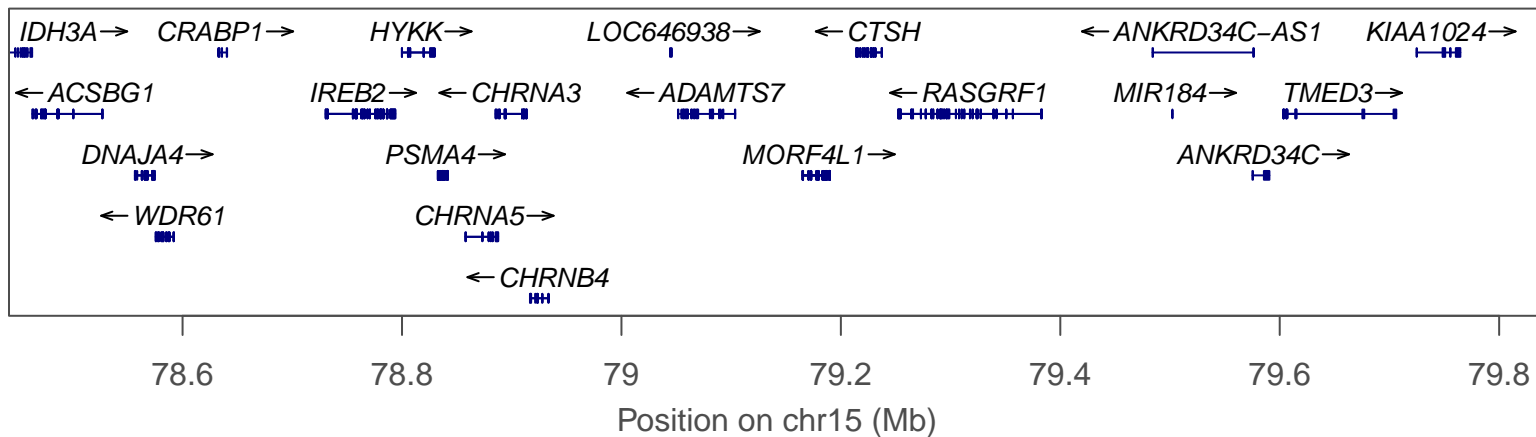

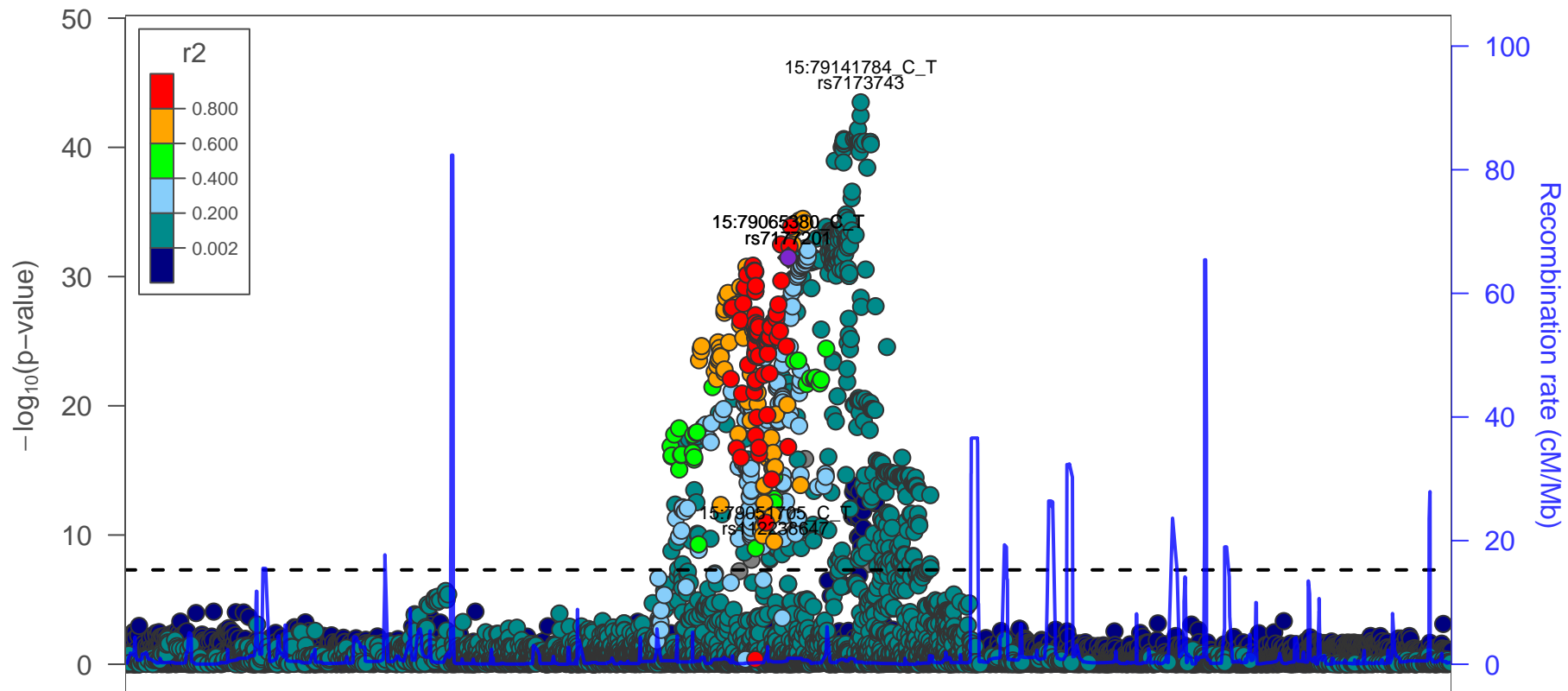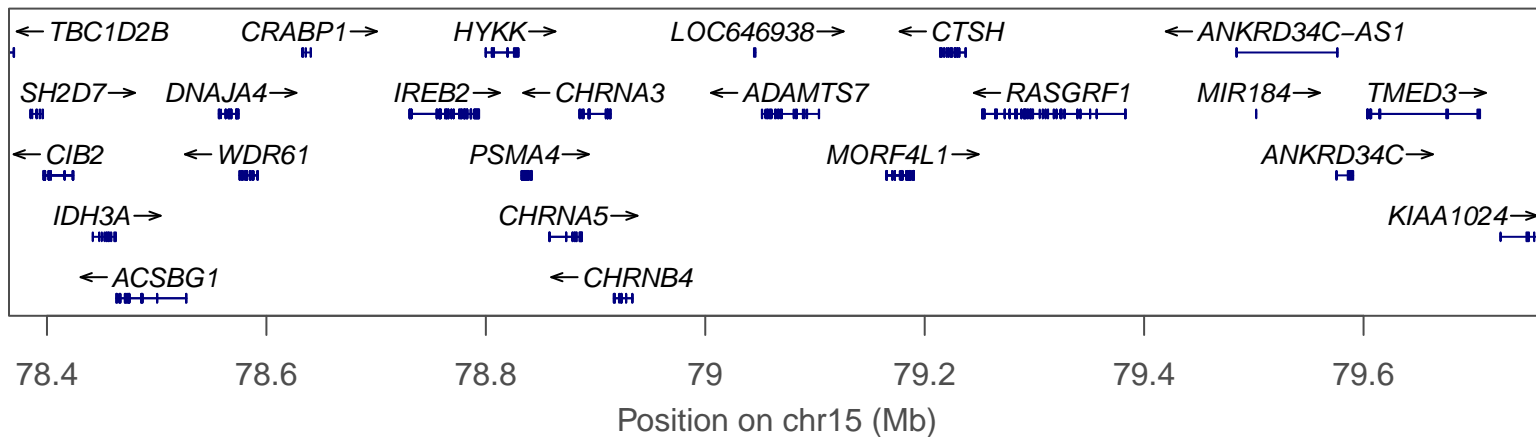

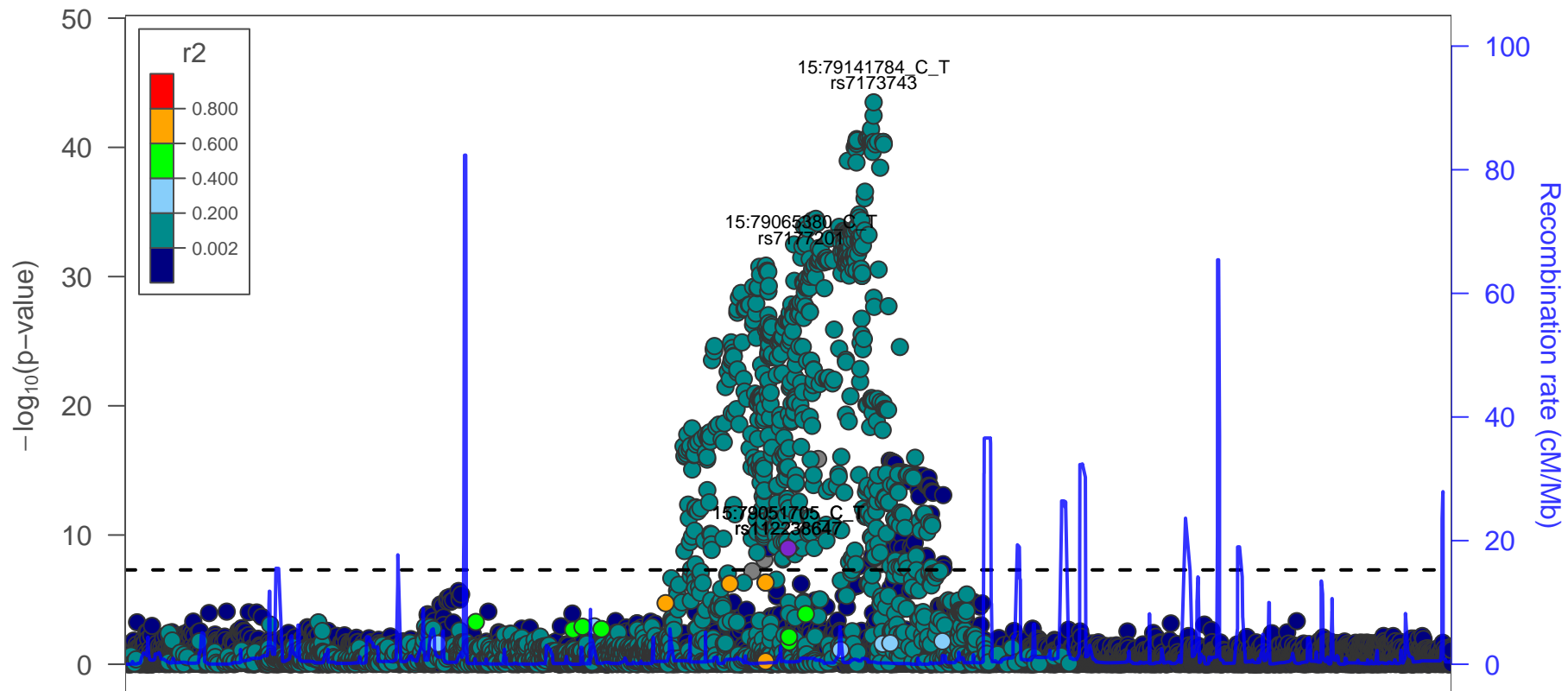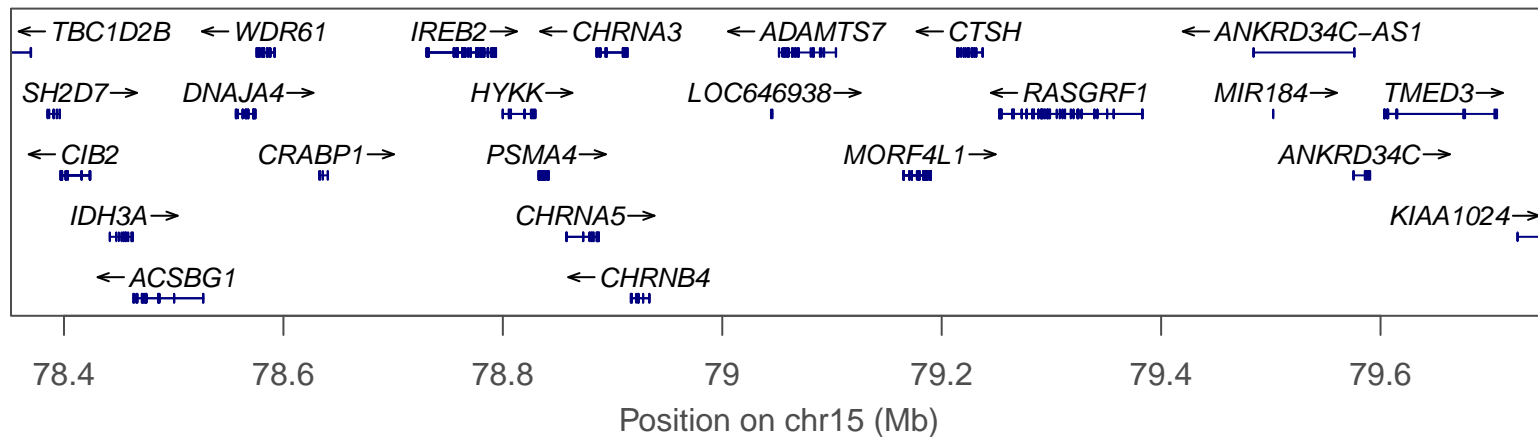

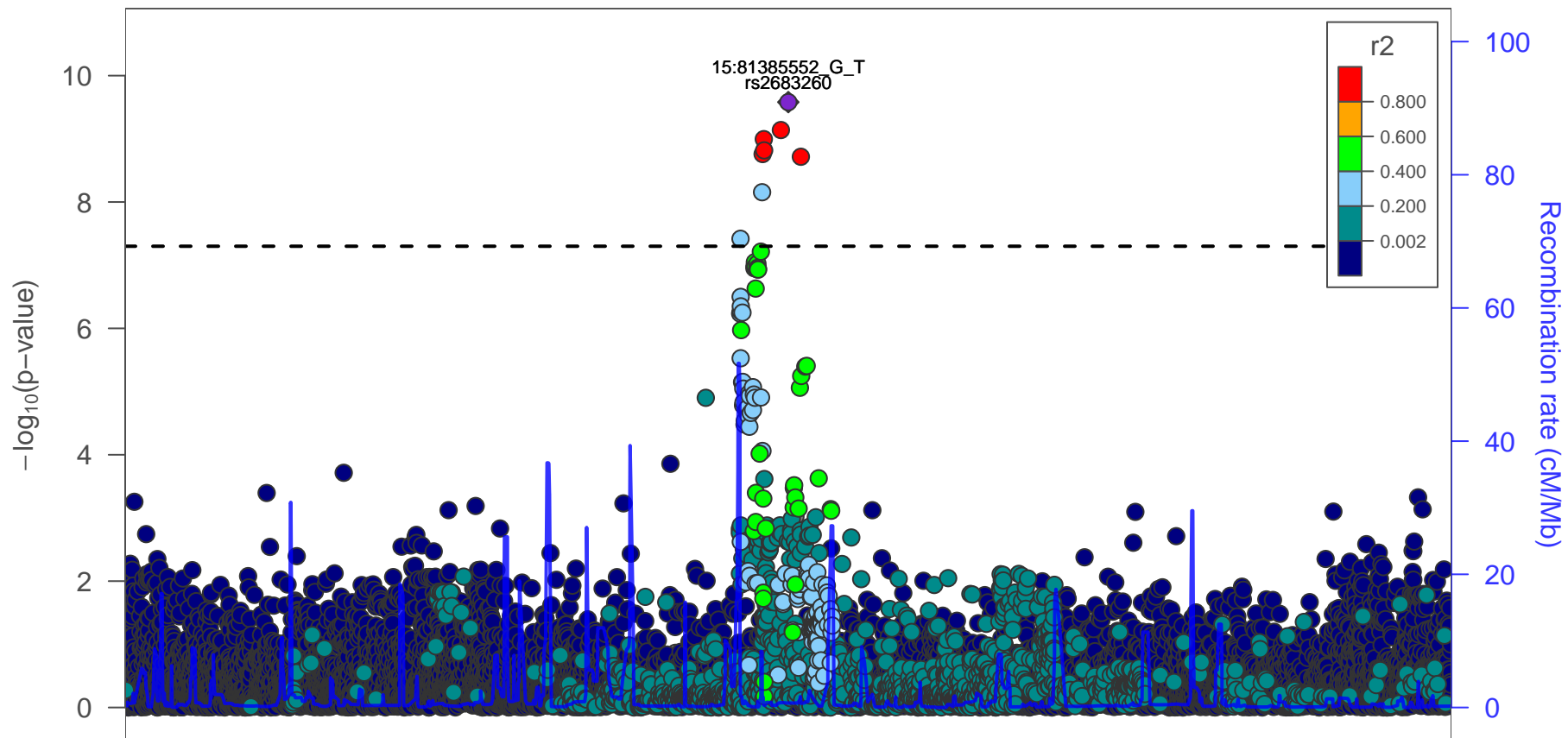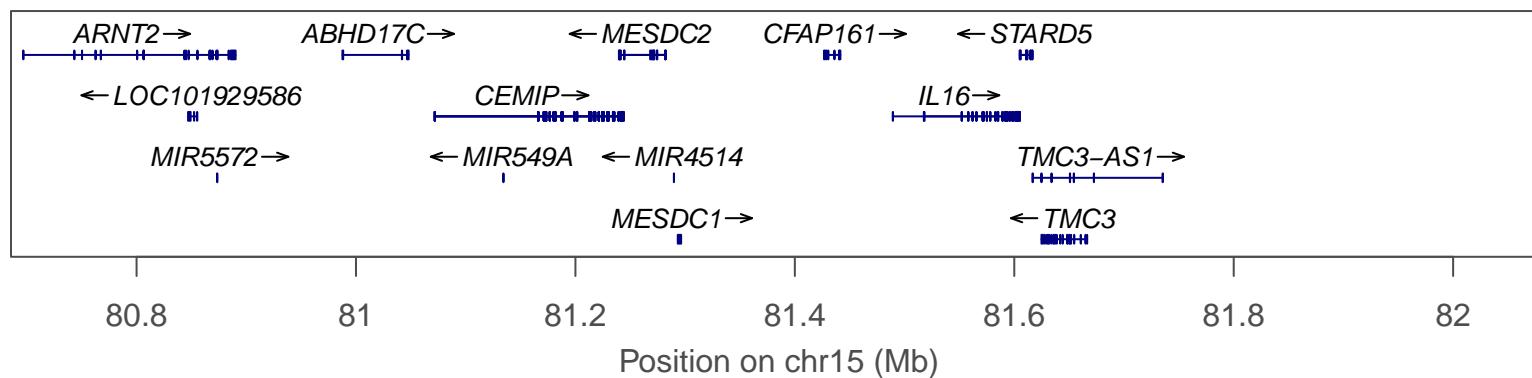

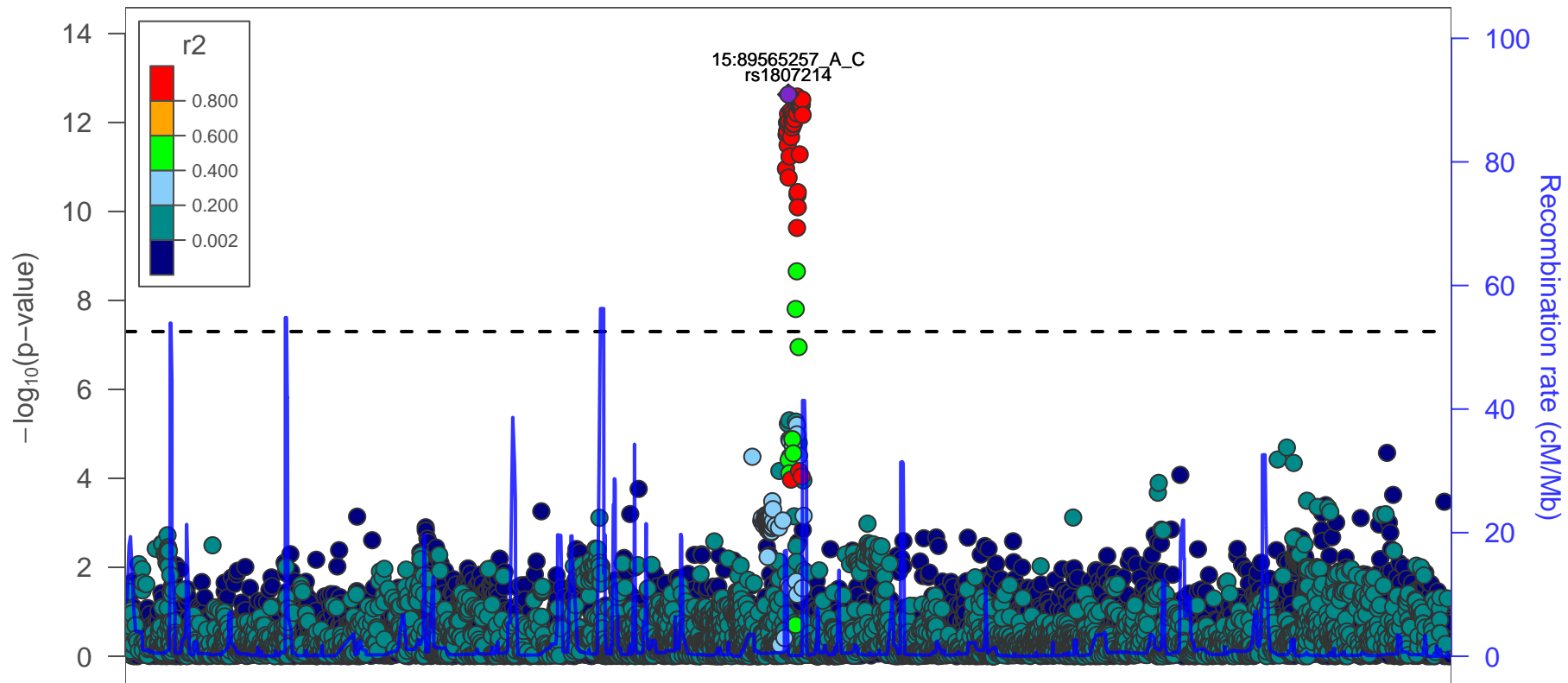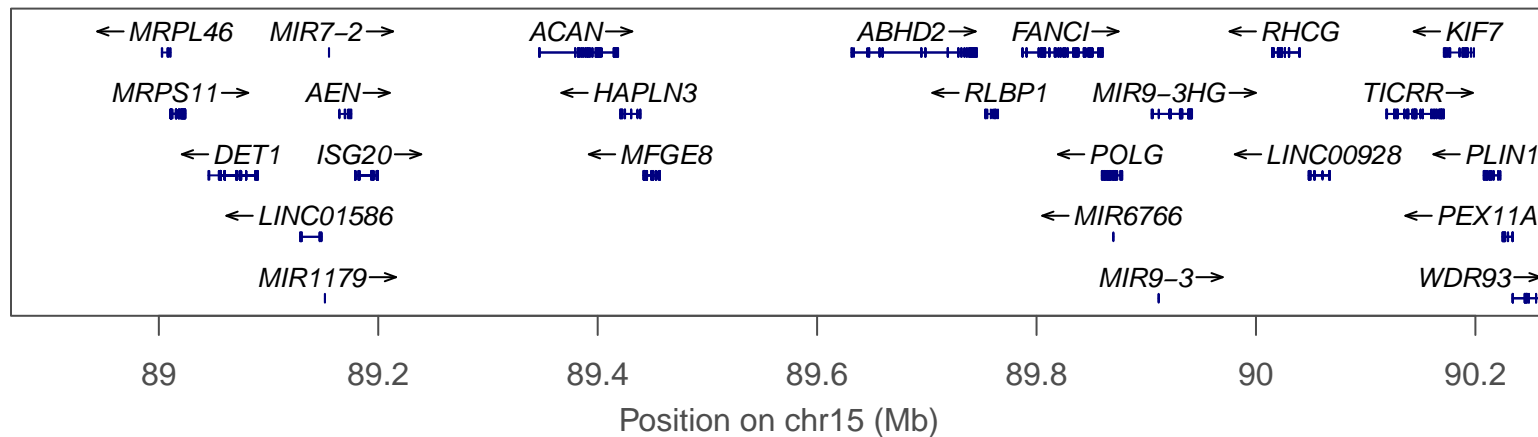

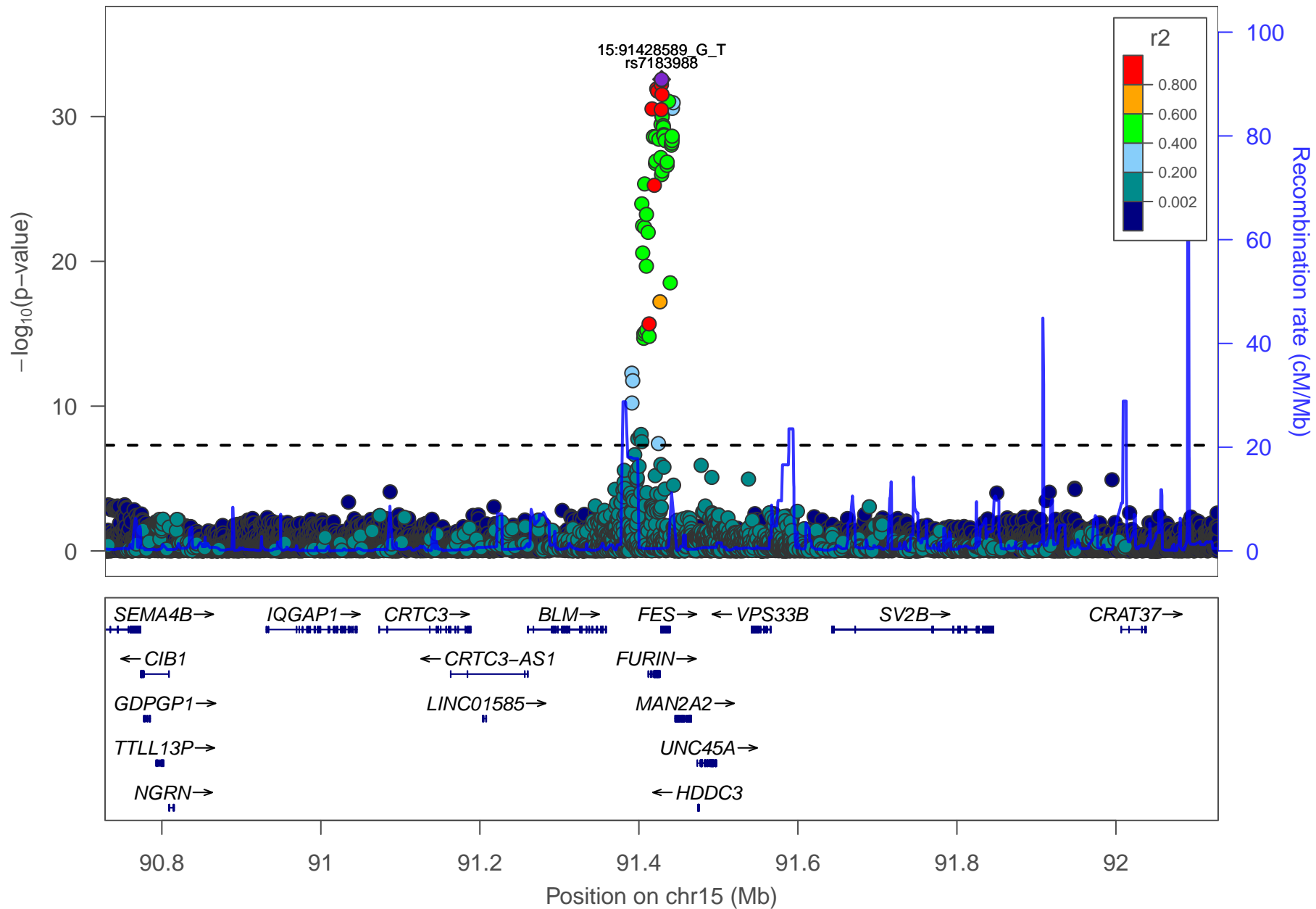

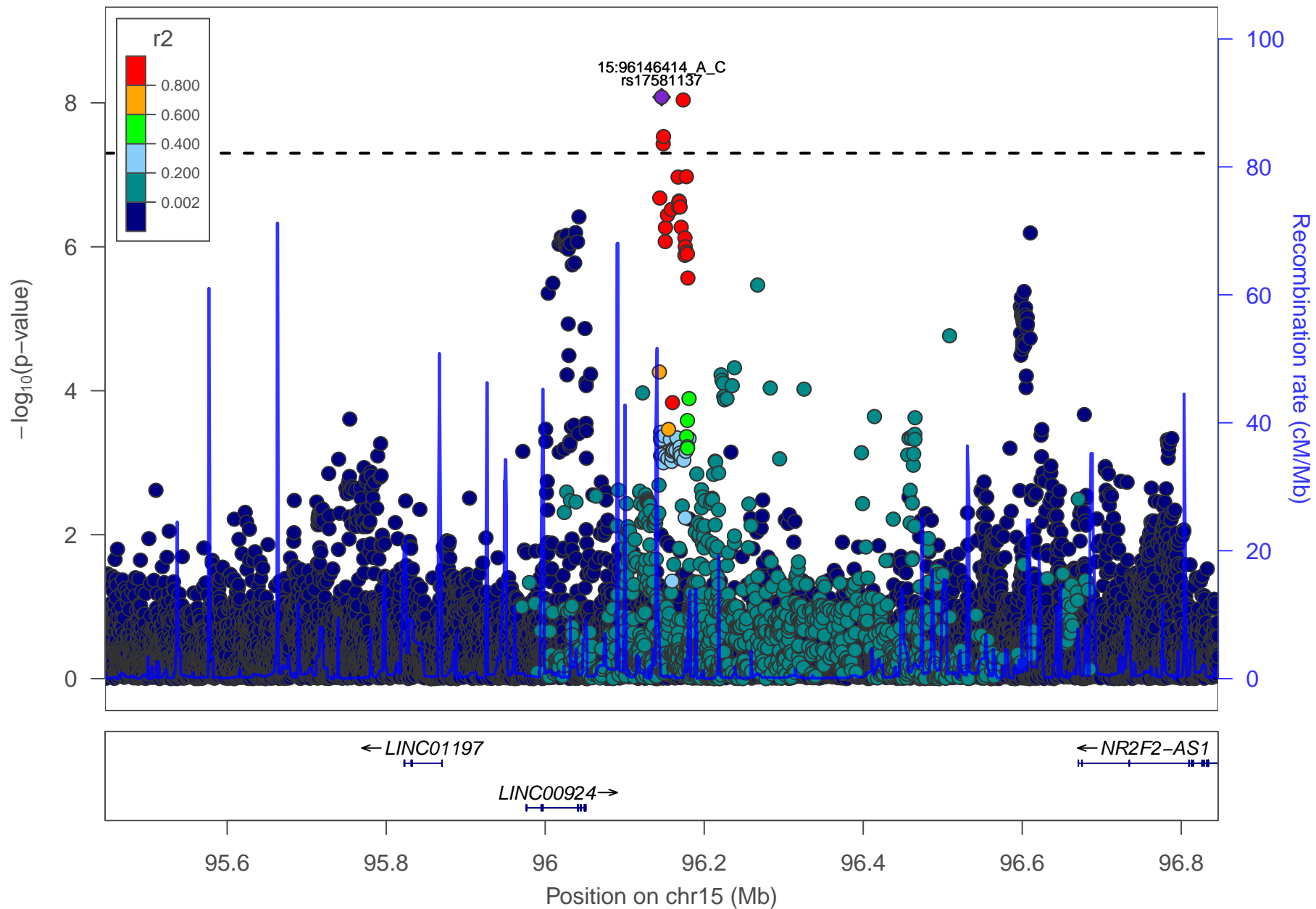

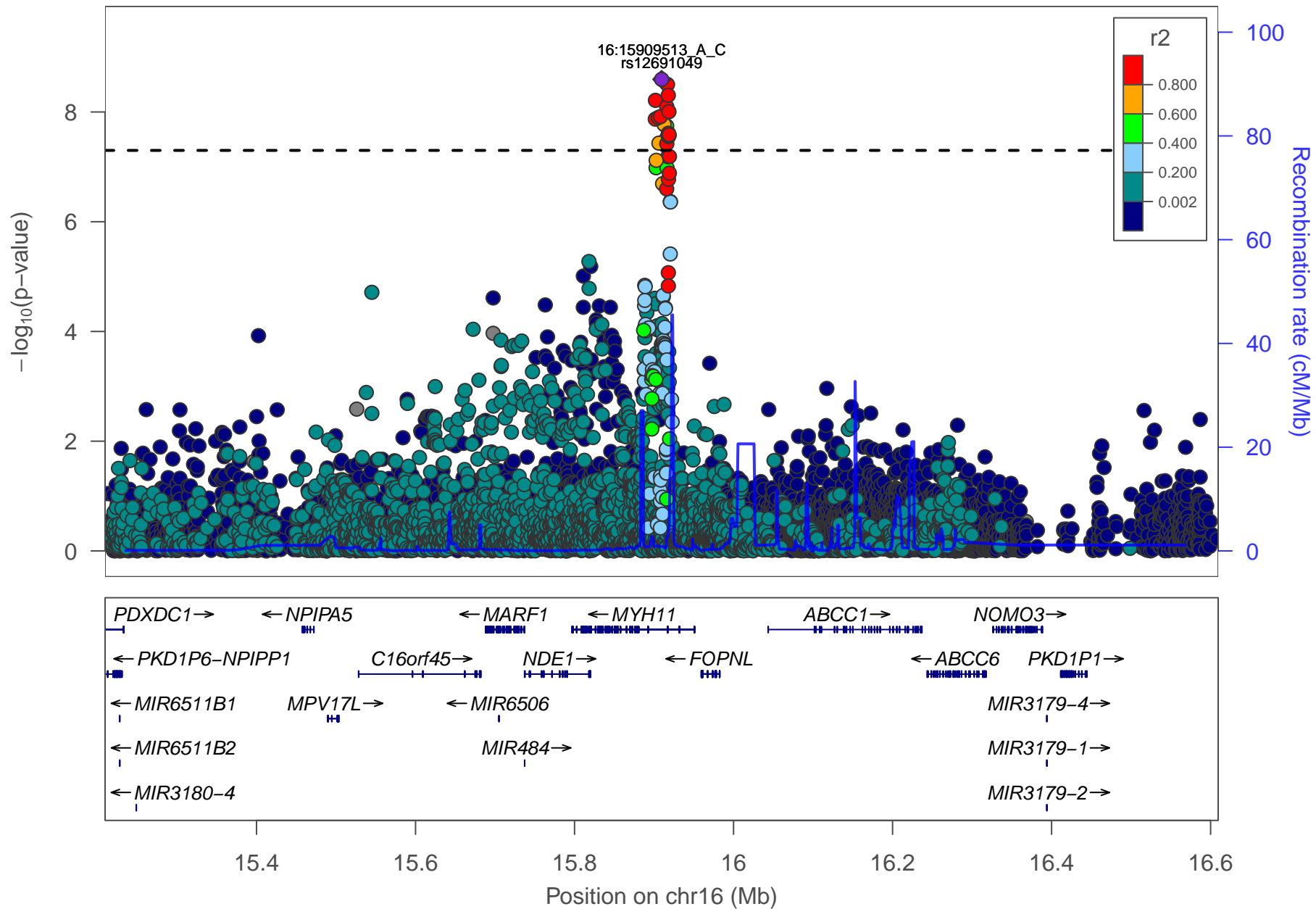

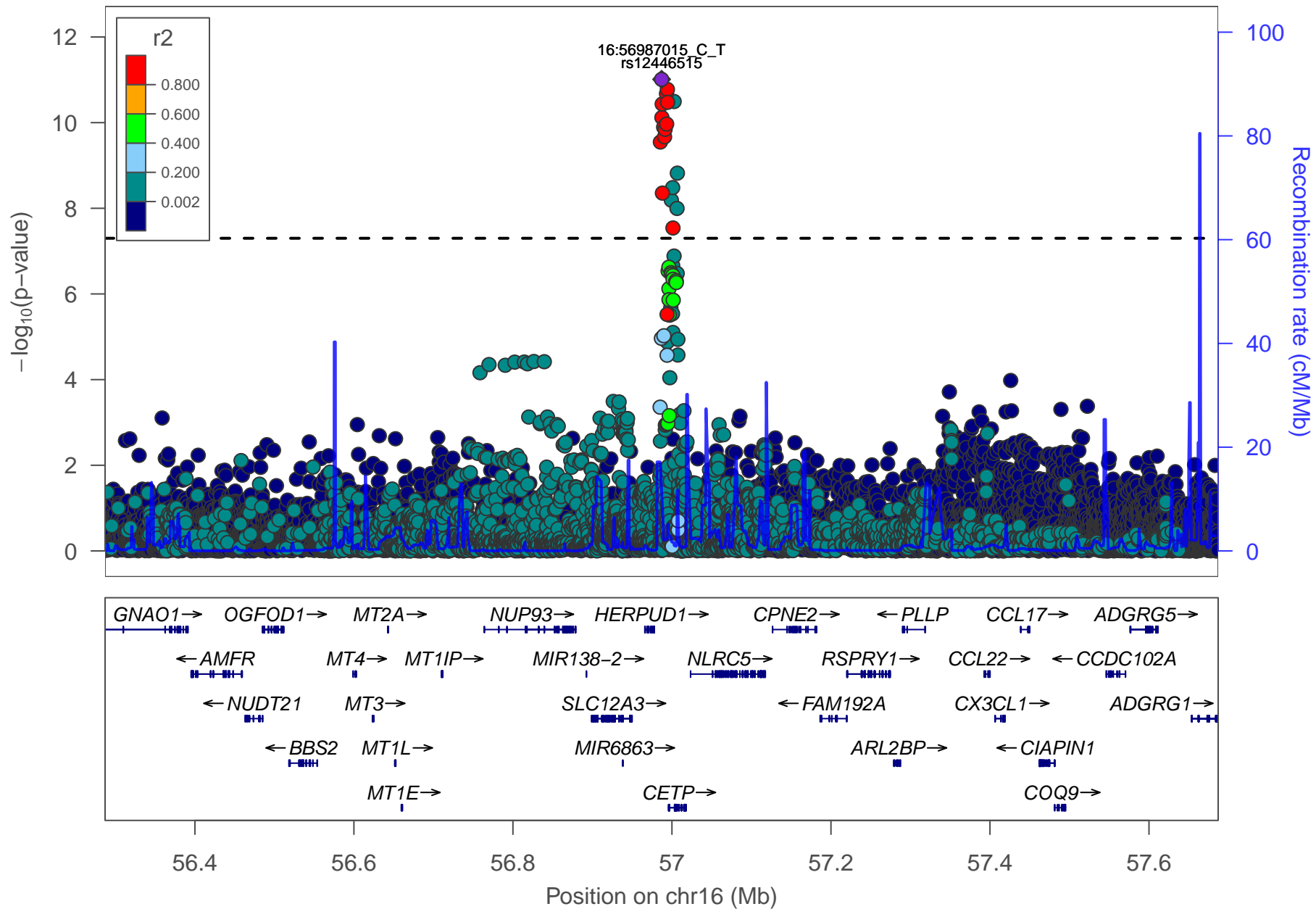

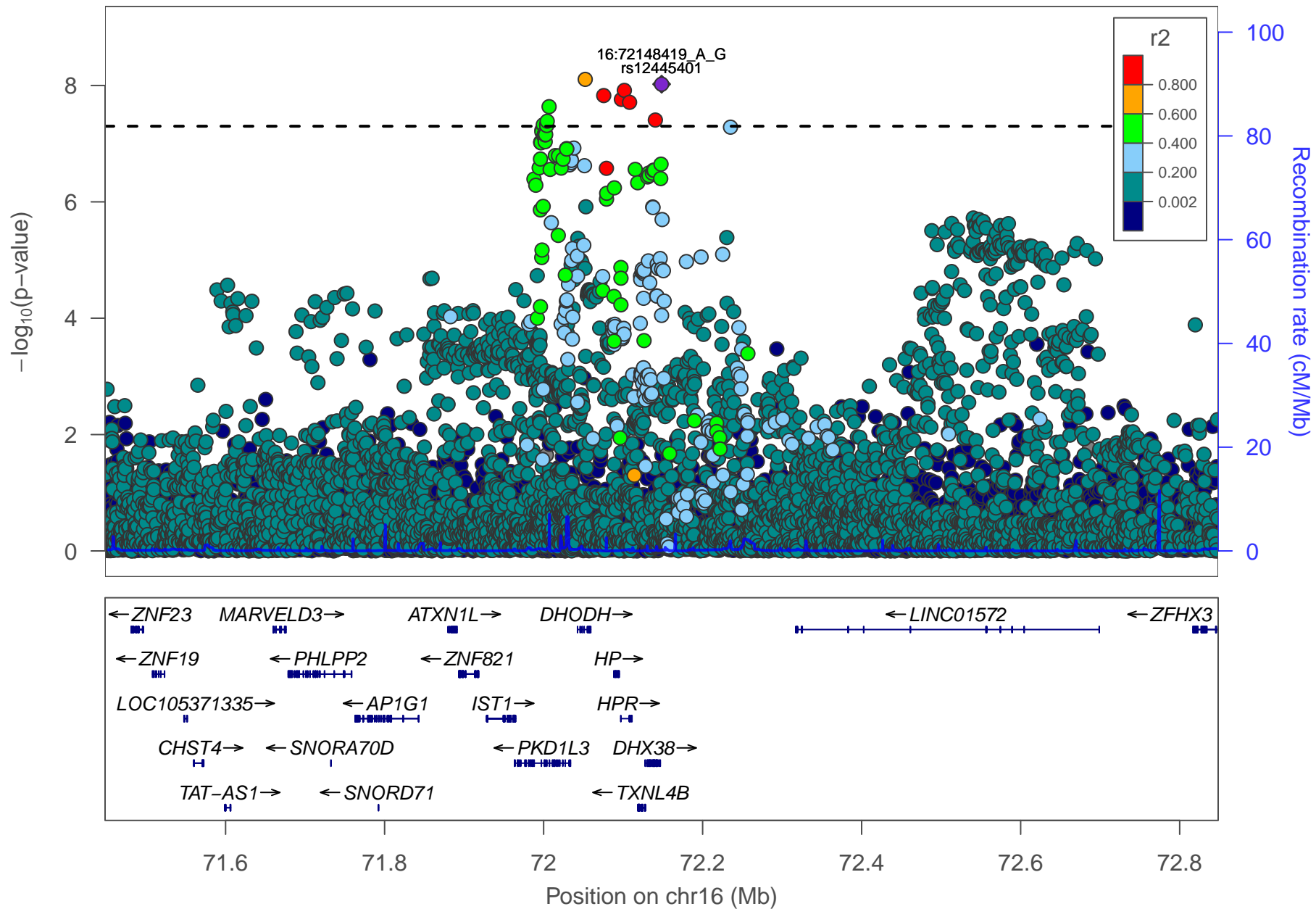

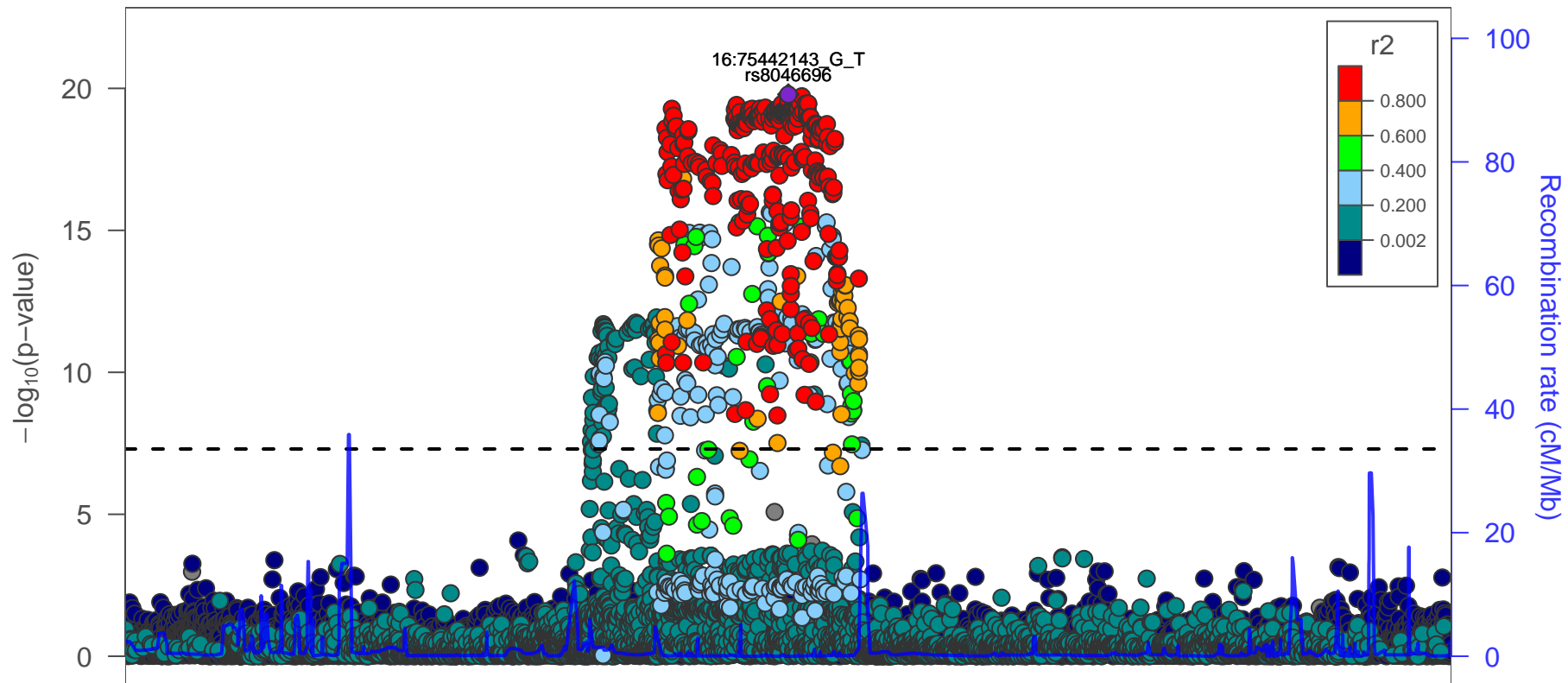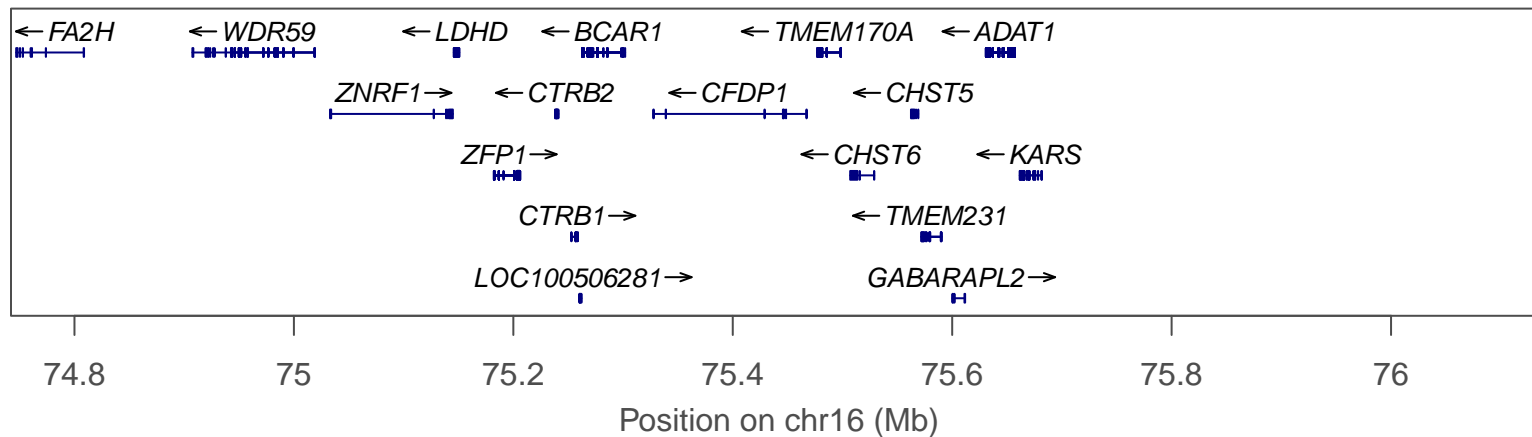

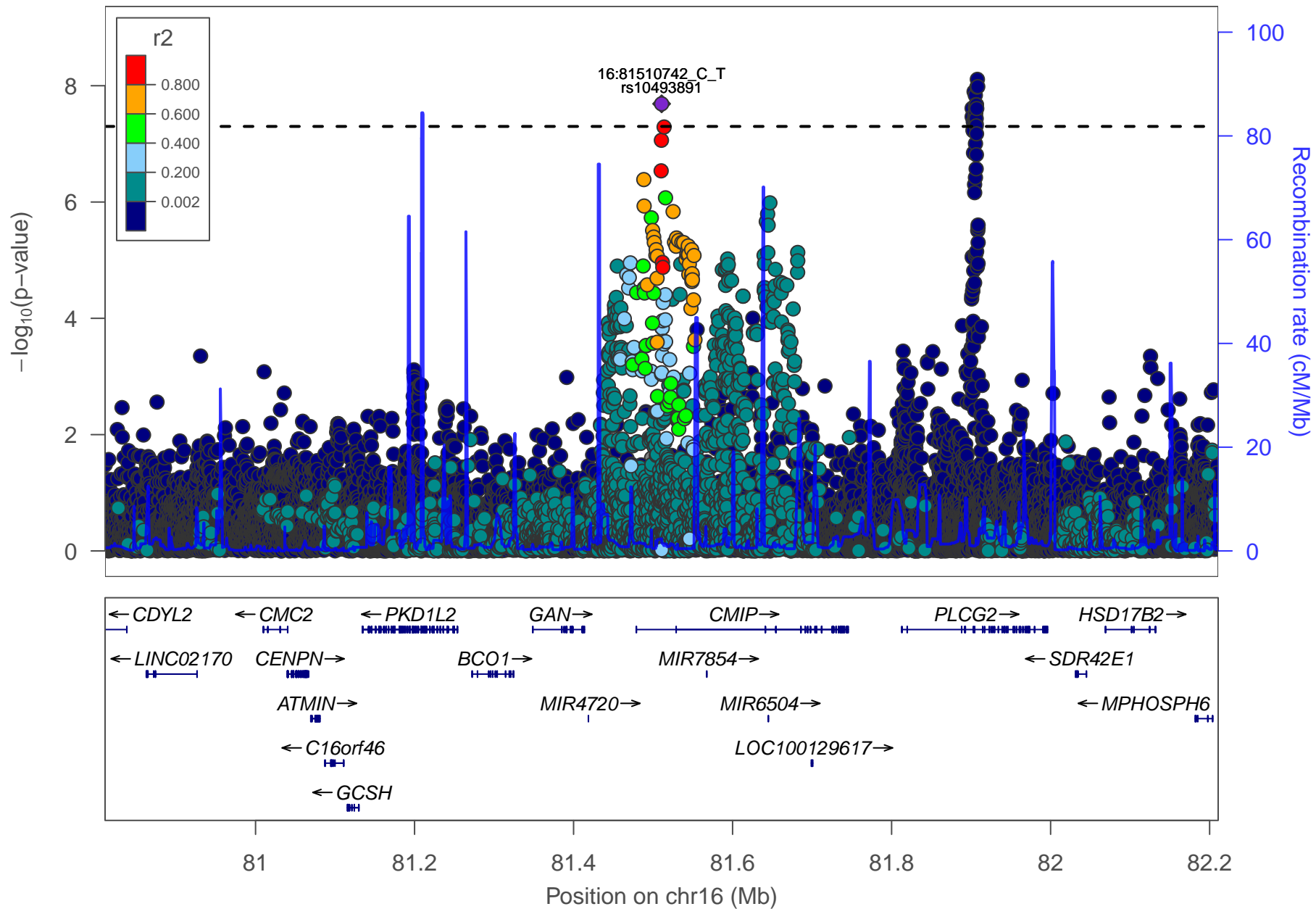

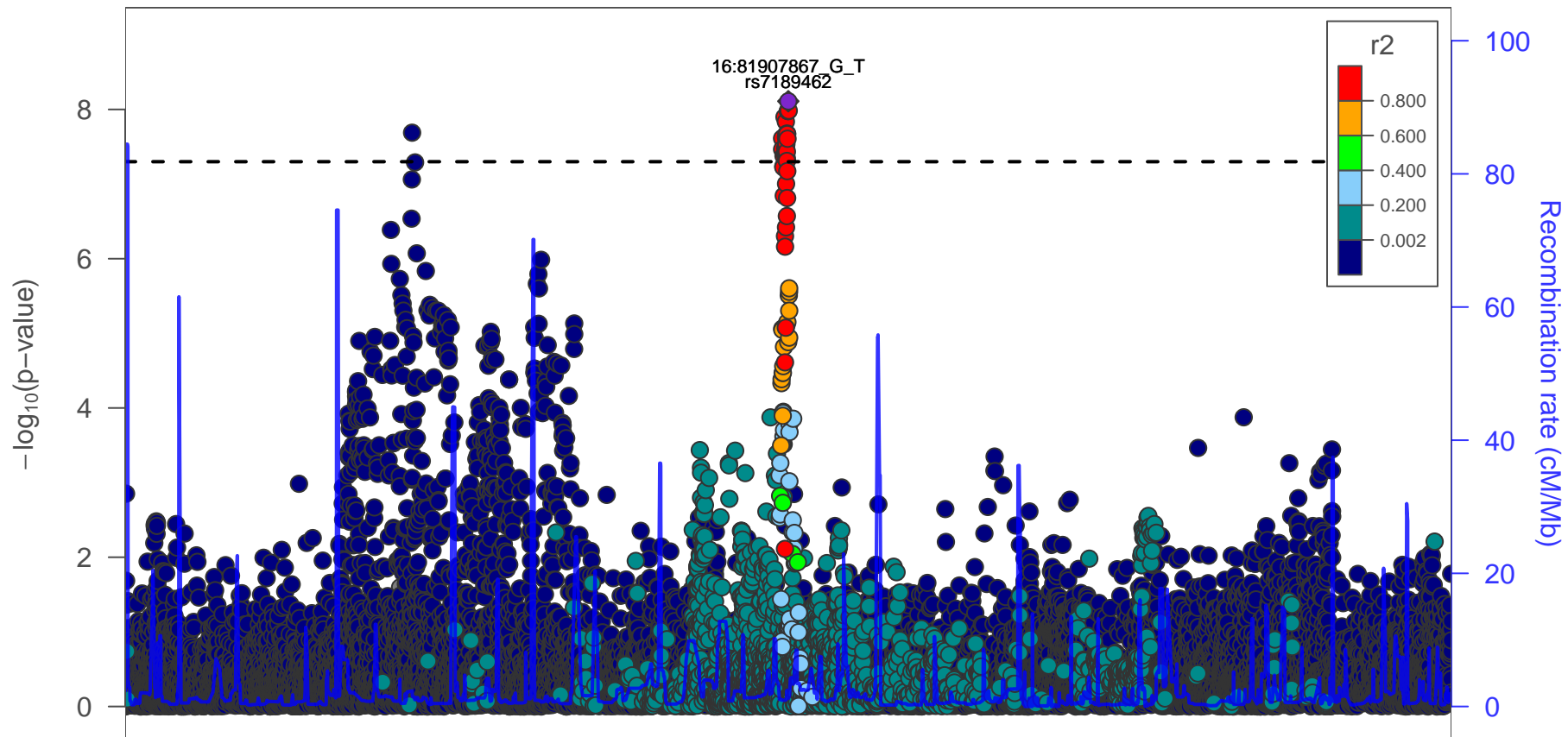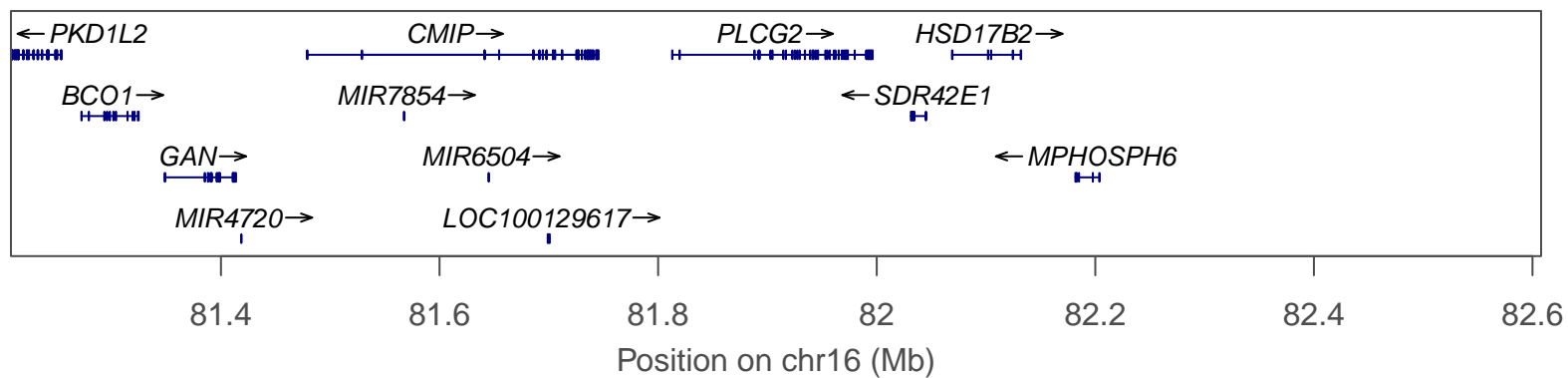

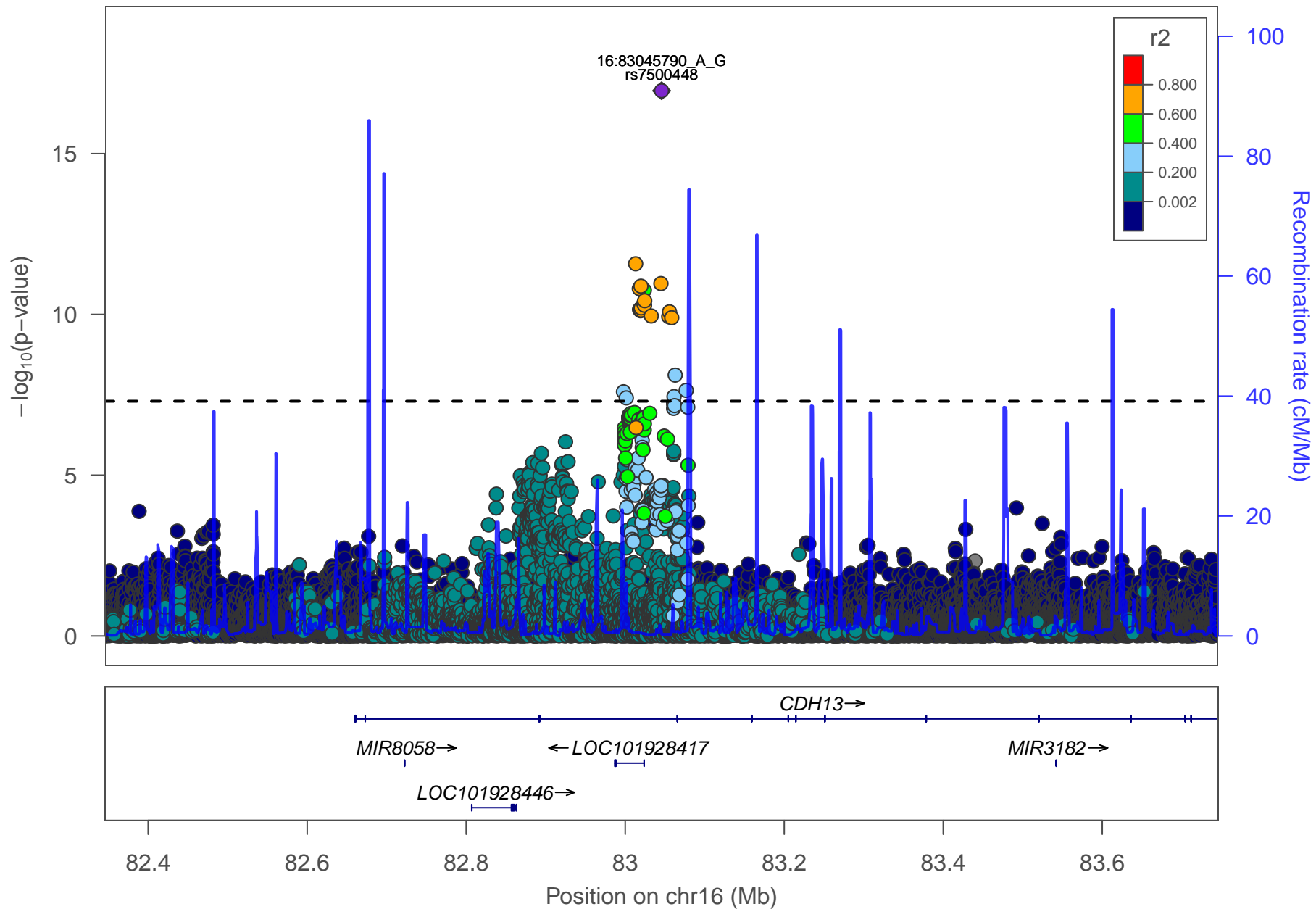

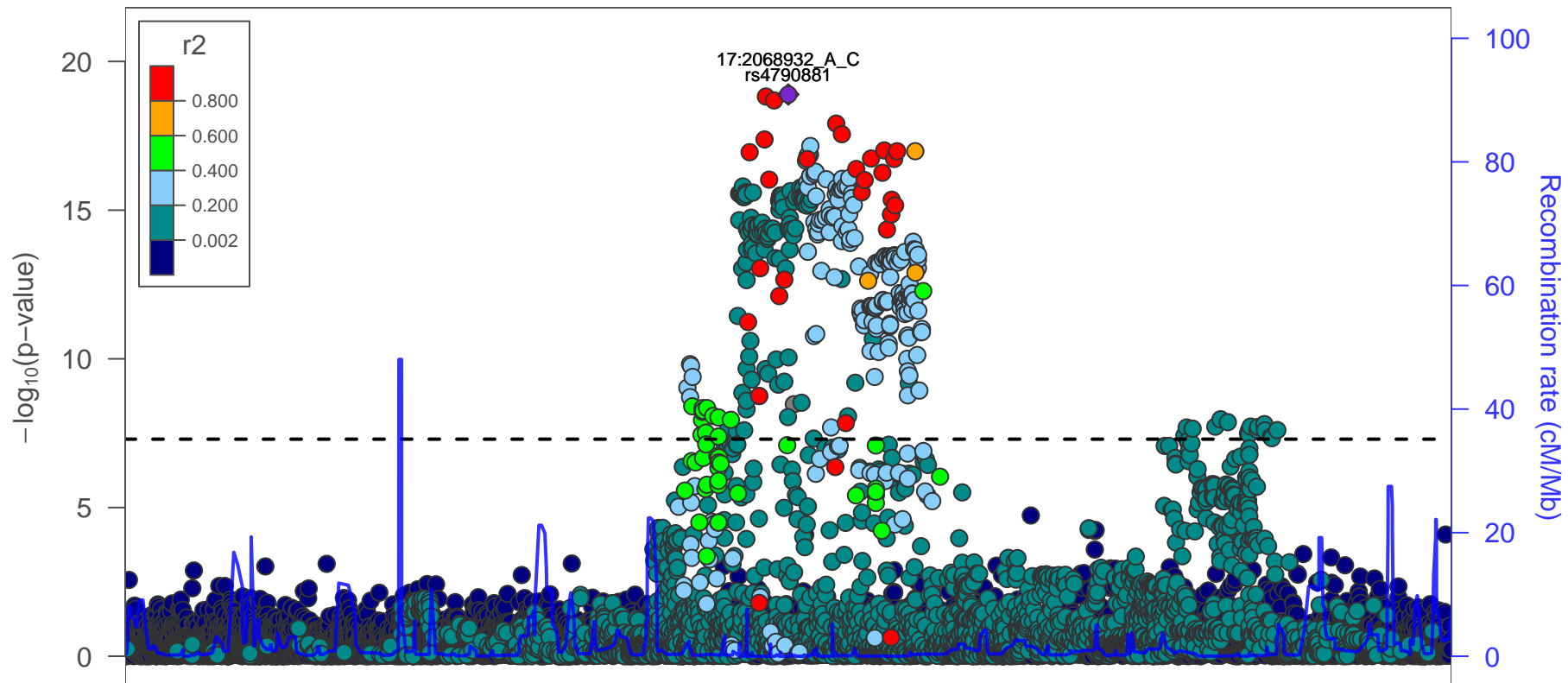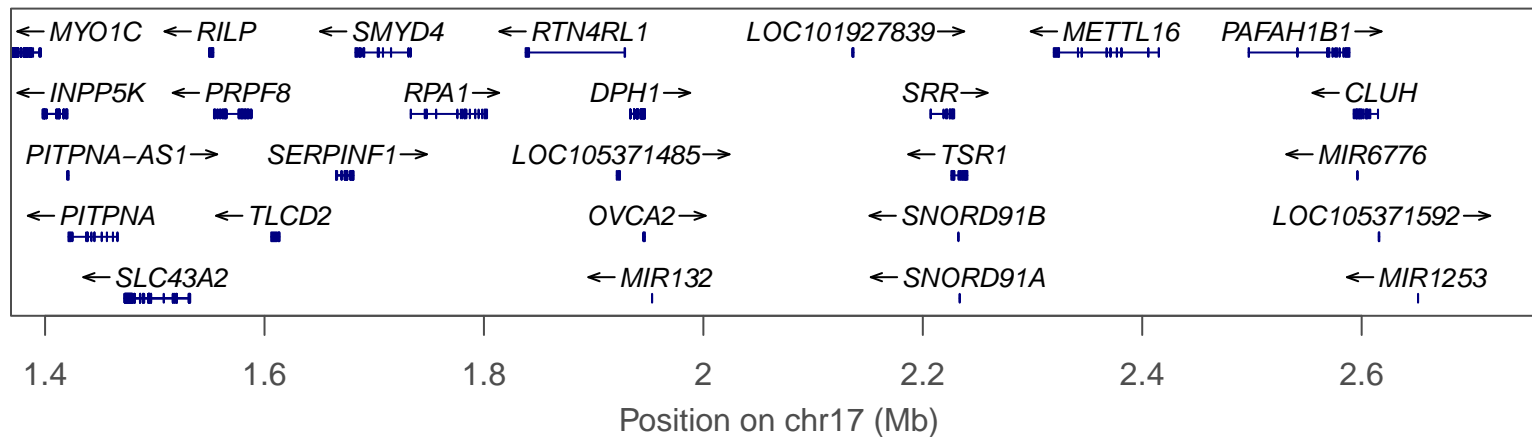

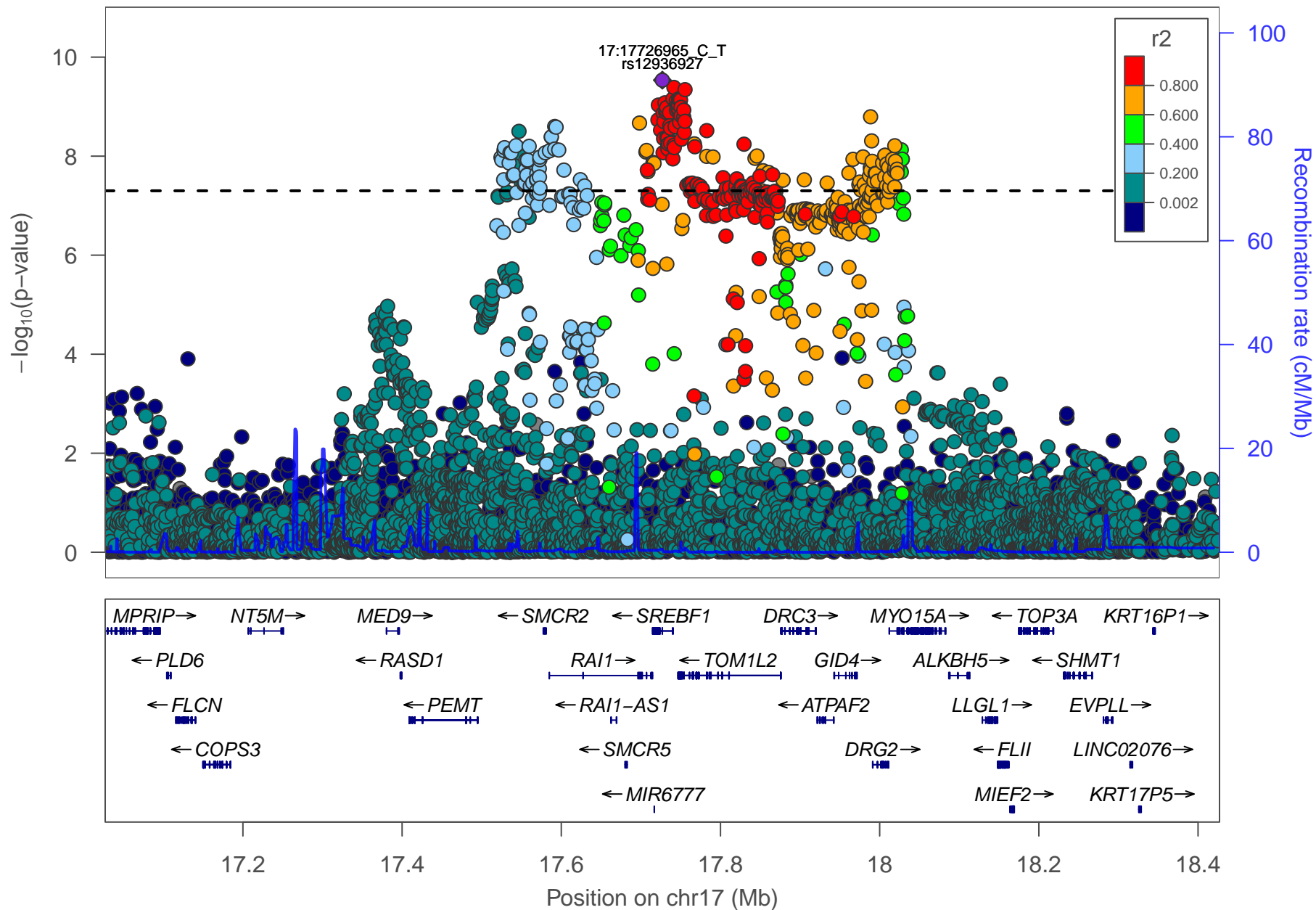

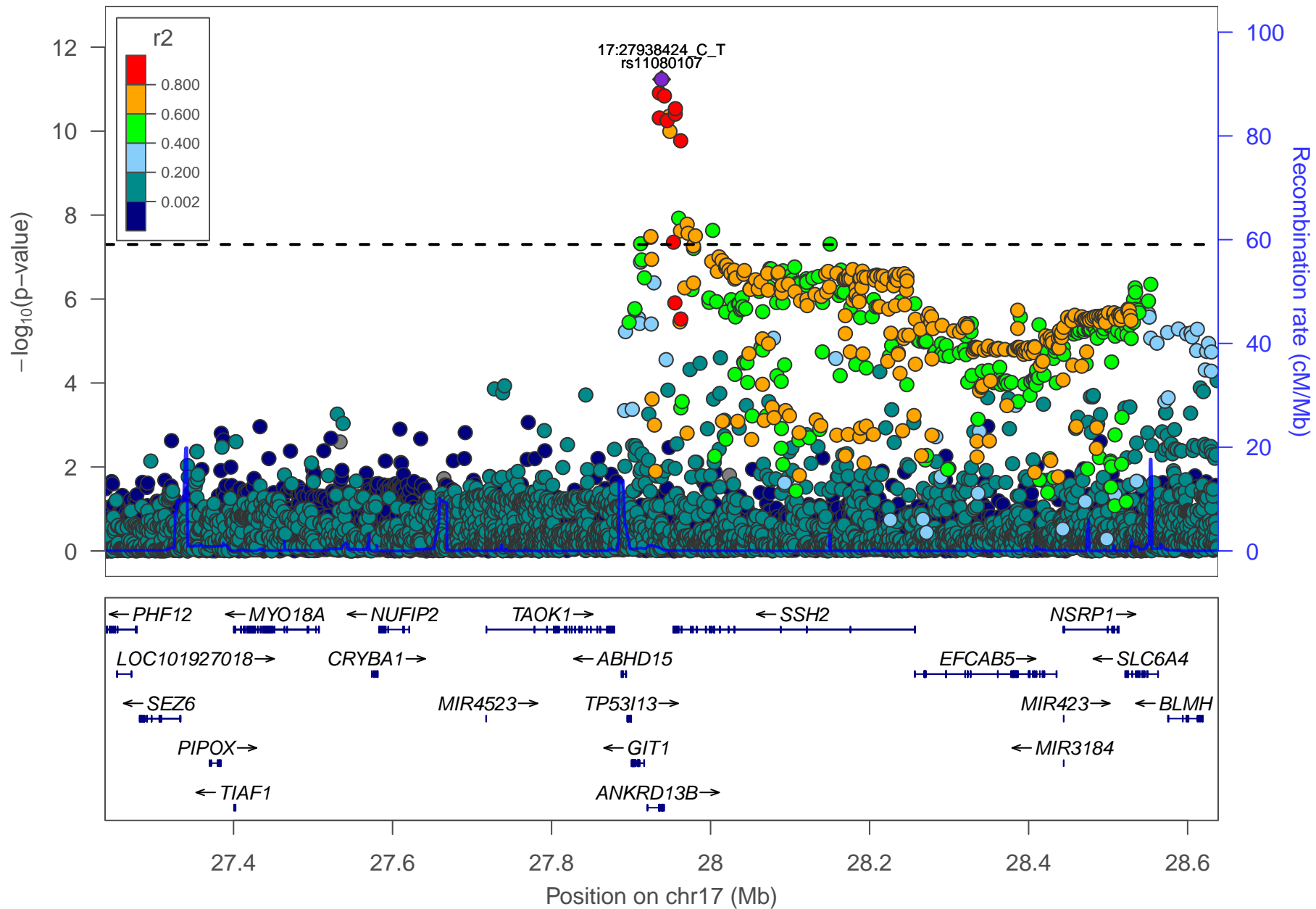

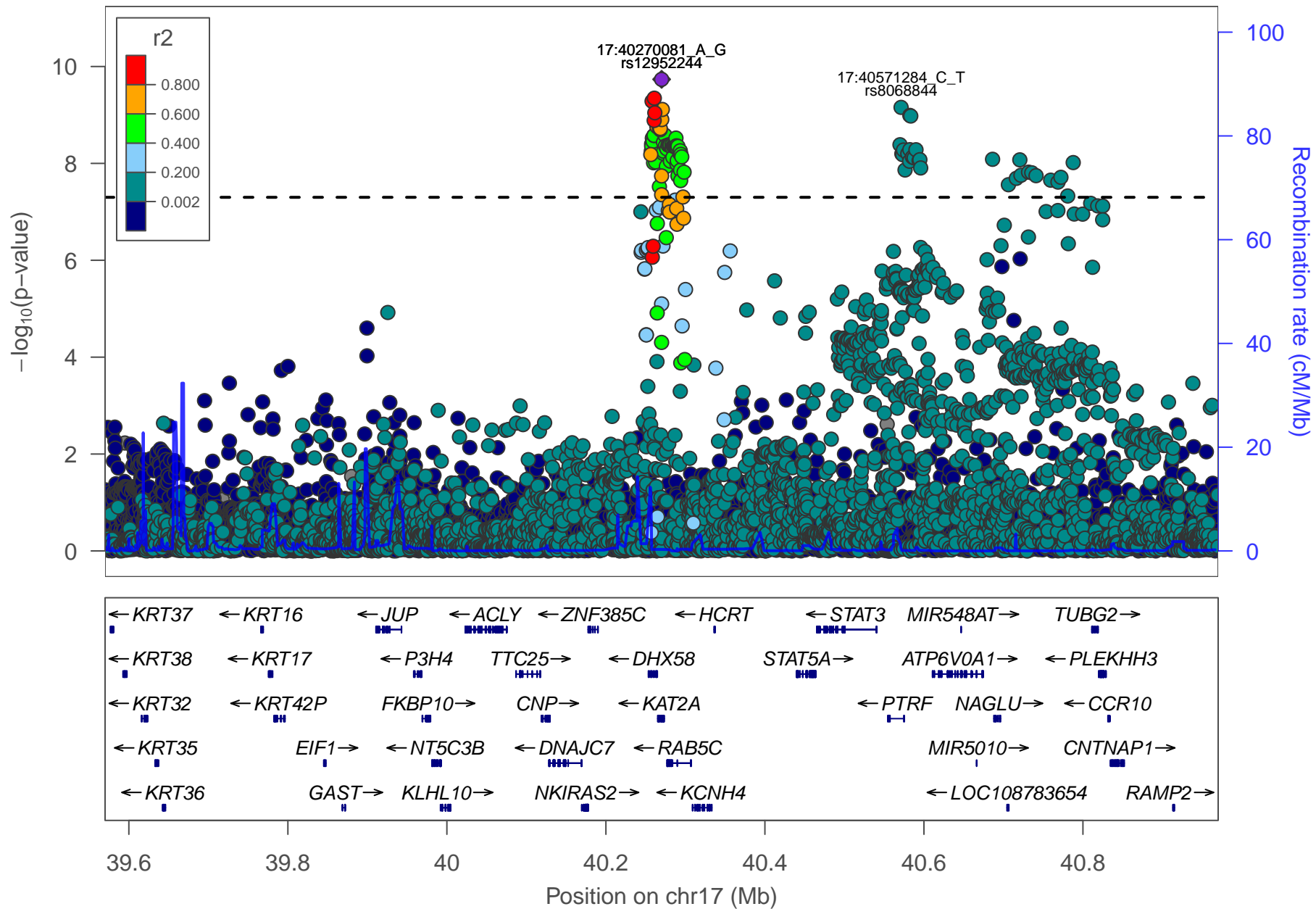

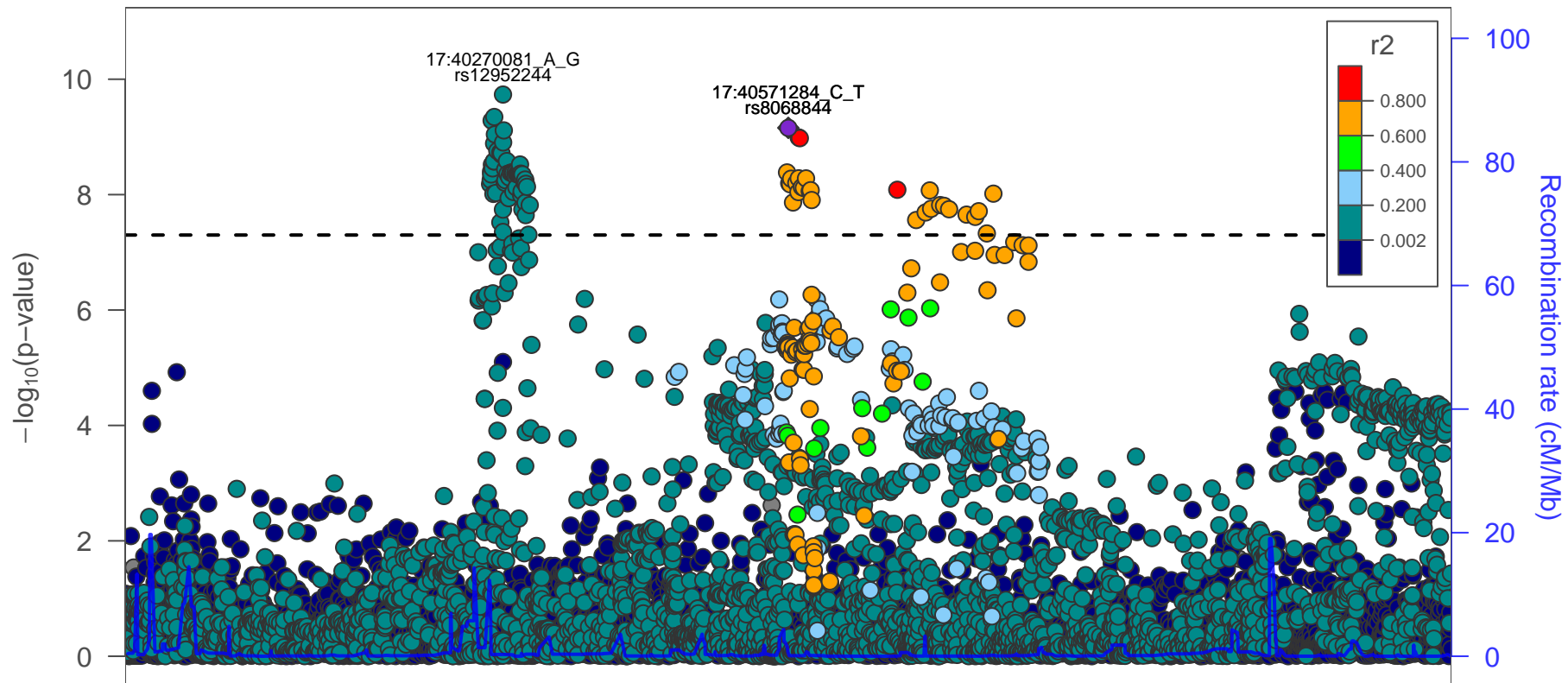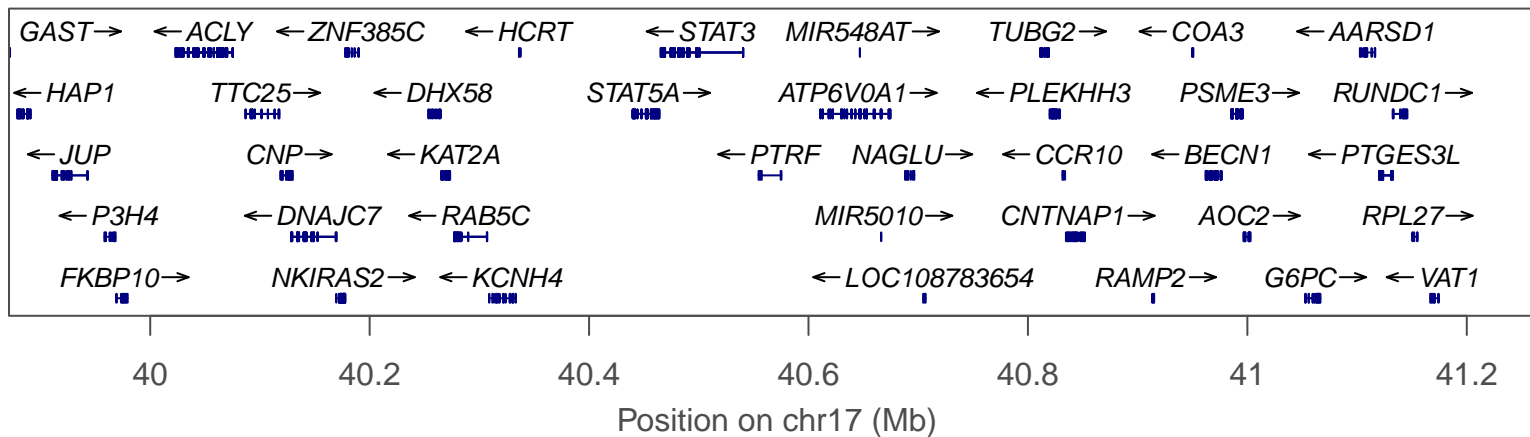

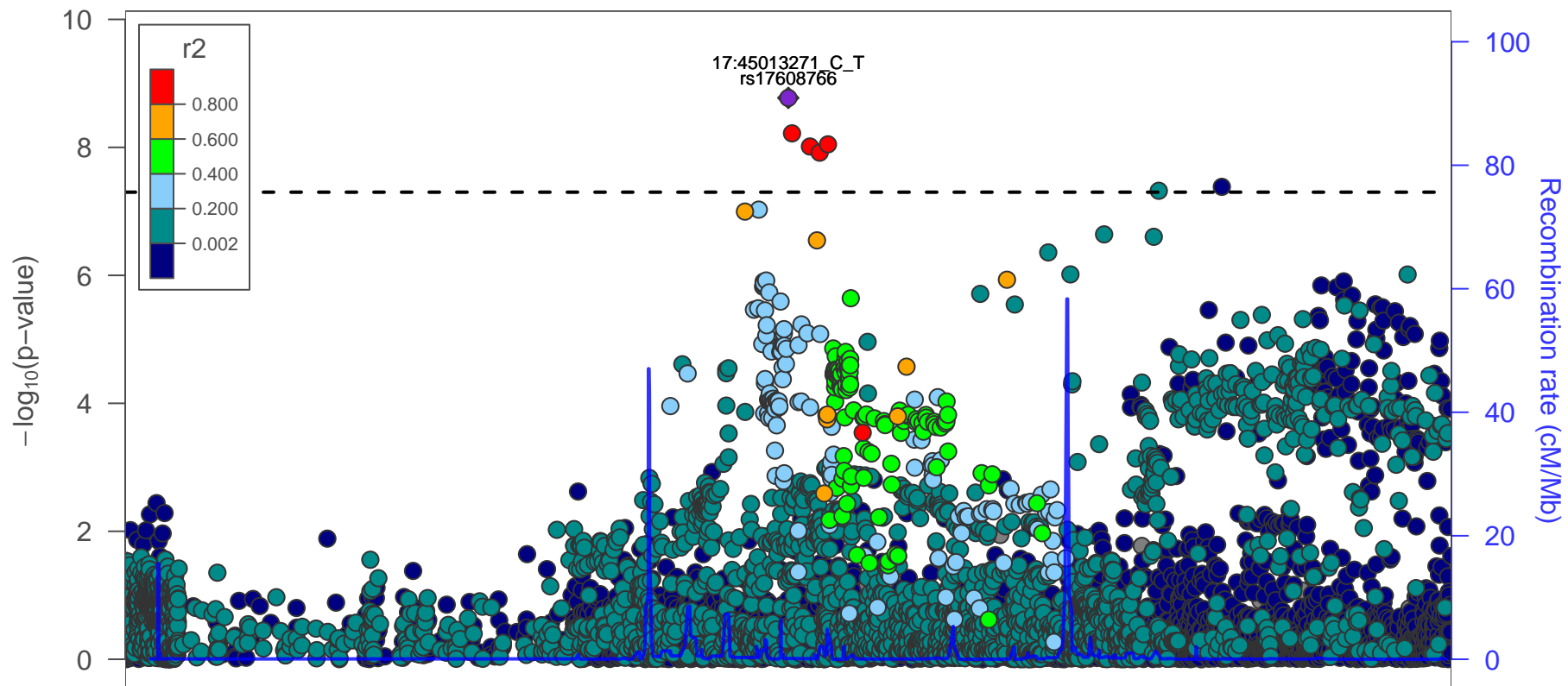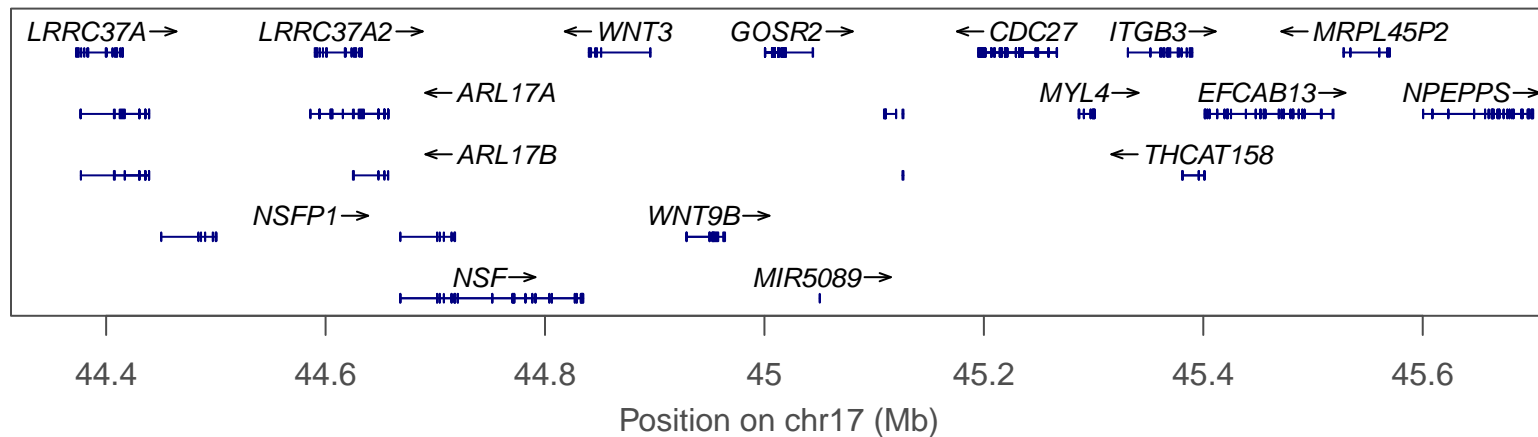

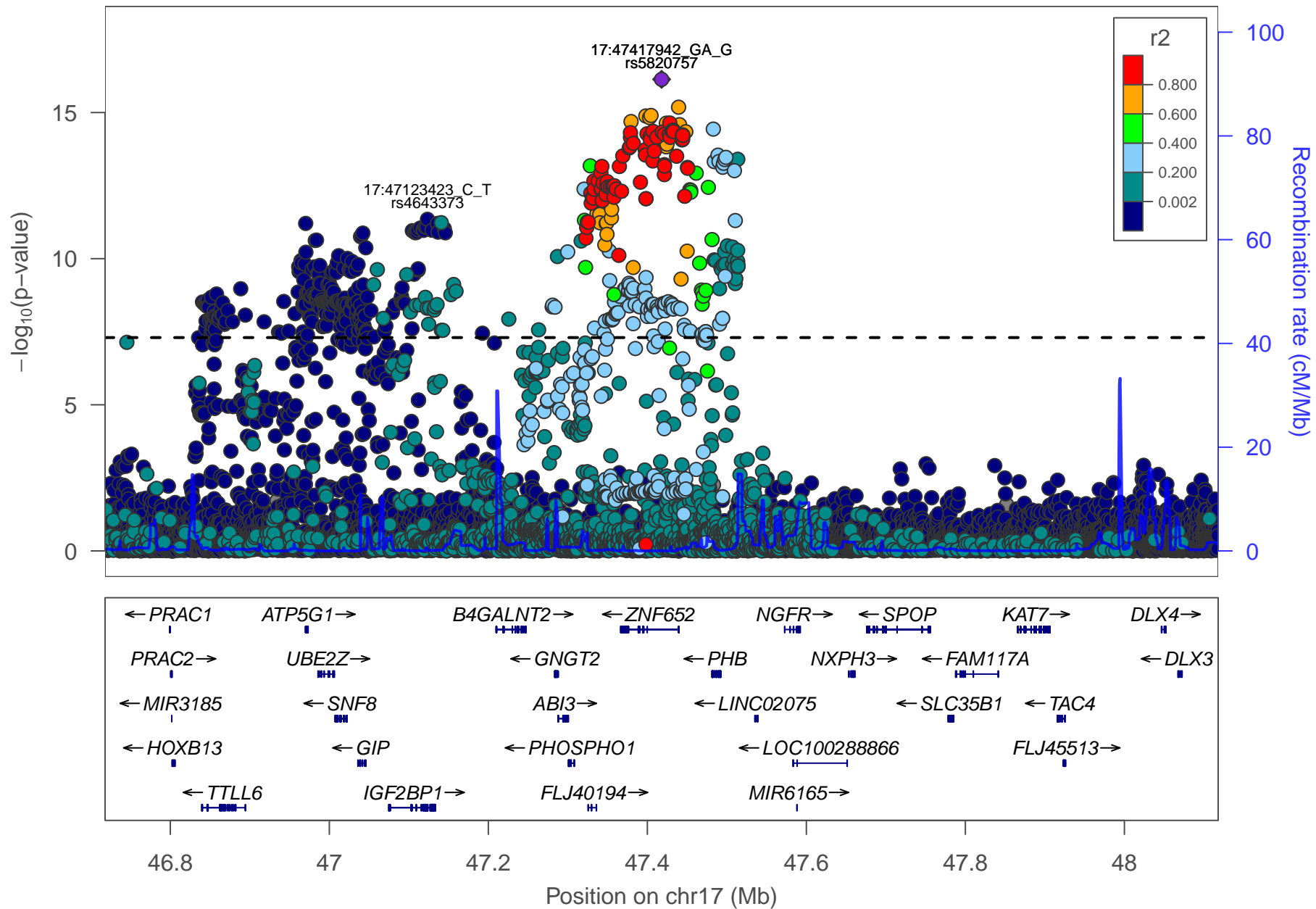

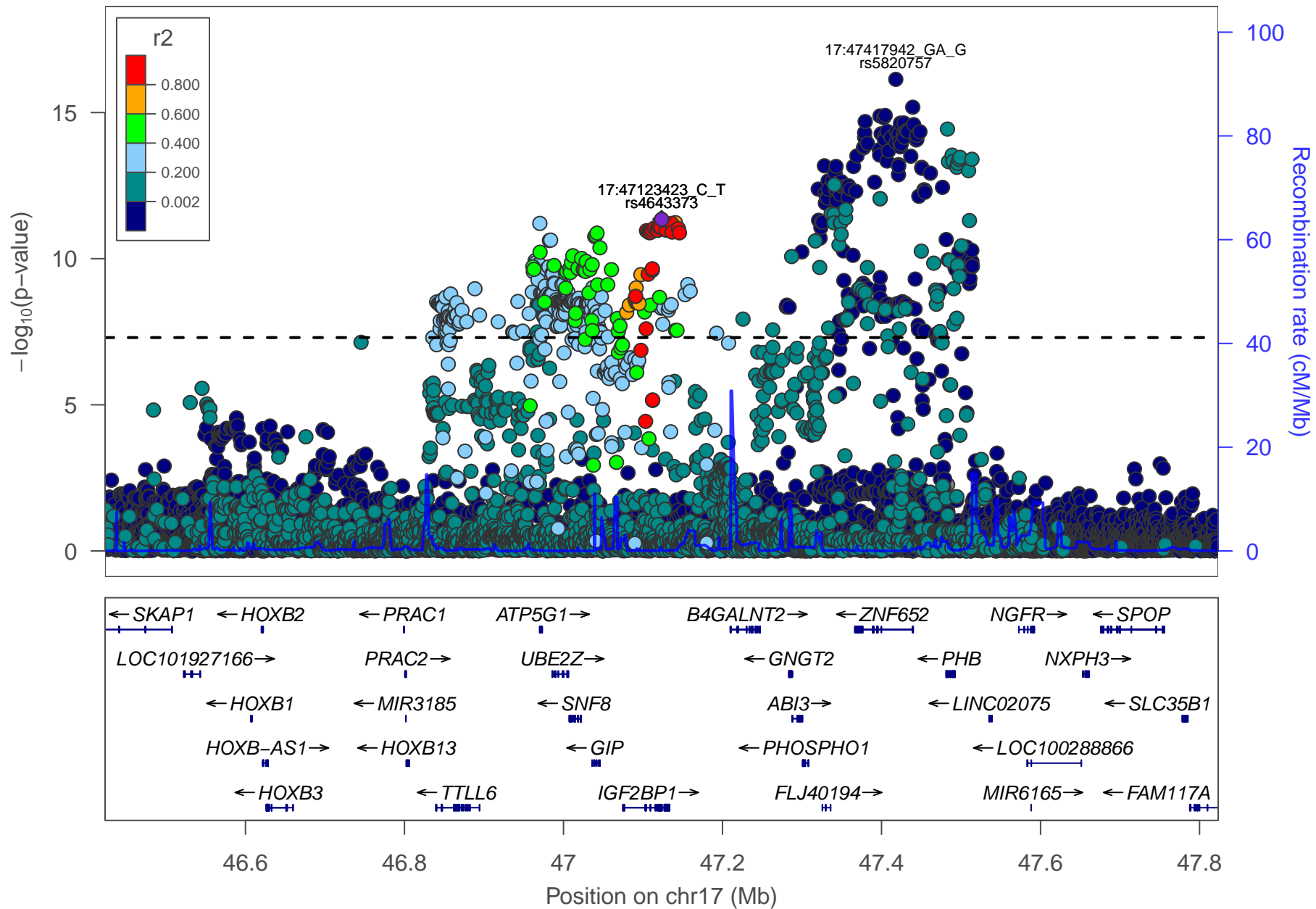

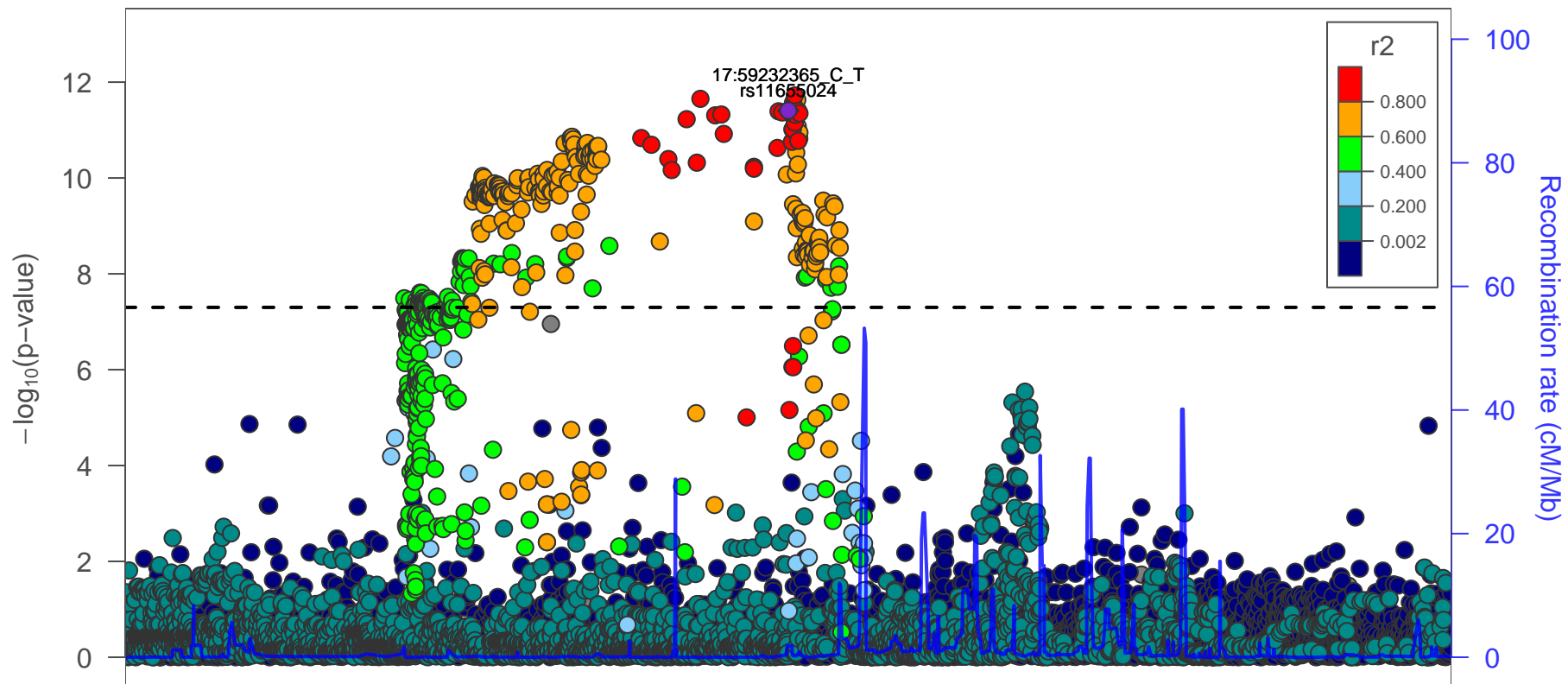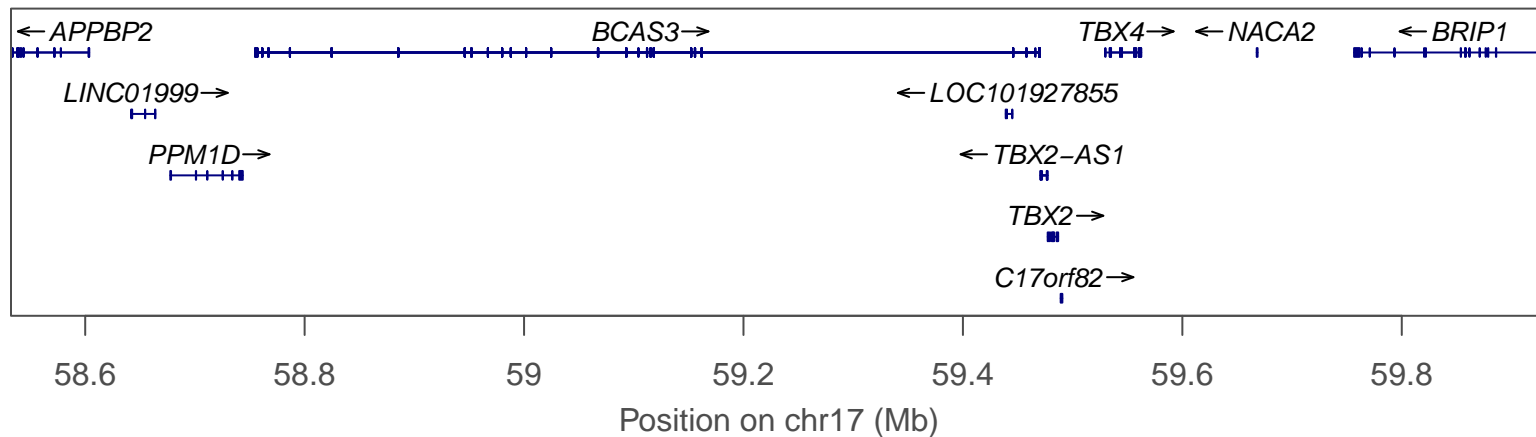

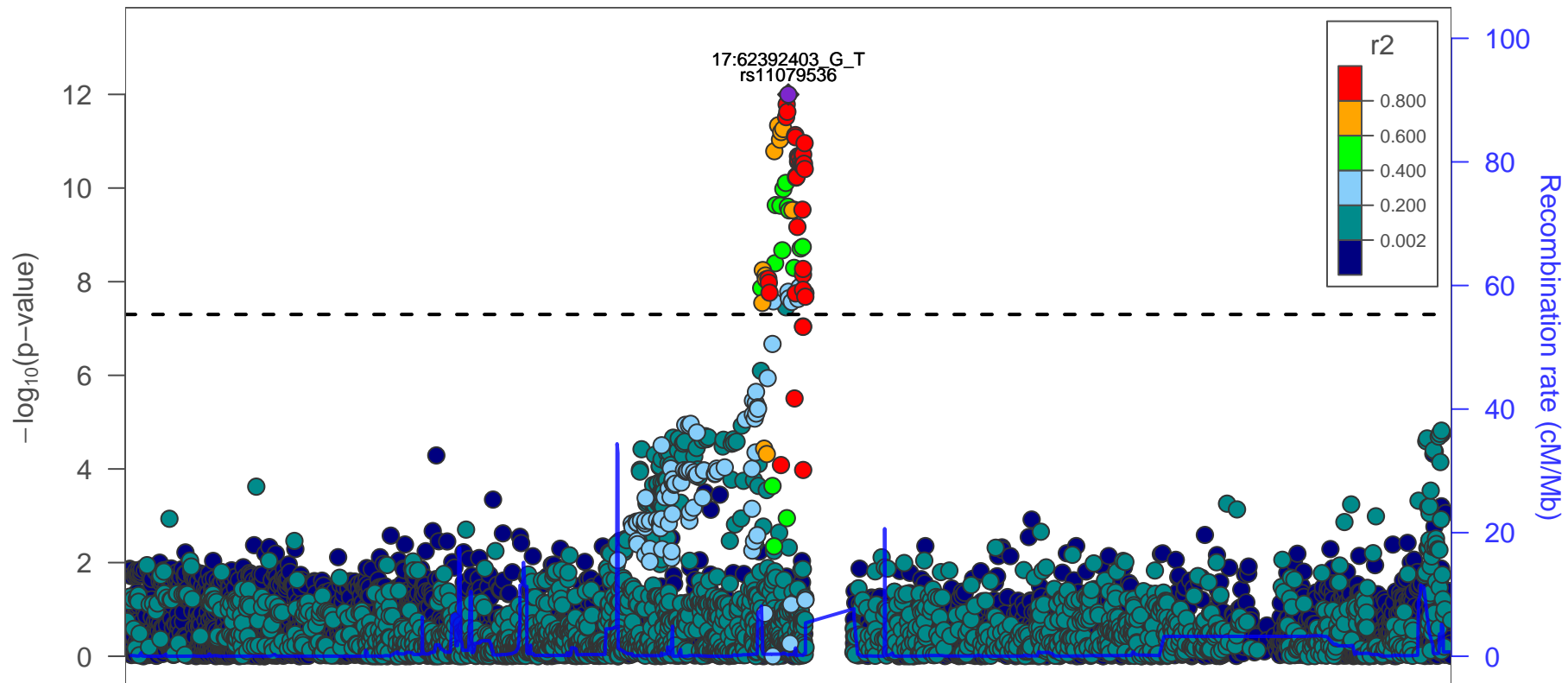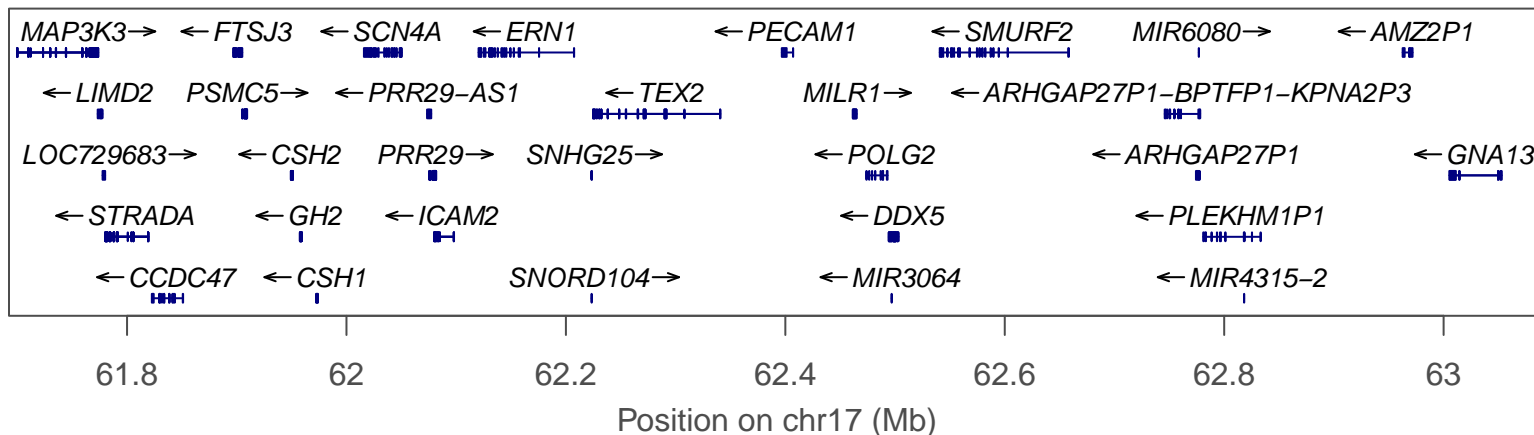

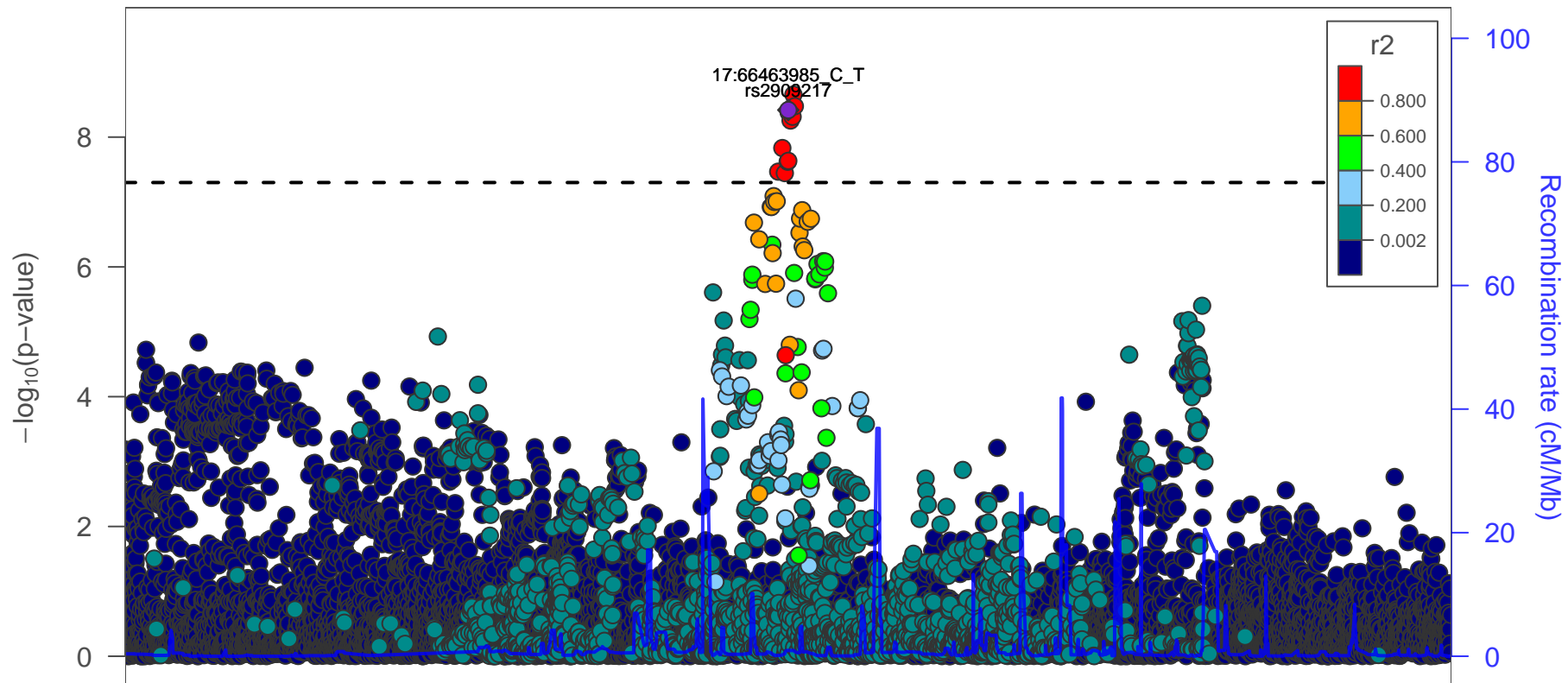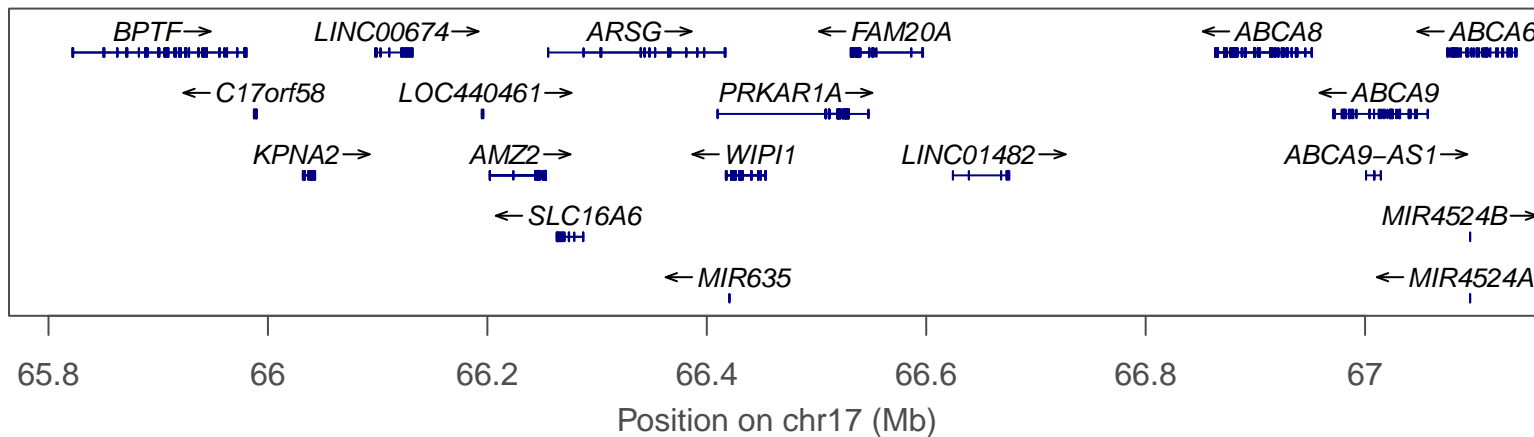

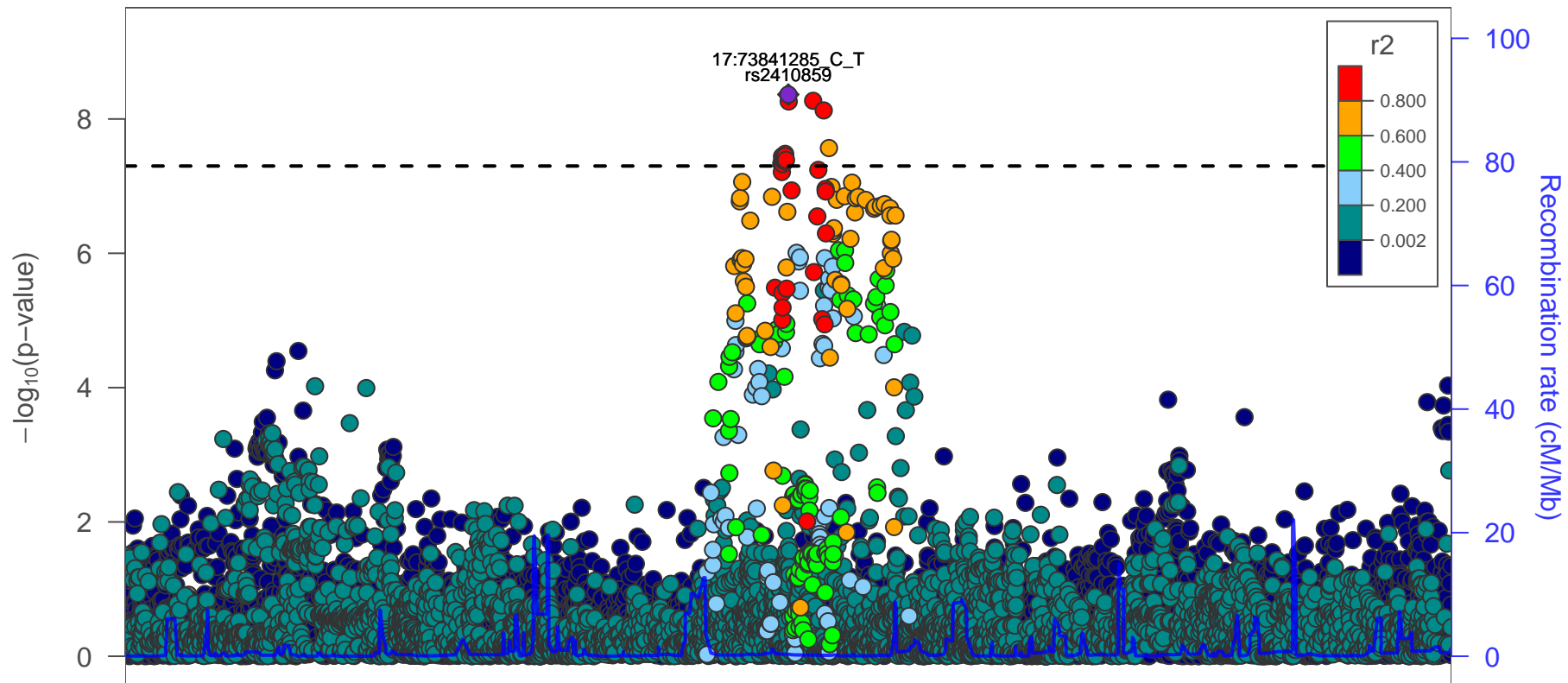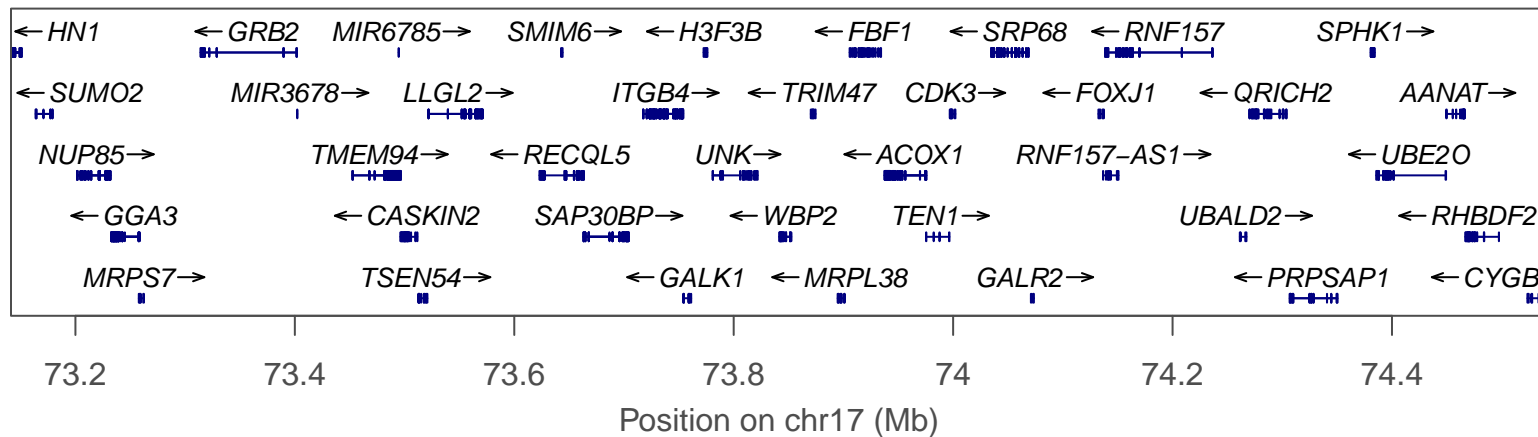

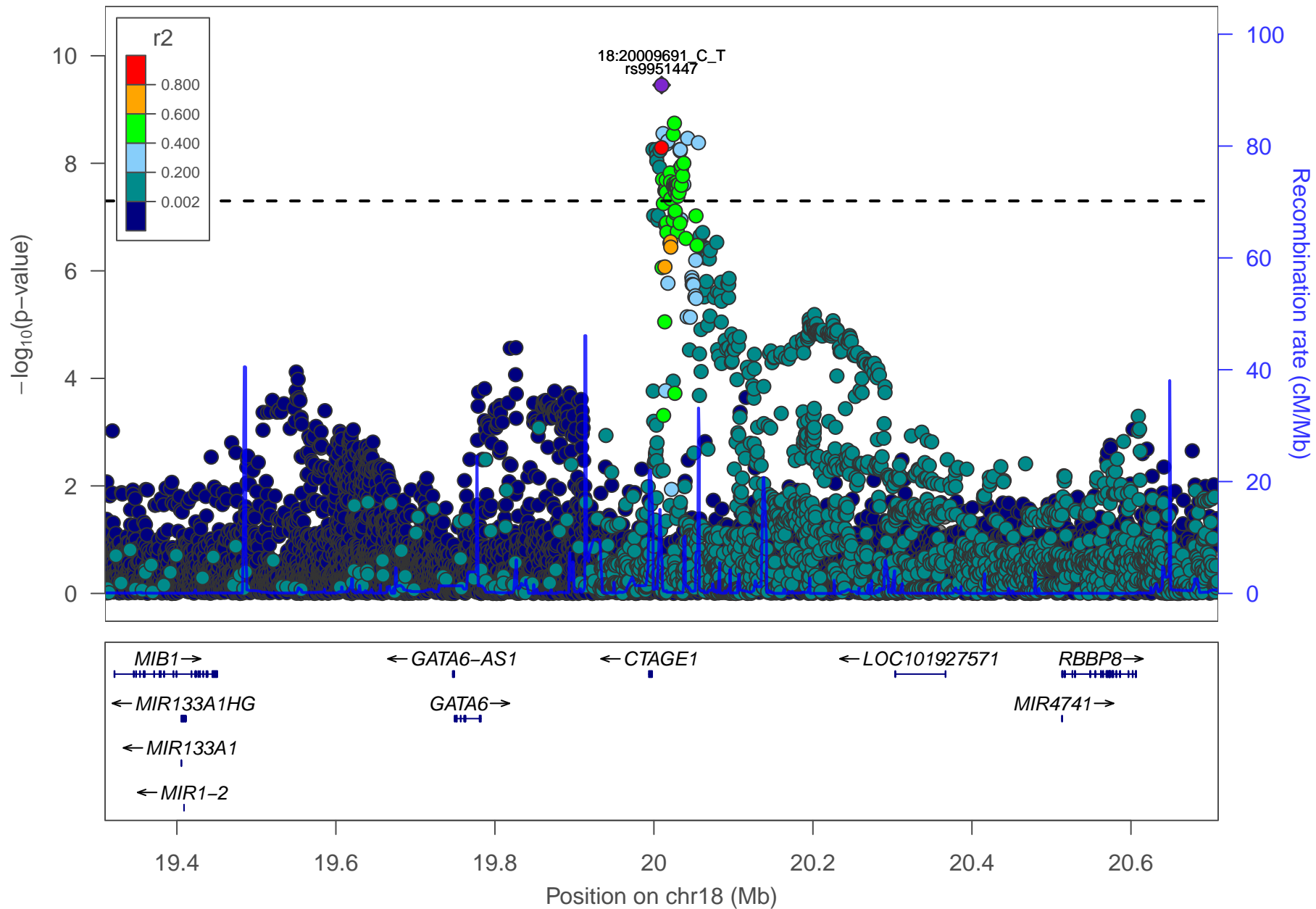

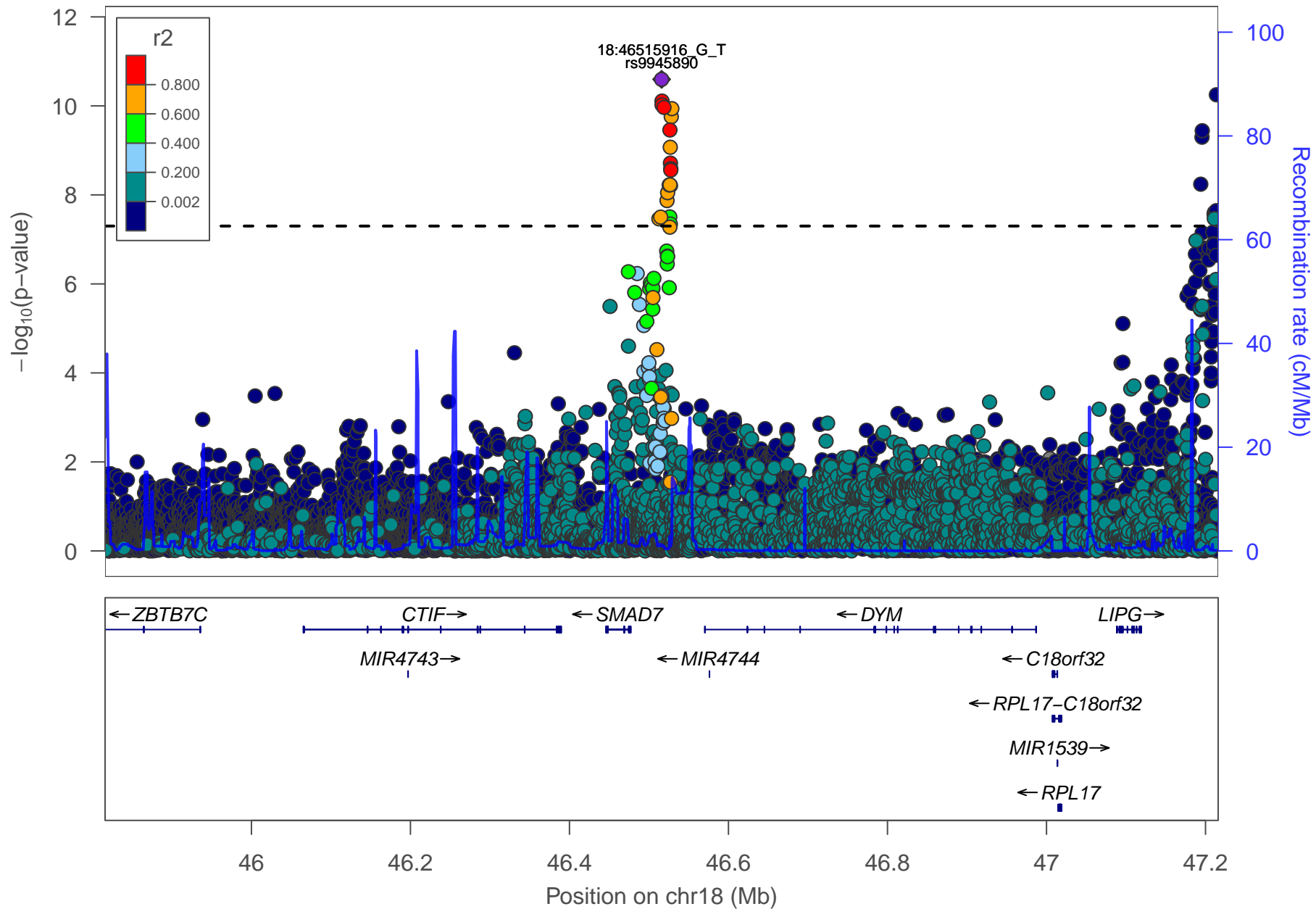

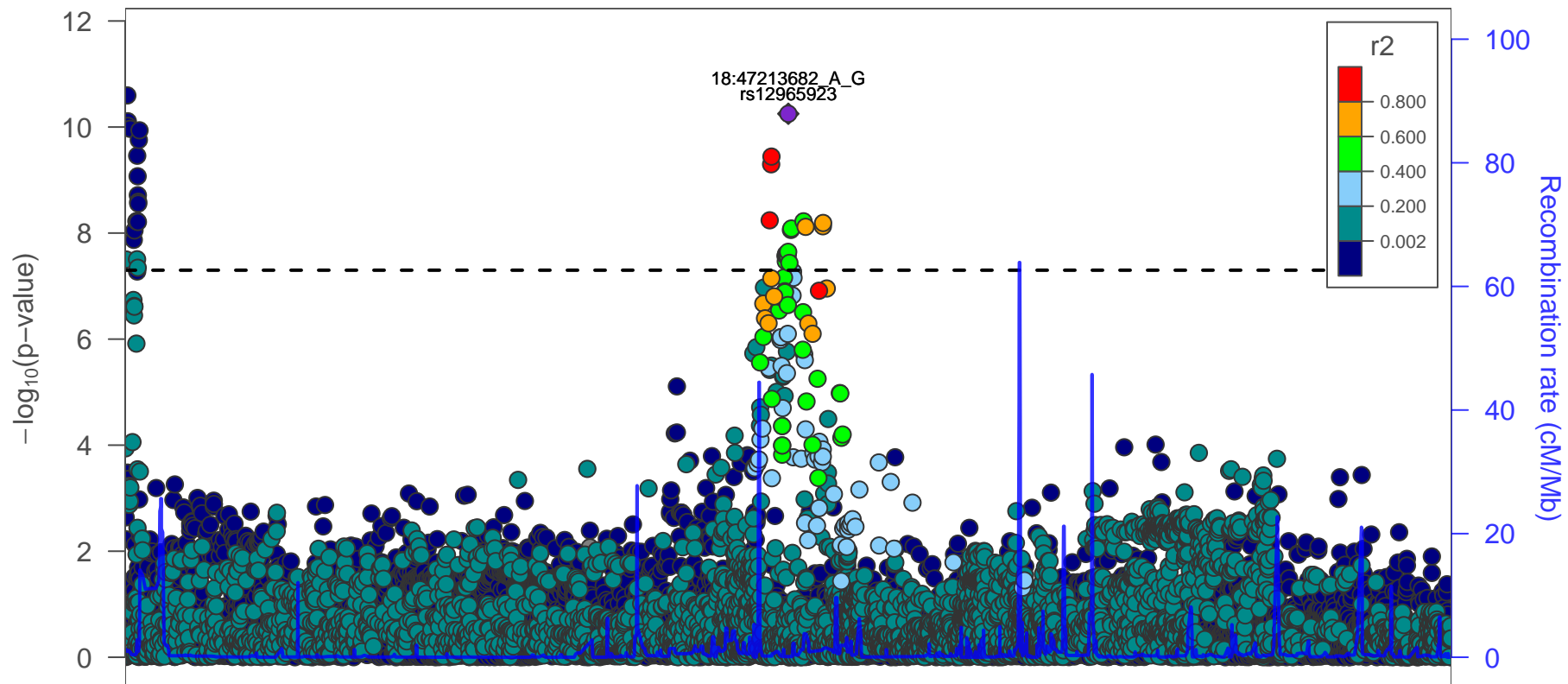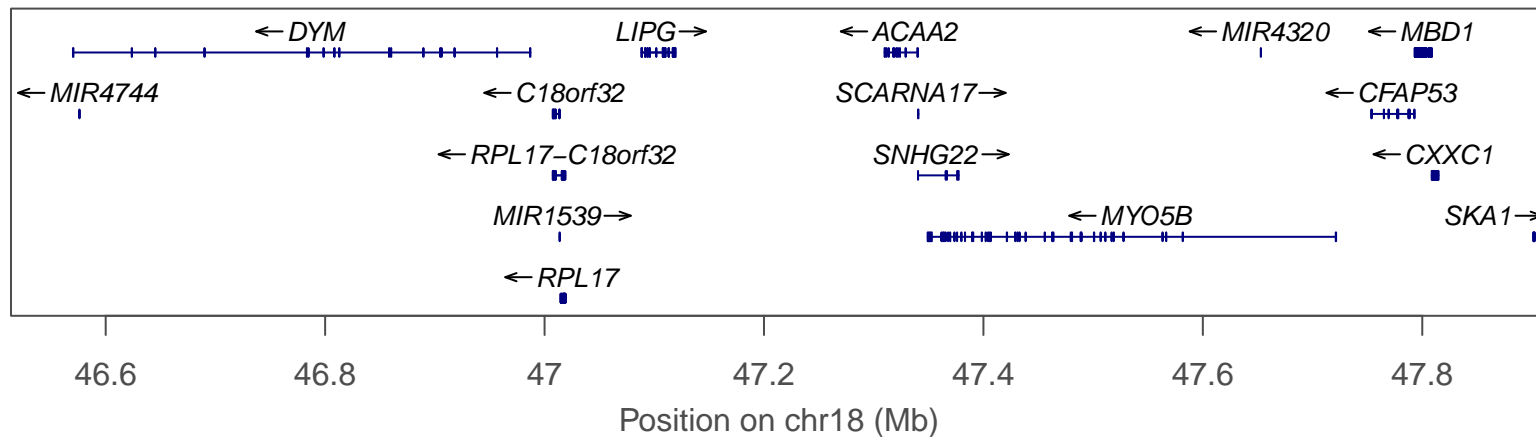

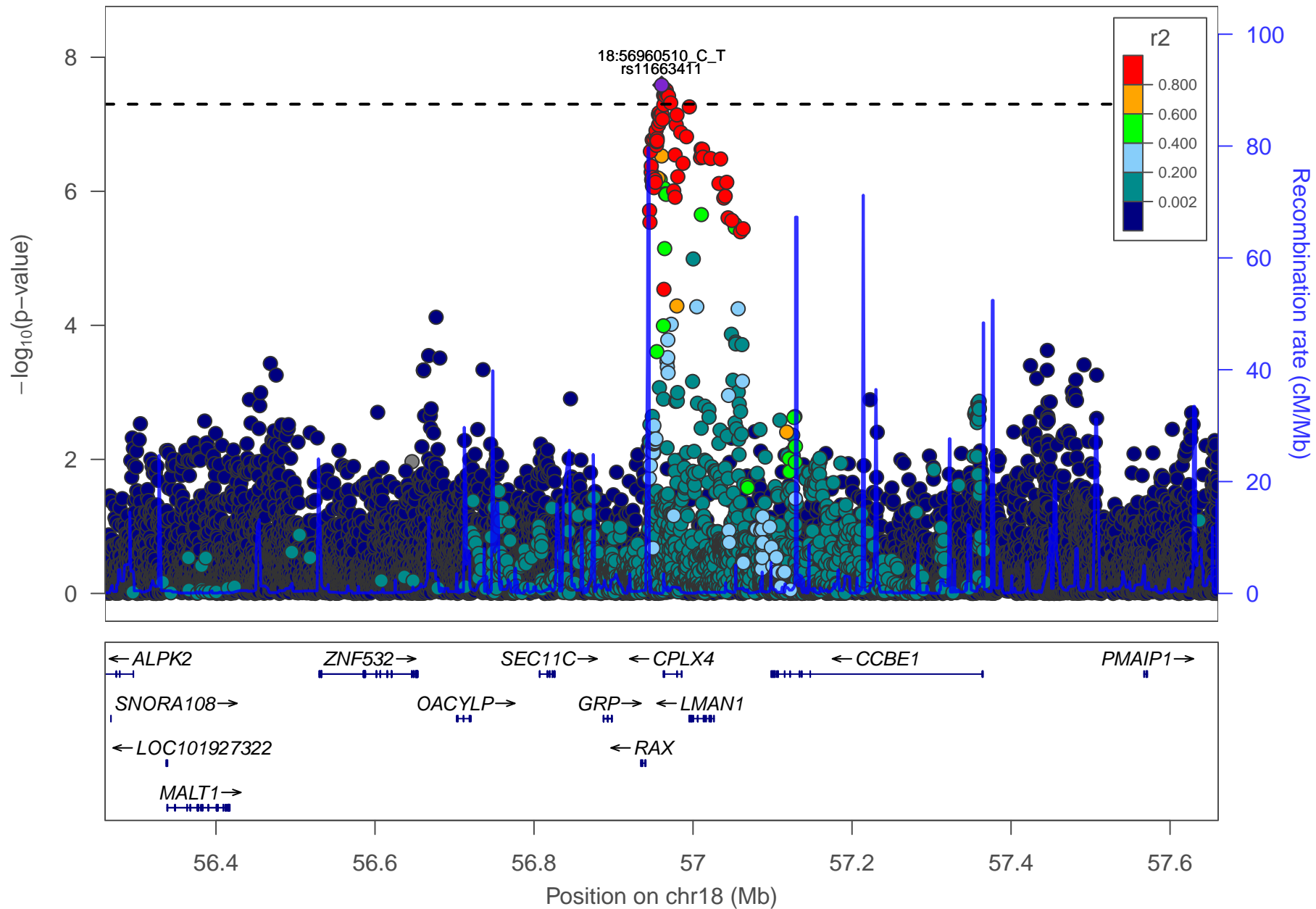

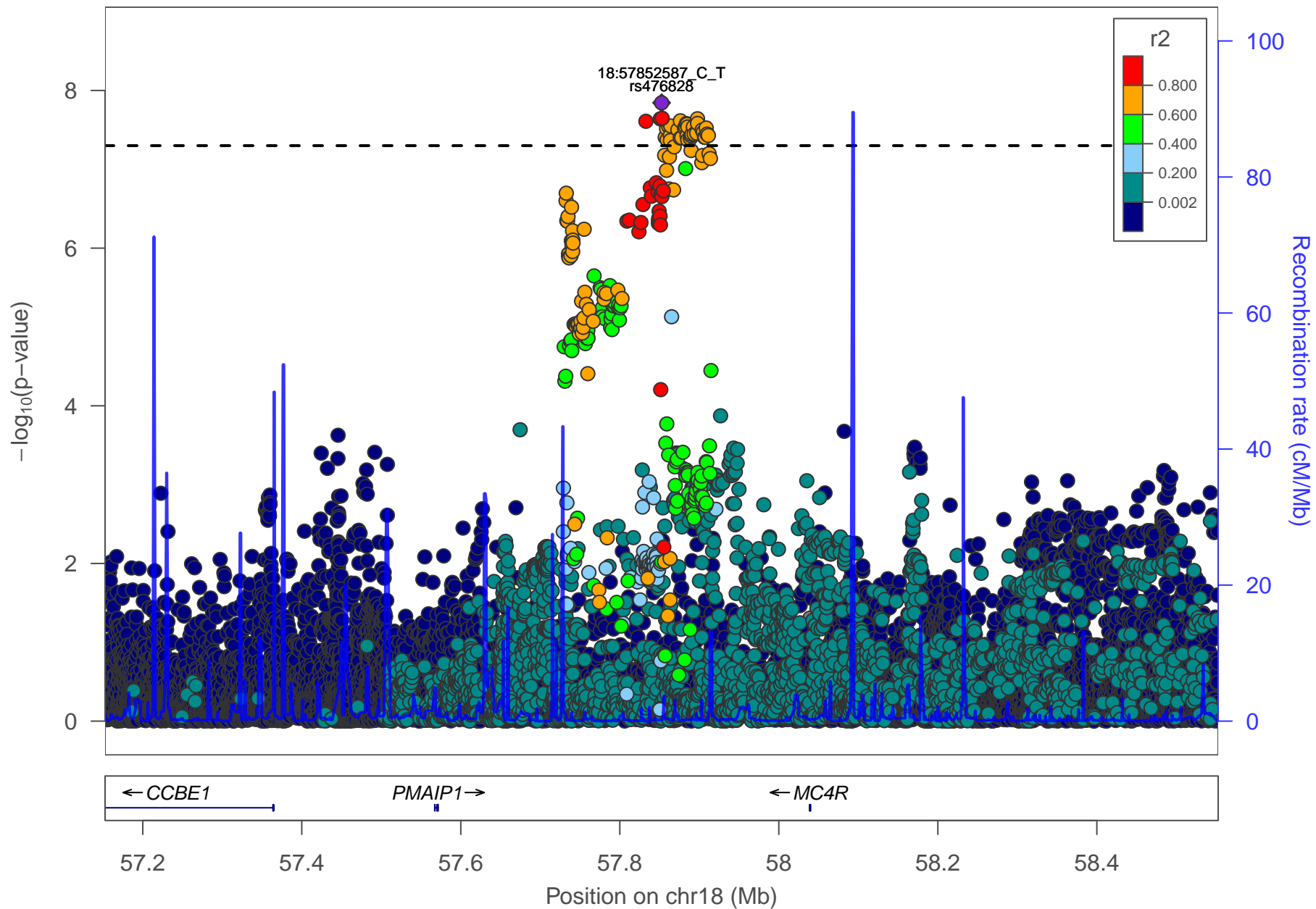

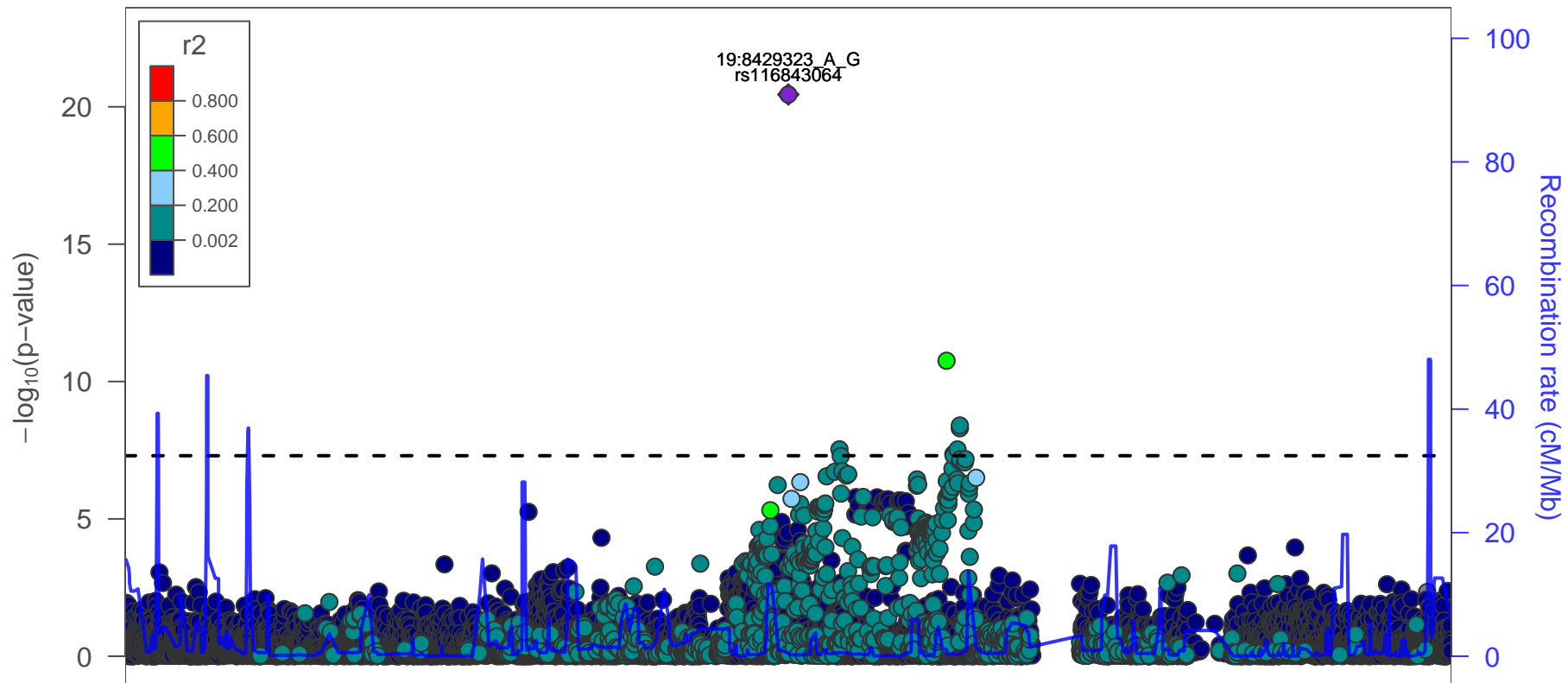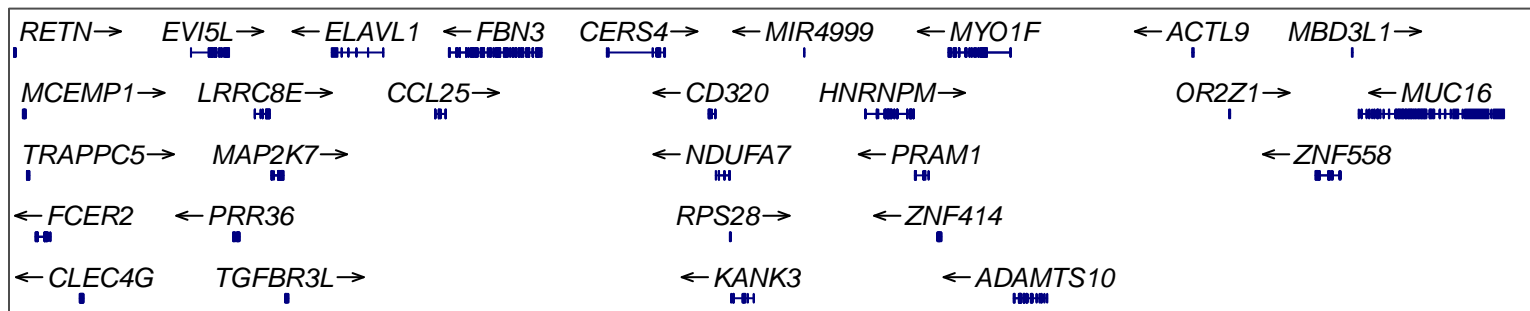

Position on chr19 (Mb)

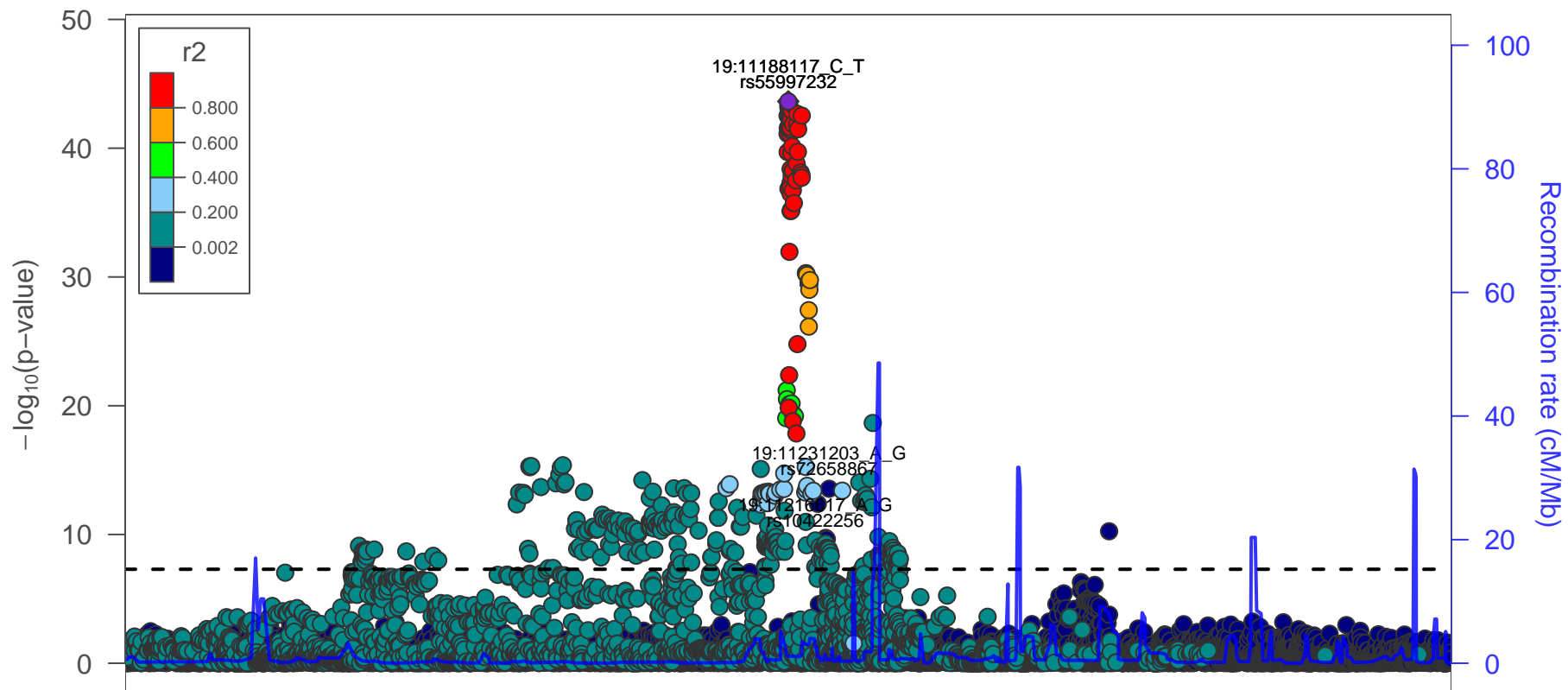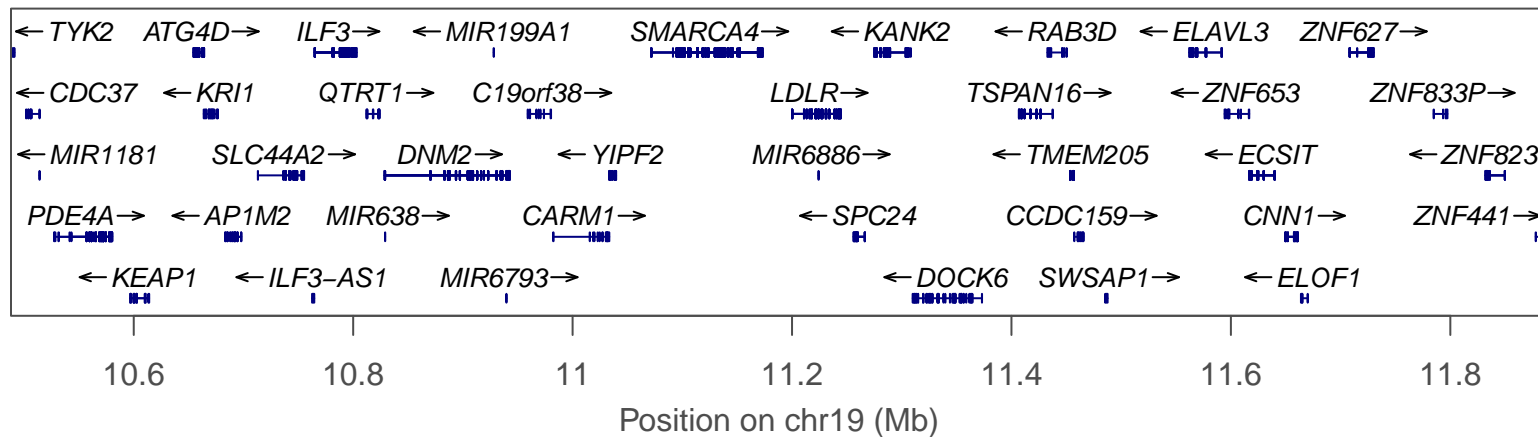

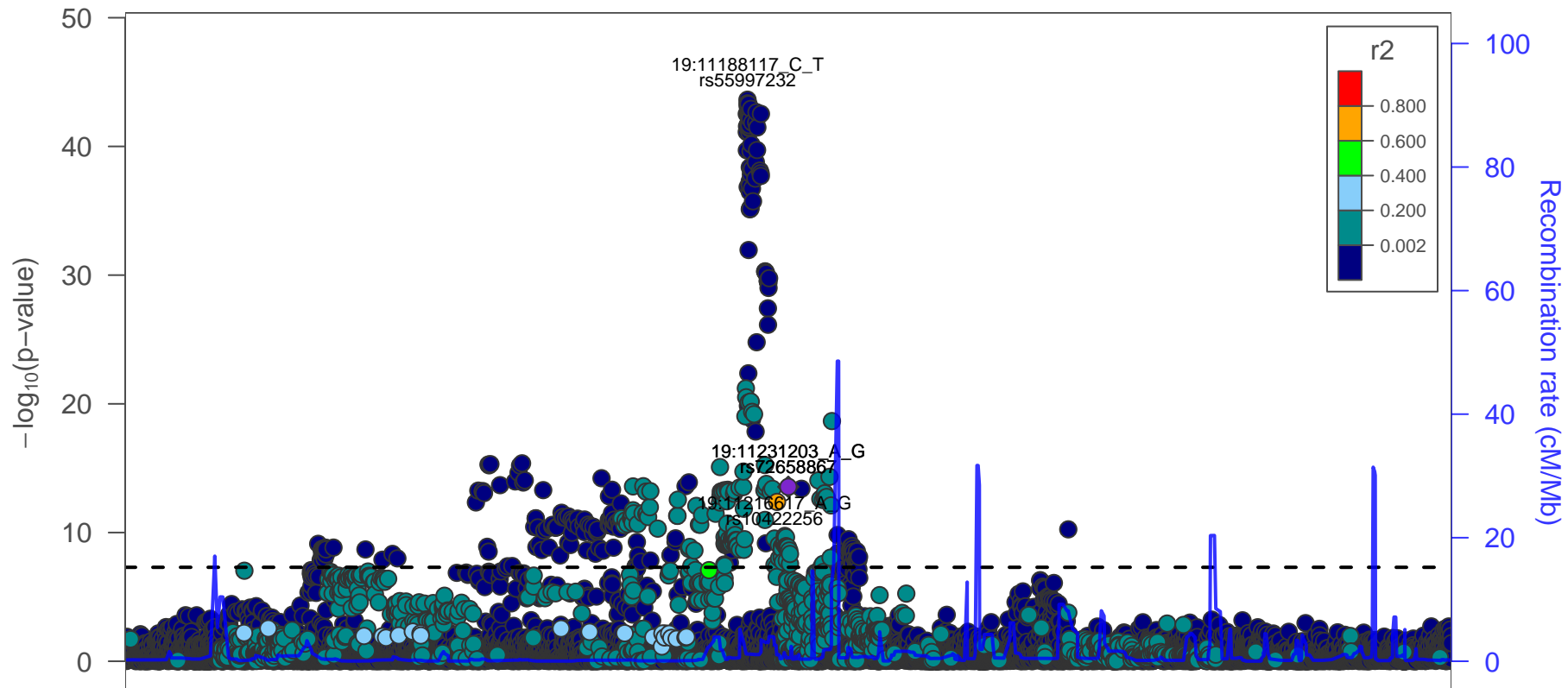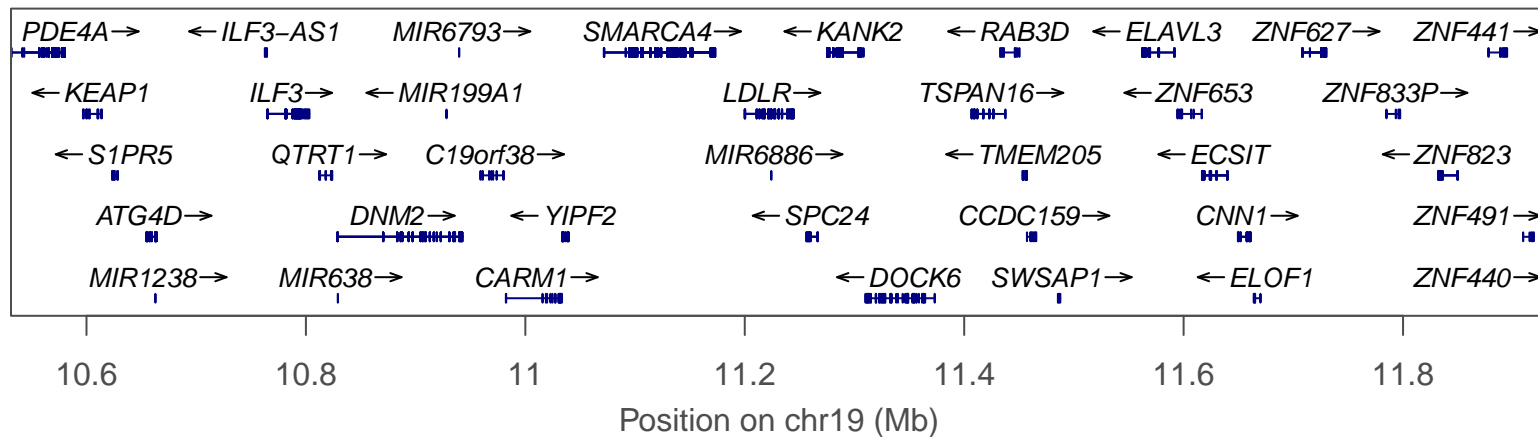

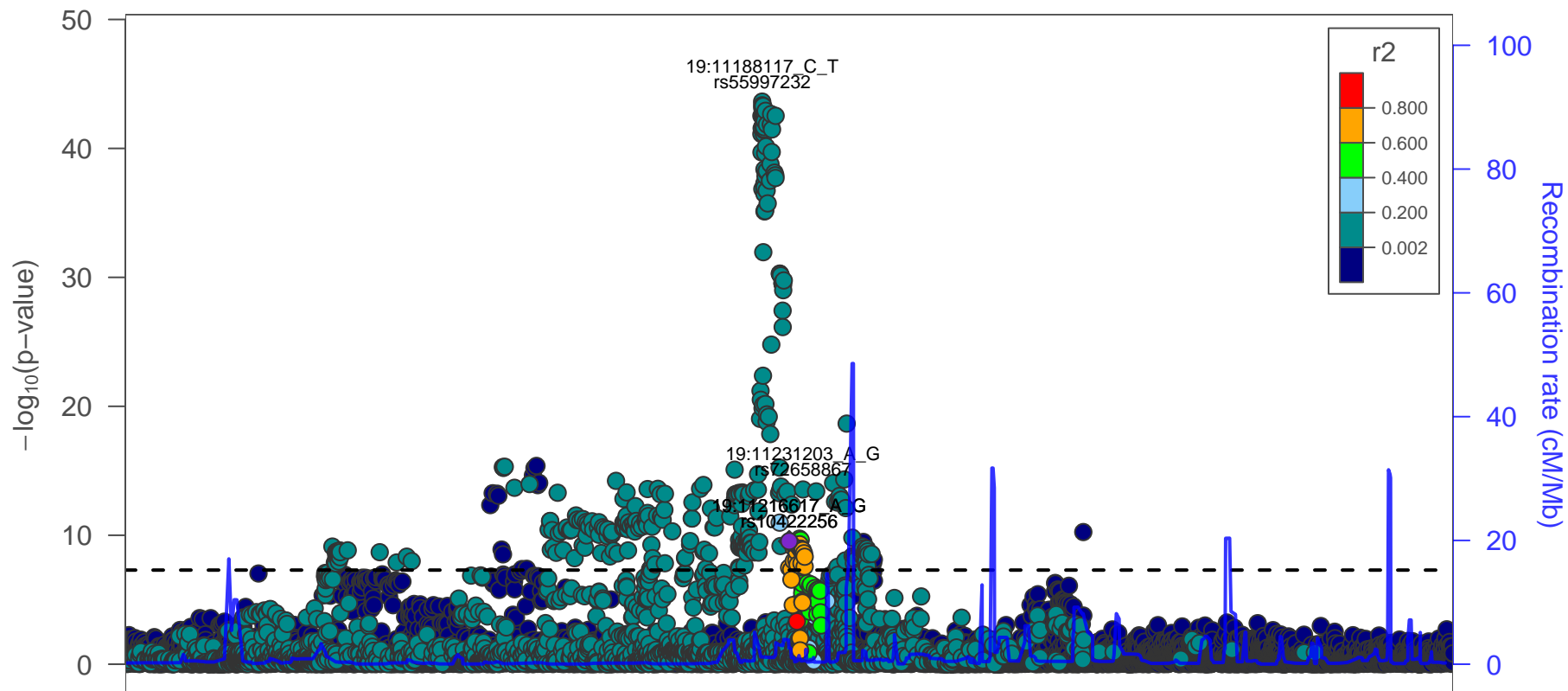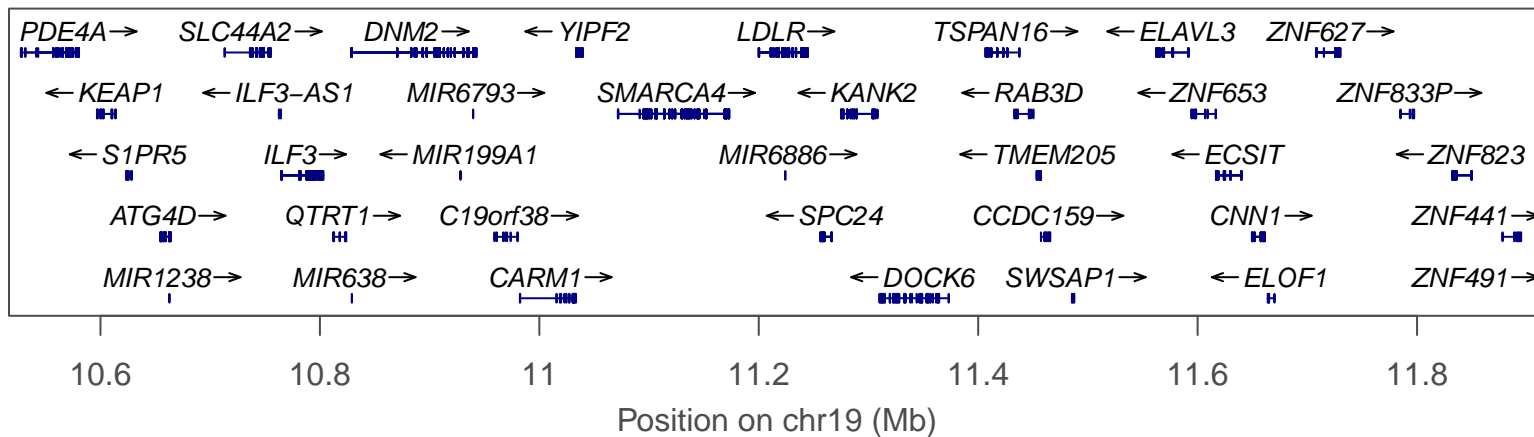

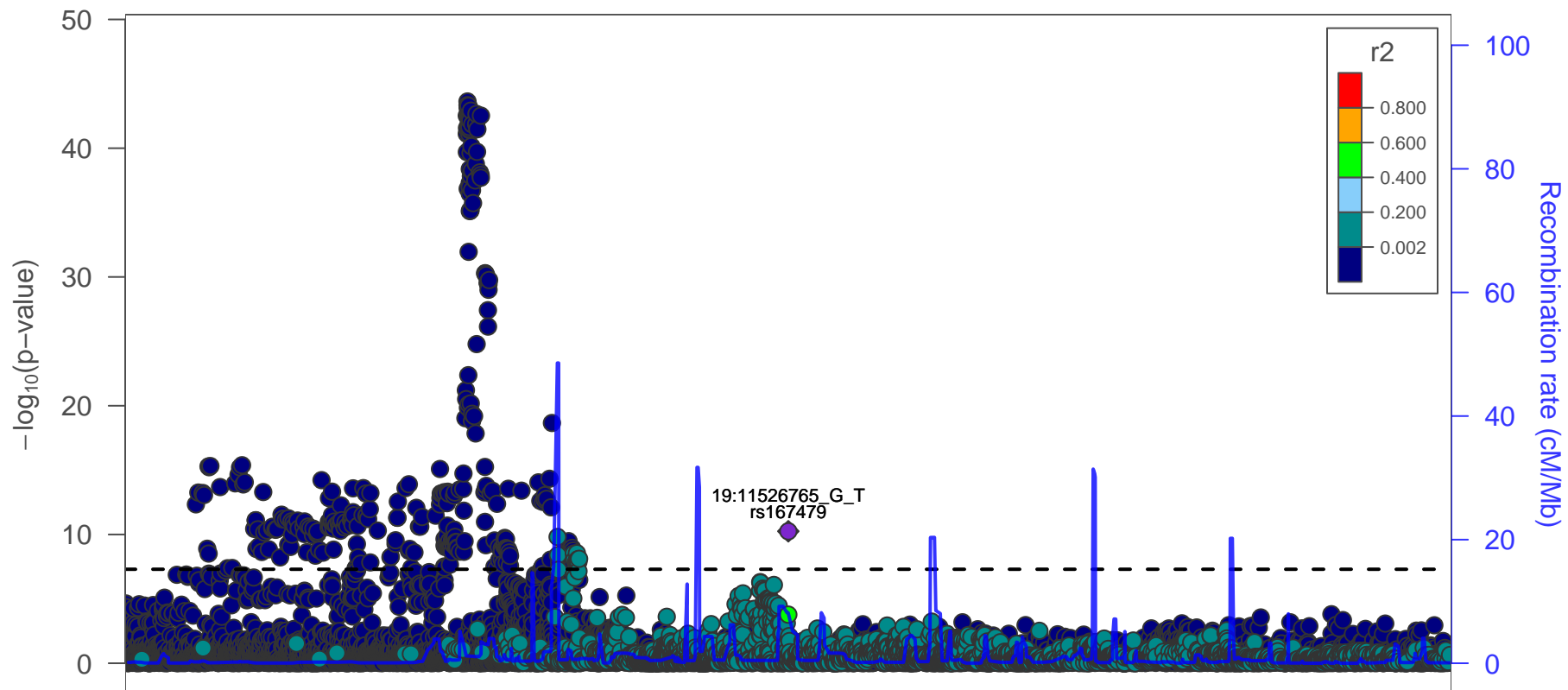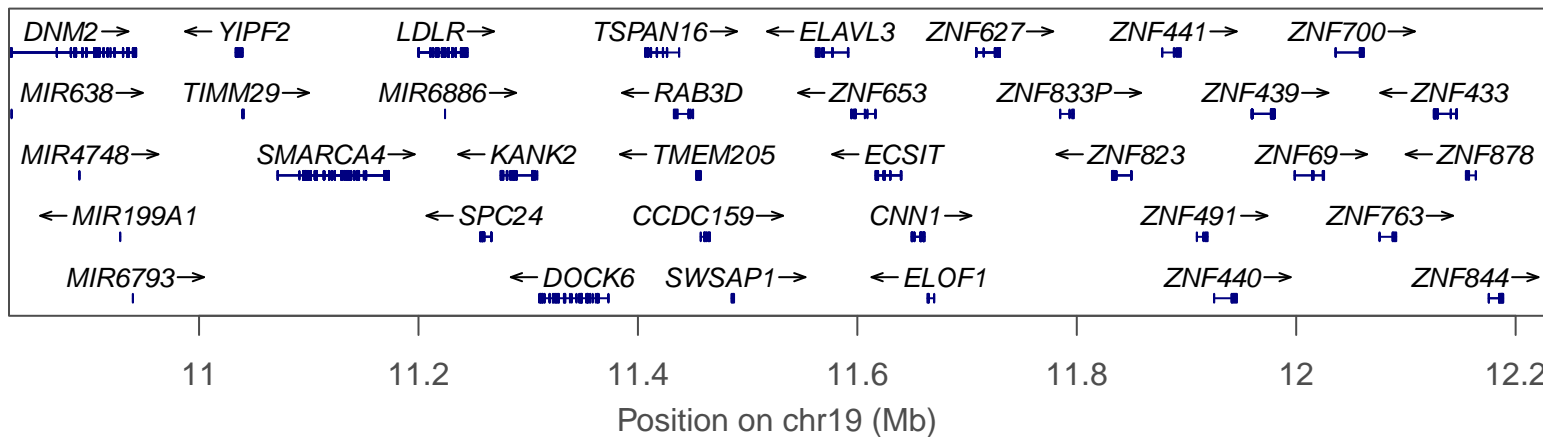

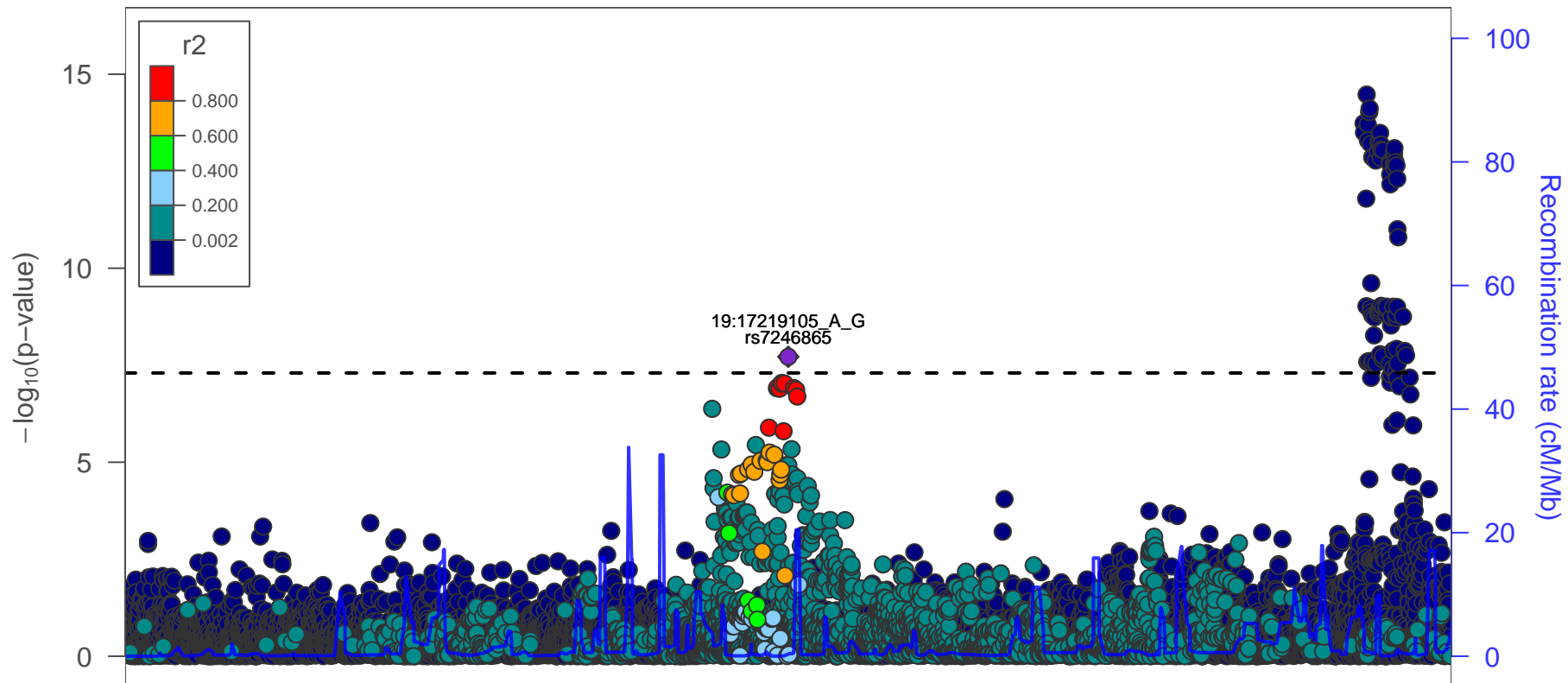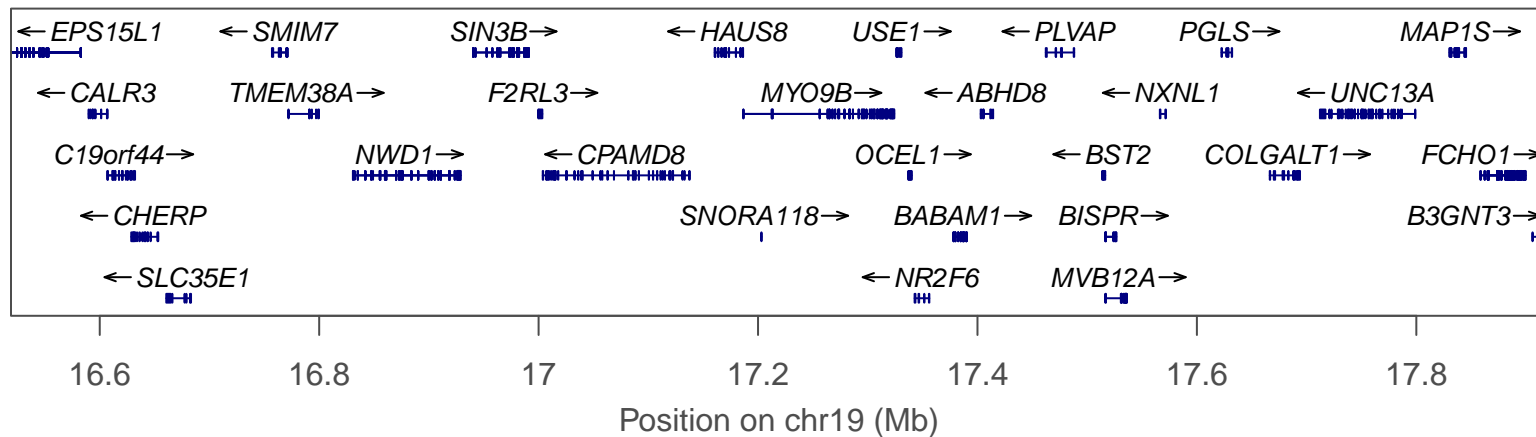

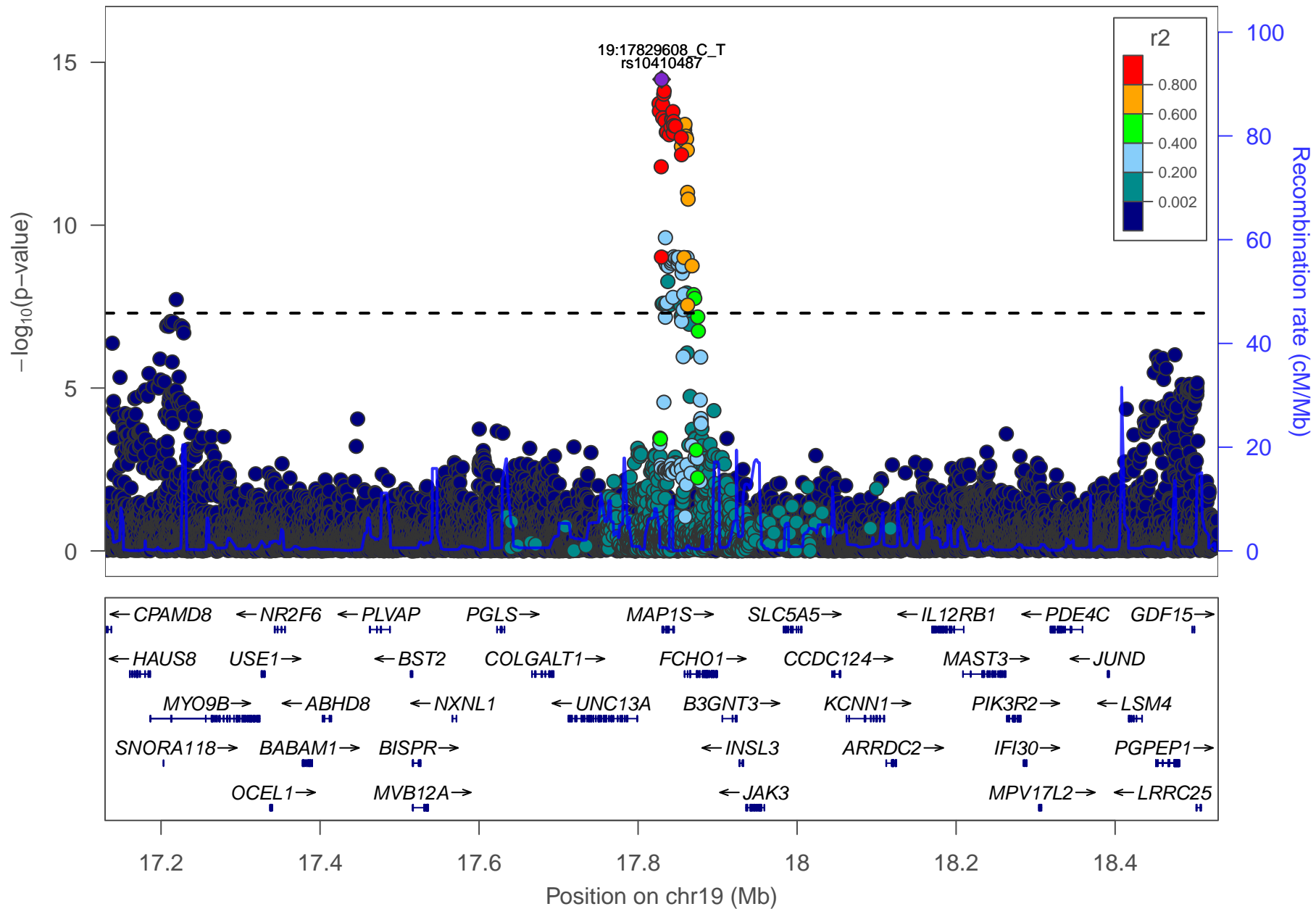

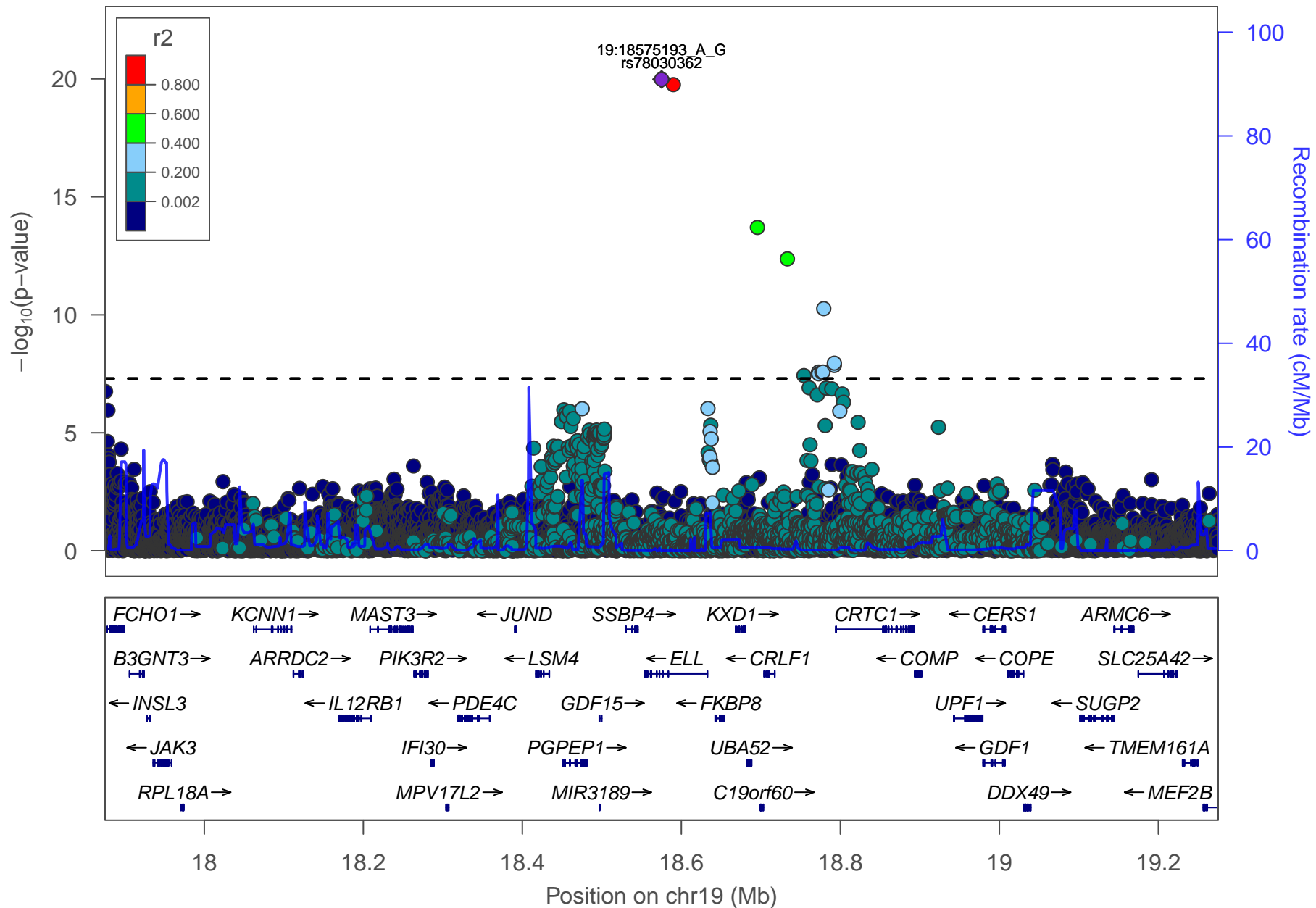

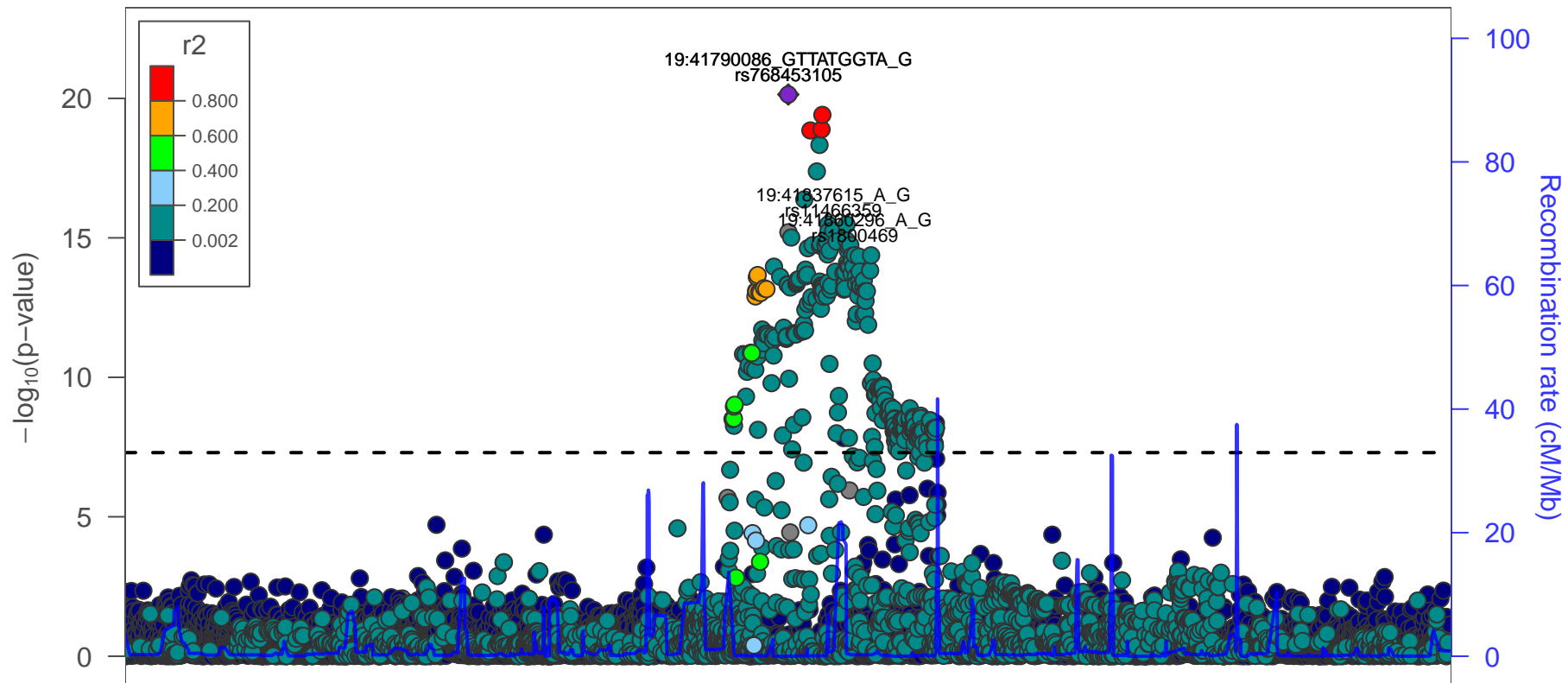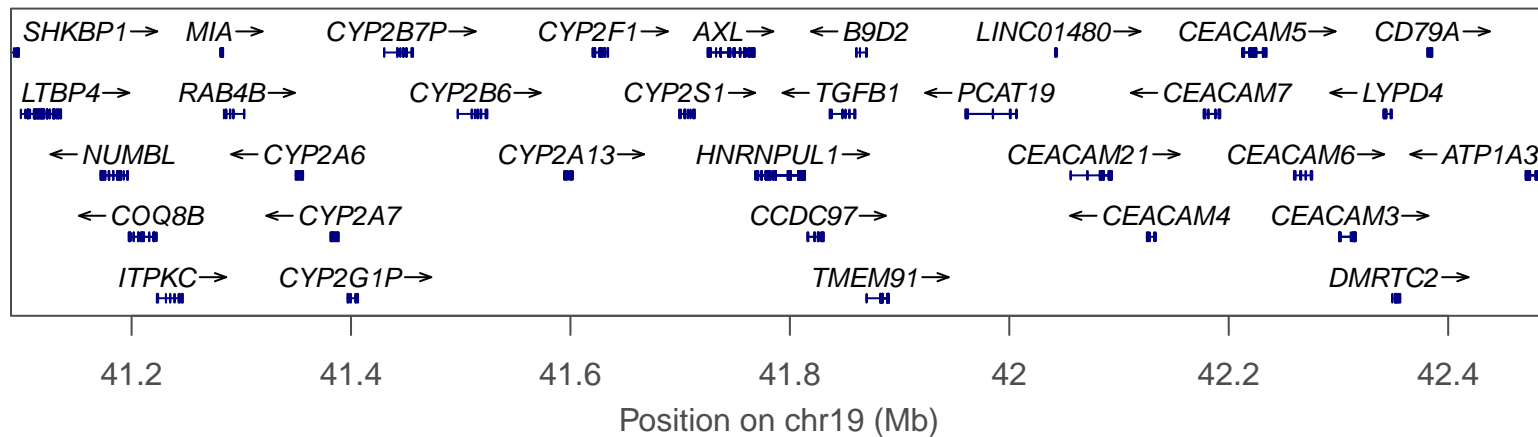

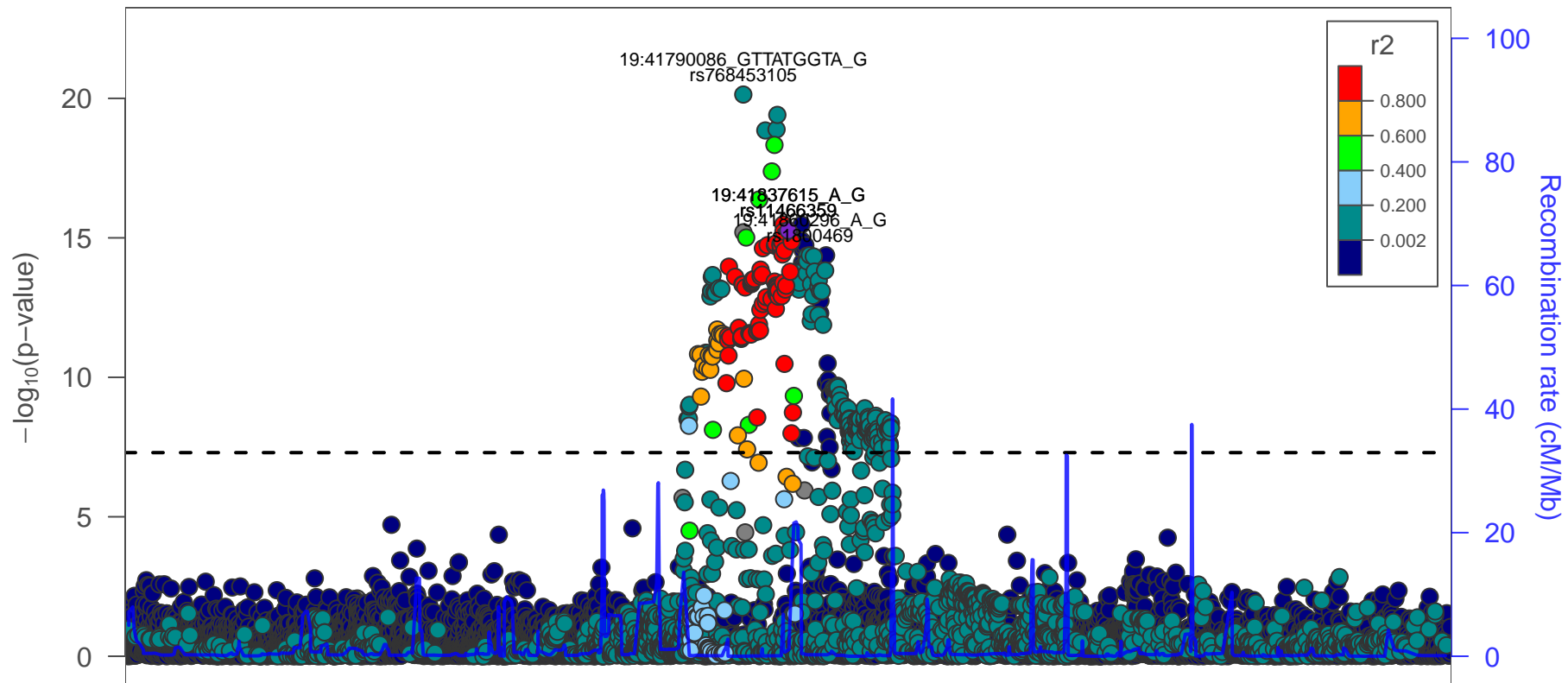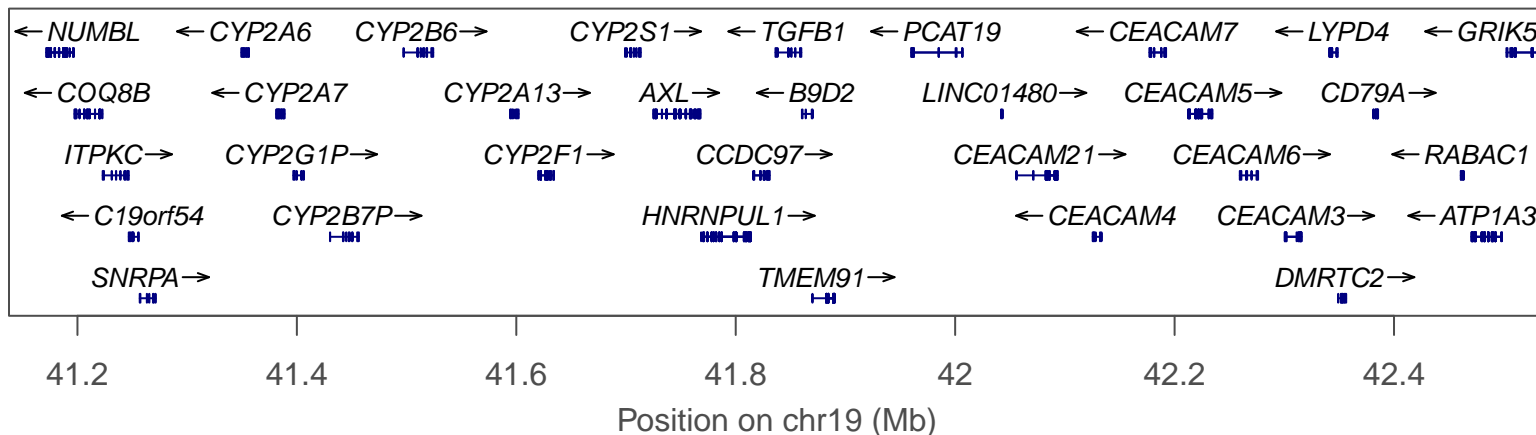

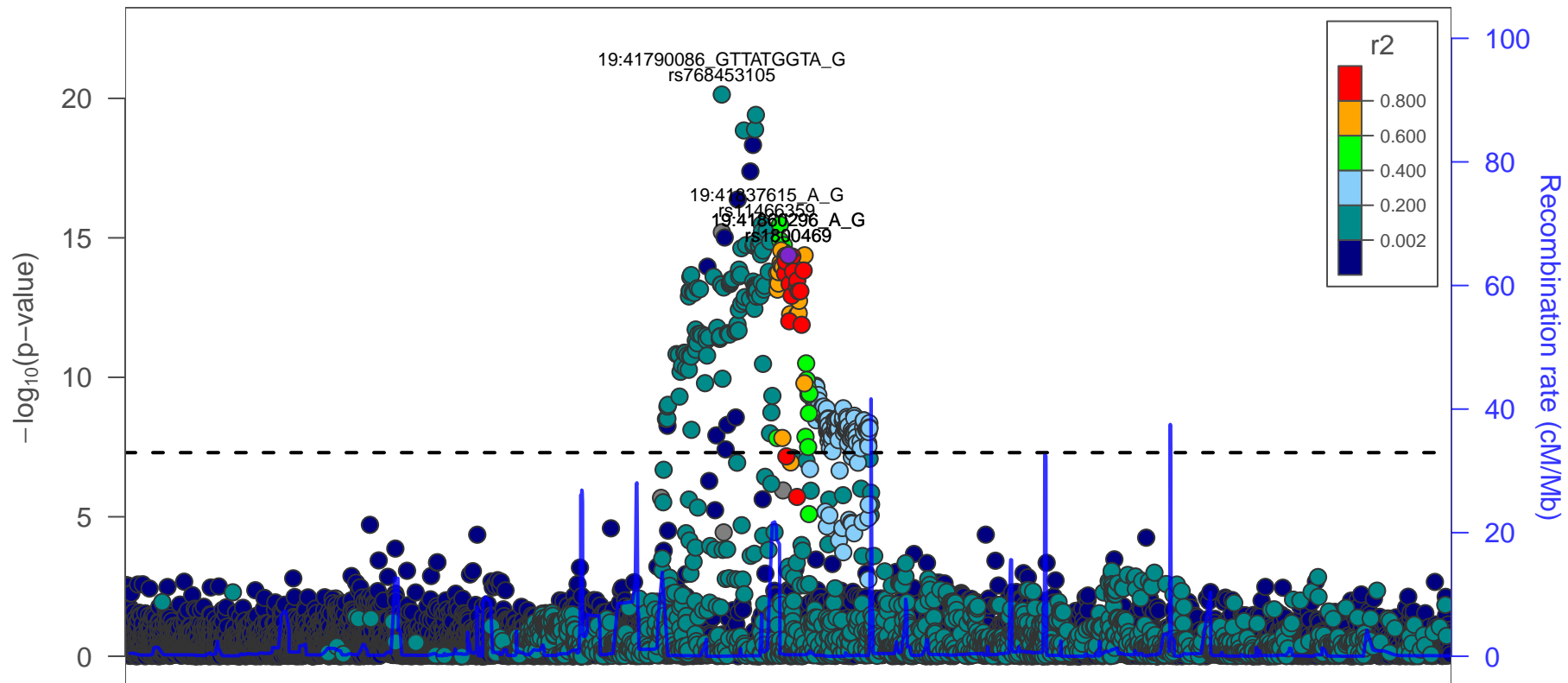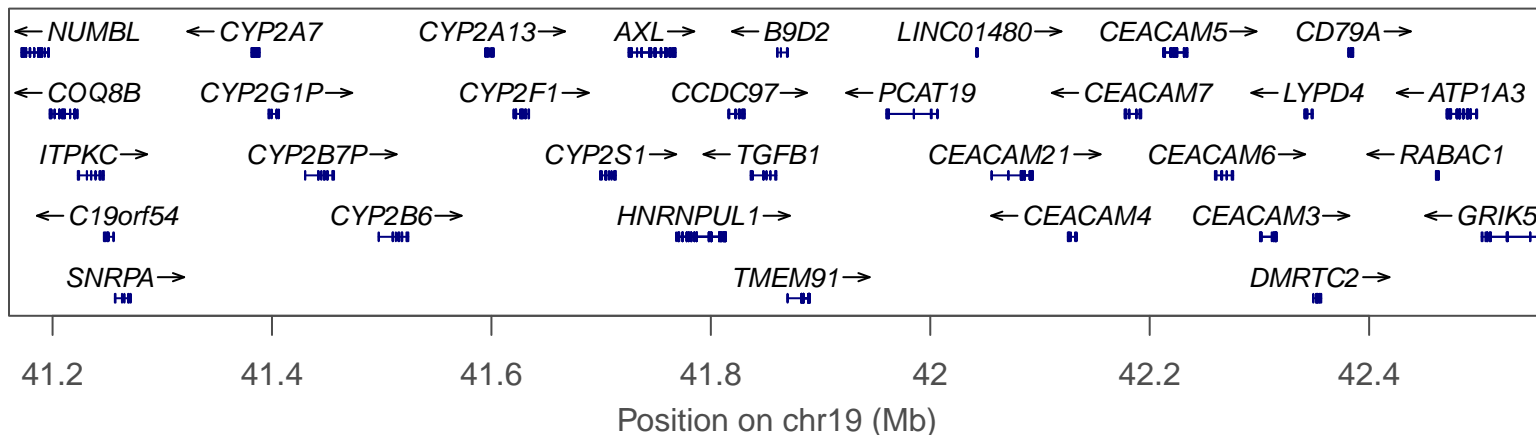

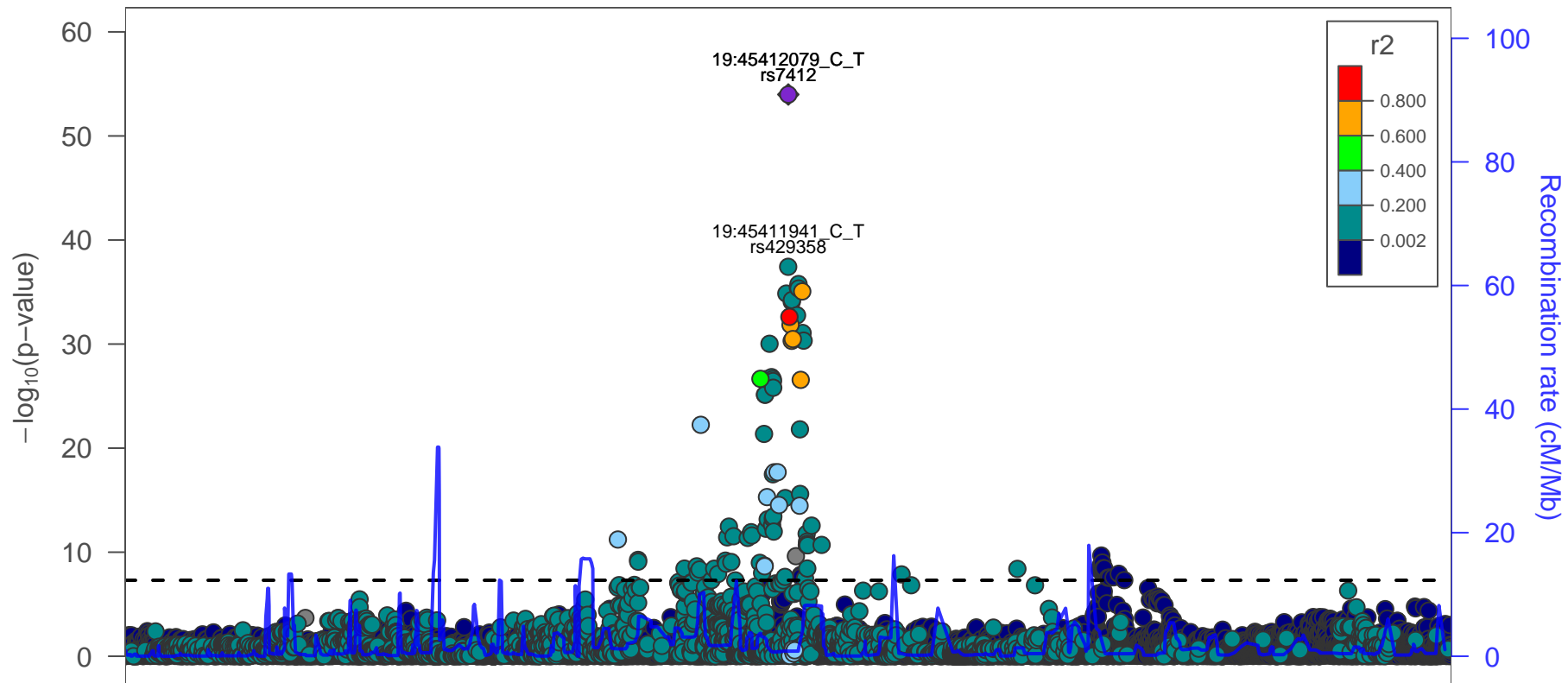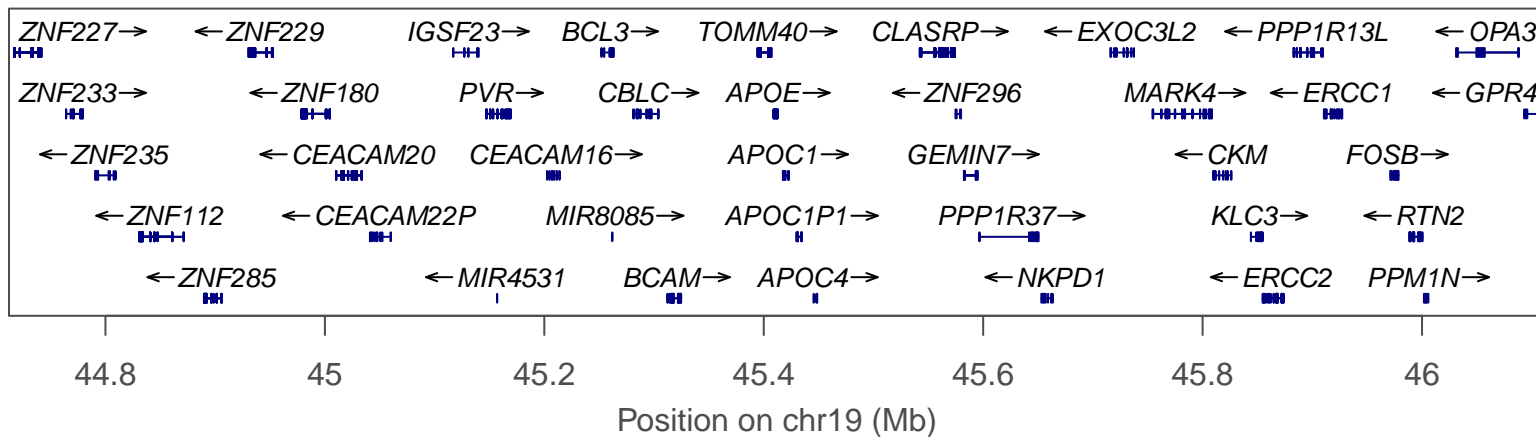

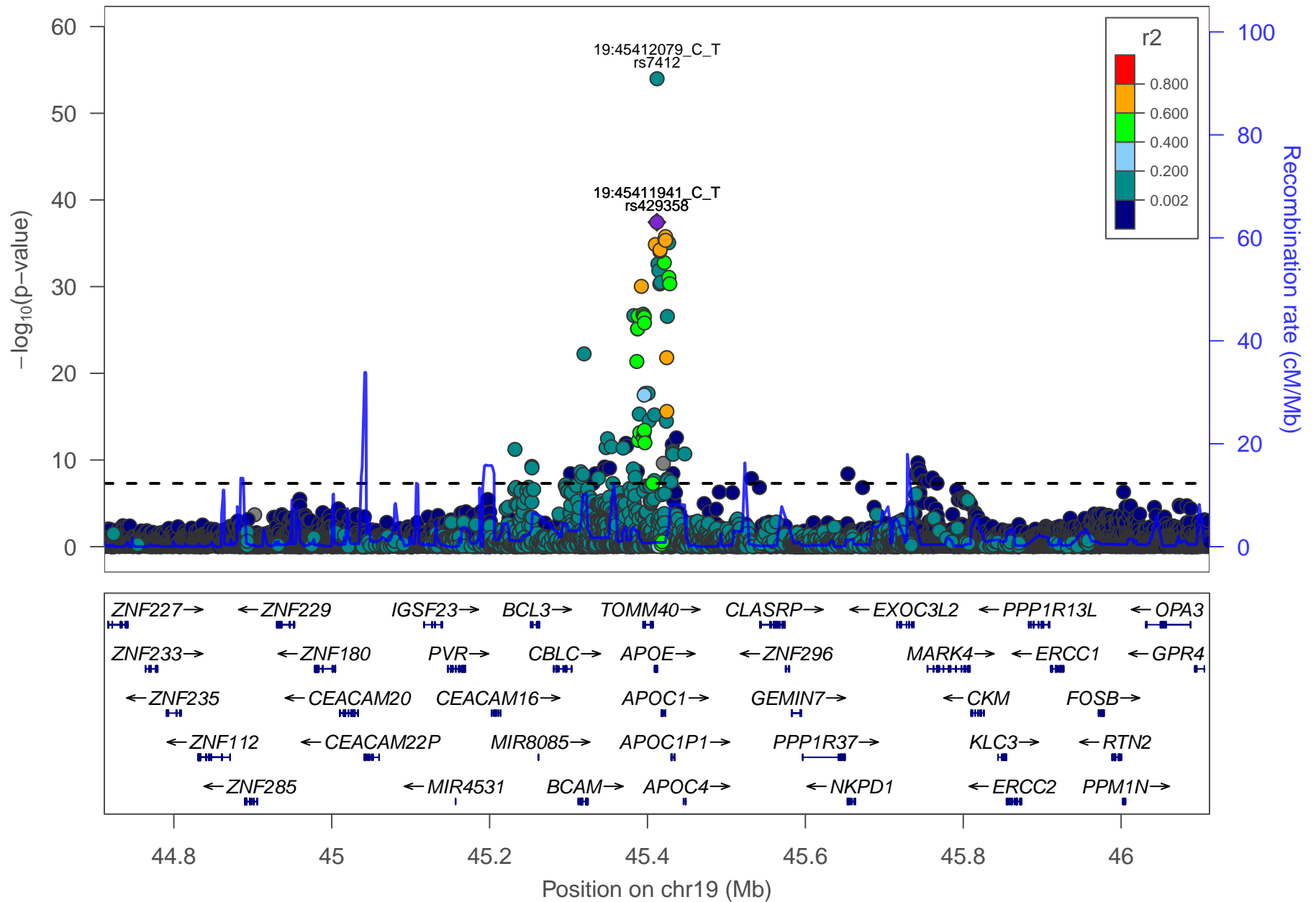

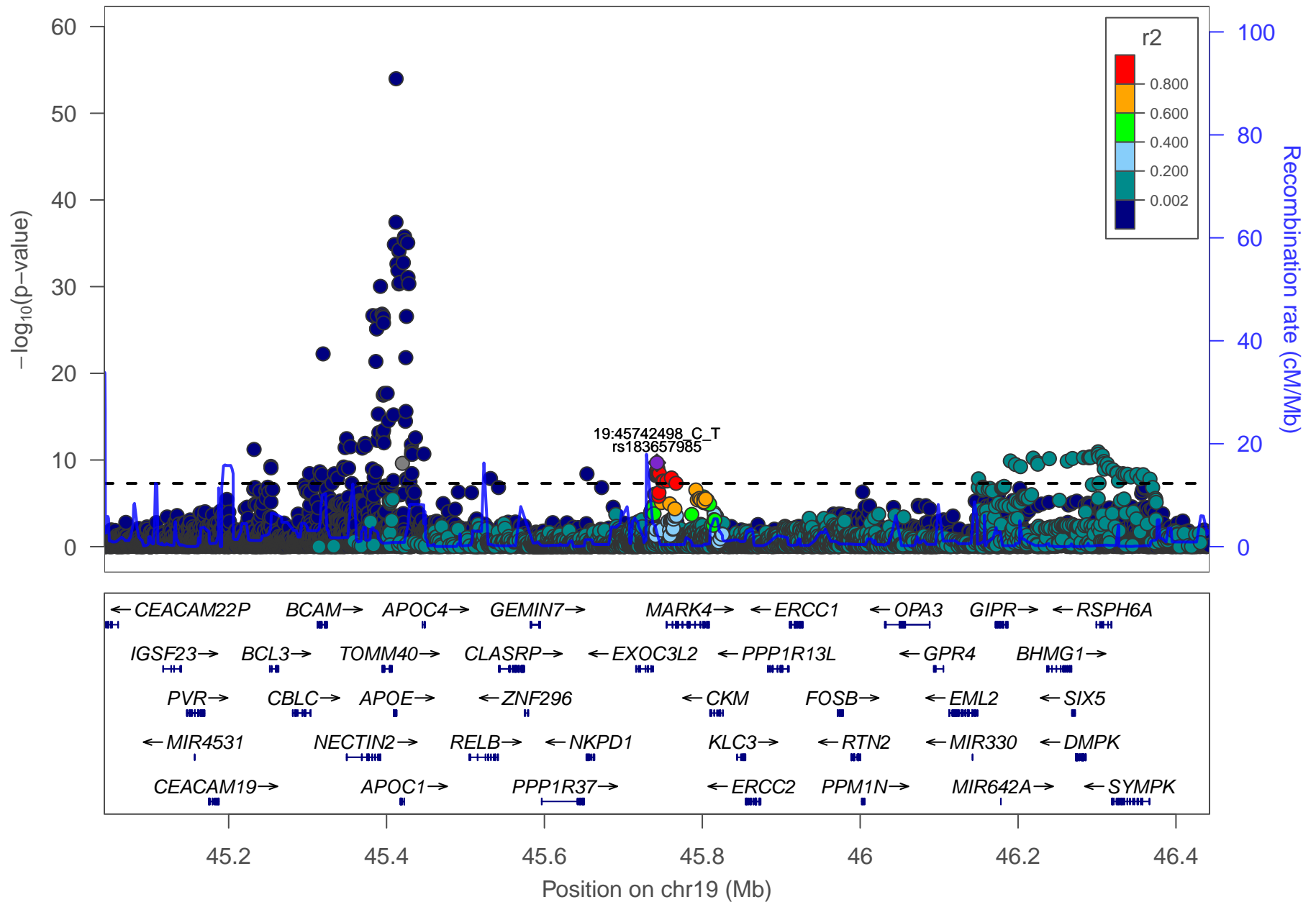

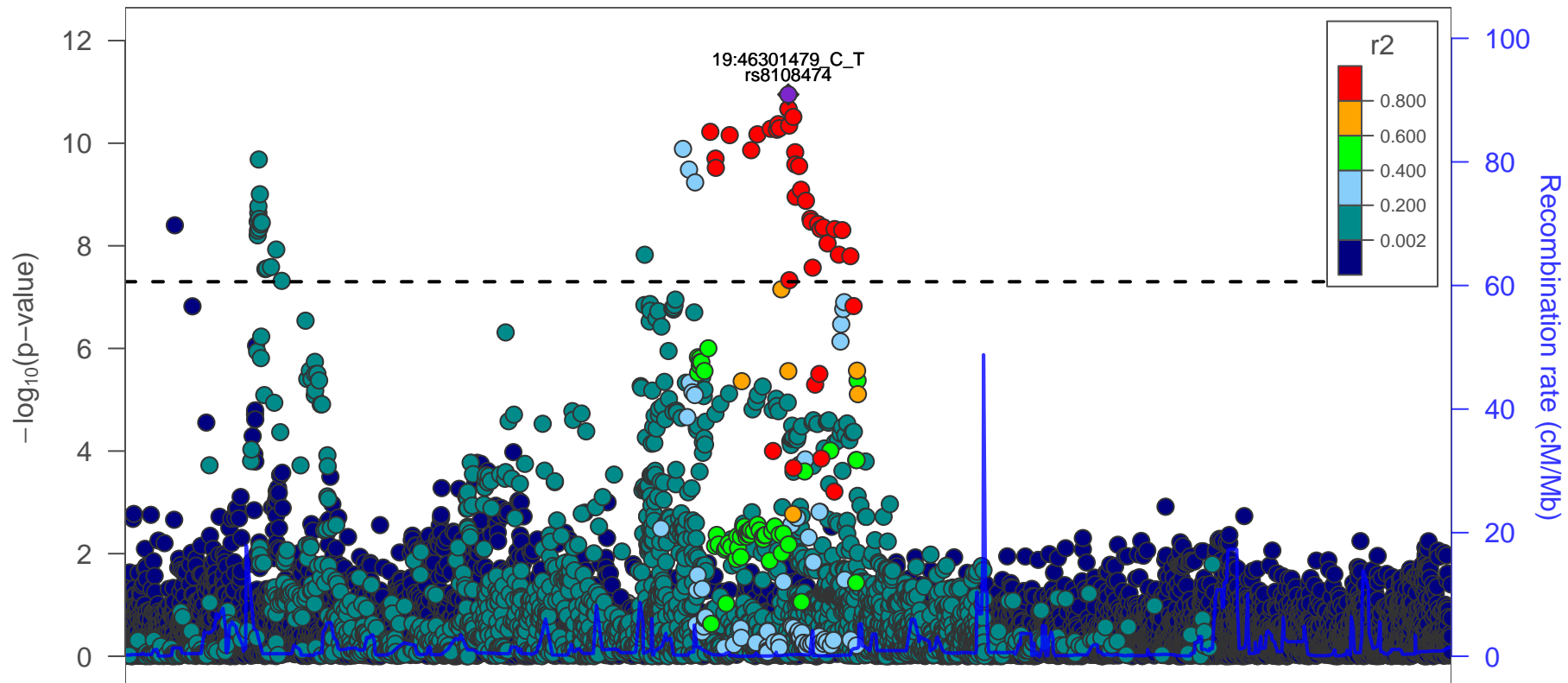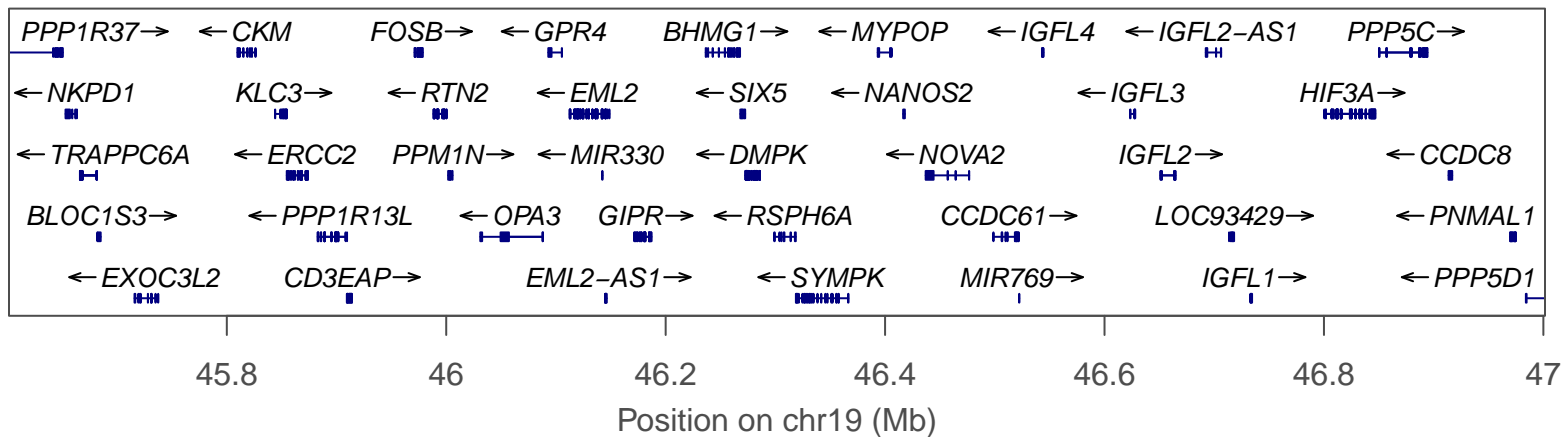

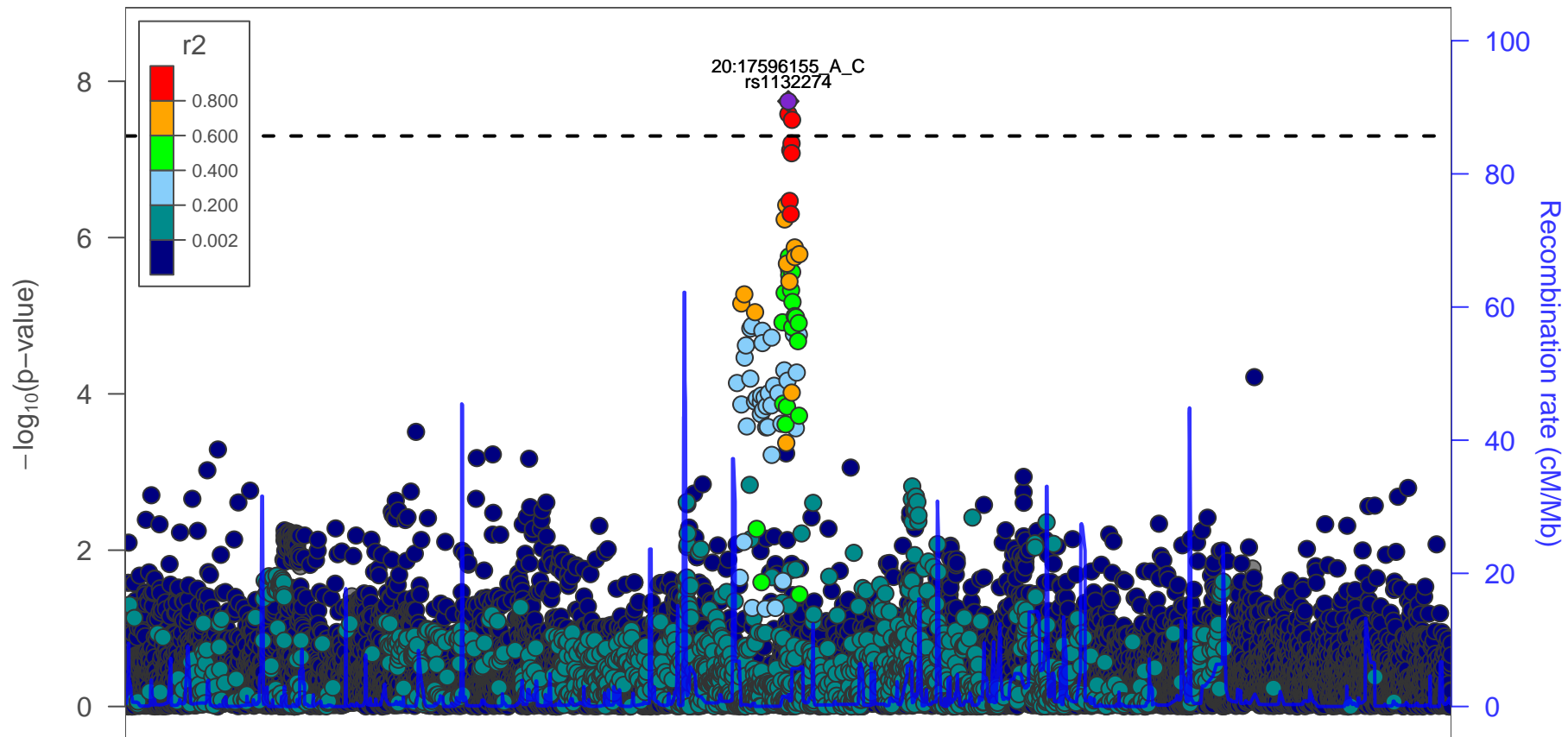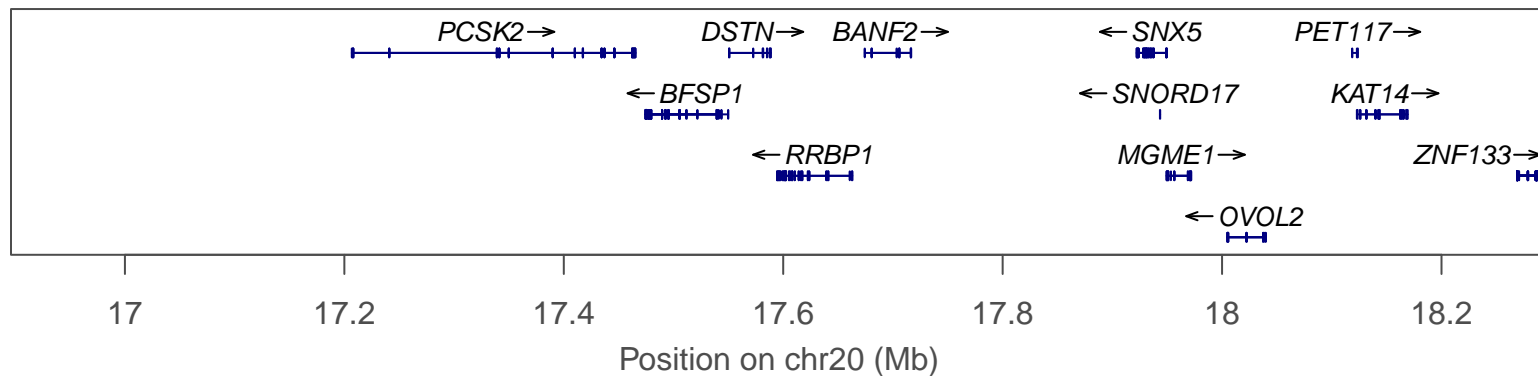

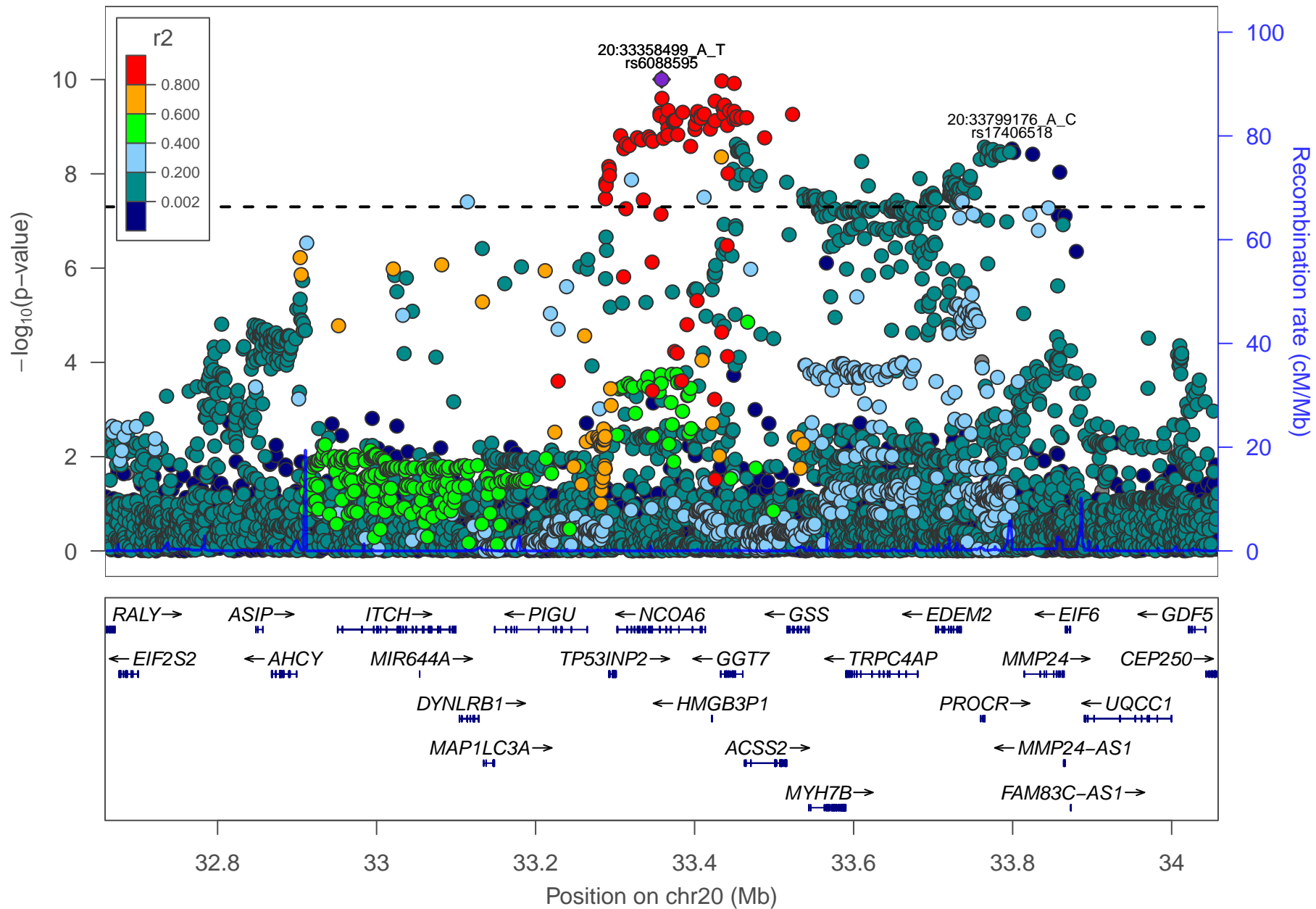

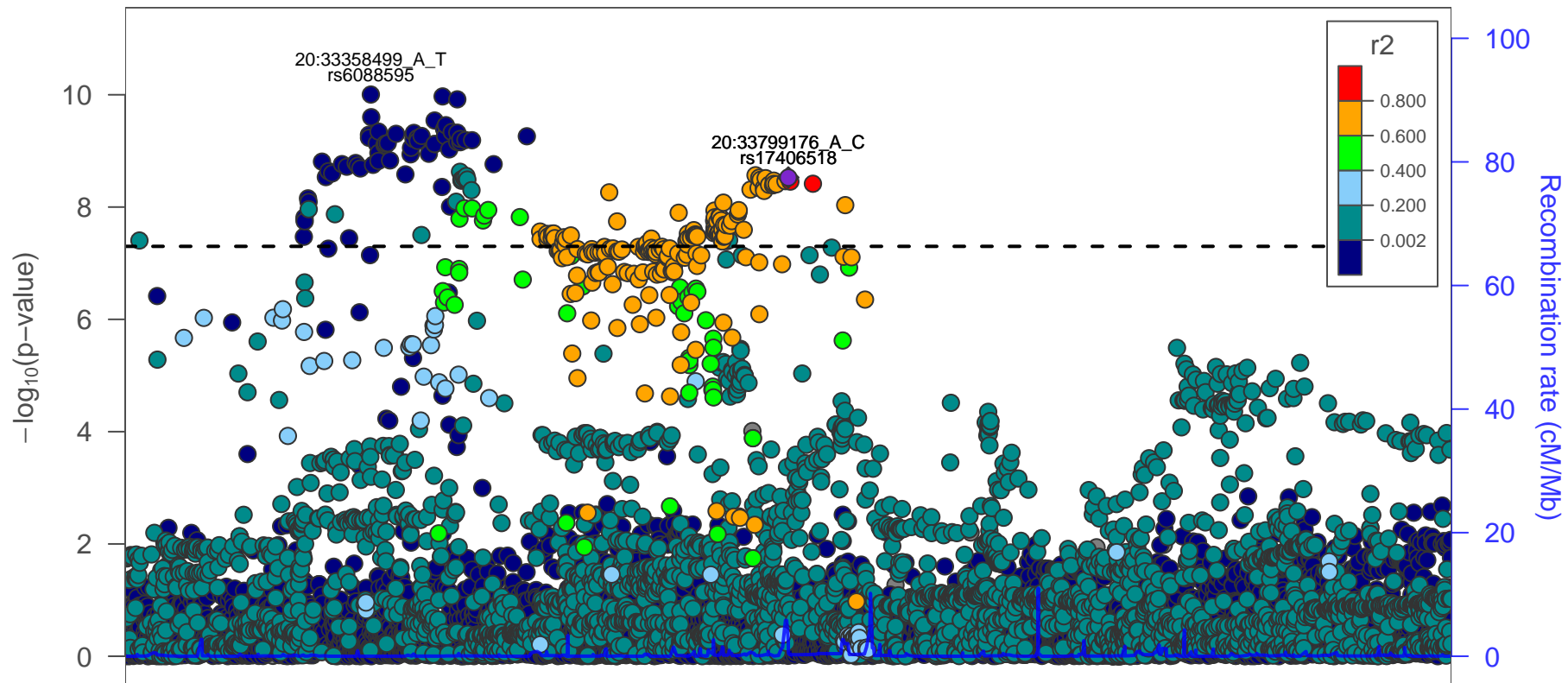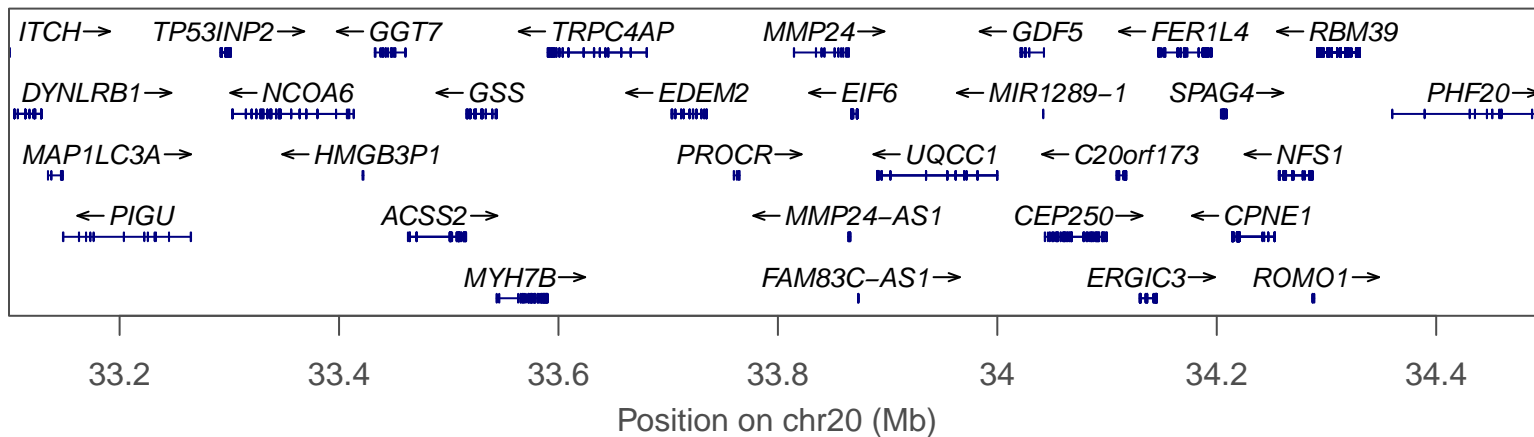

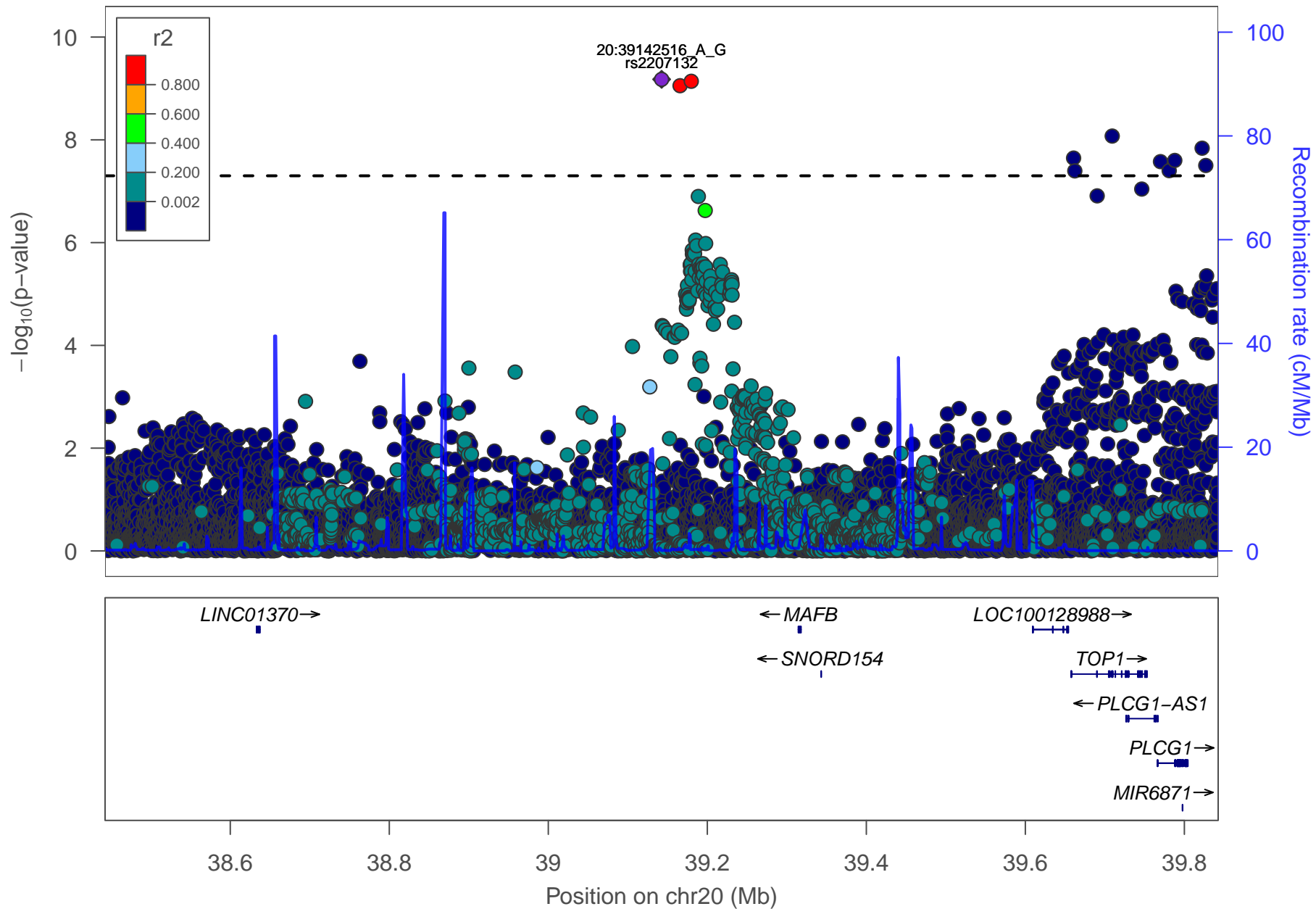

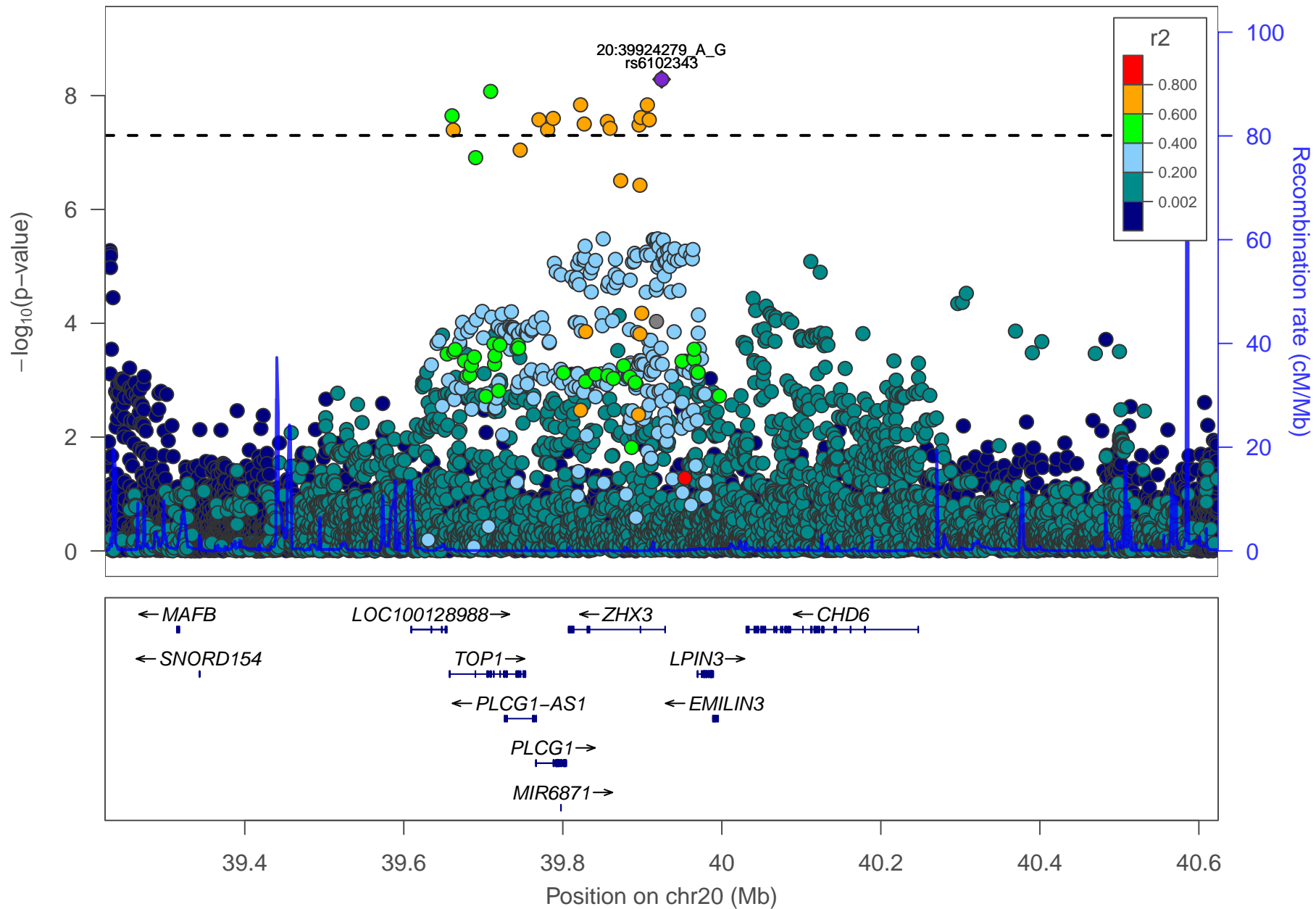

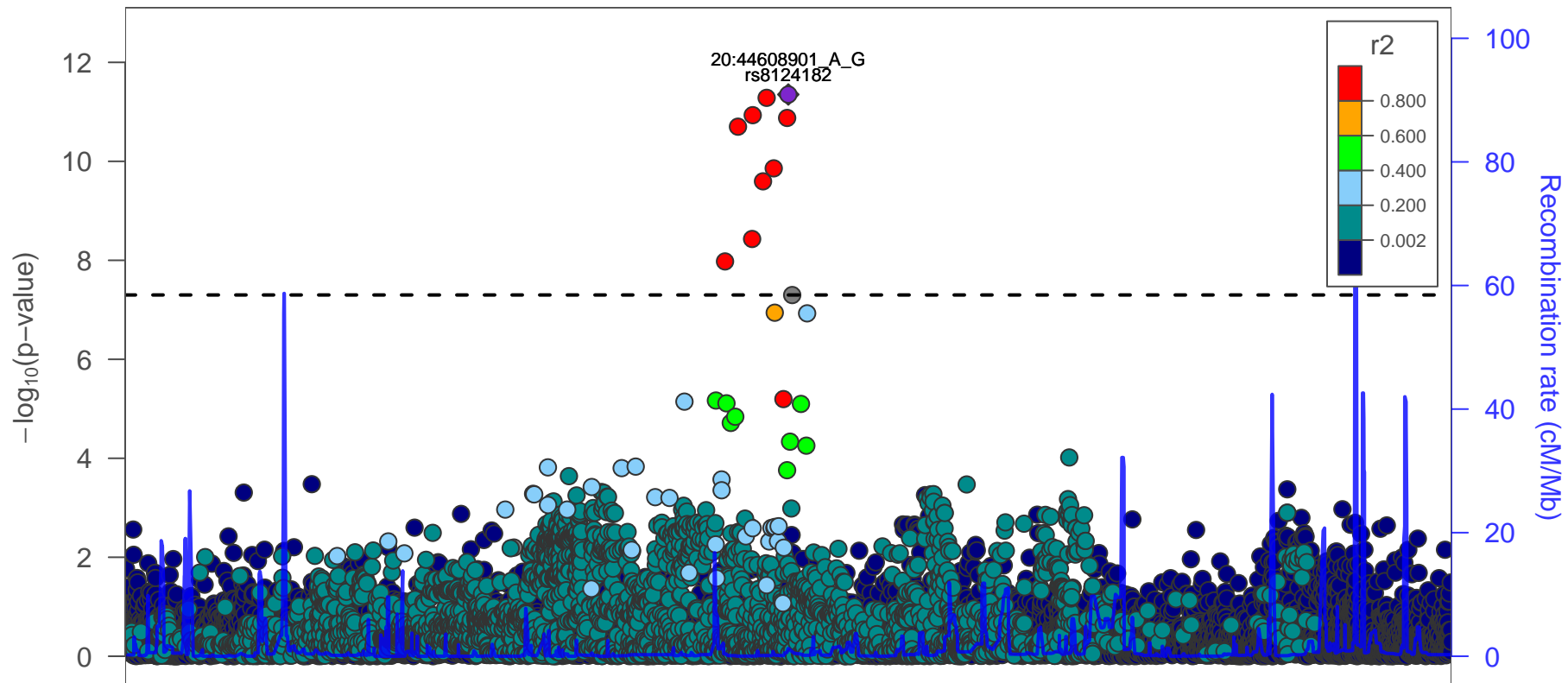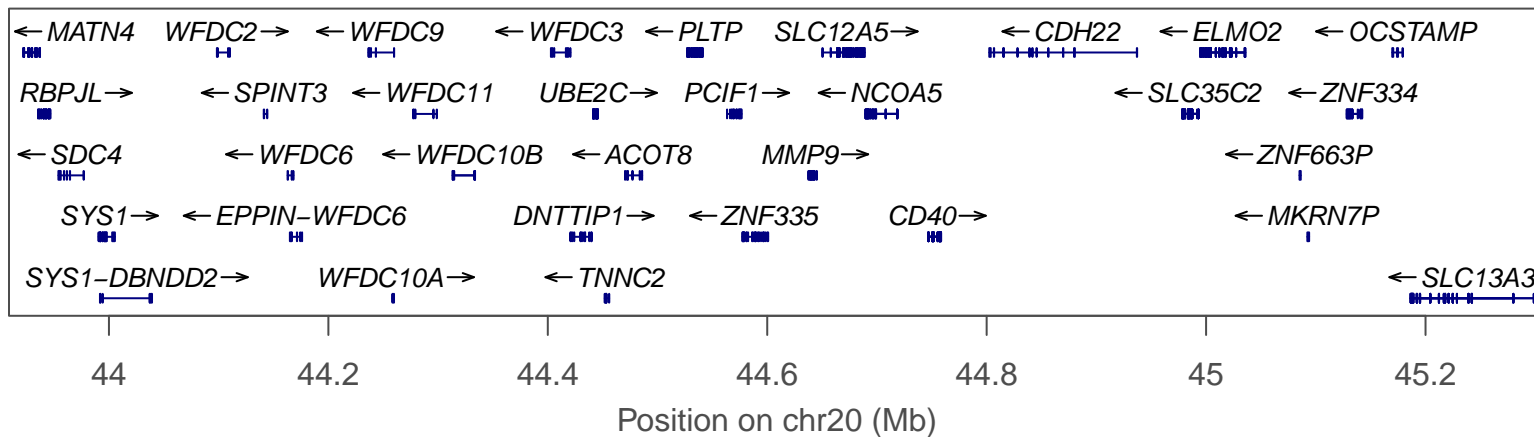

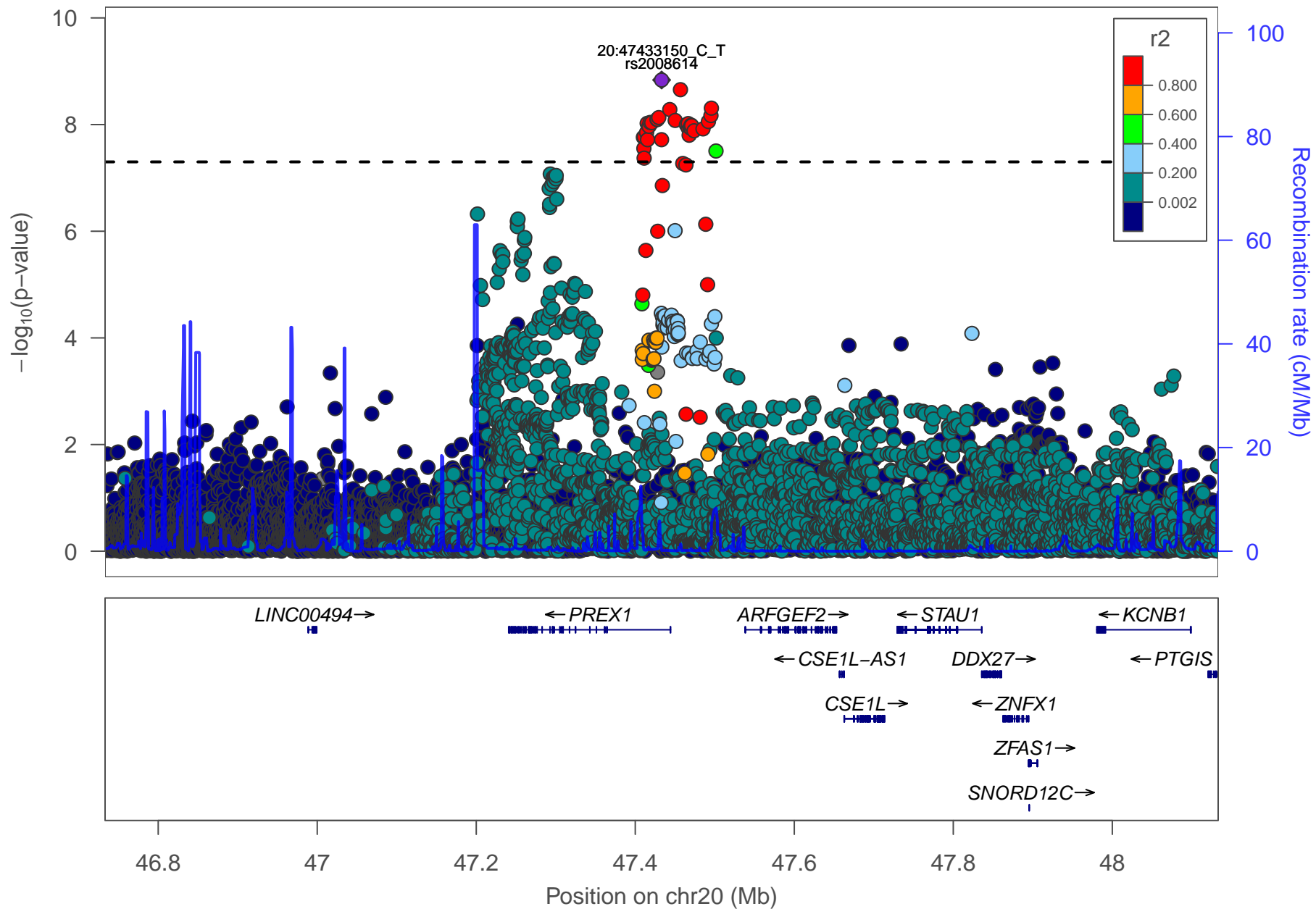

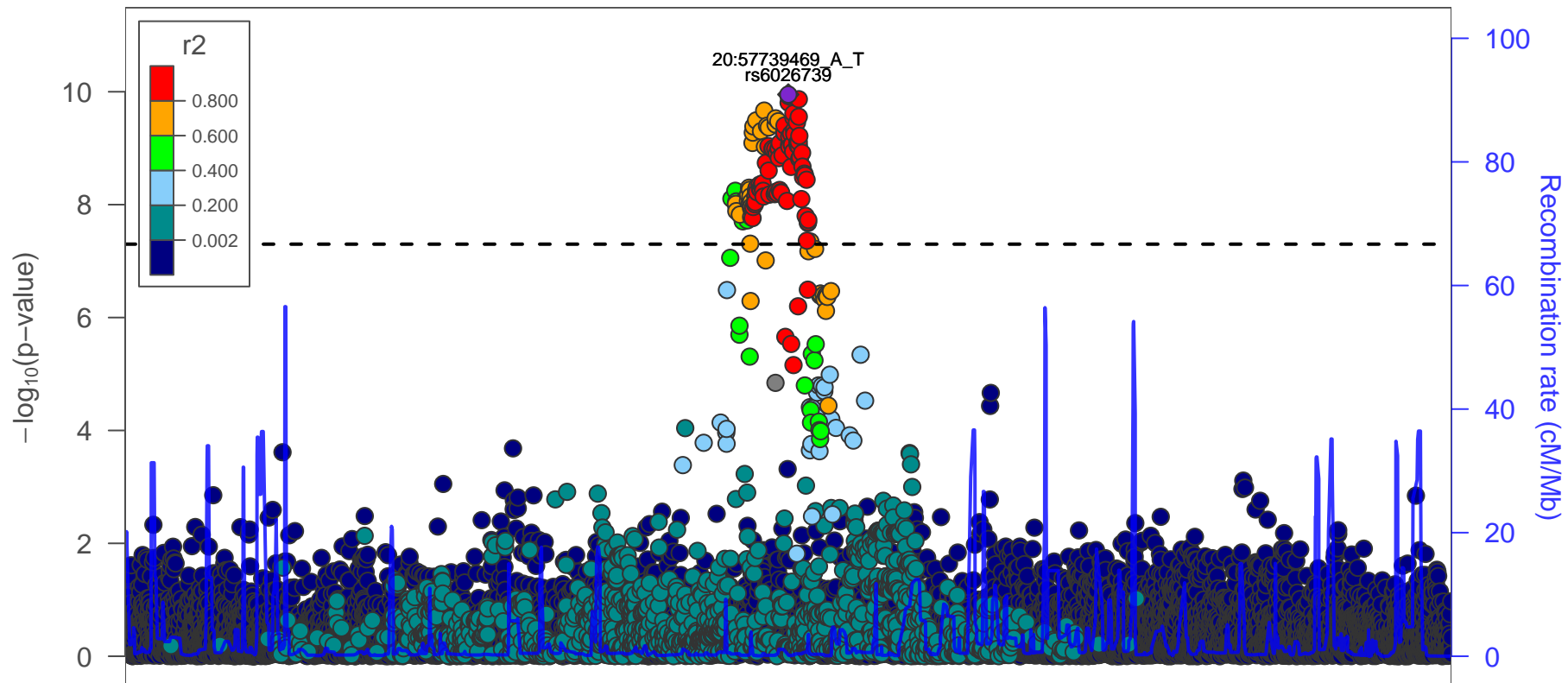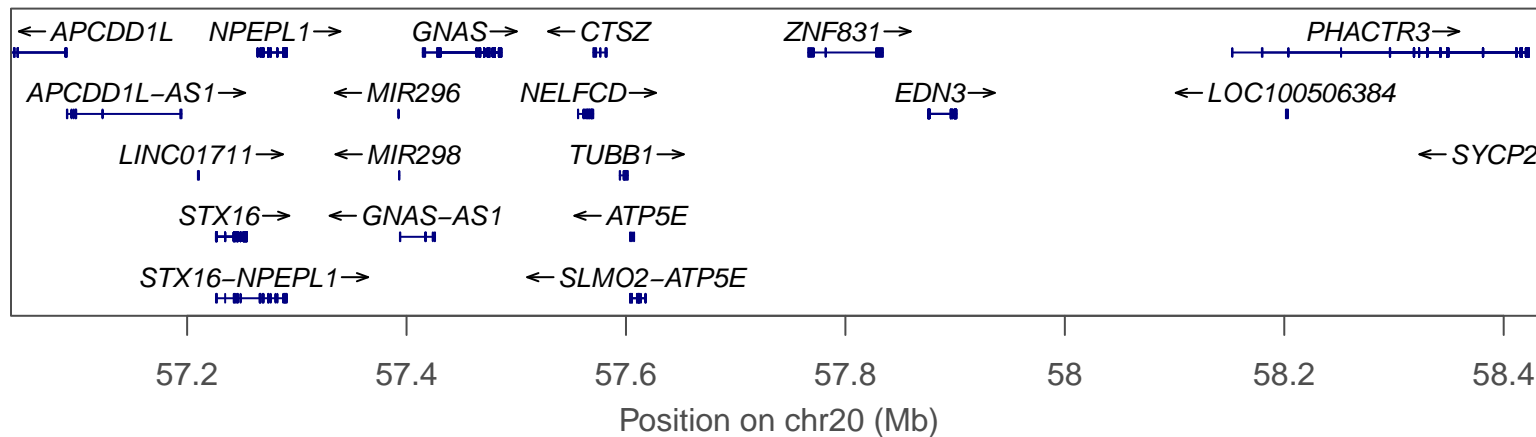

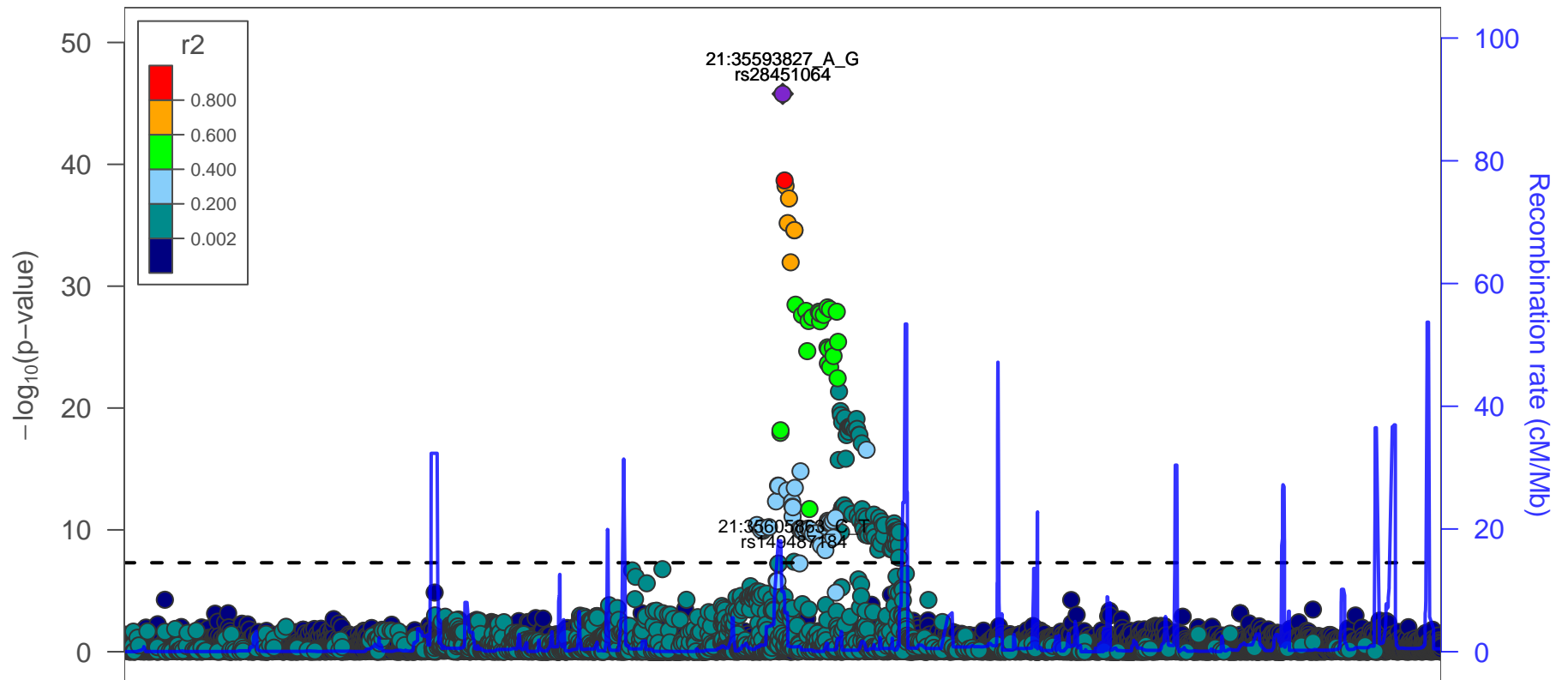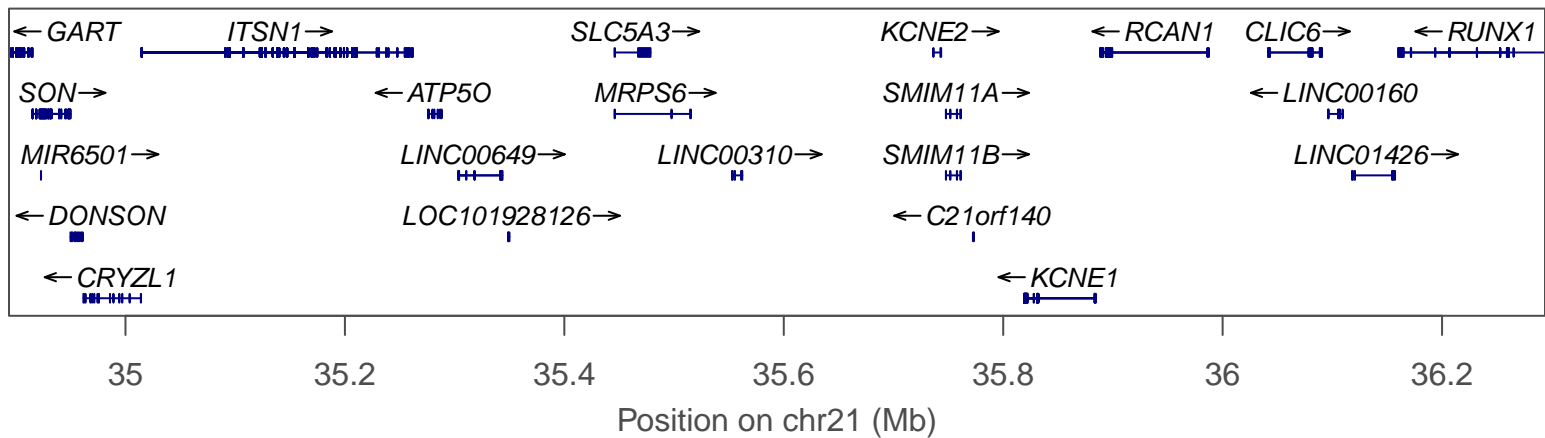

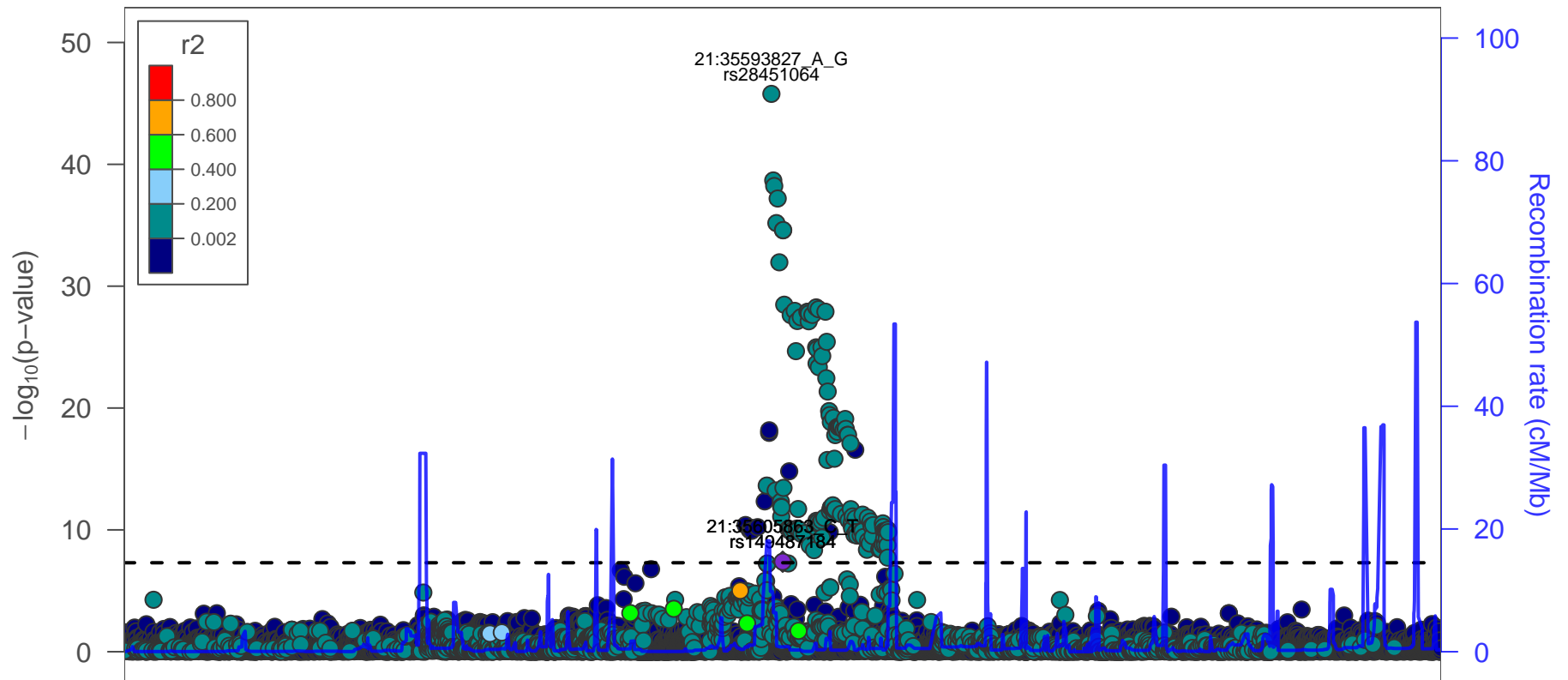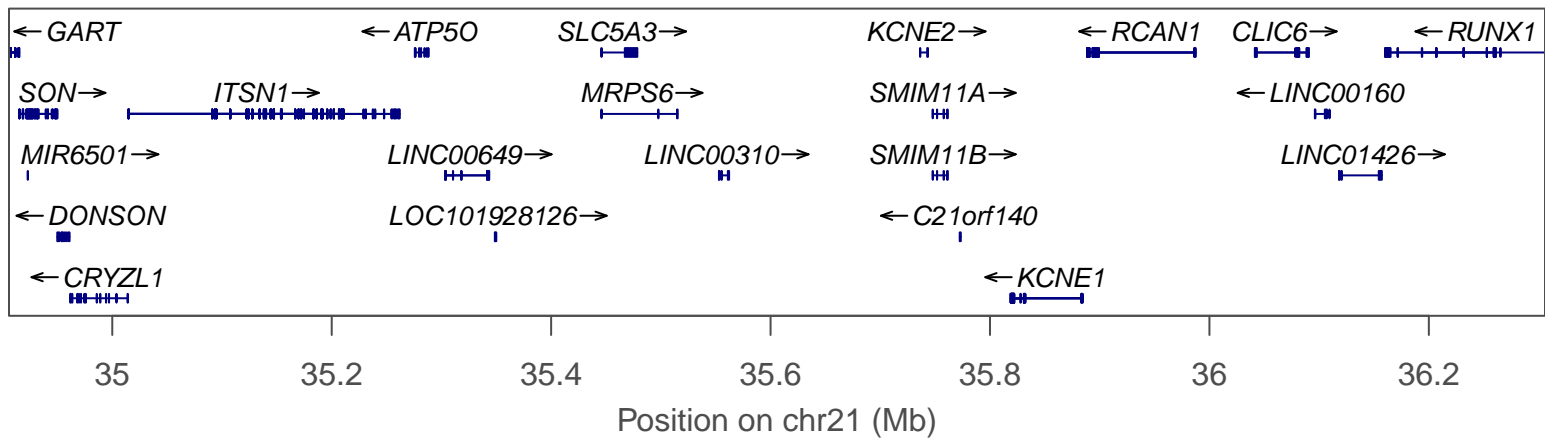

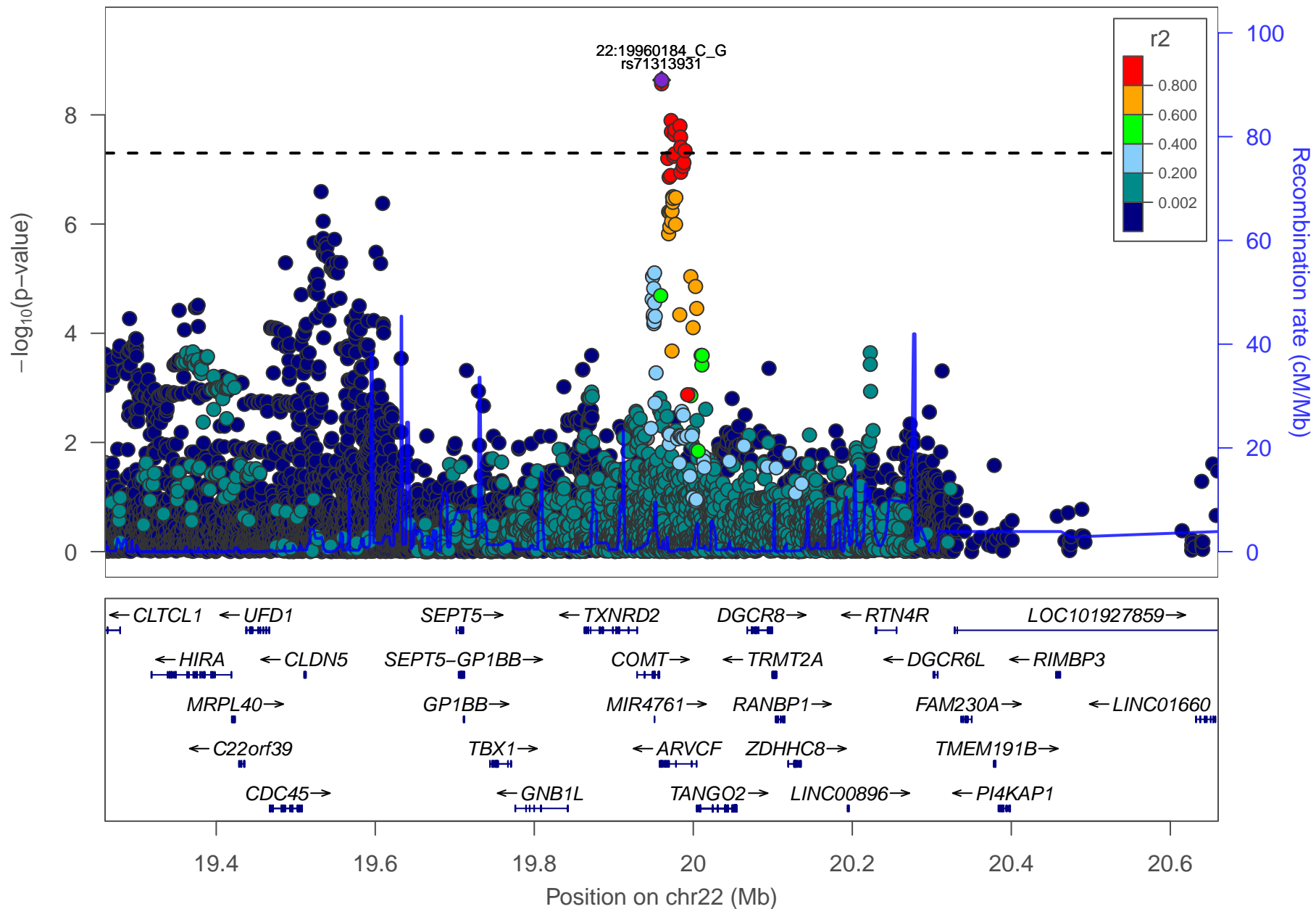

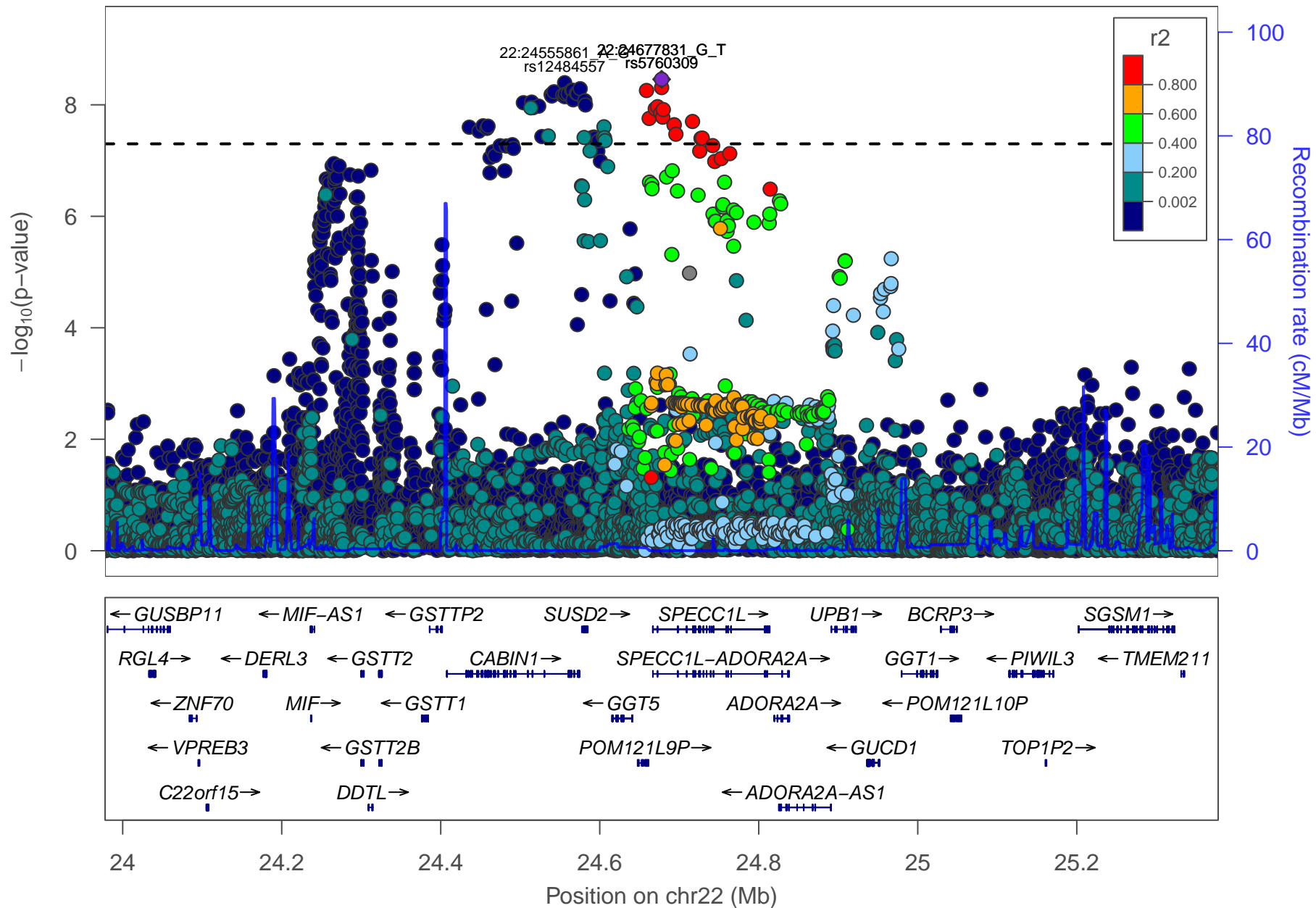

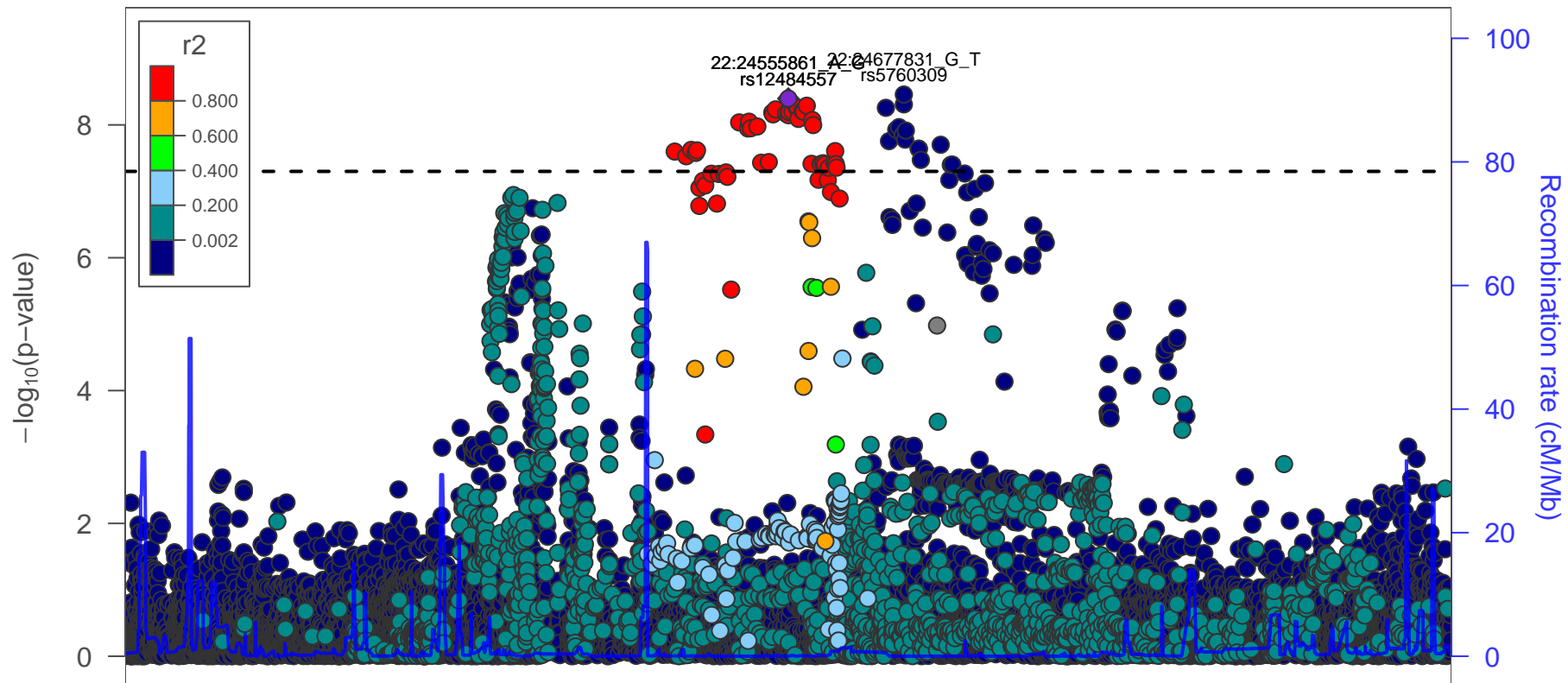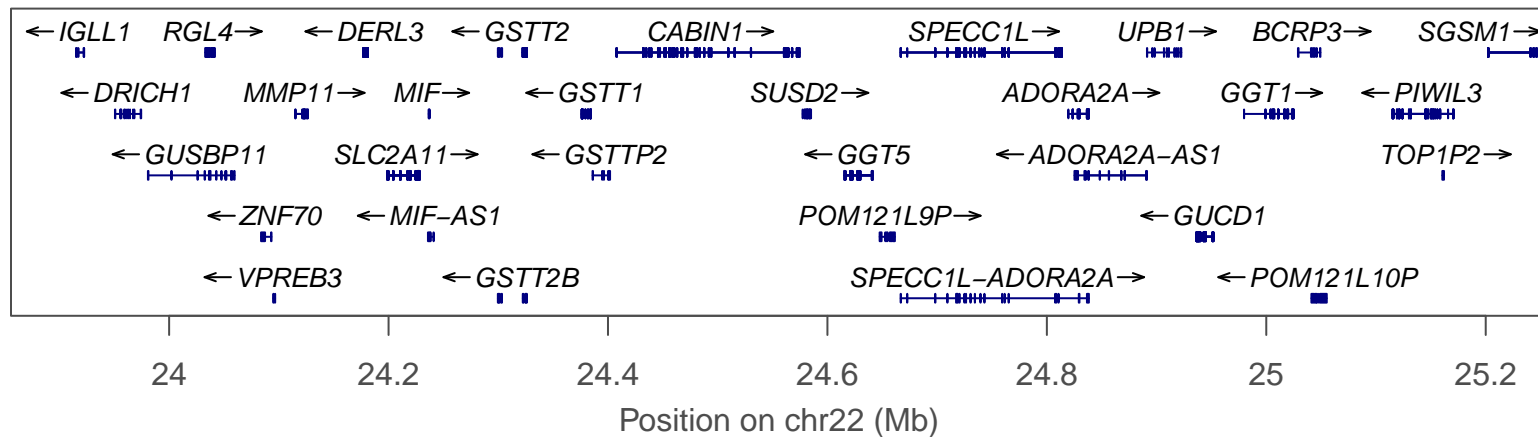

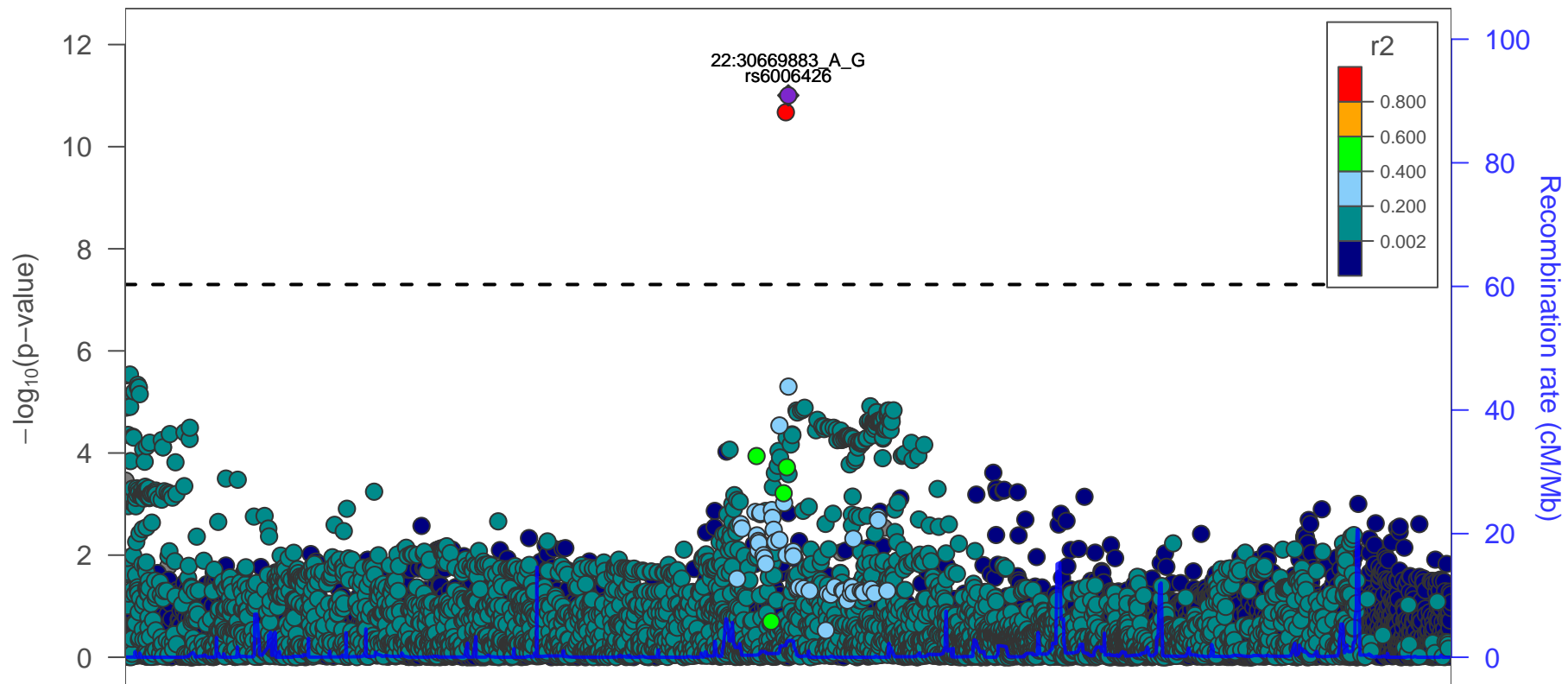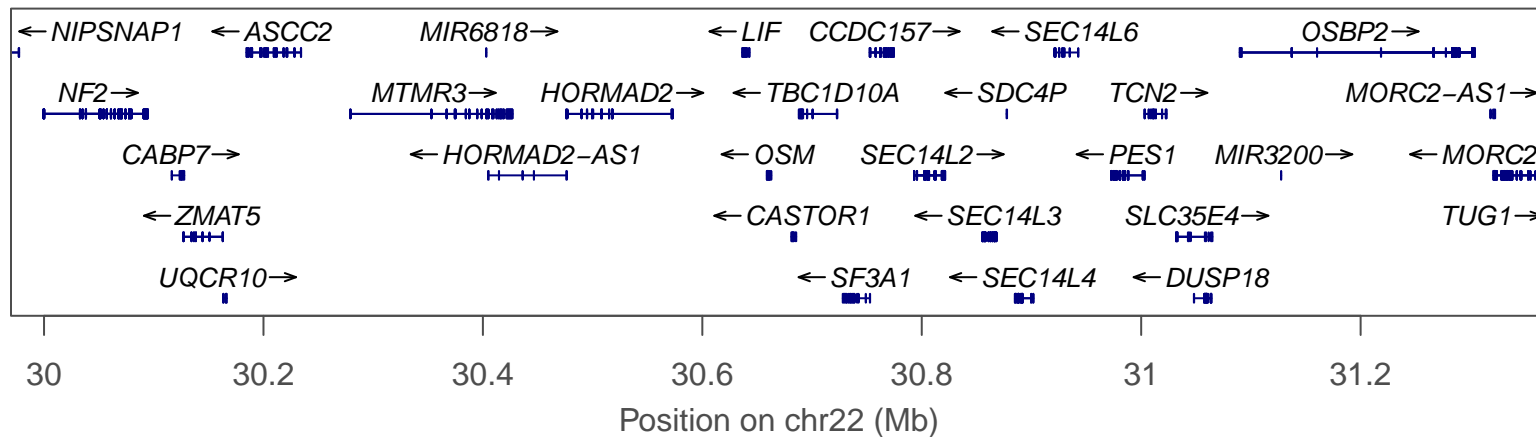

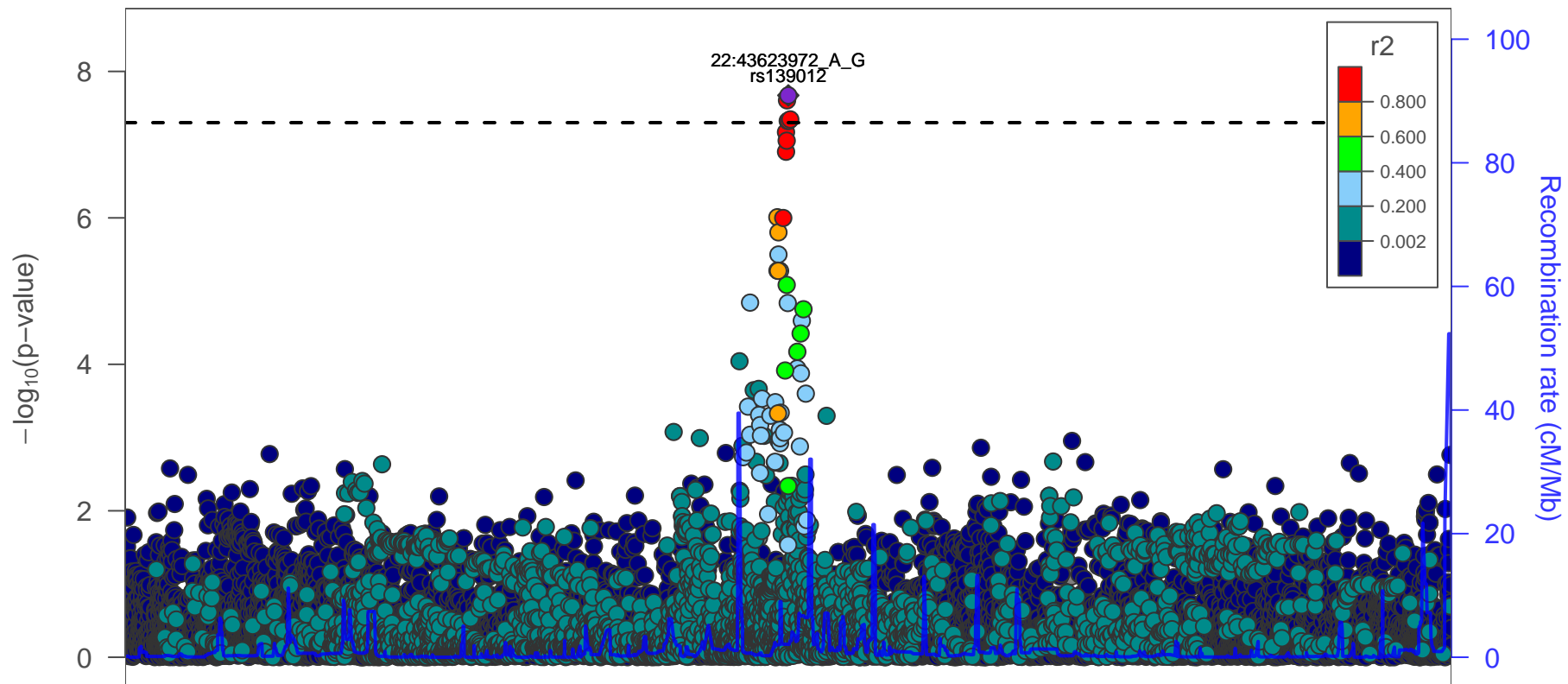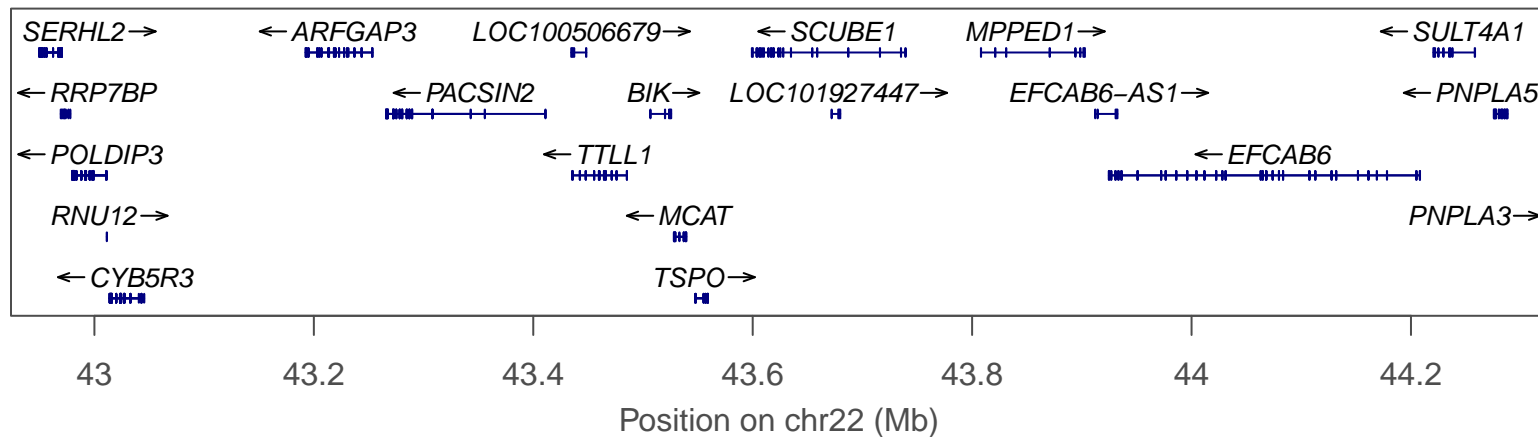

Supplement: Supplementary file 3 — Regional association plots for the 241 genome-wide significant signals from the primary CAD GWAS meta-analysis. [file 41588_2022_1233_MOESM3_ESM.pdf]
